# Cell-type-specific inhibitory circuitry from a connectomic census of mouse visual cortex

**DOI:** 10.1101/2023.01.23.525290

**Published:** 2023-01-24

**Authors:** Casey M Schneider-Mizell, Agnes Bodor, Derrick Brittain, JoAnn Buchanan, Daniel J. Bumbarger, Leila Elabbady, Daniel Kapner, Sam Kinn, Gayathri Mahalingam, Sharmishtaa Seshamani, Shelby Suckow, Marc Takeno, Russel Torres, Wenjing Yin, Sven Dorkenwald, J. Alexander Bae, Manuel A. Castro, Paul G. Fahey, Emmanouil Froudakis, Akhilesh Halageri, Zhen Jia, Chris Jordan, Nico Kemnitz, Kisuk Lee, Kai Li, Ran Lu, Thomas Macrina, Eric Mitchell, Shanka Subhra Mondal, Shang Mu, Barak Nehoran, Stelios Papadopoulos, Saumil Patel, Xaq Pitkow, Sergiy Popovych, William Silversmith, Fabian H. Sinz, Nicholas L. Turner, William Wong, Jingpeng Wu, Szi-chieh Yu, MICrONS Consortium, Jacob Reimer, Andreas Tolias, H Sebastian Seung, R Clay Reid, Forrest Collman, Nuno Maçarico da Costa

**Affiliations:** a Allen Institute for Brain Science, Seattle, WA; b Princeton Neuroscience Institute, Princeton University, NJ; c Department of Neuroscience, Baylor College of Medicine, Houston, TX; d Computer Science Department, Princeton University; e Electrical and Computer Engineering Department, Princeton University; f Center for Neuroscience and Artificial Intelligence, Baylor College of Medicine; g Department of Electrical and Computer Engineering, Rice University; h Brain & Cognitive Sciences Department, Massachusetts Institute of Technology; i Institute for Bioinformatics and Medical Informatics, University Tübingen; j Institute for Computer Science, University Göttingen; k International Max Planck Research School for Intelligent Systems, University Tübingen; l Institute of Molecular Biology and Biotechnology, Foundation for Research and Technology Hellas, Heraklion, Greece; m School of Applied and Engineering Physics, Cornell University

## Abstract

Mammalian cortex features a large diversity of neuronal cell types, each with characteristic anatomical, molecular and functional properties. Synaptic connectivity rules powerfully shape how each cell type participates in the cortical circuit, but comprehensively mapping connectivity at the resolution of distinct cell types remains difficult. Here, we used millimeter-scale volumetric electron microscopy to investigate the connectivity of inhibitory neurons across a dense neuronal population spanning all layers of mouse visual cortex with synaptic resolution. We classified all 1183 excitatory neurons within a 100 micron column into anatomical subclasses using quantitative morphological and synapse features based on full dendritic reconstructions, finding both familiar subclasses corresponding to axonal projections and novel intralaminar distinctions based on synaptic properties. To relate these subclasses to single-cell connectivity, we reconstructed all 164 inhibitory interneurons in the same column, producing a wiring diagram of inhibition with more than 70,000 synapses. We found widespread cell-type-specific inhibition, including interneurons selectively targeting certain excitatory subpopulations among spatially intermingled neurons in layer 2/3, layer 5, and layer 6. Globally, inhibitory connectivity was organized into “motif groups,” heterogeneous collections of cells that collectively target both perisomatic and dendritic compartments of the same combinations of excitatory subtypes. We also discovered a novel category of disinhibitory-specialist interneurons that preferentially targets basket cells. Collectively, our analysis revealed new organizing principles for cortical inhibition and will serve as a powerful foundation for linking modern multimodal neuronal atlases with the cortical wiring diagram.

## Introduction

In mammalian cortex, information processing involves a diverse population of neurons distributed across six layers in an arrangement described as a cortical column^1–4^. The function of this circuit depends on not only the properties of cells individually, but also the network of synaptic connectivity through which they interact. The concept of cell types has become central to understanding how this network is organized^5^. Originally classified on the basis of morphology^6^, cortical cell types have more recently been characterized also by molecular, electrophysiological, and functional properties believed to subserve specialized computational roles^7–17^. There are thus a variety of powerful methods to define cell types, but determining how — if at all — these definitions reflect differences in cortical connectivity remains difficult.

Inhibitory neurons, despite making up little more than 1 in 10 cortical neurons^18^, have at least as much cell type diversity as vastly more numerous excitatory neurons^11,13,19^, offering the potential for highly selective control of cortical activity. However, most of our understanding of inhibitory connectivity is based not on individual cell types, but on a few coarse molecular subclasses^20–22^ with certain shared developmental, functional, and synaptic properties. Parvalbumin (PV)-expressing neurons target the perisomatic region of excitatory cells, including basket cells that target the soma and proximal dendrites of excitatory neurons and chandelier cells that target the axon initial segment. Additionally, PV basket cells synapse with one another^23,24^ and other basket cells^25^. Somatostatin (SST)-expressing neurons, such as Martinotti cells, target the distal and apical dendrites of excitatory cells and also inhibit non-SST inhibitory subclasses. The connectivity of Vasointenstinal protein (VIP)-expressing neurons is heterogeneous, including not only a subclass of disinhibitory specialists that preferentially target SST cells^24,26^, but also excitatory-targeting small basket cells that coexpress Cholecystokinin (CCK)^27–29^. A fourth subclass expresses Id2^22^, spanning *lamp5* and *sncg* transcriptomic groups^19,21^, and includes neurogliaform cells with diffuse synaptic outputs and a variety of cell types in layer 1. However, within these coarse subclasses, individual cell types are highly diverse^11,12,30^ and functionally distinct^17^ but in most cases little is known about connectivity at that level.

Moreover, the structure of how inhibition is distributed across different excitatory cells is still a matter of debate, despite being a key determinant of how cortical activity is controlled. While some studies have observed largely unspecific connectivity onto nearby cells^31,32^, other studies have found examples of selective targeting of certain subpopulations of excitatory cells based on their layer position^33^ or long-range axonal projection target^34–36^. It is not known whether such selectivity is common or rare relative to unspecific connectivity. Likewise, many other basic questions remain unclear, for example what subclasses of excitatory neurons receive inhibition from the same interneurons, and do neurons targeting the perisomatic compartment and neurons targeting distal dendrites have similar or different connectivity patterns to one another.

The ideal data to address such questions would capture the synaptic connectivity of individual interneurons across a broad landscape of potential targets. However, physiological^37,38^ or viral^39^ approaches to measuring connectivity are still challenging to scale to the full diversity of potential cell type interactions. In smaller model organisms like *C. elegans*^40^ and the fly^41,42^, dense reconstruction using large-scale electron microscopy (EM) has been instrumental for discovering cell types and their connectivity. In mammalian cortex, technical limitations on EM volume sizes have meant that similar studies could not examine complete neuronal arbors, making the link between cellular morphology and connectivity difficult to address^43–47^. However, recent advances in data generation and machine learning^48–50^ have enabled the acquisition and dense segmentation of EM datasets at the scale of a cubic millimeter, making circuit-scale cortical EM volumes now possible^44,51^.

In this study, we use a millimeter-scale EM volume of mouse primary visual cortex^51^ (VISp) to reconstruct anatomy and synaptic connectivity for a dense population of 1352 neurons in a column spanning from the layer 1 to white matter (WM). The scale of this data, combined with the resolution provided by EM, led us to address the fundamental question of how the morphological cell types of the neocortex relate to the synaptic connectivity of inhibitory neurons. By a columnar population of neurons, we capture a wide range of excitatory and inhibitory subclasses and extensively sample inhibitory connectivity, which is typically most dense around nearby cells^31,38^. We used morphology and synaptic features to link a data-driven characterization of anatomical diversity among excitatory neurons to single-cell connectivity, finding widespread selectivity among inhibitory neurons. Further, our results suggest a new principle underlying inhibitory connectivity, wherein heterogeneous groups of perisomatic and dendrite targeting cells collectively target specific combinations of excitatory cell types.

## Results

### A millimeter-scale reconstruction of visual cortex with synaptic resolution.

**Figure 1.**
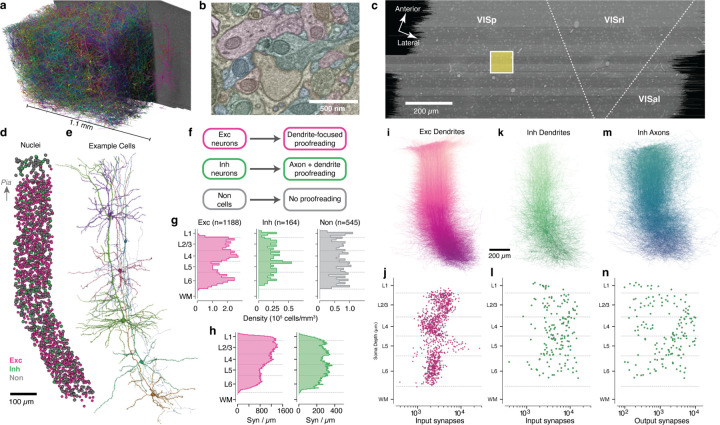
A columnar reconstruction of mouse visual cortex. **a)** The millimeter-scale EM volume is large enough to capture complete dendrites of cells across all layers. Neurons shown are a random subset of the volume, with a single example at right for clarity. **b)** The autosegmented EM data reveals ultrastructural features such as membranes, synapses, and mitochondria. **c)** Top view of EM data with approximate regional boundaries indicated. The yellow box indicates the 100*µm ×* 100*µm* column of interest. **d)** All nuclei within the column colored by cell class. **e)** Example neurons from along the column. Note that anatomical continuity required adding a slant in deeper layers. **f)** Proofreading workflow by cell class. **g)** Cell density for column cells along cortical depth by cell class. **h)** Input synapse count per *µm* of depth across all excitatory (purple) and inhibitory (green) column cells along cortical depth by target neuronal cell class. **i)** All excitatory dendrites, with arbors of cells with deeper somata colored darker. Same orientation as in **d**. **j)** Number of input synapses for each excitatory neuron as a function of soma depth. **k)** As in **j**, but for inhibitory neurons. **l)** As in **k**, but for inhibitory neurons. **m)** As in **j**, but for the proofread axons of inhibitory neurons. **n)** As in **k**, but for number of synaptic outputs on inhibitory neuron axons.

In order to measure synaptic connectivity and neuronal anatomy within a large neuronal population, we analyzed a serial section transmission EM volume of mouse visual cortex acquired as part of the broader MICrONs project^51^. The data was imaged from pia to white matter (WM) and spanned 1.1 mm from primary visual cortex (VISp) to higher order visual areas AL and RL^52^(Fig. 1a–c). Here, we analyzed the larger of two adjacent subvolumes, which was 600 *µm* deep^51^. Importantly, these dimensions were sufficient to capture the entire dendritic arbor of typical cortical neurons (Fig. 1a) at a resolution capable of resolving ultrastructural features such as synaptic vesicles (Fig. 1b). Machine learning pipelines generated an initial autosegmentation of all cells, detected synapses and segmented nuclei^49–51^. Due to reduced alignment quality near the edge of tissue, segmentation began approximately 10*µm* from the pial surface and continued into WM. To simultaneously perform analysis and correct segmentation errors, we used a scalable, centralized proofreading platform integrated with a spatial database to dynamically query annotations such as synapses across edits^53,54^.

To generate an unbiased sample of cells across all layers, we focused on a 100×100*µm* wide column spanning pia to WM, centered on the VISp portion of the volume. This location was chosen to be distant from both dataset edges and cortical region boundaries (Fig. 1c). A total of 1886 cells had their nucleus centroid inside the column. In order to follow a continuous population of neurons, the column slanted starting in layer 5, allowing the apical dendrites of deep-layer cells to be intermingled with the cell bodies of superficial cells (Figure 1d,e; see Methods). This slanted trajectory was also followed by primary axons of superficial cells and the translaminar axons of inhibitory neurons, suggesting that the pia–WM direction is not simply orthogonal to the pial surface in deep layers, but is shared across cell classes.

### A dense neuronal population sample across all layers.

We classified all cells in the column as excitatory neurons, inhibitory neurons, or non-neuronal cells on the basis of morphology (Fig. 1d). For neurons, we generated accurate reconstructions with extensive manual proofreading – more than 46,000 edits in all (Fig. 1f), guided by computational tools to focus attention on potential error locations (see Methods). Consistent with previous reports^55^, excitatory cell densities were distinct in each layer, while both inhibitory neurons and non-neuronal cells were more uniform (Fig. 1g). The reconstructions included the locations of a total of 4,471,847 synaptic inputs across all cells. Synaptic inputs onto excitatory cell dendrites were more numerous in layers 2–4 compared to layers 5–6, while inputs onto inhibitory cells were relatively uniform across depths (Fig. 1h).

Our proofreading strategy was designed to measure the connectivity of inhibitory neurons across all possible target cell types. Proofreading of excitatory neurons focused only on dendrites (Fig. 1i) and included manual edits to extend tips and correct some false merges as well as computational filtering to exclude axonal branches falsely merged onto dendrites, a common segmentation error (see Methods). Individual excitatory cells typically had thousands of synaptic inputs, with distinct laminar differences in total synaptic input per cell (Fig. 1j). For inhibitory neurons, proofreading required both completing dendrites (Fig. 1k,l) and generating extensive axonal reconstructions (Fig. 1m,n). Typical inhibitory neurons had 10^3^–10^4^ synaptic inputs and 10^2^–10^4^ outputs, but did not show strong laminar patterns(Fig. 1l, n). Collectively, inhibitory axons had 410,807 synaptic outputs. Attempts were made to follow every major inhibitory axon branch, but for large inhibitory arbors not every tip was reconstructed to completion and axonal properties should be treated as a lower bound. Non-neuronal cells were not proofread, but as a data reference we manually labeled individual subclasses.

### Connectivity-based inhibitory subclasses.

**Figure 2.**
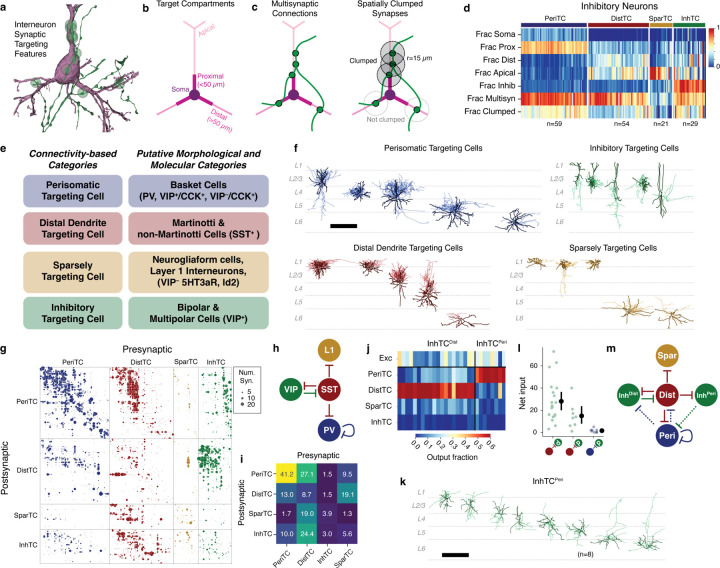
Data-driven characterization of inhibitory cell subclasses and their connectivity with one another. **a)** For determining inhibitory cell subclasses, connectivity properties were used such as an axon (green dots) making synapses (green dots) the perisomatic region of a target pyramidal cell (purple). **b)** Dendritic compartment definitions for excitatory neurons. **c)** Distribution of targets of column inhibitory cells as a function of soma depth for excitatory compartments and inhibitory neurons. **d)** Inhibitory synaptic targeting features for all inhibitory neurons, measured as fraction of synapses onto column cells, sorted by target subclass. **e)** Relationship between anatomical connectivity categories and classical cell categories that mixing molecular and morphological information. **f)** Anatomical examples of the four inhibitory subclasses. Dendrite is darker, axon is lighter. Scale bar is 500 *µm* . **g)** Inhibition of inhibition connectivity matrix. Each dot represents a connection from a presynaptic to a postsynaptic cell, with dot size proportional to synapse count. Dots are colored by presynaptic subclass and ordered by subclass, connectivity group (see Figure 5), and soma depth. **h)** Conventional model of inhibition of inhibition based on molecular subclasses. **i)** Heatmap showing the average total number of synaptic inputs a postsynaptic cell receives from cells of a given presynaptic subclass. **j)** Heatmap showing the fraction of synaptic outputs each InhTC places onto cells of other subclasses. InhTCs are clustered into two subtypes, one that targets DistTCs (InhTC^dist^) and one that targets PeriTCs (InhTC^peri^s). **k)** Morphology of all InhTC^peri^s. Scale bar is 500 *µm* . **l)** Number of synapses received by InhTC^dist^s and InhTC^peri^s from DistTCs (red dot) and PeriTCs (blue dot). **m)** Connectivity diagram for InhTC^peri^s suggested by data.

Molecular expression has emerged as a powerful organizing principle for inhibitory neurons, with four major subclasses having distinct connectivity rules, synaptic dynamics, and developmental origins^20^. However, EM data has no direct molecular information, and no simple rules map morphology to molecular identity. Classical neuroanatomical studies often used the postsynaptic compartments targeted by an inhibitory neuron as a key feature of its subclass^29,56,57^, for example distinguishing soma-targeting basket cells from soma-avoiding Martonotti cells. To classify inhibitory neurons, we thus considered purely anatomical measures of how they target other neurons (Fig. 2a).

For all excitatory neurons, we divided the dendritic arbor into four compartments: soma, proximal dendrite (<50*µm* from the soma), apical dendrite, and distal basal dendrite (Fig. 2b) (Extended Data Fig. 1) (see Methods). Inhibitory dendrites were treated as a fifth compartment. For each inhibitory neuron we measured the distribution of synaptic outputs across compartments (Fig. 2c). To further capture targeting properties, we devised two measures of how a cell distributes multiple synapses onto an individual target: 1) the fraction of all synapses that were part of a multisynaptic connection and 2) the fraction of synapses in multisynaptic connections that were also close together in space (“clumped”), defined as being within 15*µm* along the axonal arbor of another synapse in the same connection (Fig. 2d). Here, we use the term “connection” to indicate a pre- and postsynaptic pair of cells connected by one or more distinct synapses, and “multisynaptic connection” for a connection with at least two synapses. We trained a linear classifier based on expert annotations of four major subclasses for a subset of inhibitory neurons and applied it to the targeting data for all cells (Fig. 2d), (Extended Data Fig. 2).

We named each subclass based on its dominant anatomical property: perisomatic targeting cells (PeriTC) that target soma or proximal dendrites, distal dendrite targeting cells (DistTC) that primarily target distal basal or apical dendrites, sparsely targeting cells (SparTC) that make few multisynaptic connections, and inhibitory targeting cells (InhTC) that target other inhibitory neurons. Each subclass approximately corresponds to coarse classical or molecular subclasses (Fig. 2e), but is not always a one-to-one match. For example, PeriTCs would include soma-targeting cells from multiple molecular subclasses (e.g. both PV and CCK+ basket cells)^20,27,58^ and DistTCs would include both Martinotti and non-Martinotti SST cells. The SparTC subclass included neurogliaform cells and all layer 1 interneurons, suggesting it contained cells from the *lamp5* and *sncg* transcriptomic types^12^.

Some established cell types such as chandelier cells^59^ had no examples in the column, and other cells in the column did not fall cleanly into classical categories. For example, among PeriTCs we found a continuum of the degree to which cells targeted soma versus proximal dendrites (Extended Data Fig. 3). We found some PeriTCs with very few synapses onto somata but many onto proximal dendrites, a feature inconsistent with the standard definition of a basket cell^27^. Among DistTCs, we find a similar continuum of the degree to which cells target basal dendrites versus apical dendrites (Extended Data Fig. 3). One particularly striking upper layer cell with a horsetail axon almost exclusively targeted deep layer apical dendrites (Cell ID: 292721).

### Inhibition of inhibitory neurons.

The column data offered a detailed view into the inhibition of inhibition, containing 8,676 synapses between pairs of inhibitory neurons across 3,413 distinct connections (Fig. 2g). Numerous studies have described a standard connectivity pattern between inhibitory subclasses^24,26,60^ (Fig. 2h). Indeed, aggregated by subclass, the five strongest connections measured from the EM reconstructions (Fig. 2i) exactly match those found in the standard circuit model (Fig. 2h), based on the putative corresponding molecular subclasses (Fig. 2e). This suggests that both the EM reconstructions and the subclass identification are consistent with known connectivity.

However, the data revealed novel connectivity patterns at the level of individual cells (Extended Data Fig. 4). VIP neurons comprise the main population of disinhibitory specialists, where they have been described as primarily targeting SST cells^24,26,38,61^, which would generally correspond here to DistTCs. To measure if this connectivity applied for all cells, for each InhTC we computed its distribution of synaptic outputs across inhibitory subclasses (Fig. 2i). Surprisingly, we found two distinct connectivity-based populations of InhTCs (Fig. 2j): an expected one targeting DistTCs (InhTC^Dist^) and an unexpected one targeting PeriTCs (InhTC^Peri^) (see Methods). Morphologically, InhTC^Dist^ had bipolar dendrites consistent with typical VIP neurons and were concentrated in layers 2–4 (Extended Data Fig. 5), while InhTCs^Peri^ had multipolar dendrites and were distributed across all layers (Fig. 2k).

We next looked at the inhibitory inputs into InhTC^Peri^. Like InhTC^Dist^, InhTC^Peri^ received numerous inhibitory inputs from DistTCs (Fig. 2l). However, we found few reciprocal synapses from PeriTCs back onto InhTC^Peri^s (Fig. 2l). Thus unlike the competitive inhibition between InhTC^Dist^ and DistTCs, InhTC^Peri^ are instead part of a feed-forward pathway, where the inhibition of PeriTCs can be inhibited by DistTCs (Fig. 2m).

Single-neuron consideration of the InhTC^Dist^ subclass also revealed striking laminar differences in connectivity (Extended Data Fig. 5). We found that InhTC^Dist^ in layers 2–4 targeted DistTCs in layers 4–5, but not DistTCs in layer 2/3. Similarly, those DistTCs in layer 2/3 made few synapses onto InhTC^Dist^s in return. Interestingly, those layer 2/3 DistTCs preferentially target excitatory neurons in upper but not lower layer 2/3, suggesting differential InhTC-mediated disinhibition within layer 2/3.

Taken together, on average the EM data recapitulated the well-known connectivity patterns of molecularly-defined inhibitory subclasses, but dense sampling of single-cell connectivity revealed new features of cortical disinhibition.

### Synaptic properties help define excitatory subclasses.

While inhibitory neurons have frequently been described as having dense, unspecific connectivity onto nearby neurons^31,62^, many studies have revealed examples not only of layer-specific connectivity^63^, but also selectivity within spatially intermingled excitatory subpopulations^35,36,64^. It is unclear the degree to which inhibition is specific or not, and, in general, the principles underlying which excitatory neurons are inhibited by which inhibitory neurons is not well understood.

**Figure 3.**
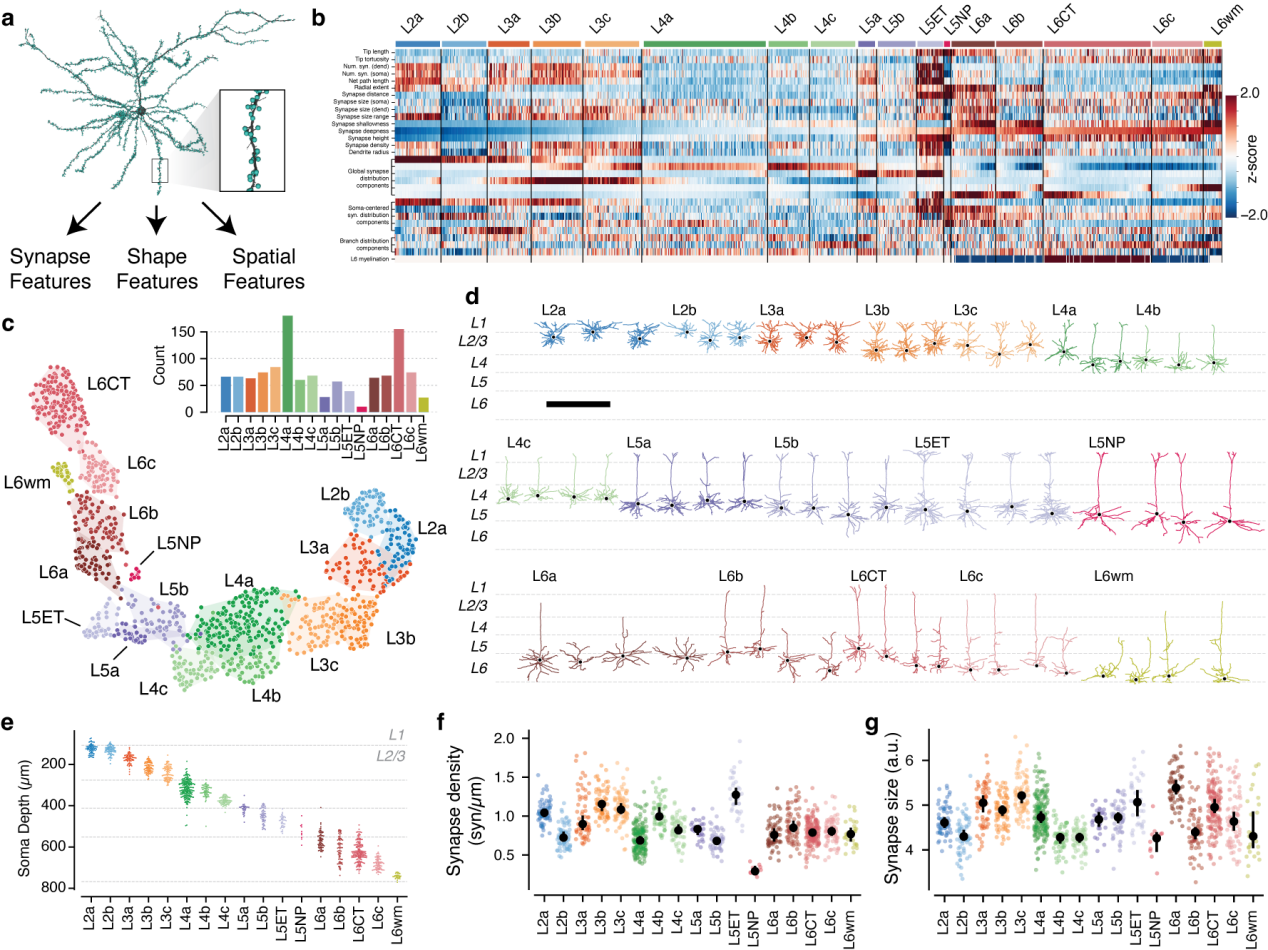
Data-driven characterization of excitatory neuron morphological types (M-types). **a)** Morphology (black) and synapse (cyan dots) properties were used to extract features for each excitatory neuron, such as this layer 2/3 pyramidal cell shown. **b)** Heatmap of z-scored feature values for all excitatory neurons, ordered by anatomical cluster (see text) and soma depth. See Methods for detailed feature descriptions. **c)** UMAP projection of neuron features colored by anatomical cluster. Inset indicates number of cells per cluster. **d)** Example morphologies for each cluster. See (Extended Data Fig. 6) for all excitatory neurons. Scale bar is 500 *µm* . **e)** Soma depth of cells in each anatomical cluster. **f)** Median linear density of input synapses across dendrites by M-type. **g)** Median synapse size (arbitrary units, see Methods). In **g** and **h**, colored dots indicate single cells, black dots and error bars indicate a bootstrapped (n=1000) estimate of the median and 95% confidence interval.

To address these questions, we first needed to characterize excitatory neurons subclasses in the EM data. Previous approaches to data-driven clustering of excitatory neuron morphology could only use information about the skeleton^13,14,16,65^, but the data here also has the location and size of all synaptic inputs (Fig. 3a). We reasoned that using synaptic features in addition to skeleton features would better represent the landscape of excitatory neurons since they directly measure how neurons interact with one another. We assembled a suite of 25 features to describe each cell, including synapse properties like median synapse size, skeleton qualities such as total branch length, and spatial properties characterizing the distribution of synapses with depth (Fig. 3b) (Extended Data Fig. 7), (Extended Data Fig. 8) (see Methods). We performed unsupervised clustering of these features (Fig. 3b–d), identifying 17 “morphological types” or “M-types”. Briefly, we repeatedly applied a graph-based clustering algorithm^66^ on subsets of the data to compute a matrix of co-clustering frequency between cells, followed by agglomerative clustering to obtain a consensus result (see Methods).

To relate this landscape to known cell types, expert neuroanatomists labeled cells by the layer of the cell body and long-range projection type (IT: inter-telencephalic or inter-cortical; ET: extra-telenceophalic or subcortical projecting, NP: near projecting, and CT: Corticothalamic)^67^. M-types were named by the dominant expert label (Extended Data Fig. 9), with IT M-types within the same layer being ordered by average soma depth. For clarity, we use the letter “L” in the name of M-types (which may include some cells outside the given layer) and the word “layer” to refer to a spatial region. Interestingly, upper and lower layer 2/3 emerged as having distinct clusters, which we denoted “L2” and “L3” respectively.

Each layer contained multiple M-types (Fig. 3e), some spatially intermingled (e.g. L2a/L2b and L5b/L5ET) and others separating into subdomains within layer (e.g. L4b/L4c and L6a–c). Most M-types had visually distinguishable characteristics, consistent with previous studies^13,14,16^ (Fig. 3d) (Extended Data Fig. 6), but in some cases subtle differences in skeleton features were accentuated by synaptic properties. For example, the two layer 2 M-types are visually similar, although L2a had a 29% higher overall dendritic length (L2a, 4532 *µm* ; L2b, 3510 *µm* ) (Fig. 7.) However, L2a cells had 80% more synaptic inputs than L2b cells (L2a, 4758; L2b, 2649), a 40% higher median synapse density (L2a, 1.04 syn/*µm* ; L2b, 0.72 syn/*µm* ) (Fig. 3f,g), and even a wider distribution of synapse sizes (Extended Data Fig. 7). Median synapse size turned out to differ across M-types, often matching layer transitions (Fig. 3g). Strikingly, L5NP cells were outliers across all synaptic properties, with the fewest total dendritic inputs, lowest synaptic input density, and small synapses (Fig. 3g,h).

Based on this analysis, excitatory M-types differ not only in morphology, but also in both cell-level synaptic properties like total synaptic input and local synapse properties like density and median size.

### Coordination of inhibition across excitatory M-types.

While excitatory M-types may have different properties, this may or may not be meaningful with regards to cortical circuitry. One piece of evidence that they do matter would be if they have different sources of local inhibition. Having classified inhibitory subclasses and excitatory M-types, we thus analyzed how inhibition is distributed across the landscape of excitatory neurons.

**Figure 4.**
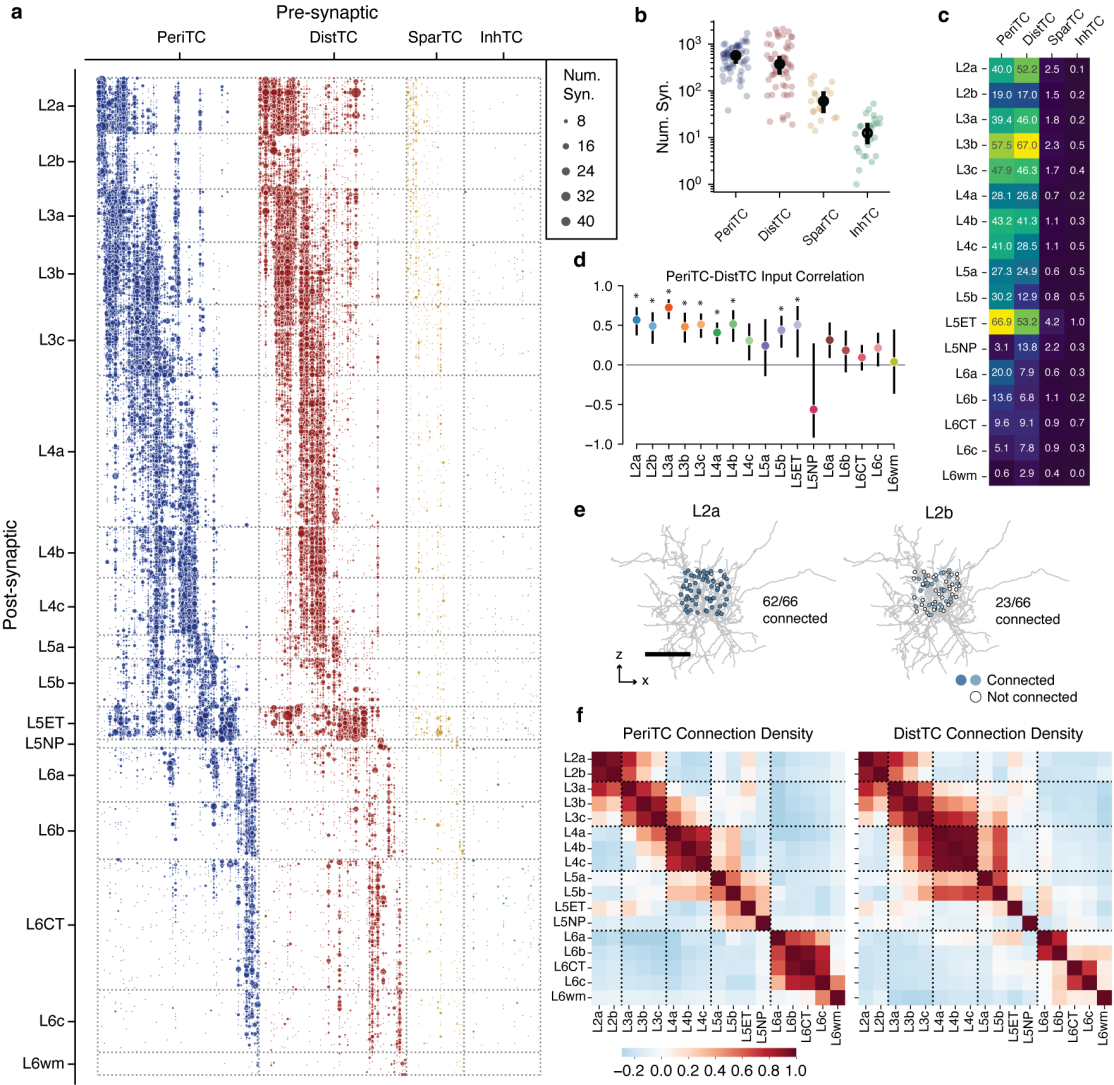
Inhibition of excitatory neurons. **a)** Connectivity from all inhibitory neurons (columns) onto all excitatory neurons, sorted by M-type and soma depth. Dot size indicates net number of synapses observed. **b)** Net synapses onto column cells for each inhibitory subclass. Black dots indicate median, bars show 5% confidence interval. **c)** Mean net synapses per target cell from each inhibitory subclass onto each excitatory M-type. **d)** Spearman correlation of PeriTC and DistTC net input onto individual cells, measured within each M-type. Bars indicate 95% confidence interval based on bootstrapping (n=2000). Stars indicate M-types significantly different from zero with a p-value < 0.05 after Holm-Sidak multiple test correction. **e)** Example of connectivity density calculation. Connectivity density from a single interneuron (gray) onto all cells within two example M-types (left: L2a right, L2b). Potential target cell body positions shown as dots, filled if synaptically connected and gray otherwise. Scale bar is 100*µm* . **f)** Pearson correlation of connectivity density between excitatory M-types, based on PeriTCs (left) and DistTCs (right). Dotted lines indicate layers.

The column reconstructions included 68,527 synapses from inhibitory neurons onto excitatory neurons (Fig. 4a). PeriTCs and DistTCs were by far the dominant source of inhibition, with individual cells having as many as 2,118 synapses onto excitatory cells in the column (mean PeriTC: 581 synapses; DistTC: 596 synapses), while SparTCs and InhTCs had far fewer synapses (SparTC: 74 synapses, InhTC: 16 synapses) (Fig. 4b). Inhibition was distributed unequally across M-types (Fig. 4c) but input from PeriTC and DistTC was typically in balance for each M-type. This balance between somatic and dendritic inhibition extended to individual cells. To control for potential input differences between subclasses, we examined the number of PeriTC and DistTC inputs onto individual excitatory neurons for each M-type separately. PeriTC and DistTC input was significantly positively correlated for 9/17 M-types (Fig. 4d), suggesting coordinated inhibitory input across the entire arbor of target cells.

M-types in upper layers had particularly heterogeneous net input, with L2b cells receiving 40% as many inhibitory synapses as spatially intermingled L2a cells (L2b: 37.7 ± 0.27 syn; L2a: 94.8 ± 0.58 syn), while L3b cells had as many intracolumnar inhibitory inputs as much larger L5ET cells. All layer 6 M-types had relatively few intracolumnar inputs compared to upper layers (Fig. 4c). However, note that due to the columnar sampling, this reflects only local sources of inhibition and does not eliminate the possiblity that deep layer neurons receive inhibitory input from more distant cells than in upper layers.

Individual inhibitory neurons often targeted multiple M-types, suggesting that certain cell type combinations are inhibited together. To gain insight into the structure of this co-inhibition, for each inhibitory neuron we computed the fraction of each M-type it targeted (Fig. 4e) and computed the correlation of inhibitory connection density between M-types for PeriTCs and DistTCs separately (Fig. 4f). A high correlation would indicate that the same inhibitory neurons that connected more (or less) to one M-type also connect more (or less) to another, while zero correlation would suggest independent sources of inhibition between M-types.

These correlations revealed several notable features of the structure of inhibition across layers. In superficial cortex, the layer 2 and layer 3 M-types are strongly correlated within layer, but have relatively weak correlation with one another, suggesting different sources of inhibition. Layer 4 M-types, in contrast, are highly correlated with one another. Layer 5 M-types are more complex with weak correlation with one another, suggesting largely non-overlapping sources of inhibition, particularly among neurons with different long-range projection targets. Interestingly, layer 5 IT cells share co-inhibition with layer 4 M-types. Layer 6 inhibition is virtually independent from all other layers. Importantly, these features are found within PeriTC and DistTC connectivity independently, suggesting broad coordination between inhibitory subclasses in how they distribute output across M-types.

### Cellular contributions of inhibition.

**Figure 5.**
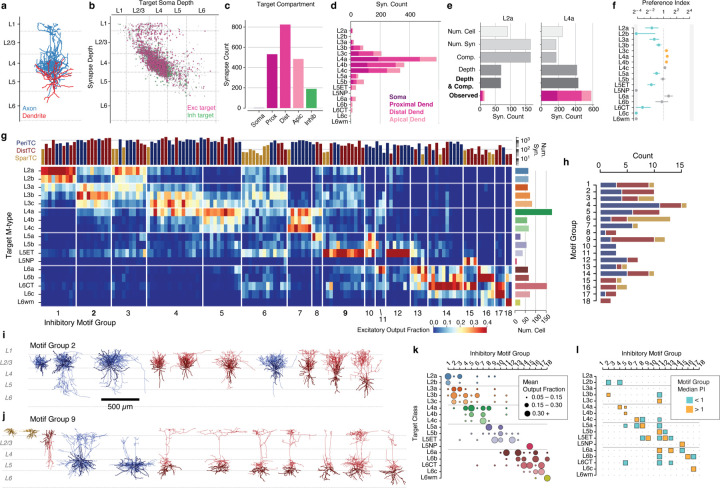
Inhibitory connectivity groups selectively organize inhibitory connectivity. **a–f)** Single-cell anatomical connectivity profiles. **a** Example inhibitory neuron. Axon in blue, dendrite in red. **b–e** Output connectivity profile of the cell in **a**. **b** Scatterplot of synaptic outputs, showing synapse depth (y-axis) vs depth of the postsynaptic soma (x-axis). Purple dots are excitatory, green dots are inhibitory, and gray dots are targets outside the column data. **c** Distribution of synaptic outputs across target compartments. **d** Synapse distribution across all potential excitatory types **e** Observed synapse count onto L2a (left) and L4a cells (right) compared to the expected value from various null models of connectivity (see text). Internal colors for observed data indicate contributions from each compartment. **f)** The preference index (PI) of each M-type for the example cell. PI is measured as the ratio of the observed synapse count to the depth and compartment-matched null model. Colored dots indicate a statistically significant deviation from a PI of 1 (orange is high, blue is low, lines indicate 95% confidence interval after Holm-Sidak correction for multiple tests), using stratified tables compared across depth bins (see Methods). **g)** Distribution of synaptic output for all interneurons, organized into common targeting motif groups. Each row is an excitatory target M-type, each column is an interneuron, and color indicates fraction of observed synapses from the interneuron onto the target M-type. Sort order is by motif group and soma depth. **h)** Cell counts in each motif group, colored by inhibitory subclass. **i)** All inhibitory cells in motif groups 1 (**g**) and 8 (**j**), colored by subclass as in **f**. Note diversity in both subclasses and individual cells. **k)** Mean fraction of synapse targeting for each motif group. **l)** Median selectivity PI within each motif group. Cells with non-significant PIs were considered to be 0.

How do individual neurons distribute their output to produce the patterns of inhibition described above? To address this, we assembled a rich collection of information about the morphology of each interneuron (Fig. 5a), synaptic connectivity (Fig. 5b), and how it distributes output across compartments (Fig. 5c) and excitatory M-types (Fig. 5d) within the column. We further attempted to quantify the degree to which the observed target distribution reflected selectivity beyond spatial overlap. This is a nuanced question, depending on both presynaptic factors such as compartment preference and bouton density and postsynaptic factors such as the amount of inhibition a given cell wants and the heterogeneity of potential targets in a particular region^68–70^. For each interneuron and each target M-type, we computed the expected number of synapses based on randomly sampling input synapses in the column, preserving the observed distribution of synapse depths and target compartments (Fig. 5e) (Extended Data Fig. 11). While this sampling includes both excitatory and inhibitory synapses, previous studies^71,72^ and our data suggest that excitatory and inhibitory inputs are proportional to one another, even at the level of individual cells (Extended Data Fig. 10). A type-specific preference index (PI) was calculated as the ratio of the observed synapse count to the expected synapse count, and statistical significance was assessed by considering the synapse distribution as a collection of stratified tables across depth bins (Fig. 5f) (see Methods).

To compare patterns of output across inhibitory neurons, for each inhibitory neuron we measured the fraction of synaptic outputs made onto each M-type. This normalized synaptic distribution reflects factors such as the number of synapses per connection and the number of potential targets, while not being strongly affected by partial axonal arbors. We performed a consensus clustering based on repeated k-means followed by agglomerative clustering (see Methods) (Fig. 5g), producing 18 “motif groups”, or sets of cells with similar patterns of output connectivity. Groups were ordered by average target soma depth. Some motif groups focused their output onto single excitatory M-types (e.g. Group 9), while others spanned broadly (e.g. Group 6). Motif groups were typically comprised of diverse combinations of PeriTCs, DistTCs, and SparTCs (Fig. 5h), suggesting that particular combinations of M-types received coordinated inhibition onto both perisomatic and dendritic compartments from cells in different inhibitory subclasses.

Averaging within motif groups, we find a collection of targeting patterns that span the excitatory population, with most M-types being the target of more than one motif group (Fig. 5k). To examine the contribution of cell type selectivity in producing these patterns, we computed at the median PI for each target M-type for each motif group (Fig. 5l). For several motif groups, cells consistently avoided or preferred to synapse onto specific M-types in order. For example, the median cell in Group 2 makes significantly fewer synapses onto L2a cells and significantly more synapses onto L3b cells than expected from a depth- and compartment-matched null model. In contrast, Group 1 almost exclusively targets L2a and L2b, but because of the narrow spatial domain of most axons in this group, the median Group 1 cell did not show additional selectivity (although see the Extended Data Cell Atlas for individual examples of selective cells). Indeed, for many neurons the tight control of their axonal extent was sufficient to produce highly specific connectivity without additional cell type selectivity. This is particularly true for PeriTCs, as the perisomatic compartments of different M-types overlap significantly less than their distal dendrites (Extended Data Fig. 11).

Taken together, our data show that cortical inhibition is comprised of groups of neurons which target specific collections of M-types with sub-laminar precision, and that these patterns result from a combination of both well-structured spatial overlap and selective connectivity.

### Inhibitory motifs across layers.

Presynaptic selectivity alone does not tell us about the importance of a connection to its postsynaptic partner. Given the biological requirements to develop and maintain structured synaptic connectivity^73^, we reasoned that, all else being equal, an inhibitory neuron is more likely have a strong impact on a target M-type if it forms connections onto a higher fraction of cells and if each individual connection has more synapses.

**Figure 6.**
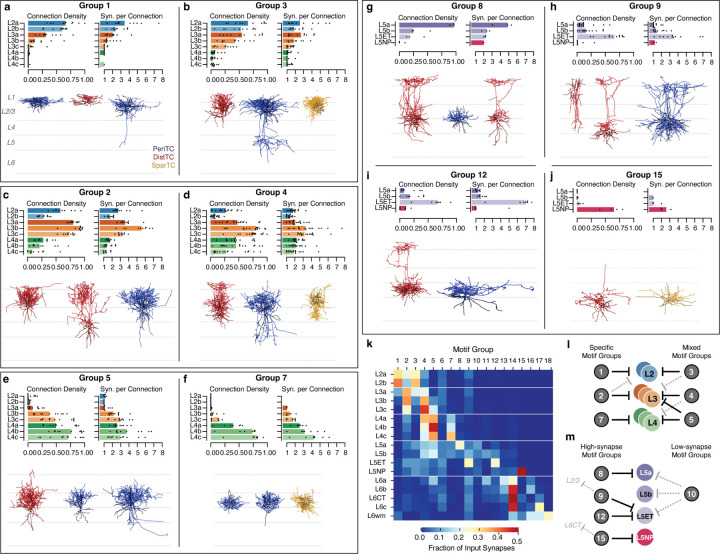
Inhibitory connectivity groups targeting layers 2–5 make specific, strong connections. **a, b)** Inhibitory motif groups targeting layer 2: Groups 1 (**a**) and 3 (**b**). **a)** Top row, left: Connection density for Group 1 measured as the fraction of cells of a target M-type that get synaptic input from each cell in the group. Bar indicates median, dots show each cell. Top row, right: Mean number of synapses per connection. Bar indicates median, dots show each cell. Only target M-types from layers 2–4 shown; see Extended Data Cell Atlas for all data. Bottom row: Example morphologies for neurons in the group, colored by cell subclass. Examples were chosen to capture diversity within groups. **b)** As in **a)**, for Group 3. **c, d)** Inhibitory motif groups targeting layer 3: Groups 2 (**c**) and 4 (**d**), as in **a,b**. **e, f)** Inhibitory motif groups targeting layer 4: Groups 5 (**e**) and f (**f**), as in **a,b**. **g, j)** Inhibitory motif groups targeting layer 5: L5a-targeting Group 8 (**g**), ET targeting Groups 9 (**h**) and 12 (**i**), and NP-targeting Group 15 (**j**), as in **a,b**. Here, only target M-types from layer 5 shown. **k)** Fraction of all measured inhibitory input into each target excitatory M-type, by motif group. **l)** Schematic of major inhibitory motif groups onto layers 2–4. **m)** Schematic of major inhibitory motif groups onto layer 5.

Based on these criteria, we found that motif groups were a strong source of inhibition for their main postsynaptic targets. Here, we summarize the dominant patterns of inhibitory connectivity. Two motif groups strongly targeted each of layer 2, 3, and 4. Among motif groups targeting layer 2, Group 1 was highly specific to layer 2 (Fig. 6a), while Group 3 also targeted layer 3 (Fig. 6b) and often even L5ET cells (Fig. 5g). Among layer 3 specific motif groups, Group 2 was specific to layer 3, while Group 4 additionally targeted layer 4 subtypes with multisynaptic connections and often very high connection densities (Fig. 6d). Interestingly, both Group 2 and 4 selectively avoided L2b but not L2a cells in terms of output fraction, connection density, and synapses per connection, suggesting that they are a source of biased inhibition within layer 2. Among layer 4 specific motif groups, Group 5 also widely targeted layer 3 cells (Fig. 6e), while Group 7 was extremely specific for layer 4, and targeted the deeper M-types with particularly high-synapse connections (Fig. 6f).

In layer 5, most inhibitory input came from four motif groups: Group 8 targeted L5a IT cells (Fig. 6g), Groups 9 and 12 targeted L5ET cells (Fig. 6h,i), and Group 15 targeted L5NP cells (Fig. 6j). The two L5ET-targeting groups had similar connectivity within layer 5, but differed in their translaminar connectivity, with several Group 9 cells sending ascending axons that targeted cells in layer 2/3 (Fig. 5g,k). We saw no group specific to L5b IT cells, although there was one cell in the data (Cell ID: 267068) that was highly selective for them, and a wider survey may find such a category. Uniquely, L5NP cells avoided inhibition from all motif groups other than the one selective for them. Interestingly, the L5NP-selective group also targets L6CTs but no other subclass, suggesting putative functional coordination between the two types of excitatory neuron. A fifth group also targeted L5a, L5b, and L5ET cells with high density, multi-synapse connections, but contributed few synapses compared to the others (Fig. 6k).

These data suggest that inhibitory circuits were organized differently in upper layers compared to layer 5. Measured as a fraction of intracolumnar inhibition, each of the motif groups described above contribute a substantial amount of all measured inhibition onto at least one M-type in layers 2–5 (Fig. 6k). However, in layers 2–4, each excitatory M-type received strong inhibition from 2–3 motif groups with overlapping combinations of targets, some specific within layers and others that cross layer boundaries (Fig. 6l). In contrast, most motif groups targeted only single M-types in layer 5, although in some cases also targeted cells in other layers (Fig. 6m).

Connectivity patterns in layer 6 were a combination of specific and mixed (Extended Data Fig. 12). Although several motif groups had specific connectivity with layer 6 M-types, particularly in upper layer 6, the plurality of synapses onto L6a, L6b, L6c, and L6CT cells all came from Group 14 (Fig. 6k). Nonetheless, several individual cells selectively targeted or avoided L6-CTs (Extended Data Fig. 12), suggesting that similar to layer 5, different projection subclasses in layer 6 have the potential to be independently inhibited.

## Discussion

Here, we generated an EM reconstruction of neuronal anatomy and inhibitory connectivity across a column of visual cortex. This dataset offered new insights into not only the connectivity of individual neurons, but also the broader organization of inhibition across neuronal subclasses. Using synaptic properties in addition to traditional morphological features, we found a collection of excitatory cell types with sublaminar structure and differential inhibitory input, demonstrating that these anatomical distinctions are reflected in the cortical circuit. Inhibitory neurons were organized into motif groups, heterogeneous collections of cells that targeted both the perisomatic and dendritic compartments of particular combinations of M-types. We also identified new aspects to the inhibition of inhibition, including a novel type of disinhibitory specialist that targets basket cells.

### A. Implications of inhibitory organization for cortical function.

While excitatory neurons display morphological and transcriptomic diversity beyond layer boundaries or even long range projection classes^13,74^, it has been unclear how or if these differences have functional consequences in the local cortical circuit. We found that synaptic properties like synapse density and synapse size can distinguish spatially-intermingled excitatory subpopulations that have distinct sources of local inhibition. A key question will be if the landscape of M-types relates to other aspects of their connectivity, such as the target region of their axonal projections or input from long-range sources^75^. For example, the differential inhibition onto L2a and L2b M-types we observed in VISp is reminiscent of differential inhibition observed in layer 2 of prefrontal cortex, where amygdala-projecting cells receive inhibition that selectively avoids neighboring cortical-projecting cells^35,64^. In layer 2/3 of VISp, excitatory neurons project to different higher order visual areas in a heterogeneous and depth-dependent manner^76^, offering the possibility that the inhibitory selectivity we observed could produce a similar pattern of pathway-specific gating or modulation. More generally, the distinct inhibitory environments of upper and lower layer 2/3 have been observed across the brain, from primary sensory cortices^43,77^ to higher-order association areas^78^, suggesting that it may reflect a general functional specialization. For example, the distinct inhibitory environments of layer 2 and layer 3 are potentially well-posed for differential inhibition of top-down versus sensory-driven activity that has been recently described^79,80^.

The particular distribution of inhibitory motif groups also offers insights into the functional relationships of excitatory cell types. In layers 5 and 6, each M-type with a different long-range projection classes (ET, NP, CT, and IT) had a collection of inhibitory cells for which they were the predominant target. This affords the network the potential to individually control each projection class via selective inhibition both at the soma and across dendrites, potentially with different inhibitory types active under different network conditions and behavioral states^17^, or with synapses changing via different plasticity rules^81^. However, inhibitory input was distributed unequally across excitatory targets. For example, ET cells received inputs from a greater number and diversity of inhibitory cell types than layer 5 IT or NP cells. This large population of highly selective inhibitory cell types, consistent with recent observations of transcriptomically-defined SST neurons^36^, could allow for more complex regulation of these key cortical outputs compared to other layer 5 cells. Most M-types received input from multiple motif groups, suggesting there could be network conditions when different combinations of excitatory subpopulations controlled together as part of a shared circuit. The functional consequences will depend on how different groups of inhibitory neurons are recruited. It will be important to understand the extent to which cells with overlapping targets are active simultaneously, collectively setting an inhibitory tone, or instead if they switch activity under different contexts, as in hippocampal basket cells^25^.

Our data also suggest important refinements to our understanding of the inhibition of inhibition. While the EM connectivity agreed well with reciprocal connectivity between VIP and SST neurons observed previously^24,38^, we found that that putative SST cells in upper layer 2/3 did not participate in this microcircuit. These are exactly the cells that preferentially targeted layer 2 M-types, implying that VIP-mediated disinhibition during arousal or locomotion^82,83^ applies to layer 3 and 4, but not layer 2. Additionally, the lack of VIP-mediated disinhibition in layer 2 complements direct inhibition from chandelier cells, a GABAergic neuron that also preferentially targets excitatory cells in upper layer 2/3^35,43^ and that are active during similar behavioral states^17,43,84^. All else being equal, the net result could alter dynamics in the upper layers by shifting inhibition away from layer 3 cells and onto layer 2.

Finally, the observation of a novel class of disinhibitory specialists that target basket cells offers an intriguing pathway for the network to locally enhance excitatory gain. Such a circuit motif could be well suited to many functions, thus discovering the conditions under which these cells are active will be a useful avenue of future research. Further analysis using Patch-seq data will be useful to shed light as to the transcriptomic types that have this connectivity.

### B. Limitations.

A principle concern is the generalizability of data, which comes from a single animal, is located toward the edge of VISp, and has at most a few examples per cell type. Concurrent work in the same dataset has found that morphologically-defined cell types show consistent target preferences^85^, and our data also agrees with recent functional measurements of type-specific connectivity of SST cells^36^. We thus believe that the connectivity results will apply generally, although it will be important to measure the variability across individual cells, distinct animals, and locations in cortex. However, it is an interesting question if the connection specificity we observed is entirely determined by molecularly-defined cell types or if other factors such as developmental timing or activity-dependent plasticity^86^ play a significant role in shaping these patterns.

Additionally, this study only considered cells and connectivity within a narrow range of distances and limited volume. If cells change their connectivity with distance, as has been seen in excitatory neurons^87^, or if some regions of the axon were within the column but others were not, this would bias the observed connectivity distributions. Further, while the columnar approach did a good job of sampling excitatory neurons across layers, we had few examples for any given inhibitory cell type, and some known cell types such as chandelier cells were not observed at all. Extending a similar analysis across a much wider extent will be important for building a complete map of inhibitory cell types and firmly establishing the nature of inhibitory motif groups. Finally, the lack of segmentation in the top 10 *µm* of layer 1 truncates some apical tufts as well as limiting reconstruction quality of layer 1 interneurons. For those excitatory neurons with extensive apical tufts, particularly layer 2 and L5ET cells, the reconstructions here might both miss distinguishing characteristics and sources of inhibitory input to that region.

### C. Comparison to other cell type definitions.

Recent surveys aimed at discovering excitatory cell types have used morphology alone^88^, morphology and electrophysiology^13^, transcriptomics^19,89^, or all three together using Patch-seq^16^. The M-types found here from morphology and synaptic properties generally agree with these approaches, distinguishing cells in upper layer and lower layer 2/3, finding layer 4 diversity, and differentiating between projection subclasses. Transcriptomic studies have found multiple excitatory clusters in upper layer 2/3 in VISp^89,90^, but it is unclear if they correspond to the two morphological clusters observed here. In other cases, the M-types described here can likely be divided further. Transcriptomics finds multiple layer 5 ET subtypes that we do not distinguish in the clustering here, likely due to the relatively few ET cells in the column data. Similarly, it is likely that some of the layer 6 M-types here can be subdivided; for example, L6b contains cells with both upright and inverted apical dendrites. Improved sampling of rare cell types and more complete arbor reconstructions will help refine the landscape of cell types.

### D. Peters’ rule and White’s exceptions.

Since the seminal work of Braitenberg and Schulz^70^ and the experiments of Alan Peters^91^ and Edward White^92^, two ideas have been pitched as opposites: One is that cell type connectivity simply reflects the distribution of pre- and post-synaptic elements of each cell type (“Peters’ rule”) and the other is that cells preferentially target certain cells over others (“White’s exceptions”). These concepts have been of great value in estimating the cortical wiring diagram^68,93^ and suggesting null hypotheses for targeted studies^94^. Both mechanisms are likely to be used by cortical neurons. Our data show that while some inhibitory cell types present remarkably selective connectivity, other cell types appear to broadly target available partners. In addition, some cells have extremely specific connectivity, for example onto layer 2 M-types, but with no evidence of selectivity beyond tight control of their axonal arbors. Instead of considering Peters’ rule as opposite to White’s exceptions, our data suggests that neurons use a combination of spatial overlap, morphology, and synaptic selectivity together, with cell-type specific differences in the degree to which each aspect contributes to observed connectivity.

### E. A foundation for the multimodal study of cell types.

This work provides the foundation to dissect the connectivity of cortical cell types^5^, but to make the most use of this type of data will require following in the footsteps of *Drosophila* and linking EM to genetic tools^95^. In many cases, there are striking morphological similarities between interneurons reconstructed here in EM and specific samples from a Patch-seq dataset of VISp interneurons^30^, and a concurrent study has used Patch-seq data to link EM reconstructions of layer 5 Martinotti cells to transcriptomic types^85^. Currently, however, many cell subclasses based on Patch-seq data include diverse morphologies, each likely to have different connectivity^16,30^. This suggests that the process of linking structural and molecular datasets should aim to become bi-directional, not only decorating EM reconstructions with transcriptomic information, but also using EM to identify cell types with distinct connectivity and analyzing Patch-seq data to identify transcriptomic markers.

The anatomical data presented here are exceptionally rich, and this study offered just one approach to its analysis. To facilitate subsequent analysis of anatomy, connectivity, and ultrastructure, all EM data, segmentations, skeletons and tables of synapses and cell types are available will be available via MICrONs-Explorer^51^. In addition to being a rich resource for detailed neuroanatomy, this highly-curated population within a larger volume will serve an important role for future analyses. The data presented here can serve as training data for anatomical classifiers^96,97^ or improvements in automated proofreading^98^. Moreover, it can serve as a rich seed from which to consider other aspects of the cortical circuit in the same dataset, such as thalamic input, excitatory connectivity, and functional properties.

## Methods

### Animal preparation for EM.

All animal procedures were approved by the Institutional Animal Care and Use Committee at the Allen Institute for Brain Science or Baylor College of Medicine. Neurophysiology data acquisition was conducted at Baylor College of Medicine prior to EM imaging, aftearwards the mice were transferred to the Allen Institute in Seattle and kept in a quarantine facility for 1–3 days, after which they were euthanized and perfused. All results described here are from a single male mouse, age 64 days at onset of experiments, expressing GCaMP6s in excitatory neurons via SLC17a7-Cre and Ai162 heterozygous transgenic lines (recommended and generously shared by Hongkui Zeng at Allen Institute for Brain Science; JAX stock 023527 and 031562, respectively). Two-photon functional imaging took place between P75 and P80 followed by two-photon structural imagiung of cell bodies and blood vessels at P80. The mouse was perfused at P87. Details of animal preparation are described in detail elsewhere^51^ and summarized below.

### *In vivo* two photon imaging.

Neurophysiology data acquisition was conducted for this mouse^51^ but not used in the analysis presented in this manuscript. Briefly, anesthesia was induced with 3% isoflurane and maintained with 1.5 – 2% isoflurane during the surgical procedure. Mice were injected with 5–10 mg/kg ketoprofen subcutaneously at the start of the surgery. The anesthetized mice were placed in a stereotaxic head holder (Kopf Instruments) and its body temperature was maintained at 37° throughout the surgery using a homeothermic blanket system (Harvard Instruments). After shaving the scalp, bupivicane (0.05 mL, 0.5%, Marcaine) was applied subcutaneously. An area of skin was removed above the skull and the underlying fascia was scraped and removed. The wound margins were sealed with a thin layer of surgical glue (VetBond, 3M), and a 13 mm stainless-steel washer clamped in the headbar was attached with dental cement (Dentsply Grip Cement). A 4 mm diameter circular craniotomy was made centered on the border between primary visual cortex and lateromedial visual cortex. The cortical window was then sealed with a 4 mm coverslip (Warner Instruments), using cyanoacrylate glue (VetBond). The mice were allowed to recover for 1 day prior to imaging. After imaging, the washer was released from the headbar and the mouse was returned to the home cage. Prior to surgery and throughout the imaging period, the mouse was singly-housed and maintained on a reverse 12-hour light cycle (off at 11 am, on at 11 pm). During imaging, mice were head-mounted above a cylindrical treadmill and calcium imaging was performed using Chameleon Ti-Sapphire laser (Coherent) tuned to 920 nm and a large field of view mesoscope equipped with a custom objective (excitation NA 0.6, collection NA 1.0, 21 mm focal length). For the two photon structural imaging, approximately 55 minutes prior to collecting the stack, the animal was injected subcutaneously with 60 µL of 8.3 mM Dextran Texas Red fluorescent dye (Invitrogen, D3329).

### Tissue preparation.

After optical imaging at Baylor College of Medicine, candidate mice were shipped via overnight air freight to the Allen Institute. All mice were housed in individually ventilated cages, 20–26 C, 30–70% Relative Humidity, with a 12-hour light/dark cycle. Mice were transcardially perfused with a fixative mixture of 2.5% paraformaldehyde, 1.25% glutaraldehyde, and 2 mM calcium chloride, in 0.08 M sodium cacodylate buffer, pH 7.4. After dissection, the neurophysiological recording site was identified by mapping the brain surface vasculature. A thick (1200 µm) slice was cut with a vibratome and post-fixed in perfusate solution for 12–48 h. Slices were extensively washed and prepared for reduced osmium treatment (rOTO) based on the protocol of Hua and colleagues^99^. All steps were performed at room temperature, unless indicated otherwise. 2% osmium tetroxide (78 mM) with 8% v/v formamide (1.77 M) in 0.1 M sodium cacodylate buffer, pH 7.4, for 180 minutes, was the first osmication step. Potassium ferricyanide 2.5% (76 mM) in 0.1 M sodium cacodylate, 90 minutes, was then used to reduce the osmium. The second osmium step was at a concentration of 2% in 0.1 M sodium cacodylate, for 150 minutes. Samples were washed with water, then immersed in thiocarbohydrazide (TCH) for further intensification of the staining (1% TCH (94 mM) in water, 40 °C, for 50 minutes). After washing with water, samples were immersed in a third osmium immersion of 2% in water for 90 minutes. After extensive washing in water, lead aspartate (Walton’s (20 mM lead nitrate in 30 mM aspartate buffer, pH 5.5), 50°, 120 minutes) was used to enhance contrast. After two rounds of water wash steps, samples proceeded through a graded ethanol dehydration series (50%, 70%, 90% w/v in water, 30 minutes each at 4 °C, then 3 × 100%, 30 minutes each at room temperature). Two rounds of 100% acetonitrile (30 minutes each) served as a transitional solvent step before proceeding to epoxy resin (EMS Hard Plus). A progressive resin infiltration series (1:2 resin:acetonitrile (e.g. 33% v/v), 1:1 resin:acetonitrile (50% v/v), 2:1 resin acetonitrile (66% v/v), then 2 × 100% resin, each step for 24 hours or more, on a gyrotary shaker) was done before final embedding in 100% resin in small coffin molds. Epoxy was cured at 60° for 96 hours before unmolding and mounting on microtome sample stubs. The sections were then collected at a nominal thickness of 40 nm using a modified ATUMtome (RMC/Boeckeler^48^) onto 6 reels of grid tape^48,100^.

### Transmission electron microscopy imaging.

The parallel imaging pipeline used in this study^48^ (Yin et al. 2020) used a fleet of transmission electron microscopes that had been converted to continuous automated operation. It is built upon a standard JEOL 1200EXII 120kV TEM that had been modified with customized hardware and software. The key hardware modifications included an extended column and a custom electron-sensitive scintillator. A single large-format CMOS camera outfitted with a low distortion lens was used to grab image frames at an average speed of 100 ms. The autoTEM was also equipped with a nano-positioning sample stage that offered fast, high-fidelity montaging of large tissue sections and an advanced reel-to-reel tape translation system that accurately locates each section using index barcodes for random access on the GridTape. In order for the autoTEM system to control the state of the microscope without human intervention and ensure consistent data quality, we also developed customized software infrastructure piTEAM that provides a convenient GUI-based operating system for image acquisition, TEM image database, real-time image processing and quality control, and closed-loop feedback for error detection and system protection etc. During imaging, the reel-to-reel GridStage moved the tape and located the targeting aperture through its barcode. The 2D montage was then acquired through raster scanning the ROI area of tissue. Images along with metadata files were transferred to the data storage server. We performed image QC on all data and reimaged sections that failed the screening.

### Image processing: Volume assembly.

The volume assembly pipeline is described in detail elsewhere^49,50^. Briefly, the images collected by the autoTEMs are first corrected for lens distortion effects. A non-linear transformation of higher order is computed for each section using a set of 10 × 10 highly overlapping images collected at regular intervals during imaging. The lens distortion correction transformations should represent the dynamic distortion effects from the TEM lens system and hence require an acquisition of highly overlapping calibration montages at regular intervals. Overlapping image pairs are identified within each section and point correspondences are extracted for every pair using a feature based approach. In our stitching and alignment pipeline, we use SIFT feature descriptors to identify and extract these point correspondences. Per image transformation parameters are estimated by a regularized solver algorithm. The algorithm minimizes the sum of squared distances between the point correspondences between these tile images. Deforming the tiles within a section based on these transformations results in a seamless registration of the section. A downsampled version of these stitched sections are produced for estimating a per-section transformation that roughly aligns these sections in 3D. A process similar to 2D stitching is followed here, where the point correspondences are computed between pairs of sections that are within a desired distance in z direction. The per-section transformation is then applied to all the tile images within the section to obtain a rough aligned volume. Mipmaps are utilized throughout the stitching process for faster processing without compromise in stitching quality. The rough aligned volume is rendered to disk for further fine alignment. The software tools used to stitch and align the dataset is available in our github repository https://github.com/AllenInstitute/render-modules. The volume assembly process is entirely based on image metadata and transformations manipulations and is supported by the Render service (https://github.com/saalfeldlab/render). To fine align the volume it was required to make the image processing pipeline robust to image and sample artifacts. Cracks larger than 30 um in 34 sections were corrected by manually defining transforms. The smaller and more numerous cracks and folds in the dataset were automatically identified using convolutional networks trained on manually labeled samples using 64 × 64 × 40 nm^3^ resolution image. The same was done to identify voxels which were considered tissue. The rough alignment was iteratively refined in a coarse-to-fine hierarchy^101^, using an approach based on a convolutional network to estimate displacements between a pair of images^102^. Displacement fields were estimated between pairs of neighboring sections, then combined to produce a final displacement field for each image to further transform the image stack. Alignment was first refined using 1024 × 1024 × 40 nm^3^ images, then 64 × 64 × 40 nm^3^ images. The composite image of the partial sections was created using the tissue mask previously computed. Pixels in a partial section which were not included in the tissue mask were set to the value of the nearest pixel in a higher-indexed section that was considered tissue. This composite image was used for downstream processing, but not included with the released images.

### Image processing: Segmentation.

The image segmentation pipeline is described in Macrina et al^49^. Remaining misalignments were detected by cross-correlating patches of image in the same location between two sections, after transforming into the frequency domain and applying a high-pass filter. Combining with the tissue map previously computed, a mask was generated that sets the output of later processing steps to zero in locations with poor alignment. This is called the segmentation output mask. Using previously described methods^103^, a convolutional network was trained to estimate inter-voxel affinities that represent the potential for neuronal boundaries between adjacent image voxels. A convolutional network was also trained to perform a semantic segmentation of the image for neurite classifications, including (1) soma+nucleus, (2) axon, (3) dendrite, (4) glia, and (5) blood vessel. Following the methods described in Wu et al^104^, both networks were applied to the entire dataset at 8 × 8 × 40 nm^3^ in overlapping chunks to produce a consistent prediction of the affinity and neurite classification maps. The segmentation output mask was applied to the predictions. The affinity map was processed with a distributed watershed and clustering algorithm to produce an over-segmented image, where the watershed domains are agglomerated using single-linkage clustering with size thresholds^105,106^. The over-segmentation was then processed by a distributed mean affinity clustering algorithm^105,106^ to create the final segmentation. We augmented the standard mean affinity criterion with constraints based on segment sizes and neurite classification maps during the agglomeration process to prevent neuron-glia mergers as well as axon-dendrite and axon-soma mergers.

For synapse detection and assignment, a convolutional network was trained to predict whether a given voxel participated in a synaptic cleft. Inference on the entire dataset was processed using the methods described in Wu et al^104^ using 8 × 8 × 40 nm^3^ images. These synaptic cleft predictions were segmented using connected components, and components smaller than 40 voxels were removed. A separate network was trained to perform synaptic partner assignment by predicting the voxels of the synaptic partners given the synaptic cleft as an attentional signal^107^. This assignment network was run for each detected cleft, and coordinates of both the presynaptic and postsynaptic partner predictions were logged along with each cleft prediction. For nucleus detection^51,96^ a convolutional network was trained to predict whether a voxel participated in a cell nucleus. Following the methods described in Wu et al^104^, a nucleus prediction map was produced on the entire dataset at 64 × 64 × 40 nm^3^. The nucleus prediction was thresholded at 0.5, and segmented using connected components.

### Column description and cell classes.

The column borders were found by manually identifying a region in primary visual cortex that was as far as possible from both dataset boundaries and the boundaries with higher order visual areas. A 100 *µm ×* 100 *µm* box was placed based on layer 2/3 and was extended along the negative y axis of the dataset. While analyzing data, we observed that deep layer neurons had apical dendrites that left the column, and we adapted the definition of the column to accommodate these neuronal streamlines. Using a collection of layer 5 ET cells, we placed points along the apical dendrite to the cell body and then along the primary descending axon towards white matter. We computed the slant angle as two piecewise linear segments, one along the negative y axis to lower layer 5 where little slant was observed, and one along the direction defined by the vector averaged direction of the labeled axons.

Using these boundaries and previously computed nucleus centroids^51^, we identified all cells inside the columnar volume. Coarse cell classes (excitatory, inhibitory, and non-neuronal) were assigned based on brief manual examination and rechecked by subsequent proofreading and automated cell typing^96^. To facilitate concurrent analysis and proofreading, we split all false merges connecting any column neurons to other cells (as defined by detected nuclei) before continuing with other work.

### Proofreading.

Proofreading was performed primarily by five expert neuroanatomists using the PyChunkedGraph^53,54^ infrastructure and a modified version of Neuroglancer^108^. Proofreading was aided by on-demand highlighting of branch points and tips on user-defined regions of a neuron based on rapid skeletonization (https://github.com/AllenInstitute/Guidebook). This approach quickly directed proofreader attention to potential false merges and locations for extension, as well as allowed a clear record of regions of an arbor that had been evaluated.

For dendrites, we checked all branch points for correctness and all tips to see if they could be extended. False merges of simple axon fragments onto dendrites were often not corrected in the raw data, since they could be computationally filtered for analysis after skeletonization (see next section). Detached spine heads were not comprehensively proofread. Using this method, dendrites could be proofread in approximately ten minutes per cell.

For inhibitory axons, we began by “cleaning” axons of false merges by looking at all branch points. We then performed extension of axonal tips until either their biological completion or data ambiguities, particularly emphasizing all thick branches or tips that were well-suited to project to new laminar regions. For axons with many thousand synaptic outputs, we followed some but not all tips to completion once major branches were cleaned and established. For smaller neurons, particularly those with bipolar or multipolar morphology, most tips were extended to the point of completion or ambiguity. Axon proofreading time differed significantly by cell type not only because of differential total axon length, but axon thickness differences that resulted in differential quality of autosegmentations, with thicker axons being of higher initial quality. Typically, inhibitory axon cleaning and extension took 3–10 hours per neuron.

### Skeletonization.

To rapidly skeletonize dynamic data, we took advantage of the PyChunkedGraph data structure that collects all supervoxels belonging to the same neuronal segmentation into 2 *µm* × 2 *µm* × 20 *µm* “chunks” with a unique id and precisely defined connectivity to neighboring chunks of the same object. Each chunk is called a “level 2 chunk” and the complete set of chunks for a neuron and their adjacency we call the “level 2 graph,” based on its location in the hierarchy of the PyChunkedGraph data structure^54^. We precompute and cache a representative central point in space, the volume, and the surface area for each level 2 chunk and update this data when new chunks are created due to proofreading edits. Using the level 2 graph and assigning edge lengths corresponding to the distance between the representative points for each vertex (i.e. each level 2 chunk), we run the TEASAR^109^ algorithm (10 *µm* invalidation radius) to extract a loop-free skeleton. Each of the level 2 vertices removed by the TEASAR algorithm is associated with its closest remaining skeleton, making it possible to map surface area and volume data to the skeleton. Typical edges between skeleton vertices are about 1.7 *µm* , and new skeletons can be computed *de novo* in approximately 10 seconds, making them useful for analysis over length scales of tens of *µm* or larger.

To represent the cell body, an additional vertex was placed at the location of the nucleus centroid and all vertices within an initial 7.5 *µm* region were collapsed into this vertex with associated data mapping. Skeletons were rooted at the cell body, with “downstream” meaning away from soma and “upstream” meaning towards soma. For some cells (often layer 5 ETs), the 7.5 *µm* distance did not capture the extent of the soma, and a custom radius was defined in the skeletonization pipeline (Cell IDs with 10 *µm* radius: 303216, 267033, 311789, 347181; Cell IDs with 12 *µm* radius: 30295). Each synapse was assigned to skeleton vertices based on the level 2 chunk of its associated supervoxel. For each unbranched segment of the skeleton (i.e. between two branch points or between a branch point and end point), we computed an approximate radius *r* based on a cylinder with the same path length *L* and total volume *V* associated with that segment $\left(r=\sqrt{V/\pi L}\right)$.

### Axon/dendrite classification.

To detect axons, we took advantage of the skeleton morphology, the location of presynaptic and postsynaptic synapses, and the clear segregation between inputs and outputs of cortical neurons. For inhibitory cells, we used synapse flow centrality^110^ to identify the start of the axon as the location of maximum paths along the skeleton between sites of synaptic input and output. Two inhibitory neurons had two distinct, biologically correct axons after proofreading, for these cells we ran this method twice, masking off the axon found after the first run, in order to identify both. For excitatory neurons that did not have extended axons, there were often insufficient synaptic outputs on their axon for this approach to be reliable. Excitatory neurons with a segregation index^110^ of 0.7 (on a scale with 0 indicating random distribution of input and output synapses and 1 indicating perfect input/output segregation) or above were considered well-separated and the synapse flow centrality solution was used. For cells with a segregation index less than 0.7, we instead looked for branches near the soma with few synaptic inputs. Specifically, we took identified all skeleton vertices within 30 *µm* from the cell body and looked at the distinct branches downstream of this region. For each branch, we computed the total path length and the total number of synaptic inputs in order to get a linear input density. Branches with a path length both more than 20 *µm* and with an input density less than 0.1 synaptic inputs per *µm* were labeled as being axonal and filtered out of subsequent analysis.

We further filtered out any remaining axon fragments merged onto pyramidal cell dendrites using a similar approach. We identified all unbranched segments (regions between two branch points or between a branch point and end point) on the non-axonal region of the skeleton and computed their input synapse density. Starting from terminal segments (i.e. those with no downstream segments), we labeled a segment as a “false merge” if it had an input density less than 0.1 synaptic inputs per *µm*. This process iterated across terminal segments until all remaining had an input density of at least 0.1 inputs per *µm* . Falsely merged segments were masked out of the skeleton for all analysis.

### Excitatory dendrite compartments.

We assigned all synaptic inputs onto excitatory neurons to one of four compartments: soma, proximal dendrite, distal basal dendrite, and distal apical dendrite. The most complex part was distinguishing the basal dendrite from the apical dendrite. While easy in most cases for neurons in layer 3–5 due to the consistent nature of apical dendrites being single branches reaching toward layer 1, this is not true everywhere. In upper layer 2/3 cells often have multiple branches in layer 1 equally consistent with apical dendrites and in layer 6 there are often cells with apical dendrites that stop in layer 4, that point toward white matter, or even that lack a clear apical branch entirely. To objectively and scalably define apical dendrites, we built a classifier that could detect between 0–3 distinct apical branches per cell. Following the intuition from neuroanatomical experts, we used features based on the branch orientation, location in space, relative location compared to the cell body, and branch-level complexity. Specifically, we trained a random forest classifier to predict whether a skeleton vertex belonged to an apical dendrite based on several features: Depth of vertex, depth of soma, difference in depth between soma and vertex, vertex distance to soma along the skeleton, vertex distance to farthest tip, normalized vertex distance to tip (between 0 and 1), tortuosity of path to root, number of branch points along the path to root, radial distance from soma, absolute distance from soma, and angle relative to vertical between the vector from soma to vertex. We aggregated predictions within each branch by summing the log odds ratio from the model prediction, with the net log-odds ratio saturating at ±200. Finally, for each branch *i* with aggregated odds ratio *R*_*i*_, we compare branches to one another via a soft-max operation: $S_{i}=\exp\left(R_{i}/50\right)/\sum_{j}\exp\left(R_{i}/50\right)$. Branches with a maximum tip length of less than 50 *µm* were considered too short to be a potential apical dendrite and excluded from consideration and not included in the denominator. Branches with both *R*_*i*_ *>* 0 (evidence is positive towards being apical) and *S*_*i*_ *>* 0.25 (allowing no more than 4 apical branches with equal weight to be possible, and more likely 2–3 at most). Training data was selected from an initial 50 random cells, followed by an additional 33 cells chosen representing cases where the classifier did not perform correctly. Performance on both random and difficult cells had an F1 score of 0.9297 (86 true positives, 599 true negatives, 2 false positives, and 11 false negatives) based on leave-one-out cross validation, with at least one apical dendrite correctly classified for all cells.

Compartment labels were propagated to synapses based on the associated skeleton vertices. Soma synapses were all those associated with level 2 chunks within the soma collapse region (see Skeletonization section). Proximal dendrites were those outside of the soma, but within 50 *µm* after the start of the branch. Distal basal synapses were all those associated with vertices more distant than the proximal threshold, but not on an apical branch. Apical synapses were all those associated with vertices more distant than the proximal threshold and on an apical branch.

### Inhibitory feature extraction and clustering.

Many classical methods of distinguishing interneuron classes are based on how cells distribute their synapses across target compartments. Following proofreading, expert neuroanatomists attempted to classify all inhibitory neurons broadly into “basket cells,” “somatostatin-like cells”, “bipolar/multipolar cells”, and “neurogliaform/layer 1” cells based on connectivity properties and morphology. While 150 cells were labeled on this basis, an additional 13 neurons were considered uncertain (primarily in layer 6) and in some cases manual labels were low confidence. To classify inhibitory neurons in a data driven manner, we thus measured four properties of how cells distribute their synaptic outputs:

1. The fraction of synapses onto inhibitory neurons.
2. The fraction of synapses onto excitatory neurons that are onto soma.
3. The fraction of synapses onto excitatory neurons that are onto proximal dendrites.
4. The fraction of synapses onto excitatory neurons that are onto distal apical dendrites.

Because the fraction of synapses targeting all compartments sums to one, the last remaining property, synapses onto distal basal dendrites, was not independent and thus was measured but not included as a feature. Inspection of the data suggested two additional properties that characterized synaptic output across inhibitory neurons:

- The fraction of synapses that are part of multisynaptic connections, those with at least two synapses between the same presynaptic neuron and target neuron.
- The fraction of multisynaptic connection synapses that were also within 15 *µm* of another synapse with the same target, as measured between skeleton nodes.

Using these six features, we trained a linear discriminant classifier on cells with manual annotations and applied it to all inhibitory cells. Differences from manual annotations were treated not as inaccurate classifications, but rather a different view of the data.

### Excitatory feature extraction and clustering.

To characterize excitatory neuron morphology, we computed features based only on excitatory neuron dendrites and soma. The features were:

1. Median distance from branch tips to soma per cell.
2. Median tortuosity of the path from branch tips to soma per cell. Tortuosity is measured as the ratio of path length to the Euclidean distance from tip to soma centroid.
3. Number of synaptic inputs on the dendrite.
4. Number of synaptic inputs on the soma.
5. Net path length across all dendritic branches.
6. Radial extent of dendritic arbor. We define “radial distance” to be the distance within the same plane as the pial surface. For every neuron, we computed a pia-to-white-matter line, including slanted region in deep layers, passing through its cell body. For each skeleton vertex, we computed the radial distance to the pia-to-white-matter line at the same depth. To avoid any outliers, the radial extent of the neuron was defined to be the 97th percentile distance across all vertices.
7. Median distance to soma across all synaptic inputs.
8. Median synapse size of synaptic inputs onto the soma.
9. Median synapse size of synaptic inputs onto the dendrites.
10. Dynamic range of synapse size of dendrite synaptic inputs. This was measured as the difference between 95th and 5th percentile synapse sizes.
11. Shallowest extent of synapses, based on the 5th percentile of synapse depths.
12. Deepest extent of synapses, based on the 95th percentile of synapse depths.
13. Vertical extent of synapses, based on the difference between 95th and 5th percentile of synapse depths.
14. Median linear density of synapses. This was measured by computing the net path length and number of synapses along 50 depth bins from layer 1 to white matter and computing the median. A linear density was found by dividing synapse count by path length per bin, and the median was found across all bins with nonzero path length.
15. Median radius across dendritic skeleton vertices. To avoid the region immediately around the soma from having a potential outlier effect, we only considered skeleton vertices at least 30 *µm* from the soma.

Three additional sets of features used component decompositions. To more fully characterize the absolute depth distribution of synaptic inputs, for each excitatory neuron, we computed the number of synapses in each of 50 depth bins from the top of layer 1 to surface of white matter (bin width ≈ 20 *µm*). We z-scored synapse counts for each cell and computed the top six components using SparsePCA. The loadings for each of these components based on the net synapse distribution were used as features.

To characterize the distribution of synaptic inputs relative to the cell body instead of cortical space, we computed the number of synapses in 13 soma-adjusted depth bins starting 100 *µm* above and below the soma. As before, synapse counts were z-scored and we computed the top five components using SparsePCA. The loadings for each of these components were used as additional features.

To characterize the relationship with branching to distance, we measured the number of distinct branches as a function of distance from the soma at ten distances, every 30 *µm* starting at 30 *µm* from the soma and continuing to 300 *µm* . For robustness relative to precise branch point locations, the number of branches were computed by finding the number of distinct connected components of the skeleton found in the subgraph formed by the collection of vertices between each distance value and 10 *µm* toward the soma. We computed the top three singular value components of the matrix based on branch count vs distance for all excitatory neurons, and the loadings were used as features.

All features were computed after a rigid rotation of 5 degrees to flatten the pial surface and translation to zero the pial surface on the y axis. Features based on apical classification were not explicitly used to avoid ambiguities based on both biology and classification.

Using this collection of features, we clustered excitatory neurons by running phenograph^66^ 100 times with 97% of cells included each time. Phenograph finds a nearest neighborhood graph based on proximity in the feature space and clusters by running the Leiden algorithm for community detection on the graph. Here, we used a graph based on 10 nearest neighbors and clustered with a resolution parameter of 1.3. These values were chosen to consistently separate layer 5 ET, IT, and NP cells from one another, a well established biological distinction. A co-clustering matrix was assembled with each element corresponding to the number of times two cells were placed in the same cluster. To compute the final consensus clusters, we performed agglomerative clustering with complete linkage based on the co-clustering matrix, with the target number of clusters set by a minimum Davies-Bouldin score and a maximum Silhouette score. Clusters were then named based on the most frequent manually defined cell type within the cluster and reordered based on median soma depth.

### Inhibitory connectivity and specificity.

To measure inhibitory connectivity, we first restricted synaptic outputs to the axon of each inhibitory neuron. We have not observed any correctly classified synaptic outputs on dendritic arbors in this dataset. All presynaptic sites have been misclassified synaptic clefts, misidentified partners, or correct detection of non-synaptic junctions. One cell with fewer than 30 synaptic outputs was omitted due to size. All remaining synaptic outputs across all interneurons were then filtered to include only those that target cells sampled here. Each output synapse was also labeled with the target skeleton vertex, dendritic compartment, and M-type of the target neuron based on the compartment definitions above.

To measure inhibitory selectivity, we compared the M-type distribution of its synaptic outputs to the M-type distribution of synaptic inputs according to a null model accounting for cell abundance, synapse abundance, and depth. In general, we treated selectivity for each inhibitory neuron and each target M-type as a 2×2 contingency table problem, with one axis reflecting all input synapses for that M-type versus all input synapses of all other types and the other axis reflecting that inhibitory neuron’s outputs versus all synaptic inputs for the column. To account for depth, we made distinct tables for each of 50 depth bins spanning pia to white matter, covering ≈ 20*µm* in depth each. To account for compartment preferences, we also made distinct tables for each potential target compartment: soma, proximal dendrite, distal basal dendrite, apical dendrite, and inhibitory cells. Finally, we treat the tables generated for each depth bin and compartment as a collection of stratified tables, and use the Cochran-Mantel-Haenszel test to compute a common multiplicative factor by which that interneuron’s outputs onto each M-type are more or less frequent than the corresponding synaptic input abundances and to test the statistical significance if this factor differs from 1 with a p-value of 0.05. The confidence interval for the factor was estimated using a normality assumption in statsmodels^111^. To avoid outliers due to small numbers, we excluded compartment and depth bins that had fewer than 10 output synapses from the inhibitory neuron (onto any M-type) or if the compartment and depth bin had fewer than 100 input synapses across all column cells. In the supplemental cell cards, we also measure a similar factor for each target compartment, using the same procedure but with depth bin and cell type tables forming the collection of stratified tables.

### Software and data availability.

Data for this paper was analyzed at materialization version 507, a snapshot taken Sept. 26, 2022 at 8:10 am UTC. Synapse tables for column cells, and tables for manual and automatic cell types and connectivity groups will be made available with this paper. Analysis code will be made publicly available on an Allen Institute Github repository (forthcoming). EM imagery and segmentations, as well as skeletons and cell type tables, are publicly available via https://www.microns-explorer.org/cortical-mm3. All analysis was performed in Python 3.9 using custom code, making extensive use of CAVEclient (https://github.com/seung-lab/CAVEclient) and CloudVolume^112^ to interact with data infrastructure, MeshParty^113^ to analyze skeletons, and libraries Matplotlib^114^, Numpy^115^, Pandas^116^, Scikit-learn^117^, Scipy^118^, stats-models^111^ and VTK^119^ for general computation, machine learning and data visualization.

## Supplementary Material

Supplement 1

Supplement 2
