# Supplementary material for: Cell-type-specific inhibitory circuitry from a connectomic census of mouse visual cortex
Source: bioRxiv. 2023 Feb 14:2023.01.23.525290. Originally published 2023 Jan 24. Preprint. [Version 2] doi: 10.1101/2023.01.23.525290 (PMC9900837; doi:10.1101/2023.01.23.525290)
Supplement: Supplementary file 1 [file NIHPP2023.01.23.525290v2-supplement-2.pdf]

bioRxiv preprint doi: https://doi.org/10.1101/2023.01.23.525290; this version posted February 14, 2023. The copyright holder for this preprint (which was not certified by peer review) is the author/funder, who has granted bioRxiv a license to display the preprint in perpetuity. It is made available under aCC-BY 4.0 International license.

Supplemental Information

Extended Data Figure 1

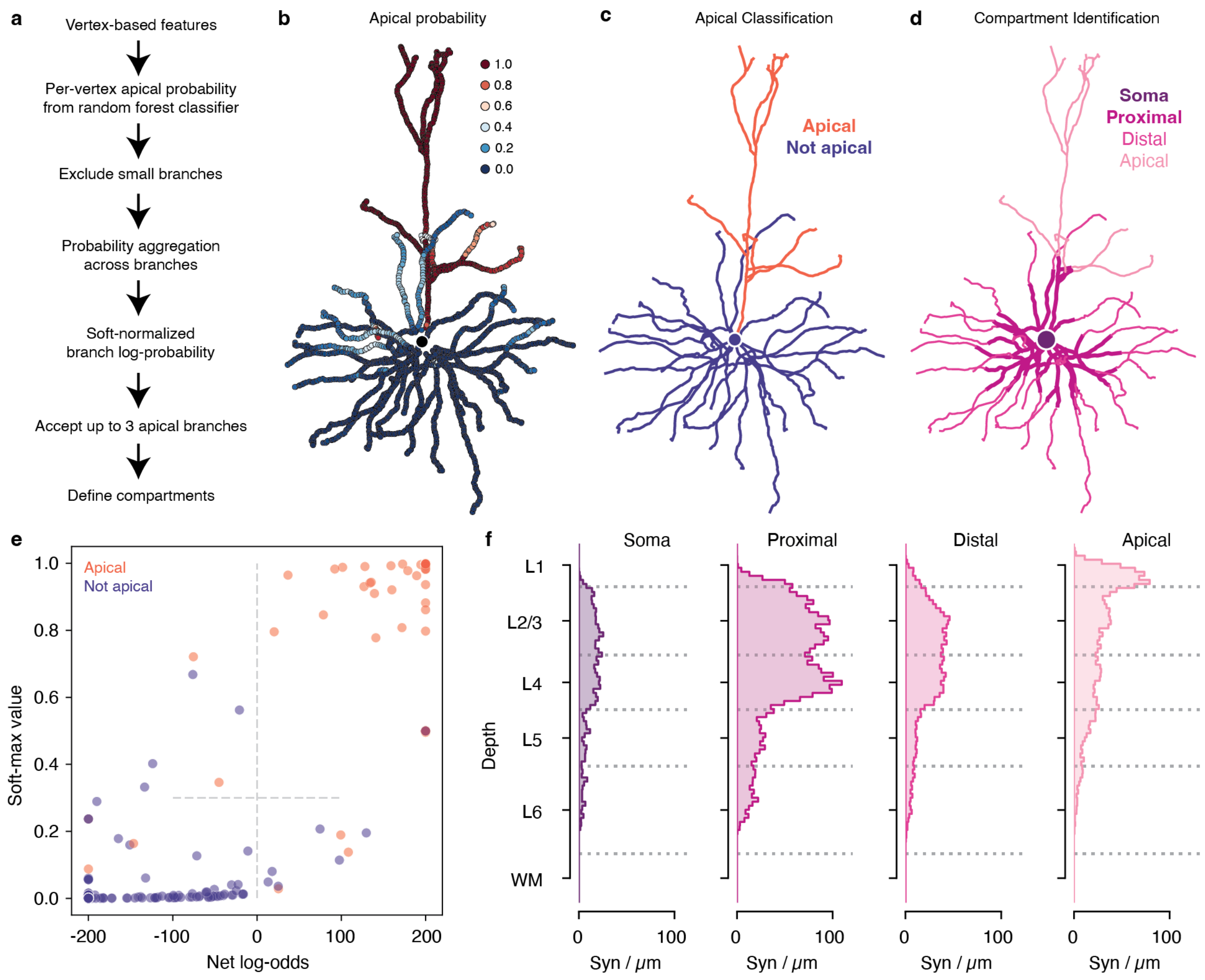

Extended Data Figure 1. Compartment classification pipeline. **a)** Description of the compartment classification pipeline. **b–d)** Pipeline applied to an example layer 3 pyramidal cell. **b)** Apical probability per vertex. **c)** Branch-level apical classification. **d)** Final organization into four dendritic compartments based on apical classification and distance rules. **e)** Quantification of quality of apical branch classification based on leave-one-out classification with a training set based on 50 randomly selected cells and 23 cells chosen to improve difficult classifications. Each dot is a branch of a test pyramidal cell, colored red if apical and blue if not apical. X-axis is the net log-odds of the branch being apical (capped at ± 200) and the y-axis is the relative apical quality based on a soft-max operation (see Methods for details). Branches in the upper right quadrant were classified as apical. The method was able to correctly classify at least one apical branch for all cells, and "false positives" were often associated with borderline cases. **f)** Distribution of synaptic inputs onto excitatory neurons with depth by dendritic compartment. Values are based on counting synapses in bins at a given depth, but at any location laterally.

bioRxiv preprint doi: https://doi.org/10.1101/2023.01.23.525290; this version posted February 14, 2023. The copyright holder for this preprint (which was not certified by peer review) is the author/funder, who has granted bioRxiv a license to display the preprint in perpetuity. It is made available under aCC-BY 4.0 International license.

Extended Data Figure 2

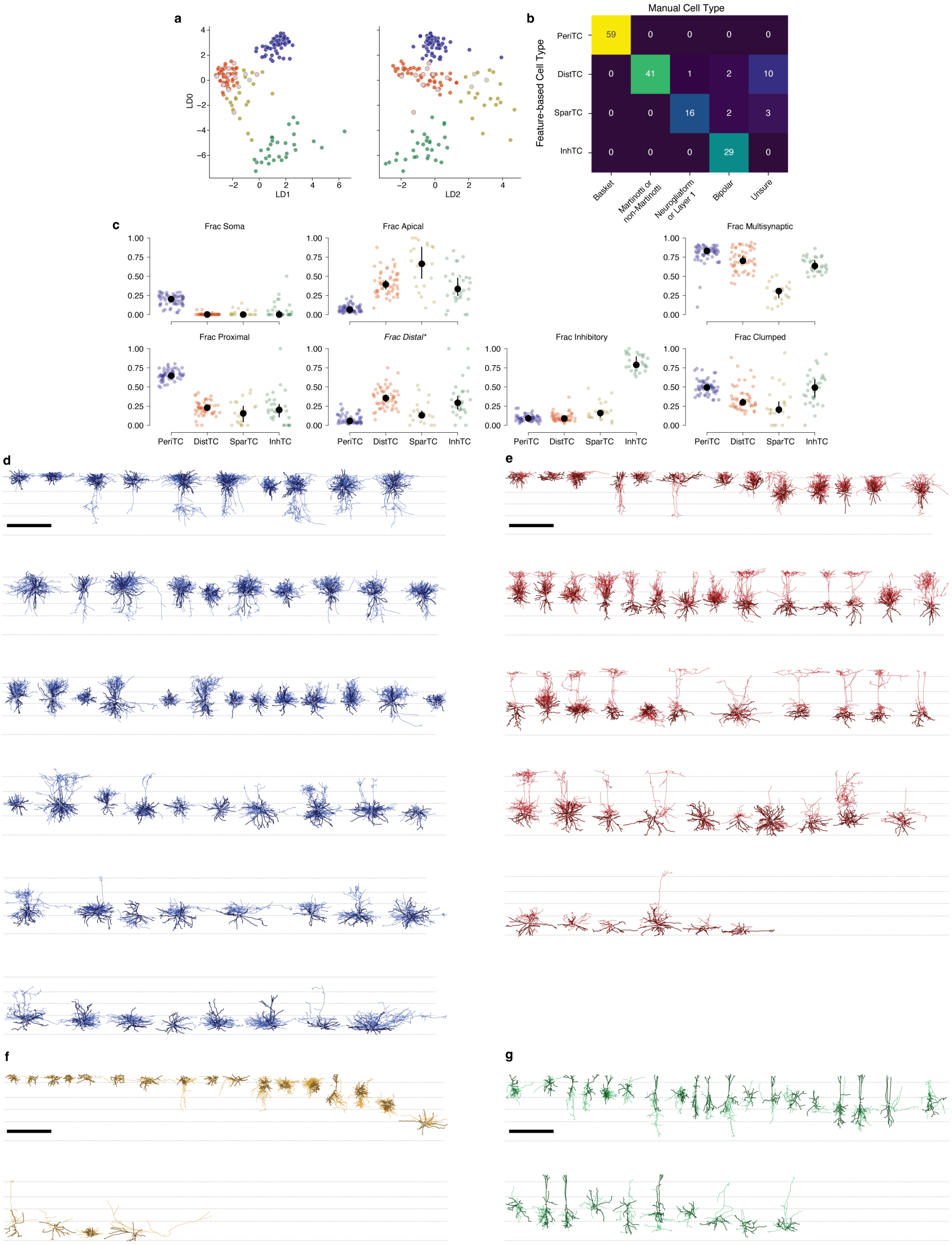

Extended Data Figure 2. Inhibitory neuron properties. **a)** Projections of all analyzed interneurons (n=163) projected on a 3-d space based on linear discriminant analysis (LDA) using connectivity features (shown in **c**). Fully colored dots indicate manually classified cells used as training data for LDA, while dots with grey centers were labeled based on this classification. **b)** Matrix showing relationship between anatomical subclasses and manual classifications. **c)** Individual connectivity features, organized by subclass. Colored dots are individual cells, black dots indicate median with error bars showing a bootstrapped 95% confidence interval. **d–g)** Morphology of all PeriTCs (**d**), DistTCs (**e**), SparTCs (**f**), and InhTCs (**g**). Scale bars are 500 μm . Dark and thick lines are dendrite, thinner and lighter are axon. Cells are ordered by soma depth.

bioRxiv preprint doi: https://doi.org/10.1101/2023.01.23.525290; this version posted February 14, 2023. The copyright holder for this preprint (which was not certified by peer review) is the author/funder, who has granted bioRxiv a license to display the preprint in perpetuity. It is made available under aCC-BY 4.0 International license.

Extended Data Figure 3

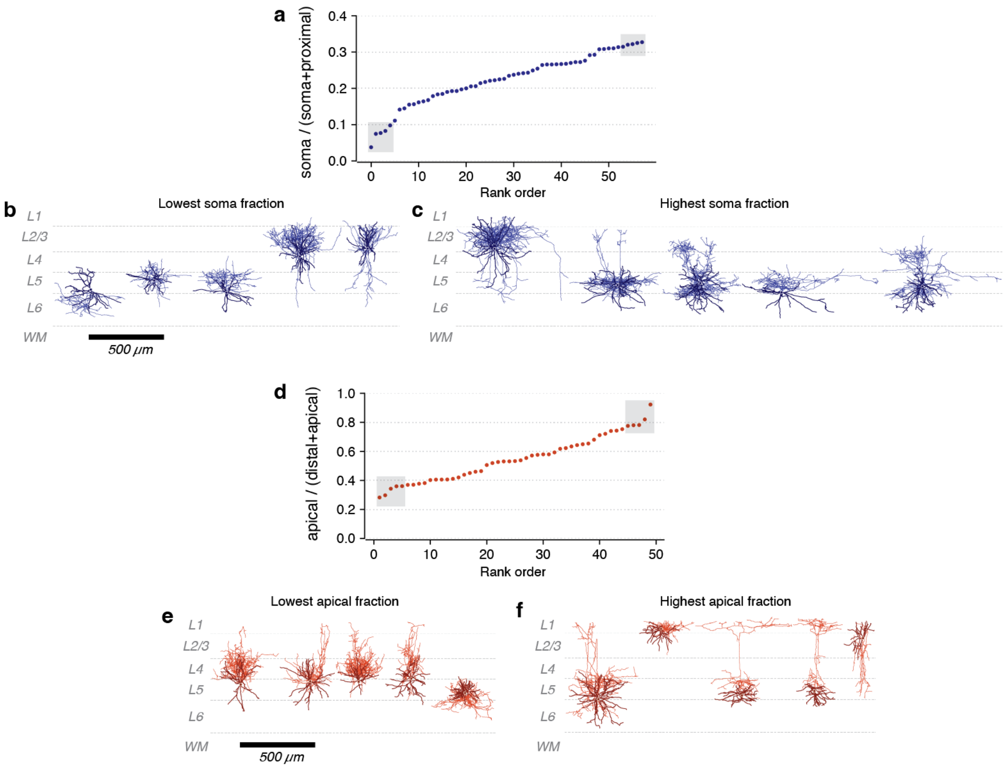

Extended Data Figure 3. Diversity of compartment preference within perisomatic targeting cells and distal dendrite targeting cells. **a)** Among PeriTCs, the rank-ordered fraction of output synapses targeting somata among only those those synapses targeting somata or proximal dendrites. Boxes indicate the top and bottom five cells, shown below. **b)** The five PeriTC cells with the lowest soma fraction, ordered as in **a**. **c)** The five PeriTC cells with the highest soma fraction, ordered as in **a**. **d)** Among DistTCs, the rank-ordered fraction of output synapses targeting apical synapses among only those those synapses targeting apical or distal dendrites. **e)** The five DistTC cells with the lowest apical fraction, ordered as in **a**. **f)** The five DistTC cells with the highest apical fraction, ordered as in **a**.

bioRxiv preprint doi: https://doi.org/10.1101/2023.01.23.525290; this version posted February 14, 2023. The copyright holder for this preprint (which was not certified by peer review) is the author/funder, who has granted bioRxiv a license to display the preprint in perpetuity. It is made available under aCC-BY 4.0 International license.

Extended Data Figure 4

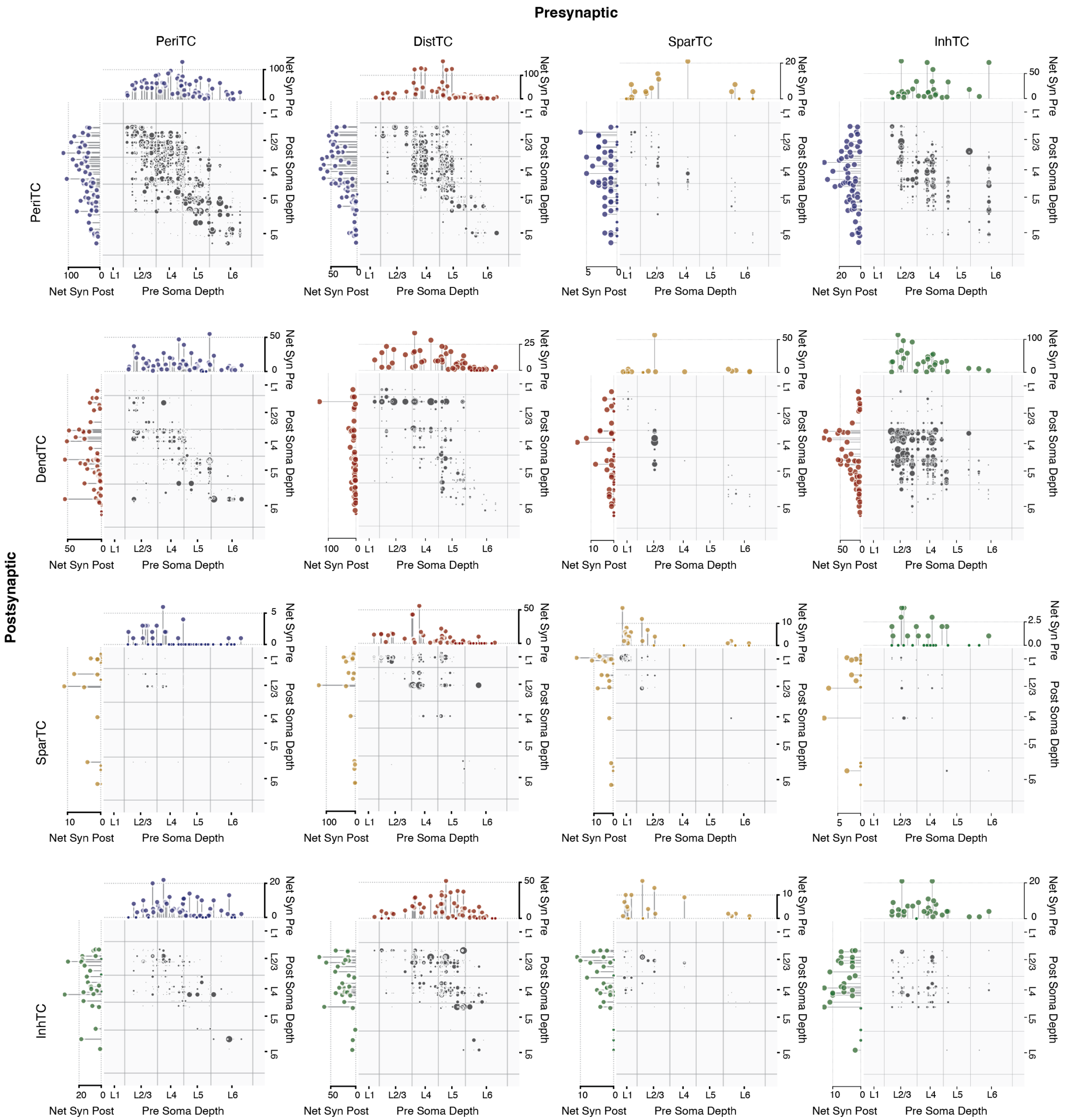

Extended Data Figure 4. Inhibition of inhibition. Connectivity scatterplot between inhibitory neurons, organized by inhibitory subclasses. For each panel, the scatterplot reflects the connectivity from cells in the presynaptic subclass to cells in the postsynaptic subclass. Each dot is a single connection, with larger dots having more synapses. The location of each dot corresponds to the depth of the pre- and post-synaptic cell bodies. Stem plots on top and side indicate the net synaptic inputs and net synaptic outputs of each cell in each subclass.

bioRxiv preprint doi: https://doi.org/10.1101/2023.01.23.525290; this version posted February 14, 2023. The copyright holder for this preprint (which was not certified by peer review) is the author/funder, who has granted bioRxiv a license to display the preprint in perpetuity. It is made available under aCC-BY 4.0 International license.

Extended Data Figure 5

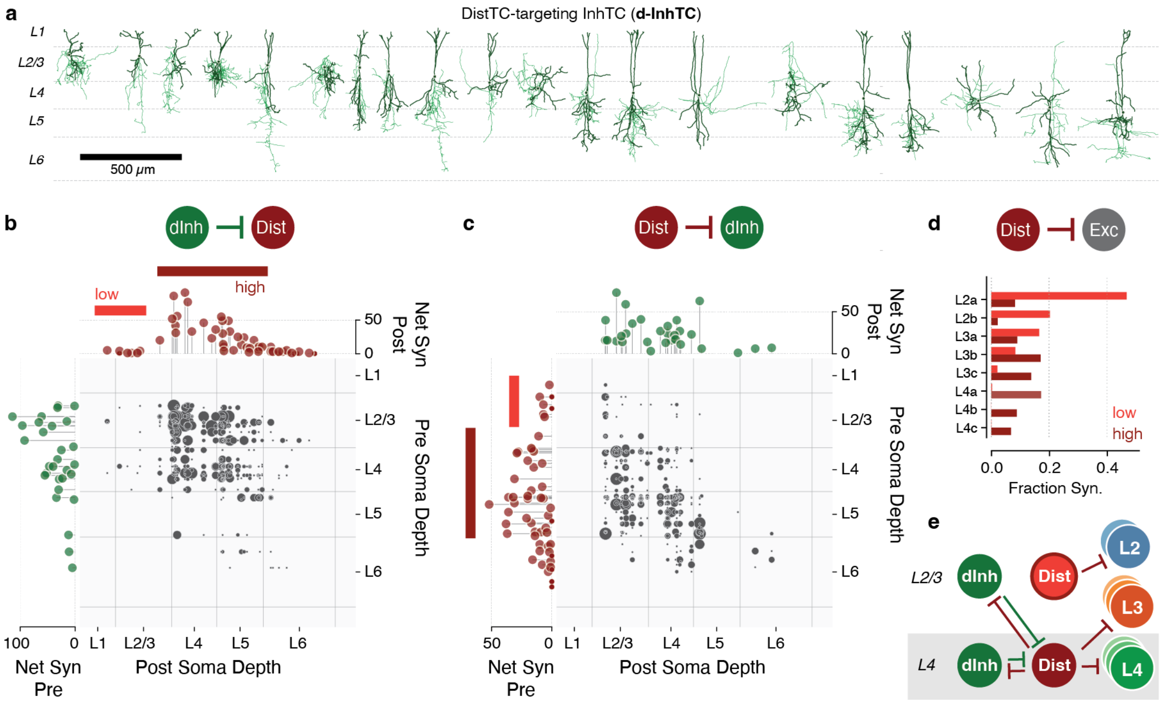

Extended Data Figure 5. A laminar-specific circuit for InhTC^{dist} cells. **a)** Morphology of all InhTC that preferentially target DistTCs. Cells are sorted by soma depth. **b)** Connectivity dotplot for synapses from InhTC^{dist} onto DistTCs. In the grid, each dot represents a connection from one InhTC onto one DistTC, with the number of synpases indicated by dot size. The location of the dot corresponds to the soma depth of the pre- and post-synaptic cells. Stem plots on top and side indicate the net synaptic inputs and net synaptic outputs of each InhTC^{dist} and DistTC. Note that DistTCs in layer 2/3 receive little input from InhTCs, compared to those in layer 4 and upper layer 5. **c)** Connectivity scatterplot for synapses from DistTCs onto InhTC^{dist}, as in **b**. Note that the DistTCs in layer 2/3 also form few synaptic outputs onto InhTC^{dist}. **d)** Distribution across M-types of synaptic outputs across low-connection DistTCs and high-connection DistTCs. **e)** Connectivity cartoon suggested by this data.

bioRxiv preprint doi: https://doi.org/10.1101/2023.01.23.525290; this version posted February 14, 2023. The copyright holder for this preprint (which was not certified by peer review) is the author/funder, who has granted bioRxiv a license to display the preprint in perpetuity. It is made available under aCC-BY 4.0 International license.

Extended Data Figure 6

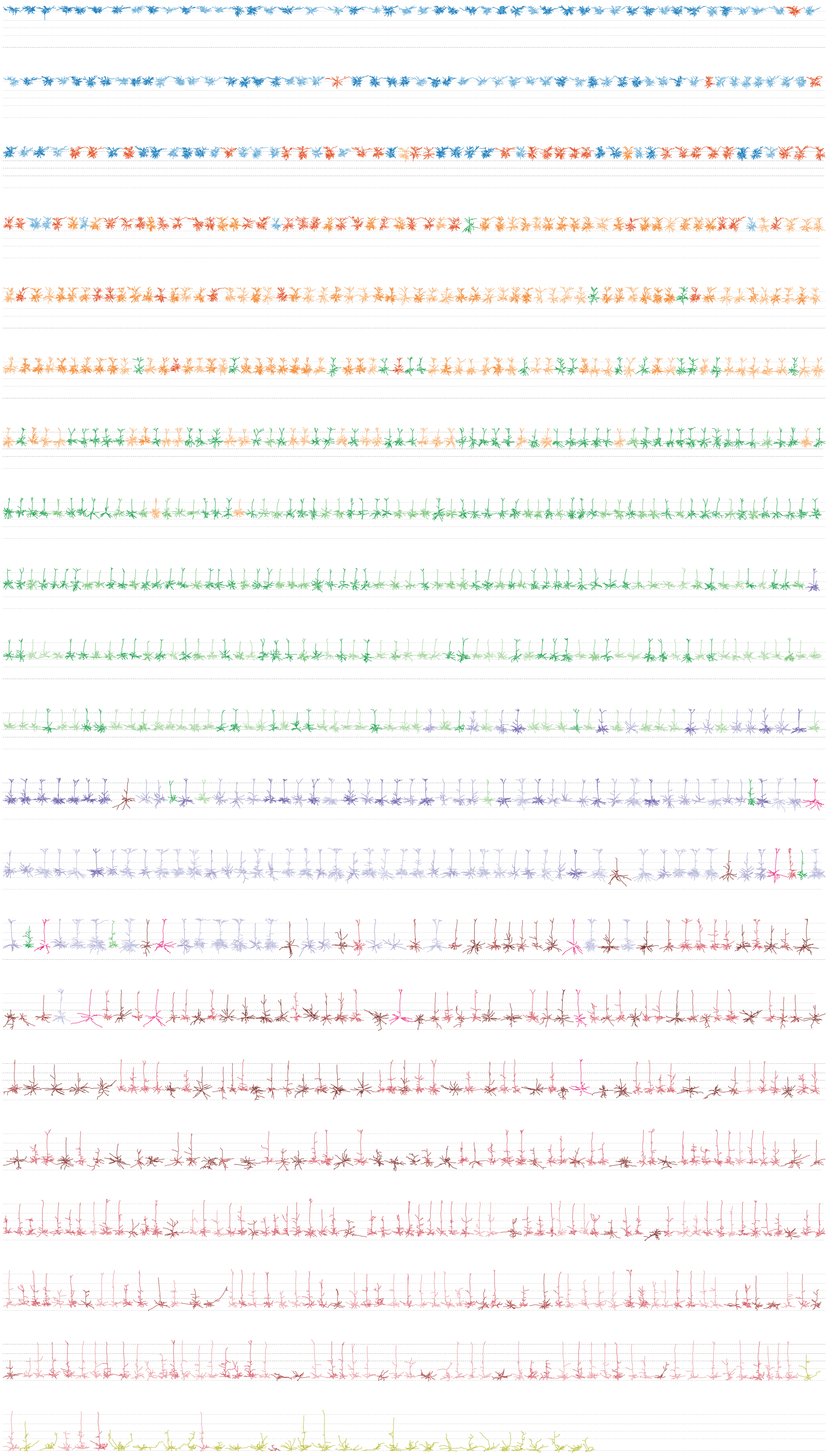

Extended Data Figure 6. See next page

bioRxiv preprint doi: https://doi.org/10.1101/2023.01.23.525290; this version posted February 14, 2023. The copyright holder for this preprint (which was not certified by peer review) is the author/funder, who has granted bioRxiv a license to display the preprint in perpetuity. It is made available under aCC-BY 4.0 International license.

Extended Data Figure 6. (Previous page.) Morphology of all excitatory neurons, sorted by soma depth and colored by anatomical class.

bioRxiv preprint doi: https://doi.org/10.1101/2023.01.23.525290; this version posted February 14, 2023. The copyright holder for this preprint (which was not certified by peer review) is the author/funder, who has granted bioRxiv a license to display the preprint in perpetuity. It is made available under aCC-BY 4.0 International license.

Extended Data Figure 7

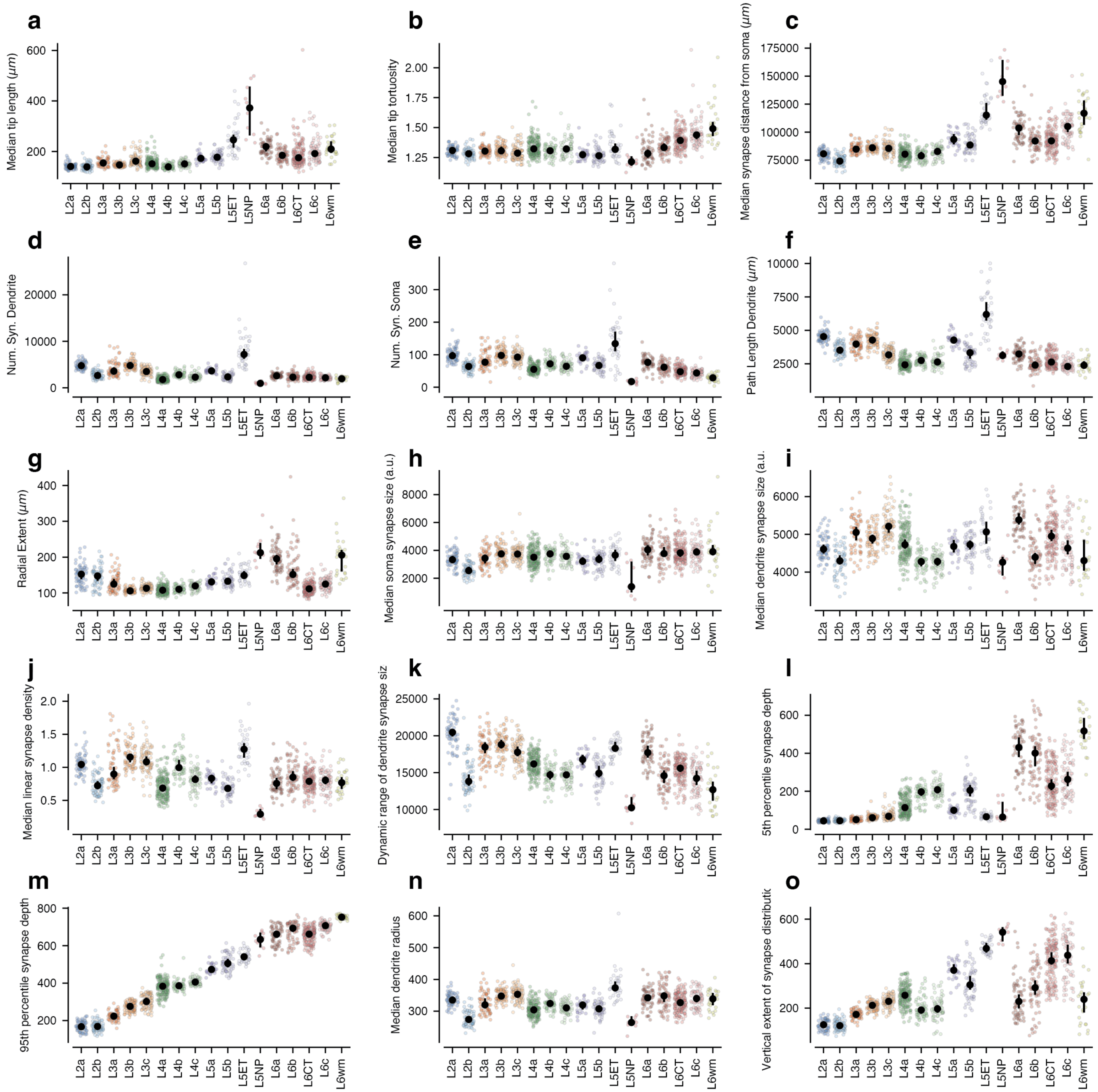

Extended Data Figure 7. Dendritic features — properties. Features used for classifying M-types, in addition to data-driven components shown in Extended Data Figure 8. See Methods for full definitions of each feature. Colored dots are individual cells, black dots indicate median, and error bars are a bootstrapped 95% confidence interval of the median. **a)** Median branch tip length. **b)** Median tip tortuosity. **c)** Median input synapse distance from soma. **d)** Number of dendritic synaptic inputs (not including soma). **e)** Number of synaptic inputs on soma. **f)** Net path length of the dendritic arbor. **g)** Radial extent of dendritic arbor, measured as a function of distance from the pia to white matter axis.

bioRxiv preprint doi: https://doi.org/10.1101/2023.01.23.525290; this version posted February 14, 2023. The copyright holder for this preprint (which was not certified by peer review) is the author/funder, who has granted bioRxiv a license to display the preprint in perpetuity. It is made available under aCC-BY 4.0 International license.

Extended Data Figure 8

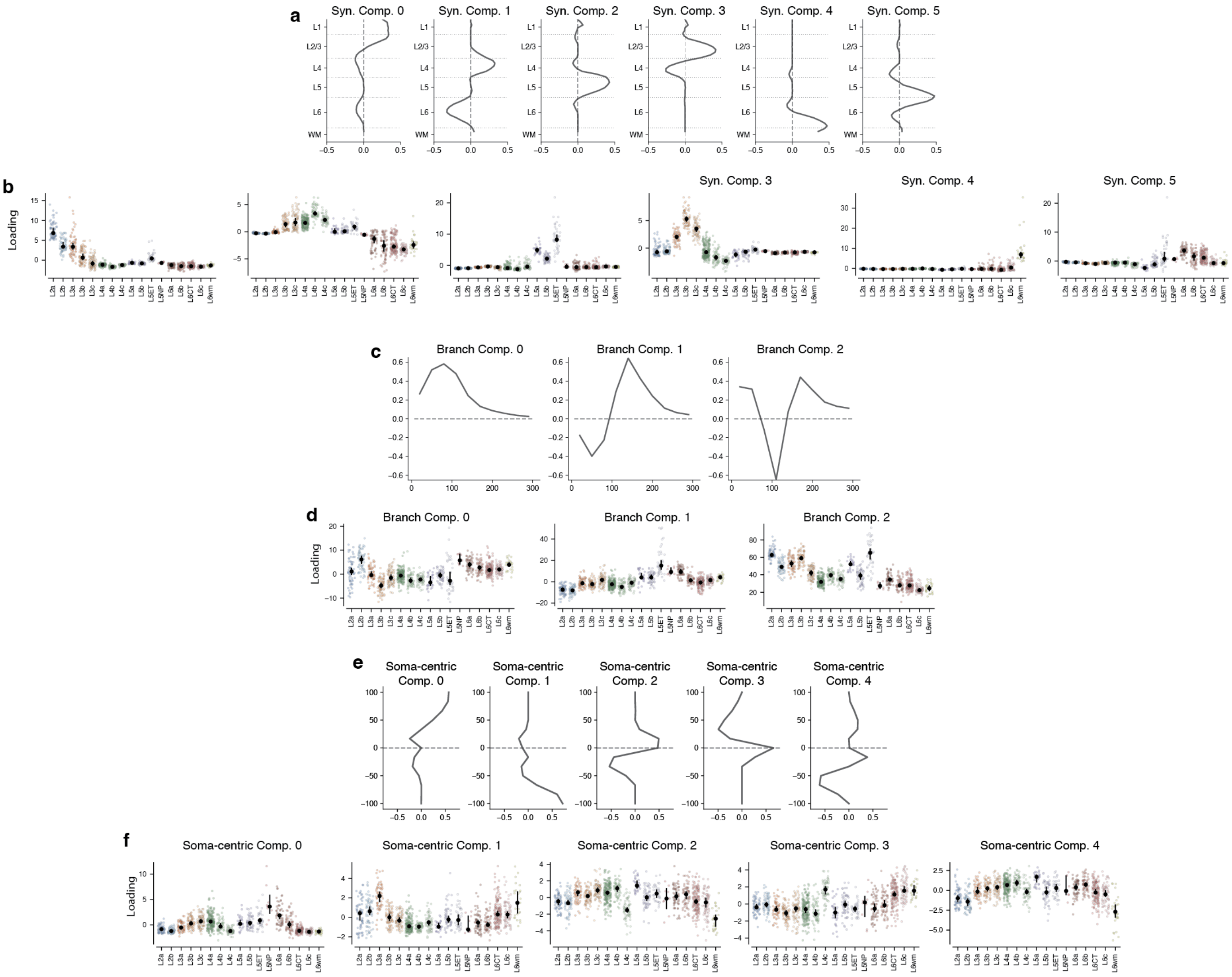

Extended Data Figure 8. Dendritic features — compartment loadings. Several of the M-type features were based on data-driven components in addition to the basic features shown in Extended Data Figure 7. **a)** SparsePCA components of synapse count across 50 depth bins (approximately 20μm per bin) across all excitatory neurons. **b)** Loadings of each resulting M-type cluster onto the components in **a**. Each colored dot is a cell, black dots are median with error bars indicating bootstrapped confidence interval of the median. **c)** Singular value decomposition (SVD) components of the number of distinct branches as a function of distance from the soma in 30 μm increments. **d)** Loadings of each resulting M-type cluster onto the branching SVD components in **c**. **e)** SparsePCA components of synapse count as a function of depth relative to the soma location, measured in 13 uniform bins from 100 μm below the soma to 100 μm above the soma. **f)** Loadings of each resulting M-type cluster onto the soma-centric synapse components in **e**.

bioRxiv preprint doi: https://doi.org/10.1101/2023.01.23.525290; this version posted February 14, 2023. The copyright holder for this preprint (which was not certified by peer review) is the author/funder, who has granted bioRxiv a license to display the preprint in perpetuity. It is made available under aCC-BY 4.0 International license.

Extended Data Figure 9

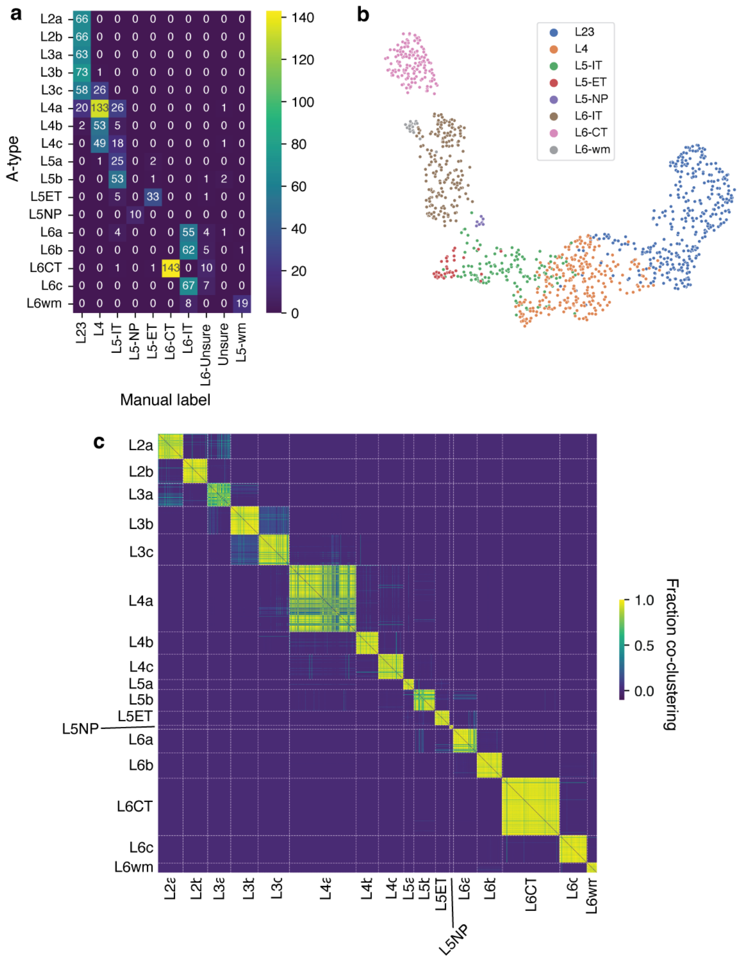

Extended Data Figure 9. M-type clustering and manual labels. **a)** Matrix of manual labels (x-axis) vs M-types. **b)** UMAP representation of features, colored by manually labeled cell types. **c)** Co-clustering matrix of excitatory cells, indicating ther number of times a pair of cells was clustered together by iterations of phenograph. Cells are ordered by subsequent agglomerative clustering on this matrix.

bioRxiv preprint doi: https://doi.org/10.1101/2023.01.23.525290; this version posted February 14, 2023. The copyright holder for this preprint (which was not certified by peer review) is the author/funder, who has granted bioRxiv a license to display the preprint in perpetuity. It is made available under aCC-BY 4.0 International license.

Extended Data Figure 10

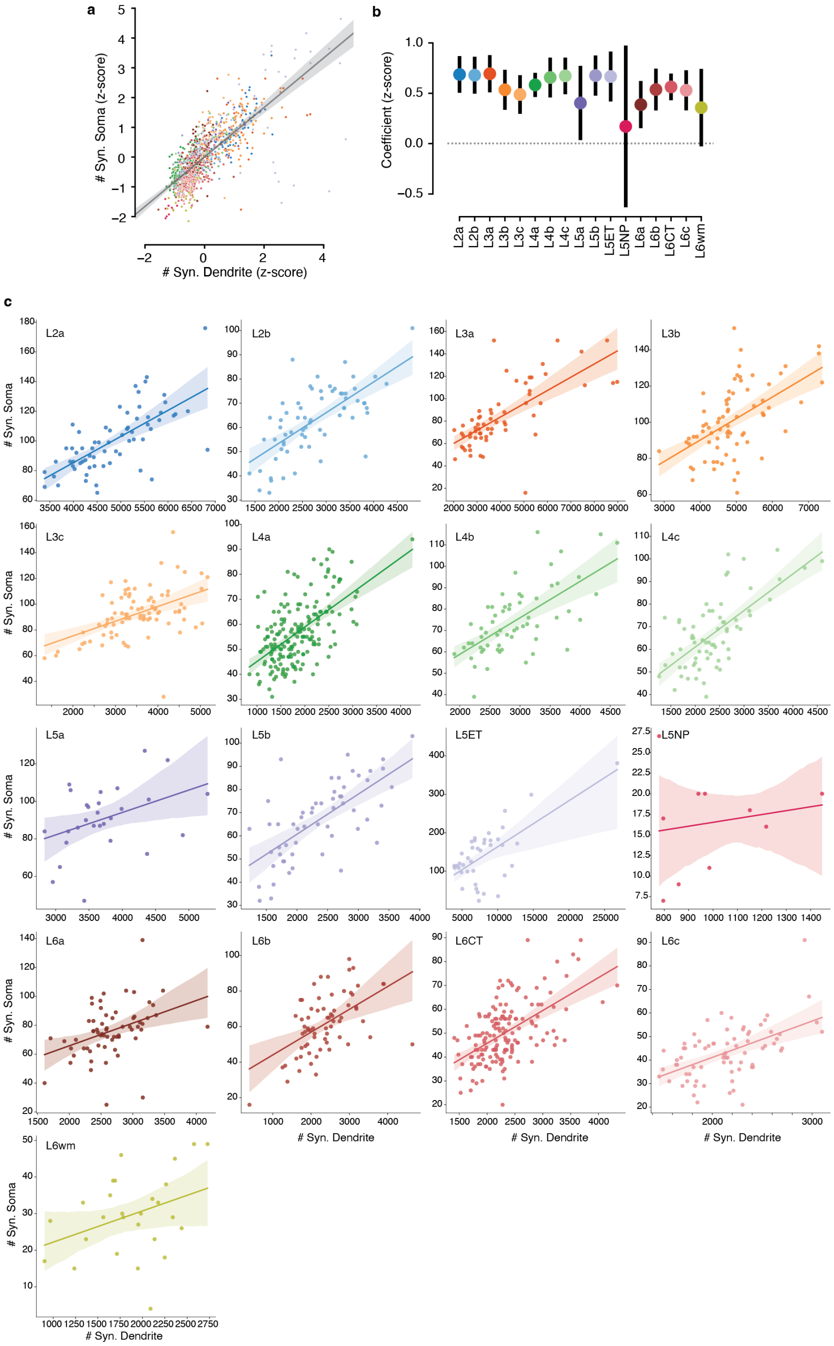

Extended Data Figure 10. Soma vs dendritic synapses across all anatomical types. **a)** Z-scored dendritic vs somatic input synapses across all excitatory neurons, colored by M-type. Black line indicates linear fit with 95% confidence intervals from bootstrapping. **b)** Individual ordinary least square fits (with confidence interval) for each M-type of z-scored dendritic synapses vs z-scored somatic synpases. With the exception of L5NP cells and deep WM-adjacent L6wm cells, all other M-types have a positive relationship between mostly-inhibitory somatic synapses and mostly excitatory dendritic synapses. **c)** Number of dendritic vs somatic input synapses for each M-type separately, linear fit line and confidence interval.

bioRxiv preprint doi: https://doi.org/10.1101/2023.01.23.525290; this version posted February 14, 2023. The copyright holder for this preprint (which was not certified by peer review) is the author/funder, who has granted bioRxiv a license to display the preprint in perpetuity. It is made available under aCC-BY 4.0 International license.

Extended Data Figure 11

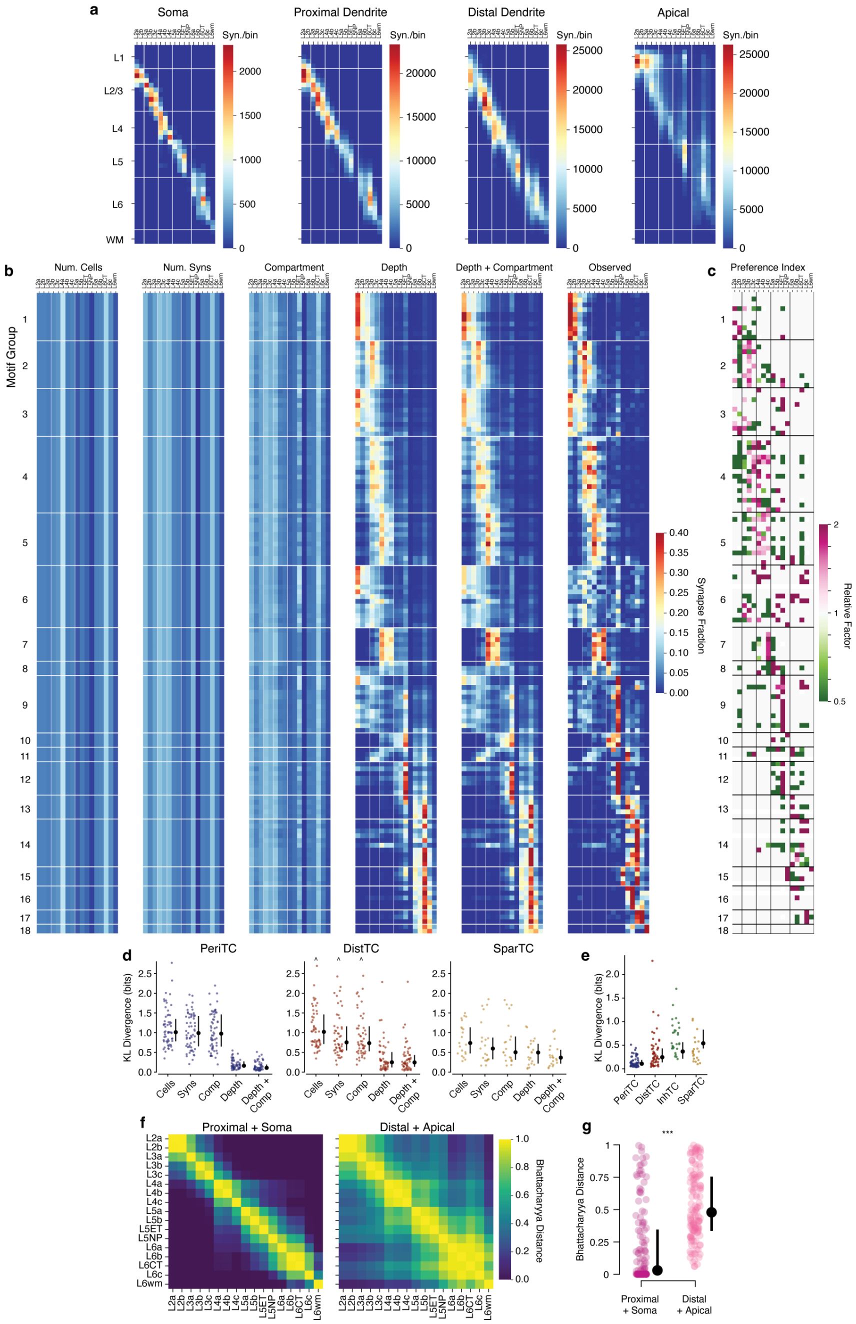

Extended Data Figure 11. See caption on next page.

bioRxiv preprint doi: https://doi.org/10.1101/2023.01.23.525290; this version posted February 14, 2023. The copyright holder for this preprint (which was not certified by peer review) is the author/funder, who has granted bioRxiv a license to display the preprint in perpetuity. It is made available under aCC-BY 4.0 International license.

Extended Data Figure 11. (Previous page.) Specificity and null models for inhibitory connectivity. **a)** Number of synapses per M-type, compartment, and depth bin. These values were used as a the baseline against which to compare synaptic output distributions for each inhibitory neuron. **b)** Expected value of each presynaptic inhibitory neuron according to an increasingly complex set of null models. Each row represents the fraction of synaptic outputs from a given inhibitory neuron (ordered as in Figure 5e), distributed across excitatory M-types. From the left: 1) Synaptic outputs were proportional to the number of cells in a given M-type, regardless of location in space. This approach accounts for the differing cell frequency for each M-type. 2) Synaptic outputs were proportional to the net number of input synapses for a given M-type, regardless of location in space. This approach accounts for the diversity in synaptic inputs for each M-type. 3) Synaptic outputs were distributed across compartments for each inhibitory cell as observed, and distributed across M-types for each compartment separately. This approach accounts for the observed differences in compartment preference for different interneurons. 4) Synaptic outputs were distributed across M-types within each of 50 depth bins, matching the observed depth distribution of synaptic outputs for each inhibitory neuron. This approach accounts for the spatial distribution of synapses, but not compartment targeting. 5) Synaptic outputs were distributed across M-types within both depth bins and compartments, matching the observed distribution of both. This approach accounts for both the spatial distribution of synapses and compartment targeting, and is the most complete model considered here. At the far right, the observed distribution on the same scale, repeating the data in Figure 5. **c)** Preference index for all cells, as described in the main text. Purple values have the observed number of output synapses significantly higher than a null model with matched compartment and depth targeting, while green are significantly less. Non-significant preference index values are treated as 0 . **d)** Difference between the observed distribution and the null model distribution for each cell as measured by the Kullback-Leibler divergence (from observed distribution to null distribution), by inhibitory subclass. Each colored dot is a cell, black dots are median with error bars indicating a 95% confidence interval based on a bootstrap. **e)** Comparison between the most complete null model across inhibitory subclasses. The PeriTCs have the lowest KL divergence of all types, indicating that the null model best predicts their connectivity. Note also that the individual cells exhibit a range of specificity. **f)** Similarity of M-type synapse distributions in space, using the Bhattacharyya distance between the depth distribution of synaptic inputs onto soma and proximal dendrites (left) and distal and apical dendrite (right). Values closer to 1 indicate more similar distributions, values closer to 0 indicate more distinct distributions. **g)** All Bhattacharyya distance comparisons in **e**, with colored dots indicating pairs of distinct M-types, black dots indicating the median, and error bars showing a bootstrapped 95% confidence interval. Across all pairs, synaptic inputs onto the perisomatic and somatic compartments are more spatially segregated across different M-types than synaptic inputs onto distal and apical dendrites ($p = 3.0 \times 10^{-19}$, Mann-Whitney U test).

bioRxiv preprint doi: https://doi.org/10.1101/2023.01.23.525290; this version posted February 14, 2023. The copyright holder for this preprint (which was not certified by peer review) is the author/funder, who has granted bioRxiv a license to display the preprint in perpetuity. It is made available under aCC-BY 4.0 International license.

Extended Data Figure 12

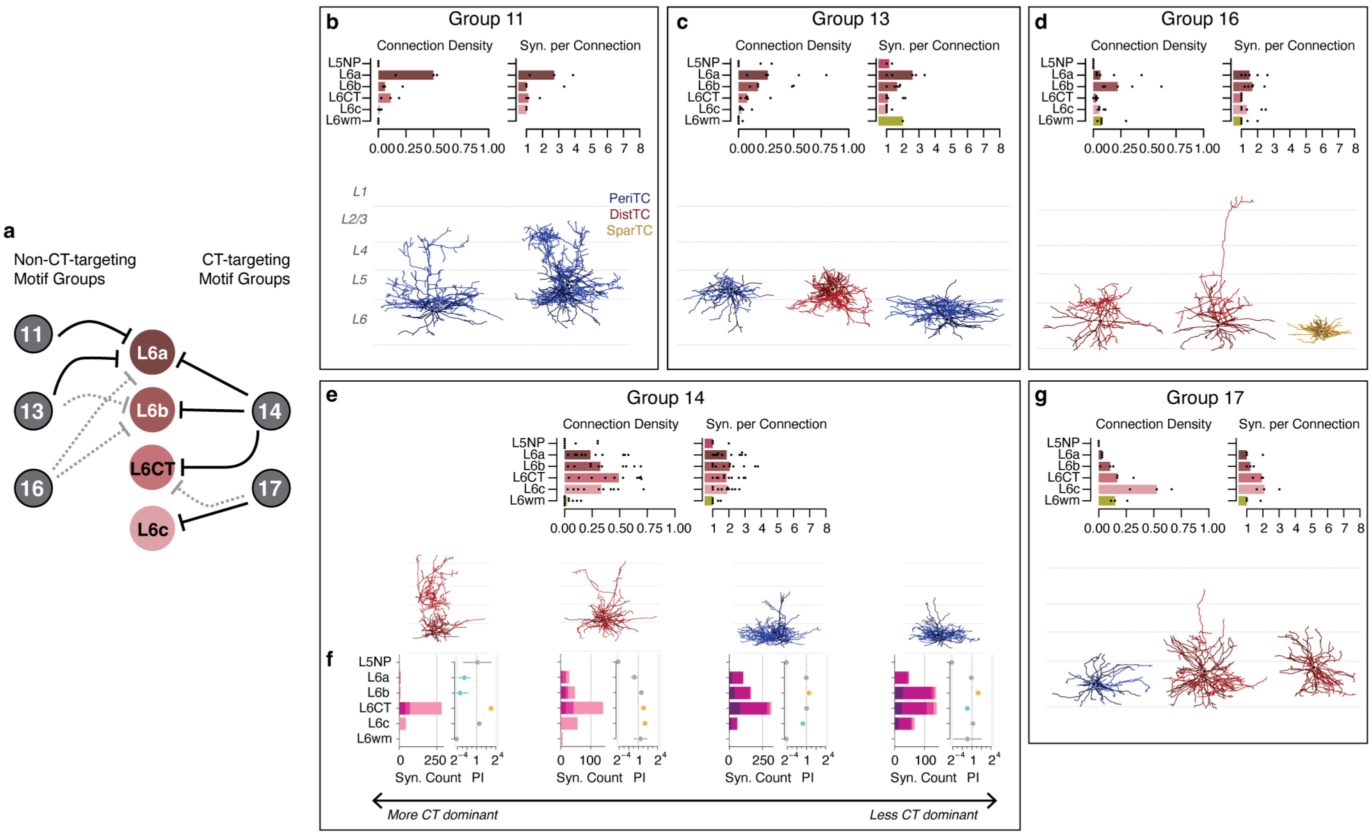

Extended Data Figure 12. Layer 6 connectivity patterns. **a)** Cartoon schematic of motif groups and their their layer 6 connectivity. Dark lines indicate connections that are strong in presynaptic fraction, connection density, and synapses per connection; gray lines indicate common but less strong connections. **b–d)** Connectivity measures for non-CT targeting motif groups 11 (**b**), 13 (**c**), and 16 (**d**), as in Figure 6a–j. Top row, left shows connection density; top row, right shows synapses per connection. Each dot indicates one cell, bars show median value within the group. Bottom row shows example morphologies colored by inhibitory cell class. **e)** Motif group 14 connectivity measures. Top and middle row as in **b–d**. **f)** Example connectivity for individual group 14 cells (matched with morphology above), ranging in how specifically they target CT neurons. Bar plots indicate synapse count by compartment. Dot and whisker plots show preference index by cell type, as in Figure5f. **g)** Motif group 17 connectivity measures, as in **b–d**.

bioRxiv preprint doi: https://doi.org/10.1101/2023.01.23.525290; this version posted February 14, 2023. The copyright holder for this preprint (which was not certified by peer review) is the author/funder, who has granted bioRxiv a license to display the preprint in perpetuity. It is made available under aCC-BY 4.0 International license.

Extended Data Cell Atlas

Extended Data Cell Atlas 1. Atlas of morphology and connectivity of all inhibitory neurons, organized by motif group. Cards depicting the connectivity and morphology of all inhibitory neurons in the column data, ordered first by motif group and then by soma depth. First row, left: Morphology of the cell, dendrites in red and axon in blue. Title reflects the subclass, cell id, and cell neuroglancer root id. First row, center: Violinplot showing the density of input synapses (red) and output synapses (blue) as a function of depth. Text labels give the total number of pre- and postsynaptic sites in the reconstruction. First row, right: Scatterplot depicting all synaptic outputs of the cell onto identified neurons in the dataset. Each dot is a synaptic output, the x-axis location reflects the depth of the target cell, and the y-axis reflects the depth of the synapse itself. Color indicates cell status: purple are excitatory neurons in the column, green are inhibitory neurons in the column, and gray are neurons outside the column. Second row, left two panels: Scatterplots showing synaptic outputs onto column excitatory cells (left, purple dots) and inhibitory cells (right, green dots), with the x-axis indicating the distance from soma of the synapse on the target neuron and y-axis indicated the depth of the synapse. Color indicates target compartment, with dots from darkest to lightest indicating soma, proximal dendrite, distal dendrite, and apical dendrite. Below, histograms of target distances from root (colored line) with cumulative distribution function indicated in gray. Second row, center: Compartment distribution of synaptic targets. Below, histogram of number of synapses by compartment. Above, preference indicate for each compartment, accounting for the depth distribution of synapses and cell type preferences of the inhibitory neuron. Orange indicates significantly more synapses than a random shuffle, blue indicates significantly fewer, and gray indicates no significant difference. Error bar indicates 95% confidence interval. Second row, right panels: Cell type distribution of synaptic outputs. Left panel shows number of synapses per cell type, with colors indicating compartments as in the center panel. Right side, preference index per target cell type, accounting for synapse depth and compartment preferences of the inhibitory neuron. Dot colors and error bars as in center panel.
