# Supplementary material for: Cell-type-specific inhibitory circuitry from a connectomic census of mouse visual cortex
Source: bioRxiv. 2023 Jan 24:2023.01.23.525290. Preprint. [Version 1] doi: 10.1101/2023.01.23.525290 (PMC9900837; doi:10.1101/2023.01.23.525290)
Supplement: Supplementary file 2 [file media-1.pdf]

Group 1

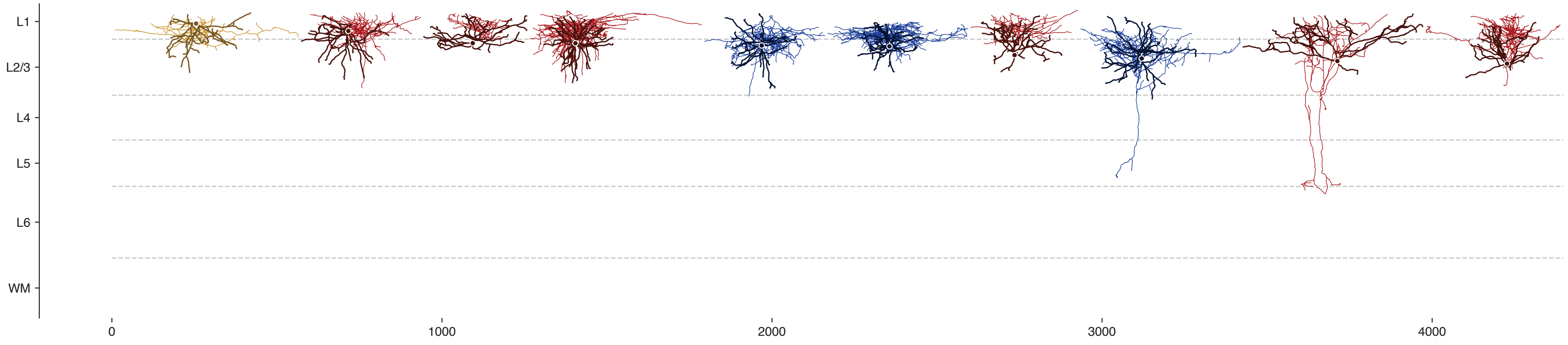

L1
L2/3
L4
L5
L6
WM
0
1000
2000
3000
4000

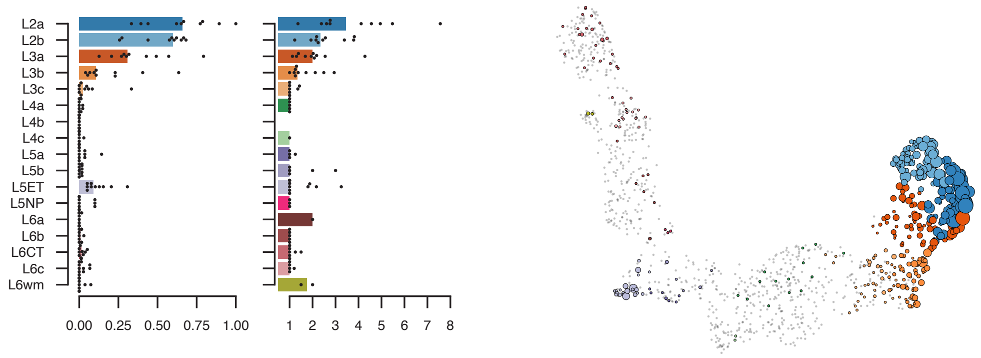

L2a
L2b
L3a
L3b
L3c
L4a
L4b
L4c
L5a
L5b
L5ET
L5NP
L6a
L6b
L6CT
L6c
L6wm
0.00 0.25 0.50 0.75 1.00
1 2 3 4 5 6 7 8

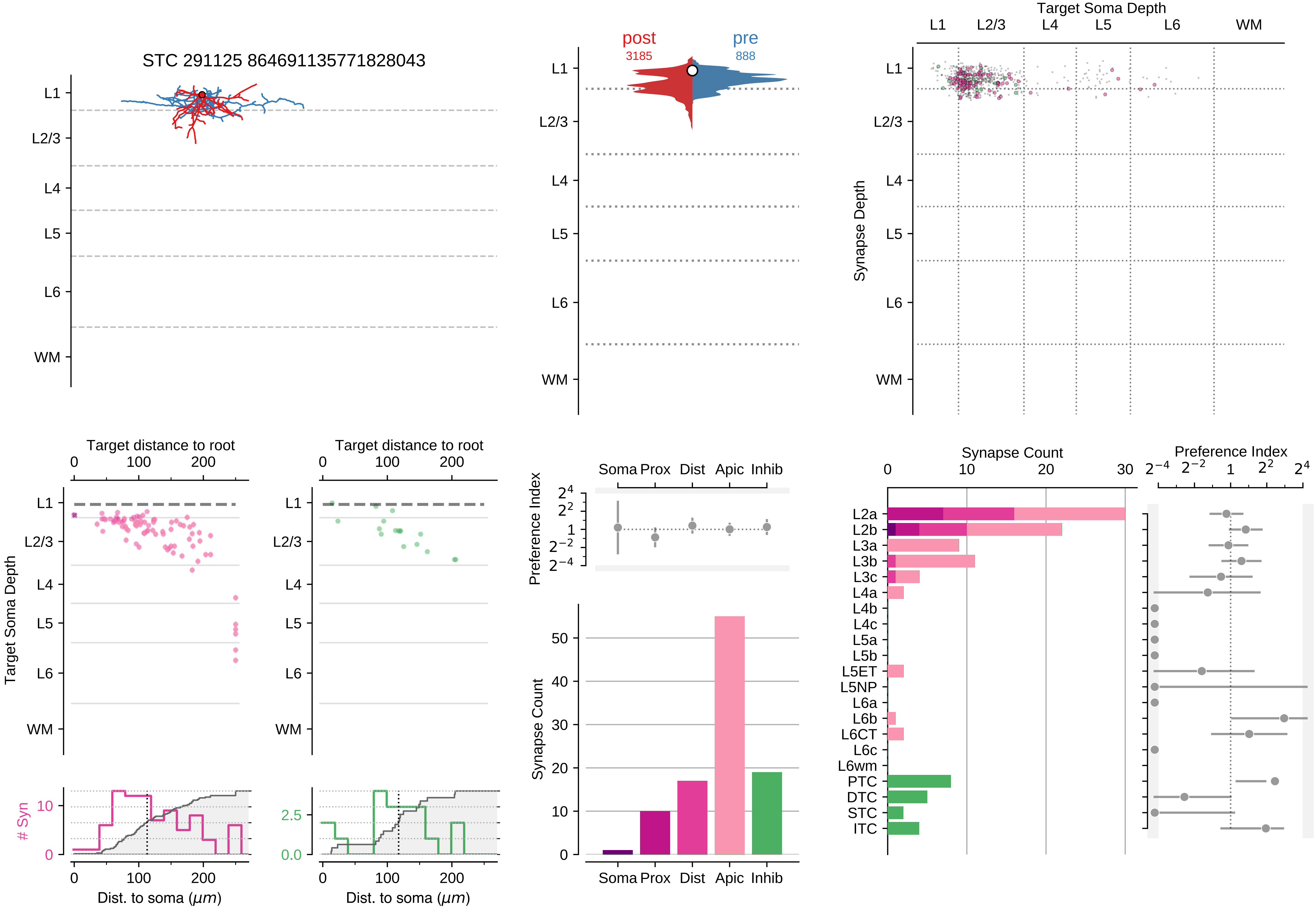

STC 291125 864691135771828043
L1
L2/3
L4
L5
L6
WM
post
3185
pre
888
Target Soma Depth
Synapse Depth
Target distance to root
Dist. to soma (μm)
Syn
Preference Index
Soma
Prox
Dist
Apic
Inhib
Synapse Count
L2a
L2b
L3a
L3b
L3c
L4a
L4b
L4c
L5a
L5b
L5ET
L5NP
L6a
L6b
L6CT
L6c
L6wm
PTC
DTC
STC
ITC

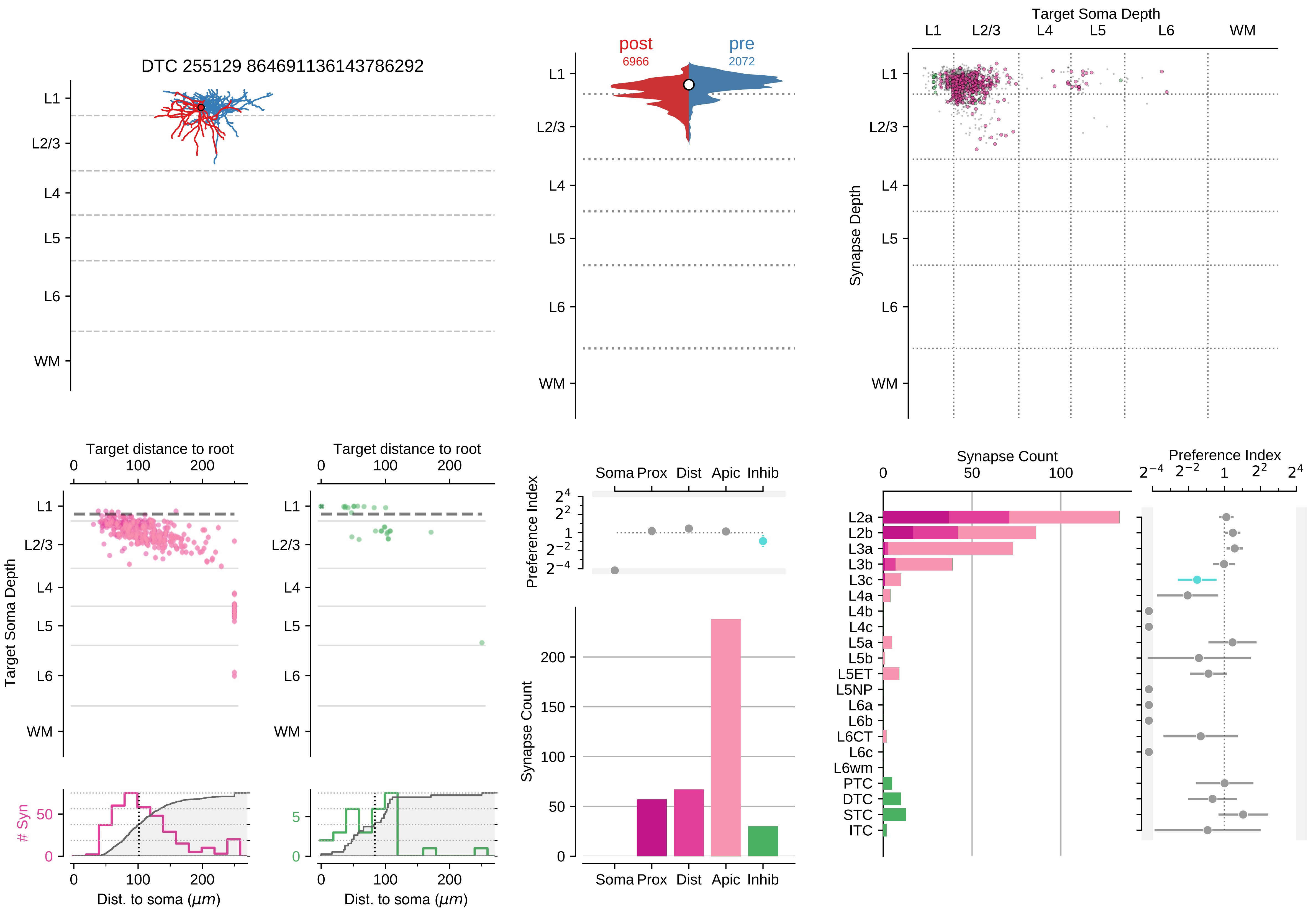

DTC 255129 864691136143786292
L1
L2/3
L4
L5
L6
WM
post
6966
pre
2072
Target Soma Depth
Synapse Depth
Target distance to root
0
100
200
Target Soma Depth
Syn
Dist. to soma (μm)
Preference Index
Soma Prox Dist Apic Inhib
Synapse Count
Synapse Count
Preference Index
L2a
L2b
L3a
L3b
L3c
L4a
L4b
L4c
L5a
L5b
L5ET
L5NP
L6a
L6b
L6CT
L6c
L6wm
PTC
DTC
STC
ITC

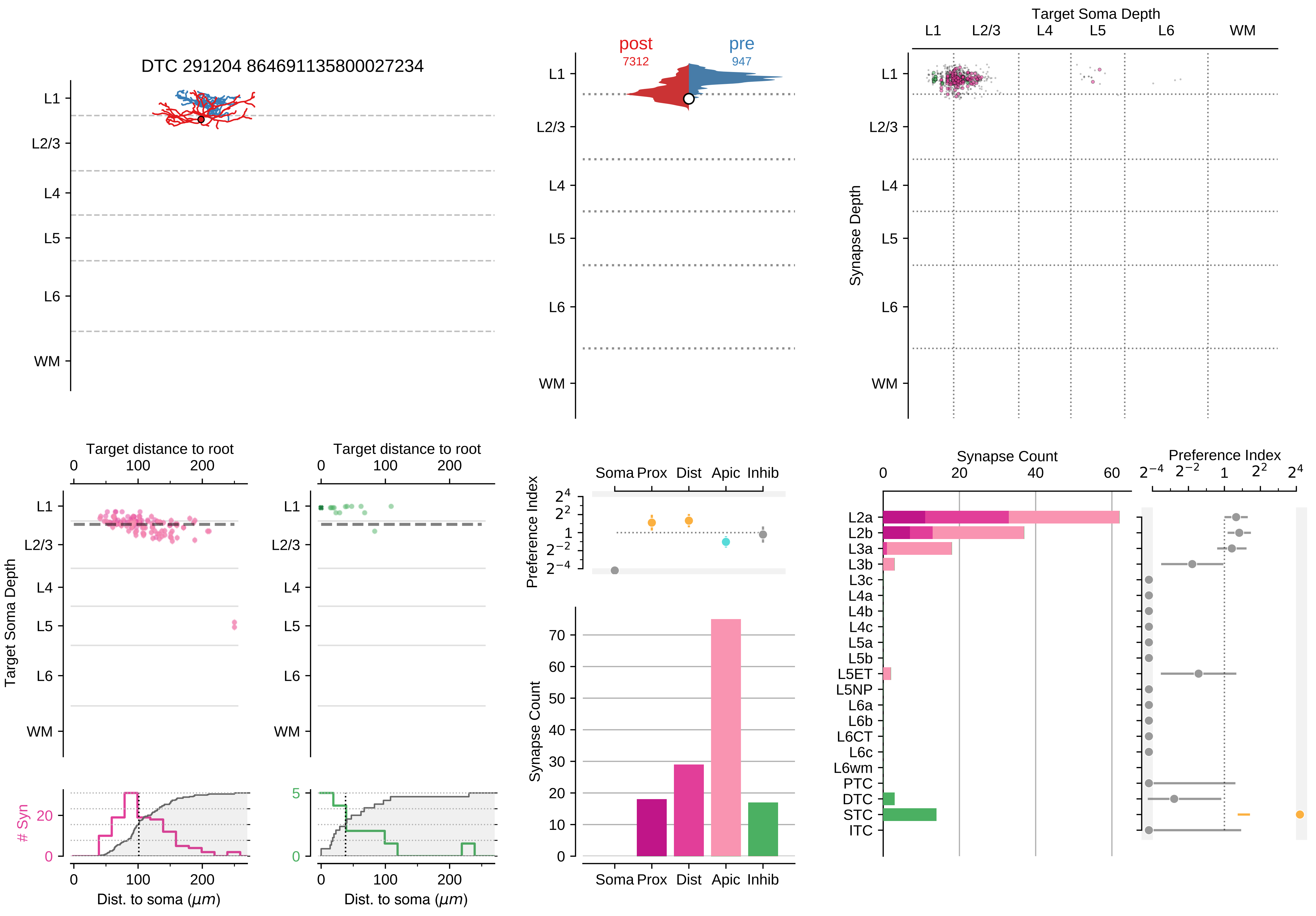

DTC 291204 864691135800027234
post
7312
pre
947
Target Soma Depth
Synapse Depth
L1
L2/3
L4
L5
L6
WM
Target distance to root
Target Soma Depth
Syn
Dist. to soma (μm)
Preference Index
Soma Prox
Dist
Apic
Inhib
Synapse Count
L2a
L2b
L3a
L3b
L3c
L4a
L4b
L4c
L5a
L5b
L5ET
L5NP
L6a
L6b
L6CT
L6c
L6wm
PTC
DTC
STC
ITC

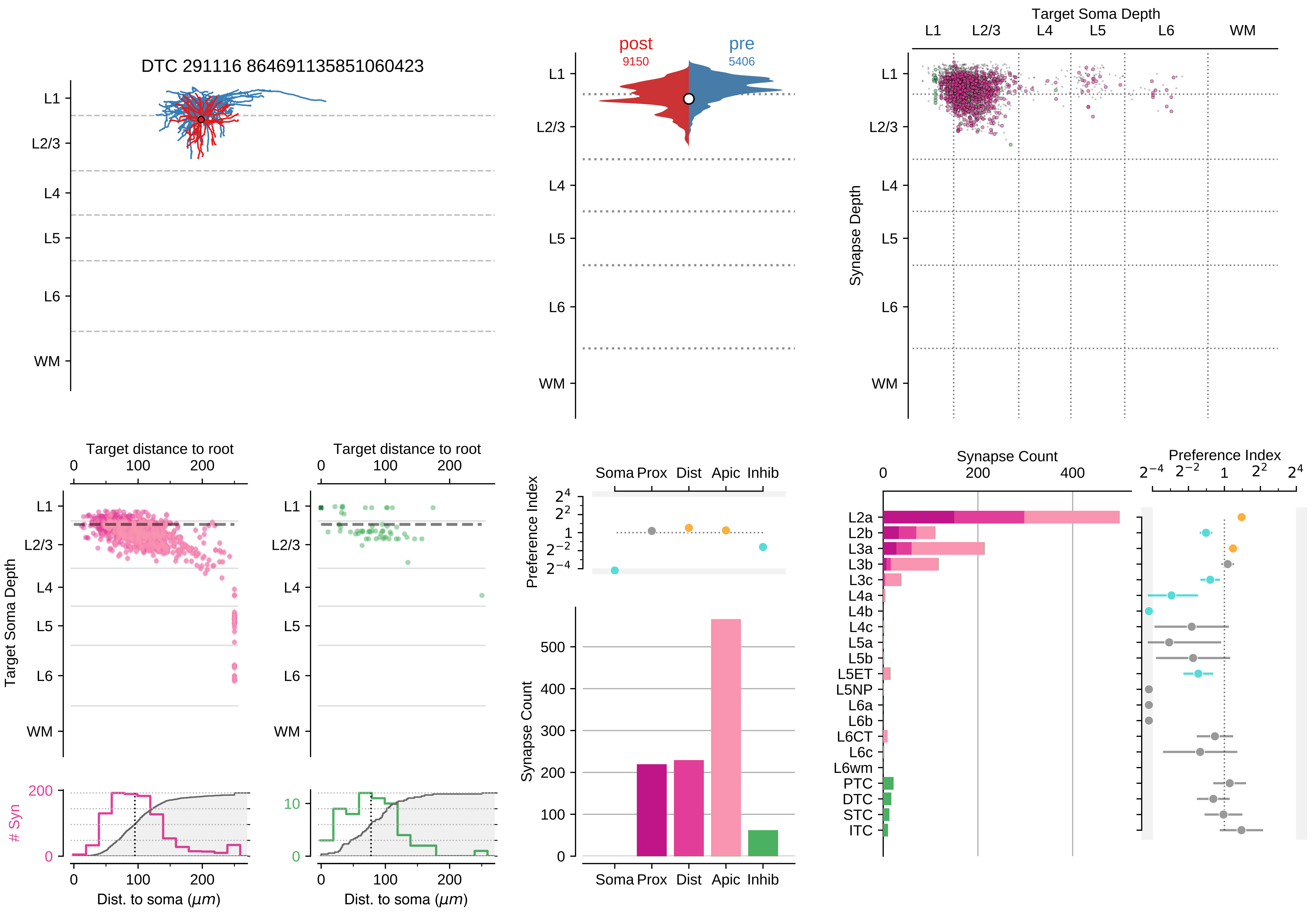

DTC 291116 864691135851060423
L1
L2/3
L4
L5
L6
WM
post
9150
pre
5406
Target Soma Depth
Synapse Depth
Target distance to root
Target Soma Depth
Syn
Dist. to soma (μm)
Preference Index
Soma Prox Dist Apic Inhib
Synapse Count
Preference Index
L2a
L2b
L3a
L3b
L3c
L4a
L4b
L4c
L5a
L5b
L5ET
L5NP
L6a
L6b
L6CT
L6c
L6wm
PTC
DTC
STC
ITC

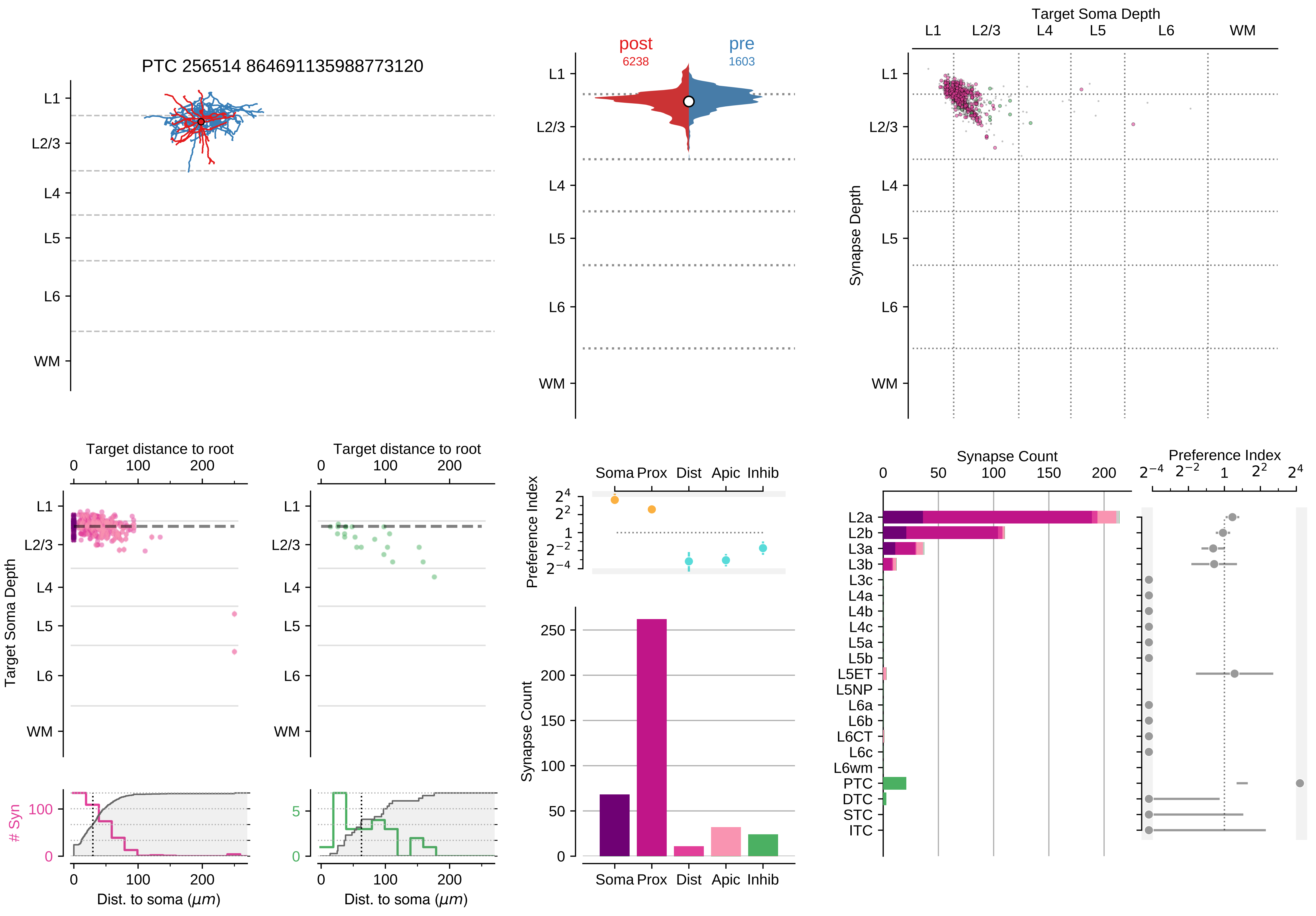

PTC 256514 864691135988773120
L1
L2/3
L4
L5
L6
WM
post
6238
pre
1603
Target Soma Depth
Synapse Depth
Target distance to root
Target Soma Depth
Syn
Dist. to soma (μm)
Preference Index
Soma
Prox
Dist
Apic
Inhib
Synapse Count
L2a
L2b
L3a
L3b
L3c
L4a
L4b
L4c
L5a
L5b
L5ET
L5NP
L6a
L6b
L6CT
L6c
L6wm
PTC
DTC
STC
ITC

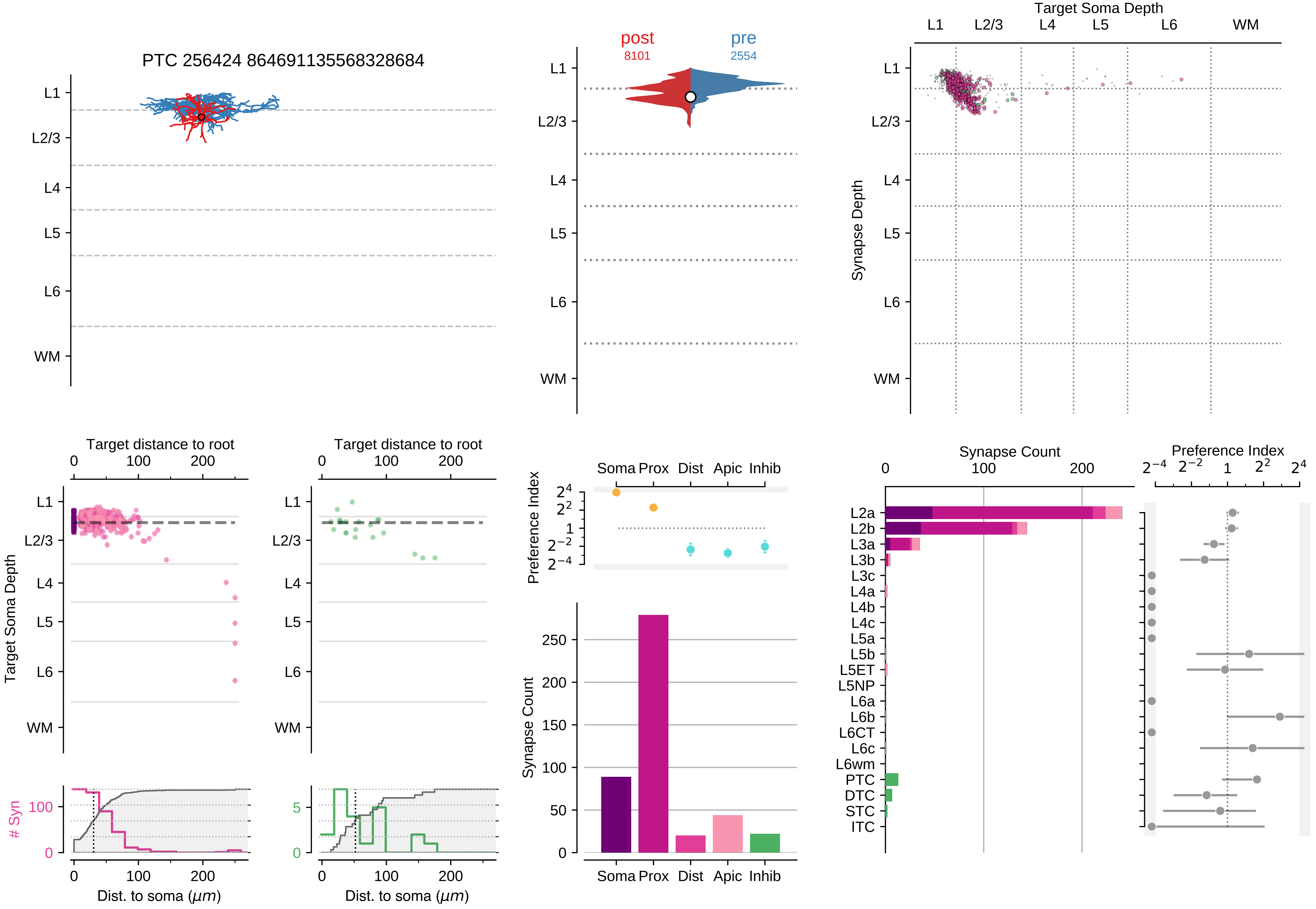
PTC 256424 864691135568328684
L1
L2/3
L4
L5
L6
WM
post
8101
pre
2554
Target Soma Depth
Synapse Depth
Target distance to root
Target Soma Depth
Syn
Dist. to soma (μm)
Preference Index
Soma Prox Dist Apic Inhib
Synapse Count
Synapse Count
Preference Index
L2a
L2b
L3a
L3b
L3c
L4a
L4b
L4c
L5a
L5b
L5ET
L5NP
L6a
L6b
L6CT
L6c
L6wm
PTC
DTC
STC
ITC

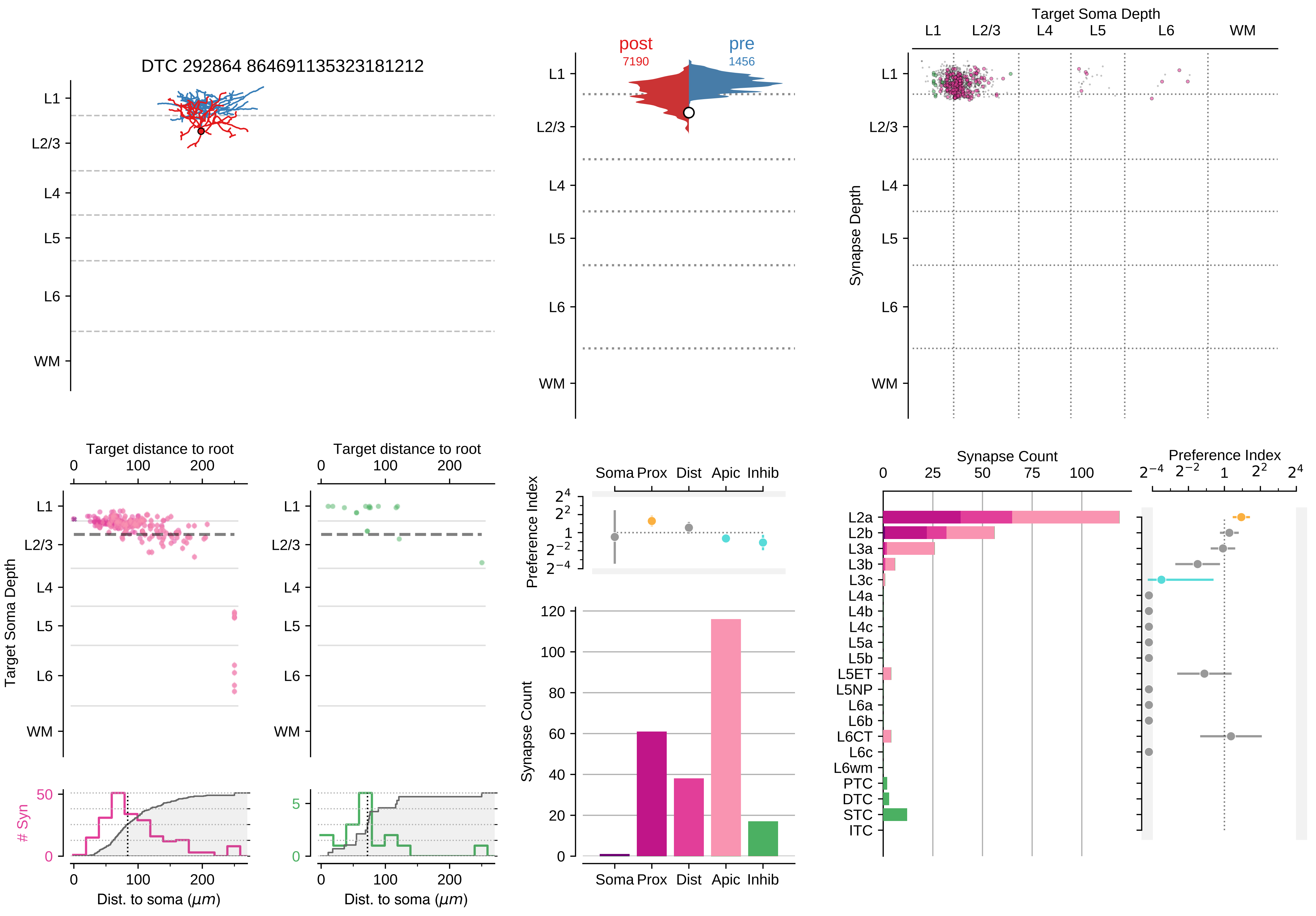
DTC 292864 864691135323181212
L1
L2/3
L4
L5
L6
WM
post
7190
pre
1456
Target Soma Depth
Synapse Depth
Target distance to root
Target Soma Depth
Syn
Dist. to soma (μm)
Preference Index
Soma Prox Dist Apic Inhib
Synapse Count
L2a
L2b
L3a
L3b
L3c
L4a
L4b
L4c
L5a
L5b
L5ET
L5NP
L6a
L6b
L6CT
L6c
L6wm
PTC
DTC
STC
ITC

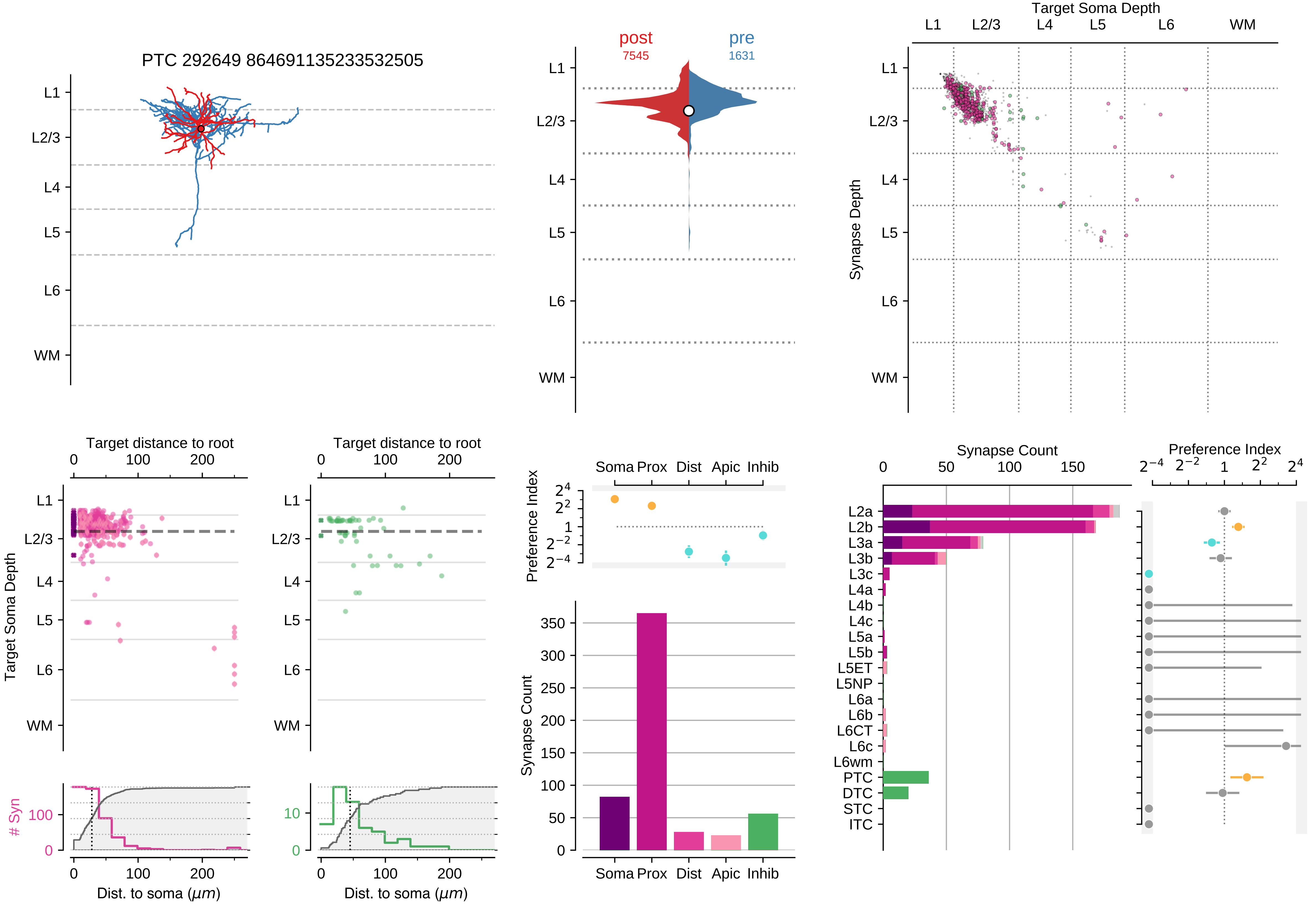

PTC 292649 864691135233532505
L1
L2/3
L4
L5
L6
WM
post
7545
pre
1631
Target Soma Depth
Synapse Depth
Target distance to root
Target Soma Depth
Syn
Dist. to soma (μm)
Soma Prox Dist Apic Inhib
Preference Index
Synapse Count
L2a
L2b
L3a
L3b
L3c
L4a
L4b
L4c
L5a
L5b
L5ET
L5NP
L6a
L6b
L6CT
L6c
L6wm
PTC
DTC
STC
ITC

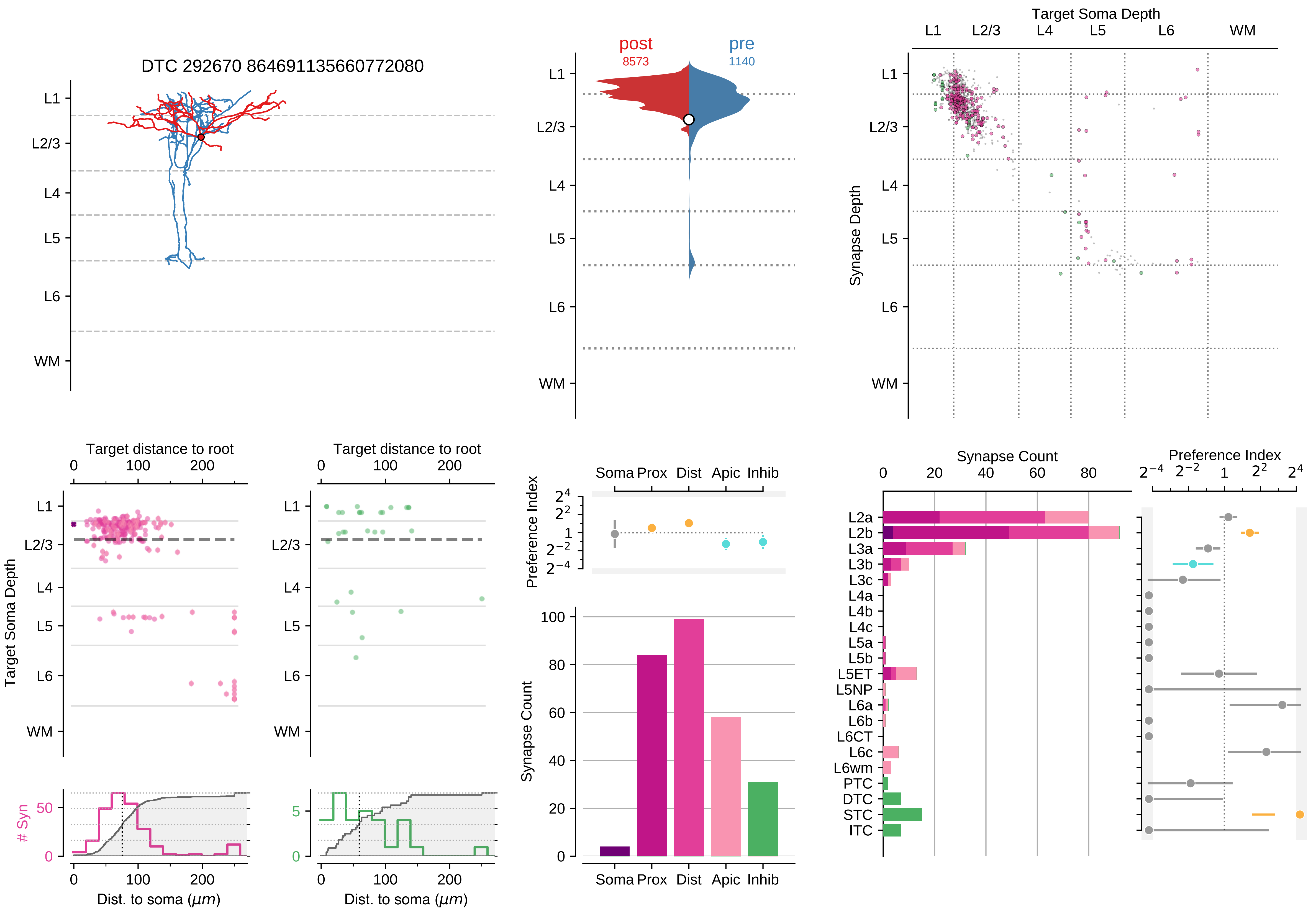
DTC 292670 864691135660772080
L1
L2/3
L4
L5
L6
WM
post
8573
pre
1140
Target Soma Depth
Synapse Depth
Target distance to root
Target Soma Depth
Syn
Dist. to soma (μm)
Preference Index
Soma Prox Dist Apic Inhib
Synapse Count
L2a
L2b
L3a
L3b
L3c
L4a
L4b
L4c
L5a
L5b
L5ET
L5NP
L6a
L6b
L6CT
L6c
L6wm
PTC
DTC
STC
ITC

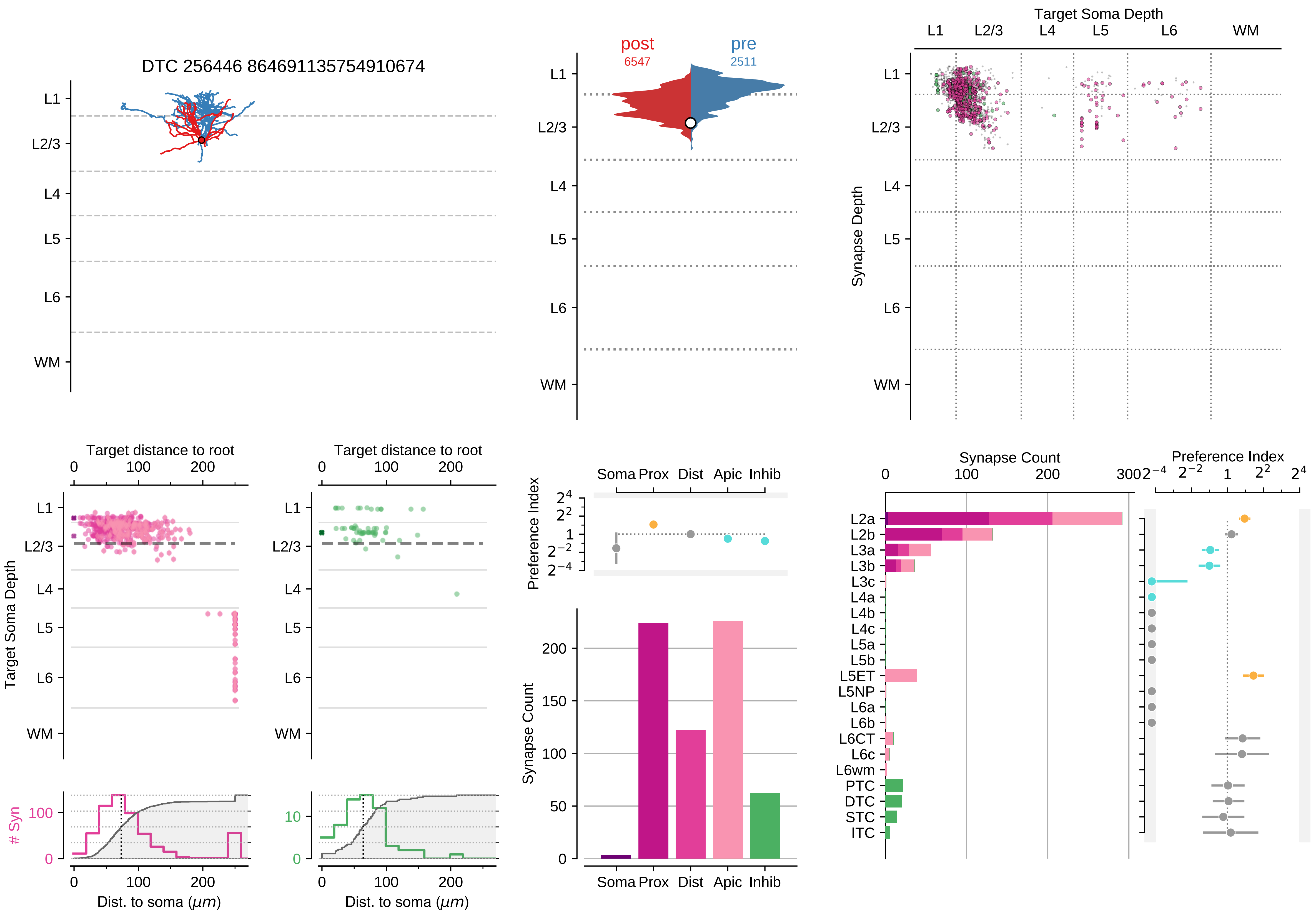

DTC 256446 864691135754910674
post
6547
pre
2511
Target Soma Depth
Synapse Depth
L1
L2/3
L4
L5
L6
WM
Target distance to root
Target Soma Depth
Dist. to soma (μm)
Syn
Preference Index
Soma Prox Dist Apic Inhib
Synapse Count
L2a
L2b
L3a
L3b
L3c
L4a
L4b
L4c
L5a
L5b
L5ET
L5NP
L6a
L6b
L6CT
L6c
L6wm
PTC
DTC
STC
ITC

Group 2

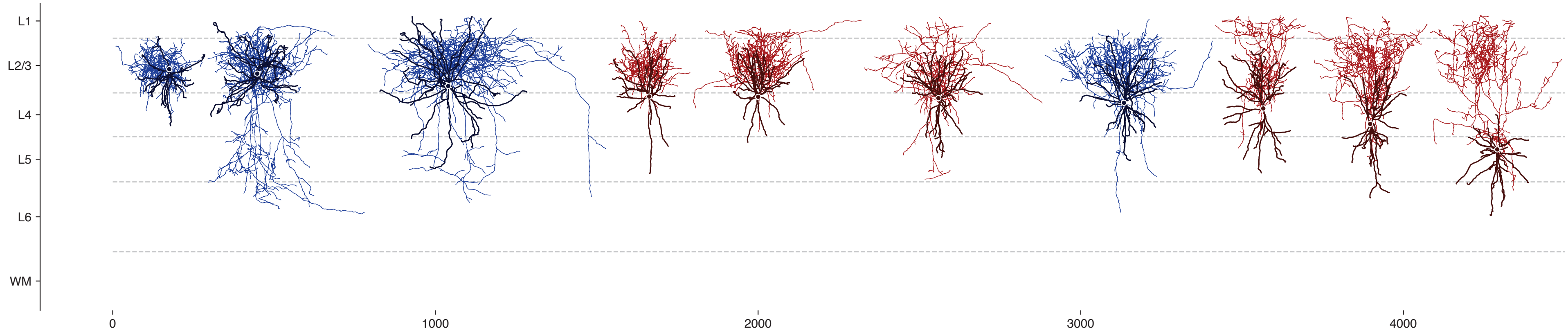
L1
L2/3
L4
L5
L6
WM
0
1000
2000
3000
4000

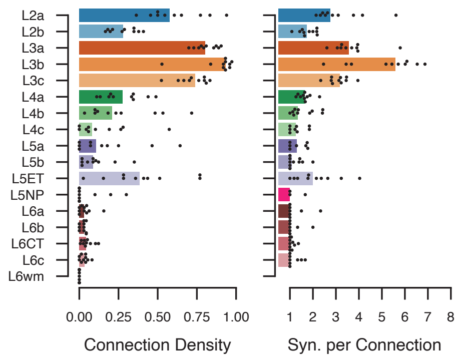
L2a
L2b
L3a
L3b
L3c
L4a
L4b
L4c
L5a
L5b
L5ET
L5NP
L6a
L6b
L6CT
L6c
L6wm
0.00 0.25 0.50 0.75 1.00
Connection Density
1 2 3 4 5 6 7 8
Syn. per Connection

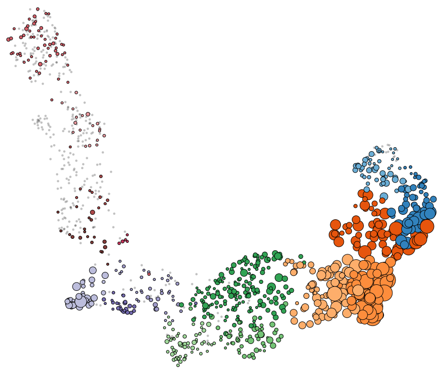

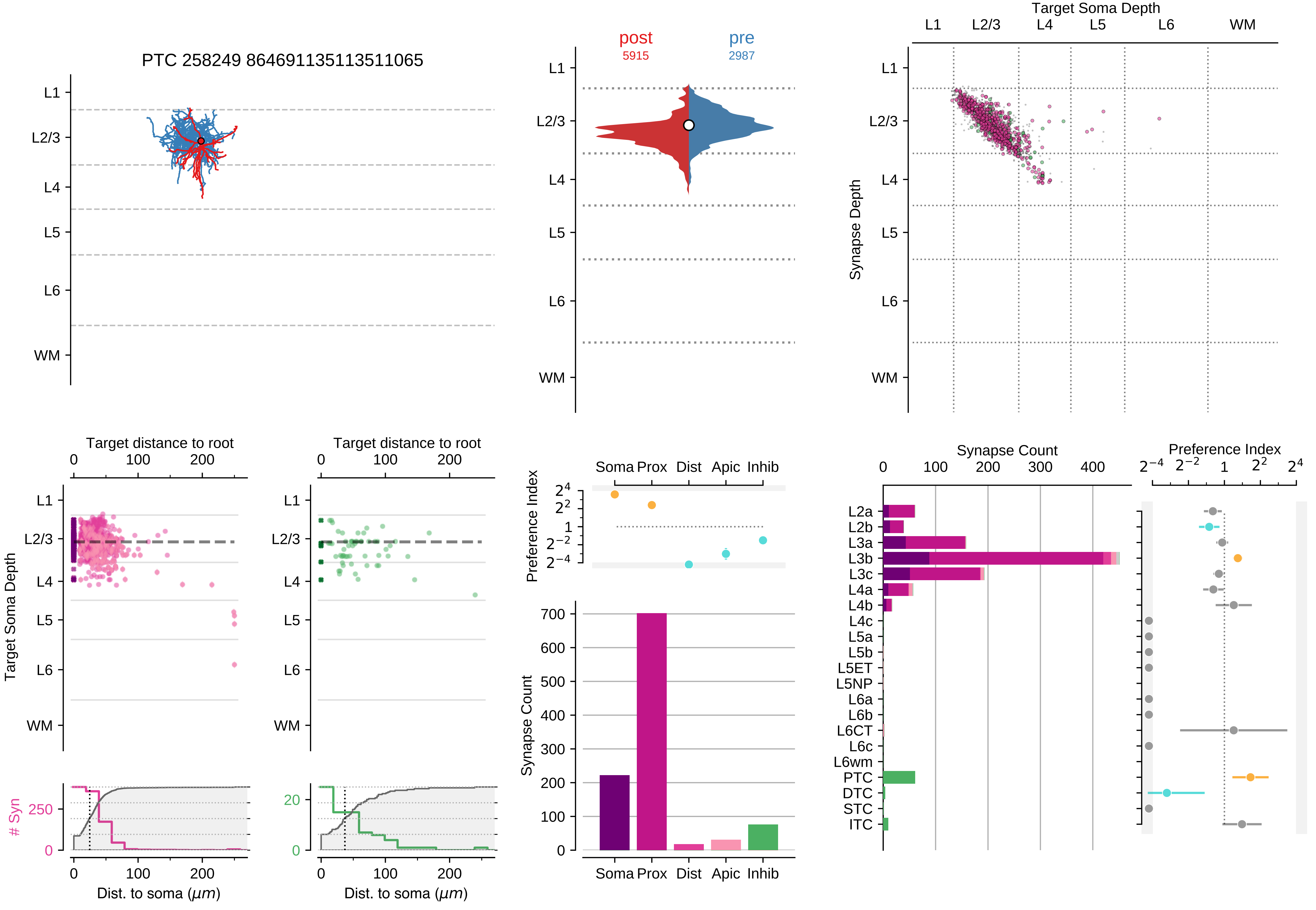

PTC 258249 864691135113511065
L1
L2/3
L4
L5
L6
WM
post
5915
pre
2987
Target Soma Depth
Synapse Depth
Target distance to root
Target Soma Depth
Dist. to soma (μm)
Syn
Preference Index
Soma
Prox
Dist
Apic
Inhib
Synapse Count
L2a
L2b
L3a
L3b
L3c
L4a
L4b
L4c
L5a
L5b
L5ET
L5NP
L6a
L6b
L6CT
L6c
L6wm
PTC
DTC
STC
ITC

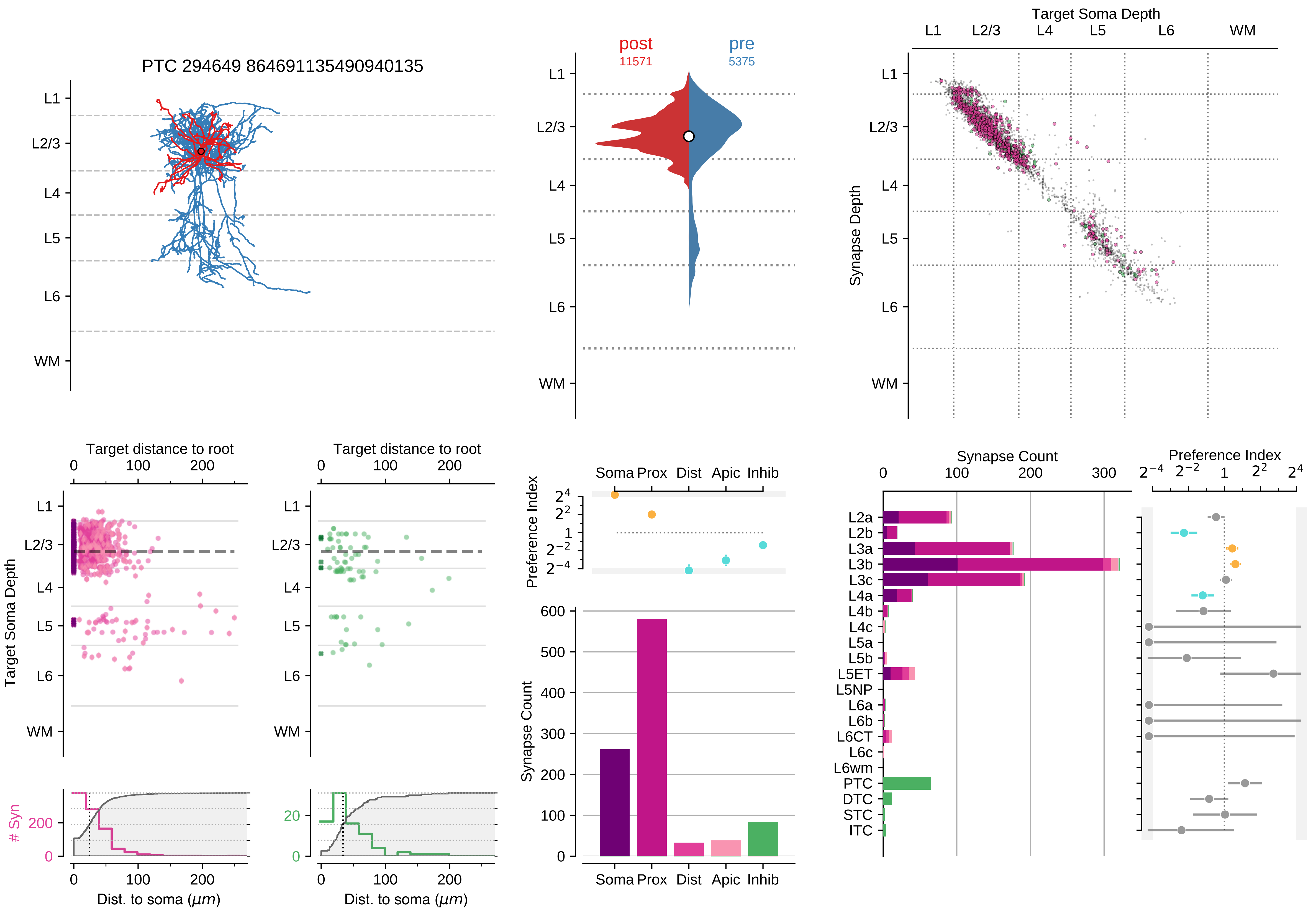

PTC 294649 864691135490940135
L1
L2/3
L4
L5
L6
WM
post
11571
pre
5375
Target Soma Depth
Synapse Depth
Target distance to root
Target Soma Depth
Syn
Dist. to soma (μm)
Preference Index
Soma Prox Dist Apic Inhib
Synapse Count
L2a
L2b
L3a
L3b
L3c
L4a
L4b
L4c
L5a
L5b
L5ET
L5NP
L6a
L6b
L6CT
L6c
L6wm
PTC
DTC
STC
ITC

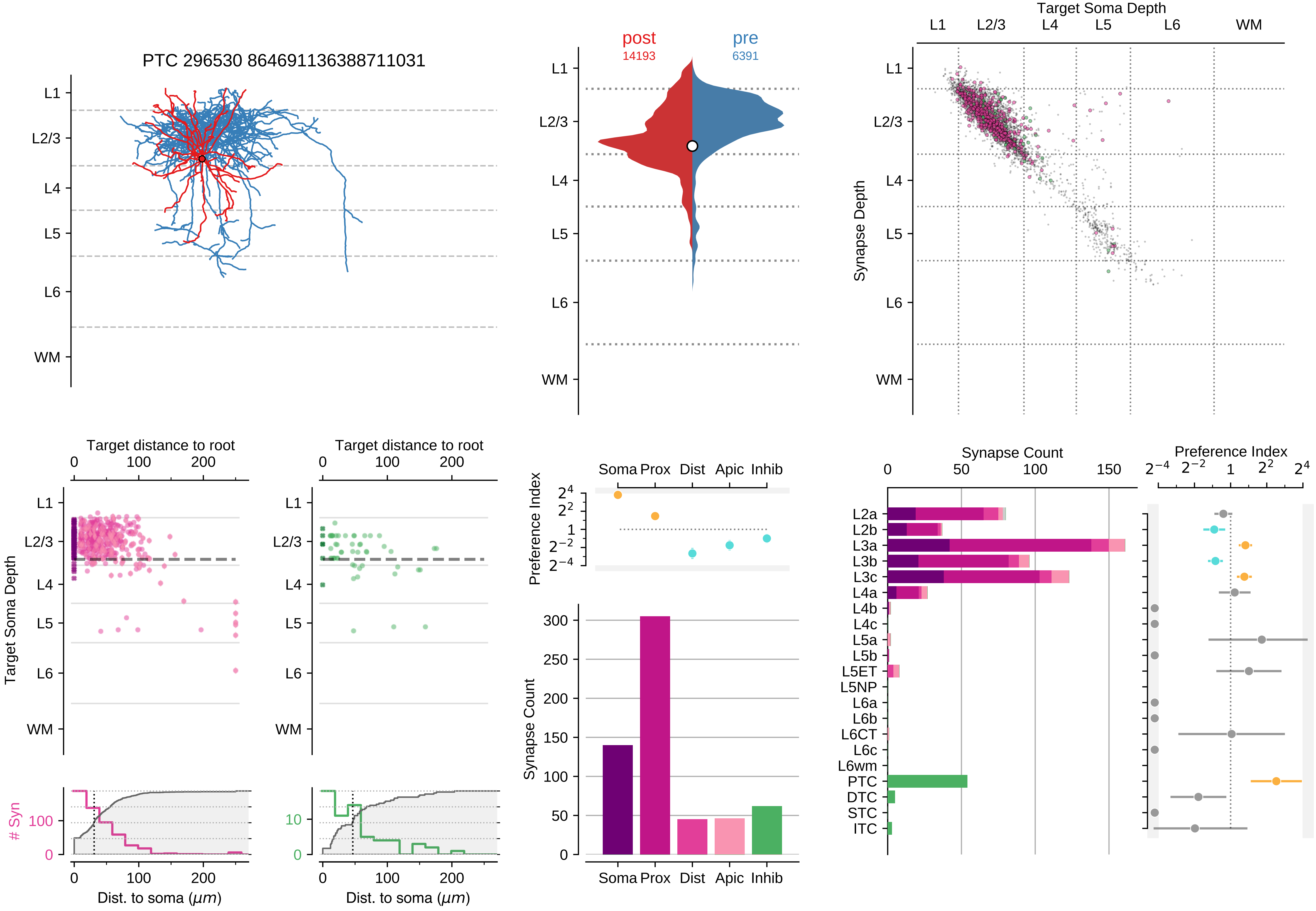

PTC 296530 864691136388711031
L1
L2/3
L4
L5
L6
WM
post
14193
pre
6391
Target Soma Depth
Synapse Depth
Target distance to root
Target Soma Depth
Syn
Dist. to soma (μm)
Preference Index
Soma
Prox
Dist
Apic
Inhib
Synapse Count
L2a
L2b
L3a
L3b
L3c
L4a
L4b
L4c
L5a
L5b
L5ET
L5NP
L6a
L6b
L6CT
L6c
L6wm
PTC
DTC
STC
ITC

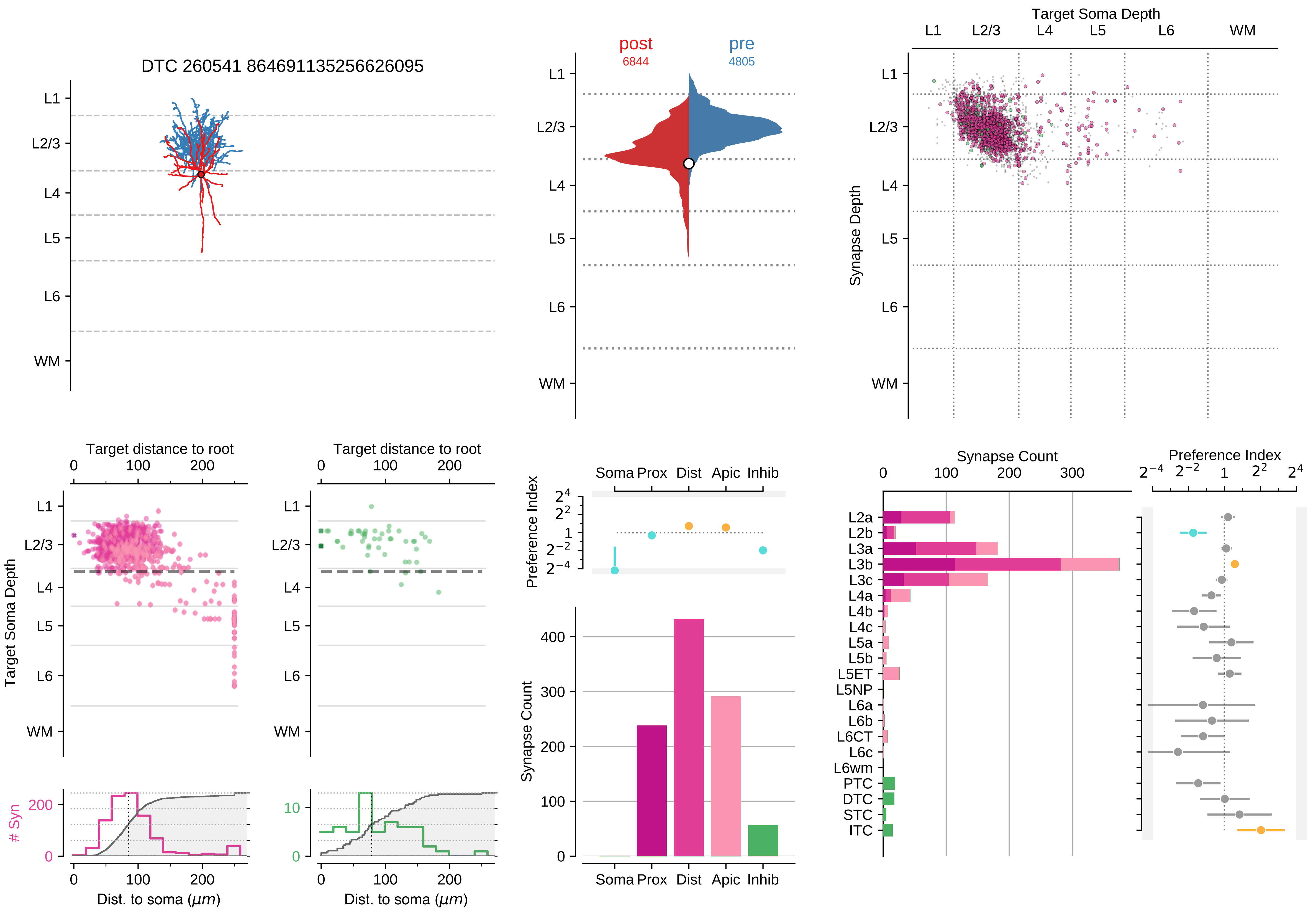

DTC 260541 864691135256626095
post
6844
pre
4805
Target Soma Depth
Synapse Depth
Target distance to root
Target Soma Depth
Syn
Dist. to soma (μm)
Preference Index
Soma Prox Dist Apic Inhib
Synapse Count
Preference Index
L1
L2/3
L4
L5
L6
WM
L2a
L2b
L3a
L3b
L3c
L4a
L4b
L4c
L5a
L5b
L5ET
L5NP
L6a
L6b
L6CT
L6c
L6wm
PTC
DTC
STC
ITC

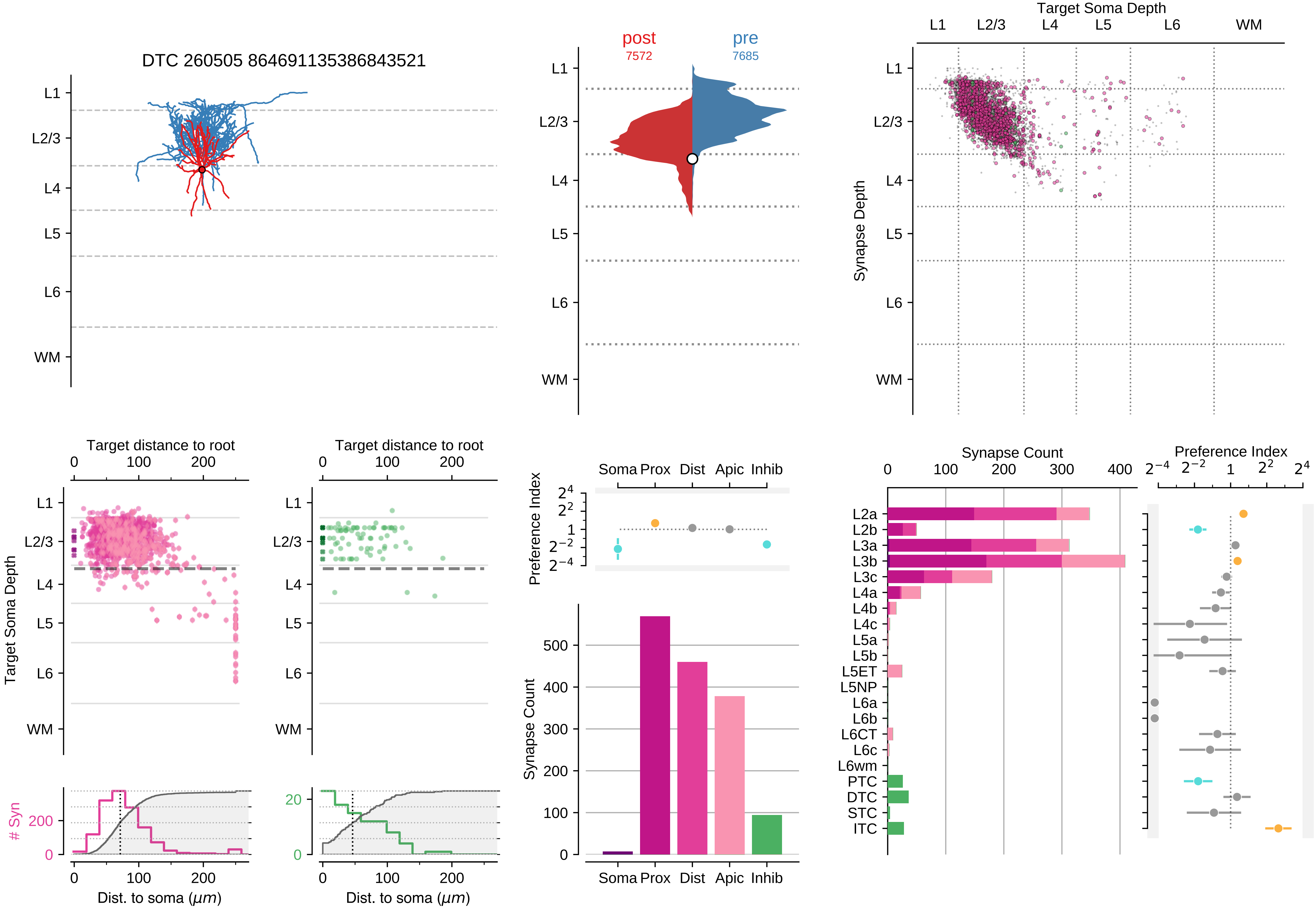
DTC 260505 864691135386843521
L1
L2/3
L4
L5
L6
WM
post
7572
pre
7685
Target Soma Depth
Synapse Depth
Target distance to root
Target Soma Depth
Syn
Dist. to soma (μm)
Preference Index
Soma
Prox
Dist
Apic
Inhib
Synapse Count
L2a
L2b
L3a
L3b
L3c
L4a
L4b
L4c
L5a
L5b
L5ET
L5NP
L6a
L6b
L6CT
L6c
L6wm
PTC
DTC
STC
ITC

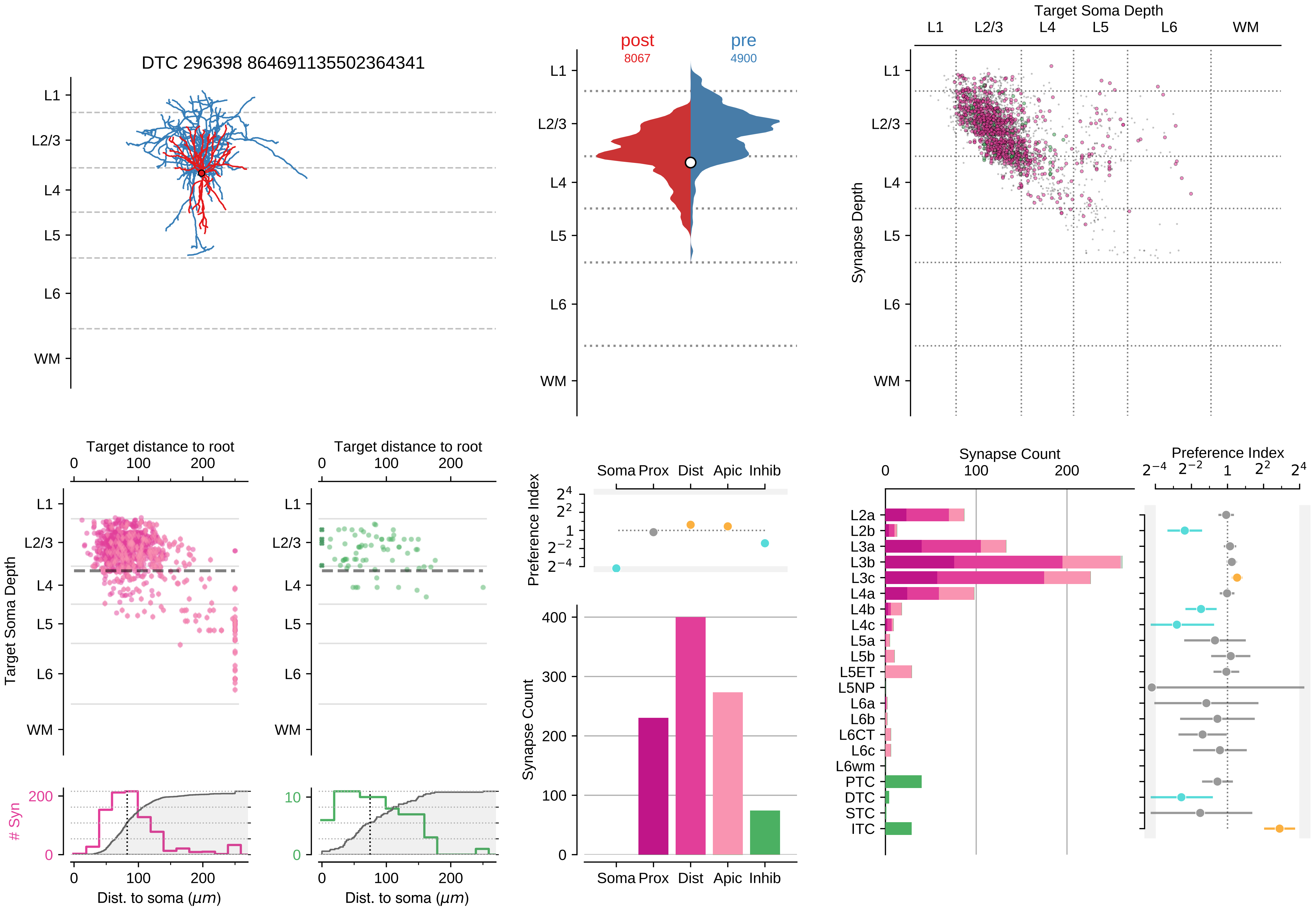

DTC 296398 864691135502364341
post
8067
pre
4900
Target Soma Depth
Synapse Depth
L1
L2/3
L4
L5
L6
WM
Target distance to root
Target Soma Depth
Dist. to soma (μm)
Syn
Preference Index
Soma
Prox
Dist
Apic
Inhib
Synapse Count
L2a
L2b
L3a
L3b
L3c
L4a
L4b
L4c
L5a
L5b
L5ET
L5NP
L6a
L6b
L6CT
L6c
L6wm
PTC
DTC
STC
ITC

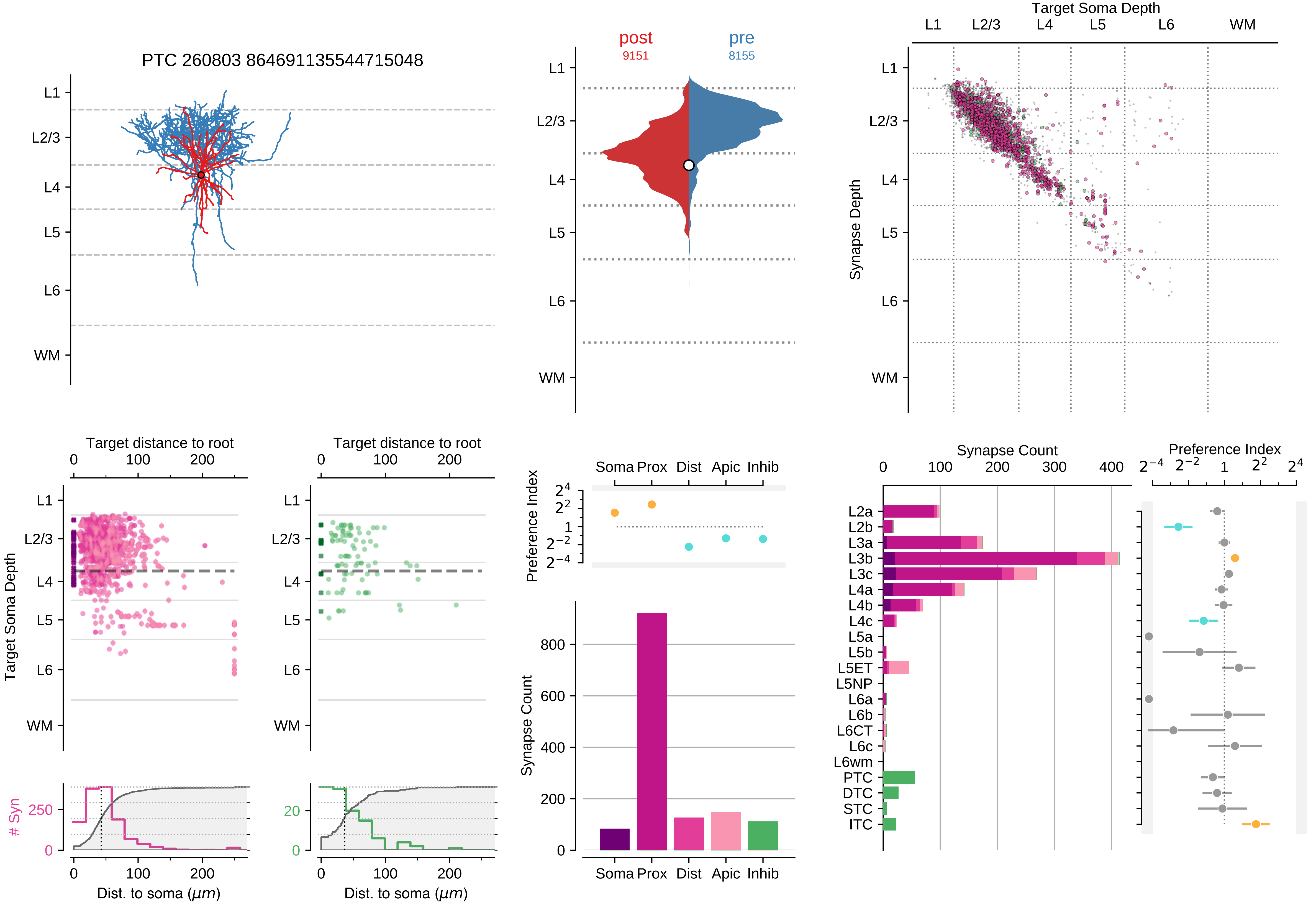

PTC 260803 864691135544715048
L1
L2/3
L4
L5
L6
WM
post
9151
pre
8155
Target Soma Depth
Synapse Depth
Target distance to root
Target Soma Depth
Syn
Dist. to soma (μm)
Preference Index
Soma Prox Dist Apic Inhib
Synapse Count
Synapse Count
Preference Index
L2a
L2b
L3a
L3b
L3c
L4a
L4b
L4c
L5a
L5b
L5ET
L5NP
L6a
L6b
L6CT
L6c
L6wm
PTC
DTC
STC
ITC

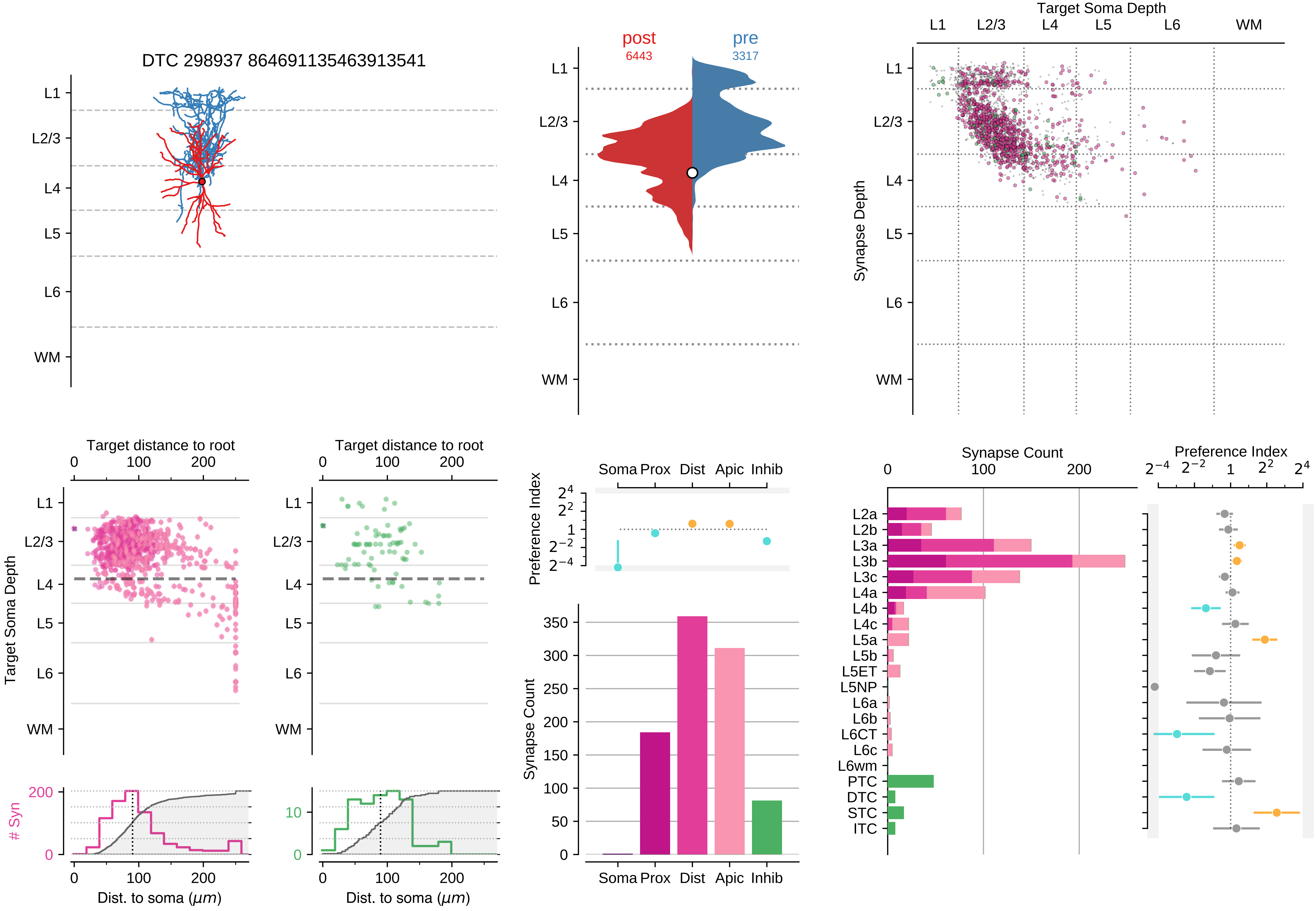

DTC 298937 864691135463913541
L1
L2/3
L4
L5
L6
WM
post
6443
pre
3317
Target Soma Depth
Synapse Depth
Target distance to root
Target Soma Depth
Syn
Dist. to soma (μm)
Preference Index
Soma
Prox
Dist
Apic
Inhib
Synapse Count
L2a
L2b
L3a
L3b
L3c
L4a
L4b
L4c
L5a
L5b
L5ET
L5NP
L6a
L6b
L6CT
L6c
L6wm
PTC
DTC
STC
ITC

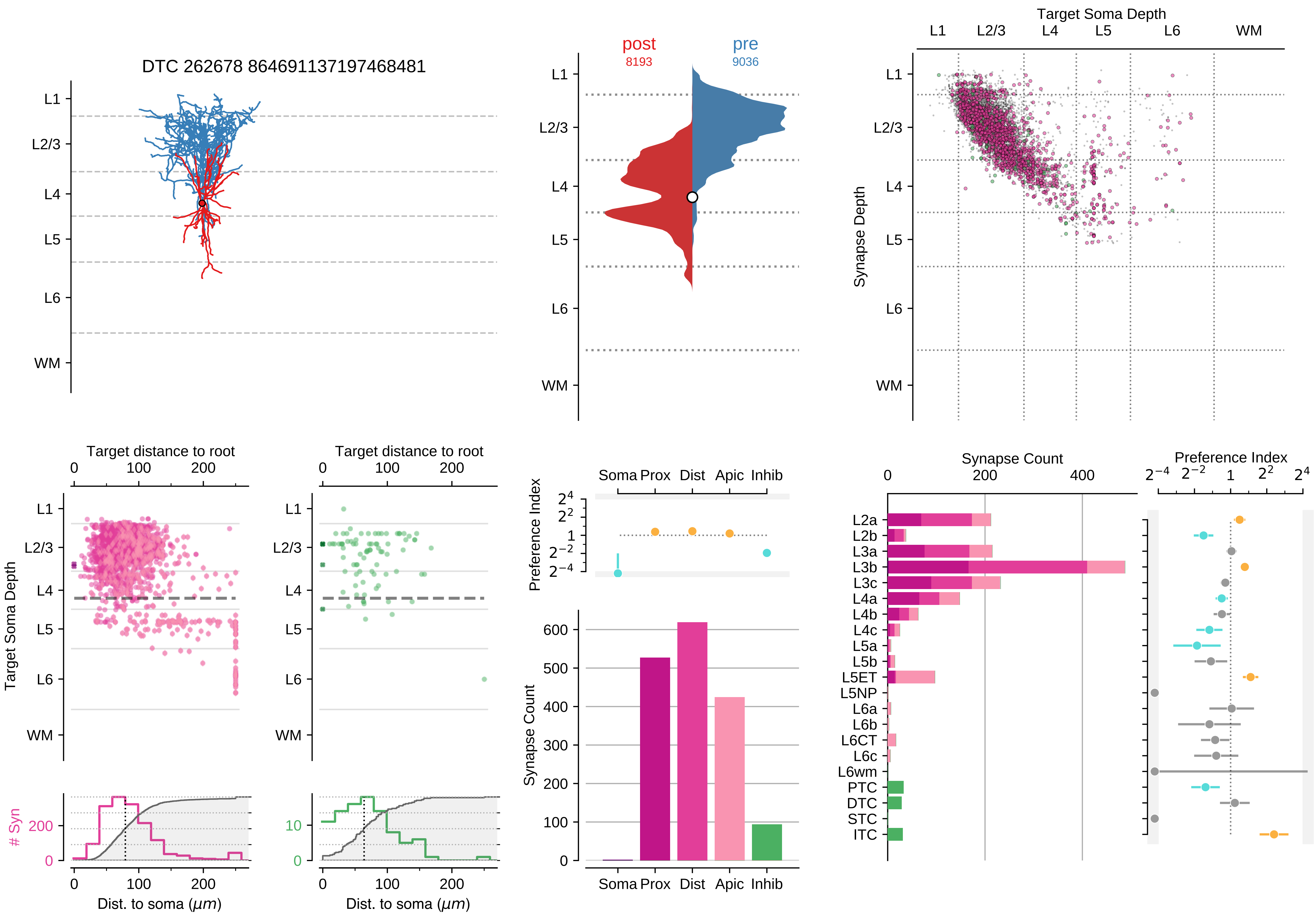
DTC 262678 864691137197468481
post
8193
pre
9036
Target Soma Depth
Synapse Depth
Target distance to root
Target Soma Depth
Dist. to soma (μm)
Syn
Preference Index
Soma Prox Dist Apic Inhib
Synapse Count
L2a
L2b
L3a
L3b
L3c
L4a
L4b
L4c
L5a
L5b
L5ET
L5NP
L6a
L6b
L6CT
L6c
L6wm
PTC
DTC
STC
ITC

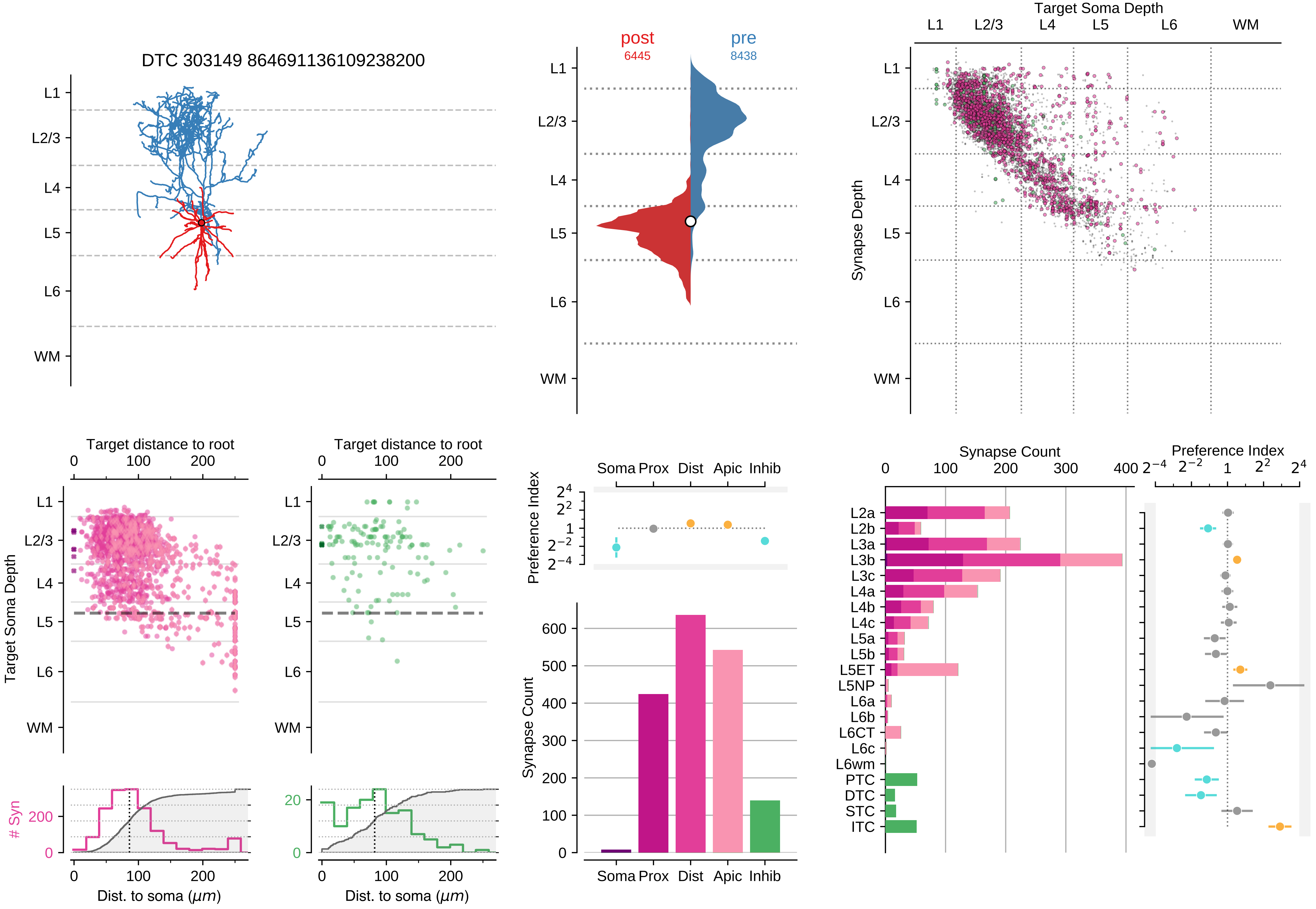
DTC 303149 864691136109238200
L1
L2/3
L4
L5
L6
WM
post
6445
pre
8438
Target Soma Depth
Synapse Depth
Target distance to root
Target Soma Depth
Syn
Dist. to soma (μm)
Preference Index
Soma Prox Dist Apic Inhib
Synapse Count
L2a
L2b
L3a
L3b
L3c
L4a
L4b
L4c
L5a
L5b
L5ET
L5NP
L6a
L6b
L6CT
L6c
L6wm
PTC
DTC
STC
ITC

Group 3

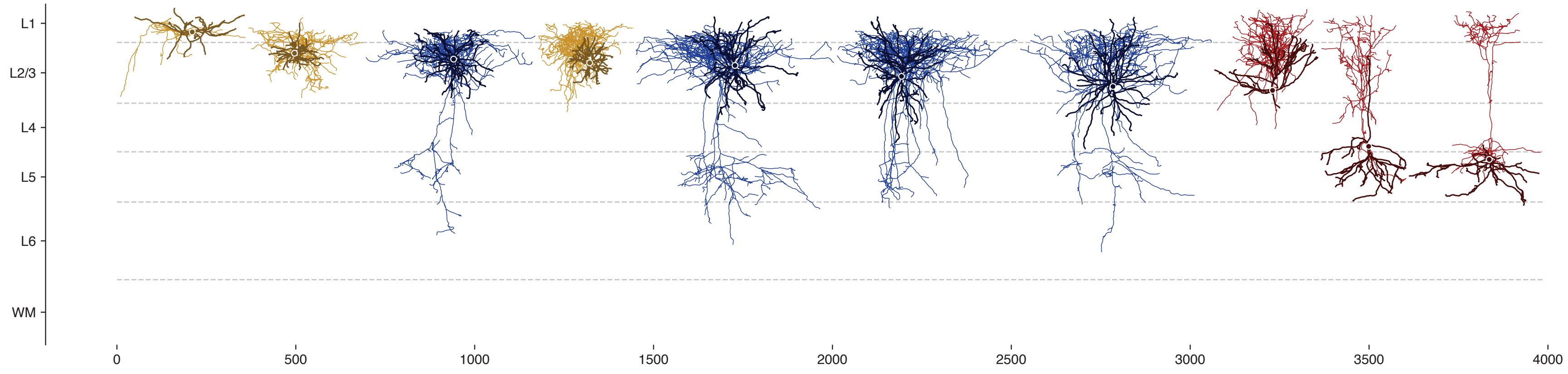
L1
L2/3
L4
L5
L6
WM
0
500
1000
1500
2000
2500
3000
3500
4000

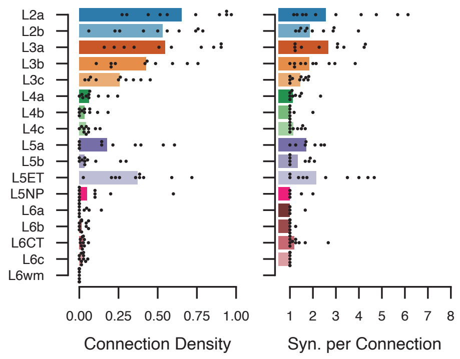
L2a
L2b
L3a
L3b
L3c
L4a
L4b
L4c
L5a
L5b
L5ET
L5NP
L6a
L6b
L6CT
L6c
L6wm
0.00
0.25
0.50
0.75
1.00
Connection Density
1
2
3
4
5
6
7
8
Syn. per Connection

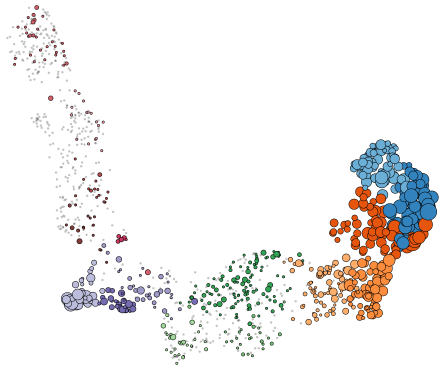

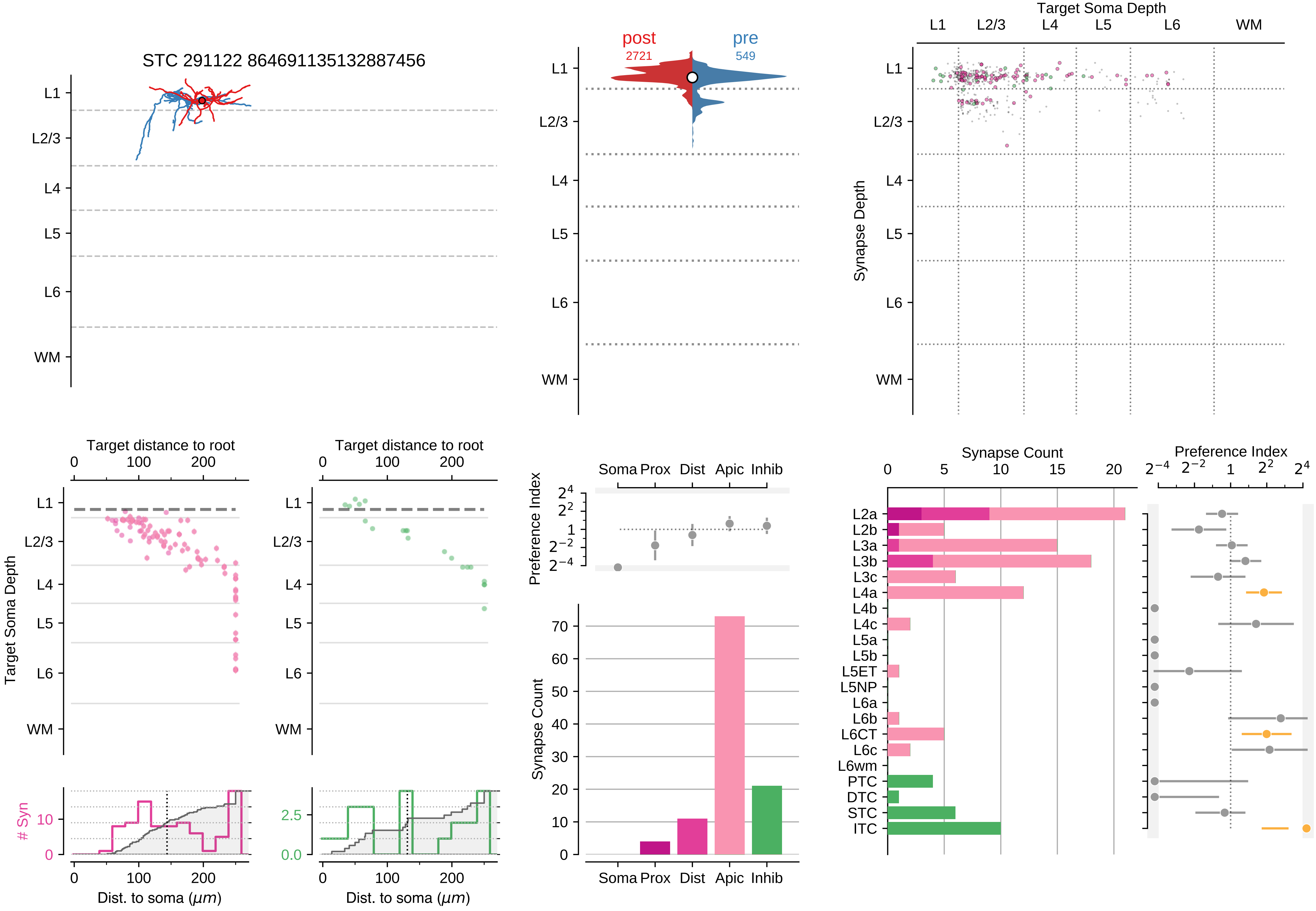

STC 291122 864691135132887456
L1
L2/3
L4
L5
L6
WM
post
2721
pre
549
Target Soma Depth
Synapse Depth
Target distance to root
Target Soma Depth
Syn
Dist. to soma (μm)
Preference Index
Soma Prox Dist Apic Inhib
Synapse Count
L2a
L2b
L3a
L3b
L3c
L4a
L4b
L4c
L5a
L5b
L5ET
L5NP
L6a
L6b
L6CT
L6c
L6wm
PTC
DTC
STC
ITC

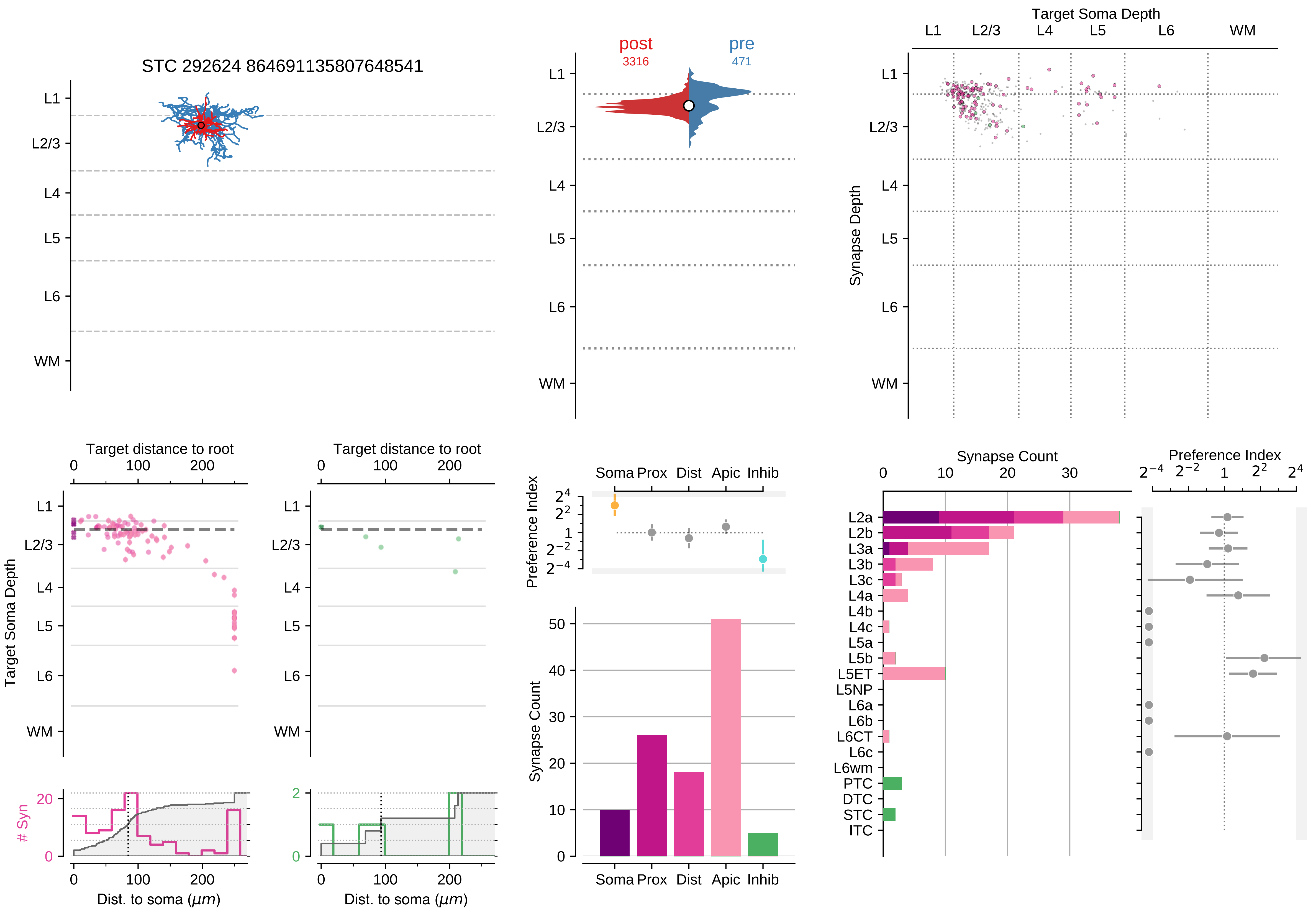
STC 292624 864691135807648541
post
3316
pre
471
Target Soma Depth
Synapse Depth
L1
L2/3
L4
L5
L6
WM
Target distance to root
Target Soma Depth
Syn
Dist. to soma (μm)
Preference Index
Synapse Count
Soma Prox Dist Apic Inhib
Synapse Count
Preference Index
L2a
L2b
L3a
L3b
L3c
L4a
L4b
L4c
L5a
L5b
L5ET
L5NP
L6a
L6b
L6CT
L6c
L6wm
PTC
DTC
STC
ITC

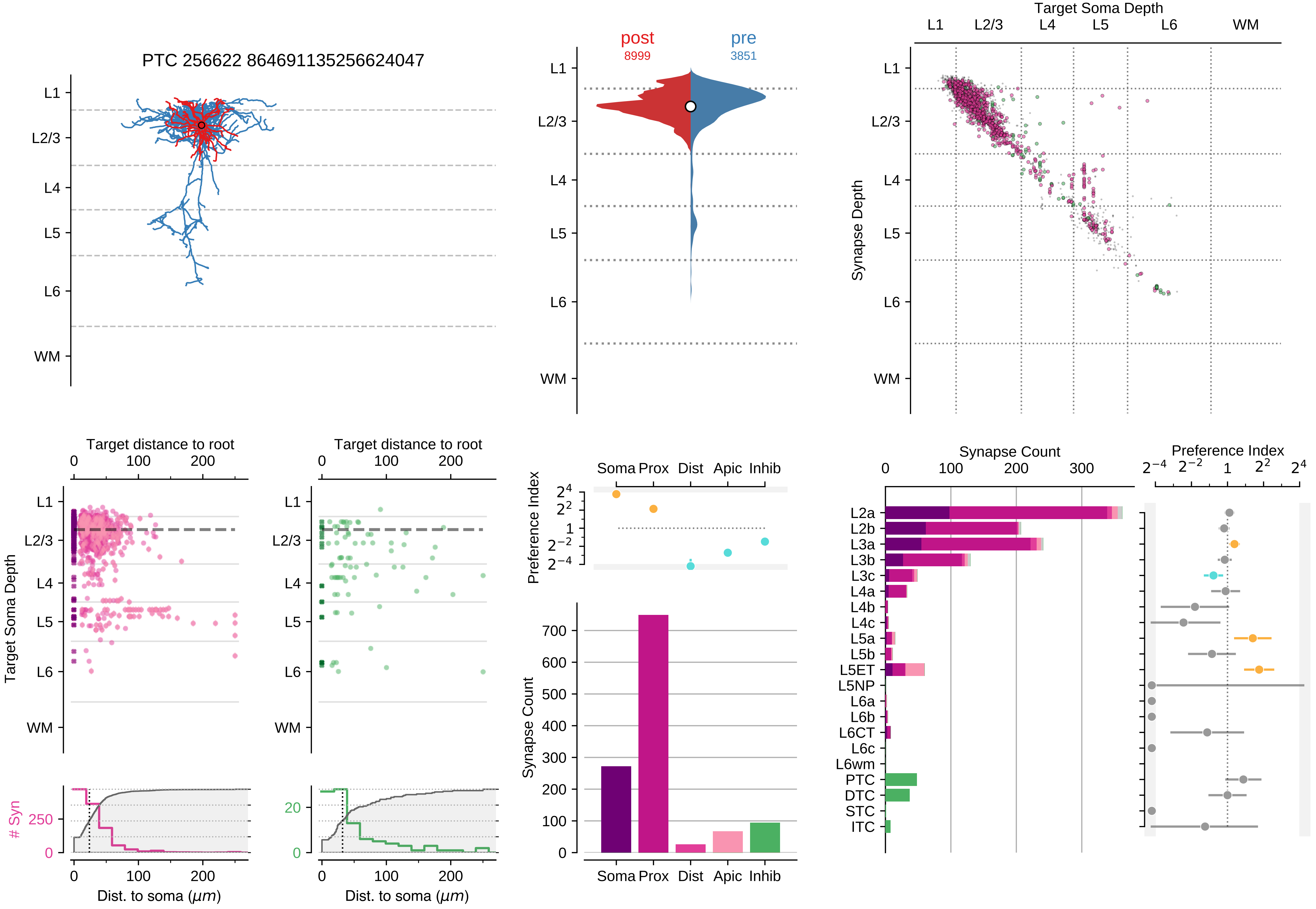
PTC 256622 864691135256624047
L1
L2/3
L4
L5
L6
WM
post
8999
pre
3851
Target Soma Depth
Synapse Depth
Target distance to root
Target Soma Depth
Syn
Dist. to soma (μm)
Preference Index
Soma
Prox
Dist
Apic
Inhib
Synapse Count
L2a
L2b
L3a
L3b
L3c
L4a
L4b
L4c
L5a
L5b
L5ET
L5NP
L6a
L6b
L6CT
L6c
L6wm
PTC
DTC
STC
ITC

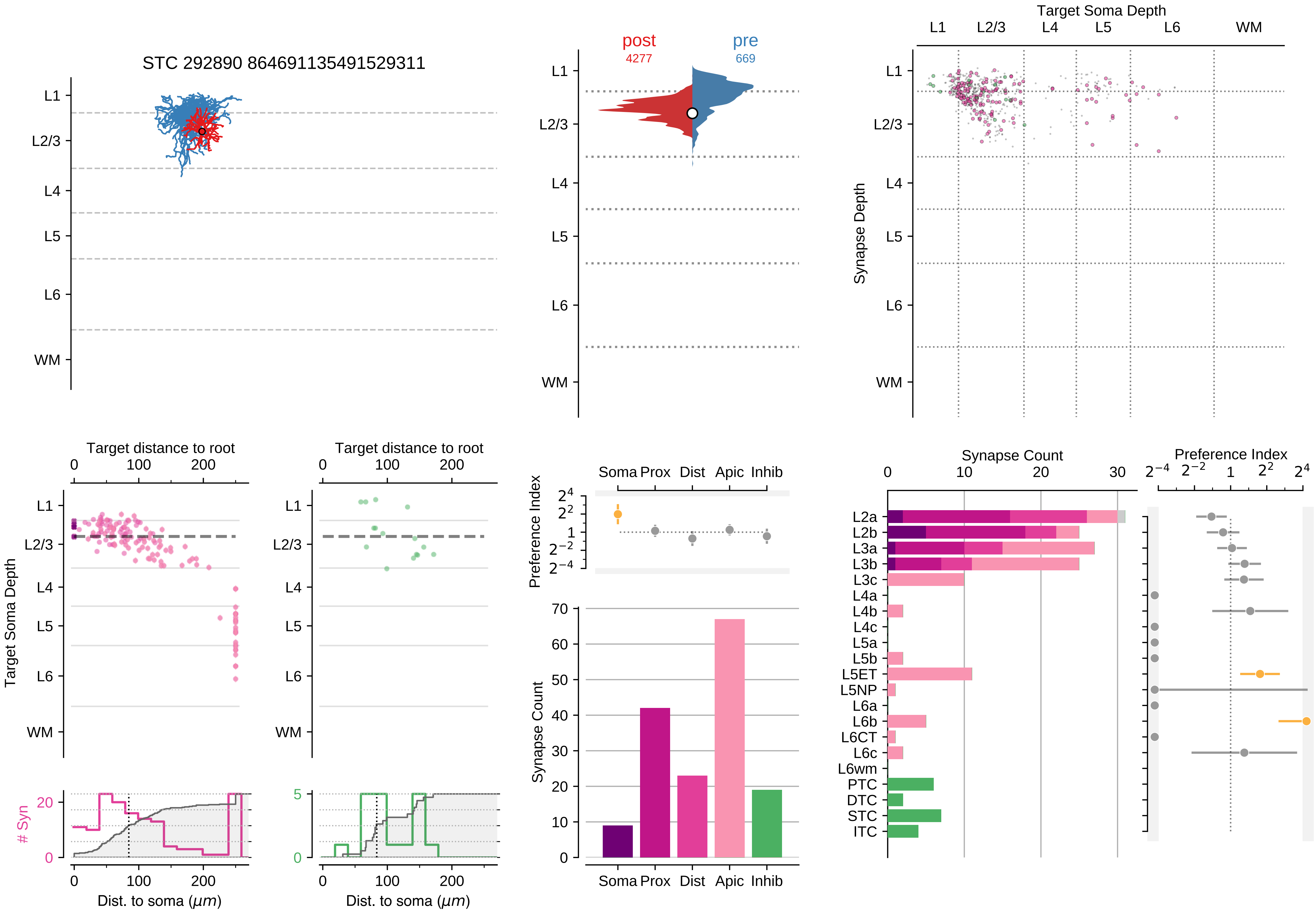

STC 292890 864691135491529311
post
4277
pre
669
Target Soma Depth
Synapse Depth
L1
L2/3
L4
L5
L6
WM
Target distance to root
Target Soma Depth
Syn
Dist. to soma (μm)
Preference Index
Soma Prox Dist Apic Inhib
Synapse Count
L2a
L2b
L3a
L3b
L3c
L4a
L4b
L4c
L5a
L5b
L5ET
L5NP
L6a
L6b
L6CT
L6c
L6wm
PTC
DTC
STC
ITC

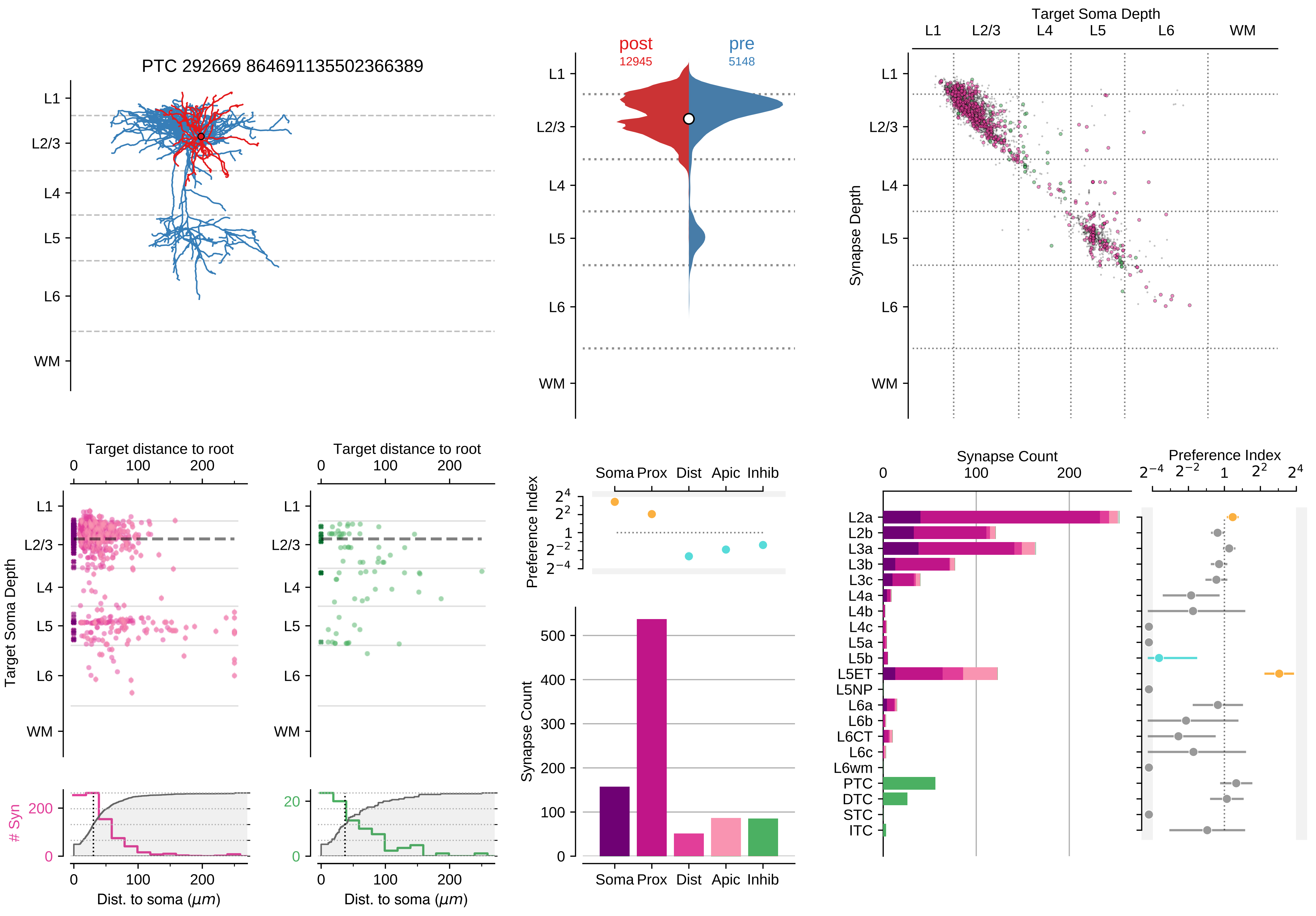

PTC 292669 864691135502366389
L1
L2/3
L4
L5
L6
WM
post
12945
pre
5148
Target Soma Depth
Synapse Depth
Target distance to root
Target Soma Depth
Syn
Dist. to soma (μm)
Preference Index
Soma Prox Dist Apic Inhib
Synapse Count
Synapse Count
Preference Index
L2a
L2b
L3a
L3b
L3c
L4a
L4b
L4c
L5a
L5b
L5ET
L5NP
L6a
L6b
L6CT
L6c
L6wm
PTC
DTC
STC
ITC

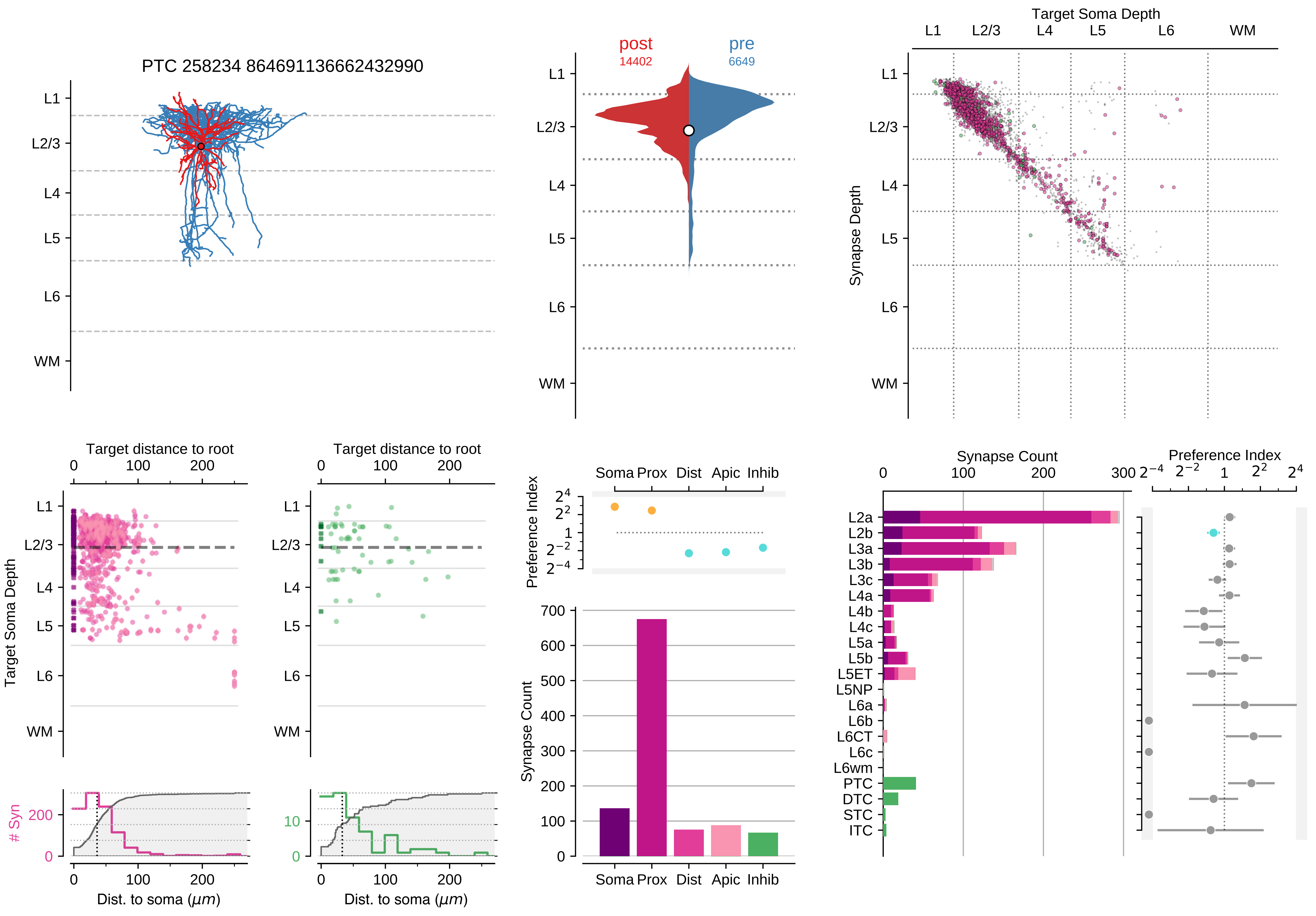
PTC 258234 864691136662432990
L1
L2/3
L4
L5
L6
WM
post
14402
pre
6649
Target Soma Depth
Synapse Depth
Target distance to root
Target Soma Depth
Syn
Dist. to soma (μm)
Preference Index
Soma
Prox
Dist
Apic
Inhib
Synapse Count
L2a
L2b
L3a
L3b
L3c
L4a
L4b
L4c
L5a
L5b
L5ET
L5NP
L6a
L6b
L6CT
L6c
L6wm
PTC
DTC
STC
ITC

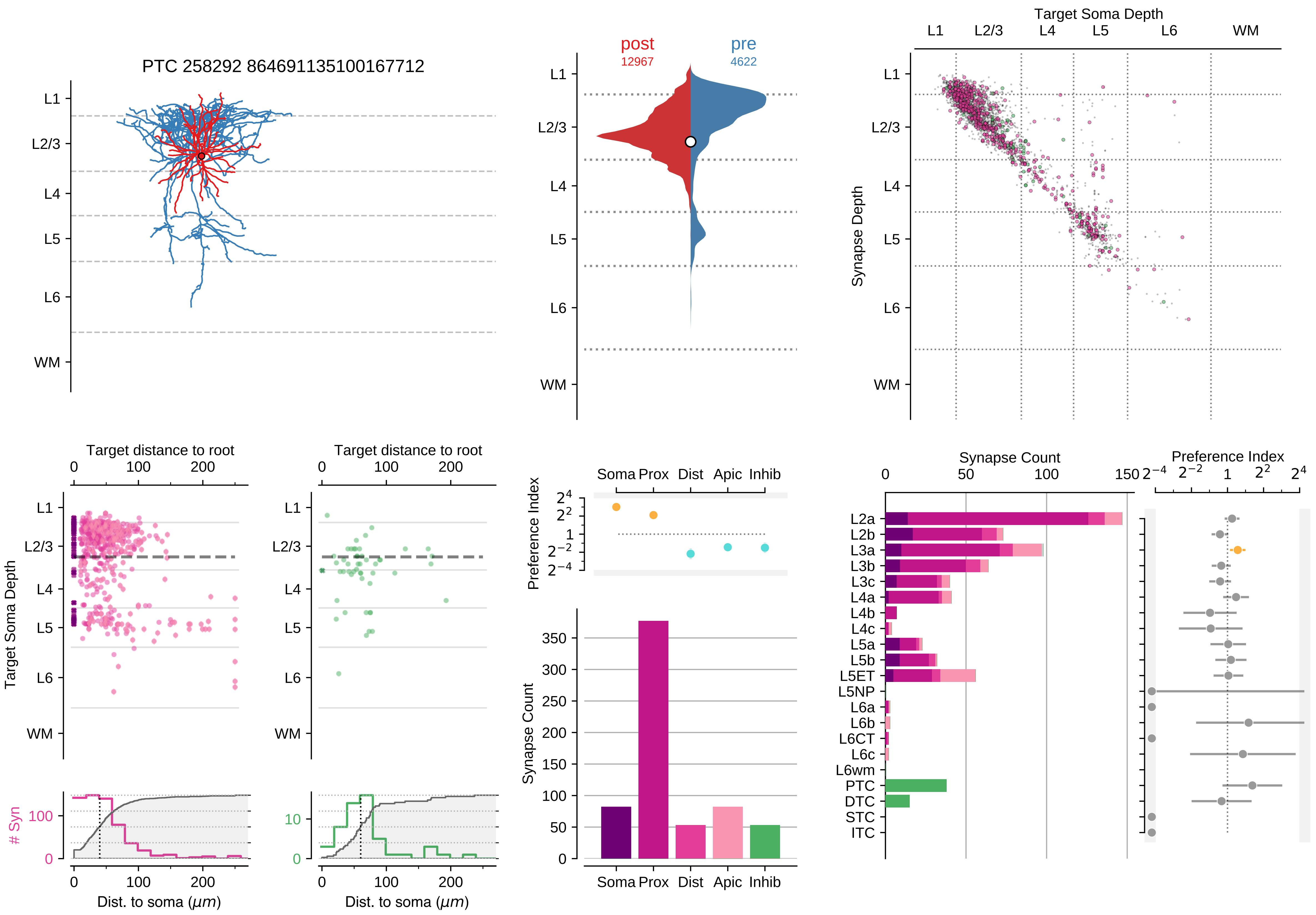
PTC 258292 864691135100167712
L1
L2/3
L4
L5
L6
WM
post
12967
pre
4622
Target Soma Depth
Synapse Depth
Target distance to root
Target Soma Depth
Syn
Dist. to soma (μm)
Preference Index
Soma
Prox
Dist
Apic
Inhib
Synapse Count
L2a
L2b
L3a
L3b
L3c
L4a
L4b
L4c
L5a
L5b
L5ET
L5NP
L6a
L6b
L6CT
L6c
L6wm
PTC
DTC
STC
ITC

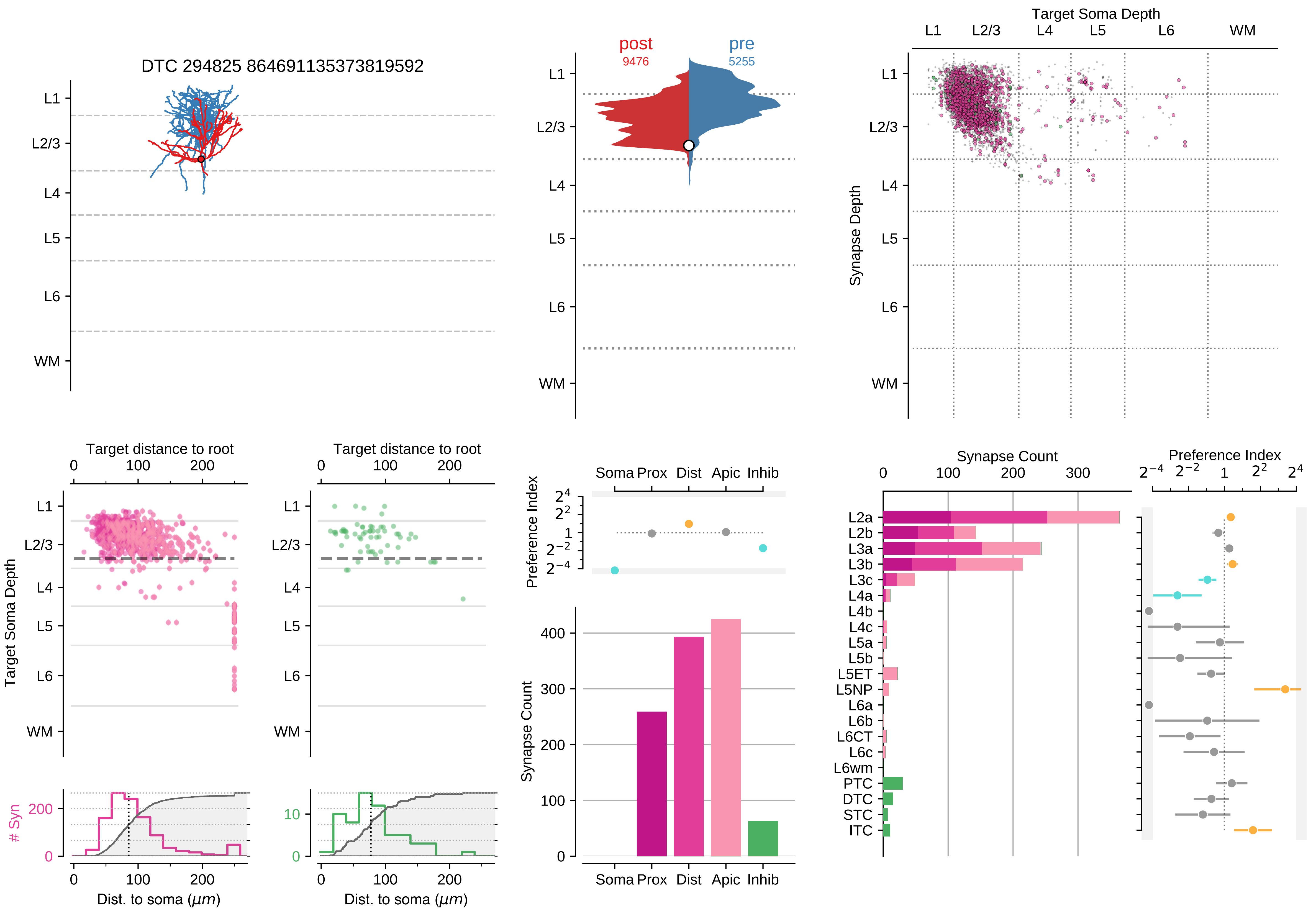
DTC 294825 864691135373819592
L1
L2/3
L4
L5
L6
WM
post
9476
pre
5255
Target Soma Depth
Synapse Depth
Target distance to root
Target Soma Depth
Syn
Dist. to soma (μm)
Preference Index
Soma Prox Dist Apic Inhib
Synapse Count
L2a
L2b
L3a
L3b
L3c
L4a
L4b
L4c
L5a
L5b
L5ET
L5NP
L6a
L6b
L6CT
L6c
L6wm
PTC
DTC
STC
ITC

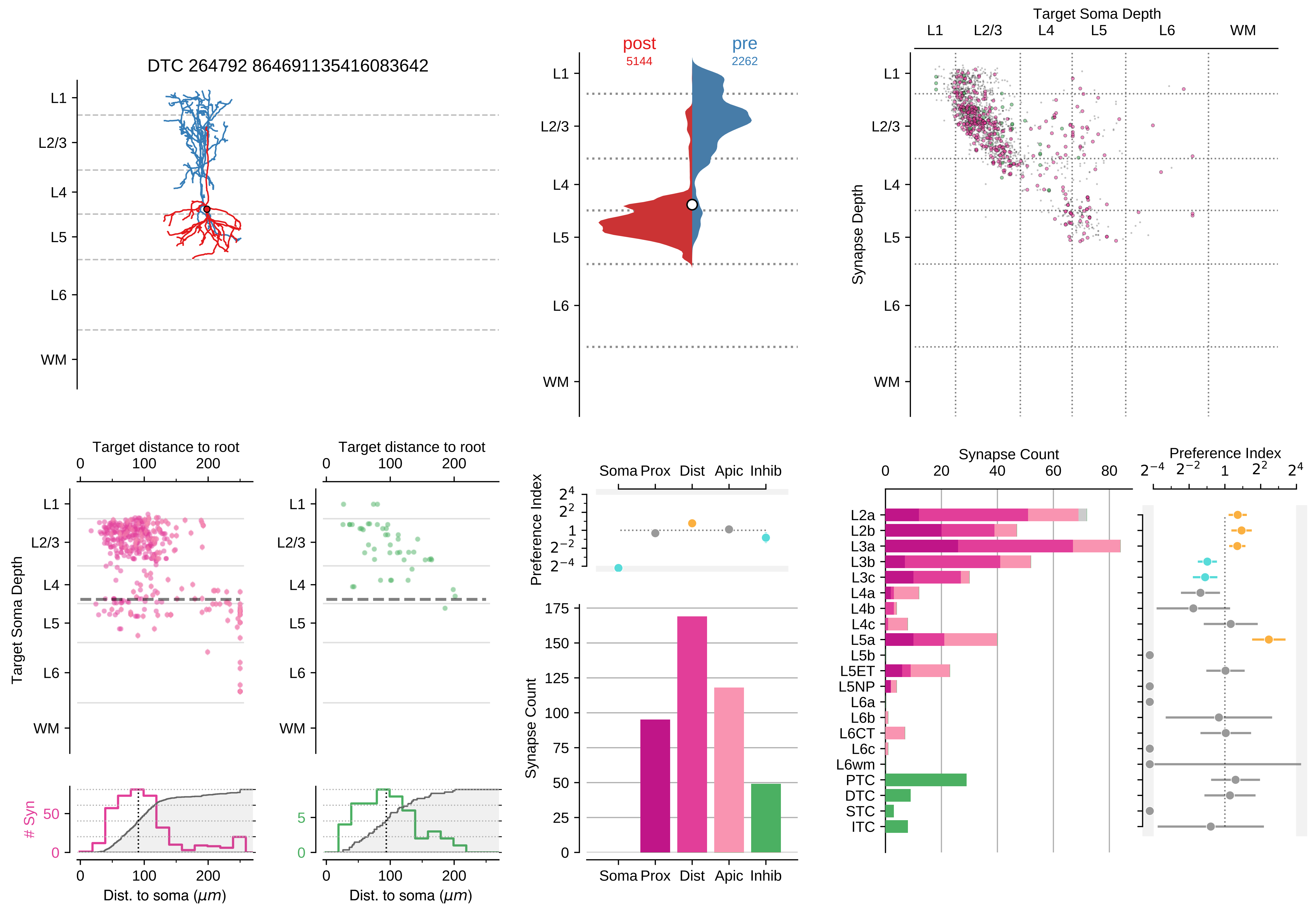

DTC 264792 864691135416083642
L1
L2/3
L4
L5
L6
WM
post
5144
pre
2262
Target Soma Depth
Synapse Depth
Target distance to root
Target Soma Depth
Dist. to soma (μm)
Syn
Preference Index
Soma Prox Dist Apic Inhib
Synapse Count
L2a
L2b
L3a
L3b
L3c
L4a
L4b
L4c
L5a
L5b
L5ET
L5NP
L6a
L6b
L6CT
L6c
L6wm
PTC
DTC
STC
ITC

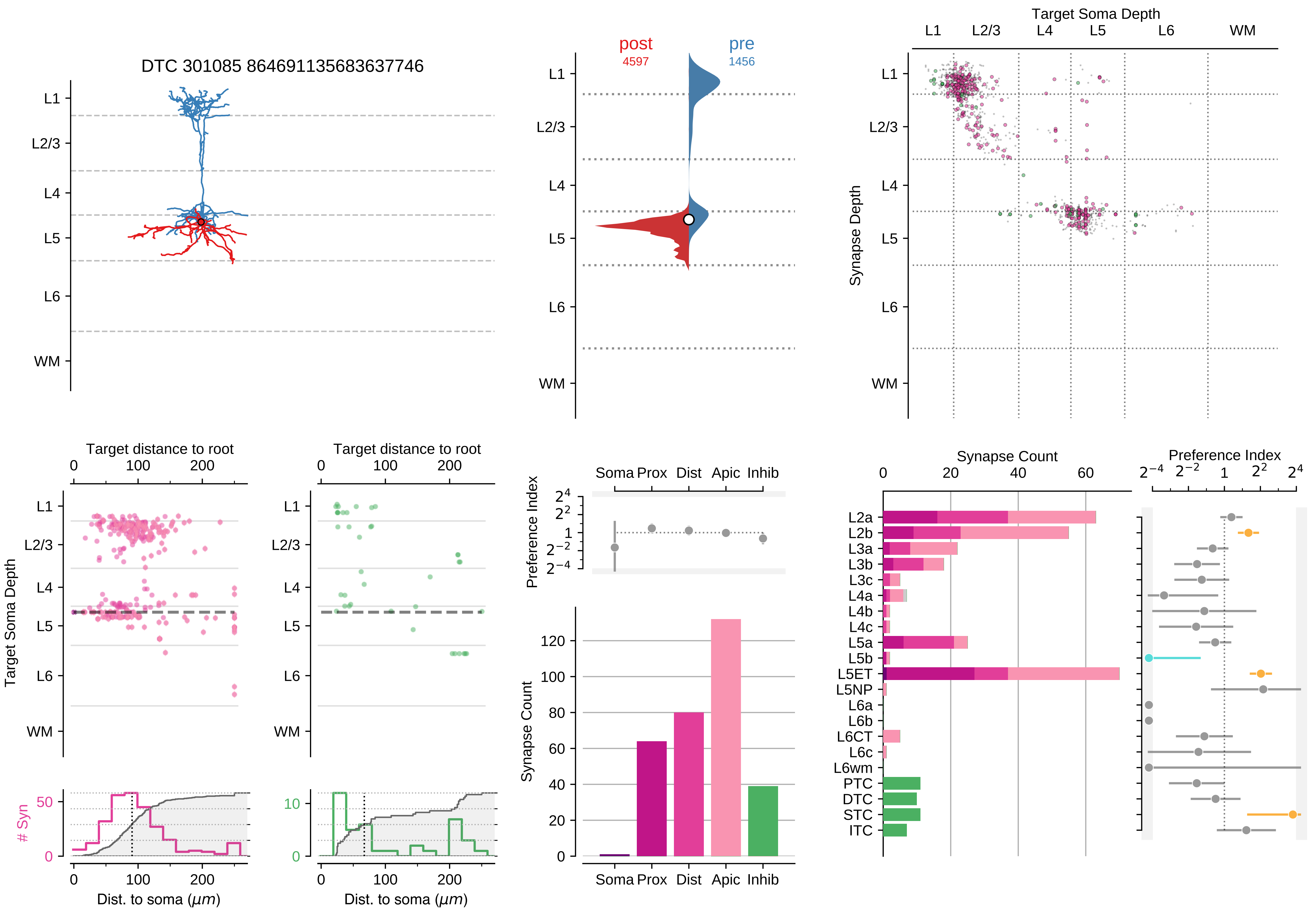

DTC 301085 864691135683637746
L1
L2/3
L4
L5
L6
WM
post
4597
pre
1456
Target Soma Depth
Synapse Depth
Target distance to root
Target Soma Depth
Syn
Dist. to soma (μm)
Preference Index
Soma Prox Dist Apic Inhib
Synapse Count
Preference Index
L2a
L2b
L3a
L3b
L3c
L4a
L4b
L4c
L5a
L5b
L5ET
L5NP
L6a
L6b
L6CT
L6c
L6wm
PTC
DTC
STC
ITC

Group 4

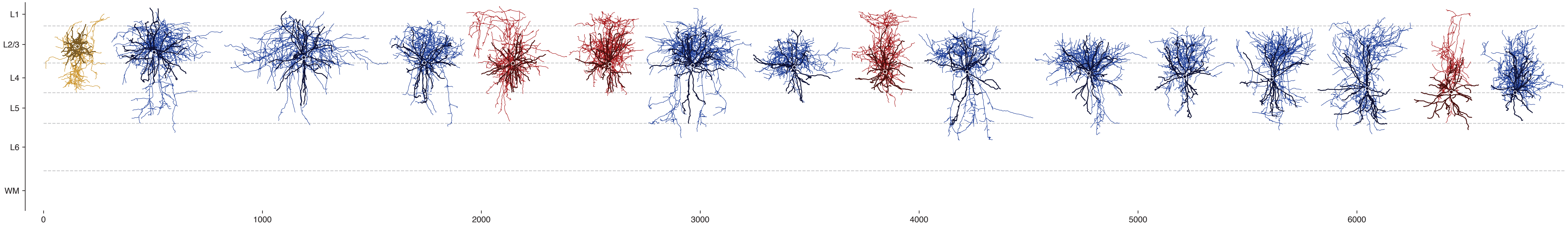
L1
L2/3
L4
L5
L6
WM
0
1000
2000
3000
4000
5000
6000

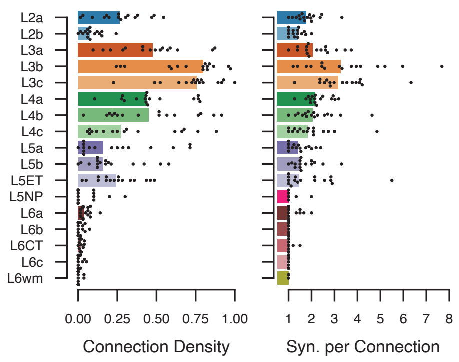
L2a
L2b
L3a
L3b
L3c
L4a
L4b
L4c
L5a
L5b
L5ET
L5NP
L6a
L6b
L6CT
L6c
L6wm
0.00 0.25 0.50 0.75 1.00
Connection Density
1 2 3 4 5 6 7 8
Syn. per Connection

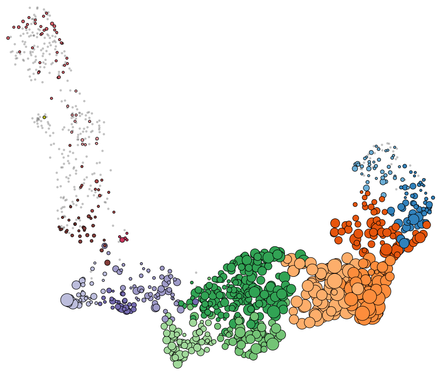

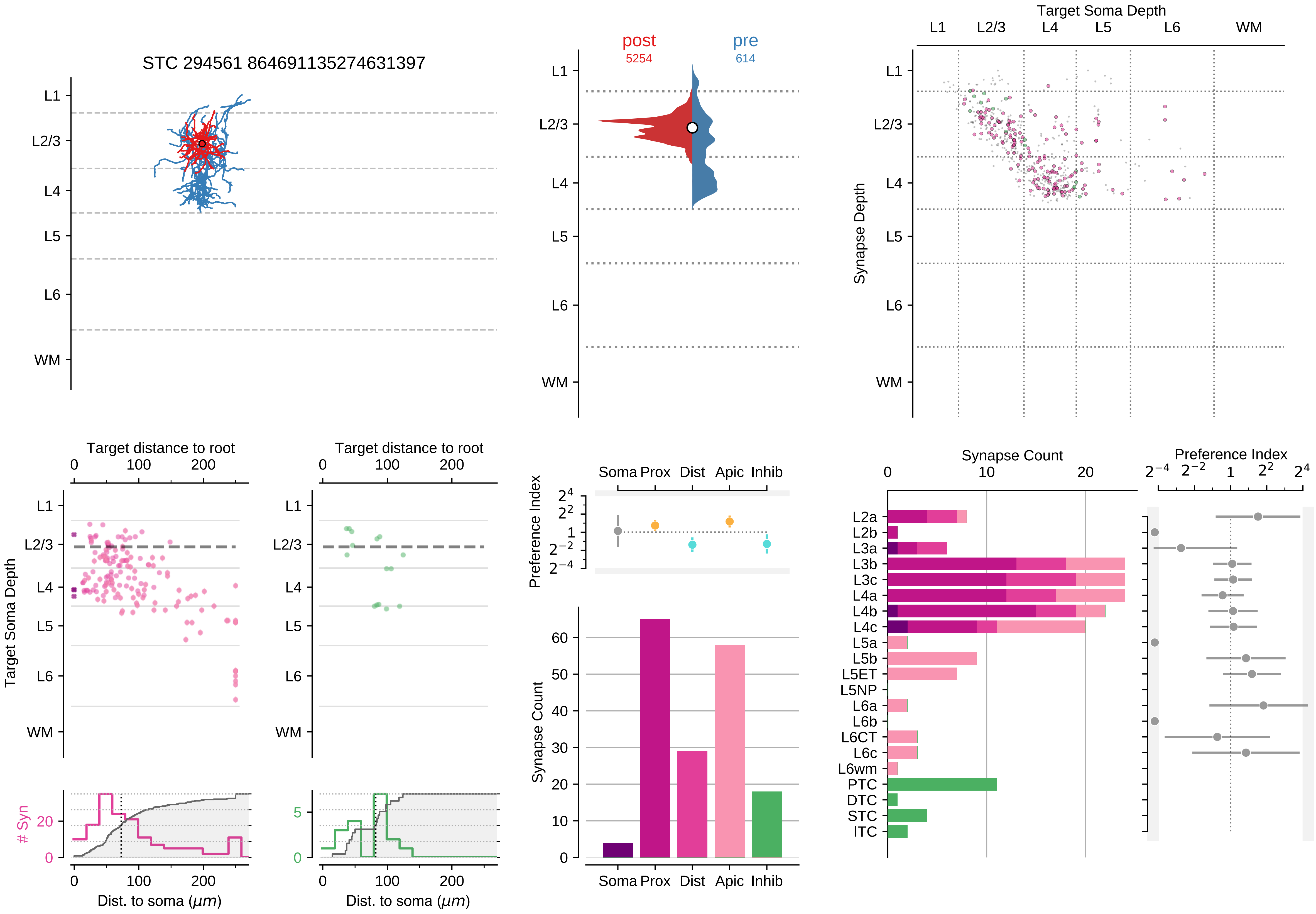

STC 294561 864691135274631397
L1
L2/3
L4
L5
L6
WM
post
5254
pre
614
Target Soma Depth
Synapse Depth
Target distance to root
Target Soma Depth
Syn
Dist. to soma (μm)
Soma
Prox
Dist
Apic
Inhib
Preference Index
Synapse Count
L2a
L2b
L3a
L3b
L3c
L4a
L4b
L4c
L5a
L5b
L5ET
L5NP
L6a
L6b
L6CT
L6c
L6wm
PTC
DTC
STC
ITC

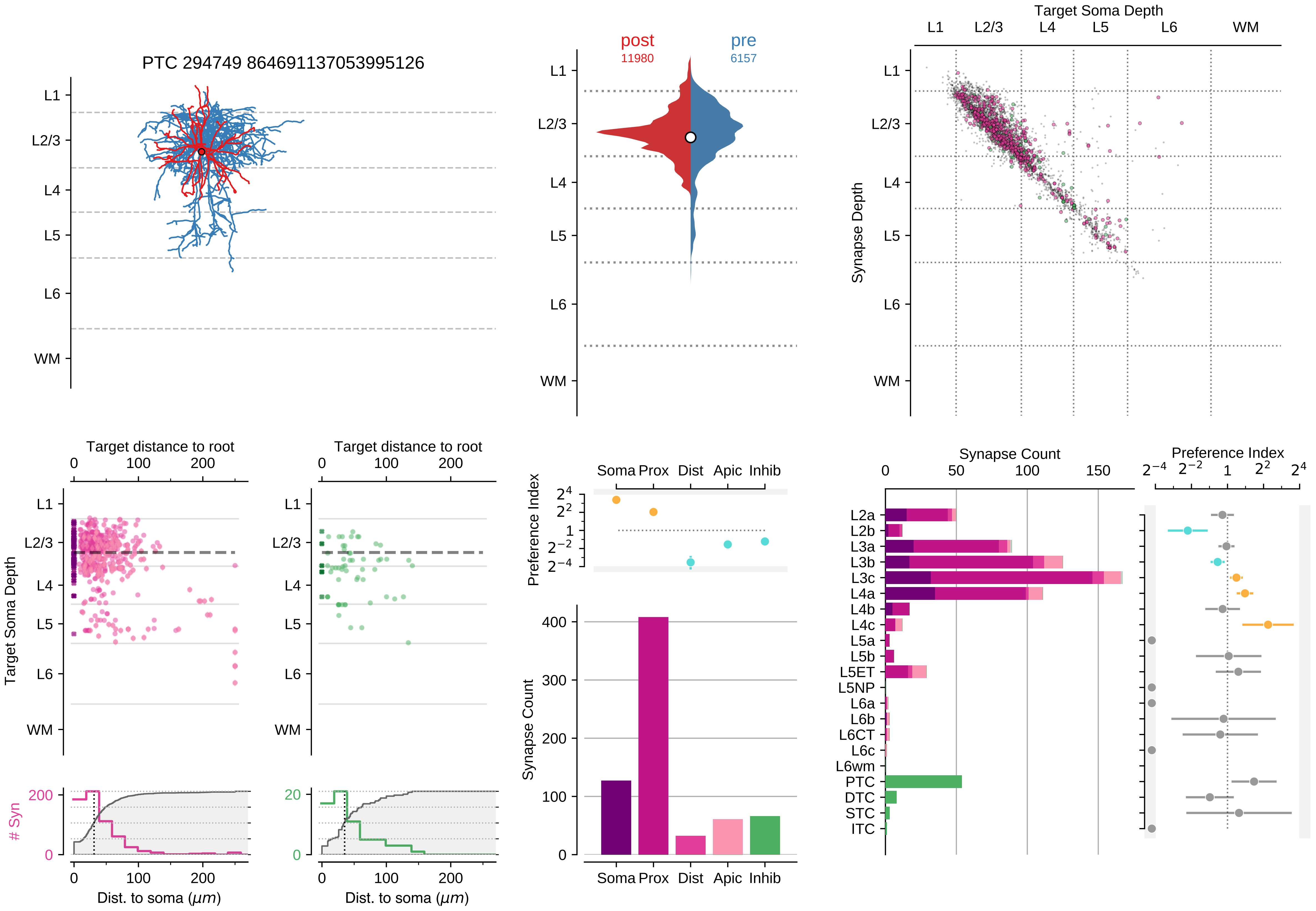
PTC 294749 864691137053995126
L1
L2/3
L4
L5
L6
WM
post
11980
pre
6157
Target Soma Depth
Synapse Depth
Target distance to root
Target Soma Depth
Syn
Dist. to soma (μm)
Preference Index
Soma Prox Dist Apic Inhib
Synapse Count
L2a
L2b
L3a
L3b
L3c
L4a
L4b
L4c
L5a
L5b
L5ET
L5NP
L6a
L6b
L6CT
L6c
L6wm
PTC
DTC
STC
ITC

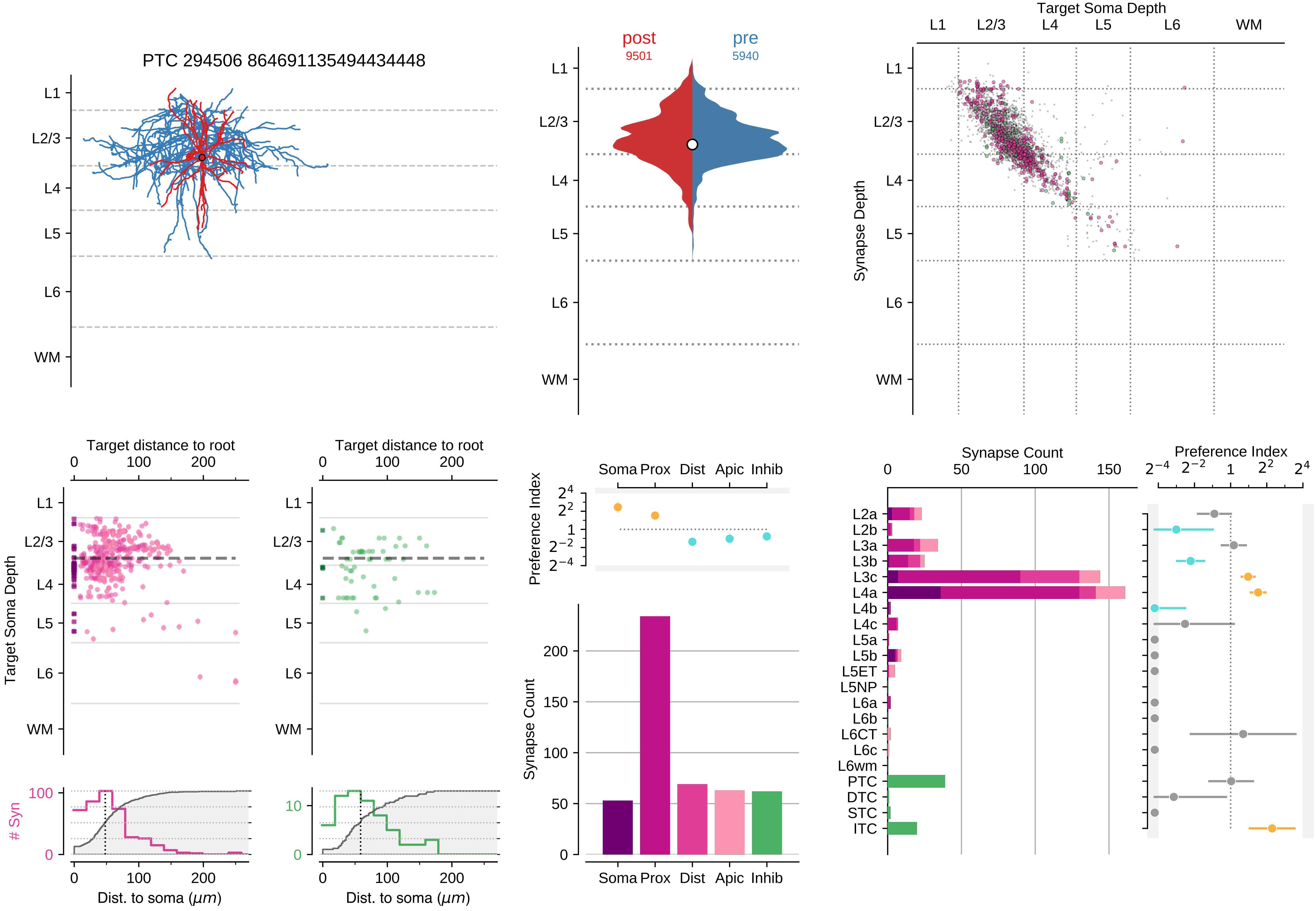

PTC 294506 864691135494434448
L1
L2/3
L4
L5
L6
WM
post
9501
pre
5940
Target Soma Depth
Synapse Depth
Target distance to root
Target Soma Depth
Syn
Dist. to soma (μm)
Preference Index
Soma
Prox
Dist
Apic
Inhib
Synapse Count
Synapse Count
Preference Index
L2a
L2b
L3a
L3b
L3c
L4a
L4b
L4c
L5a
L5b
L5ET
L5NP
L6a
L6b
L6CT
L6c
L6wm
PTC
DTC
STC
ITC

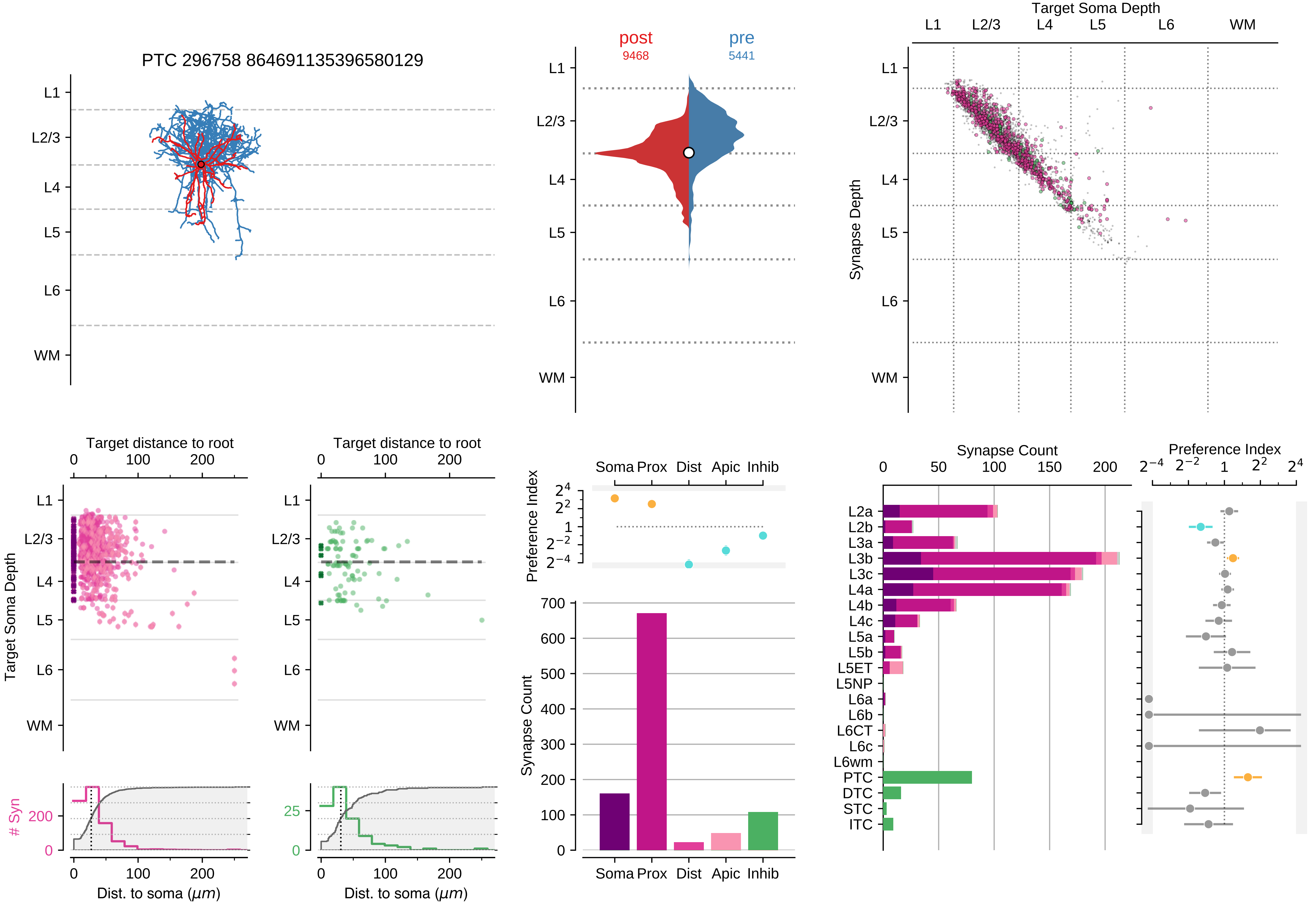
PTC 296758 864691135396580129
L1
L2/3
L4
L5
L6
WM
post
9468
pre
5441
Target Soma Depth
Synapse Depth
Target distance to root
Target Soma Depth
Syn
Dist. to soma (μm)
Preference Index
Soma Prox Dist Apic Inhib
Synapse Count
L2a
L2b
L3a
L3b
L3c
L4a
L4b
L4c
L5a
L5b
L5ET
L5NP
L6a
L6b
L6CT
L6c
L6wm
PTC
DTC
STC
ITC

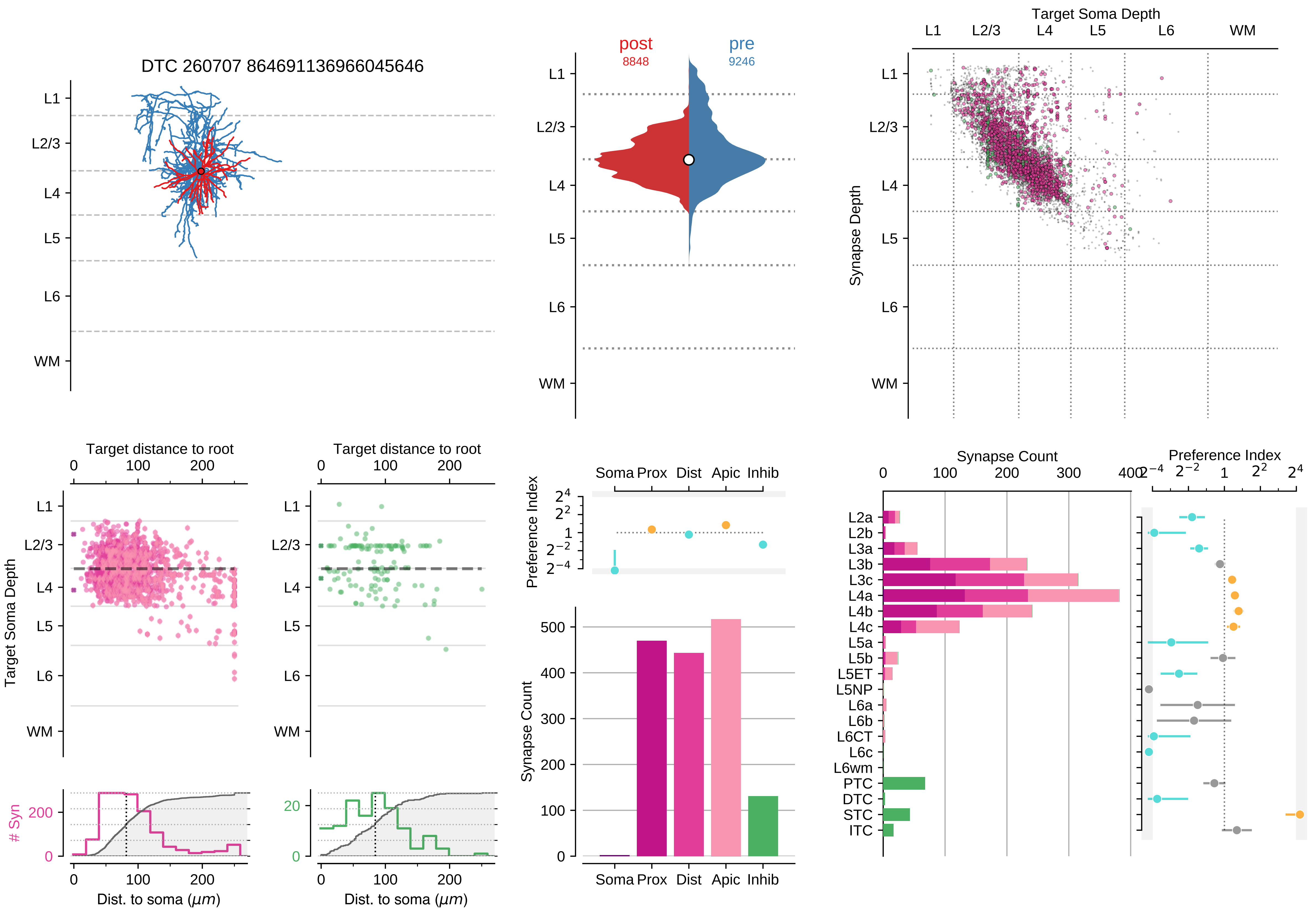

DTC 260707 864691136966045646
L1
L2/3
L4
L5
L6
WM
post
8848
pre
9246
Target Soma Depth
Synapse Depth
Target distance to root
Target Soma Depth
Syn
Dist. to soma (μm)
Preference Index
Soma Prox Dist Apic Inhib
Synapse Count
L2a
L2b
L3a
L3b
L3c
L4a
L4b
L4c
L5a
L5b
L5ET
L5NP
L6a
L6b
L6CT
L6c
L6wm
PTC
DTC
STC
ITC

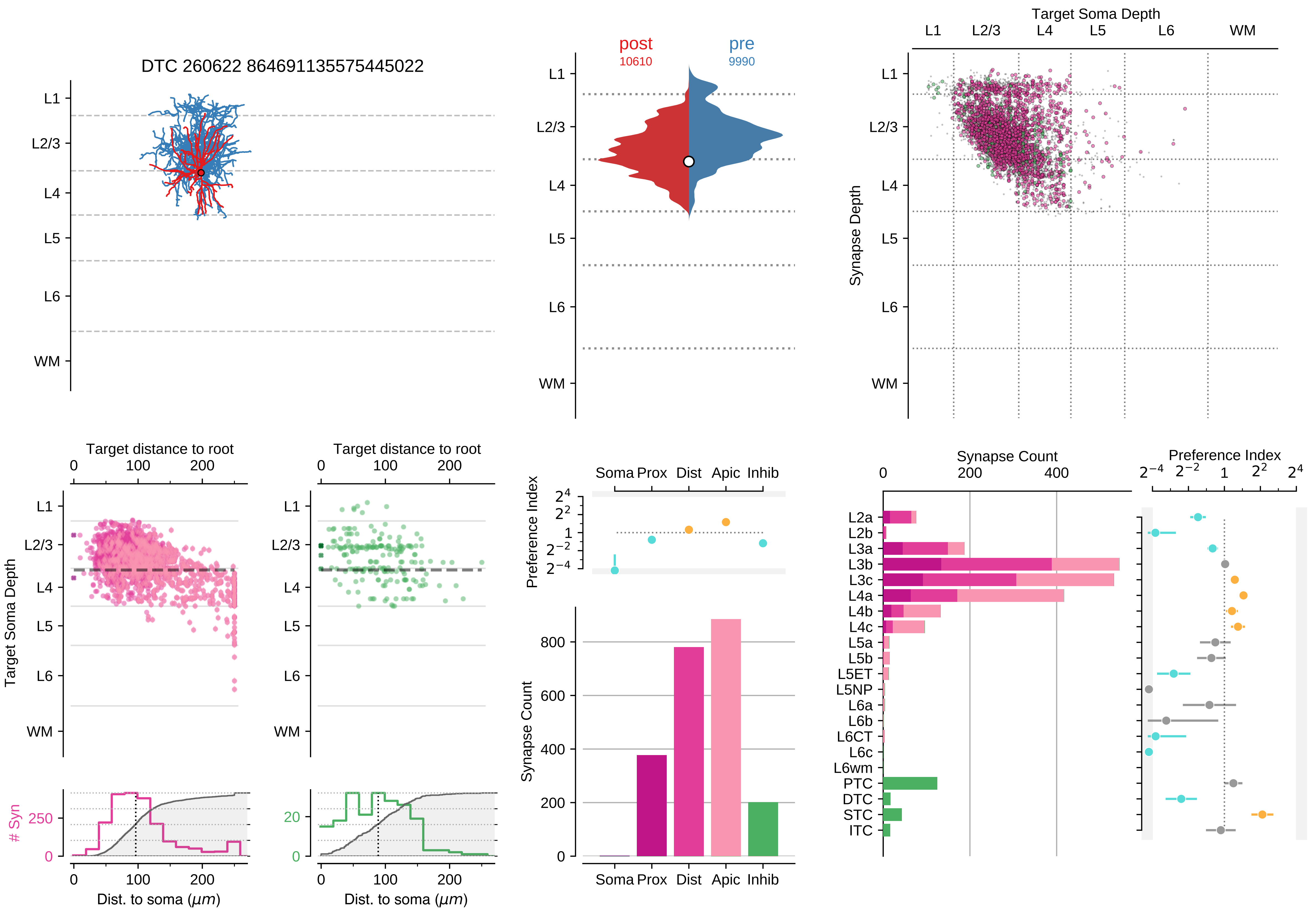

DTC 260622 864691135575445022
post
10610
pre
9990
Target Soma Depth
Synapse Depth
L1
L2/3
L4
L5
L6
WM
Target distance to root
Target Soma Depth
Syn
Dist. to soma (μm)
Preference Index
Soma Prox Dist Apic Inhib
Synapse Count
L2a
L2b
L3a
L3b
L3c
L4a
L4b
L4c
L5a
L5b
L5ET
L5NP
L6a
L6b
L6CT
L6c
L6wm
PTC
DTC
STC
ITC

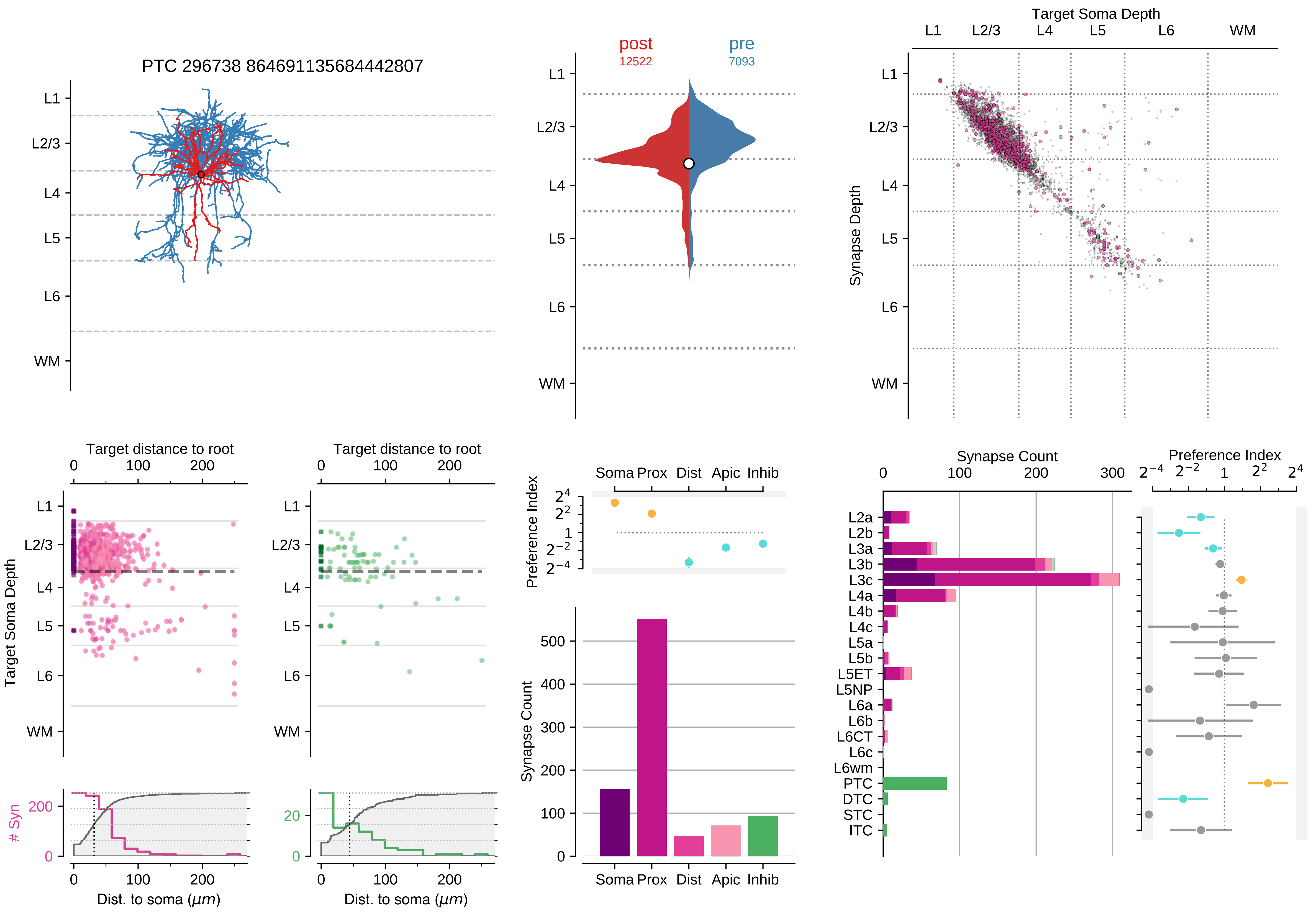

PTC 296738 864691135684442807
L1
L2/3
L4
L5
L6
WM
post
12522
pre
7093
Target Soma Depth
Synapse Depth
Target distance to root
Target Soma Depth
Syn
Dist. to soma (μm)
Preference Index
Soma
Prox
Dist
Apic
Inhib
Synapse Count
L2a
L2b
L3a
L3b
L3c
L4a
L4b
L4c
L5a
L5b
L5ET
L5NP
L6a
L6b
L6CT
L6c
L6wm
PTC
DTC
STC
ITC

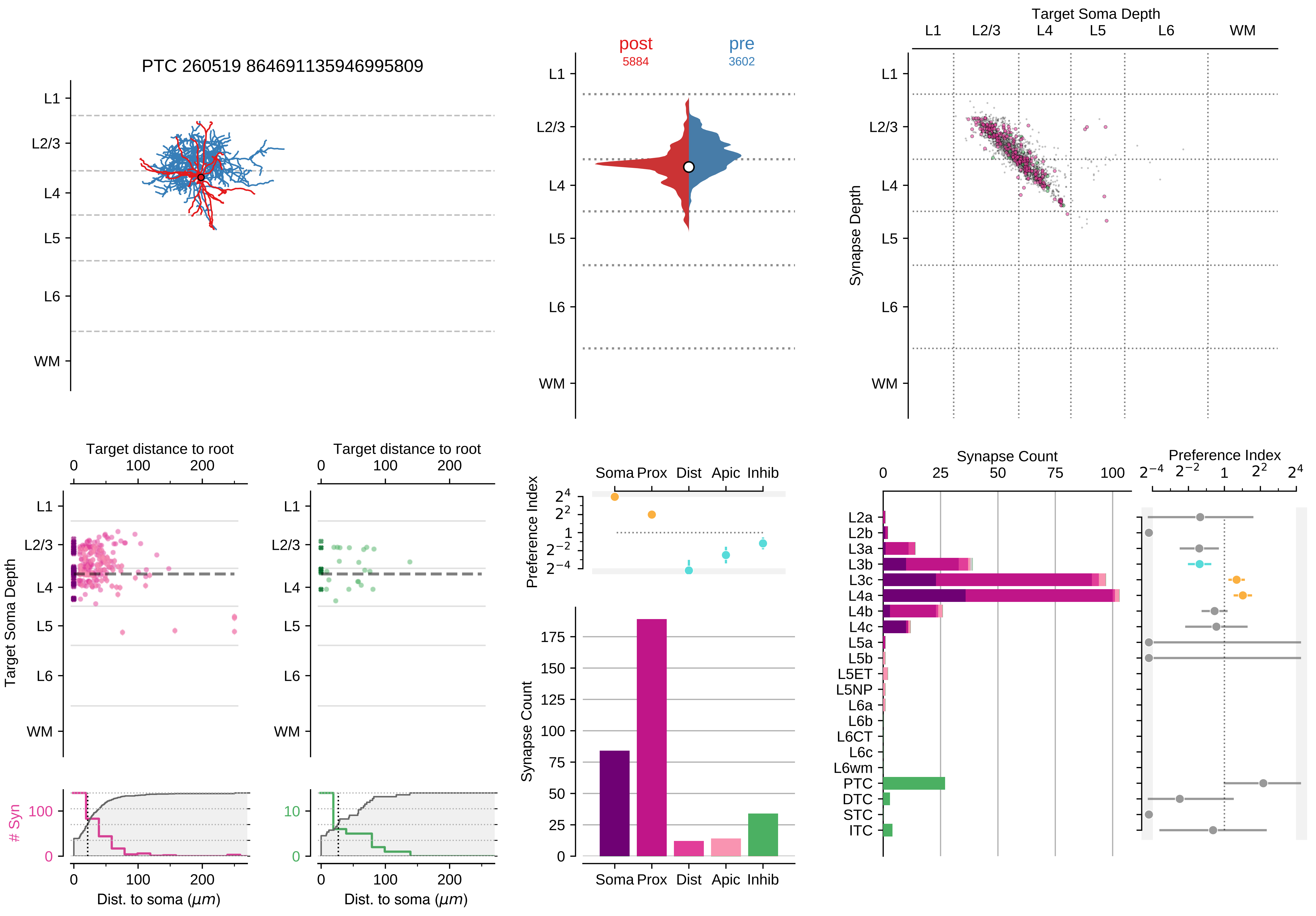
PTC 260519 864691135946995809
L1
L2/3
L4
L5
L6
WM
post
5884
pre
3602
Target Soma Depth
Synapse Depth
Target distance to root
Target Soma Depth
Dist. to soma (μm)
Syn
Preference Index
Soma Prox Dist Apic Inhib
Synapse Count
L2a
L2b
L3a
L3b
L3c
L4a
L4b
L4c
L5a
L5b
L5ET
L5NP
L6a
L6b
L6CT
L6c
L6wm
PTC
DTC
STC
ITC

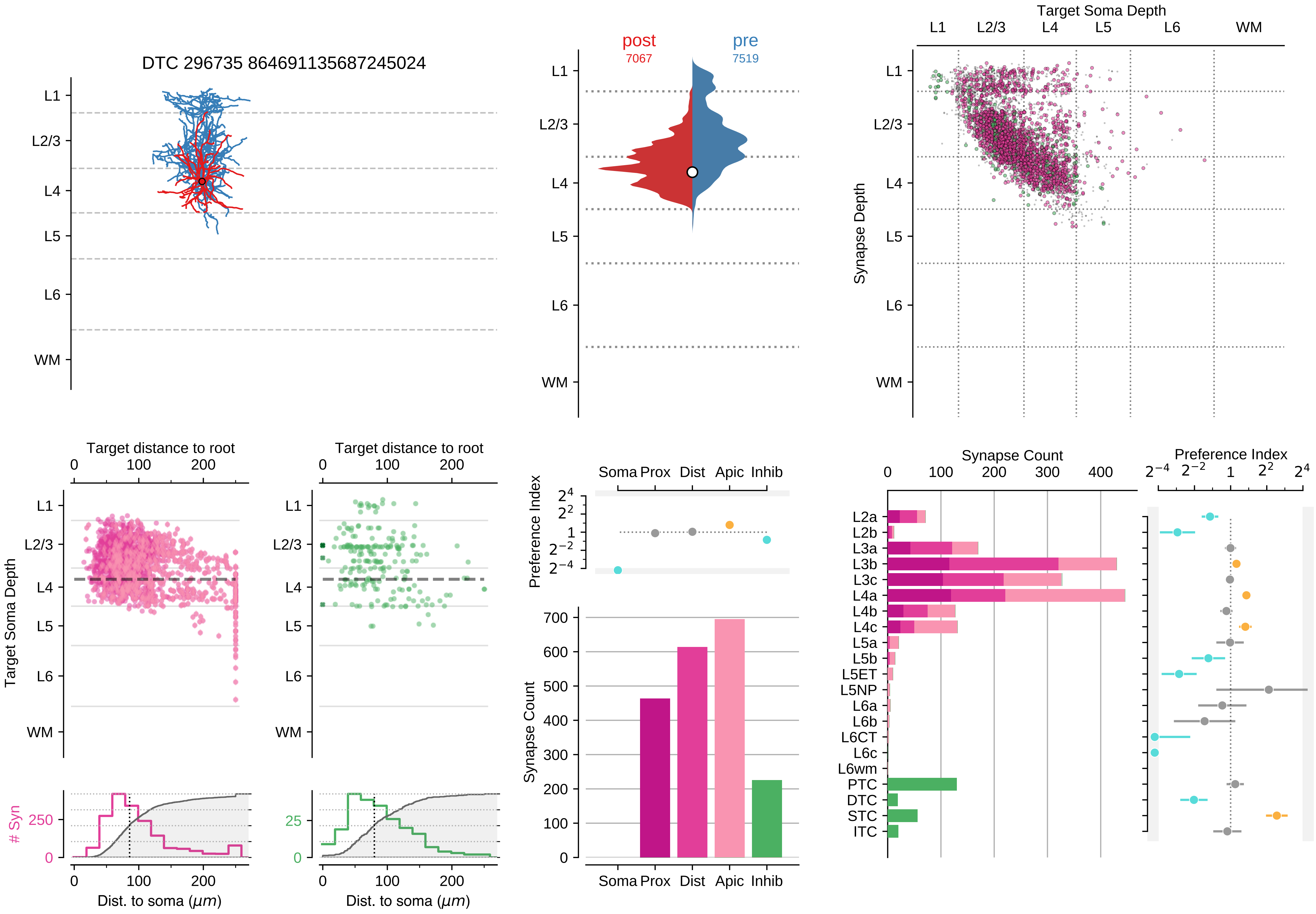

DTC 296735 864691135687245024
L1
L2/3
L4
L5
L6
WM
post
7067
pre
7519
Target Soma Depth
Synapse Depth
Target distance to root
Target Soma Depth
Dist. to soma (μm)
Syn
Preference Index
Soma Prox Dist Apic Inhib
Synapse Count
L2a
L2b
L3a
L3b
L3c
L4a
L4b
L4c
L5a
L5b
L5ET
L5NP
L6a
L6b
L6CT
L6c
L6wm
PTC
DTC
STC
ITC

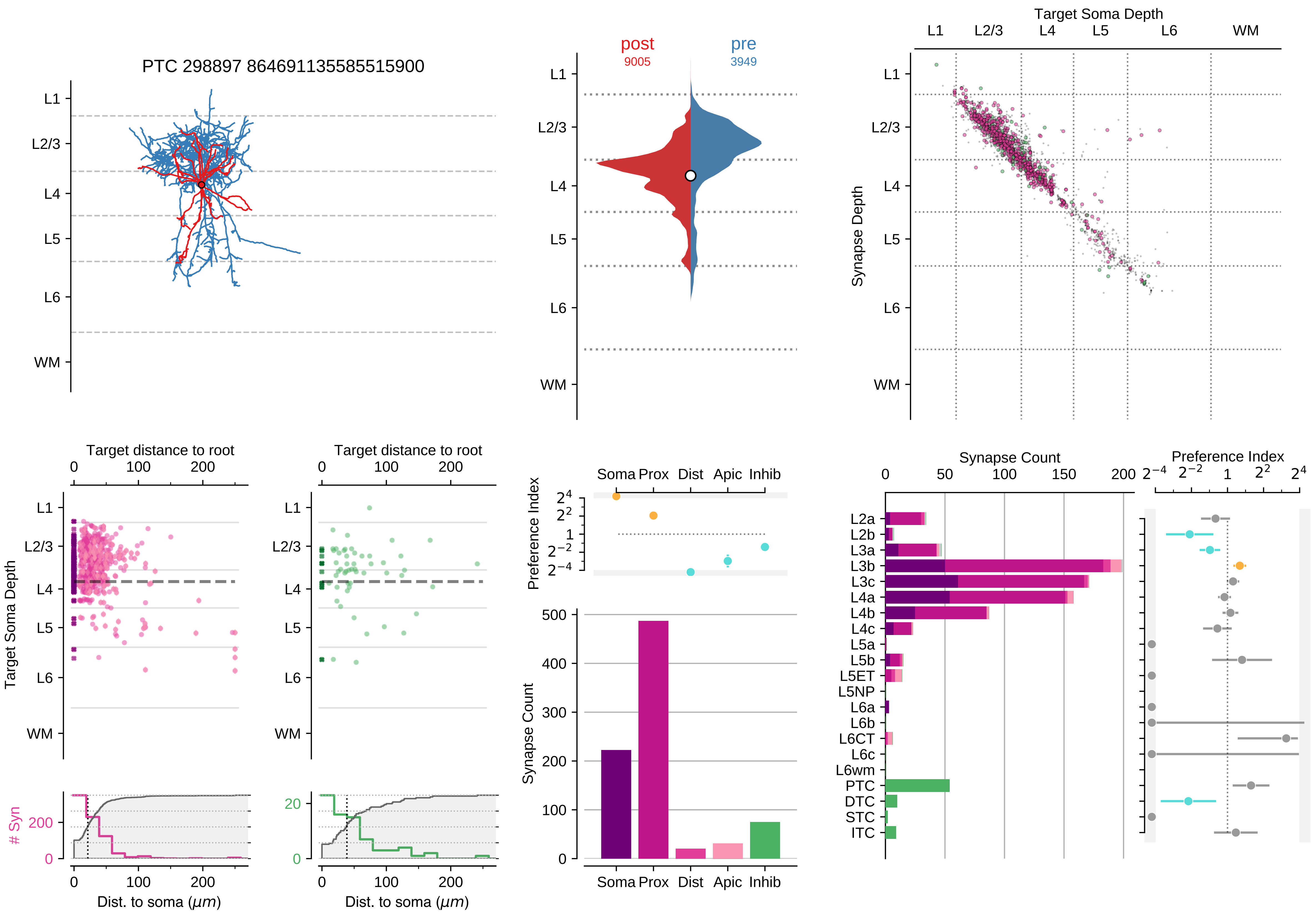
PTC 298897 864691135585515900
L1
L2/3
L4
L5
L6
WM
post
9005
pre
3949
Target Soma Depth
Synapse Depth
Target distance to root
Target Soma Depth
Syn
Dist. to soma (μm)
Preference Index
Soma Prox Dist Apic Inhib
Synapse Count
L2a
L2b
L3a
L3b
L3c
L4a
L4b
L4c
L5a
L5b
L5ET
L5NP
L6a
L6b
L6CT
L6c
L6wm
PTC
DTC
STC
ITC

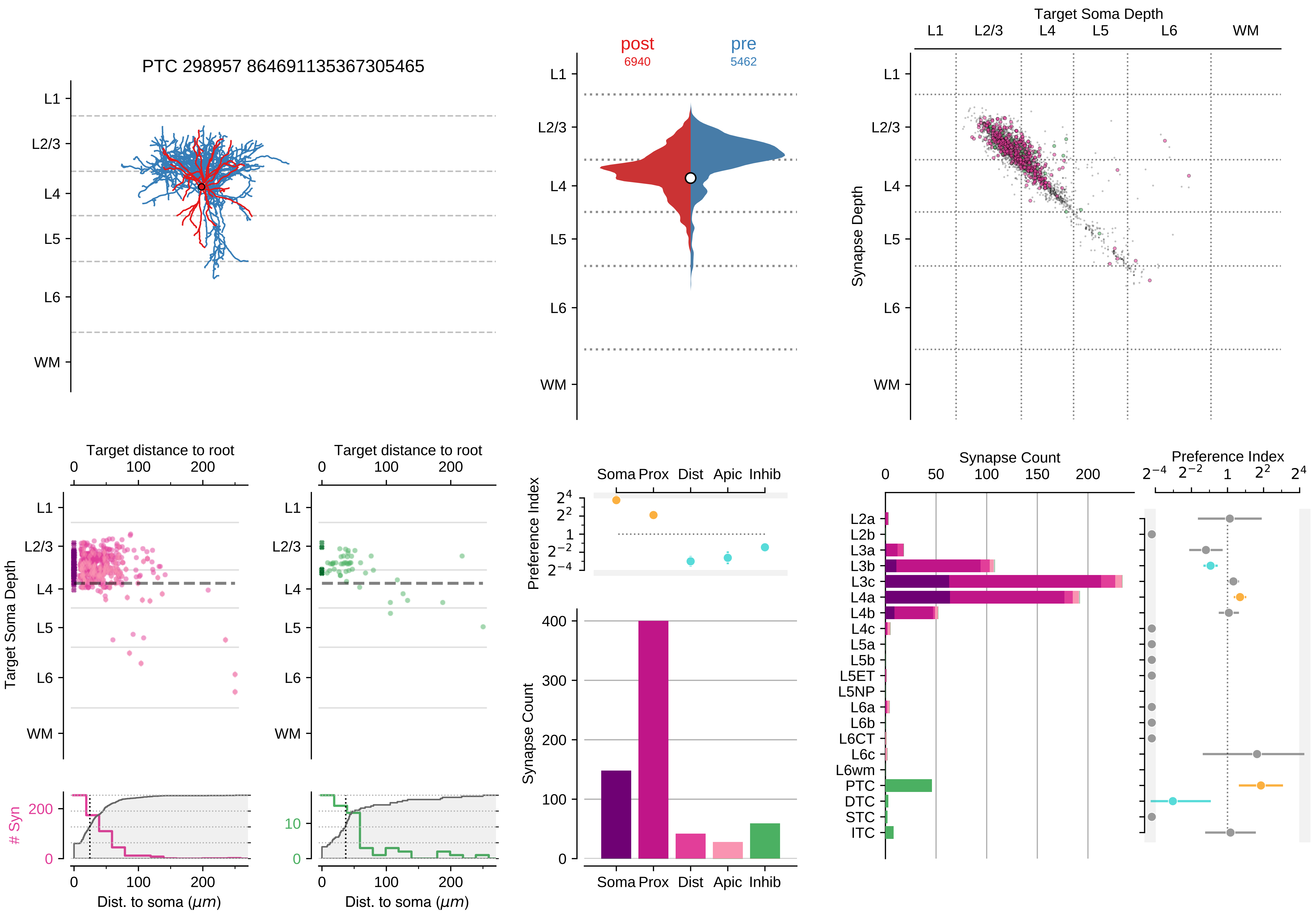
PTC 298957 864691135367305465
post
6940
pre
5462
Target Soma Depth
Synapse Depth
L1
L2/3
L4
L5
L6
WM
Target distance to root
Target Soma Depth
Syn
Dist. to soma (μm)
Preference Index
Soma
Prox
Dist
Apic
Inhib
Synapse Count
L2a
L2b
L3a
L3b
L3c
L4a
L4b
L4c
L5a
L5b
L5ET
L5NP
L6a
L6b
L6CT
L6c
L6wm
PTC
DTC
STC
ITC

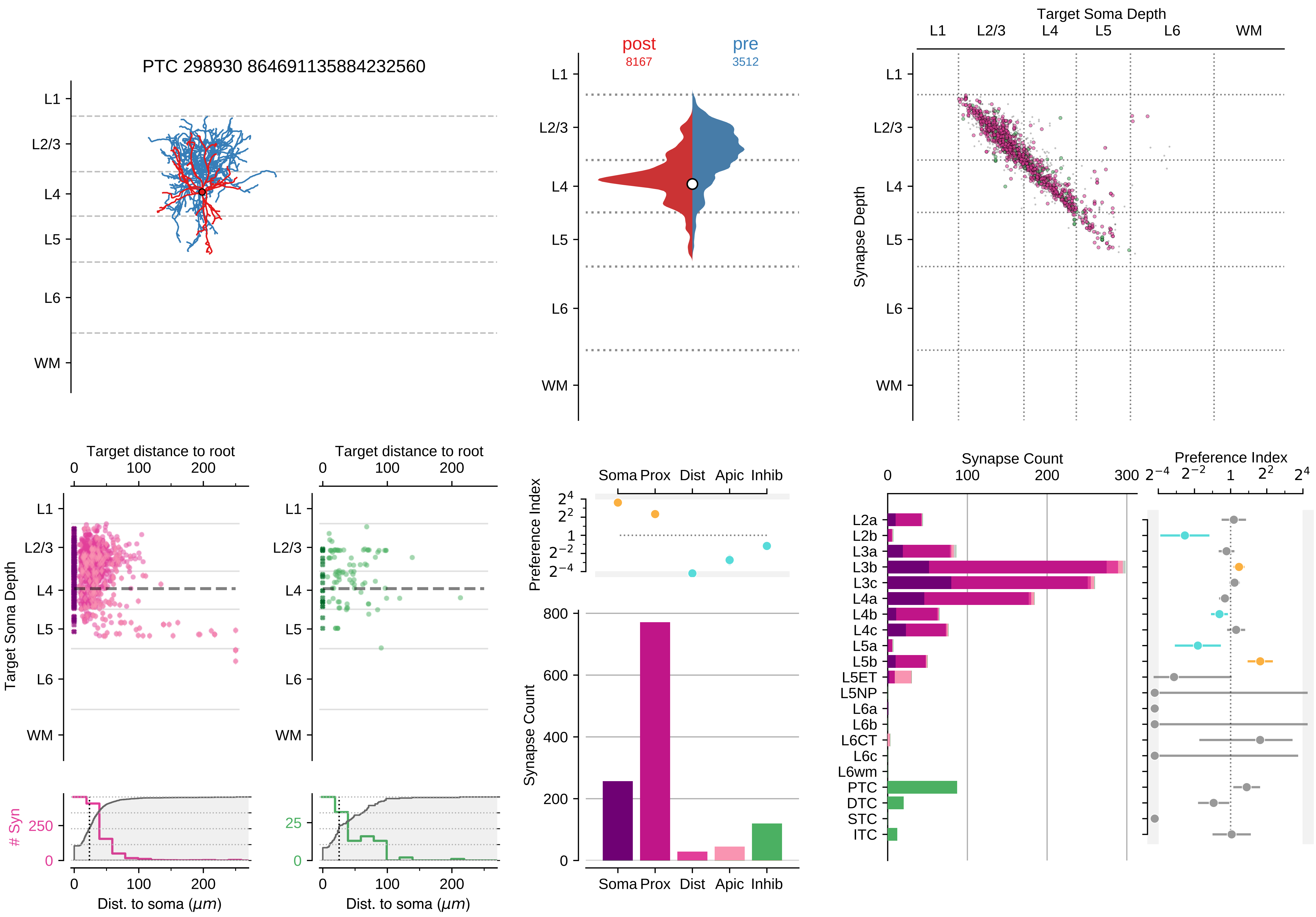
PTC 298930 864691135884232560
post
8167
pre
3512
Target Soma Depth
L1
L2/3
L4
L5
L6
WM
Synapse Depth
Target distance to root
Target Soma Depth
Syn
Dist. to soma (μm)
Preference Index
Soma
Prox
Dist
Apic
Inhib
Synapse Count
L2a
L2b
L3a
L3b
L3c
L4a
L4b
L4c
L5a
L5b
L5ET
L5NP
L6a
L6b
L6CT
L6c
L6wm
PTC
DTC
STC
ITC

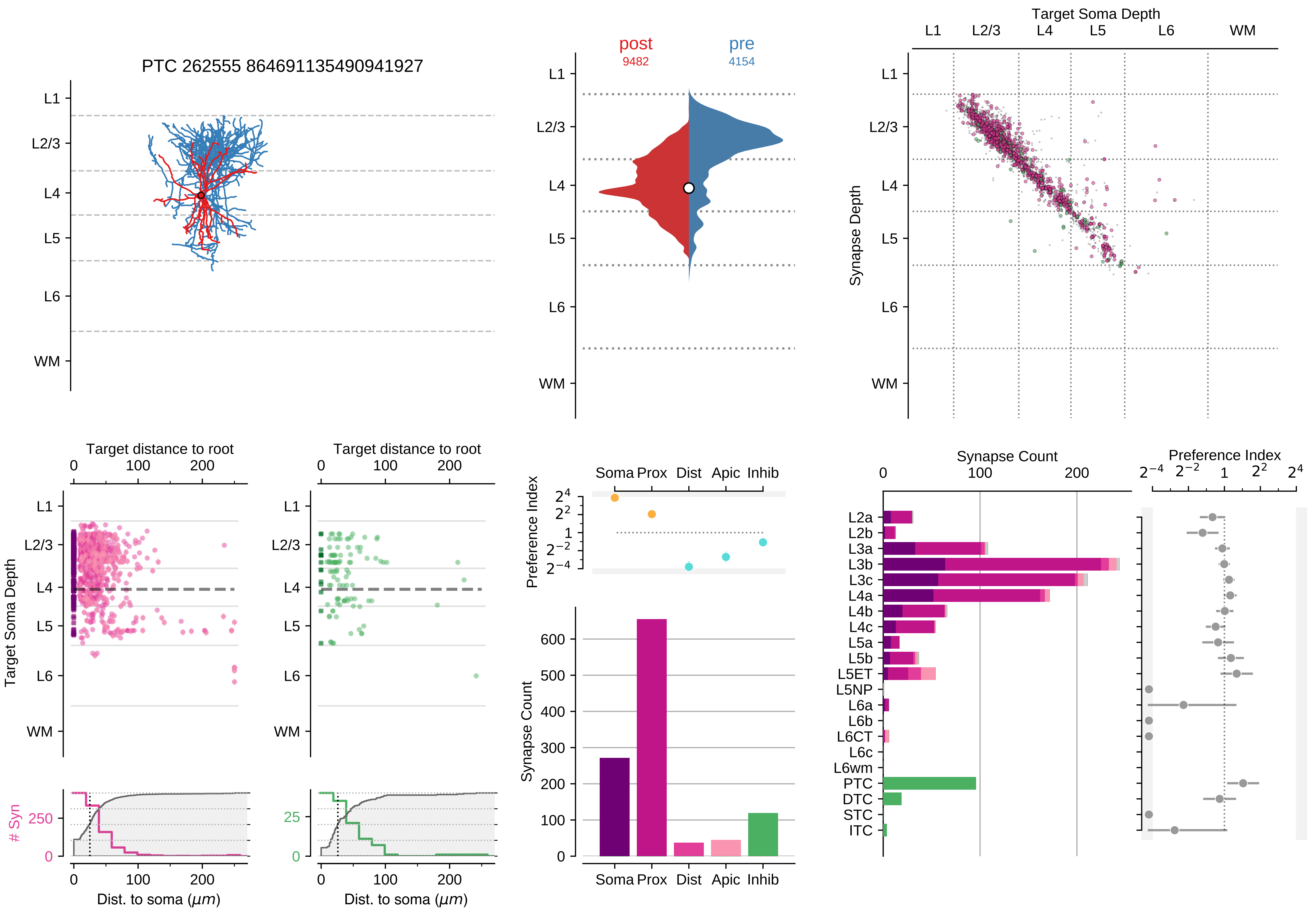
PTC 262555 864691135490941927
L1
L2/3
L4
L5
L6
WM
post
9482
pre
4154
Target Soma Depth
Synapse Depth
Target distance to root
Target Soma Depth
Syn
Dist. to soma (μm)
Preference Index
Soma Prox Dist Apic Inhib
Synapse Count
L2a
L2b
L3a
L3b
L3c
L4a
L4b
L4c
L5a
L5b
L5ET
L5NP
L6a
L6b
L6CT
L6c
L6wm
PTC
DTC
STC
ITC

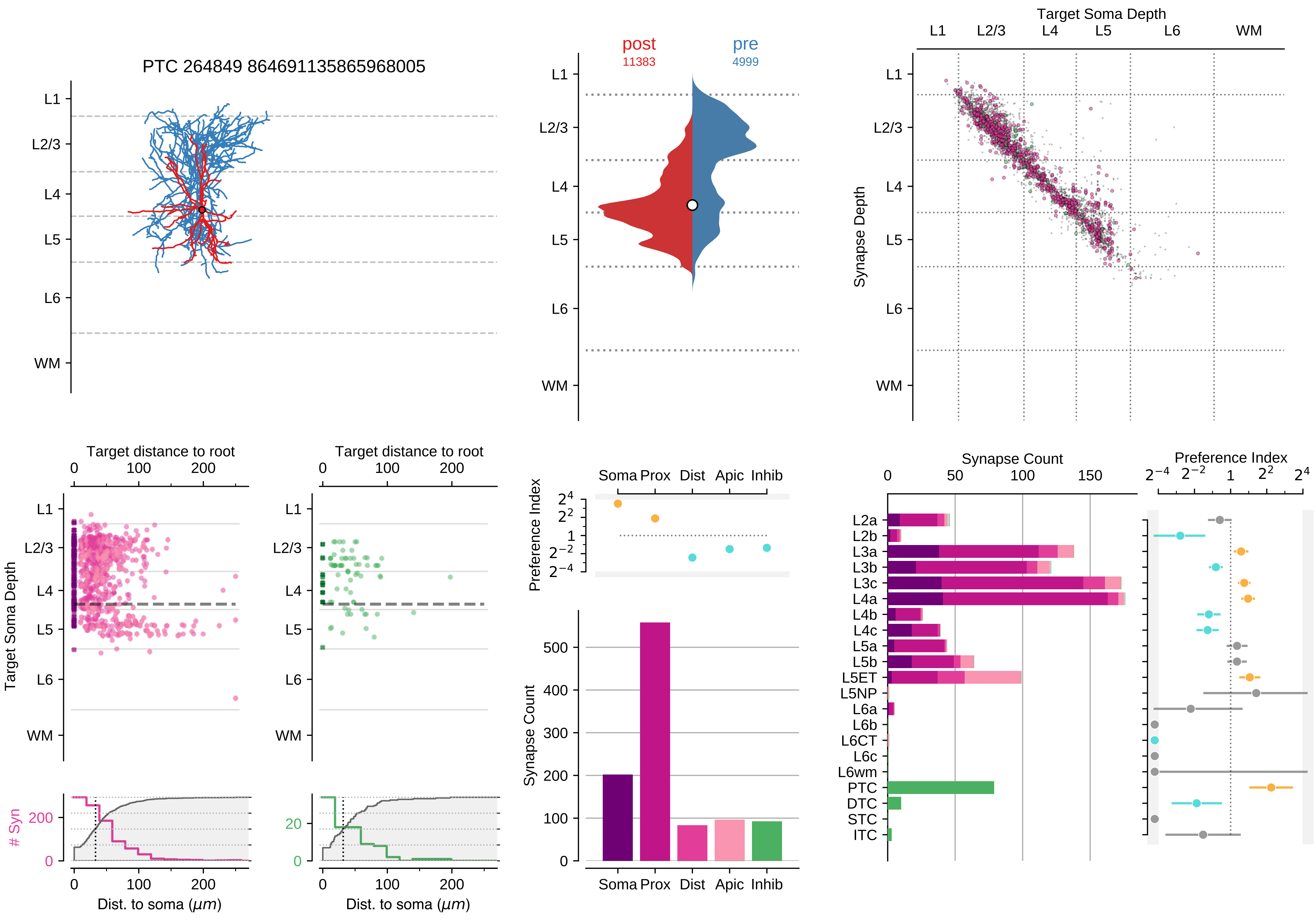
PTC 264849 864691135865968005
post
11383
pre
4999
Target Soma Depth
L1
L2/3
L4
L5
L6
WM
Synapse Depth
Target distance to root
Target Soma Depth
Dist. to soma (μm)
Syn
Preference Index
Soma
Prox
Dist
Apic
Inhib
Synapse Count
L2a
L2b
L3a
L3b
L3c
L4a
L4b
L4c
L5a
L5b
L5ET
L5NP
L6a
L6b
L6CT
L6c
L6wm
PTC
DTC
STC
ITC

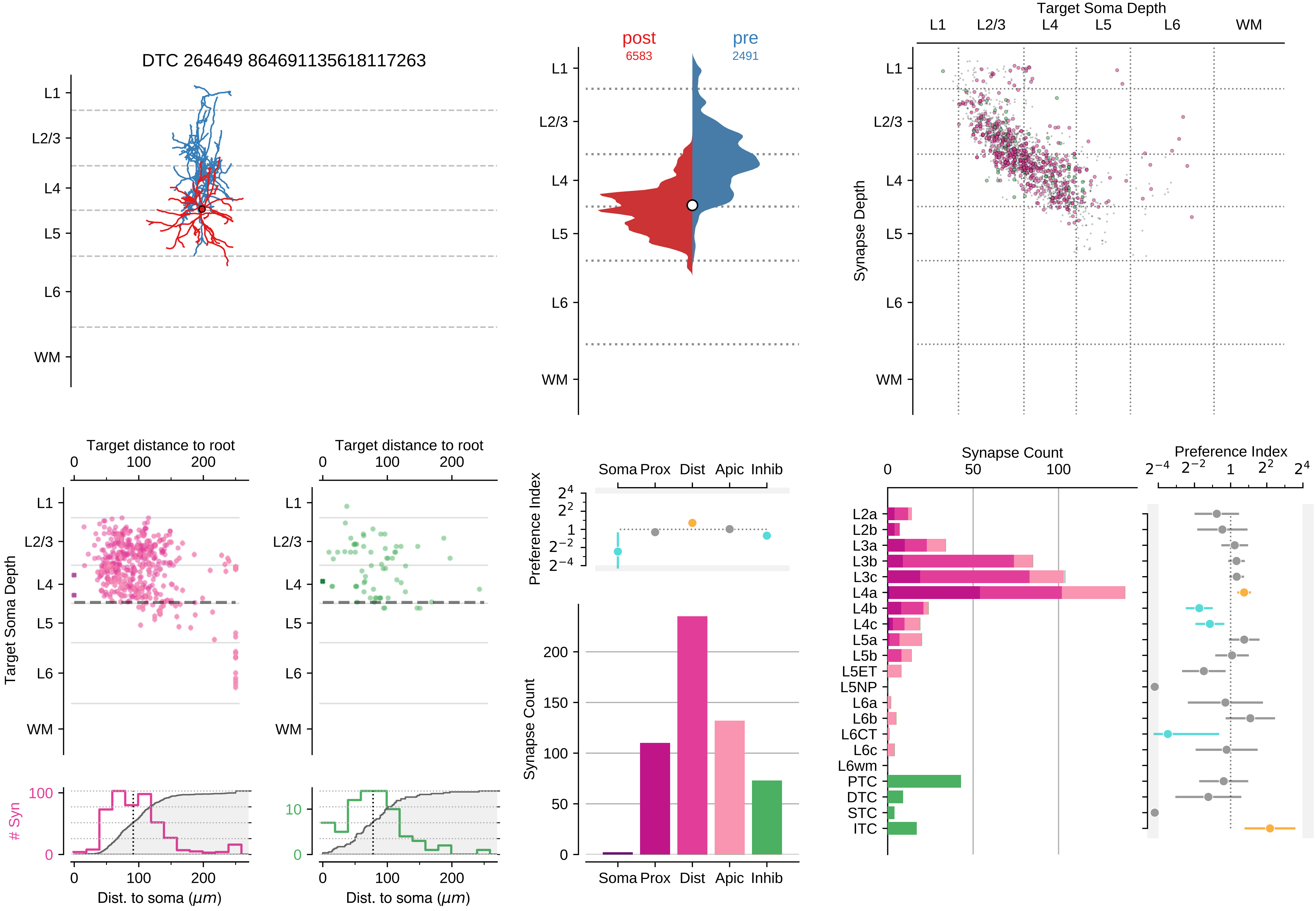

DTC 264649 864691135618117263
L1
L2/3
L4
L5
L6
WM
post
6583
pre
2491
Target Soma Depth
Synapse Depth
Target distance to root
0
100
200
Target Soma Depth
Syn
Dist. to soma (μm)
Preference Index
Soma
Prox
Dist
Apic
Inhib
Synapse Count
L2a
L2b
L3a
L3b
L3c
L4a
L4b
L4c
L5a
L5b
L5ET
L5NP
L6a
L6b
L6CT
L6c
L6wm
PTC
DTC
STC
ITC

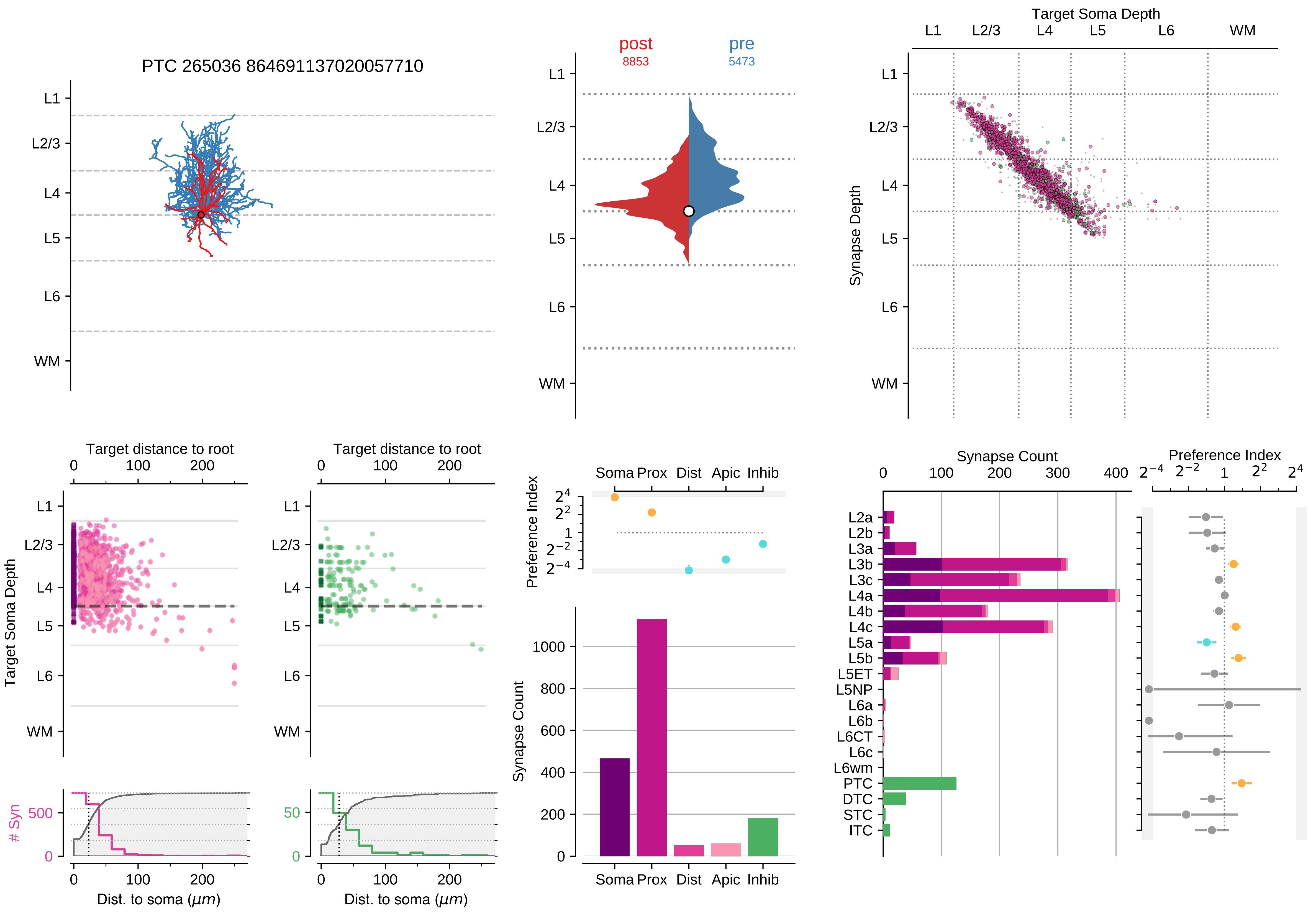

PTC 265036 864691137020057710
L1
L2/3
L4
L5
L6
WM
post
8853
pre
5473
Target Soma Depth
Synapse Depth
Target distance to root
Target Soma Depth
Syn
Dist. to soma (μm)
Preference Index
Soma Prox Dist Apic Inhib
Synapse Count
L2a
L2b
L3a
L3b
L3c
L4a
L4b
L4c
L5a
L5b
L5ET
L5NP
L6a
L6b
L6CT
L6c
L6wm
PTC
DTC
STC
ITC

Group 5

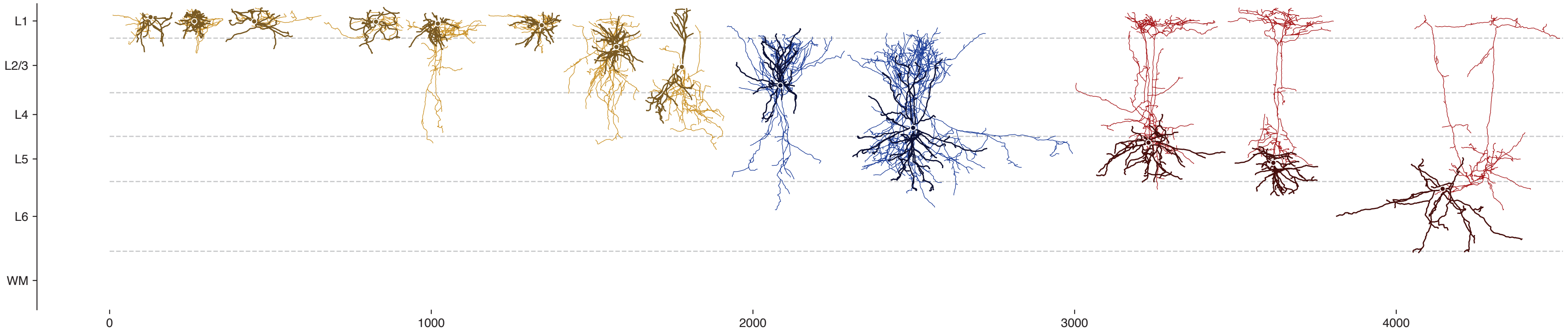

L1
L2/3
L4
L5
L6
WM
0
1000
2000
3000
4000

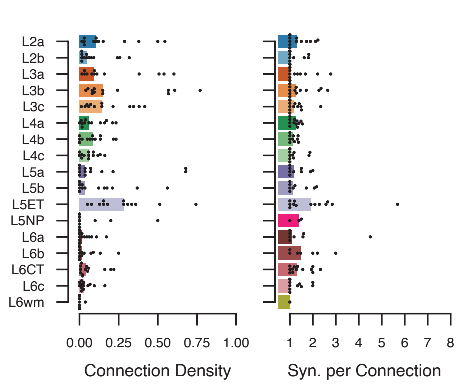

L2a
L2b
L3a
L3b
L3c
L4a
L4b
L4c
L5a
L5b
L5ET
L5NP
L6a
L6b
L6CT
L6c
L6wm
0.00
0.25
0.50
0.75
1.00
Connection Density
1
2
3
4
5
6
7
8
Syn. per Connection

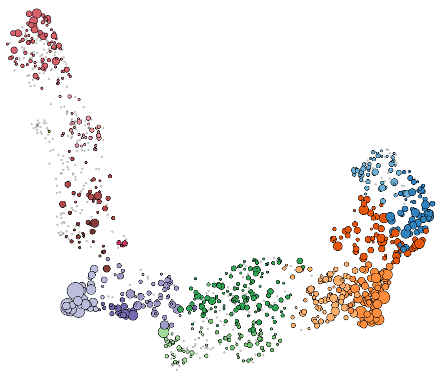

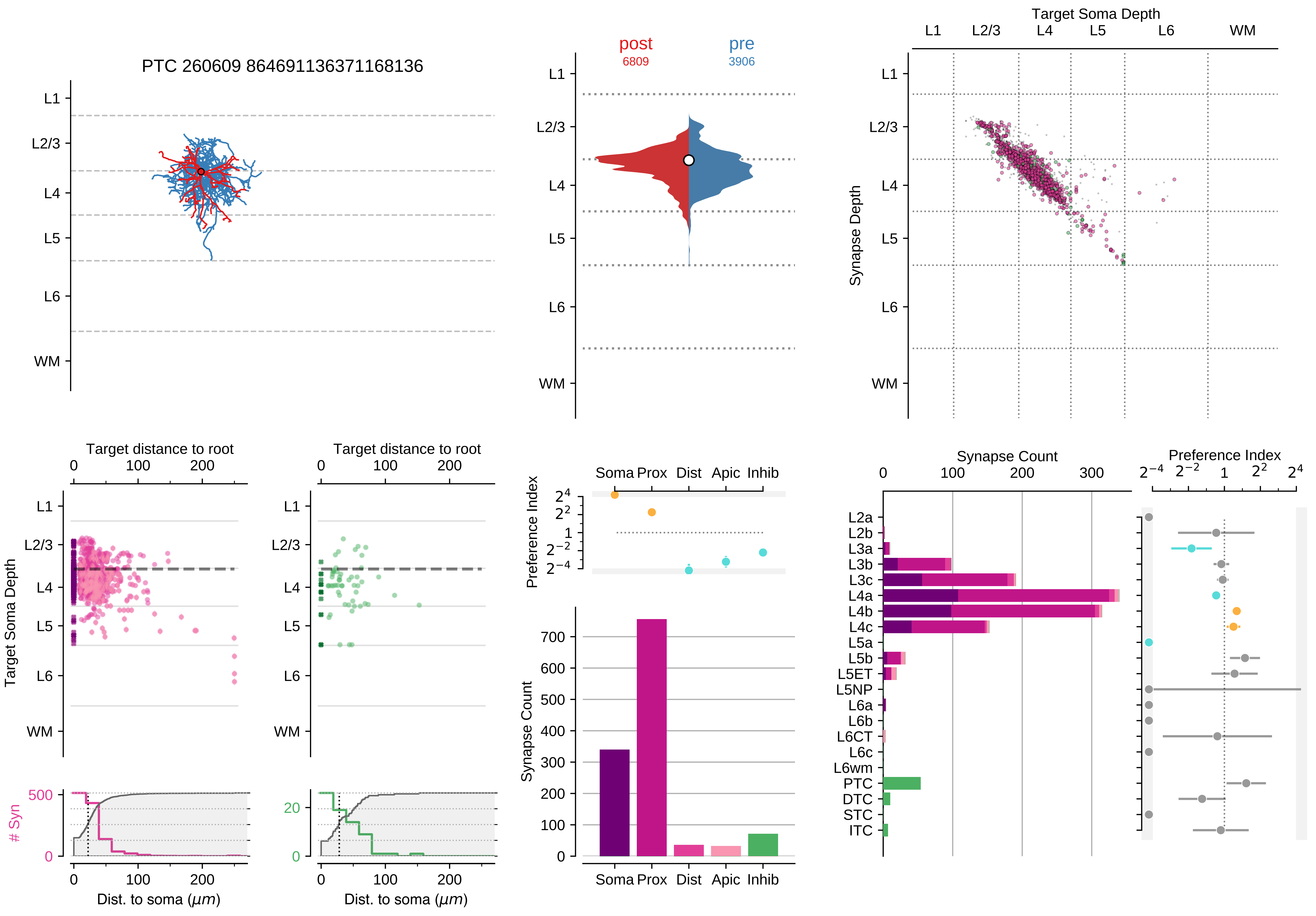
PTC 260609 864691136371168136
L1
L2/3
L4
L5
L6
WM
post
6809
pre
3906
Target Soma Depth
Synapse Depth
Target distance to root
Target Soma Depth
Syn
Dist. to soma (μm)
Preference Index
Soma Prox Dist Apic Inhib
Synapse Count
L2a
L2b
L3a
L3b
L3c
L4a
L4b
L4c
L5a
L5b
L5ET
L5NP
L6a
L6b
L6CT
L6c
L6wm
PTC
DTC
STC
ITC

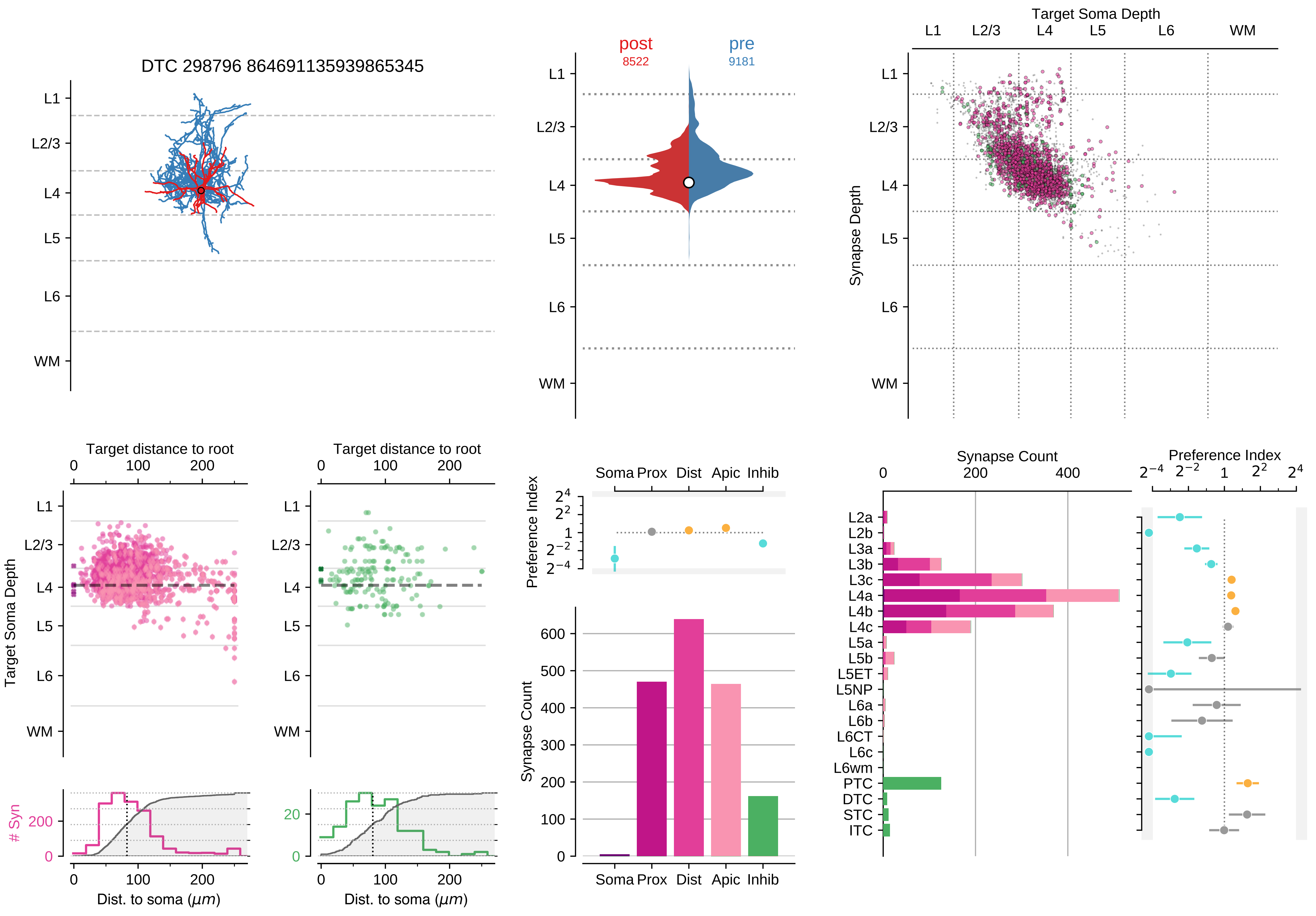

DTC 298796 864691135939865345
post
8522
pre
9181
Target Soma Depth
Synapse Depth
L1
L2/3
L4
L5
L6
WM
Target distance to root
Target Soma Depth
Syn
Dist. to soma (μm)
Preference Index
Soma Prox Dist Apic Inhib
Synapse Count
L2a
L2b
L3a
L3b
L3c
L4a
L4b
L4c
L5a
L5b
L5ET
L5NP
L6a
L6b
L6CT
L6c
L6wm
PTC
DTC
STC
ITC

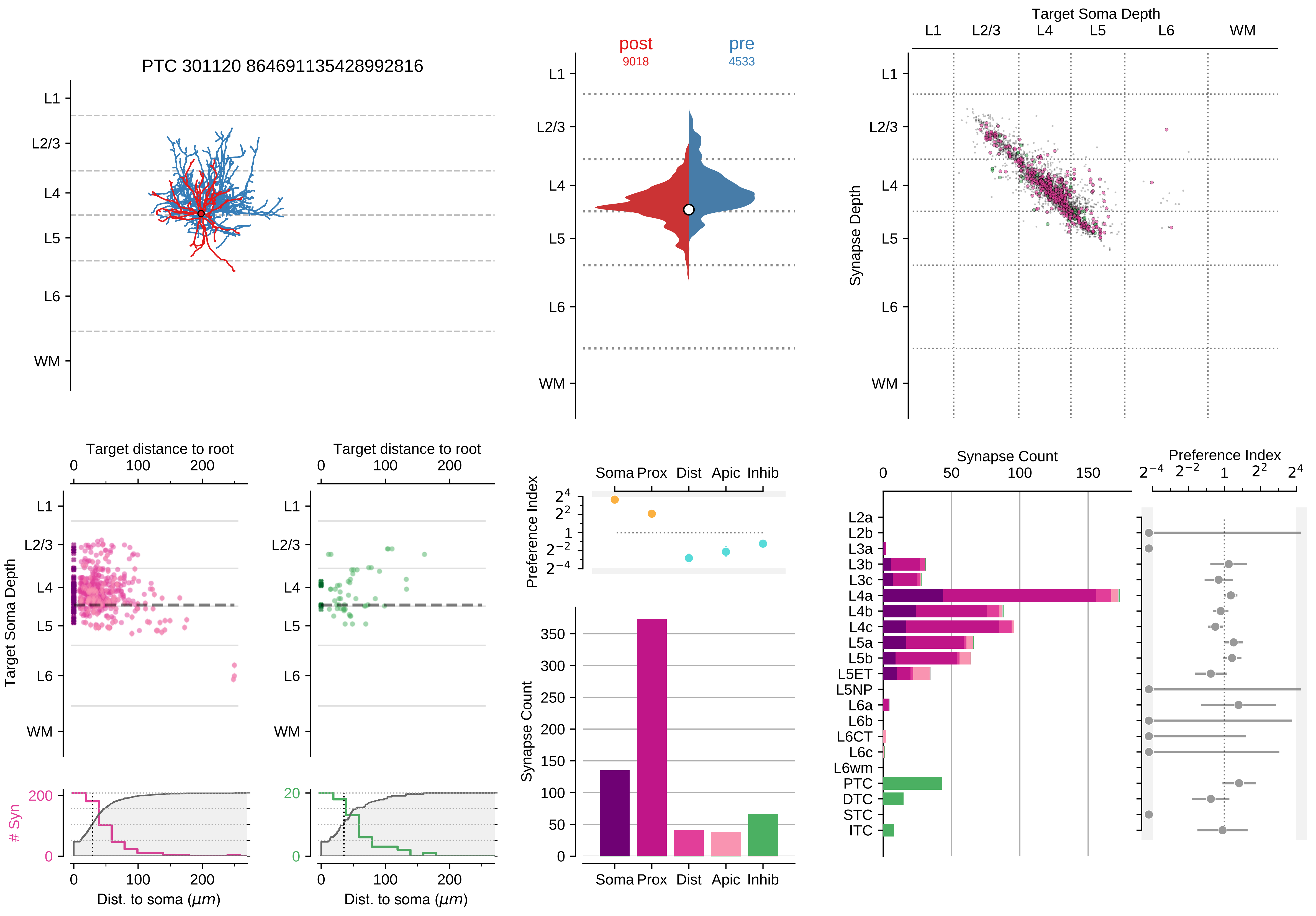

PTC 301120 864691135428992816
L1
L2/3
L4
L5
L6
WM
post
9018
pre
4533
Target Soma Depth
Synapse Depth
Target distance to root
Target Soma Depth
Syn
Dist. to soma (μm)
Preference Index
Soma
Prox
Dist
Apic
Inhib
Synapse Count
L2a
L2b
L3a
L3b
L3c
L4a
L4b
L4c
L5a
L5b
L5ET
L5NP
L6a
L6b
L6CT
L6c
L6wm
PTC
DTC
STC
ITC

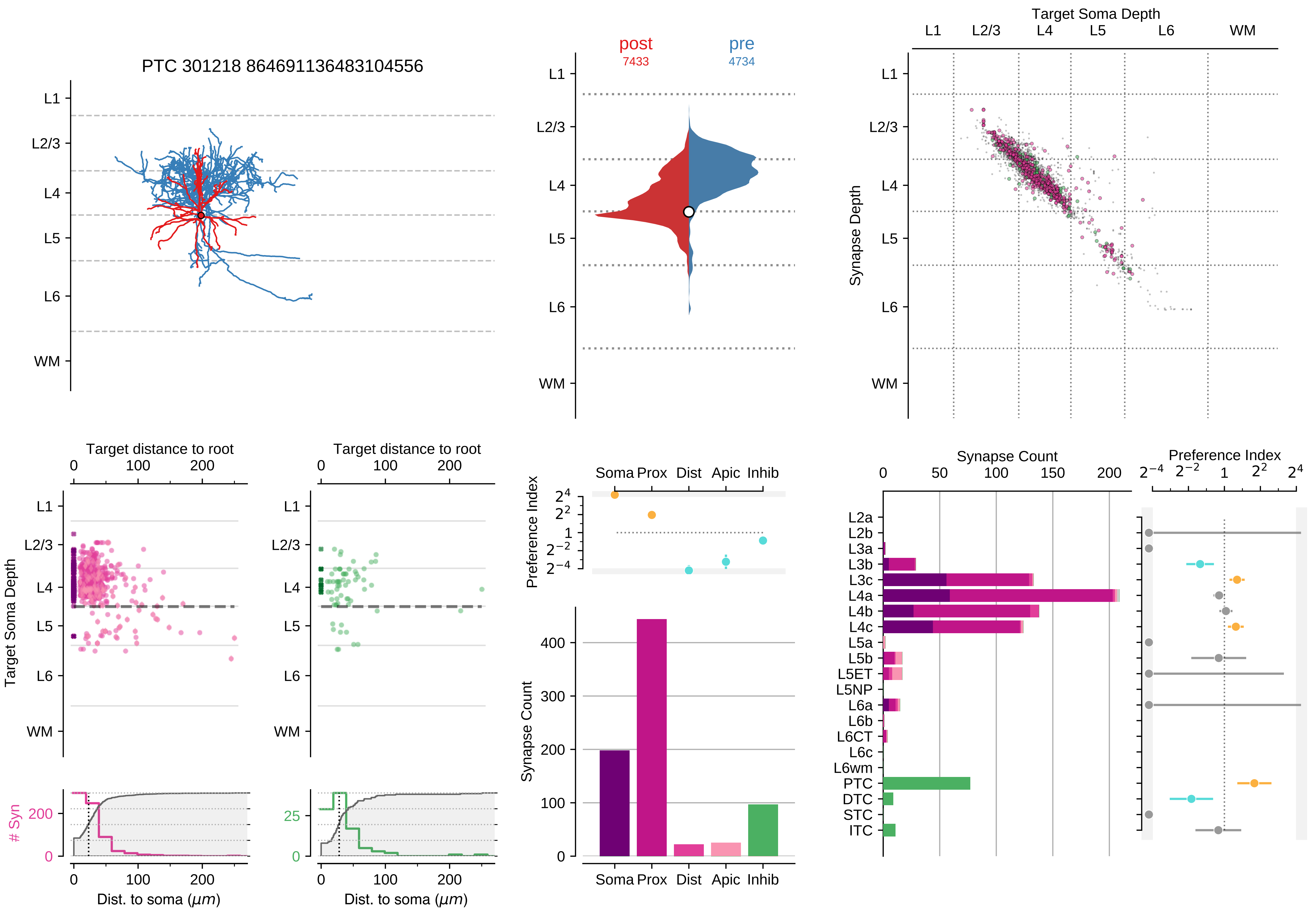

PTC 301218 864691136483104556
L1
L2/3
L4
L5
L6
WM
post
7433
pre
4734
Target Soma Depth
Synapse Depth
Target distance to root
Target Soma Depth
Syn
Dist. to soma (μm)
Preference Index
Soma
Prox
Dist
Apic
Inhib
Synapse Count
L2a
L2b
L3a
L3b
L3c
L4a
L4b
L4c
L5a
L5b
L5ET
L5NP
L6a
L6b
L6CT
L6c
L6wm
PTC
DTC
STC
ITC

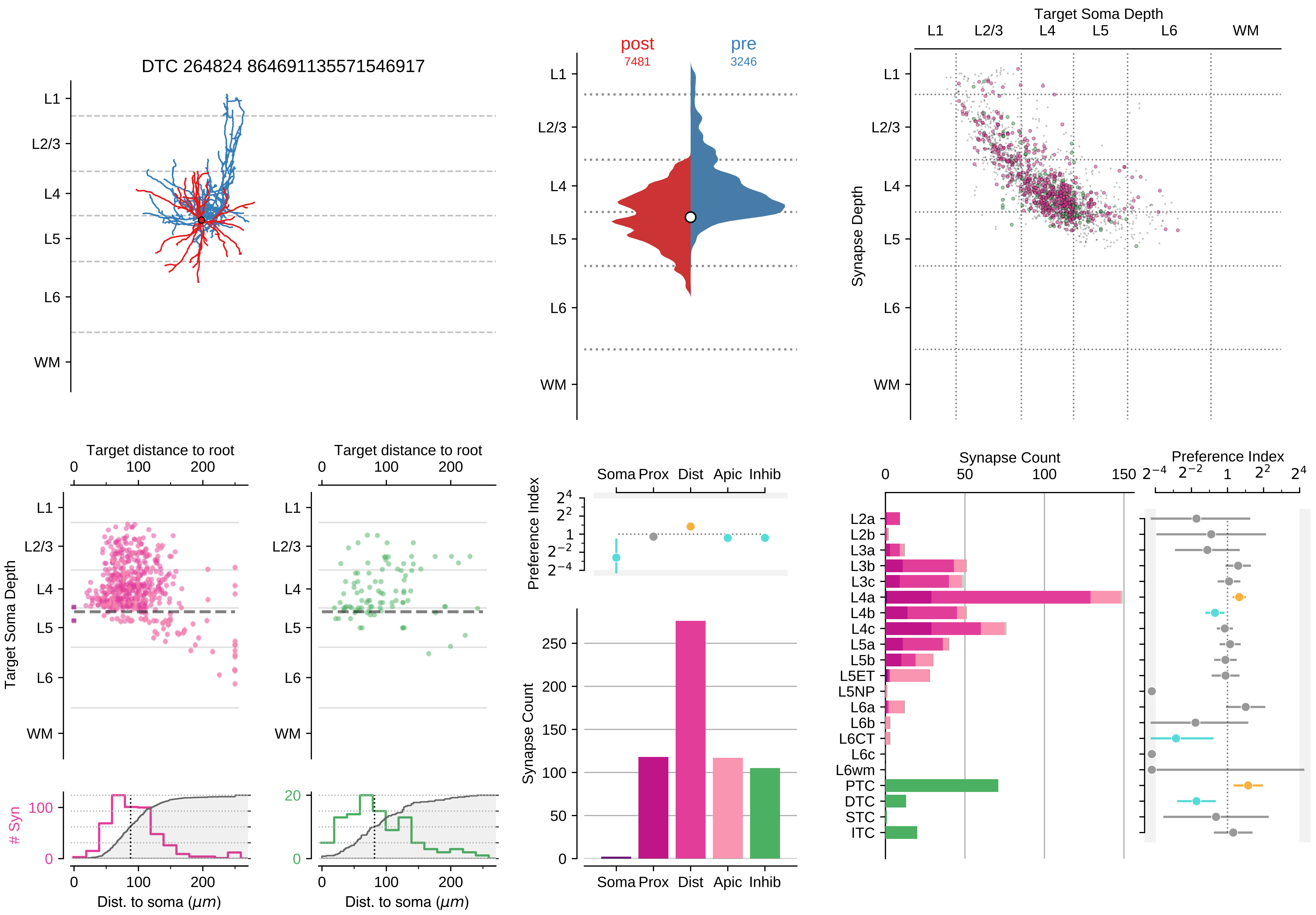

DTC 264824 864691135571546917
L1
L2/3
L4
L5
L6
WM
post
7481
pre
3246
Target Soma Depth
Synapse Depth
Target distance to root
Target Soma Depth
Syn
Dist. to soma (μm)
Preference Index
Soma
Prox
Dist
Apic
Inhib
Synapse Count
L2a
L2b
L3a
L3b
L3c
L4a
L4b
L4c
L5a
L5b
L5ET
L5NP
L6a
L6b
L6CT
L6c
L6wm
PTC
DTC
STC
ITC

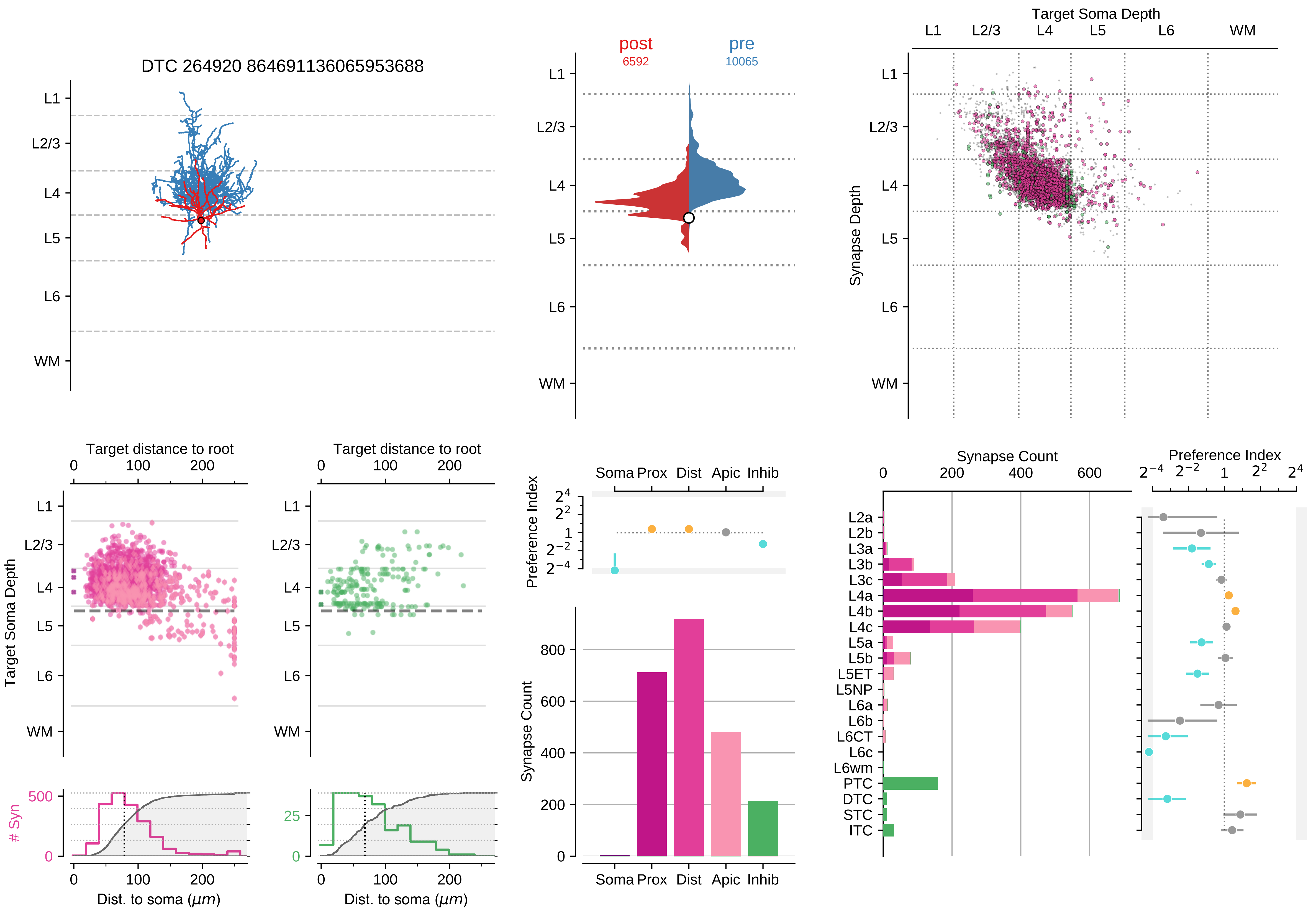

DTC 264920 864691136065953688
post
6592
pre
10065
Target Soma Depth
Synapse Depth
L1
L2/3
L4
L5
L6
WM
Target distance to root
Target Soma Depth
Syn
Dist. to soma (μm)
Preference Index
Soma Prox Dist Apic Inhib
Synapse Count
L2a
L2b
L3a
L3b
L3c
L4a
L4b
L4c
L5a
L5b
L5ET
L5NP
L6a
L6b
L6CT
L6c
L6wm
PTC
DTC
STC
ITC

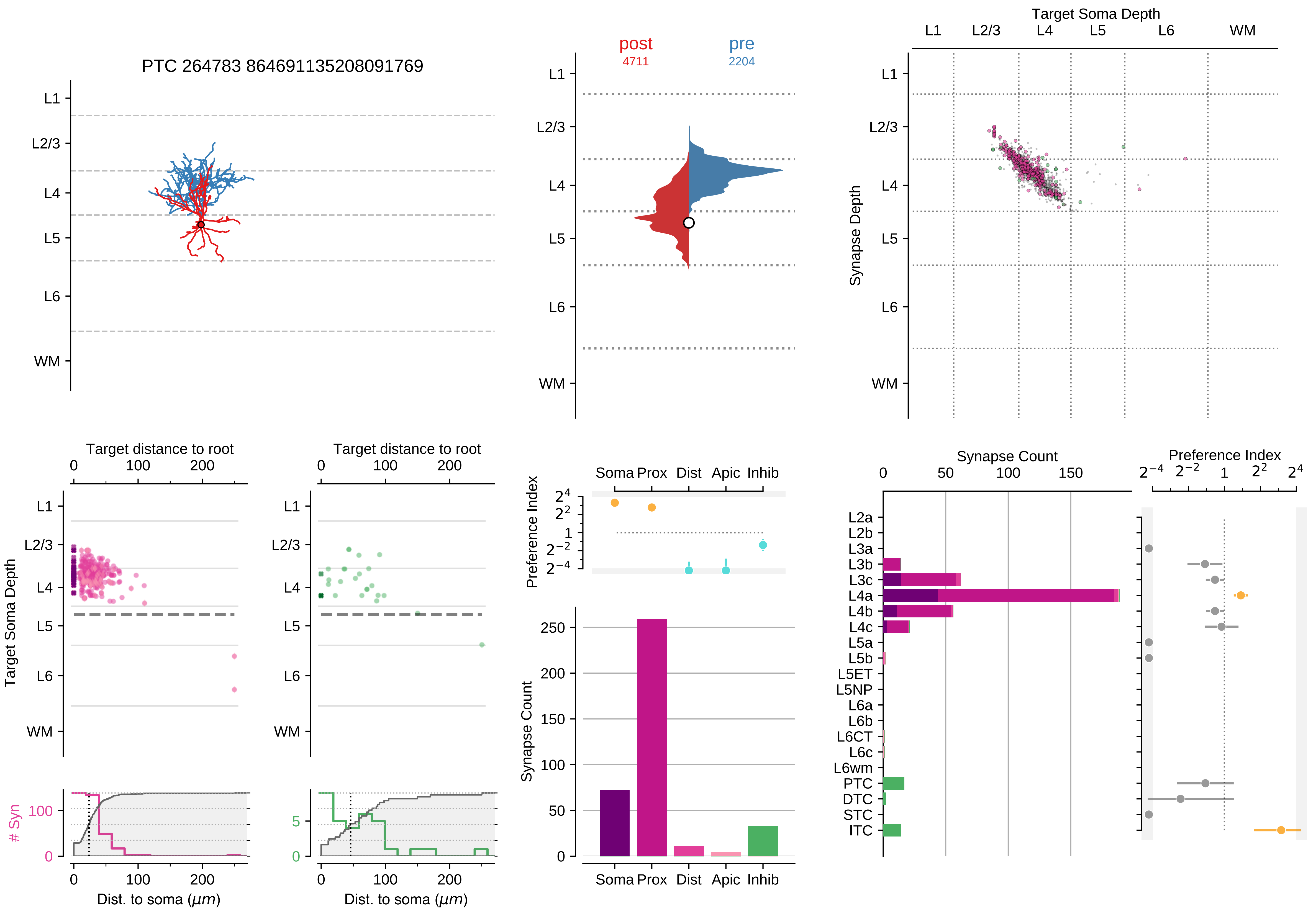

PTC 264783 864691135208091769
L1
L2/3
L4
L5
L6
WM
post
4711
pre
2204
Target Soma Depth
Synapse Depth
Target distance to root
Target Soma Depth
Syn
Dist. to soma (μm)
Preference Index
Soma Prox Dist Apic Inhib
Synapse Count
Synapse Count
Preference Index
L2a
L2b
L3a
L3b
L3c
L4a
L4b
L4c
L5a
L5b
L5ET
L5NP
L6a
L6b
L6CT
L6c
L6wm
PTC
DTC
STC
ITC

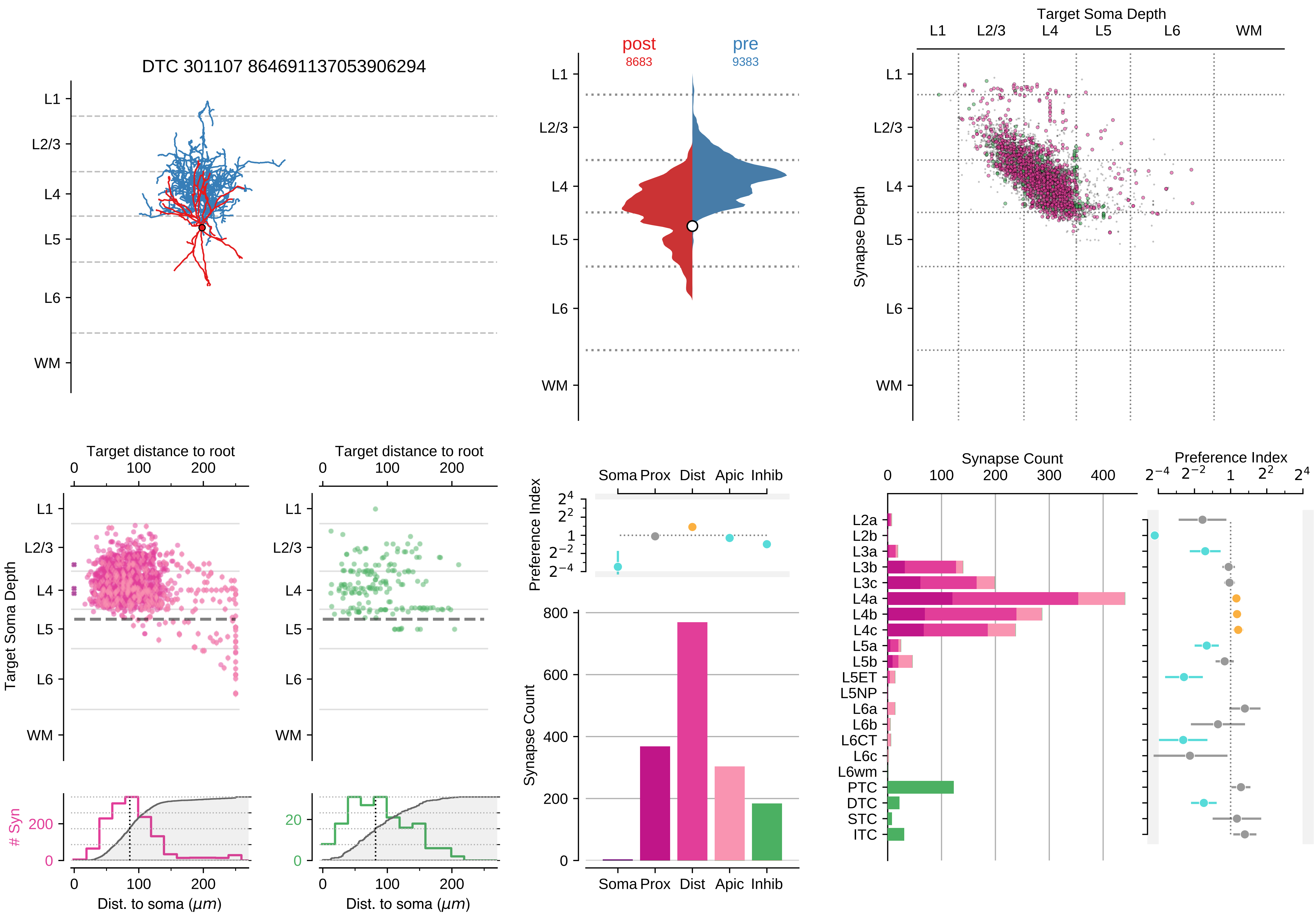

DTC 301107 864691137053906294
L1
L2/3
L4
L5
L6
WM
post
8683
pre
9383
Target Soma Depth
Synapse Depth
Target distance to root
Target Soma Depth
Syn
Dist. to soma (μm)
Preference Index
Soma Prox Dist Apic Inhib
Synapse Count
L2a
L2b
L3a
L3b
L3c
L4a
L4b
L4c
L5a
L5b
L5ET
L5NP
L6a
L6b
L6CT
L6c
L6wm
PTC
DTC
STC
ITC

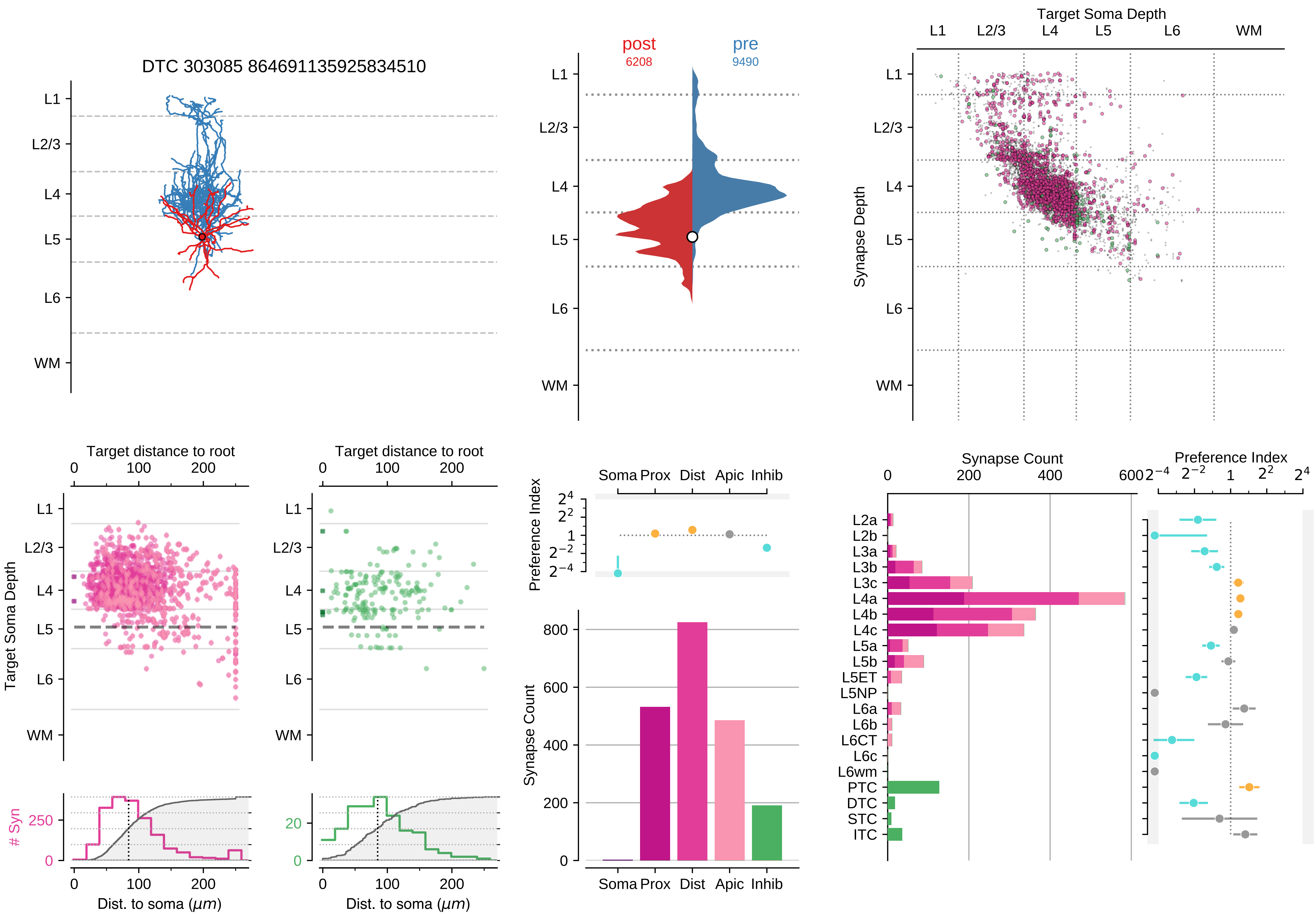

DTC 303085 864691135925834510
L1
L2/3
L4
L5
L6
WM
post
6208
pre
9490
Target Soma Depth
Synapse Depth
Target distance to root
Target Soma Depth
Syn
Dist. to soma (μm)
Preference Index
Soma Prox Dist Apic Inhib
Synapse Count
L2a
L2b
L3a
L3b
L3c
L4a
L4b
L4c
L5a
L5b
L5ET
L5NP
L6a
L6b
L6CT
L6c
L6wm
PTC
DTC
STC
ITC

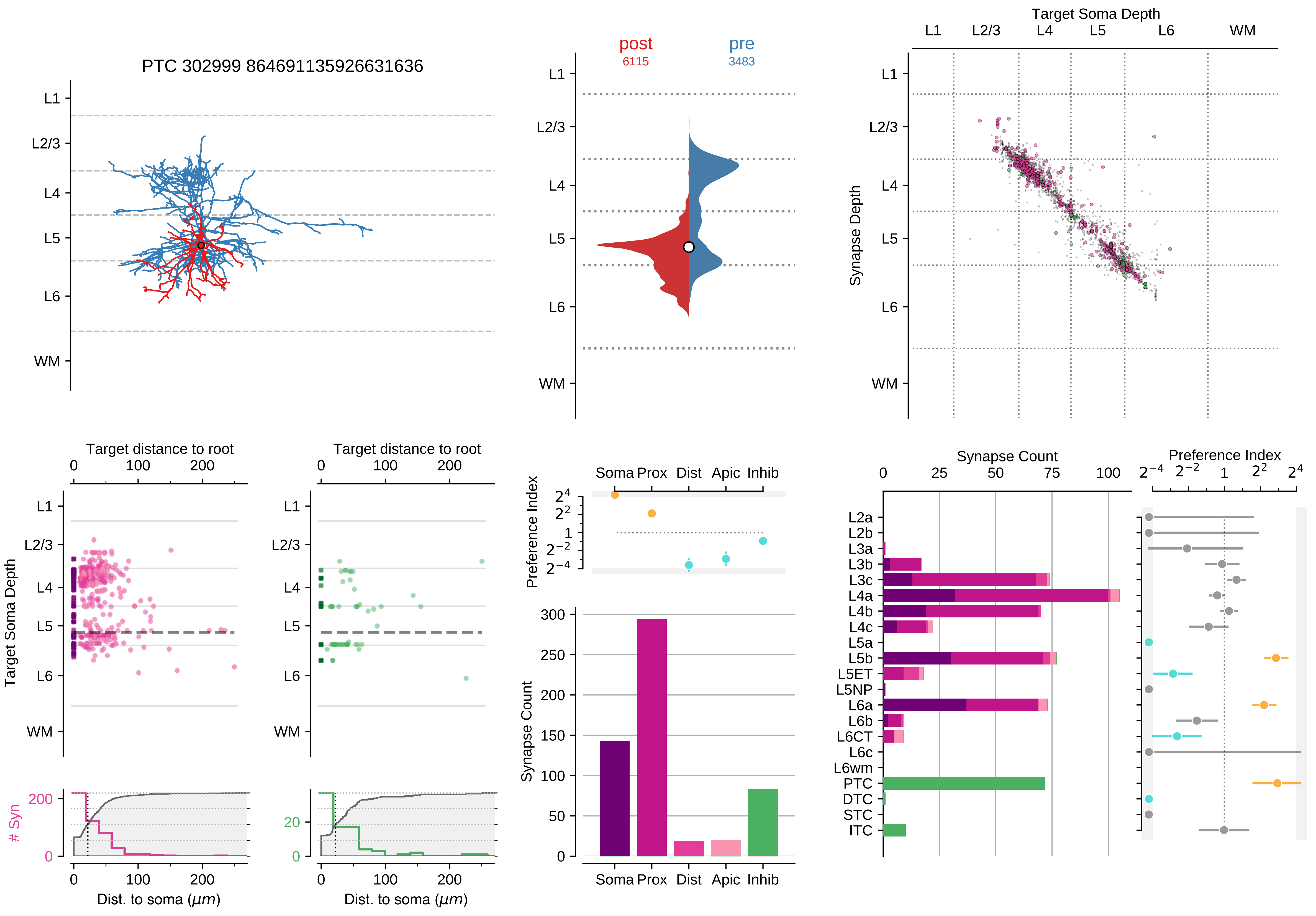

PTC 302999 864691135926631636
L1
L2/3
L4
L5
L6
WM
post
6115
pre
3483
Target Soma Depth
Synapse Depth
Target distance to root
Target Soma Depth
Dist. to soma (μm)
Syn
Preference Index
Soma Prox Dist Apic Inhib
Synapse Count
Synapse Count
Preference Index
L2a
L2b
L3a
L3b
L3c
L4a
L4b
L4c
L5a
L5b
L5ET
L5NP
L6a
L6b
L6CT
L6c
L6wm
PTC
DTC
STC
ITC

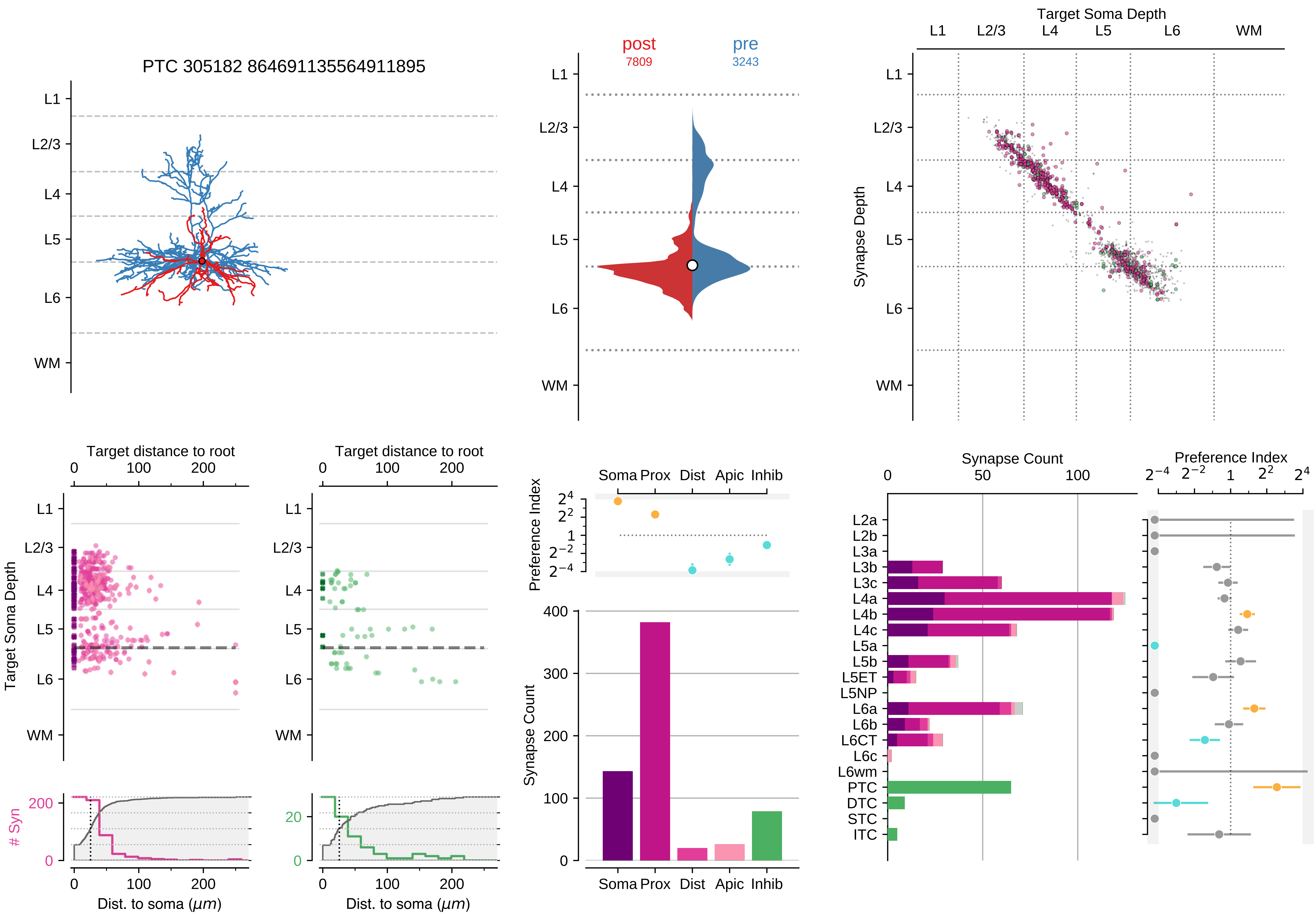

PTC 305182 864691135564911895
L1
L2/3
L4
L5
L6
WM
post
7809
pre
3243
Target Soma Depth
Synapse Depth
Target distance to root
Target Soma Depth
Syn
Dist. to soma (μm)
Preference Index
Soma
Prox
Dist
Apic
Inhib
Synapse Count
L2a
L2b
L3a
L3b
L3c
L4a
L4b
L4c
L5a
L5b
L5ET
L5NP
L6a
L6b
L6CT
L6c
L6wm
PTC
DTC
STC
ITC

Group 6

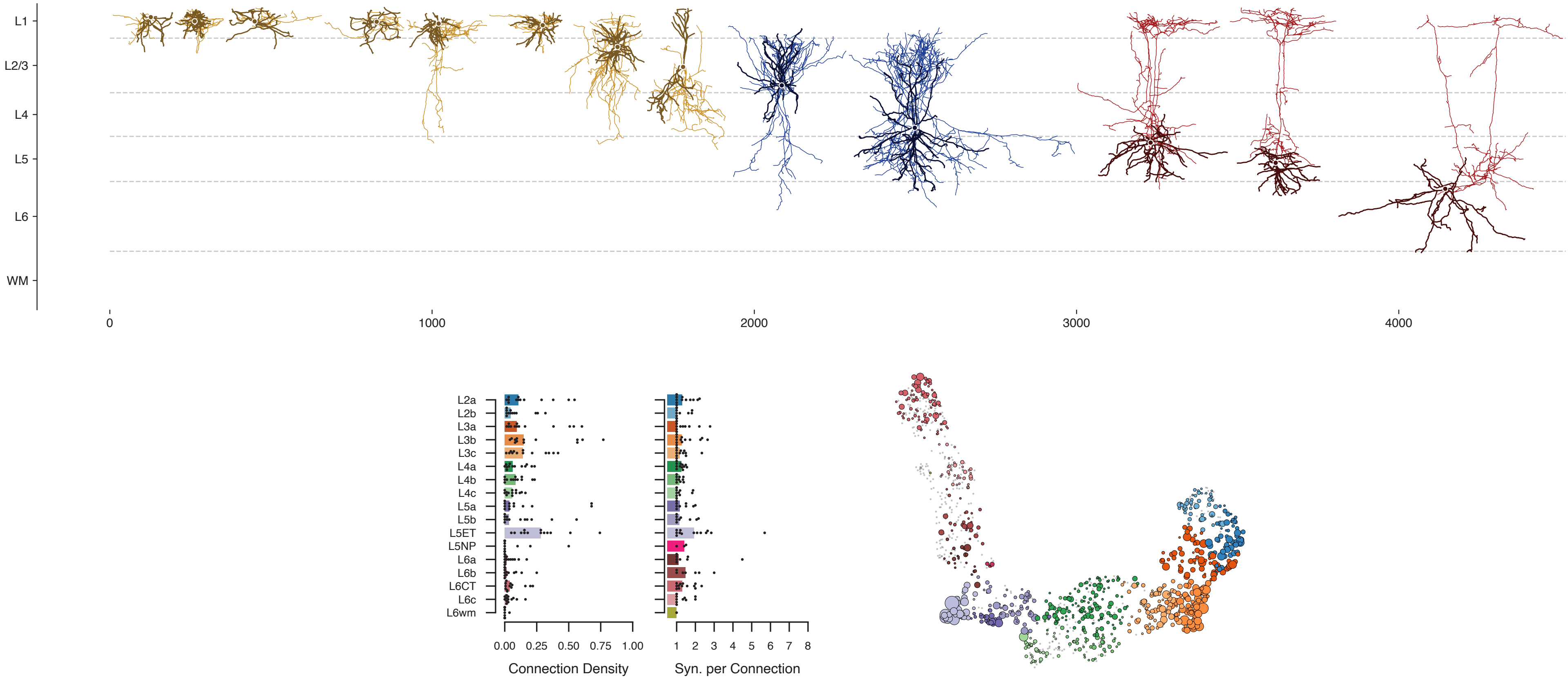

L1
L2/3
L4
L5
L6
WM
0
1000
2000
3000
4000
L2a
L2b
L3a
L3b
L3c
L4a
L4b
L4c
L5a
L5b
L5ET
L5NP
L6a
L6b
L6CT
L6c
L6wm
0.00
0.25
0.50
0.75
1.00
Connection Density
1
2
3
4
5
6
7
8
Syn. per Connection

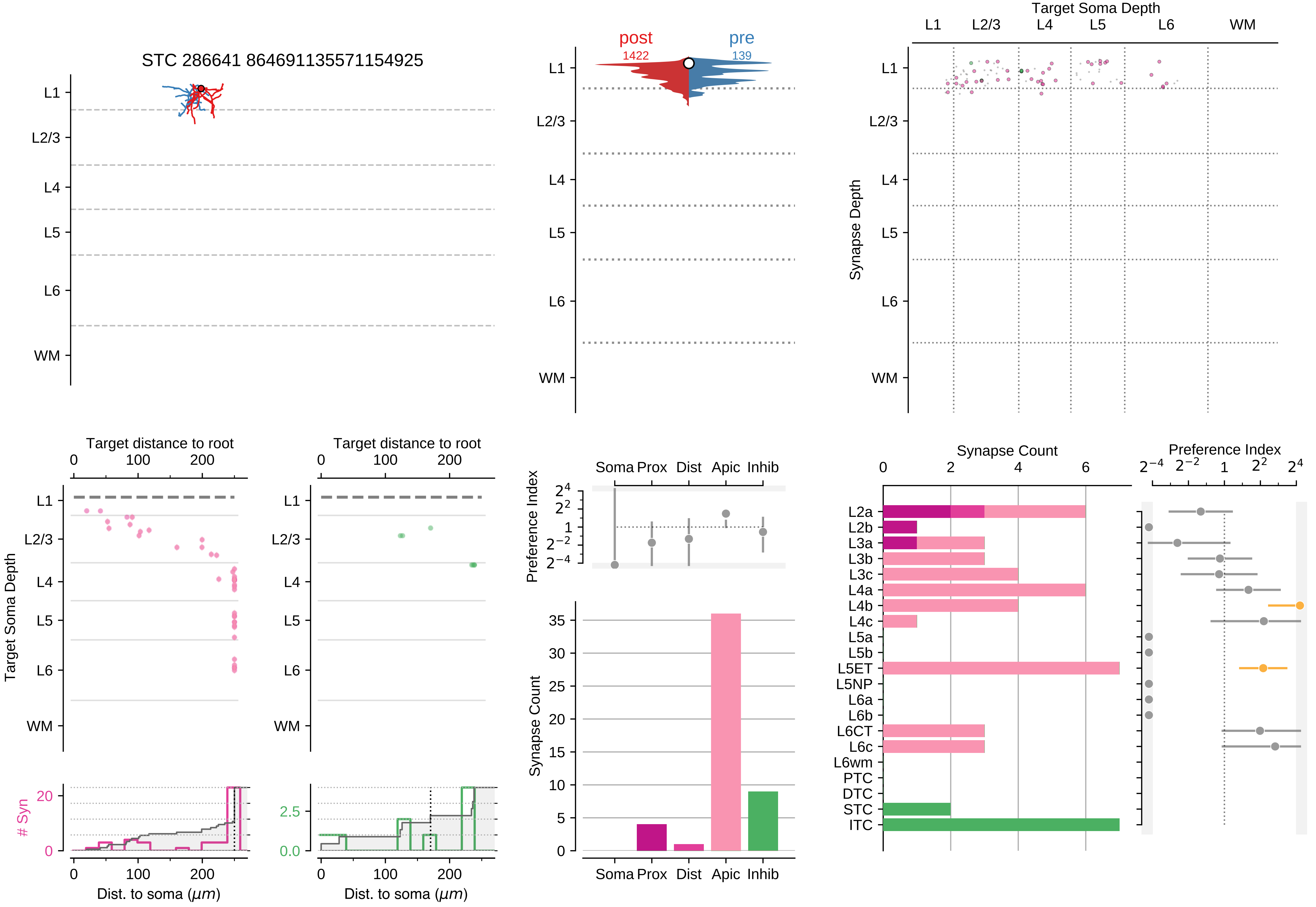
STC 286641 864691135571154925
L1
L2/3
L4
L5
L6
WM
post
1422
pre
139
Target Soma Depth
Synapse Depth
Target distance to root
Target Soma Depth
Syn
Dist. to soma (μm)
Preference Index
Soma Prox Dist Apic Inhib
Synapse Count
L2a
L2b
L3a
L3b
L3c
L4a
L4b
L4c
L5a
L5b
L5ET
L5NP
L6a
L6b
L6CT
L6c
L6wm
PTC
DTC
STC
ITC

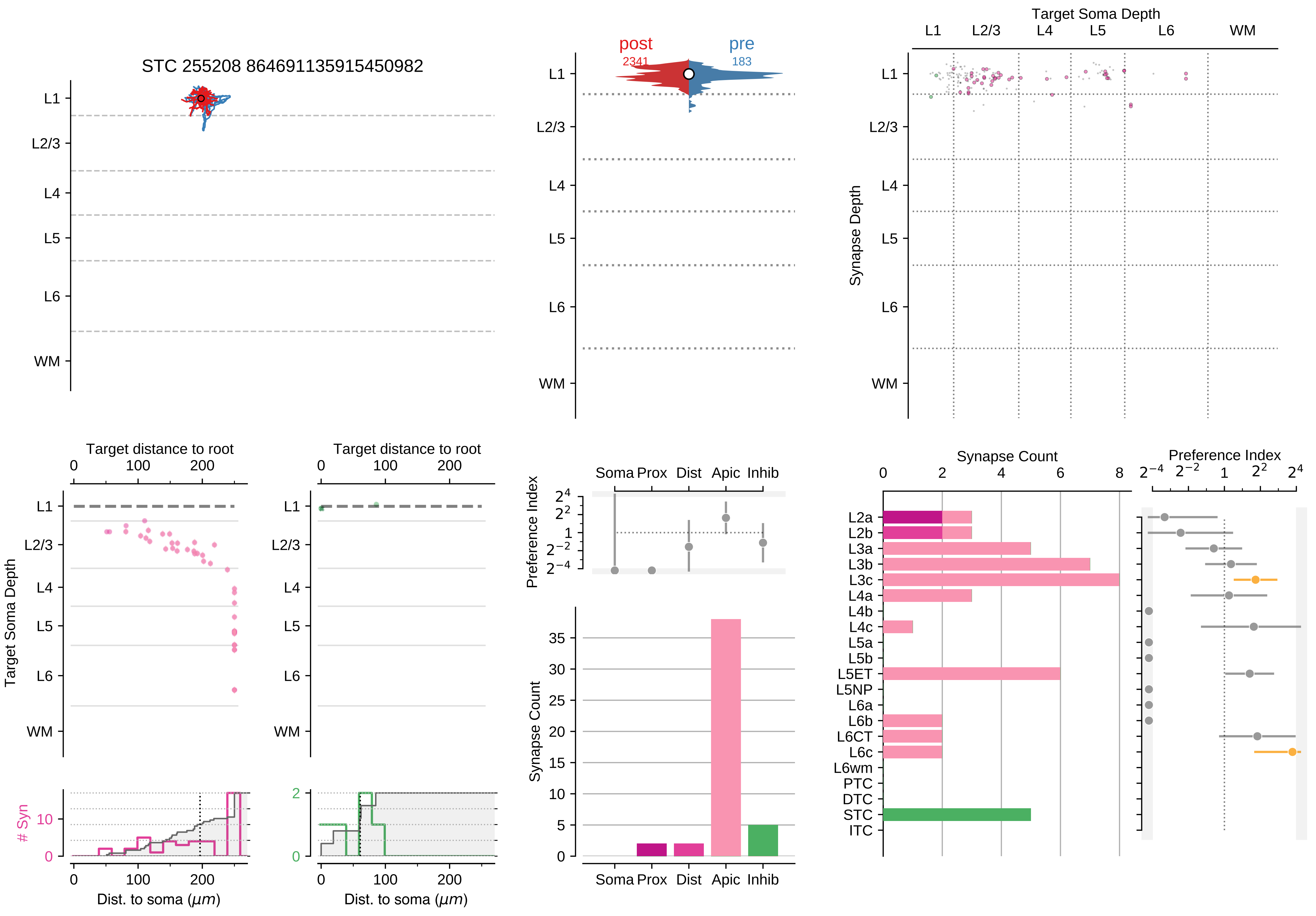
STC 255208 864691135915450982
post
2341
pre
183
Target Soma Depth
Synapse Depth
L1
L2/3
L4
L5
L6
WM
Target distance to root
Target Soma Depth
Syn
Dist. to soma (μm)
Preference Index
Soma Prox Dist Apic Inhib
Synapse Count
L2a
L2b
L3a
L3b
L3c
L4a
L4b
L4c
L5a
L5b
L5ET
L5NP
L6a
L6b
L6CT
L6c
L6wm
PTC
DTC
STC
ITC

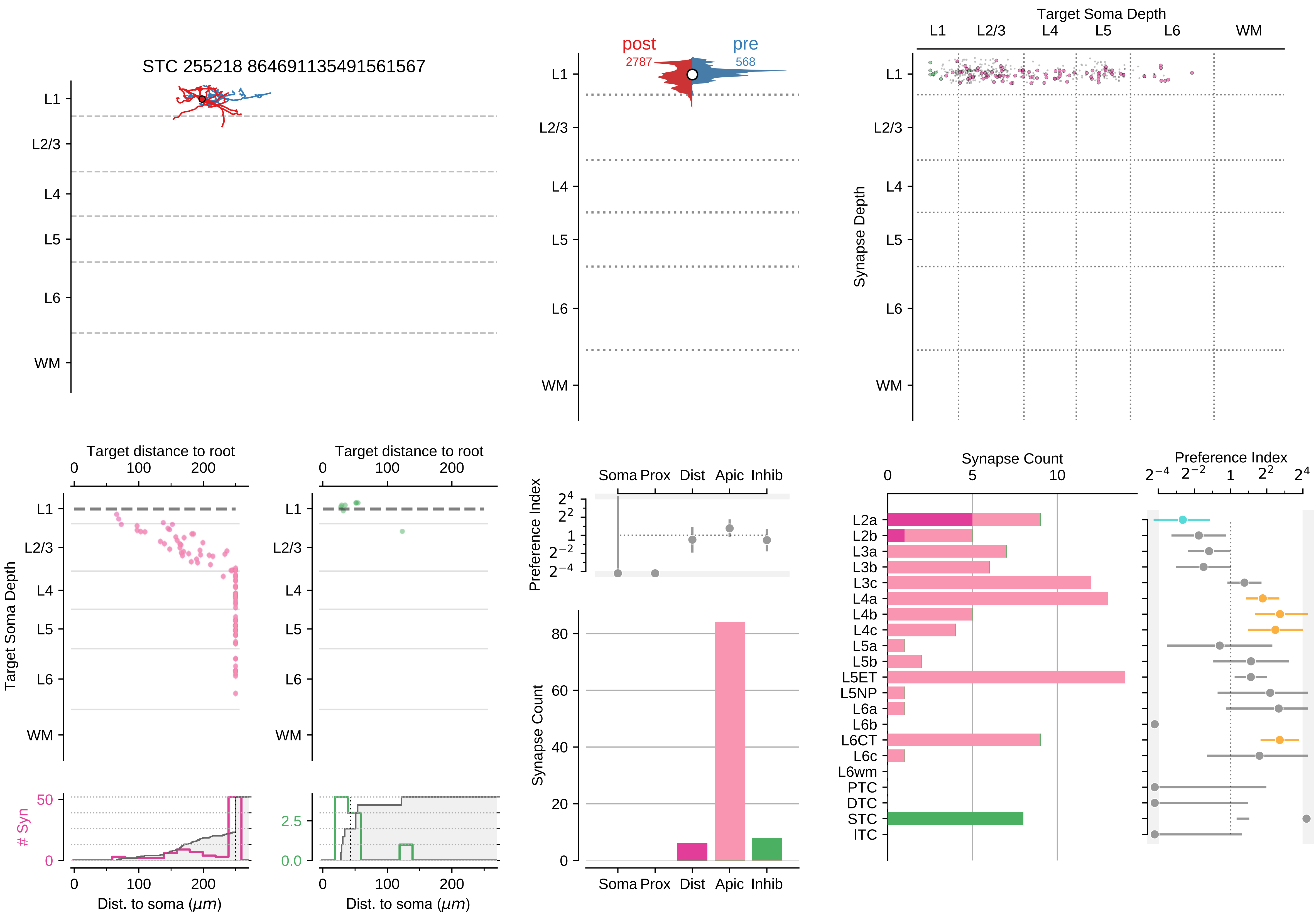

STC 255218 864691135491561567
L1
L2/3
L4
L5
L6
WM
post
2787
pre
568
Target Soma Depth
Synapse Depth
Target distance to root
Target Soma Depth
Dist. to soma (μm)
Syn
Preference Index
Soma Prox Dist Apic Inhib
Synapse Count
L2a
L2b
L3a
L3b
L3c
L4a
L4b
L4c
L5a
L5b
L5ET
L5NP
L6a
L6b
L6CT
L6c
L6wm
PTC
DTC
STC
ITC

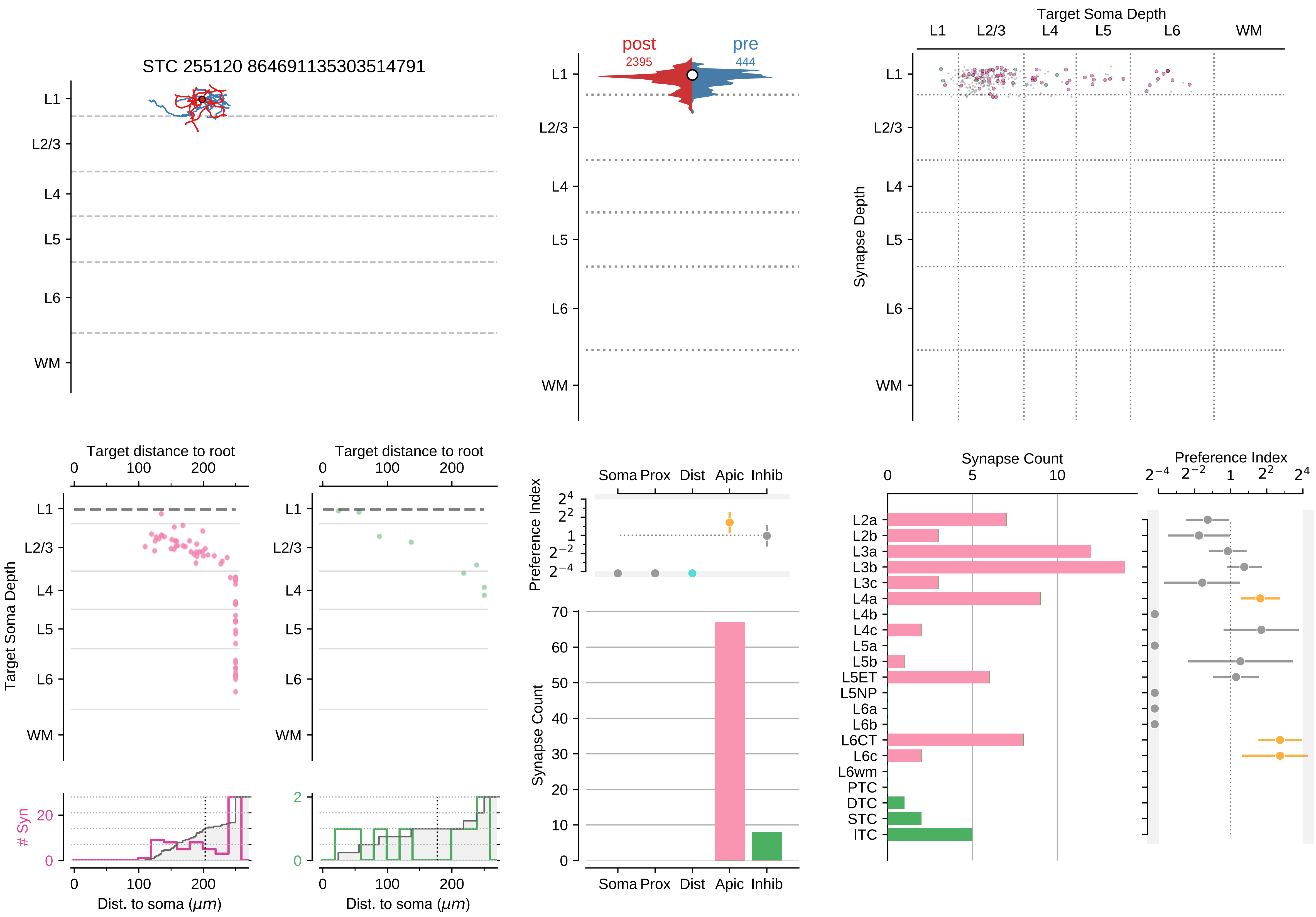

STC 255120 864691135303514791
L1
L2/3
L4
L5
L6
WM
post
2395
pre
444
Target Soma Depth
Synapse Depth
Target distance to root
Target Soma Depth
Syn
Dist. to soma (μm)
Soma Prox Dist Apic Inhib
Preference Index
Synapse Count
L2a
L2b
L3a
L3b
L3c
L4a
L4b
L4c
L5a
L5b
L5ET
L5NP
L6a
L6b
L6CT
L6c
L6wm
PTC
DTC
STC
ITC

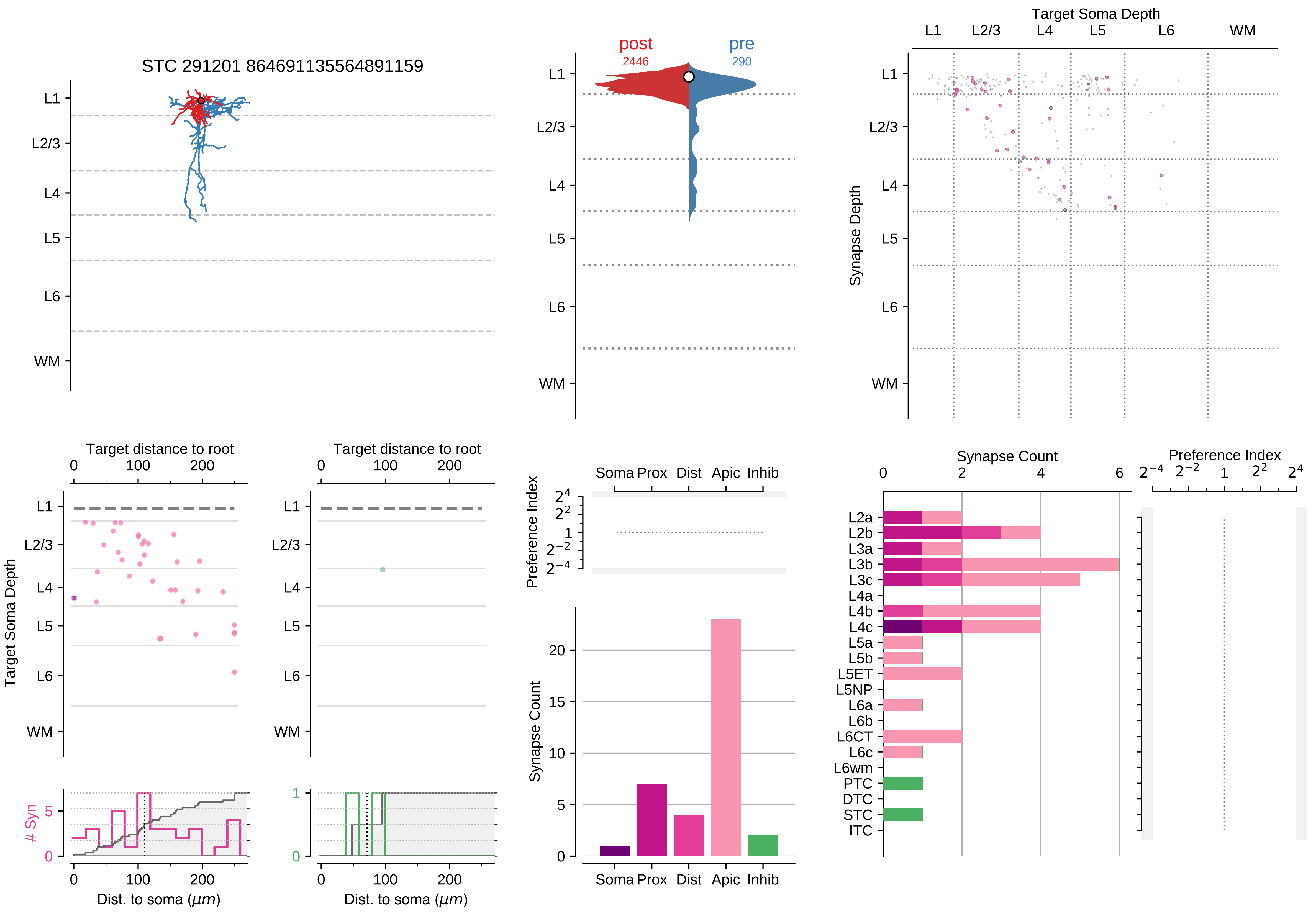
STC 291201 864691135564891159
L1
L2/3
L4
L5
L6
WM
post
2446
pre
290
Target Soma Depth
Synapse Depth
Target distance to root
0
100
200
Target Soma Depth
Syn
Dist. to soma (μm)
Preference Index
Soma Prox Dist Apic Inhib
Synapse Count
Synapse Count
Preference Index
L2a
L2b
L3a
L3b
L3c
L4a
L4b
L4c
L5a
L5b
L5ET
L5NP
L6a
L6b
L6CT
L6c
L6wm
PTC
DTC
STC
ITC

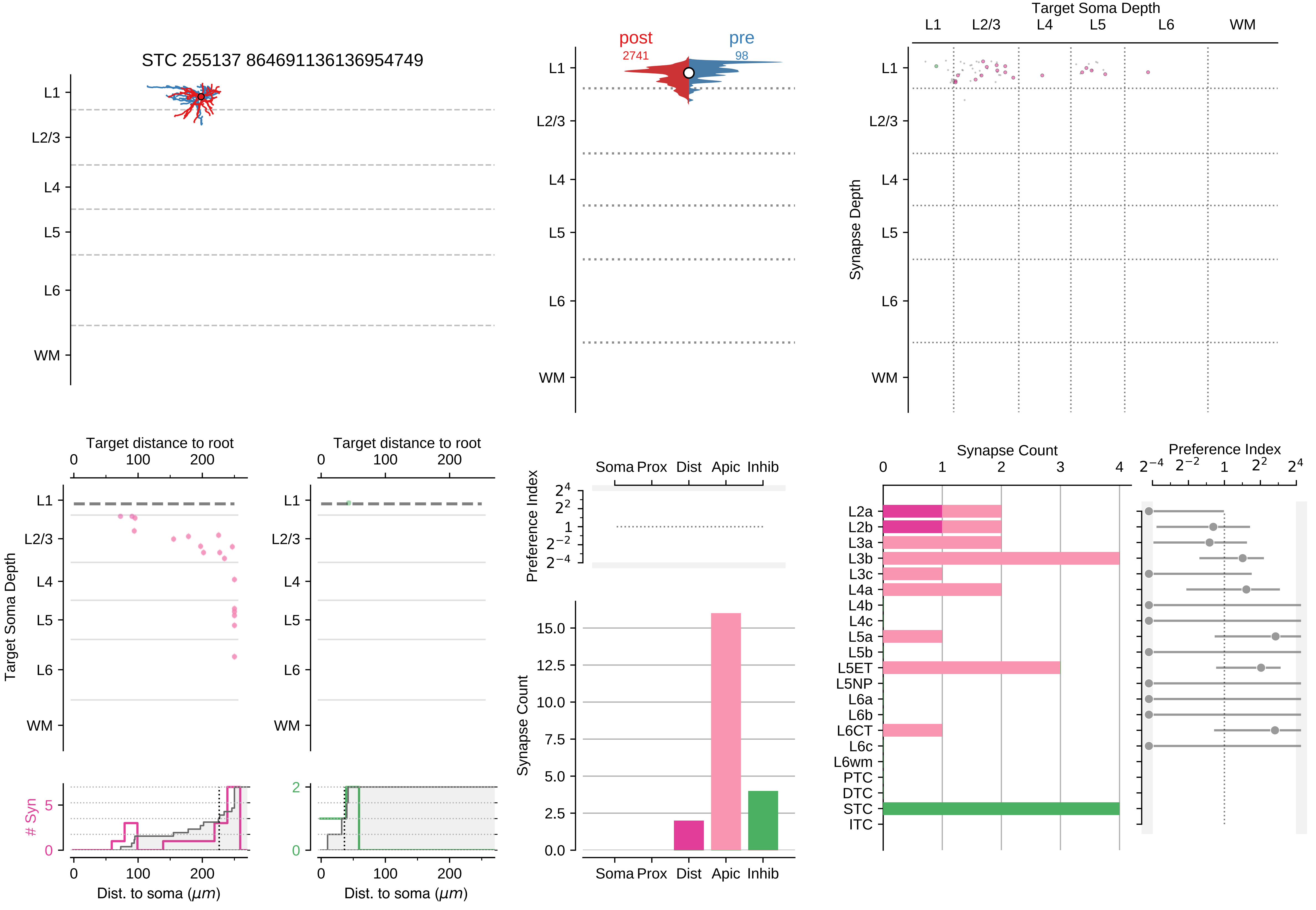

STC 255137 864691136136954749
L1
L2/3
L4
L5
L6
WM
post
2741
pre
98
Target Soma Depth
Synapse Depth
Target distance to root
Target Soma Depth
Dist. to soma (μm)
Syn
Soma Prox
Dist
Apic
Inhib
Preference Index
Synapse Count
L2a
L2b
L3a
L3b
L3c
L4a
L4b
L4c
L5a
L5b
L5ET
L5NP
L6a
L6b
L6CT
L6c
L6wm
PTC
DTC
STC
ITC

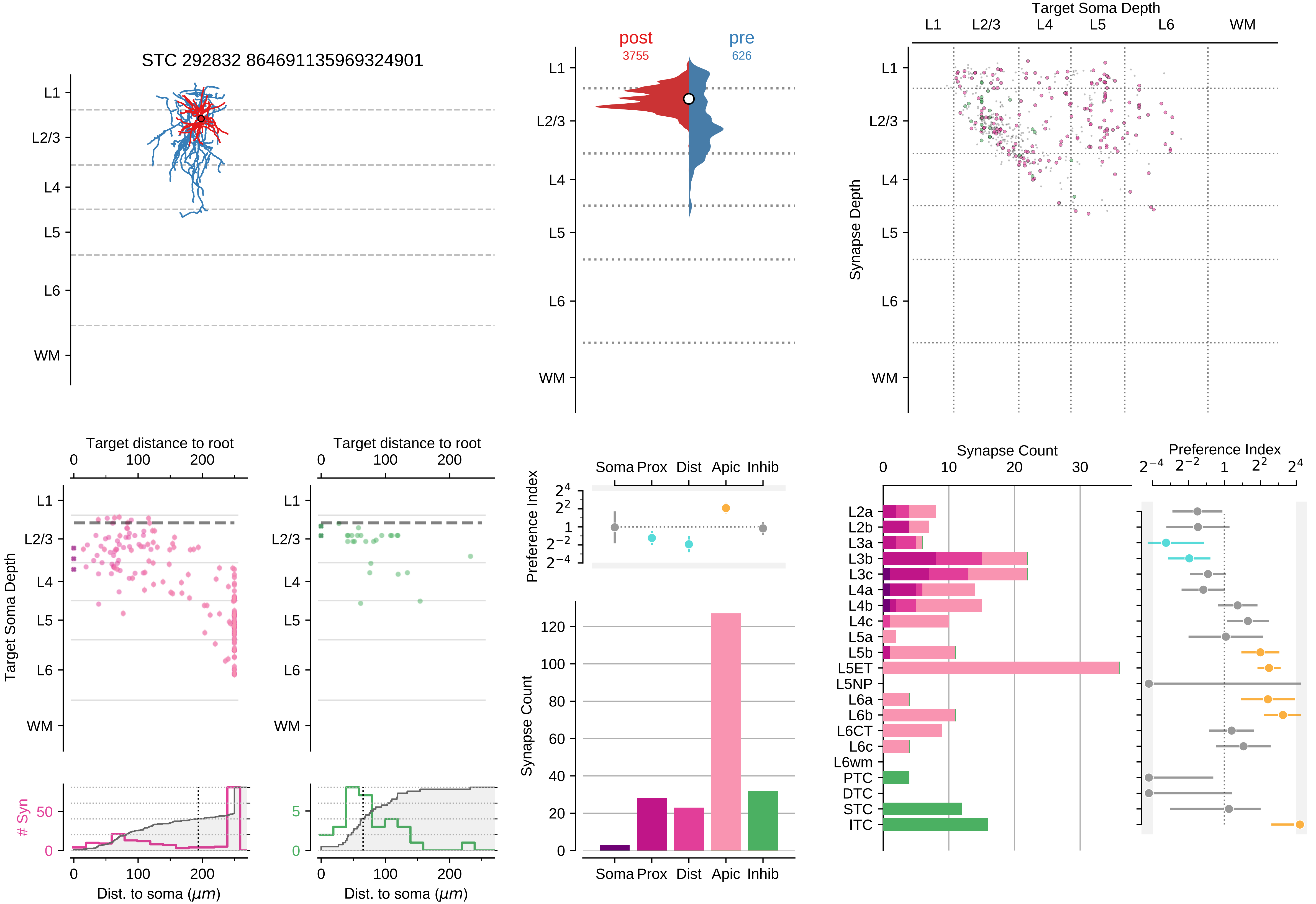

STC 292832 864691135969324901
post
3755
pre
626
Target Soma Depth
Synapse Depth
L1
L2/3
L4
L5
L6
WM
Target distance to root
Dist. to soma (μm)
Syn
Preference Index
Soma
Prox
Dist
Apic
Inhib
Synapse Count
L2a
L2b
L3a
L3b
L3c
L4a
L4b
L4c
L5a
L5b
L5ET
L5NP
L6a
L6b
L6CT
L6c
L6wm
PTC
DTC
STC
ITC

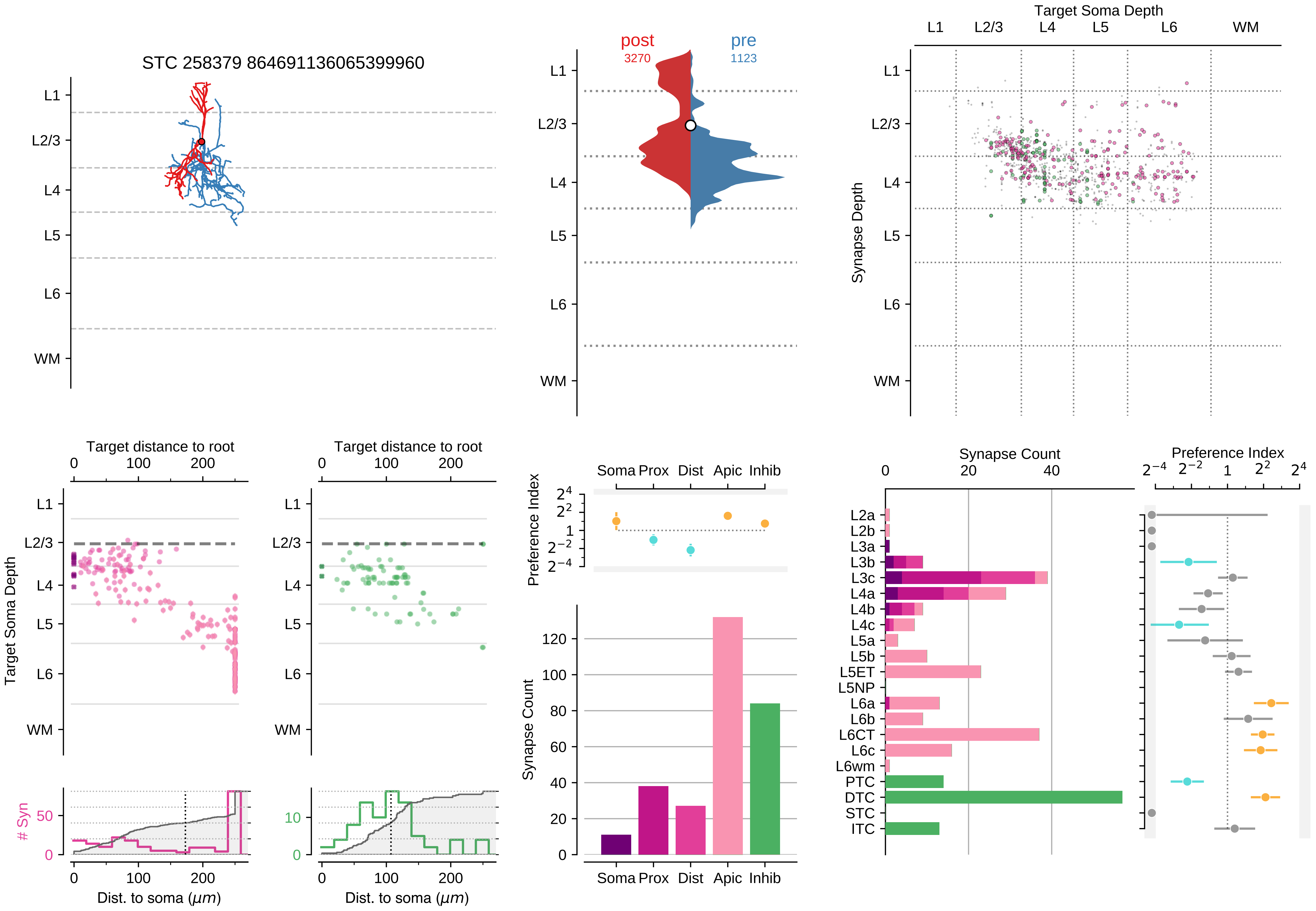
STC 258379 864691136065399960
L1
L2/3
L4
L5
L6
WM
post
3270
pre
1123
Target Soma Depth
Synapse Depth
Target distance to root
Target Soma Depth
Syn
Dist. to soma (μm)
Soma Prox Dist Apic Inhib
Preference Index
Synapse Count
Synapse Count
Preference Index
L2a
L2b
L3a
L3b
L3c
L4a
L4b
L4c
L5a
L5b
L5ET
L5NP
L6a
L6b
L6CT
L6c
L6wm
PTC
DTC
STC
ITC

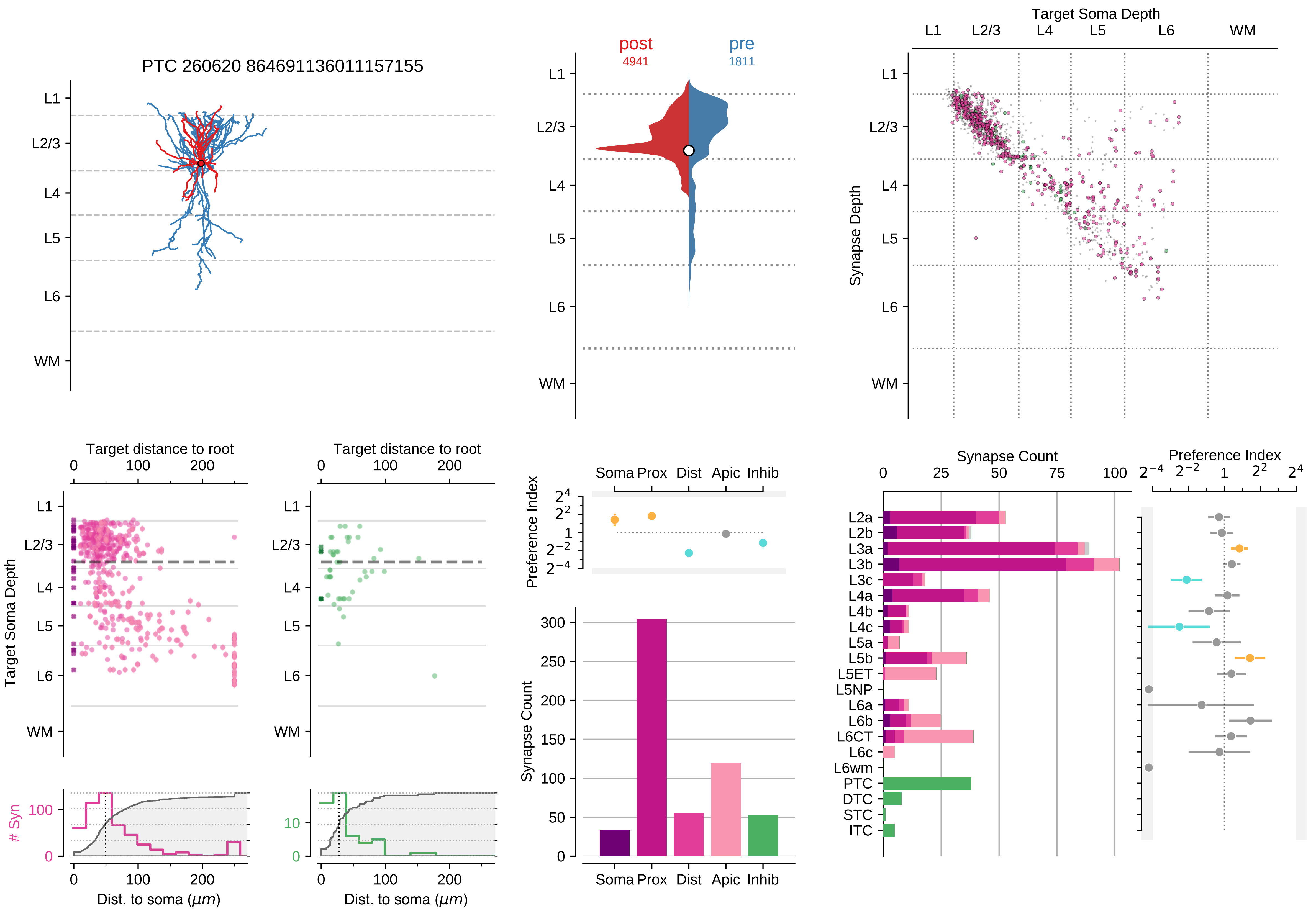

PTC 260620 864691136011157155
L1
L2/3
L4
L5
L6
WM
post
4941
pre
1811
Target Soma Depth
Synapse Depth
Target distance to root
Target Soma Depth
Syn
Dist. to soma (μm)
Preference Index
Soma
Prox
Dist
Apic
Inhib
Synapse Count
L2a
L2b
L3a
L3b
L3c
L4a
L4b
L4c
L5a
L5b
L5ET
L5NP
L6a
L6b
L6CT
L6c
L6wm
PTC
DTC
STC
ITC

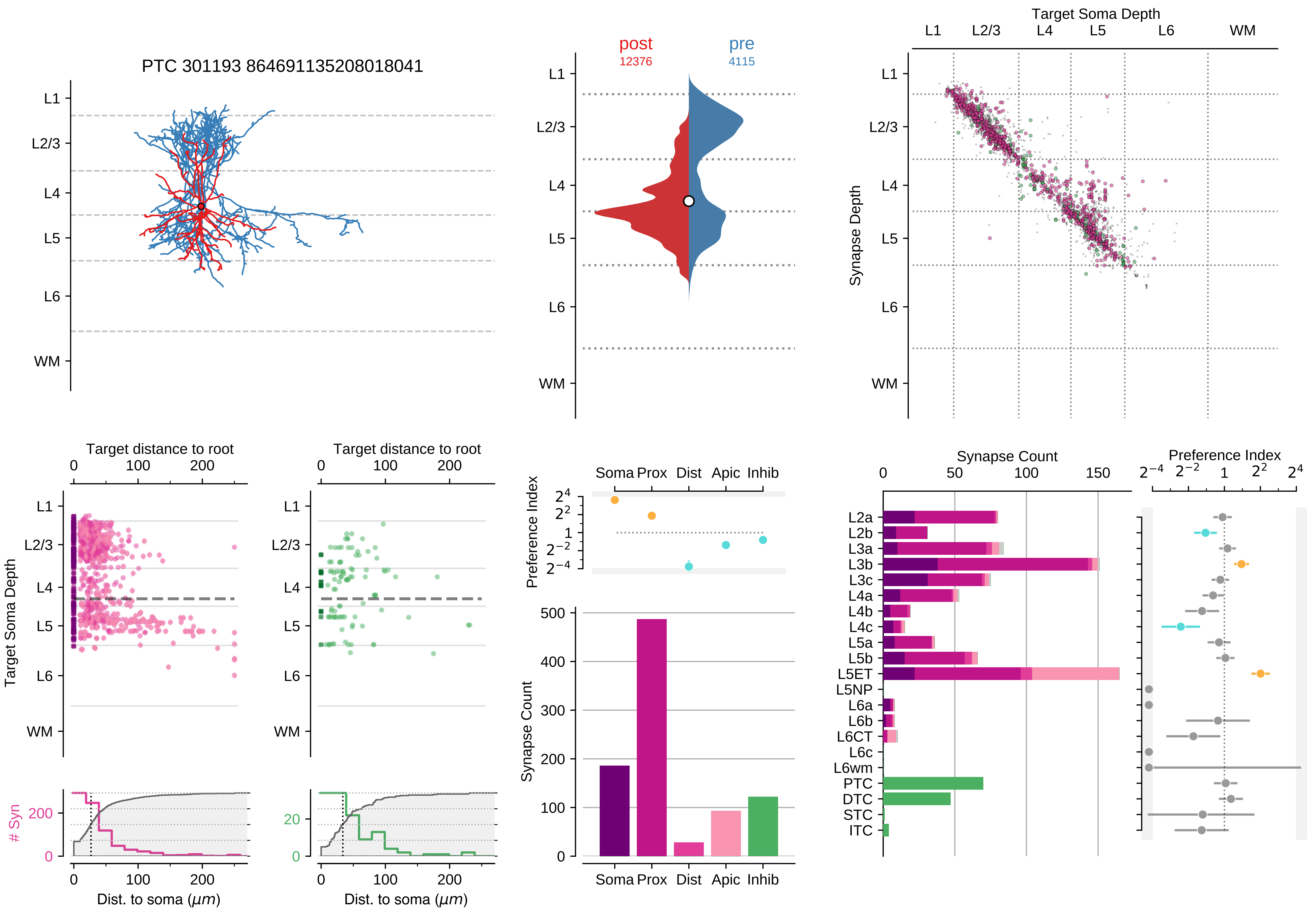

PTC 301193 864691135208018041
L1
L2/3
L4
L5
L6
WM
post
12376
pre
4115
Target Soma Depth
Synapse Depth
Target distance to root
Target Soma Depth
Syn
Dist. to soma (μm)
Preference Index
Soma Prox Dist Apic Inhib
Synapse Count
L2a
L2b
L3a
L3b
L3c
L4a
L4b
L4c
L5a
L5b
L5ET
L5NP
L6a
L6b
L6CT
L6c
L6wm
PTC
DTC
STC
ITC

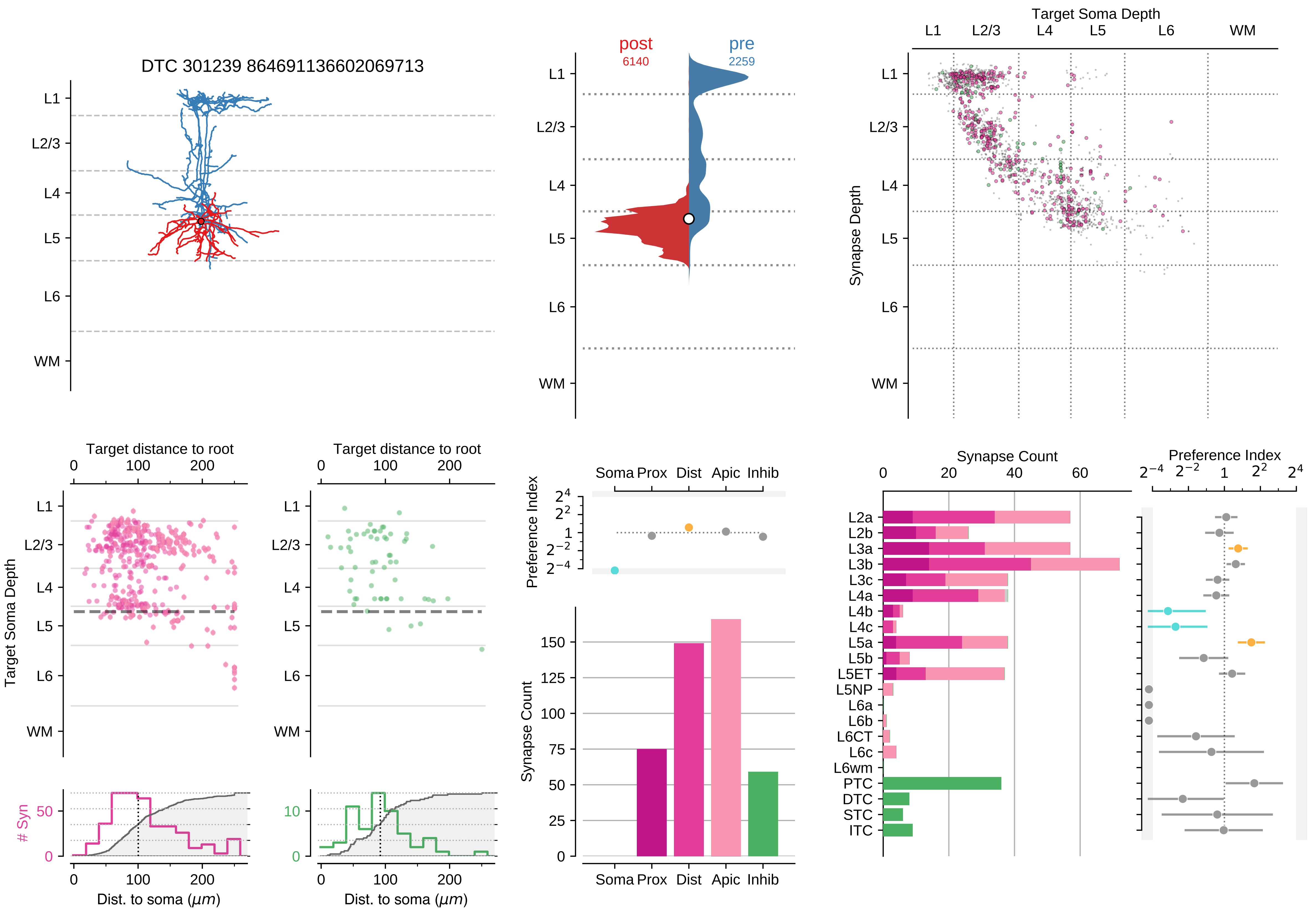
DTC 301239 864691136602069713
L1
L2/3
L4
L5
L6
WM
post
6140
pre
2259
Target Soma Depth
Synapse Depth
Target distance to root
Target Soma Depth
Syn
Dist. to soma (μm)
Preference Index
Soma Prox Dist Apic Inhib
Synapse Count
L2a
L2b
L3a
L3b
L3c
L4a
L4b
L4c
L5a
L5b
L5ET
L5NP
L6a
L6b
L6CT
L6c
L6wm
PTC
DTC
STC
ITC

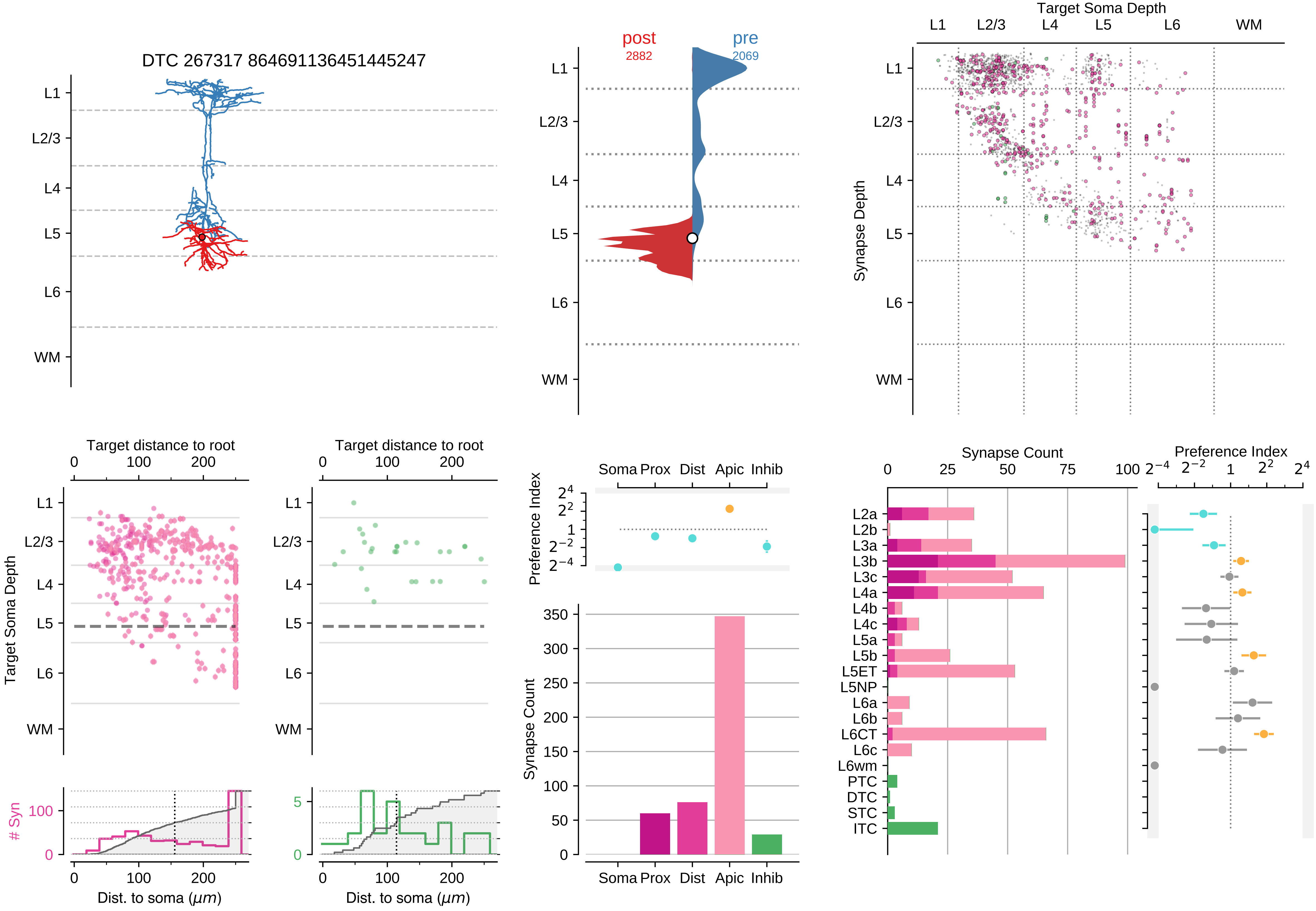
DTC 267317 864691136451445247
post
2882
pre
2069
Target Soma Depth
L1
L2/3
L4
L5
L6
WM
Synapse Depth
Target distance to root
Target Soma Depth
Syn
Dist. to soma (μm)
Preference Index
Soma Prox Dist Apic Inhib
Synapse Count
L2a
L2b
L3a
L3b
L3c
L4a
L4b
L4c
L5a
L5b
L5ET
L5NP
L6a
L6b
L6CT
L6c
L6wm
PTC
DTC
STC
ITC

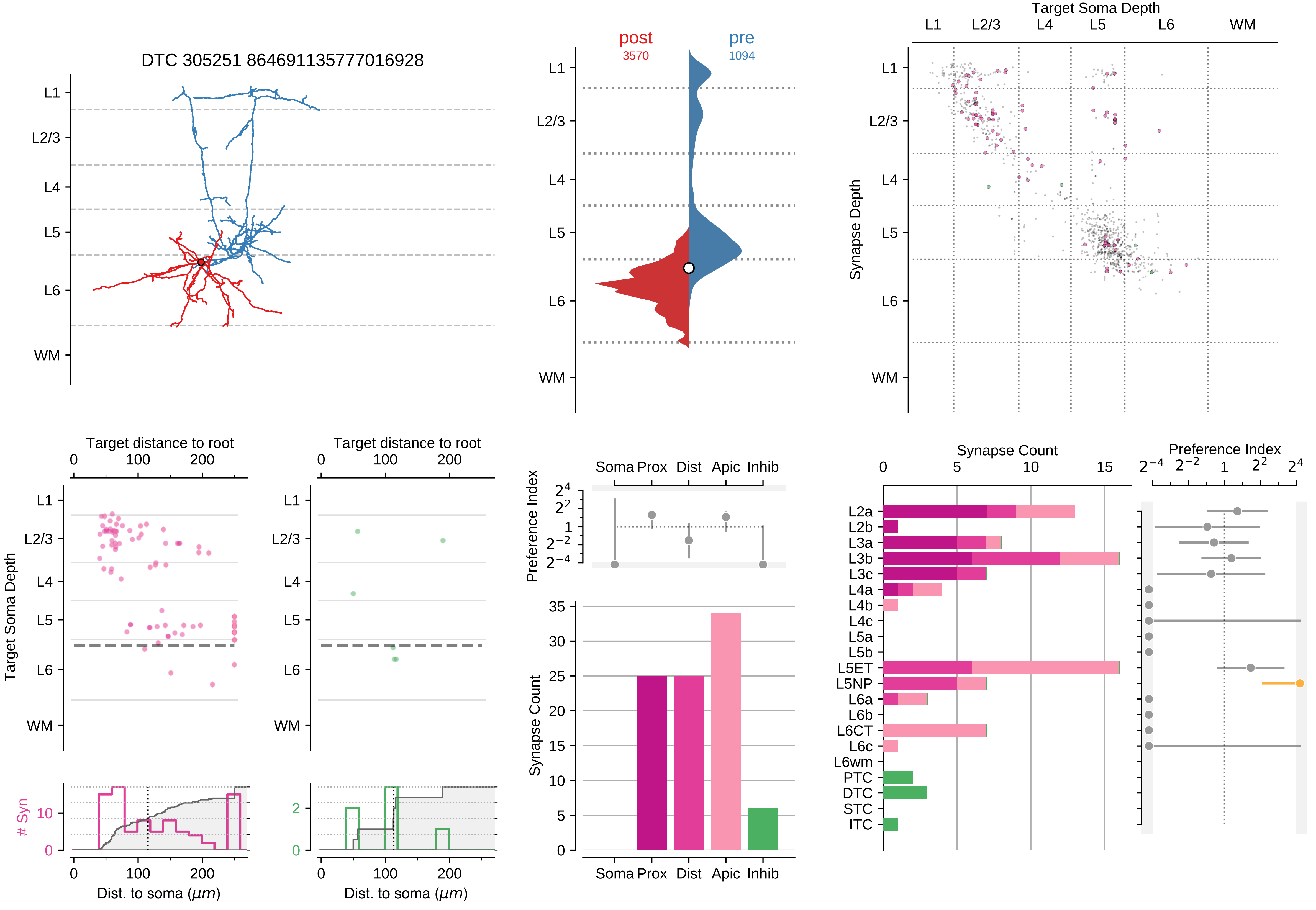

DTC 305251 864691135777016928
L1
L2/3
L4
L5
L6
WM
post
3570
pre
1094
Target Soma Depth
Synapse Depth
Target distance to root
0
100
200
Target Soma Depth
Syn
Dist. to soma (μm)
Soma Prox Dist Apic Inhib
Preference Index
Synapse Count
L2a
L2b
L3a
L3b
L3c
L4a
L4b
L4c
L5a
L5b
L5ET
L5NP
L6a
L6b
L6CT
L6c
L6wm
PTC
DTC
STC
ITC

Group 7

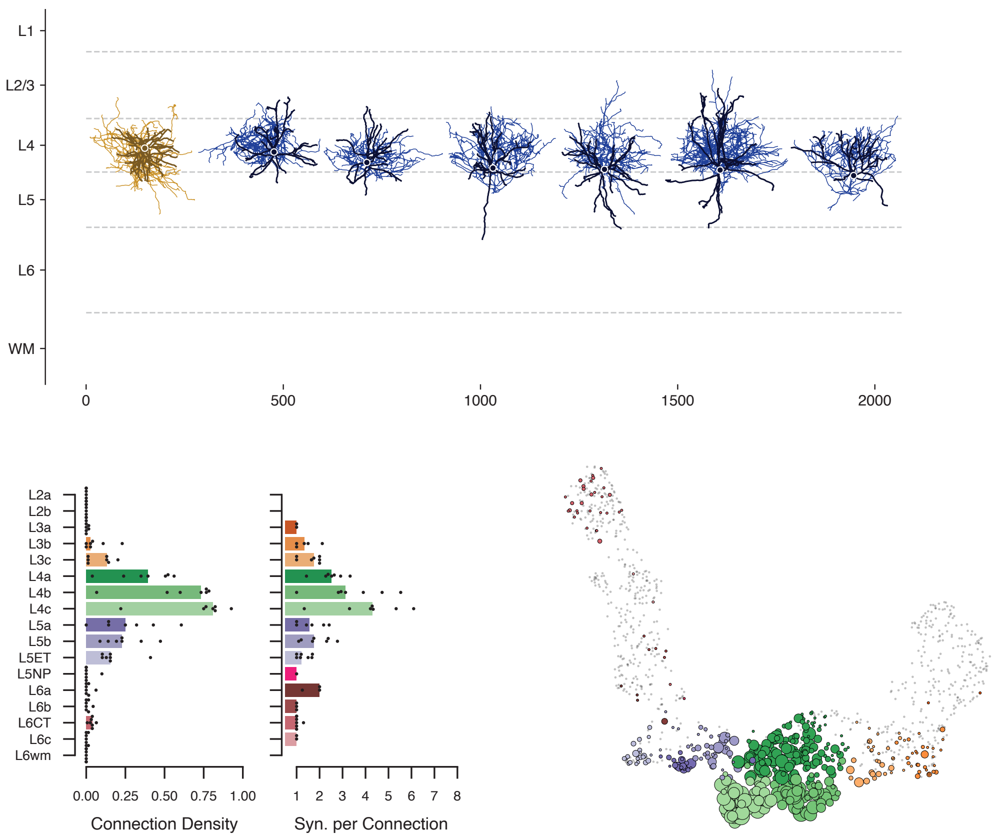
L1
L2/3
L4
L5
L6
WM
0
500
1000
1500
2000
L2a
L2b
L3a
L3b
L3c
L4a
L4b
L4c
L5a
L5b
L5ET
L5NP
L6a
L6b
L6CT
L6c
L6wm
0.00 0.25 0.50 0.75 1.00
Connection Density
1 2 3 4 5 6 7 8
Syn. per Connection

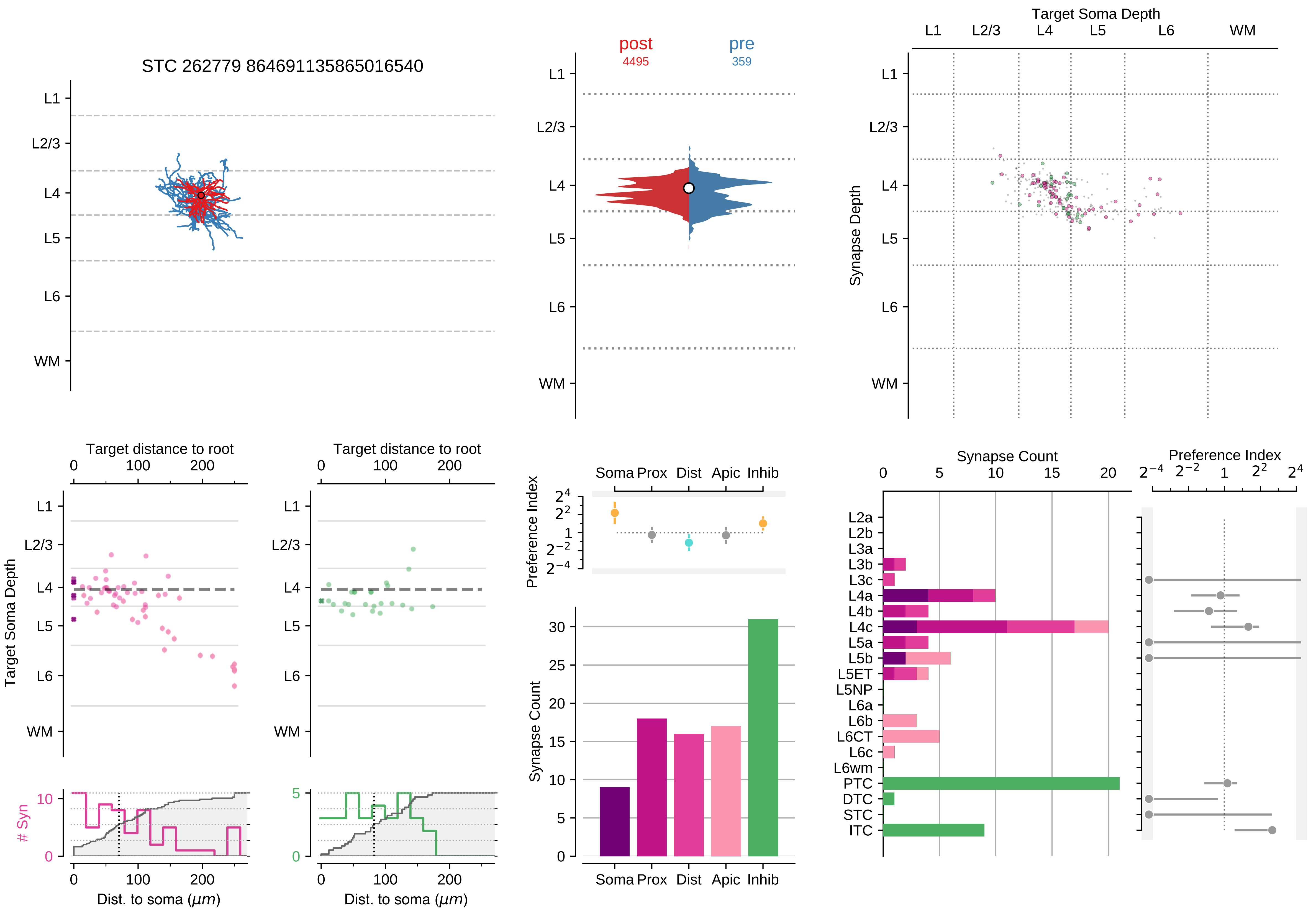

STC 262779 864691135865016540
post
4495
pre
359
Target Soma Depth
Synapse Depth
L1
L2/3
L4
L5
L6
WM
Target distance to root
Target Soma Depth
Syn
Dist. to soma (μm)
Soma Prox Dist Apic Inhib
Preference Index
Synapse Count
L2a
L2b
L3a
L3b
L3c
L4a
L4b
L4c
L5a
L5b
L5ET
L5NP
L6a
L6b
L6CT
L6c
L6wm
PTC
DTC
STC
ITC

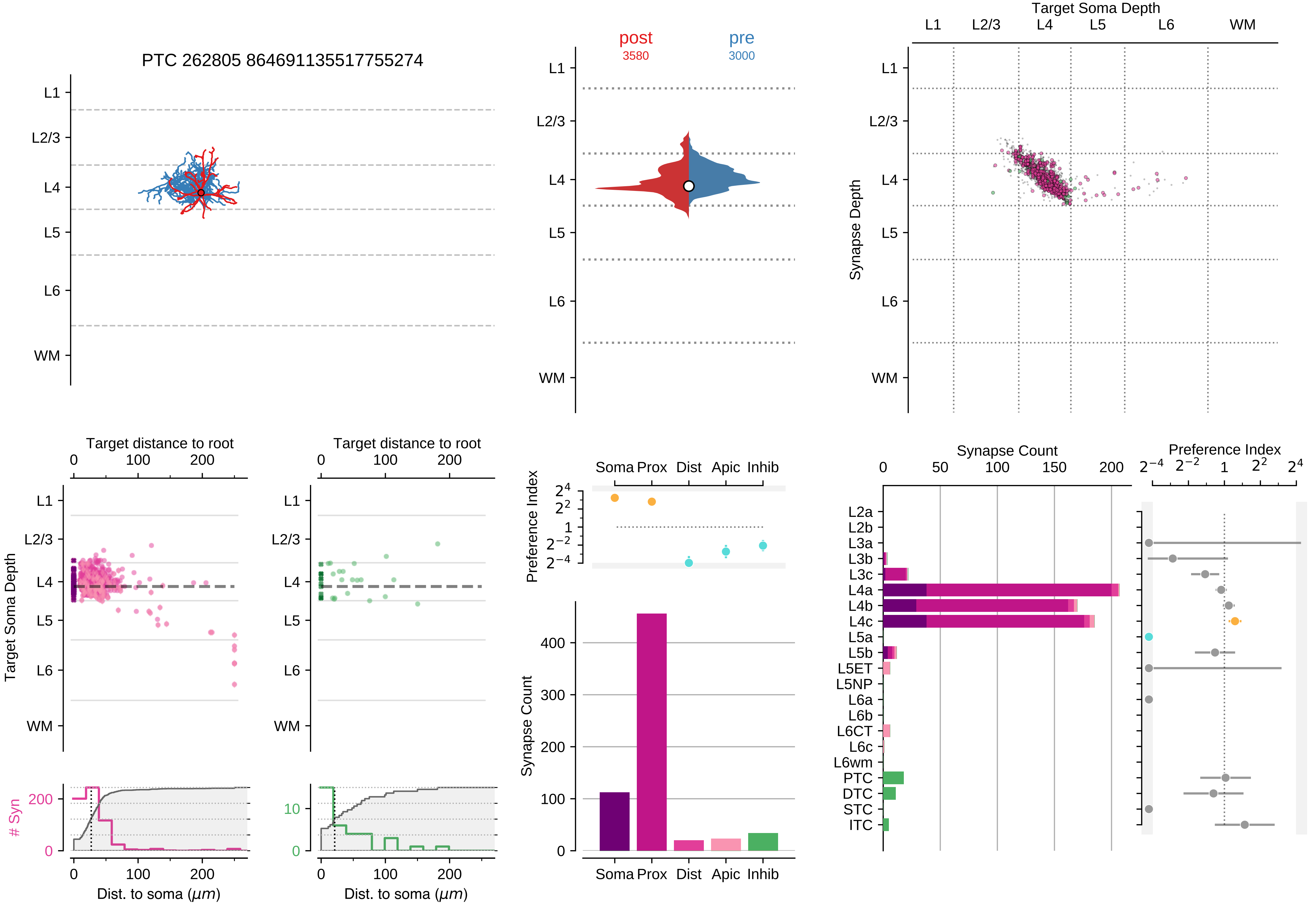
PTC 262805 864691135517755274
L1
L2/3
L4
L5
L6
WM
post
3580
pre
3000
Target Soma Depth
Synapse Depth
Target distance to root
Target Soma Depth
Syn
Dist. to soma (μm)
Preference Index
Soma Prox Dist Apic Inhib
Synapse Count
L2a
L2b
L3a
L3b
L3c
L4a
L4b
L4c
L5a
L5b
L5ET
L5NP
L6a
L6b
L6CT
L6c
L6wm
PTC
DTC
STC
ITC

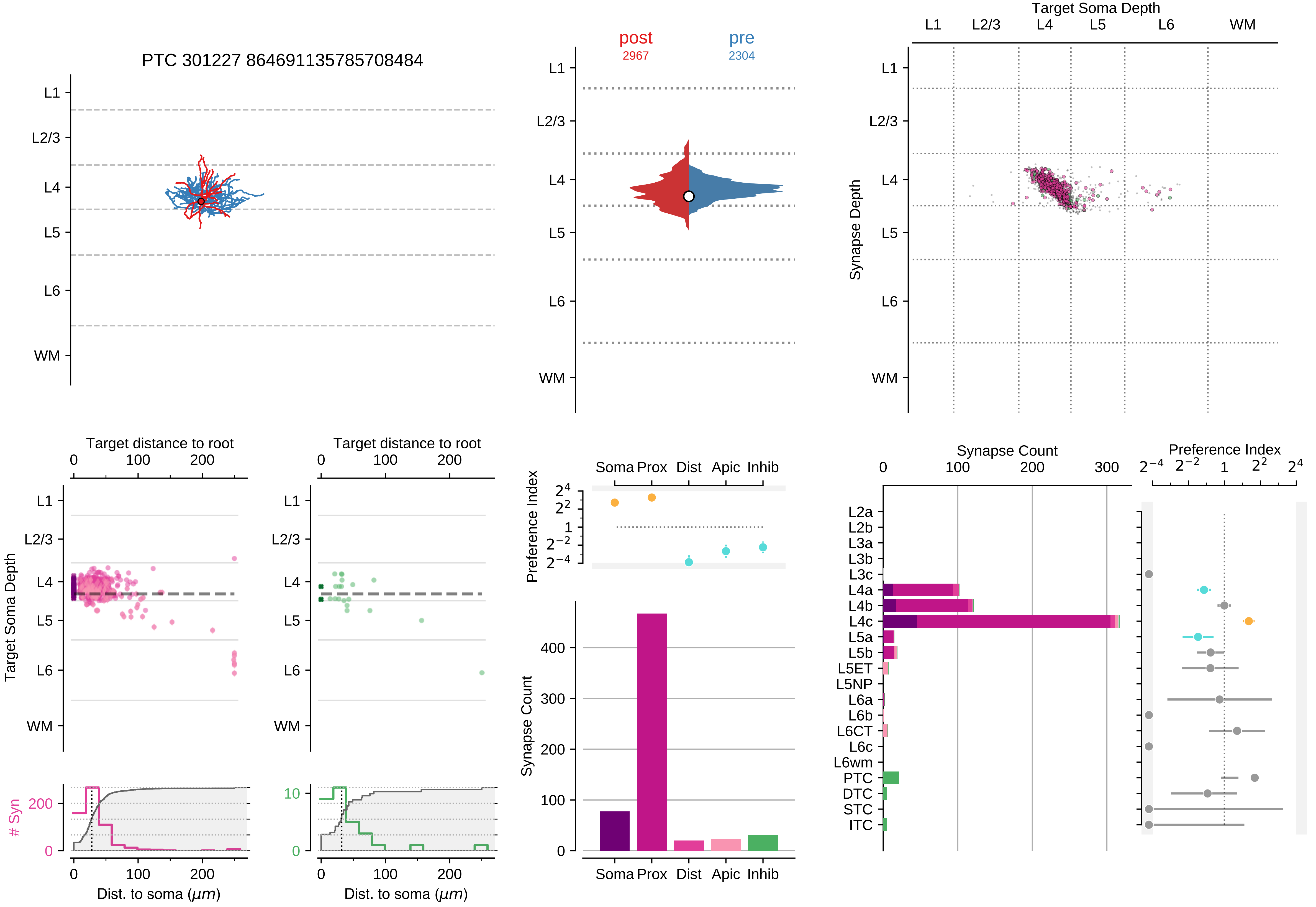

PTC 301227 864691135785708484
L1
L2/3
L4
L5
L6
WM
post
2967
pre
2304
Target Soma Depth
Synapse Depth
Target distance to root
Target Soma Depth
Syn
Dist. to soma (μm)
Preference Index
Soma Prox Dist Apic Inhib
Synapse Count
L2a
L2b
L3a
L3b
L3c
L4a
L4b
L4c
L5a
L5b
L5ET
L5NP
L6a
L6b
L6CT
L6c
L6wm
PTC
DTC
STC
ITC

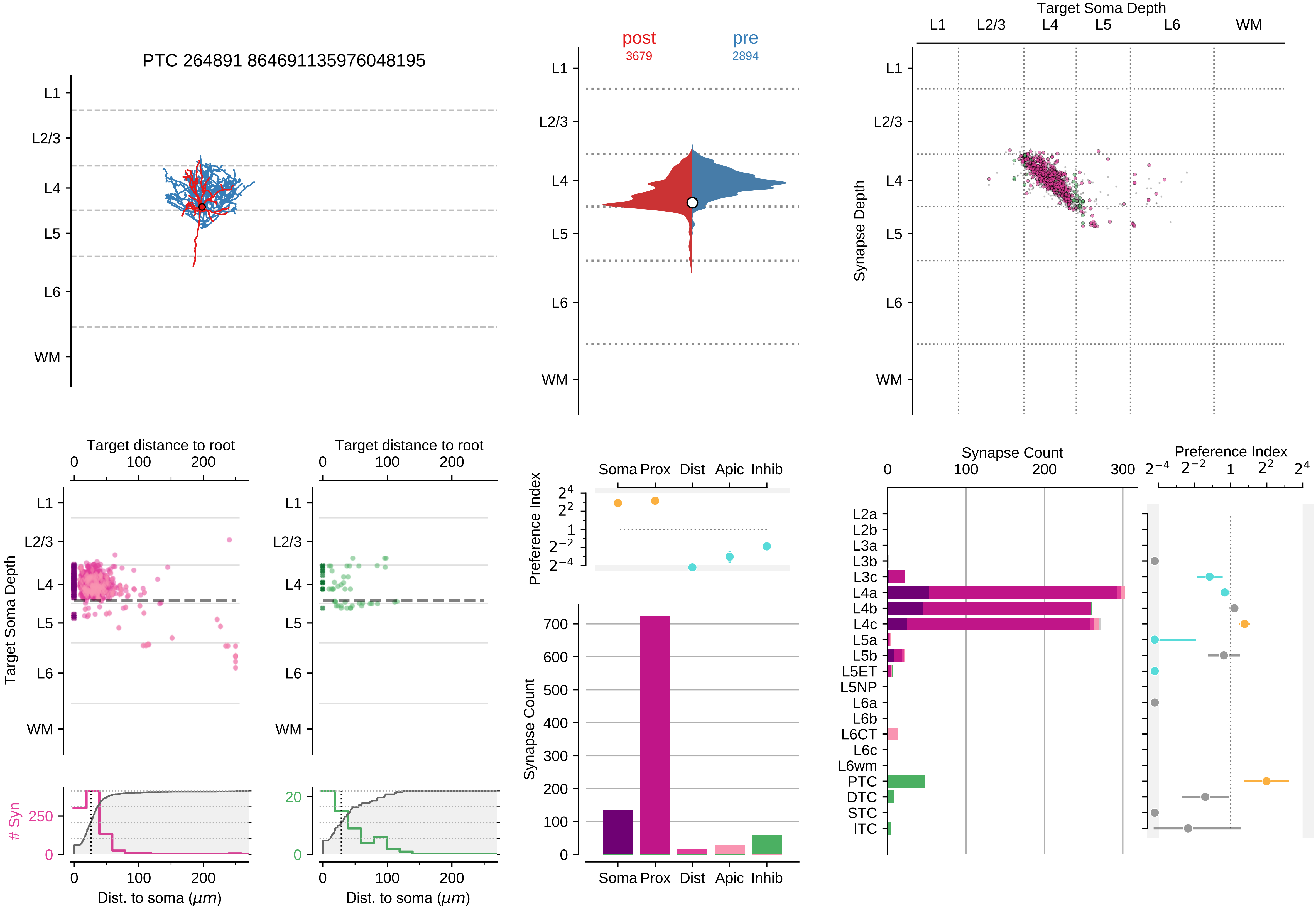
PTC 264891 864691135976048195
L1
L2/3
L4
L5
L6
WM
post
3679
pre
2894
Target Soma Depth
Synapse Depth
Target distance to root
Target Soma Depth
Syn
Dist. to soma (μm)
Preference Index
Soma
Prox
Dist
Apic
Inhib
Synapse Count
L2a
L2b
L3a
L3b
L3c
L4a
L4b
L4c
L5a
L5b
L5ET
L5NP
L6a
L6b
L6CT
L6c
L6wm
PTC
DTC
STC
ITC

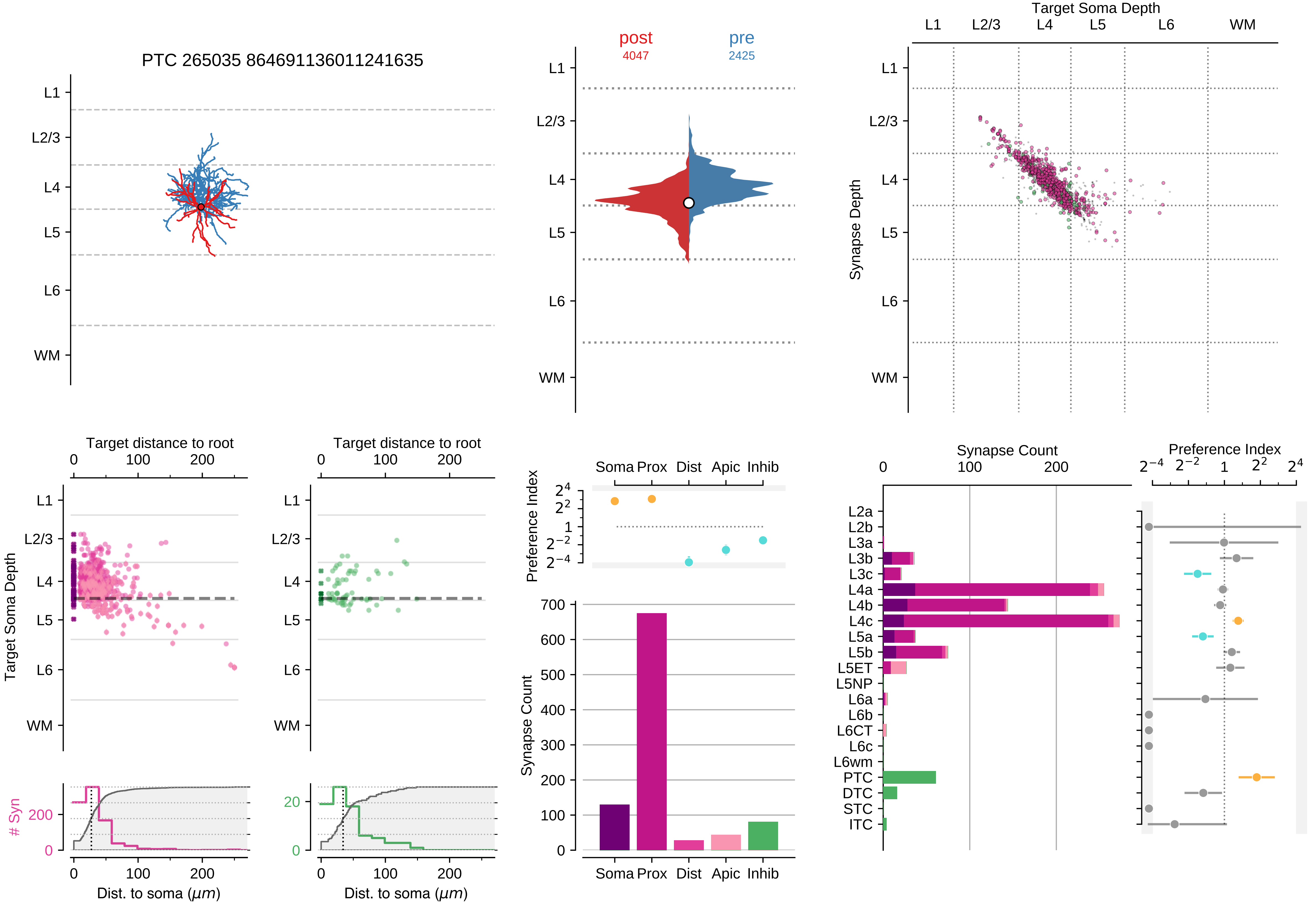

PTC 265035 864691136011241635
L1
L2/3
L4
L5
L6
WM
post
4047
pre
2425
Target Soma Depth
Synapse Depth
Target distance to root
Target Soma Depth
Syn
Dist. to soma (μm)
Soma
Prox
Dist
Apic
Inhib
Preference Index
Synapse Count
L2a
L2b
L3a
L3b
L3c
L4a
L4b
L4c
L5a
L5b
L5ET
L5NP
L6a
L6b
L6CT
L6c
L6wm
PTC
DTC
STC
ITC

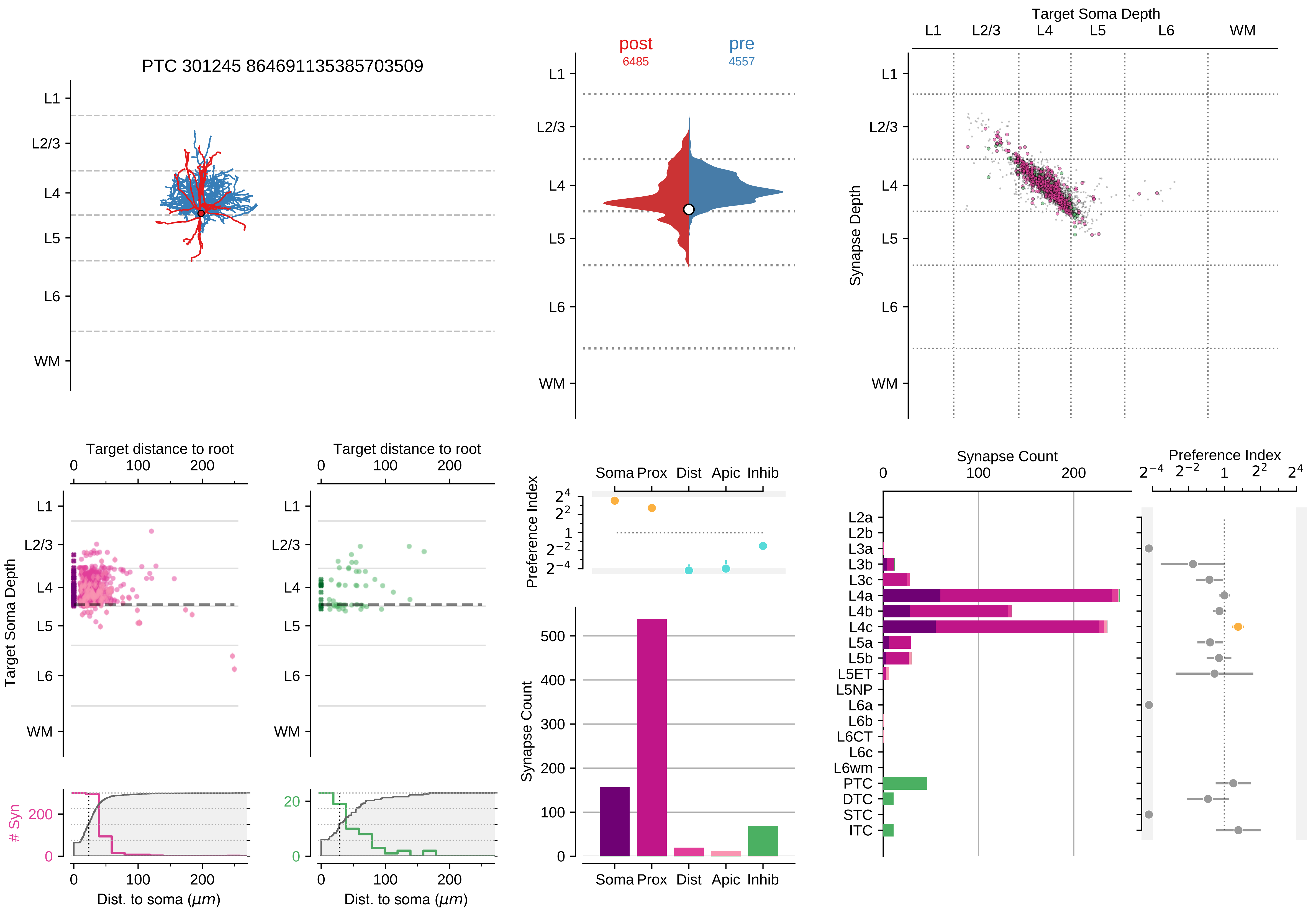

PTC 301245 864691135385703509
L1
L2/3
L4
L5
L6
WM
post
6485
pre
4557
Target Soma Depth
Synapse Depth
Target distance to root
Target Soma Depth
Syn
Dist. to soma (μm)
Preference Index
Soma
Prox
Dist
Apic
Inhib
Synapse Count
L2a
L2b
L3a
L3b
L3c
L4a
L4b
L4c
L5a
L5b
L5ET
L5NP
L6a
L6b
L6CT
L6c
L6wm
PTC
DTC
STC
ITC

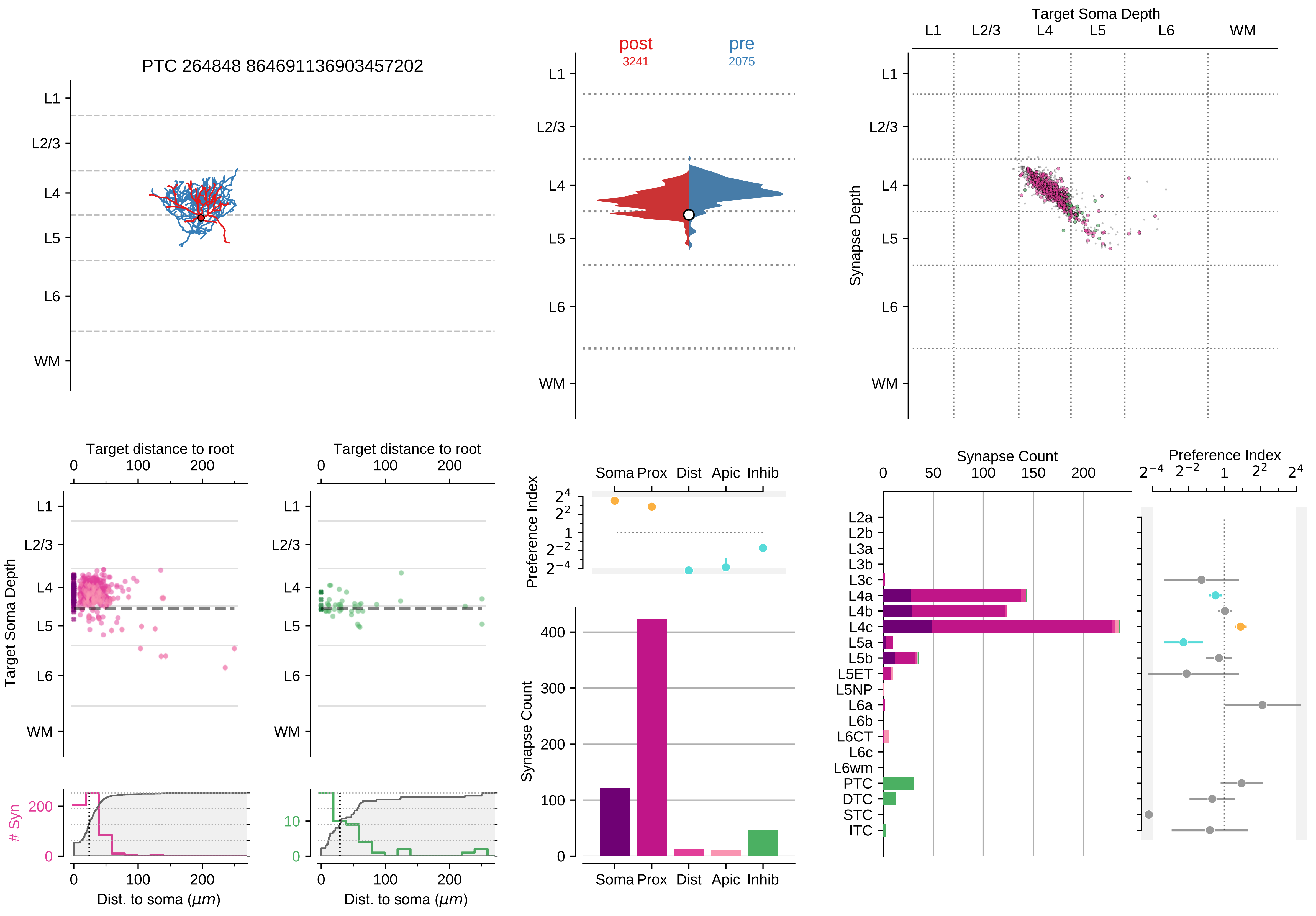

PTC 264848 864691136903457202
L1
L2/3
L4
L5
L6
WM
post
3241
pre
2075
Target Soma Depth
Synapse Depth
Target distance to root
Target Soma Depth
Syn
Dist. to soma (μm)
Preference Index
Soma Prox Dist Apic Inhib
Synapse Count
L2a
L2b
L3a
L3b
L3c
L4a
L4b
L4c
L5a
L5b
L5ET
L5NP
L6a
L6b
L6CT
L6c
L6wm
PTC
DTC
STC
ITC

Group 8

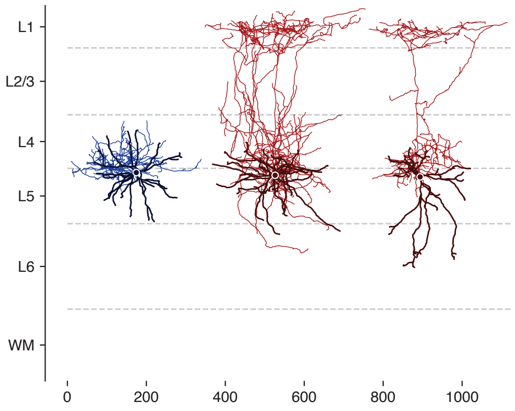

L1
L2/3
L4
L5
L6
WM
0
200
400
600
800
1000

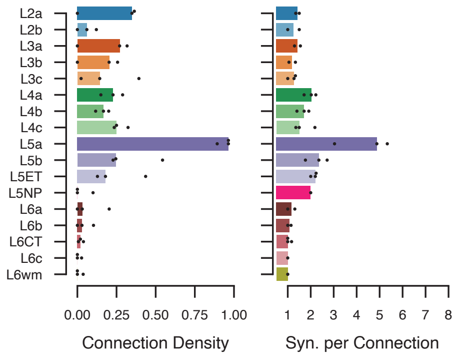

L2a
L2b
L3a
L3b
L3c
L4a
L4b
L4c
L5a
L5b
L5ET
L5NP
L6a
L6b
L6CT
L6c
L6wm
0.00 0.25 0.50 0.75 1.00
Connection Density
1 2 3 4 5 6 7 8
Syn. per Connection

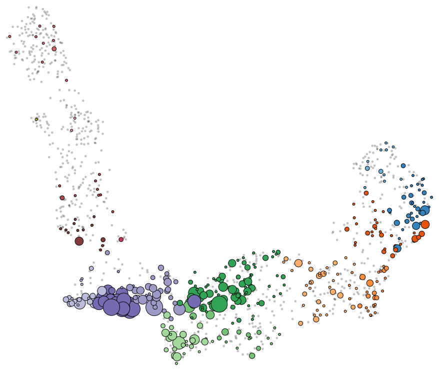

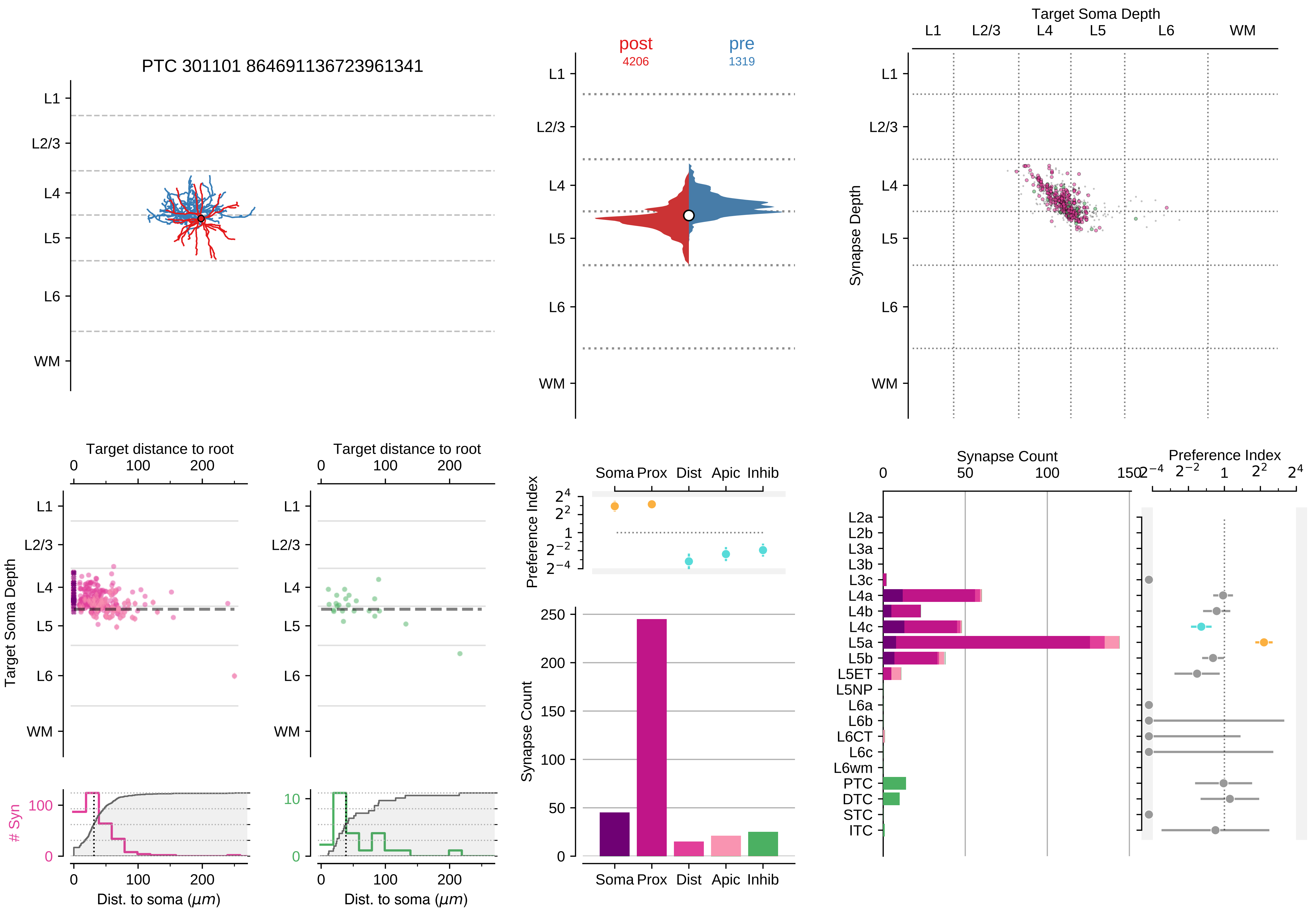

PTC 301101 864691136723961341
post
4206
pre
1319
Target Soma Depth
Synapse Depth
L1
L2/3
L4
L5
L6
WM
Target distance to root
Target Soma Depth
Syn
Dist. to soma (μm)
Preference Index
Soma
Prox
Dist
Apic
Inhib
Synapse Count
Synapse Count
Preference Index
L2a
L2b
L3a
L3b
L3c
L4a
L4b
L4c
L5a
L5b
L5ET
L5NP
L6a
L6b
L6CT
L6c
L6wm
PTC
DTC
STC
ITC

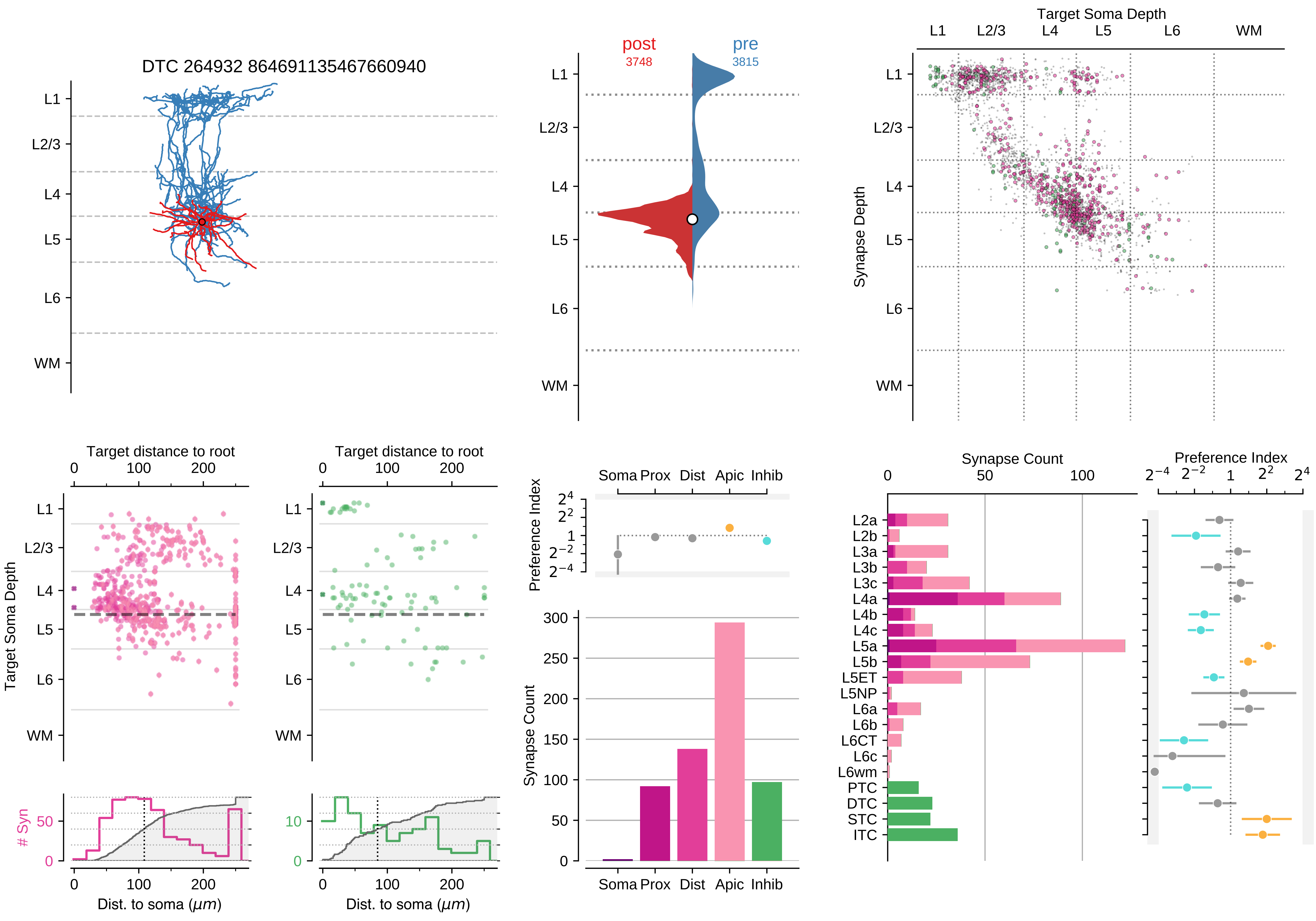

DTC 264932 864691135467660940
L1
L2/3
L4
L5
L6
WM
post
3748
pre
3815
Target Soma Depth
Synapse Depth
Target distance to root
Target Soma Depth
Syn
Dist. to soma (μm)
Preference Index
Soma Prox Dist Apic Inhib
Synapse Count
L2a
L2b
L3a
L3b
L3c
L4a
L4b
L4c
L5a
L5b
L5ET
L5NP
L6a
L6b
L6CT
L6c
L6wm
PTC
DTC
STC
ITC

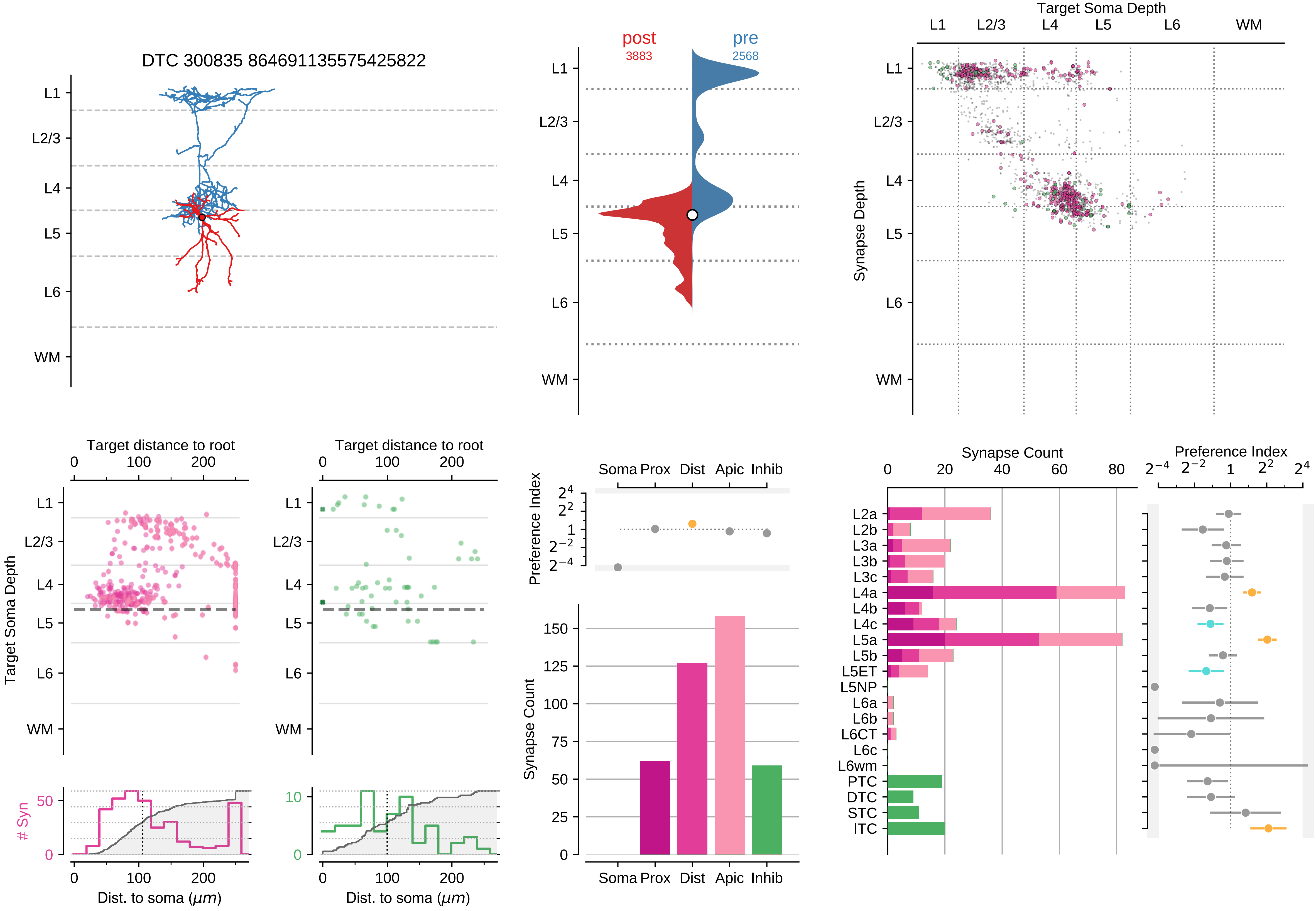

DTC 300835 864691135575425822
L1
L2/3
L4
L5
L6
WM
post
3883
pre
2568
Target Soma Depth
Synapse Depth
Target distance to root
Target Soma Depth
Syn
Dist. to soma (μm)
Preference Index
Soma Prox Dist Apic Inhib
Synapse Count
L2a
L2b
L3a
L3b
L3c
L4a
L4b
L4c
L5a
L5b
L5ET
L5NP
L6a
L6b
L6CT
L6c
L6wm
PTC
DTC
STC
ITC

Group 9

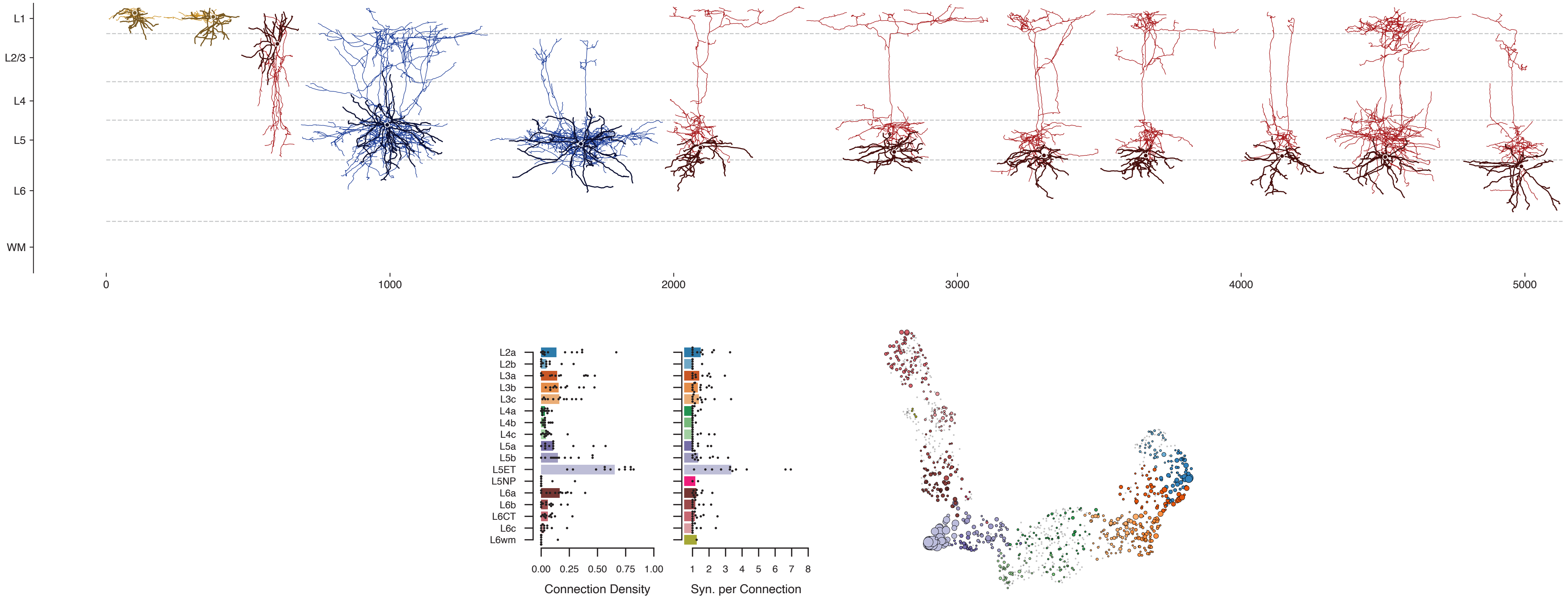
L1
L2/3
L4
L5
L6
WM
0
1000
2000
3000
4000
5000
L2a
L2b
L3a
L3b
L3c
L4a
L4b
L4c
L5a
L5b
L5ET
L5NP
L6a
L6b
L6CT
L6c
L6wm
0.00 0.25 0.50 0.75 1.00
Connection Density
1 2 3 4 5 6 7 8
Syn. per Connection

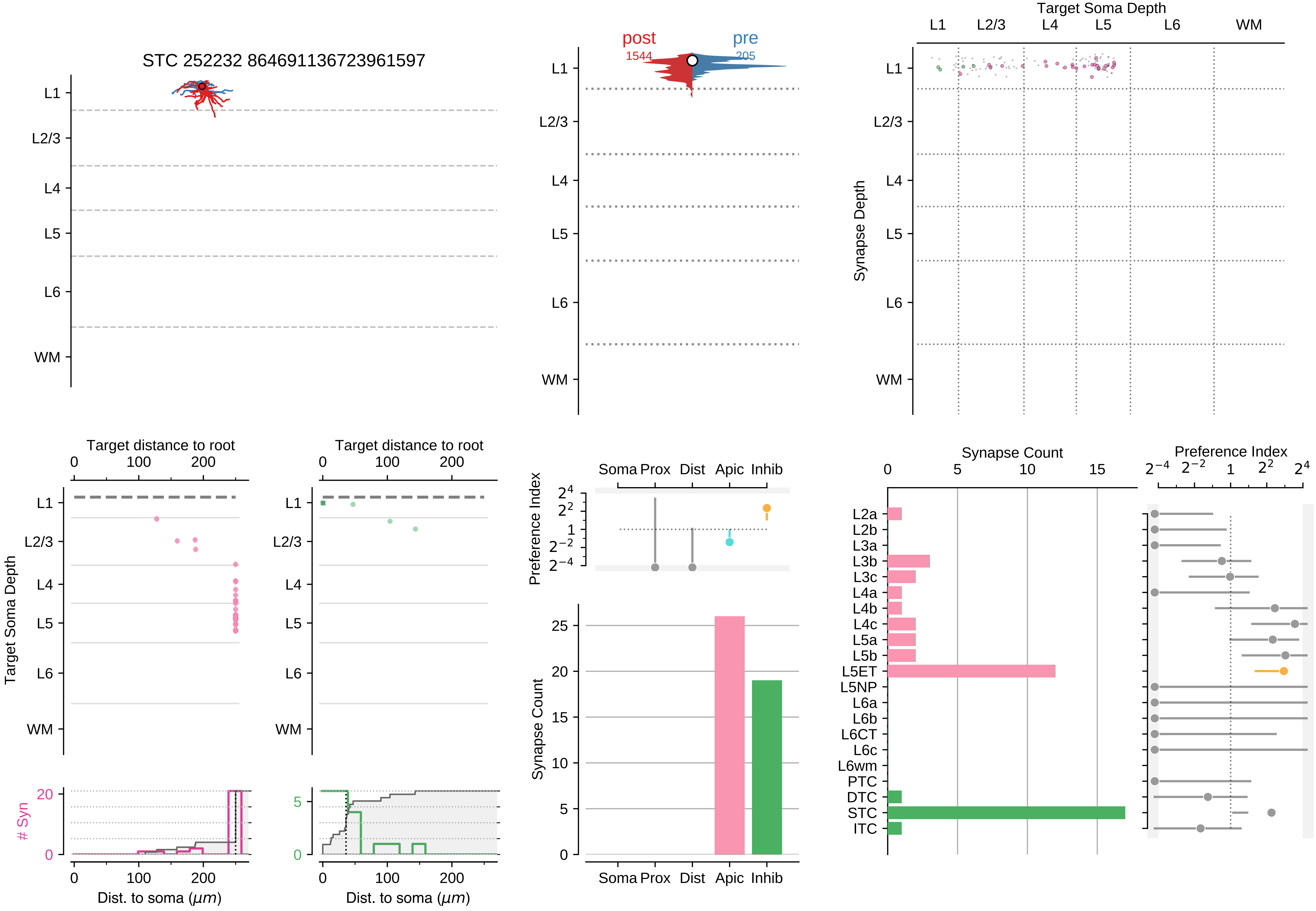
STC 252232 864691136723961597
L1
L2/3
L4
L5
L6
WM
post
1544
pre
205
Target Soma Depth
Synapse Depth
Target distance to root
Target Soma Depth
Syn
Dist. to soma (μm)
Preference Index
Soma Prox Dist Apic Inhib
Synapse Count
L2a
L2b
L3a
L3b
L3c
L4a
L4b
L4c
L5a
L5b
L5ET
L5NP
L6a
L6b
L6CT
L6c
L6wm
PTC
DTC
STC
ITC

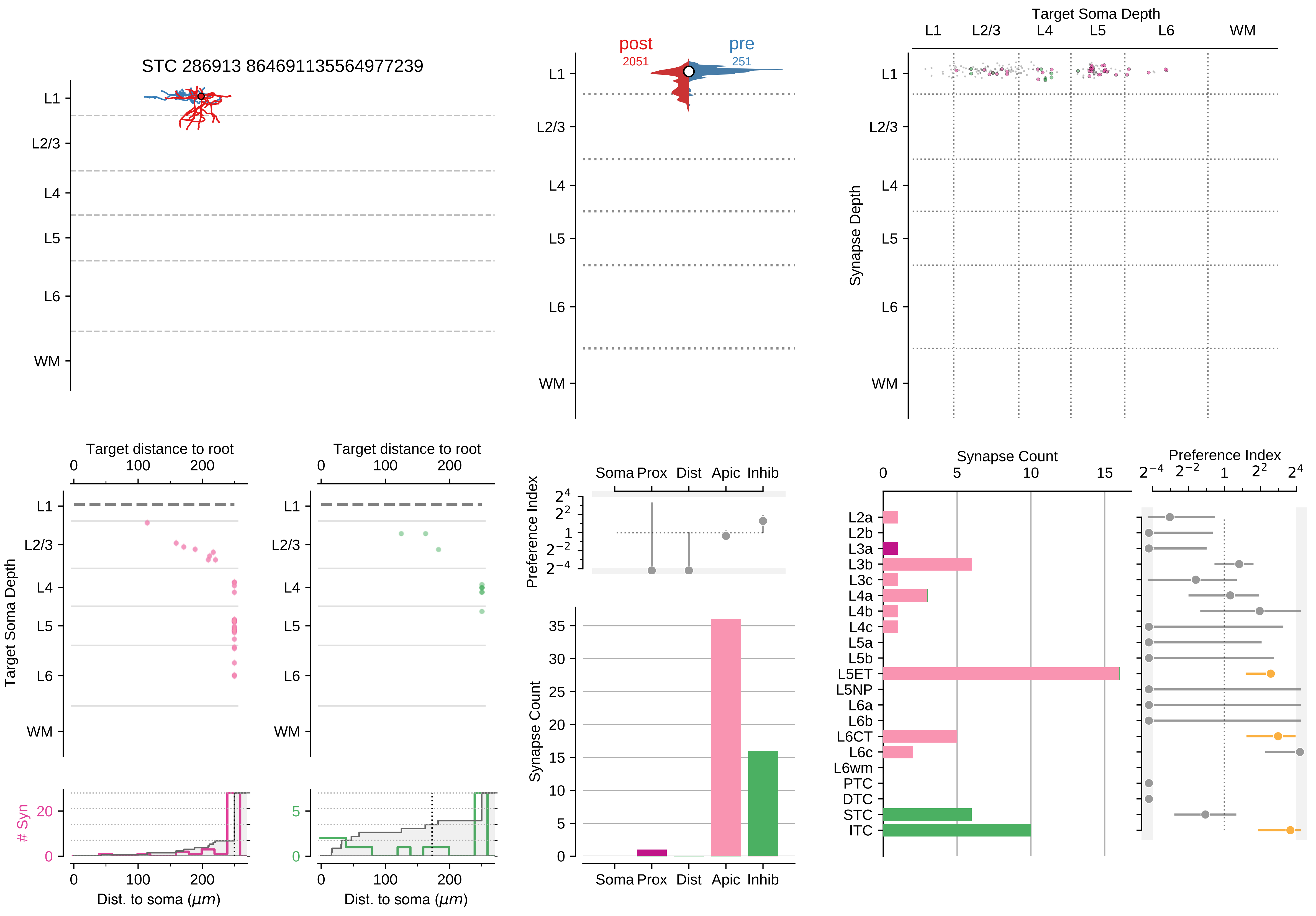

STC 286913 864691135564977239
L1
L2/3
L4
L5
L6
WM
post
2051
pre
251
Target Soma Depth
Synapse Depth
Target distance to root
Target Soma Depth
Syn
Dist. to soma (μm)
Soma Prox Dist Apic Inhib
Preference Index
Synapse Count
L2a
L2b
L3a
L3b
L3c
L4a
L4b
L4c
L5a
L5b
L5ET
L5NP
L6a
L6b
L6CT
L6c
L6wm
PTC
DTC
STC
ITC

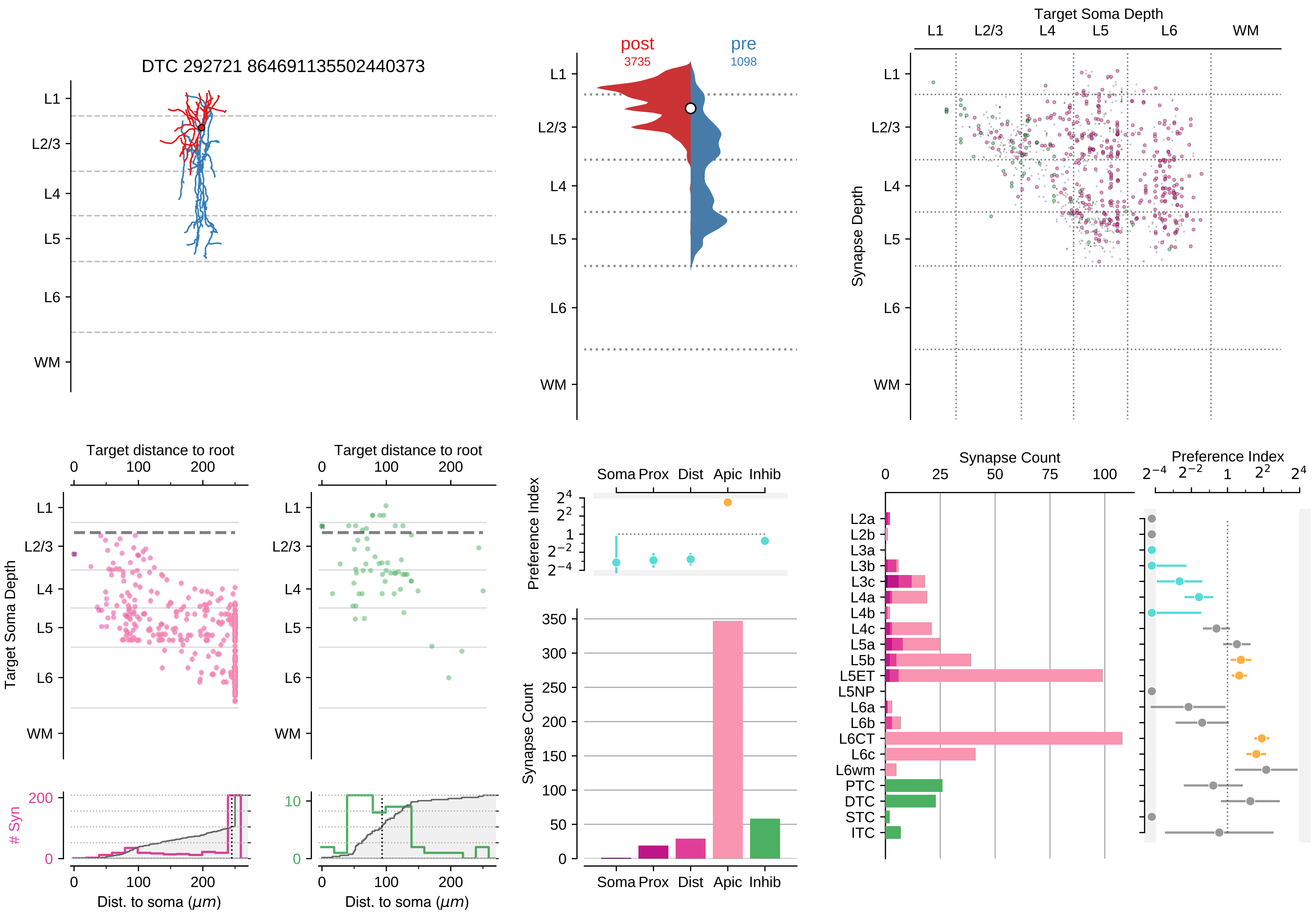

DTC 292721 864691135502440373
L1
L2/3
L4
L5
L6
WM
post
3735
pre
1098
Target Soma Depth
Synapse Depth
Target distance to root
Target Soma Depth
Syn
Dist. to soma (μm)
Preference Index
Soma
Prox
Dist
Apic
Inhib
Synapse Count
L2a
L2b
L3a
L3b
L3c
L4a
L4b
L4c
L5a
L5b
L5ET
L5NP
L6a
L6b
L6CT
L6c
L6wm
PTC
DTC
STC
ITC

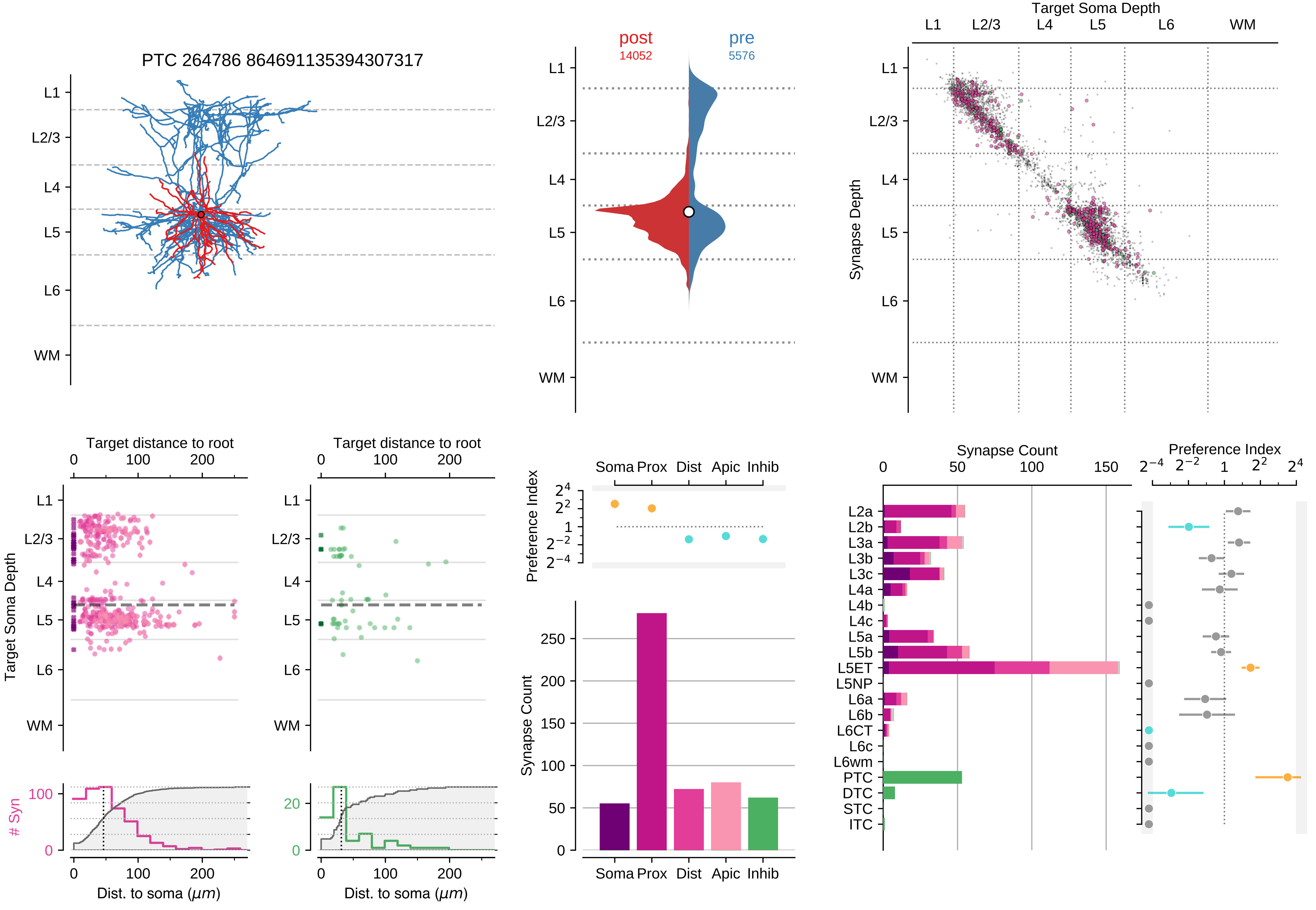

PTC 264786 864691135394307317
L1
L2/3
L4
L5
L6
WM
post
14052
pre
5576
Target Soma Depth
Synapse Depth
Target distance to root
Target Soma Depth
Syn
Dist. to soma (μm)
Preference Index
Soma
Prox
Dist
Apic
Inhib
Synapse Count
L2a
L2b
L3a
L3b
L3c
L4a
L4b
L4c
L5a
L5b
L5ET
L5NP
L6a
L6b
L6CT
L6c
L6wm
PTC
DTC
STC
ITC

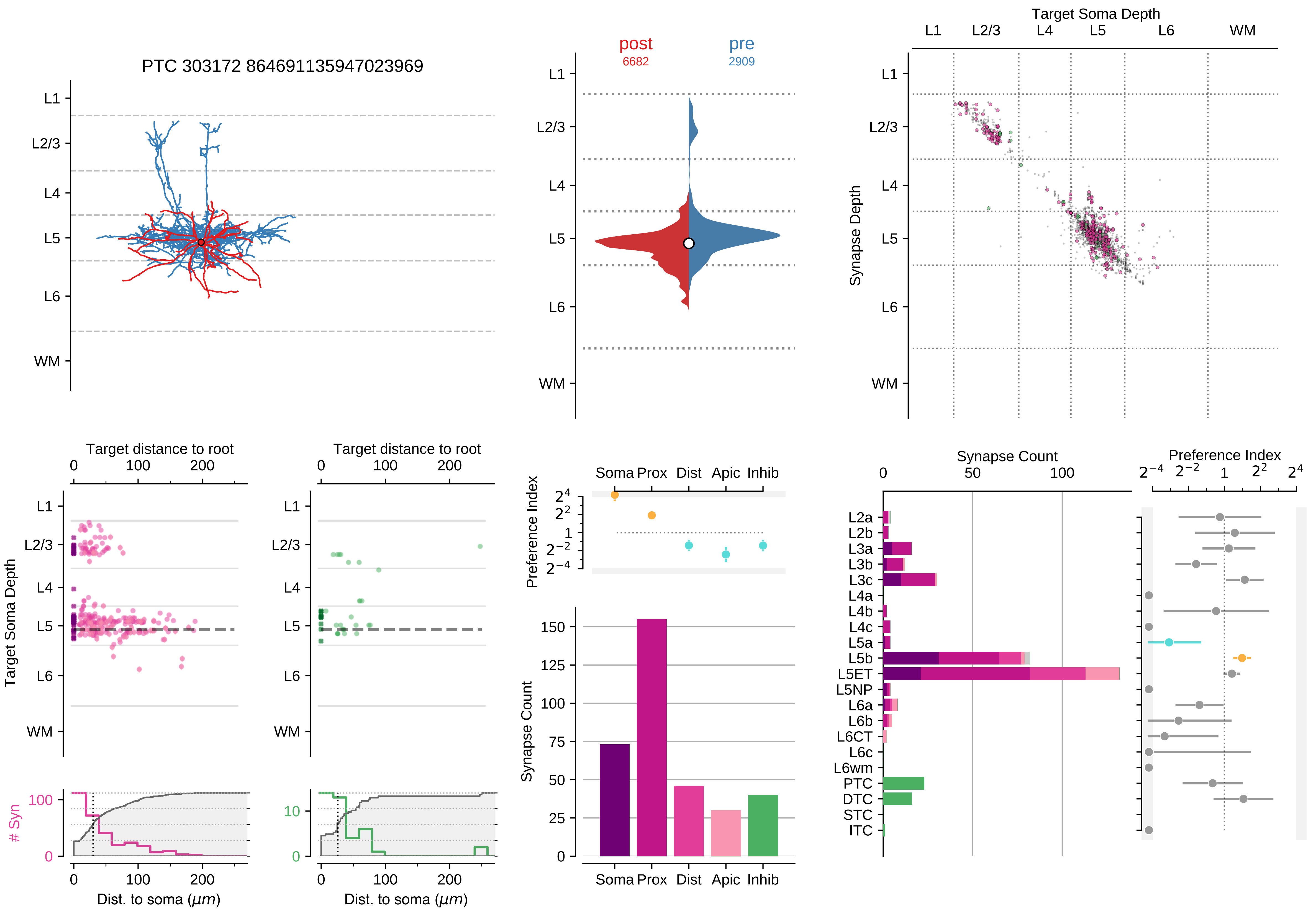
PTC 303172 864691135947023969
post
6682
pre
2909
Target Soma Depth
L1
L2/3
L4
L5
L6
WM
Synapse Depth
Target distance to root
Target Soma Depth
Syn
Dist. to soma (μm)
Preference Index
Soma
Prox
Dist
Apic
Inhib
Synapse Count
L2a
L2b
L3a
L3b
L3c
L4a
L4b
L4c
L5a
L5b
L5ET
L5NP
L6a
L6b
L6CT
L6c
L6wm
PTC
DTC
STC
ITC

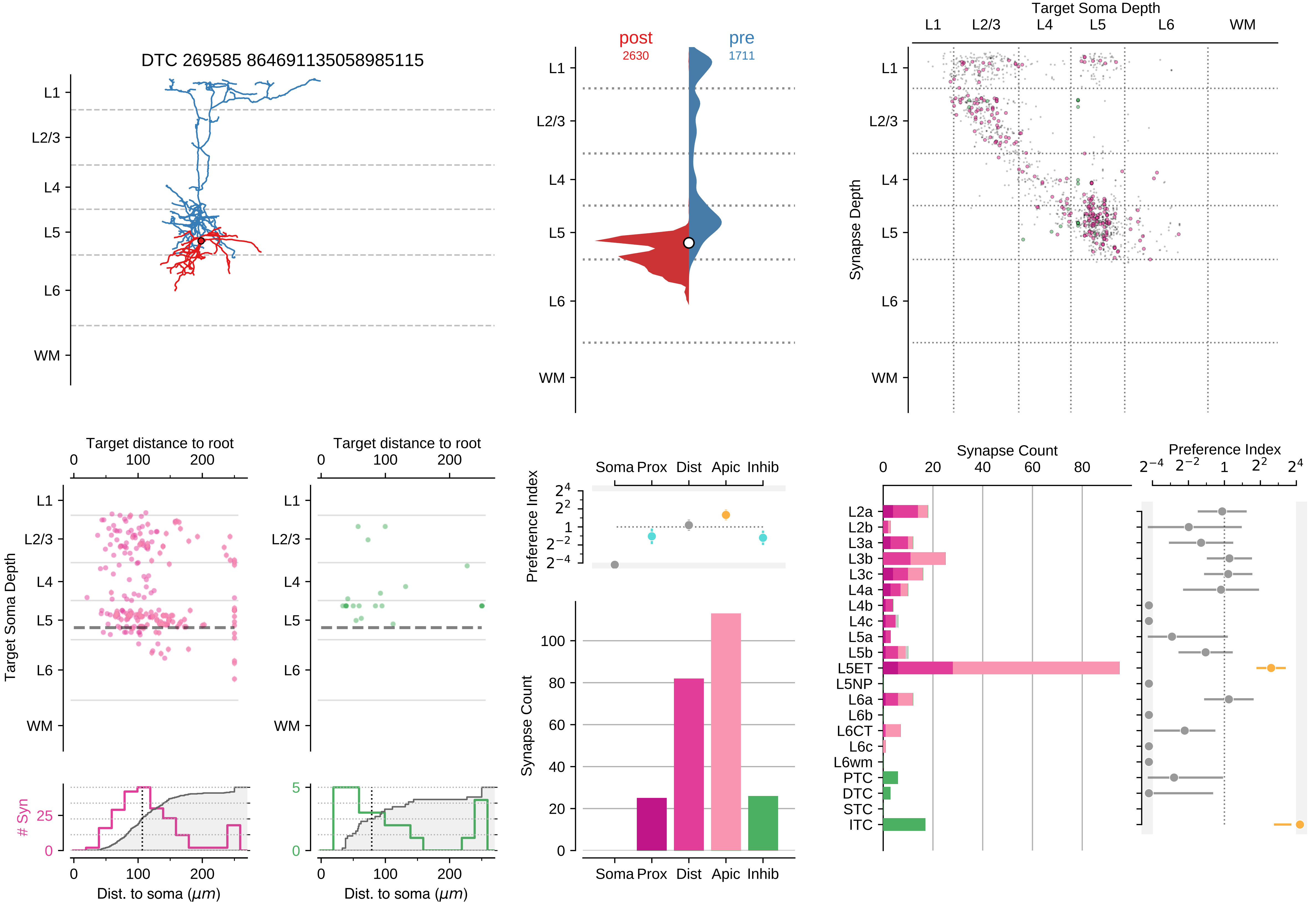

DTC 269585 864691135058985115
L1
L2/3
L4
L5
L6
WM
post
2630
pre
1711
Target Soma Depth
Synapse Depth
Target distance to root
Target Soma Depth
Syn
Dist. to soma (μm)
Preference Index
Soma Prox Dist Apic Inhib
Synapse Count
L2a
L2b
L3a
L3b
L3c
L4a
L4b
L4c
L5a
L5b
L5ET
L5NP
L6a
L6b
L6CT
L6c
L6wm
PTC
DTC
STC
ITC

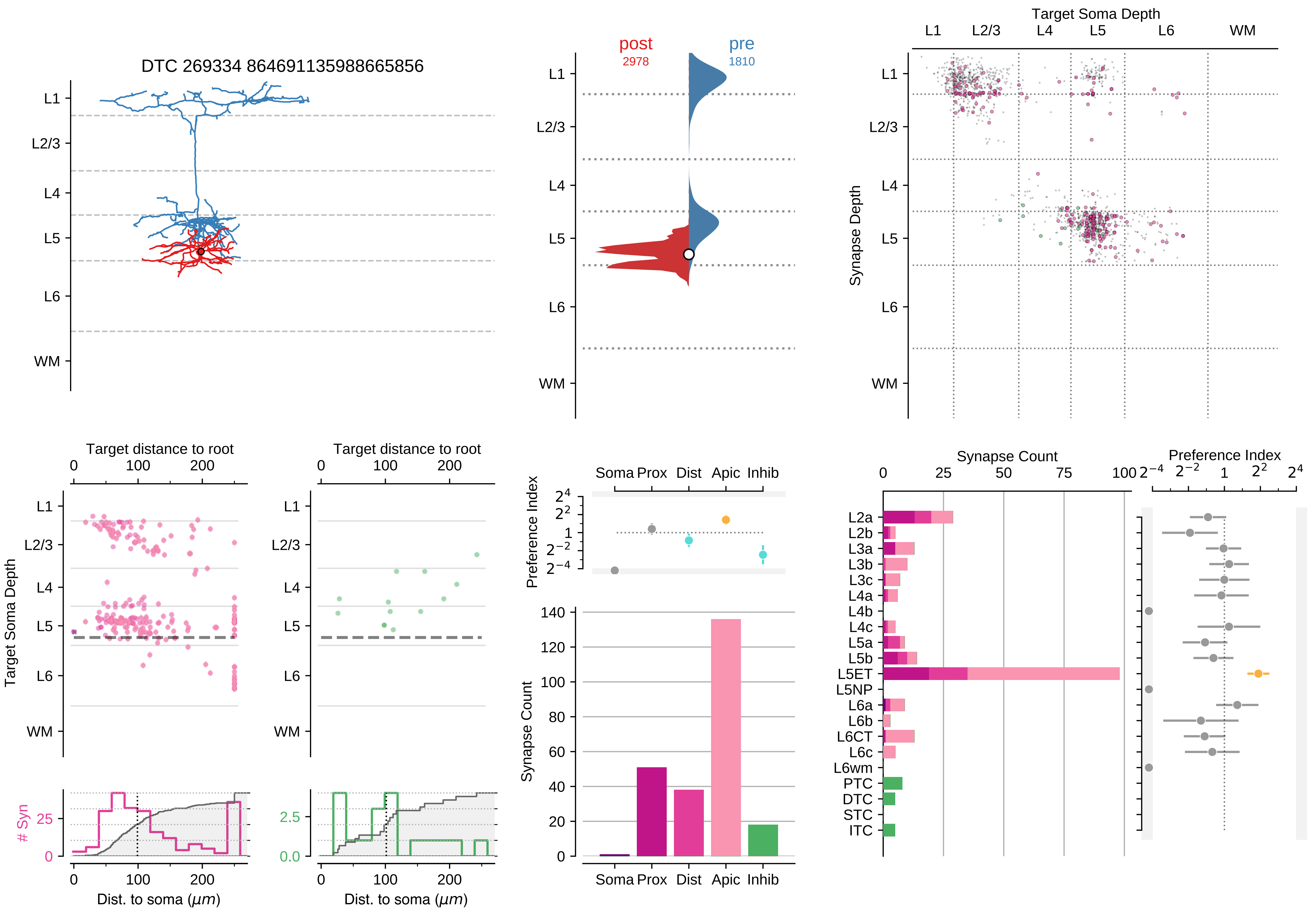

DTC 269334 864691135988665856
L1
L2/3
L4
L5
L6
WM
post
2978
pre
1810
Target Soma Depth
Synapse Depth
Target distance to root
Target Soma Depth
Syn
Dist. to soma (μm)
Preference Index
Soma
Prox
Dist
Apic
Inhib
Synapse Count
L2a
L2b
L3a
L3b
L3c
L4a
L4b
L4c
L5a
L5b
L5ET
L5NP
L6a
L6b
L6CT
L6c
L6wm
PTC
DTC
STC
ITC

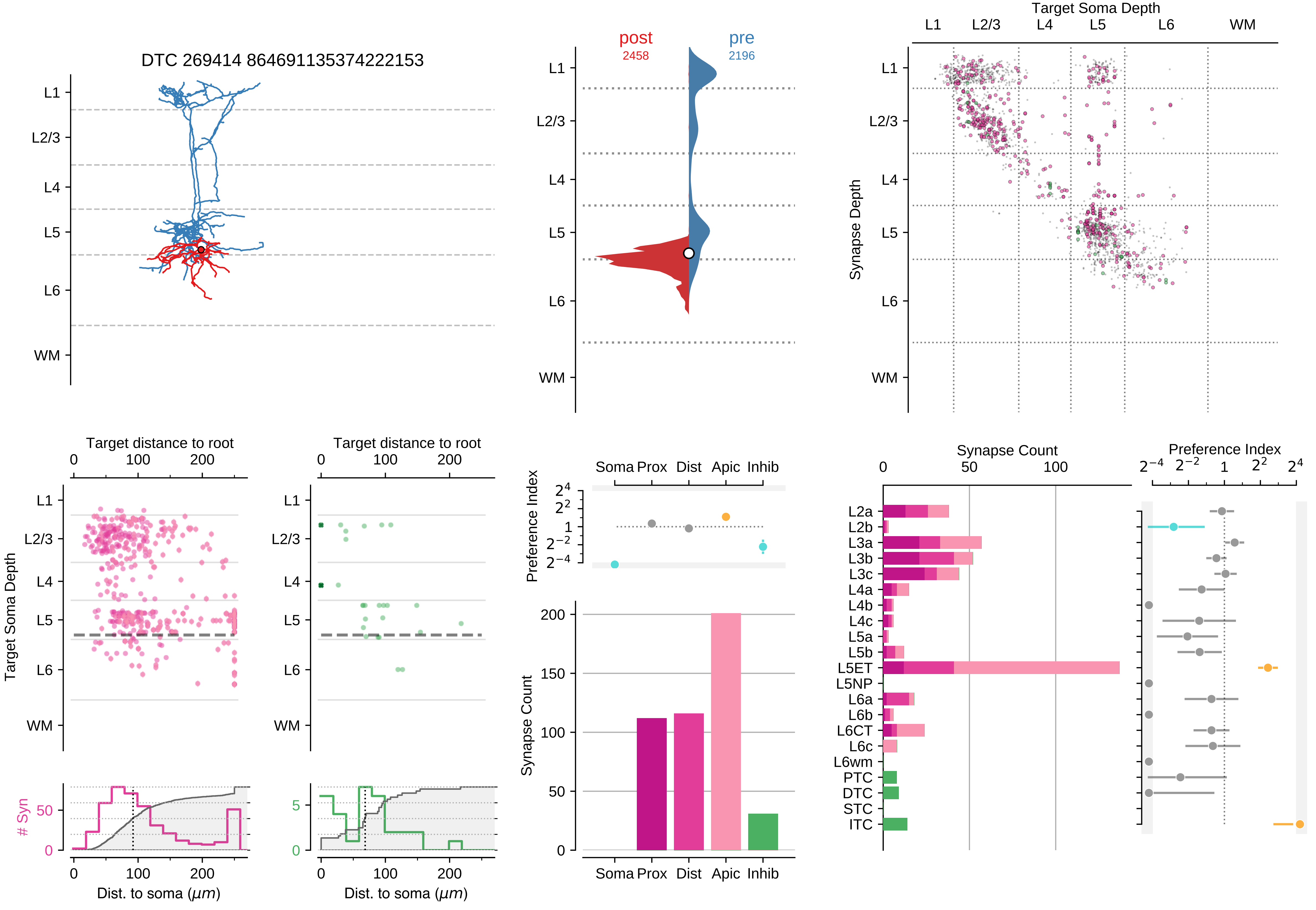

DTC 269414 864691135374222153
L1
L2/3
L4
L5
L6
WM
post
2458
pre
2196
Target Soma Depth
Synapse Depth
Target distance to root
Target Soma Depth
Syn
Dist. to soma (μm)
Preference Index
Soma Prox Dist Apic Inhib
Synapse Count
L2a
L2b
L3a
L3b
L3c
L4a
L4b
L4c
L5a
L5b
L5ET
L5NP
L6a
L6b
L6CT
L6c
L6wm
PTC
DTC
STC
ITC

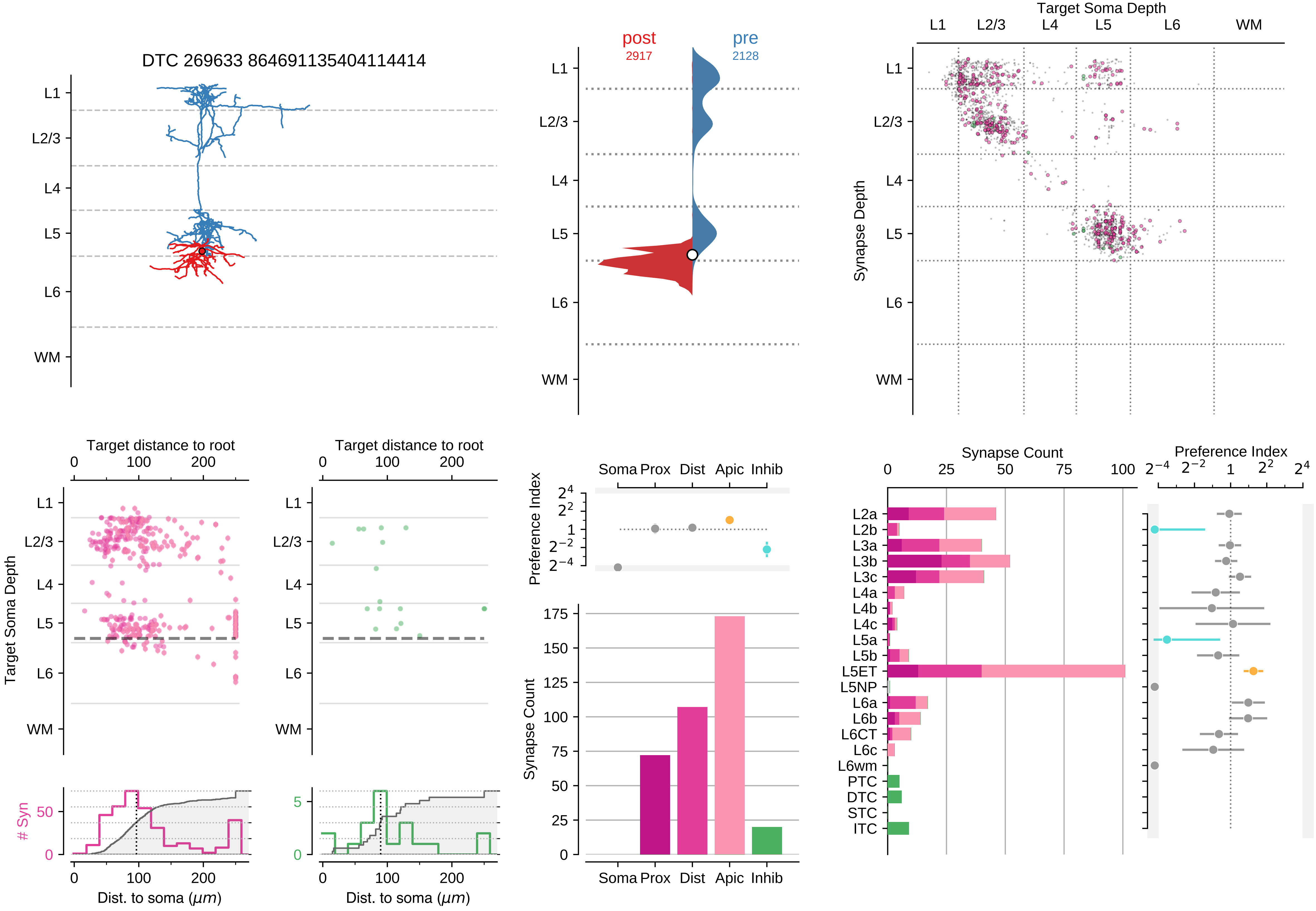

DTC 269633 864691135404114414
L1
L2/3
L4
L5
L6
WM
post
2917
pre
2128
Target Soma Depth
Synapse Depth
Target distance to root
Target Soma Depth
Syn
Dist. to soma (μm)
Preference Index
Soma Prox Dist Apic Inhib
Synapse Count
L2a
L2b
L3a
L3b
L3c
L4a
L4b
L4c
L5a
L5b
L5ET
L5NP
L6a
L6b
L6CT
L6c
L6wm
PTC
DTC
STC
ITC

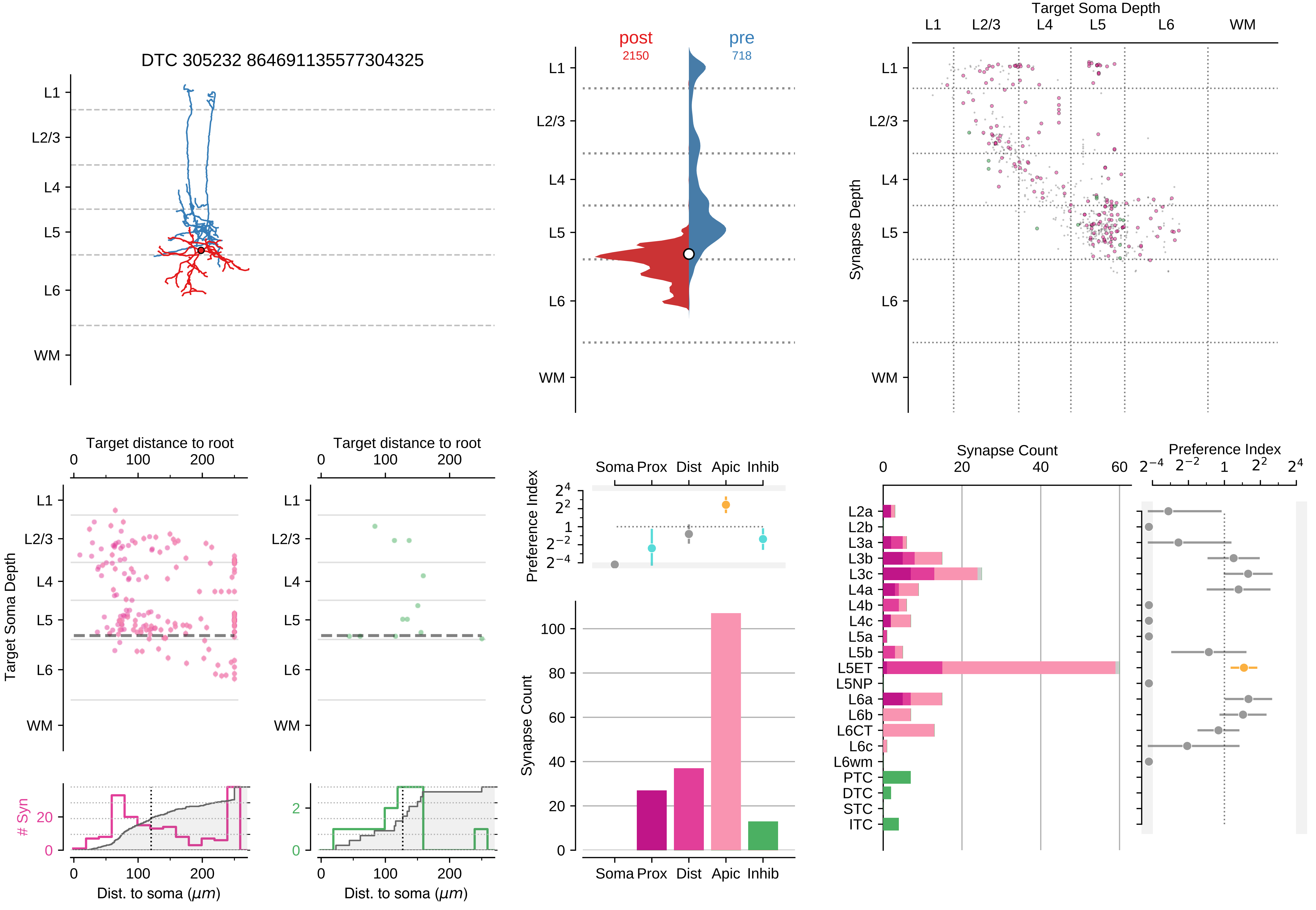

DTC 305232 864691135577304325
L1
L2/3
L4
L5
L6
WM
post
2150
pre
718
Target Soma Depth
Synapse Depth
Target distance to root
Target Soma Depth
Syn
Dist. to soma (μm)
Preference Index
Soma Prox Dist Apic Inhib
Synapse Count
L2a
L2b
L3a
L3b
L3c
L4a
L4b
L4c
L5a
L5b
L5ET
L5NP
L6a
L6b
L6CT
L6c
L6wm
PTC
DTC
STC
ITC

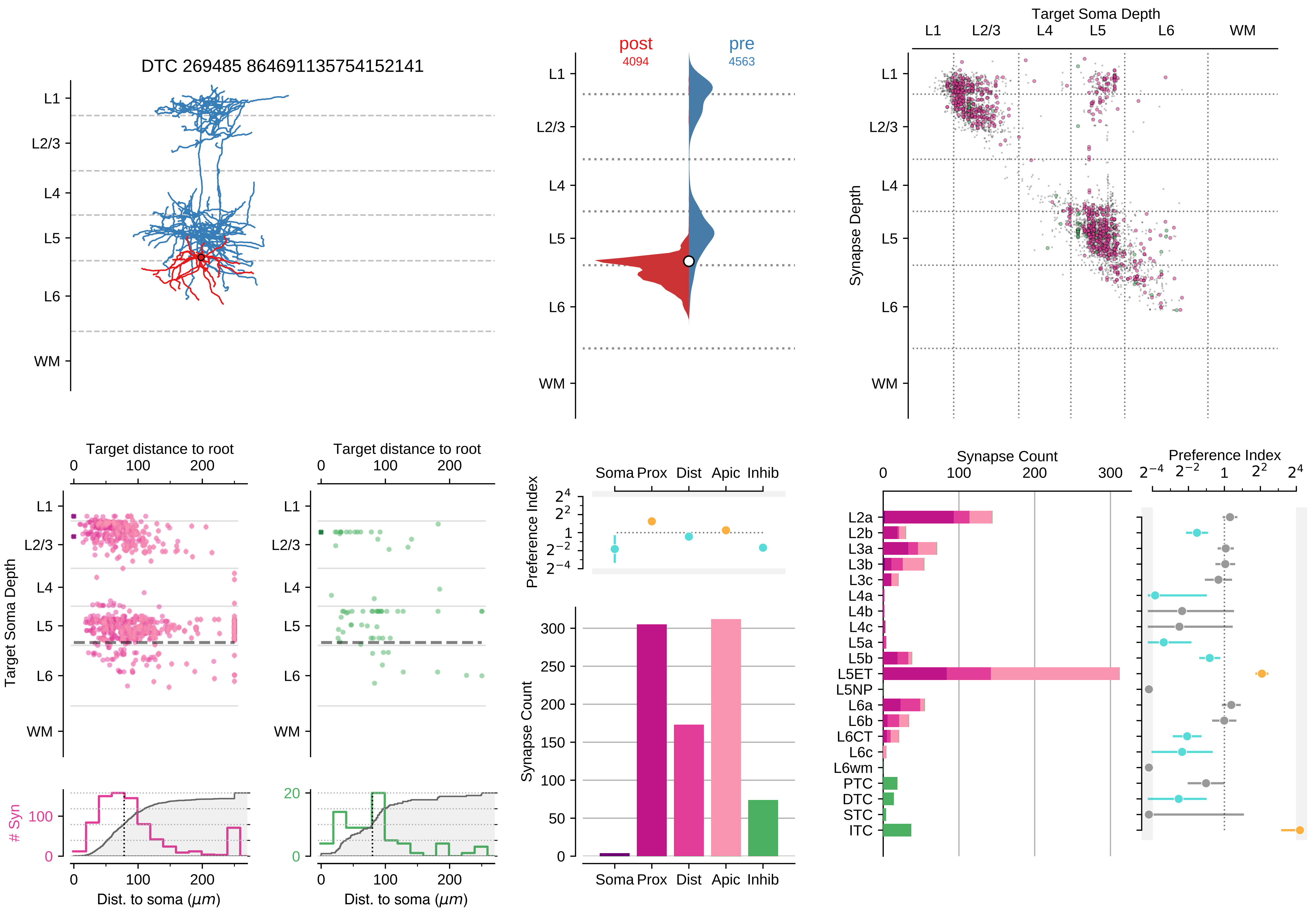

DTC 269485 864691135754152141
L1
L2/3
L4
L5
L6
WM
post
4094
pre
4563
Target Soma Depth
Synapse Depth
Target distance to root
Target Soma Depth
Syn
Dist. to soma (μm)
Preference Index
Soma Prox Dist Apic Inhib
Synapse Count
L2a
L2b
L3a
L3b
L3c
L4a
L4b
L4c
L5a
L5b
L5ET
L5NP
L6a
L6b
L6CT
L6c
L6wm
PTC
DTC
STC
ITC

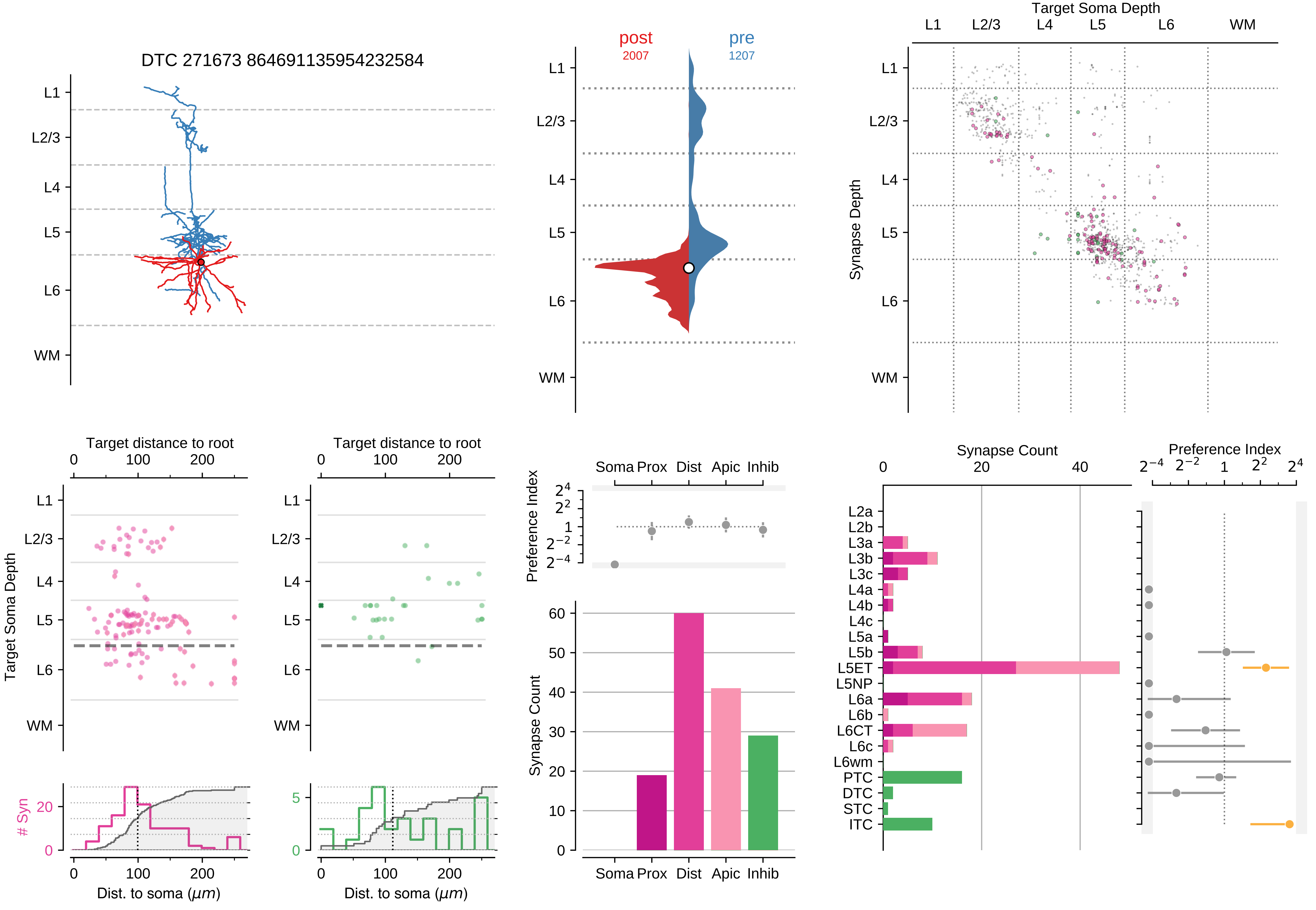

DTC 271673 864691135954232584
L1
L2/3
L4
L5
L6
WM
post
2007
pre
1207
Target Soma Depth
Synapse Depth
Target distance to root
Target Soma Depth
Syn
Dist. to soma (μm)
Preference Index
Soma Prox Dist Apic Inhib
Synapse Count
L2a
L2b
L3a
L3b
L3c
L4a
L4b
L4c
L5a
L5b
L5ET
L5NP
L6a
L6b
L6CT
L6c
L6wm
PTC
DTC
STC
ITC

Group 10

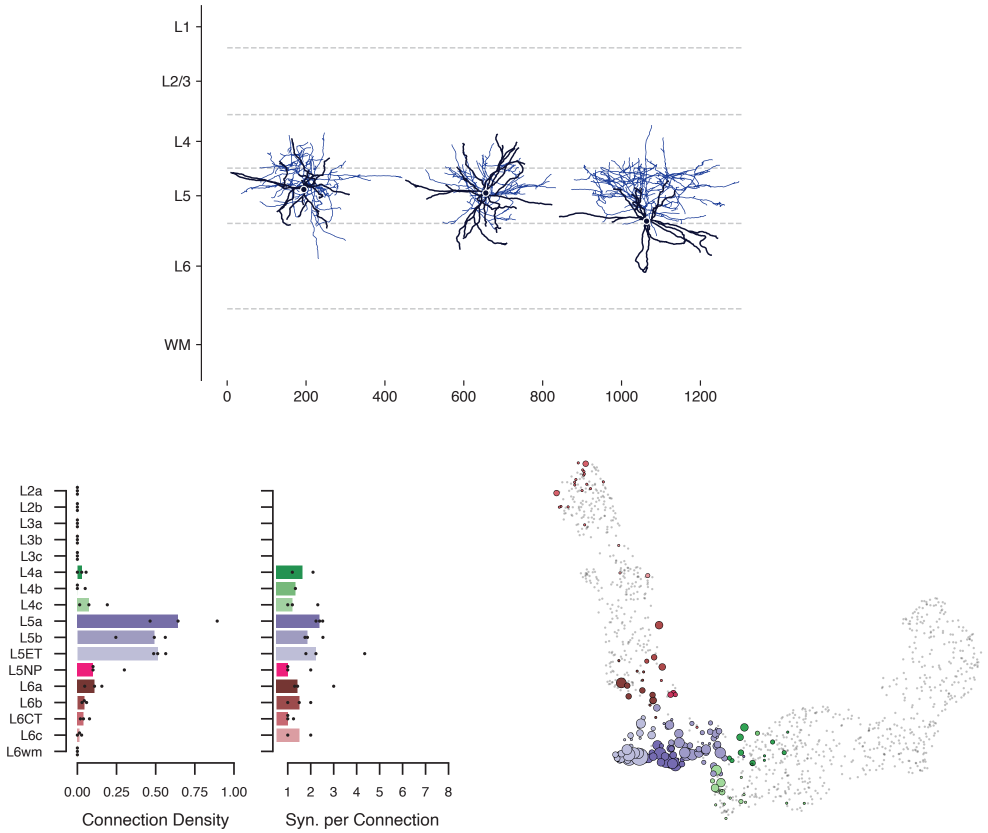
L1
L2/3
L4
L5
L6
WM
0
200
400
600
800
1000
1200
L2a
L2b
L3a
L3b
L3c
L4a
L4b
L4c
L5a
L5b
L5ET
L5NP
L6a
L6b
L6CT
L6c
L6wm
0.00
0.25
0.50
0.75
1.00
Connection Density
1
2
3
4
5
6
7
8
Syn. per Connection

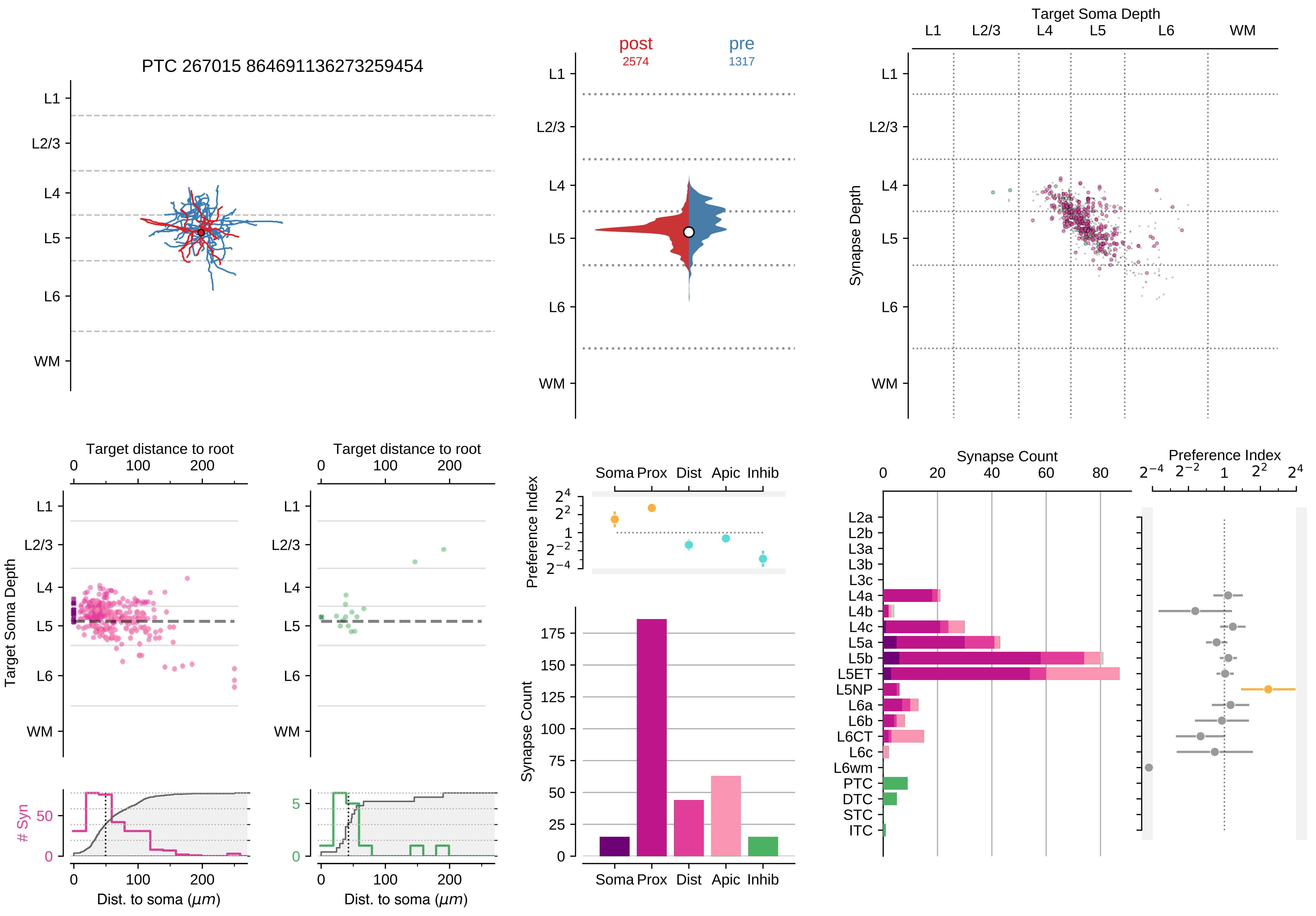
PTC 267015 864691136273259454
post
2574
pre
1317
Target Soma Depth
L1
L2/3
L4
L5
L6
WM
Synapse Depth
Target distance to root
Target Soma Depth
Syn
Dist. to soma (μm)
Preference Index
Soma
Prox
Dist
Apic
Inhib
Synapse Count
L2a
L2b
L3a
L3b
L3c
L4a
L4b
L4c
L5a
L5b
L5ET
L5NP
L6a
L6b
L6CT
L6c
L6wm
PTC
DTC
STC
ITC

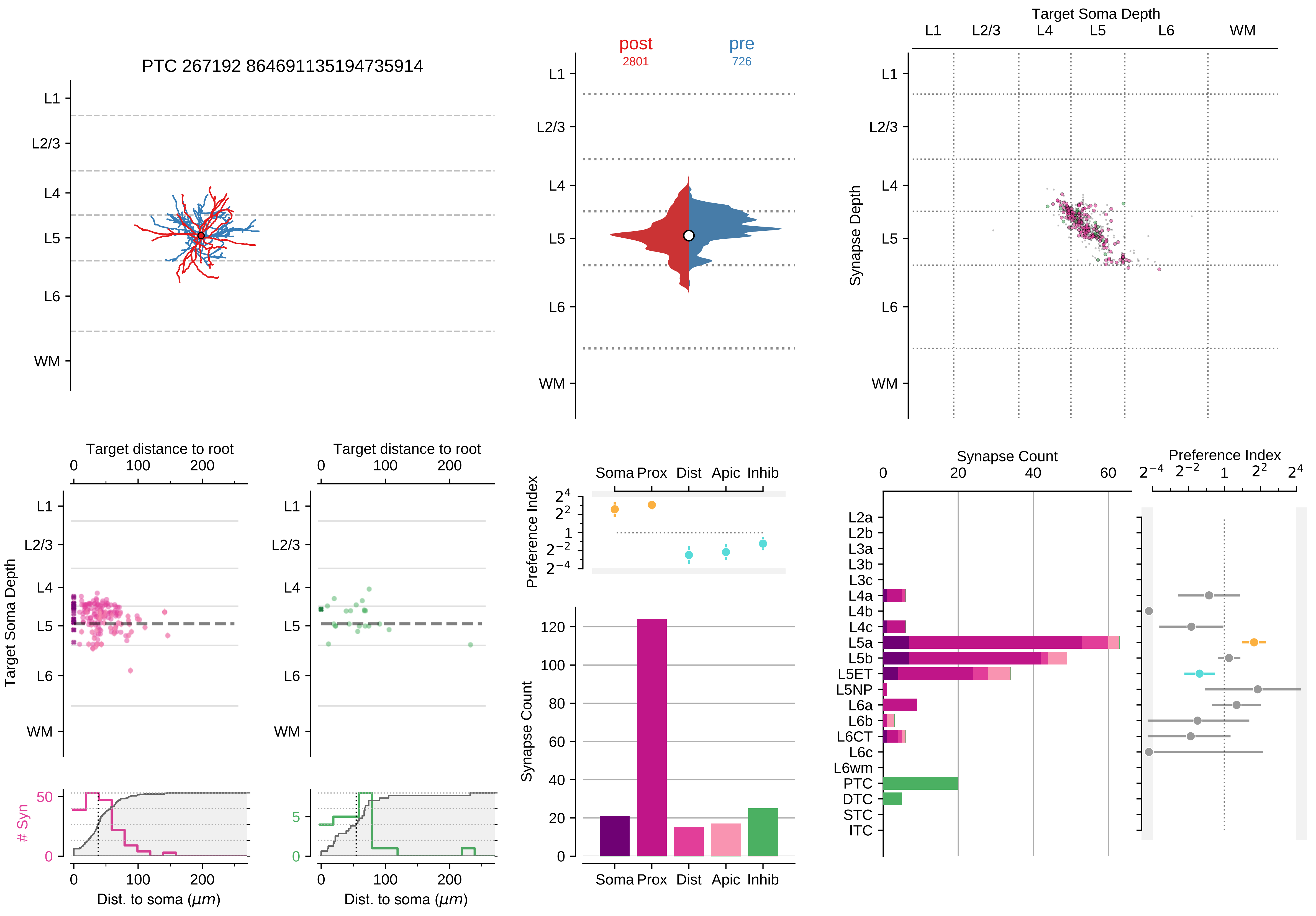

PTC 267192 864691135194735914
L1
L2/3
L4
L5
L6
WM
post
2801
pre
726
Target Soma Depth
Synapse Depth
Target distance to root
Target Soma Depth
Syn
Dist. to soma (μm)
Preference Index
Soma Prox Dist Apic Inhib
Synapse Count
L2a
L2b
L3a
L3b
L3c
L4a
L4b
L4c
L5a
L5b
L5ET
L5NP
L6a
L6b
L6CT
L6c
L6wm
PTC
DTC
STC
ITC

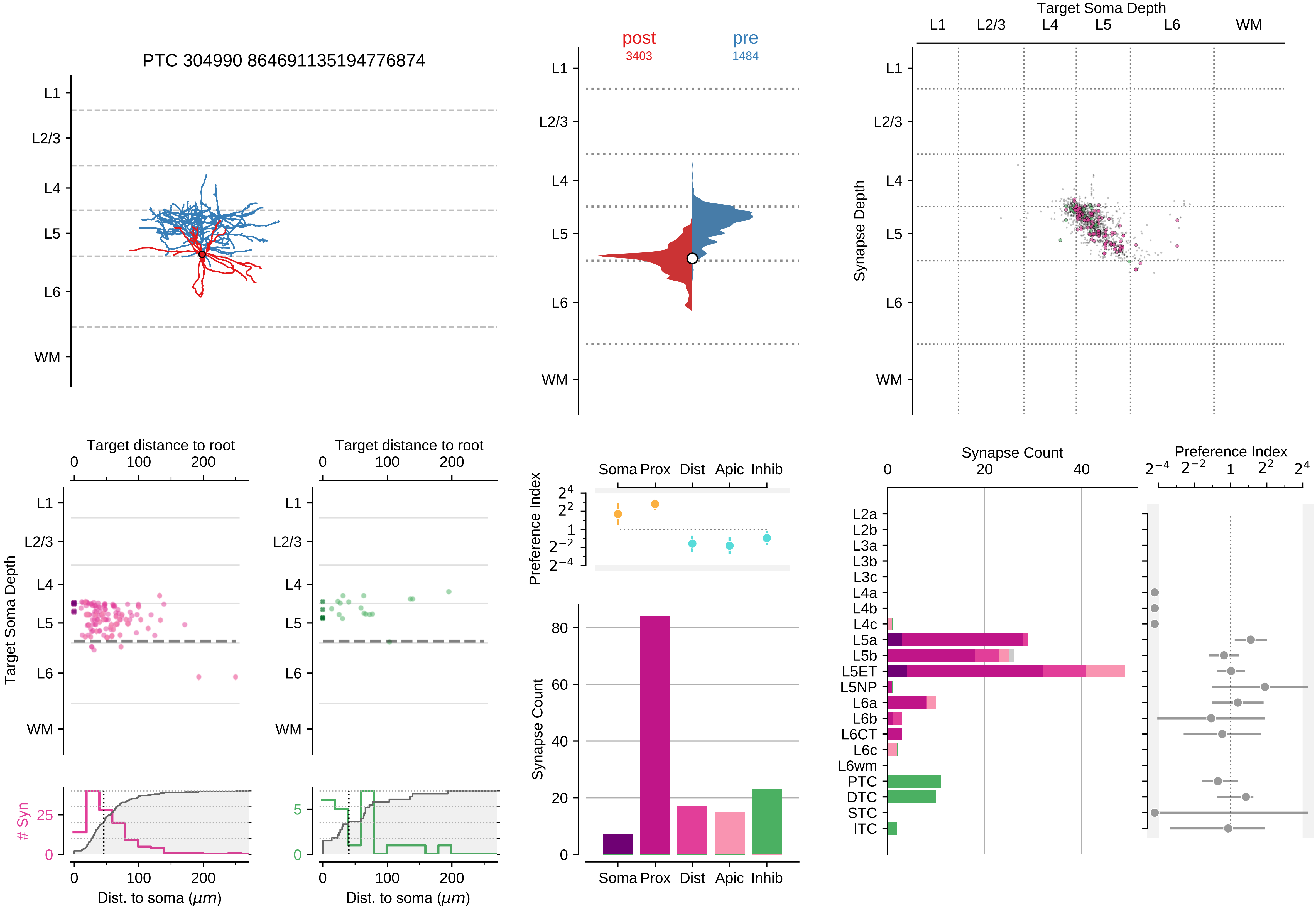

PTC 304990 864691135194776874
post
3403
pre
1484
Target Soma Depth
Synapse Depth
L1
L2/3
L4
L5
L6
WM
Target distance to root
Target Soma Depth
Syn
Dist. to soma (μm)
Preference Index
Soma Prox Dist Apic Inhib
Synapse Count
L2a
L2b
L3a
L3b
L3c
L4a
L4b
L4c
L5a
L5b
L5ET
L5NP
L6a
L6b
L6CT
L6c
L6wm
PTC
DTC
STC
ITC

Group 11

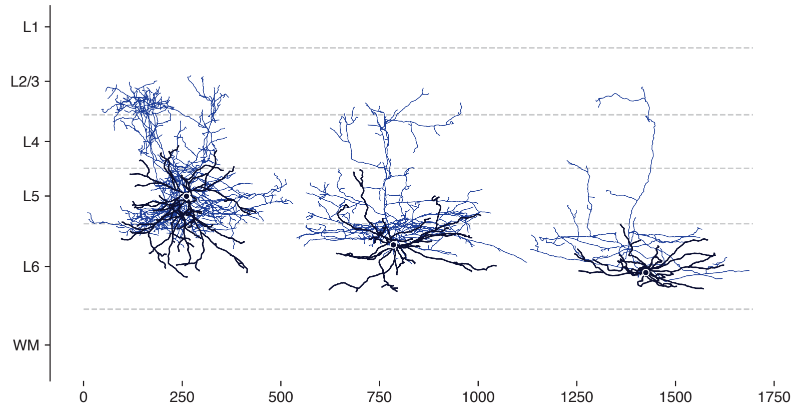

L1
L2/3
L4
L5
L6
WM
0
250
500
750
1000
1250
1500
1750

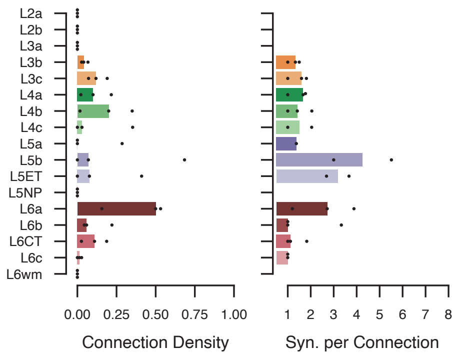

L2a
L2b
L3a
L3b
L3c
L4a
L4b
L4c
L5a
L5b
L5ET
L5NP
L6a
L6b
L6CT
L6c
L6wm
0.00
0.25
0.50
0.75
1.00
Connection Density
1
2
3
4
5
6
7
8
Syn. per Connection

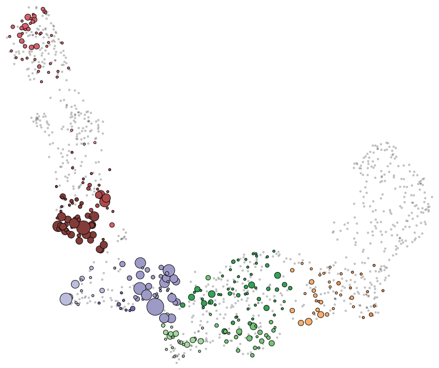

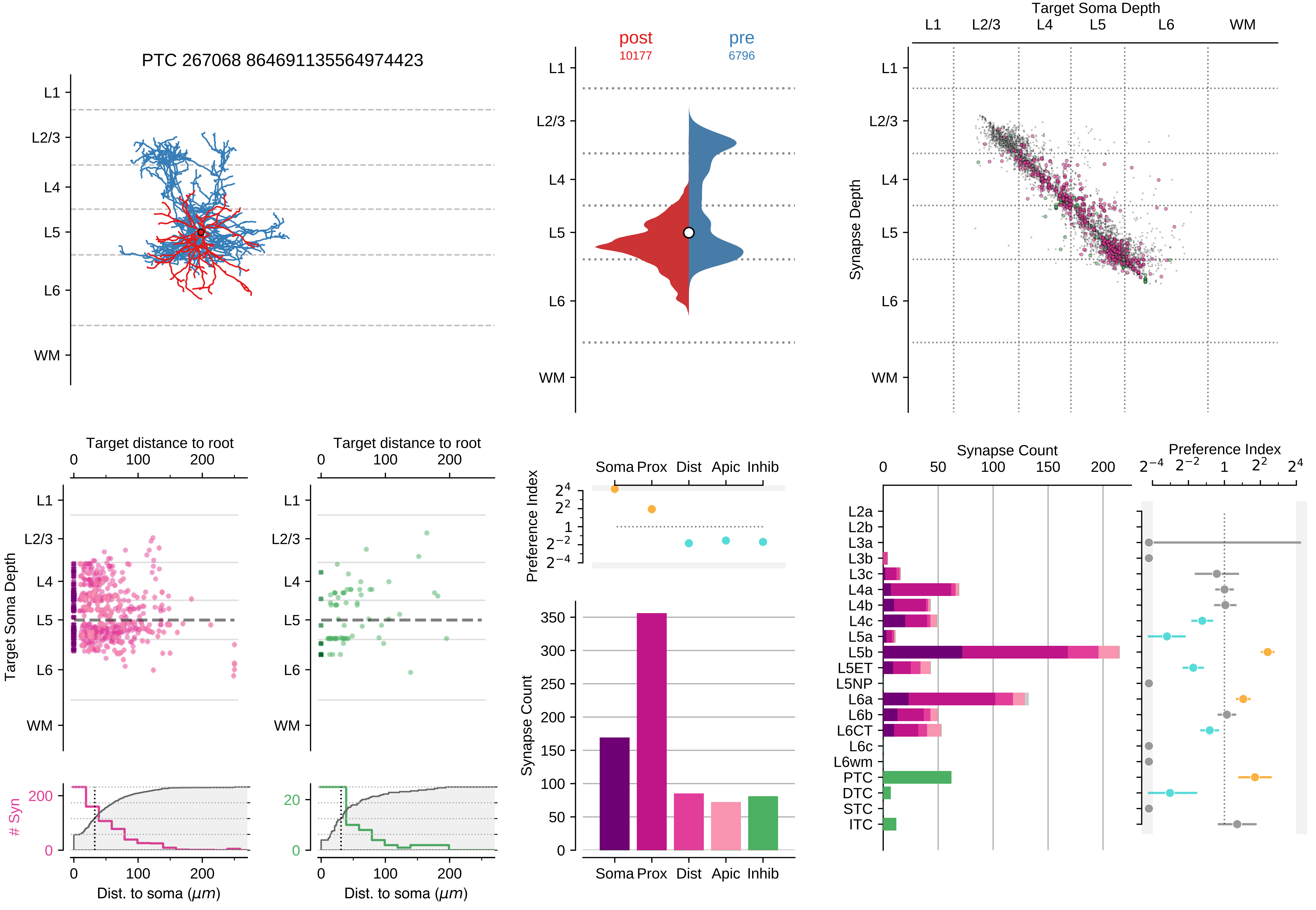
PTC 267068 864691135564974423
L1
L2/3
L4
L5
L6
WM
post
10177
pre
6796
Target Soma Depth
Synapse Depth
Target distance to root
Target Soma Depth
Syn
Dist. to soma (μm)
Preference Index
Soma
Prox
Dist
Apic
Inhib
Synapse Count
L2a
L2b
L3a
L3b
L3c
L4a
L4b
L4c
L5a
L5b
L5ET
L5NP
L6a
L6b
L6CT
L6c
L6wm
PTC
DTC
STC
ITC

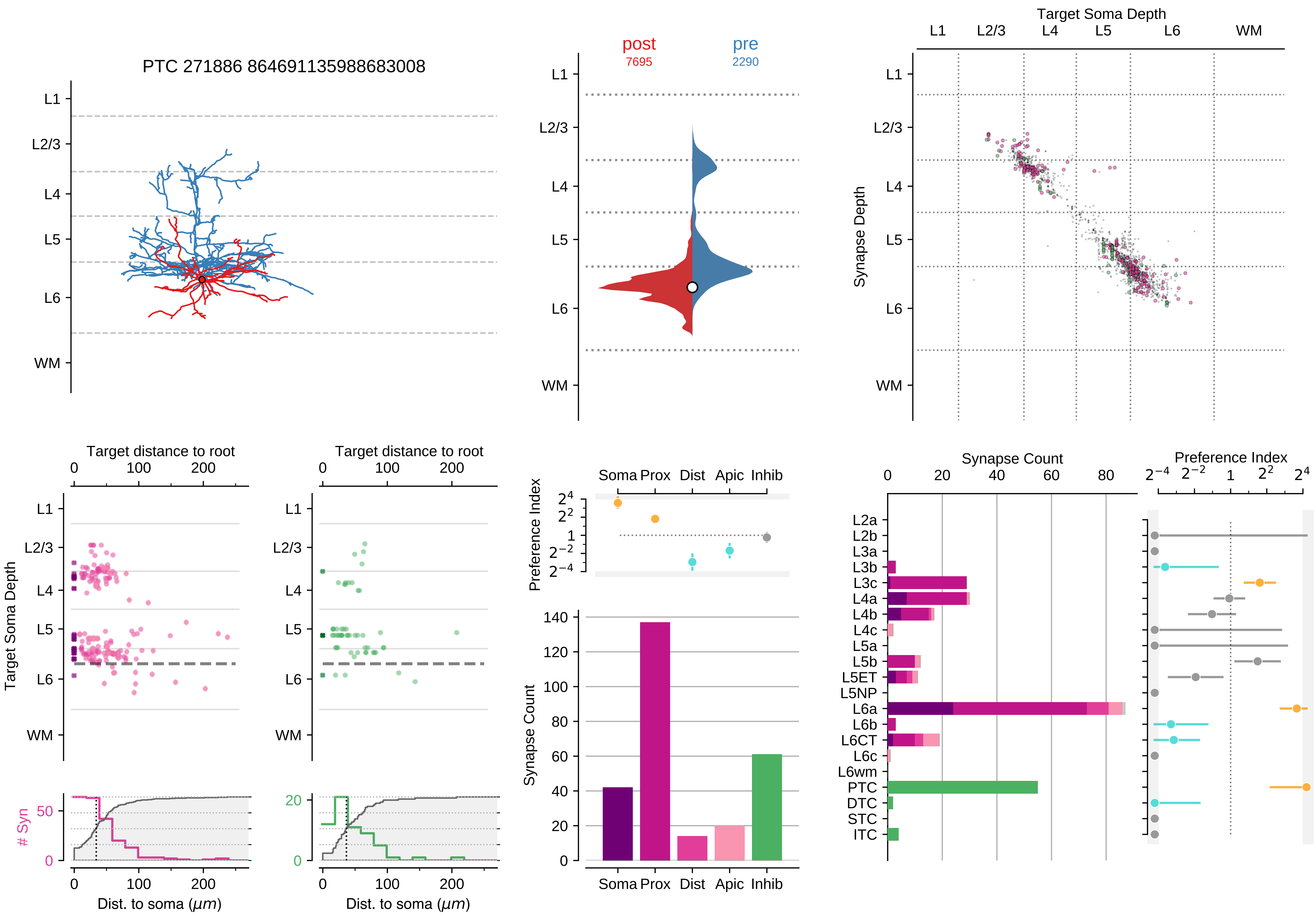
PTC 271886 864691135988683008
L1
L2/3
L4
L5
L6
WM
post
7695
pre
2290
Target Soma Depth
Synapse Depth
Target distance to root
Target Soma Depth
Syn
Dist. to soma (μm)
Preference Index
Soma Prox Dist Apic Inhib
Synapse Count
L2a
L2b
L3a
L3b
L3c
L4a
L4b
L4c
L5a
L5b
L5ET
L5NP
L6a
L6b
L6CT
L6c
L6wm
PTC
DTC
STC
ITC

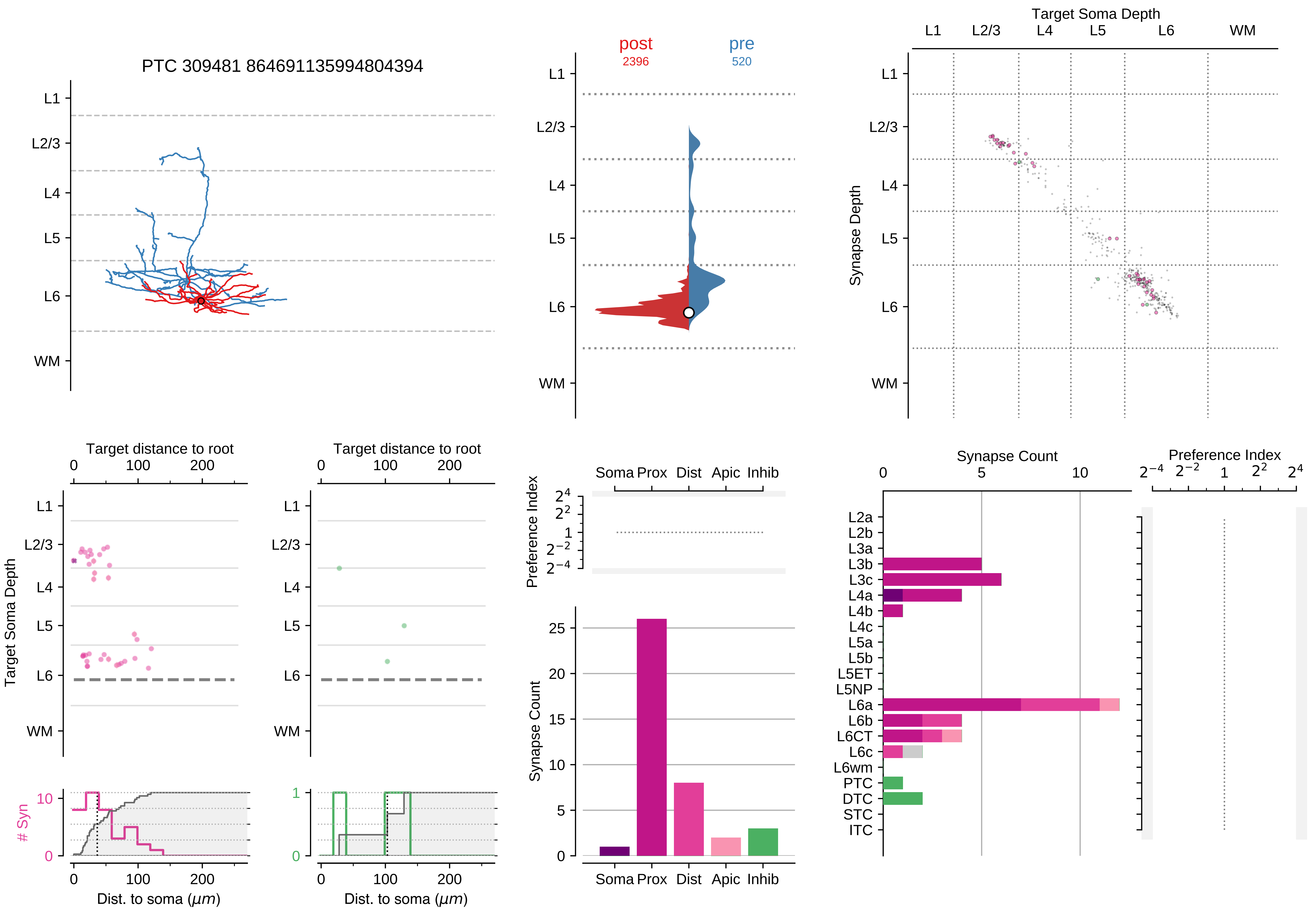

PTC 309481 864691135994804394
L1
L2/3
L4
L5
L6
WM
post
2396
pre
520
Target Soma Depth
Synapse Depth
Target distance to root
Target Soma Depth
Syn
Dist. to soma (μm)
Preference Index
Soma
Prox
Dist
Apic
Inhib
Synapse Count
L2a
L2b
L3a
L3b
L3c
L4a
L4b
L4c
L5a
L5b
L5ET
L5NP
L6a
L6b
L6CT
L6c
L6wm
PTC
DTC
STC
ITC

Group 12

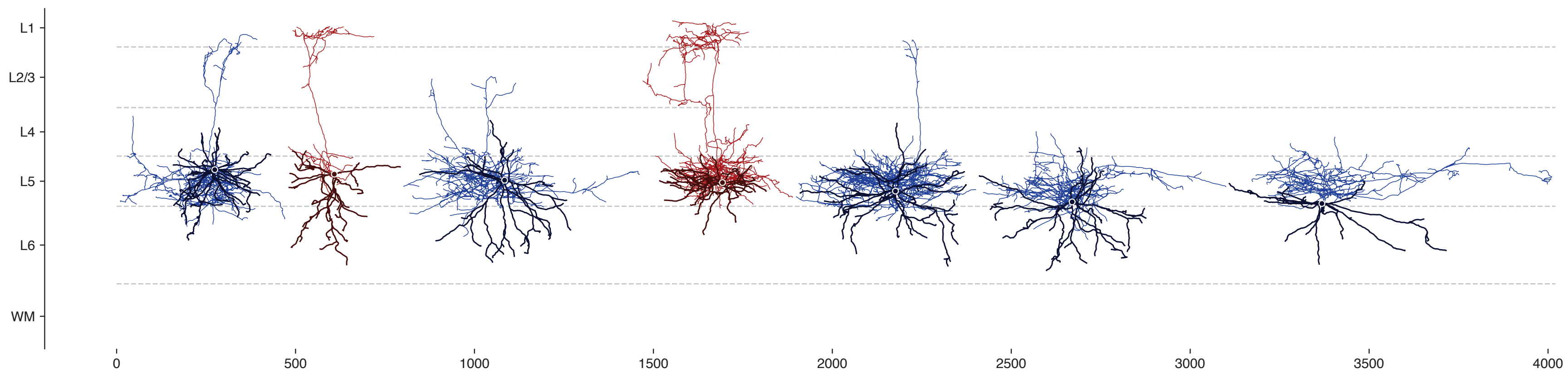

L1
L2/3
L4
L5
L6
WM
0
500
1000
1500
2000
2500
3000
3500
4000

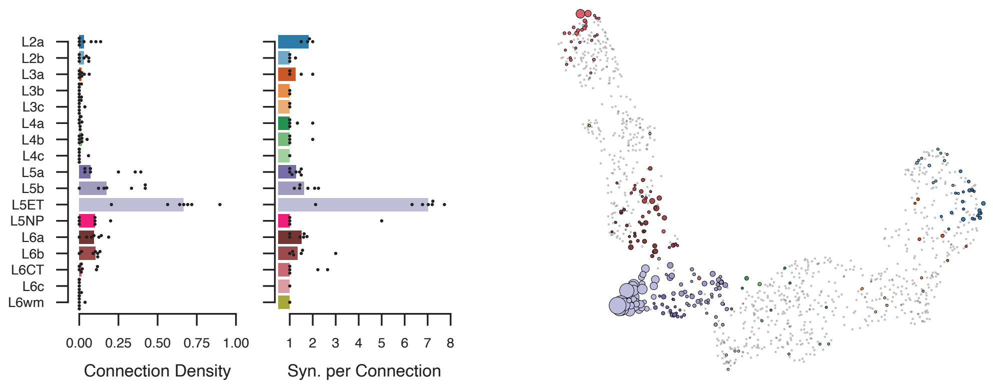

L2a
L2b
L3a
L3b
L3c
L4a
L4b
L4c
L5a
L5b
L5ET
L5NP
L6a
L6b
L6CT
L6c
L6wm
0.00
0.25
0.50
0.75
1.00
Connection Density
1
2
3
4
5
6
7
8
Syn. per Connection

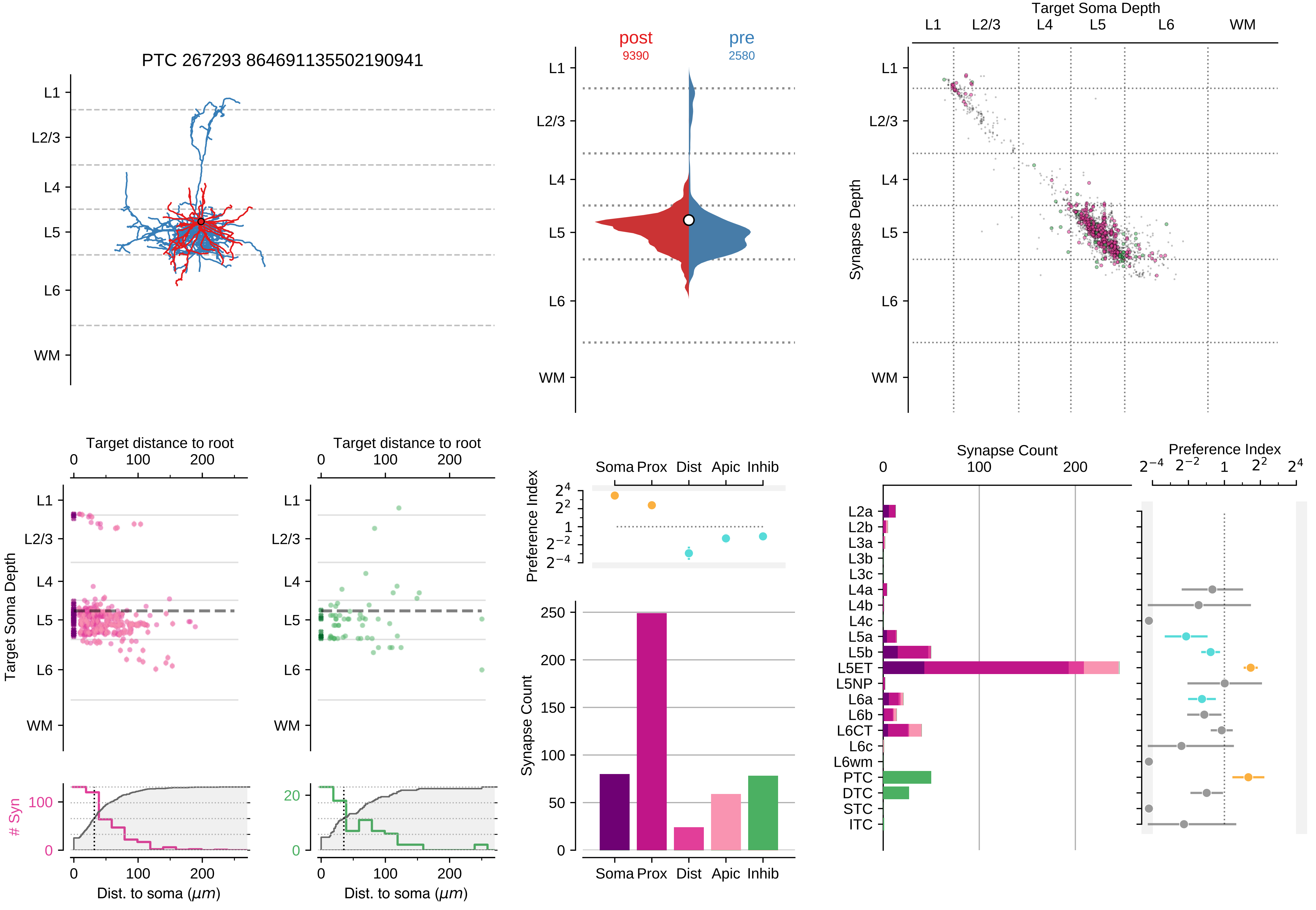

PTC 267293 864691135502190941
L1
L2/3
L4
L5
L6
WM
post
9390
pre
2580
Target Soma Depth
Synapse Depth
Target distance to root
Target Soma Depth
Dist. to soma (μm)
Syn
Preference Index
Soma
Prox
Dist
Apic
Inhib
Synapse Count
L2a
L2b
L3a
L3b
L3c
L4a
L4b
L4c
L5a
L5b
L5ET
L5NP
L6a
L6b
L6CT
L6c
L6wm
PTC
DTC
STC
ITC

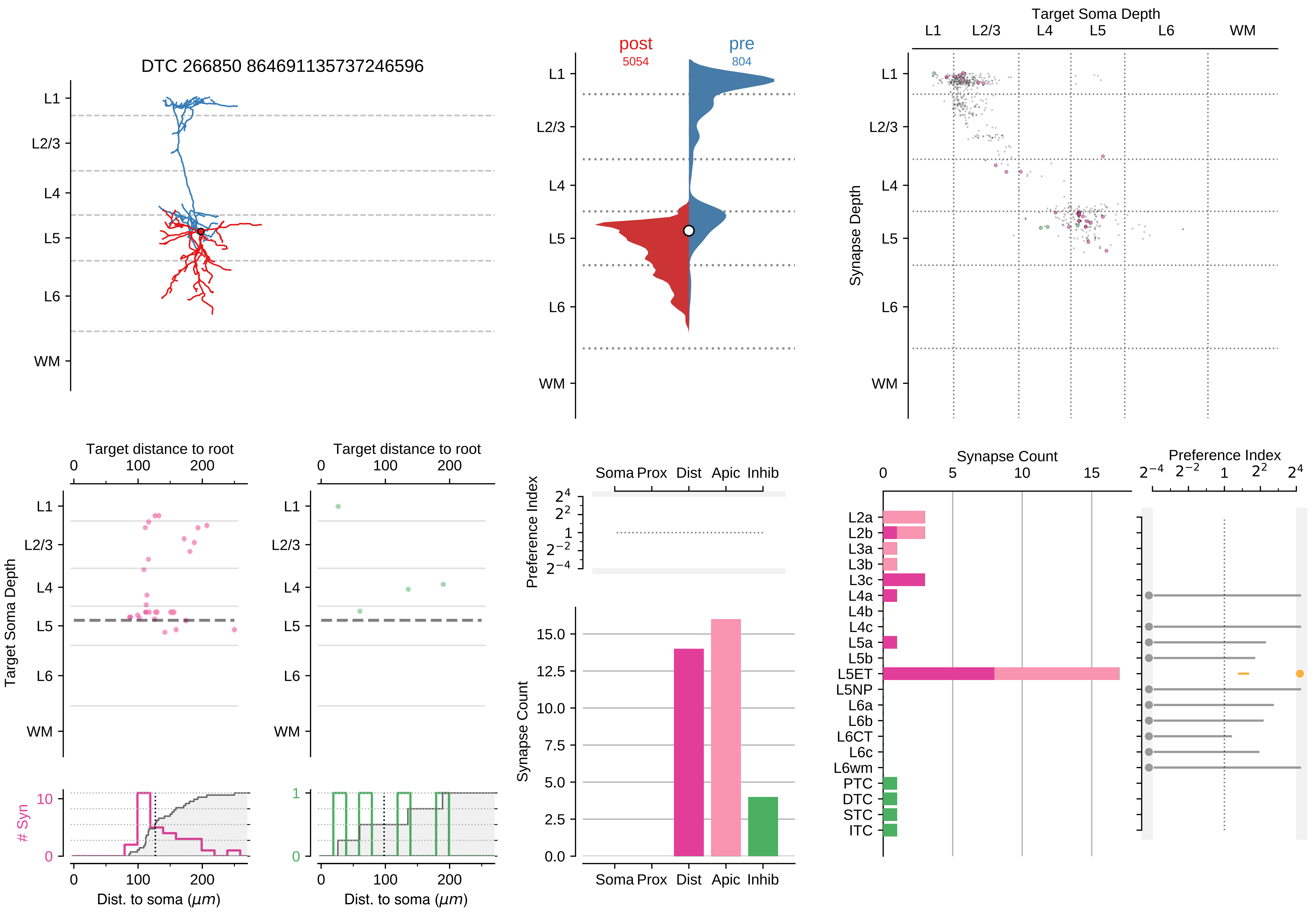

DTC 266850 864691135737246596
L1
L2/3
L4
L5
L6
WM
post
5054
pre
804
Target Soma Depth
Synapse Depth
Target distance to root
Target Soma Depth
Syn
Dist. to soma (μm)
Preference Index
Soma Prox Dist Apic Inhib
Synapse Count
L2a
L2b
L3a
L3b
L3c
L4a
L4b
L4c
L5a
L5b
L5ET
L5NP
L6a
L6b
L6CT
L6c
L6wm
PTC
DTC
STC
ITC

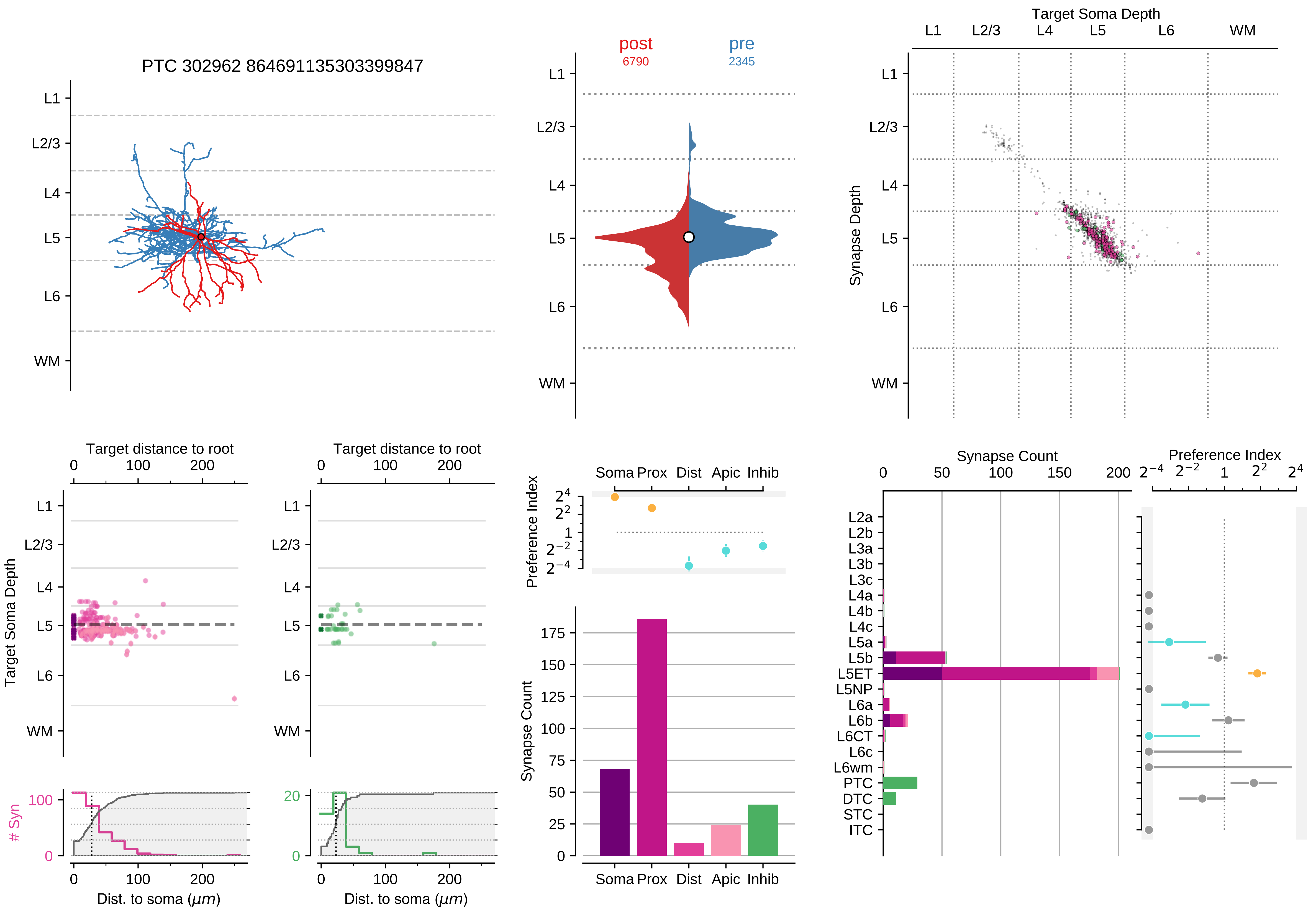

PTC 302962 864691135303399847
L1
L2/3
L4
L5
L6
WM
post
6790
pre
2345
Target Soma Depth
L1 L2/3 L4 L5 L6 WM
Synapse Depth
Target distance to root
0 100 200
Target Soma Depth
Syn
100
0
20
Dist. to soma (μm)
Preference Index
Soma Prox Dist Apic Inhib
2^4 2^2 1 2^{-2} 2^{-4}
Synapse Count
0 25 50 75 100 125 150 175
Synapse Count
0 50 100 150 200
Preference Index
L2a
L2b
L3a
L3b
L3c
L4a
L4b
L4c
L5a
L5b
L5ET
L5NP
L6a
L6b
L6CT
L6c
L6wm
PTC
DTC
STC
ITC

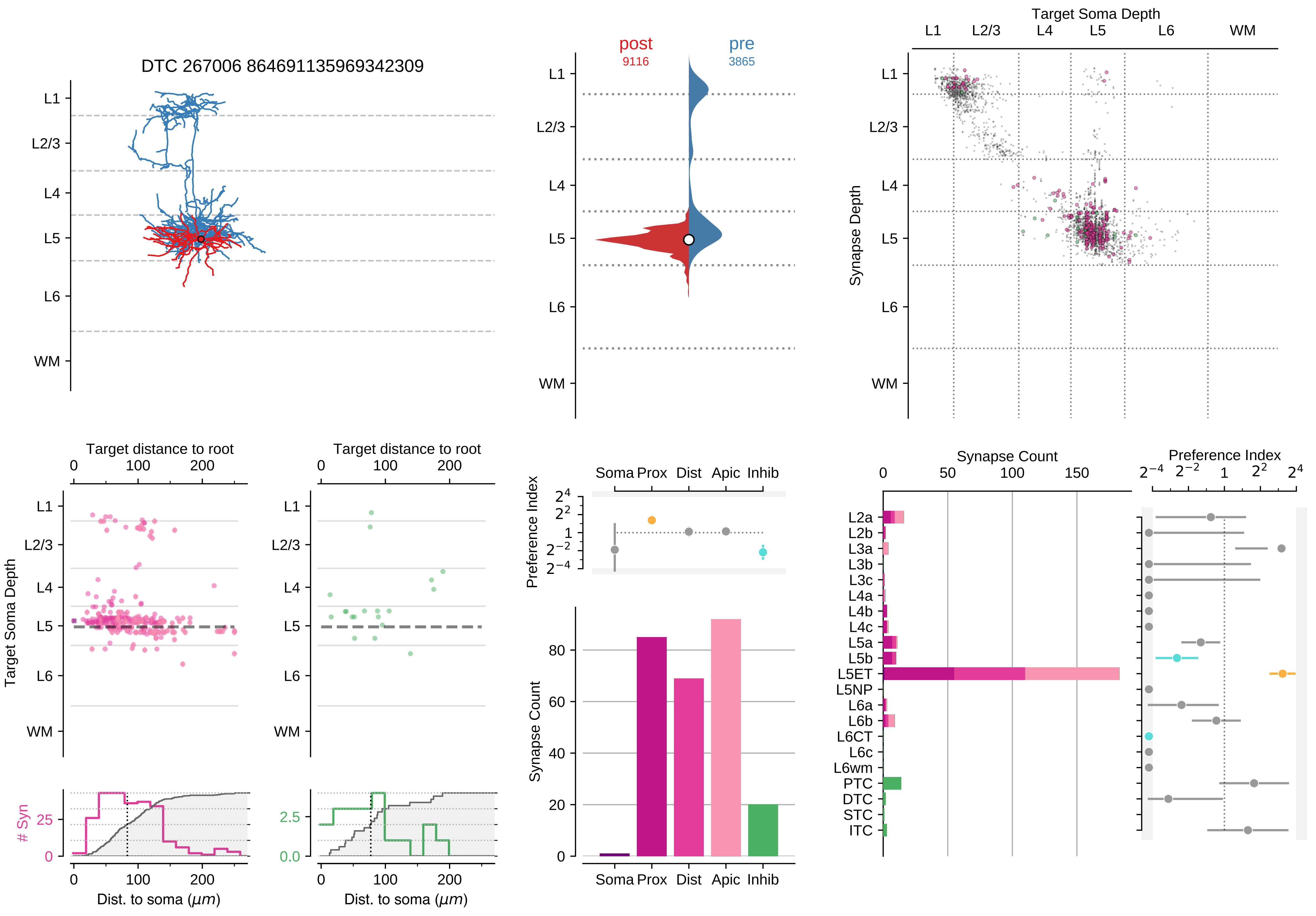

DTC 267006 864691135969342309
L1
L2/3
L4
L5
L6
WM
post
9116
pre
3865
Target Soma Depth
Synapse Depth
Target distance to root
Target Soma Depth
Syn
Dist. to soma (μm)
Preference Index
Soma
Prox
Dist
Apic
Inhib
Synapse Count
L2a
L2b
L3a
L3b
L3c
L4a
L4b
L4c
L5a
L5b
L5ET
L5NP
L6a
L6b
L6CT
L6c
L6wm
PTC
DTC
STC
ITC

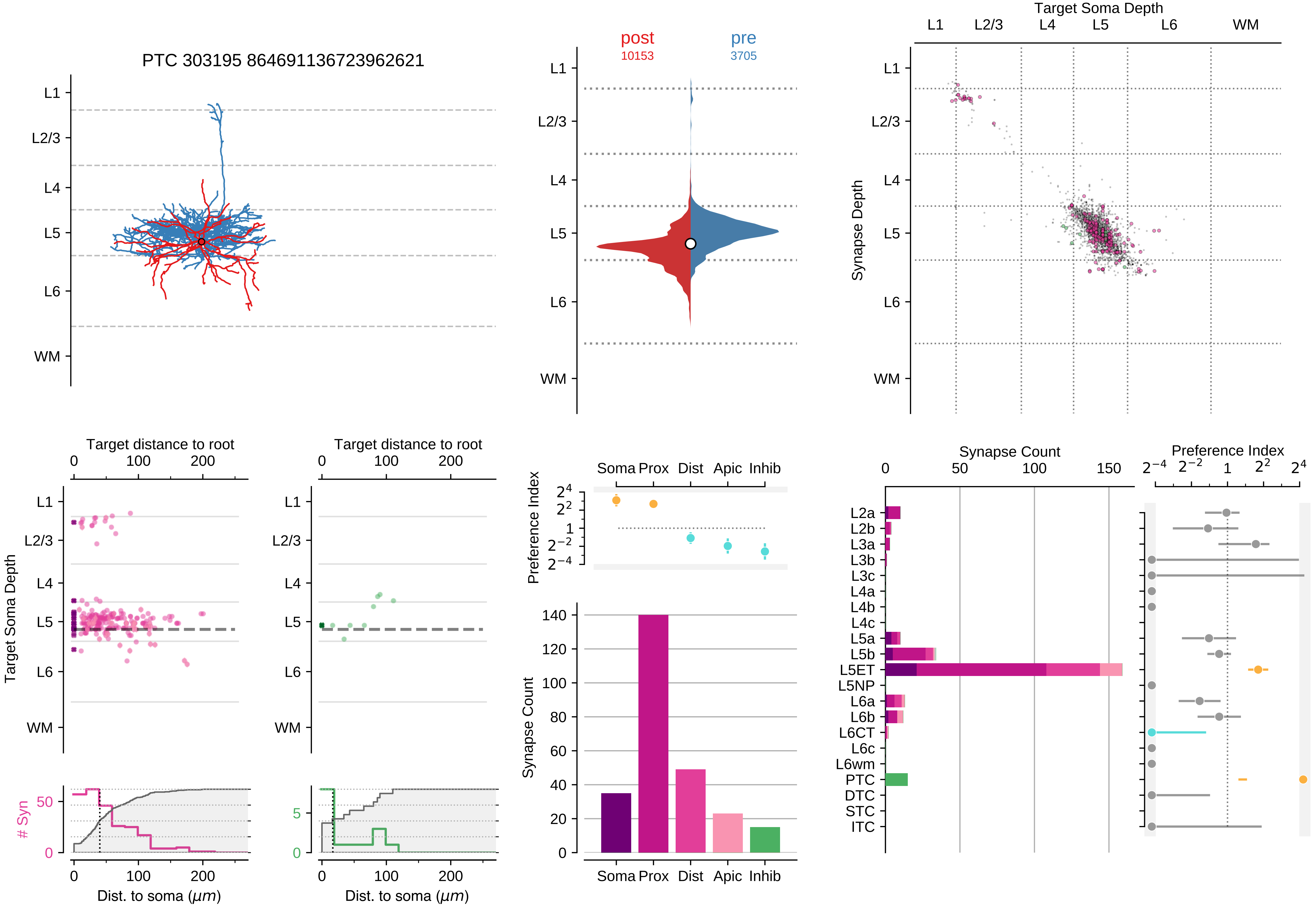

PTC 303195 864691136723962621
L1
L2/3
L4
L5
L6
WM
post
10153
pre
3705
Target Soma Depth
Synapse Depth
Target distance to root
Target Soma Depth
Syn
Dist. to soma (μm)
Preference Index
Soma Prox Dist Apic Inhib
Synapse Count
L2a
L2b
L3a
L3b
L3c
L4a
L4b
L4c
L5a
L5b
L5ET
L5NP
L6a
L6b
L6CT
L6c
L6wm
PTC
DTC
STC
ITC

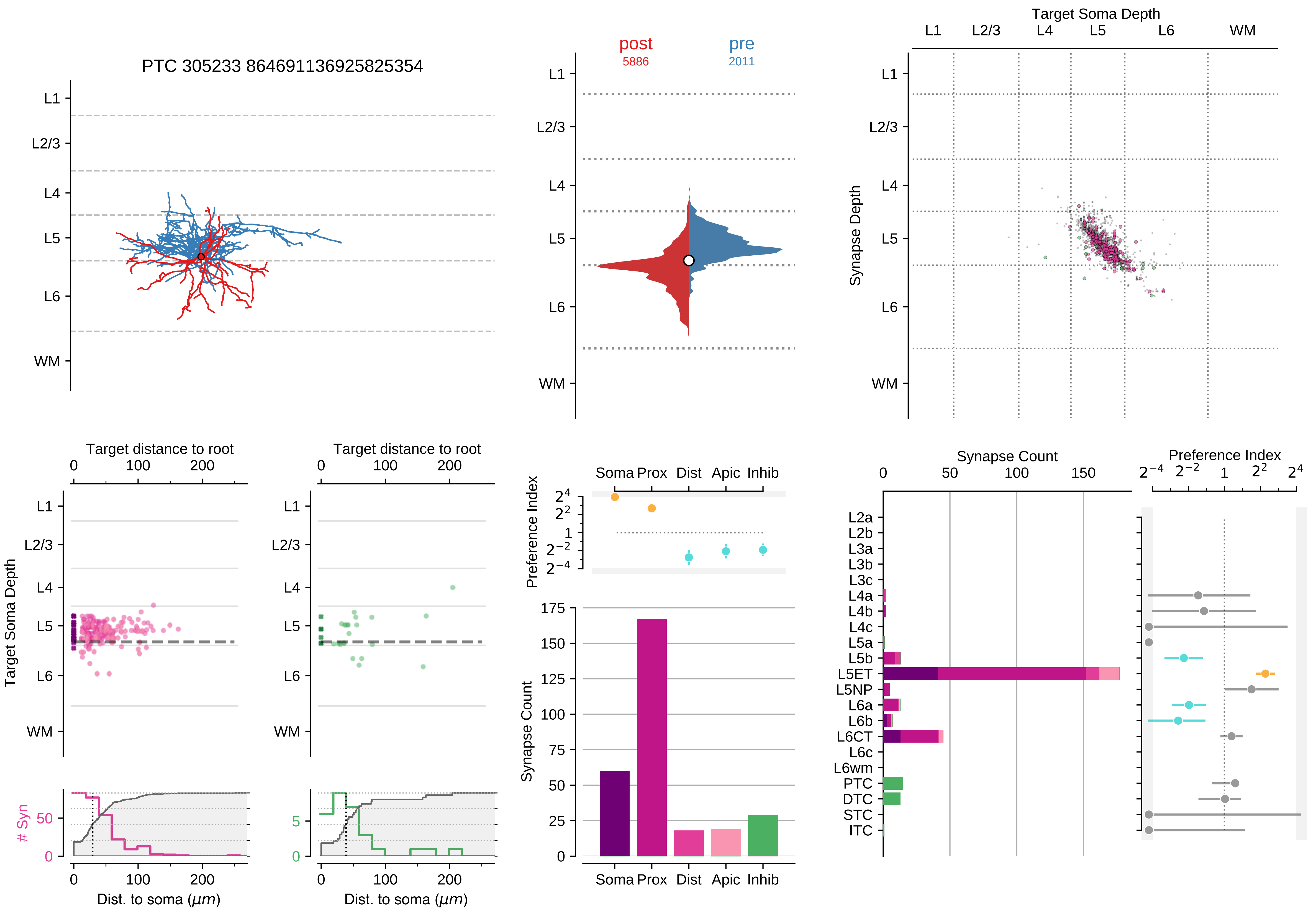

PTC 305233 864691136925825354
L1
L2/3
L4
L5
L6
WM
post
5886
pre
2011
Target Soma Depth
Synapse Depth
Target distance to root
Target Soma Depth
Syn
Dist. to soma (μm)
Preference Index
Soma Prox Dist Apic Inhib
Synapse Count
L2a
L2b
L3a
L3b
L3c
L4a
L4b
L4c
L5a
L5b
L5ET
L5NP
L6a
L6b
L6CT
L6c
L6wm
PTC
DTC
STC
ITC

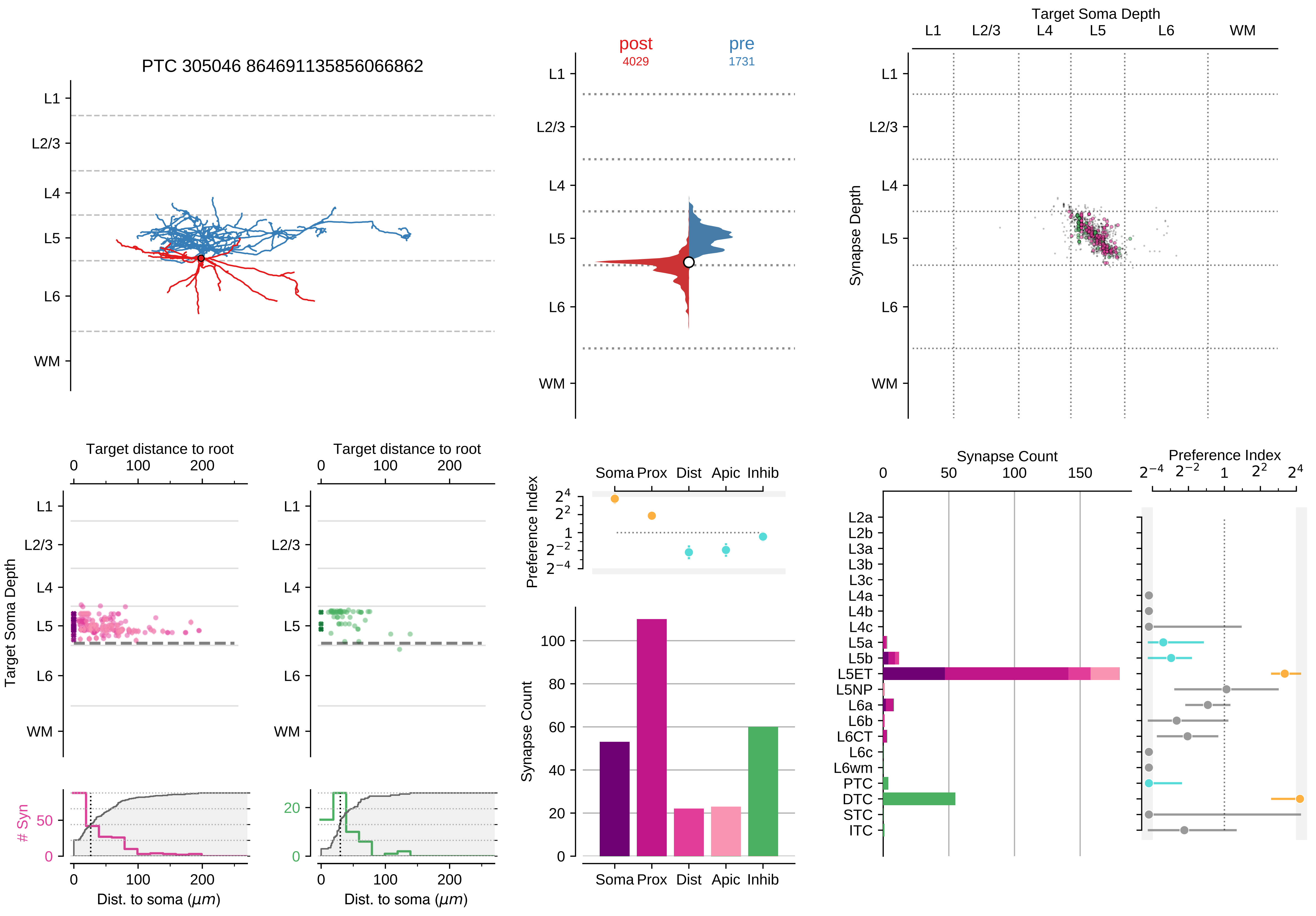

PTC 305046 864691135856066862
L1
L2/3
L4
L5
L6
WM
post
4029
pre
1731
Target Soma Depth
Synapse Depth
Target distance to root
Target Soma Depth
Syn
Dist. to soma (μm)
Preference Index
Soma Prox Dist Apic Inhib
Synapse Count
Synapse Count
Preference Index
L2a
L2b
L3a
L3b
L3c
L4a
L4b
L4c
L5a
L5b
L5ET
L5NP
L6a
L6b
L6CT
L6c
L6wm
PTC
DTC
STC
ITC

Group 13

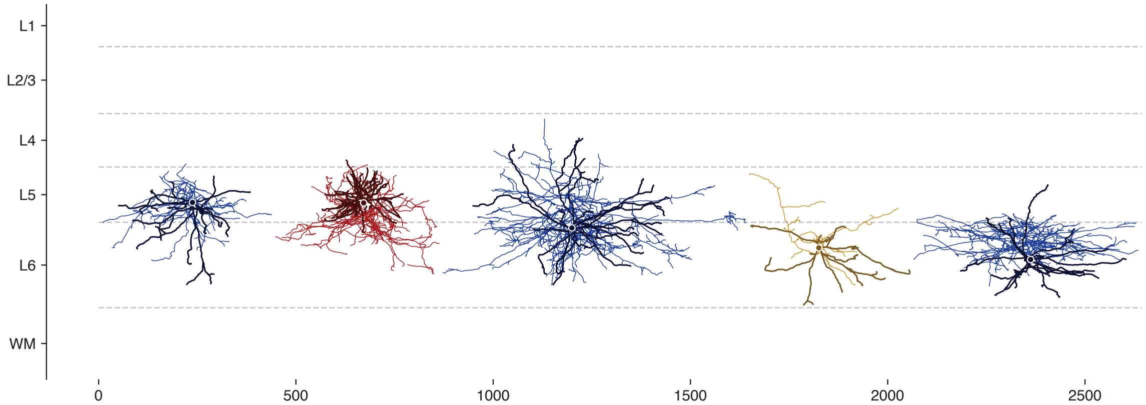

L1
L2/3
L4
L5
L6
WM
0
500
1000
1500
2000
2500

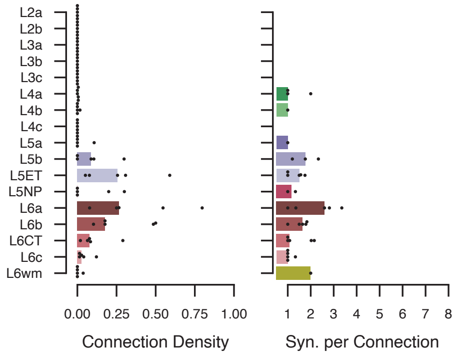

L2a
L2b
L3a
L3b
L3c
L4a
L4b
L4c
L5a
L5b
L5ET
L5NP
L6a
L6b
L6CT
L6c
L6wm
0.00
0.25
0.50
0.75
1.00
Connection Density
1
2
3
4
5
6
7
8
Syn. per Connection

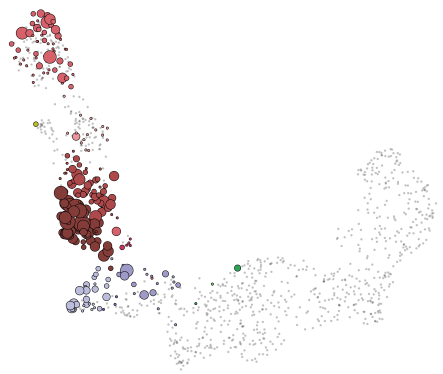

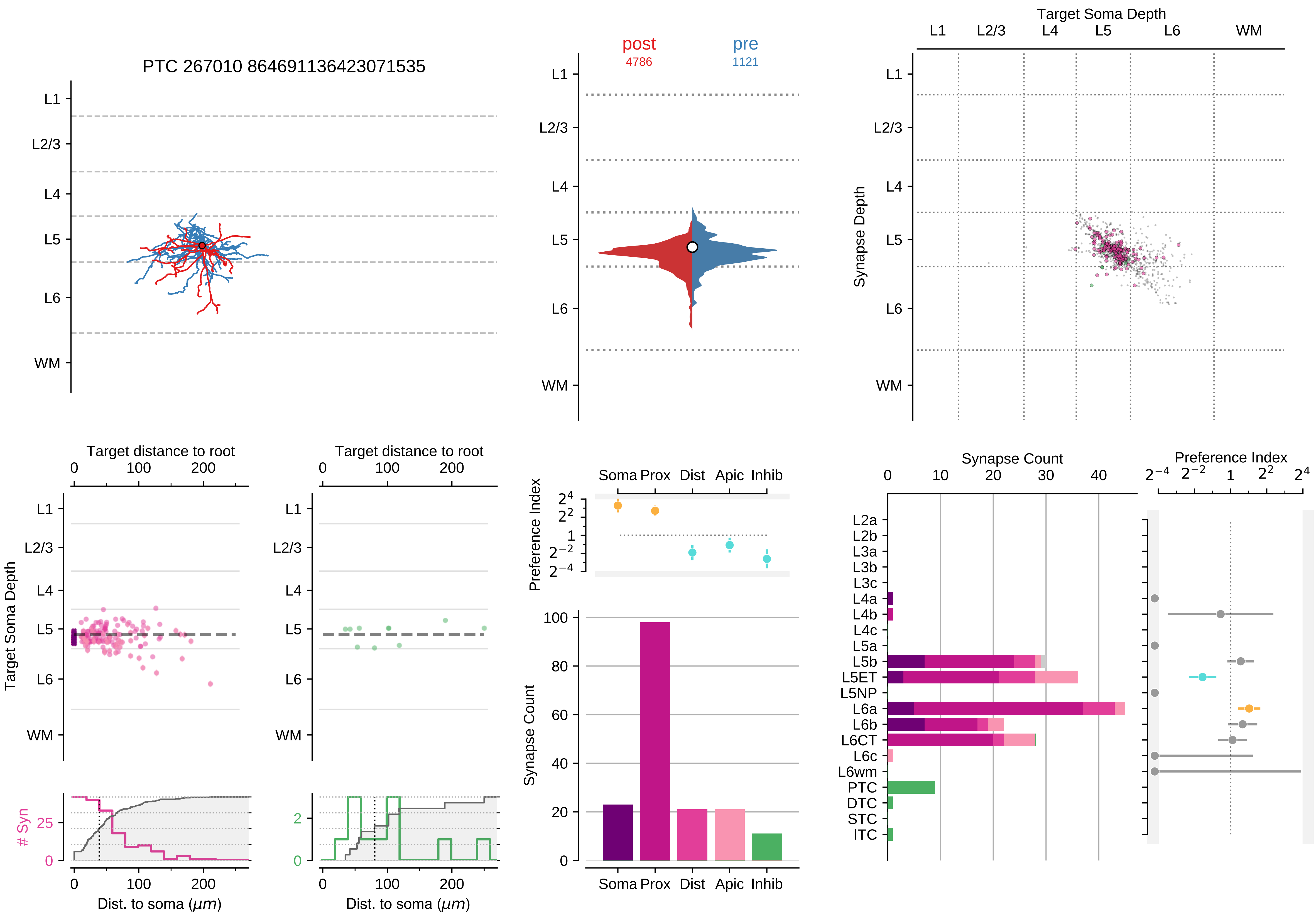

PTC 267010 864691136423071535
L1
L2/3
L4
L5
L6
WM
post
4786
pre
1121
Target Soma Depth
Synapse Depth
Target distance to root
Target Soma Depth
Syn
Dist. to soma (μm)
Preference Index
Soma
Prox
Dist
Apic
Inhib
Synapse Count
L2a
L2b
L3a
L3b
L3c
L4a
L4b
L4c
L5a
L5b
L5ET
L5NP
L6a
L6b
L6CT
L6c
L6wm
PTC
DTC
STC
ITC

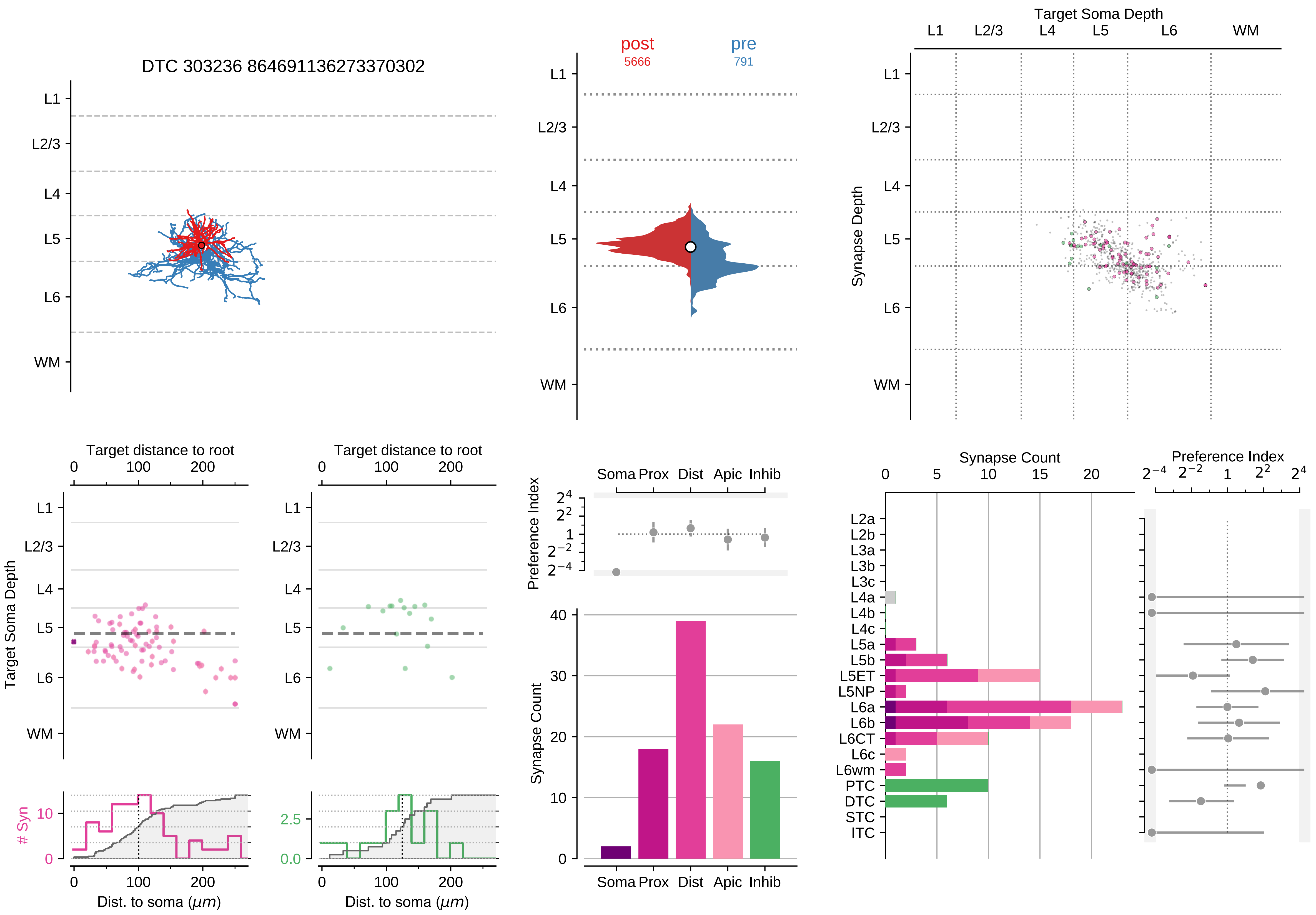

DTC 303236 864691136273370302
L1
L2/3
L4
L5
L6
WM
post
5666
pre
791
Target Soma Depth
Synapse Depth
Target distance to root
Target Soma Depth
Syn
Dist. to soma (μm)
Preference Index
Soma Prox Dist Apic Inhib
Synapse Count
L2a
L2b
L3a
L3b
L3c
L4a
L4b
L4c
L5a
L5b
L5ET
L5NP
L6a
L6b
L6CT
L6c
L6wm
PTC
DTC
STC
ITC

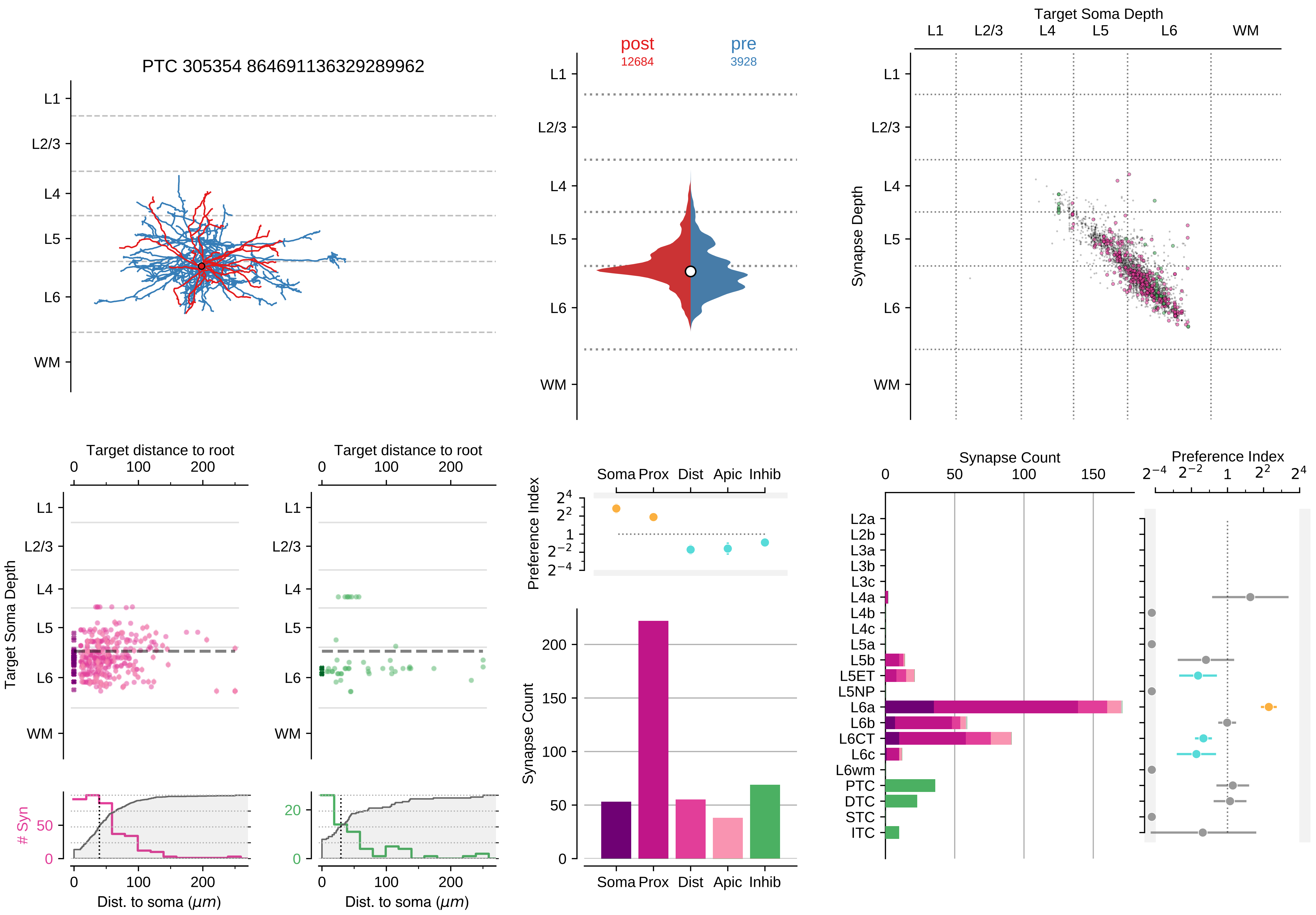

PTC 305354 864691136329289962
post
12684
pre
3928
Target Soma Depth
Synapse Depth
L1
L2/3
L4
L5
L6
WM
Target distance to root
Target Soma Depth
Syn
Dist. to soma (μm)
Preference Index
Soma Prox Dist Apic Inhib
Synapse Count
L2a
L2b
L3a
L3b
L3c
L4a
L4b
L4c
L5a
L5b
L5ET
L5NP
L6a
L6b
L6CT
L6c
L6wm
PTC
DTC
STC
ITC

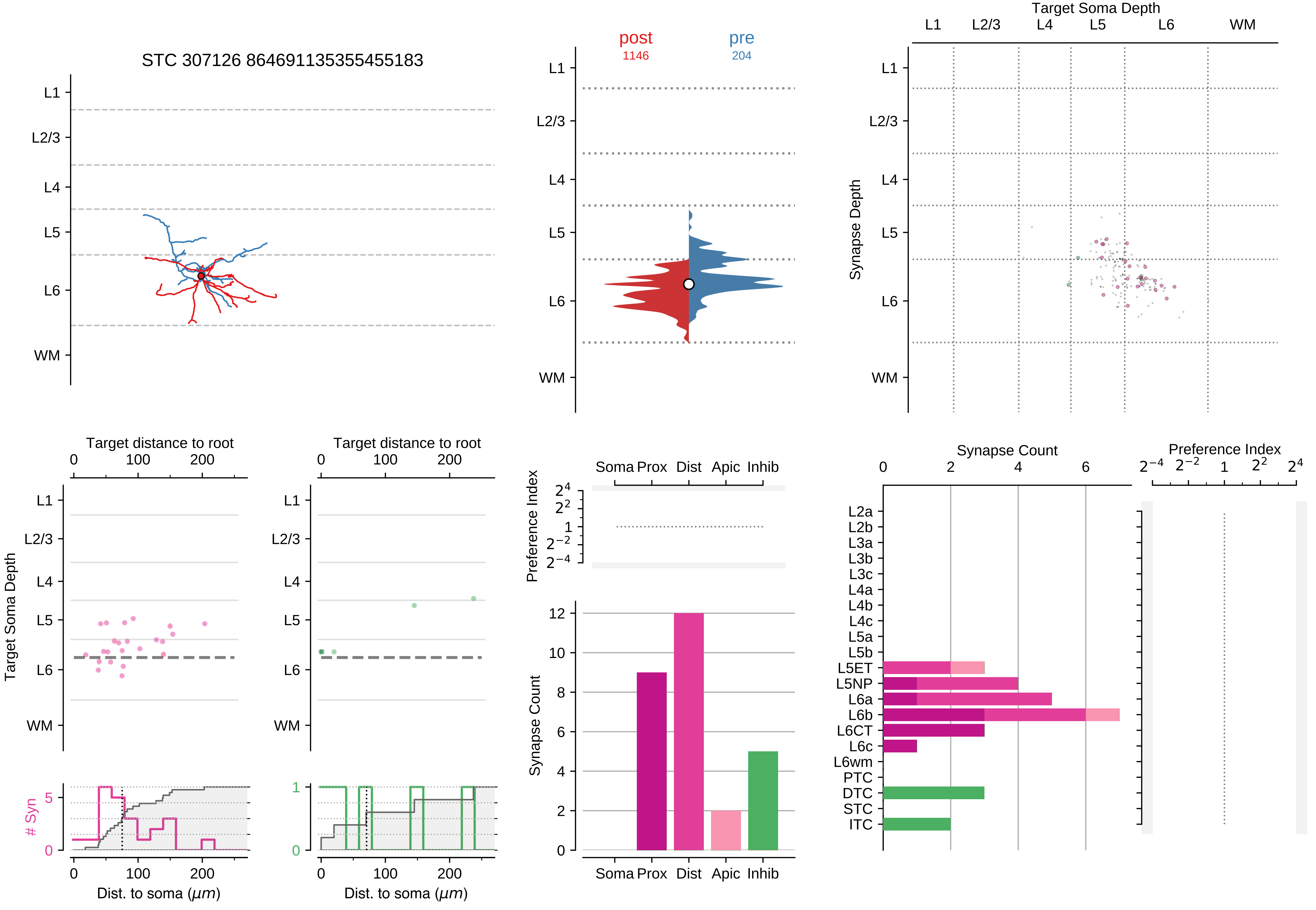

STC 307126 864691135355455183
L1
L2/3
L4
L5
L6
WM
post
1146
pre
204
Target Soma Depth
Synapse Depth
Target distance to root
Target Soma Depth
Dist. to soma (μm)
Syn
Preference Index
Soma Prox Dist Apic Inhib
Synapse Count
L2a
L2b
L3a
L3b
L3c
L4a
L4b
L4c
L5a
L5b
L5ET
L5NP
L6a
L6b
L6CT
L6c
L6wm
PTC
DTC
STC
ITC

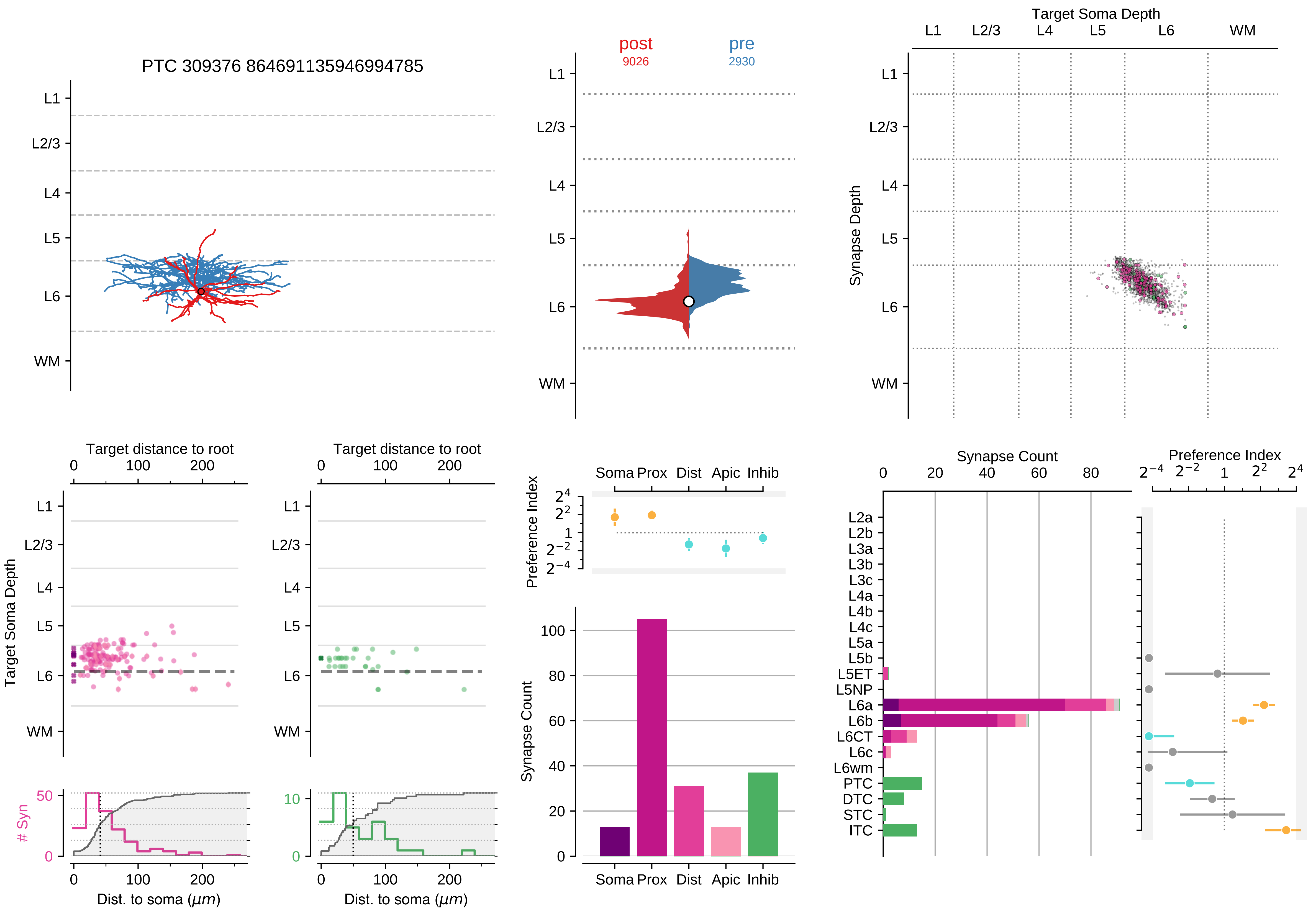

PTC 309376 864691135946994785
post
9026
pre
2930
Target Soma Depth
Synapse Depth
L1
L2/3
L4
L5
L6
WM
Target distance to root
Target Soma Depth
Syn
Dist. to soma (μm)
Preference Index
Soma Prox Dist Apic Inhib
Synapse Count
L2a
L2b
L3a
L3b
L3c
L4a
L4b
L4c
L5a
L5b
L5ET
L5NP
L6a
L6b
L6CT
L6c
L6wm
PTC
DTC
STC
ITC

Group 14

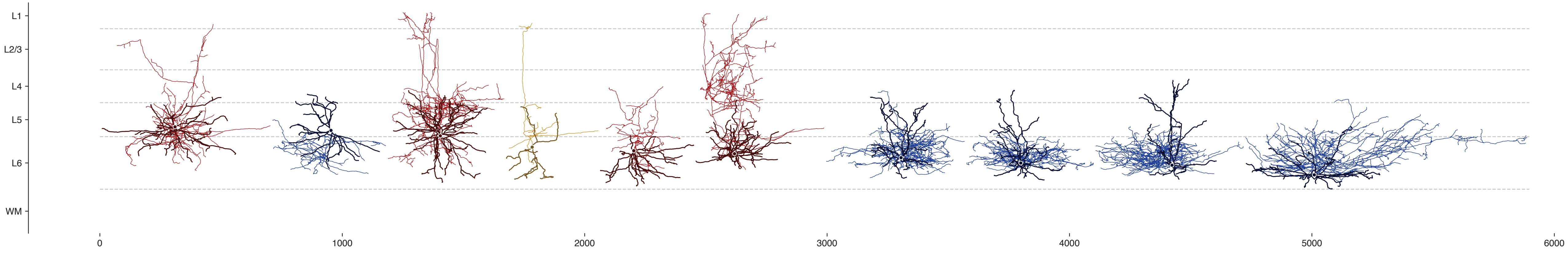

L1
L2/3
L4
L5
L6
WM
0
1000
2000
3000
4000
5000
6000

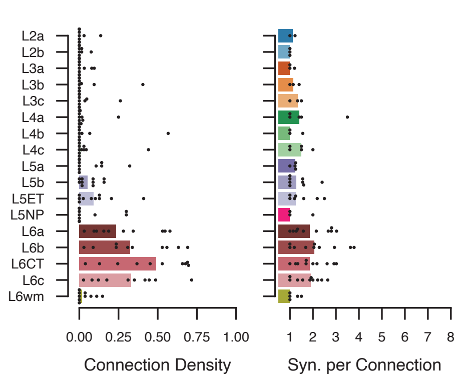

L2a
L2b
L3a
L3b
L3c
L4a
L4b
L4c
L5a
L5b
L5ET
L5NP
L6a
L6b
L6CT
L6c
L6wm
0.00 0.25 0.50 0.75 1.00
Connection Density
1 2 3 4 5 6 7 8
Syn. per Connection

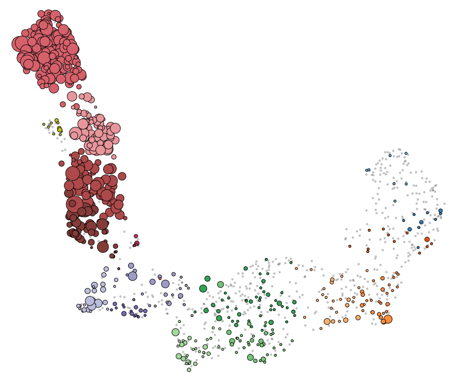

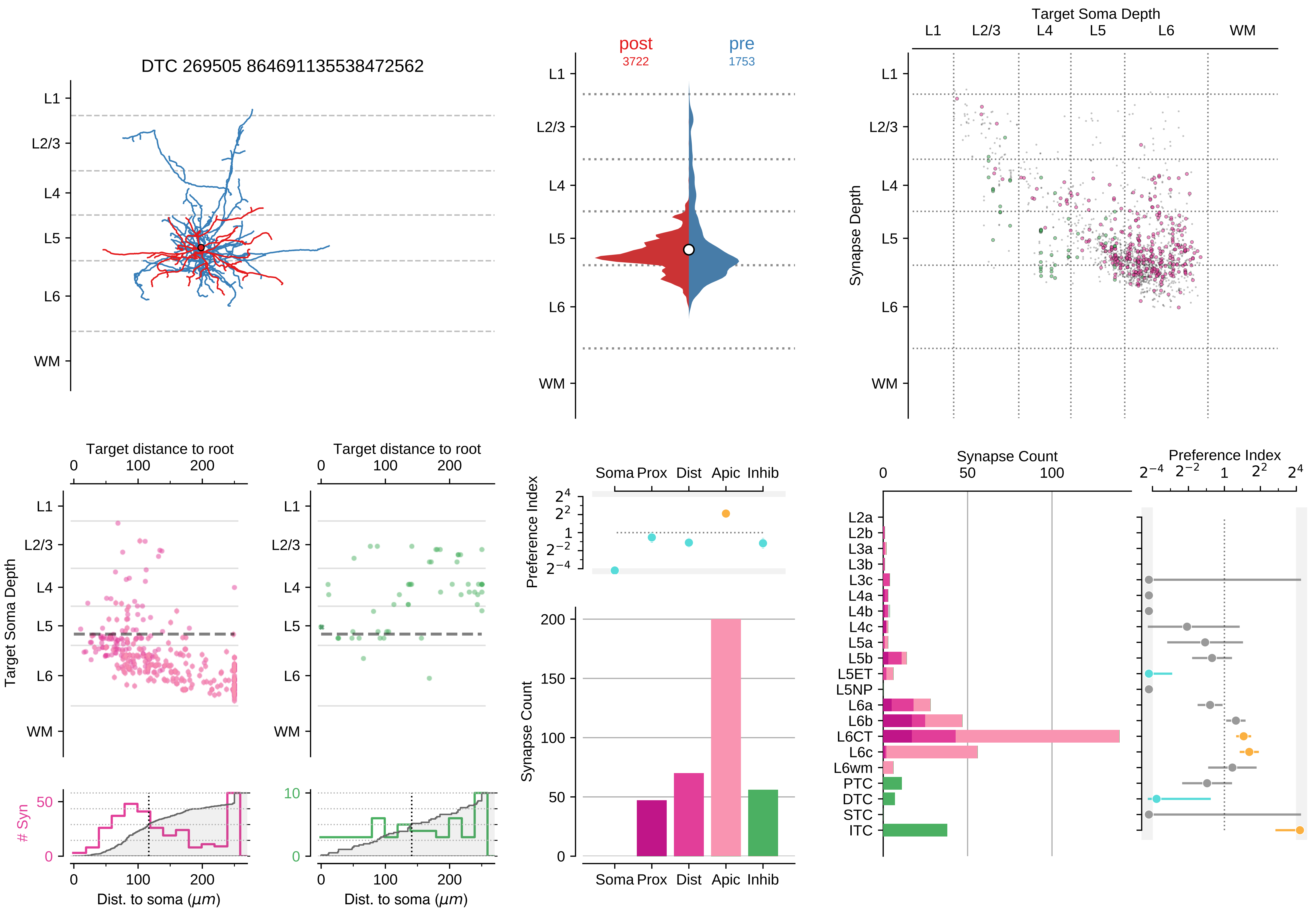

DTC 269505 864691135538472562
L1
L2/3
L4
L5
L6
WM
post
3722
pre
1753
Target Soma Depth
Synapse Depth
Target distance to root
Target Soma Depth
Syn
Dist. to soma (μm)
Preference Index
Soma
Prox
Dist
Apic
Inhib
Synapse Count
L2a
L2b
L3a
L3b
L3c
L4a
L4b
L4c
L5a
L5b
L5ET
L5NP
L6a
L6b
L6CT
L6c
L6wm
PTC
DTC
STC
ITC

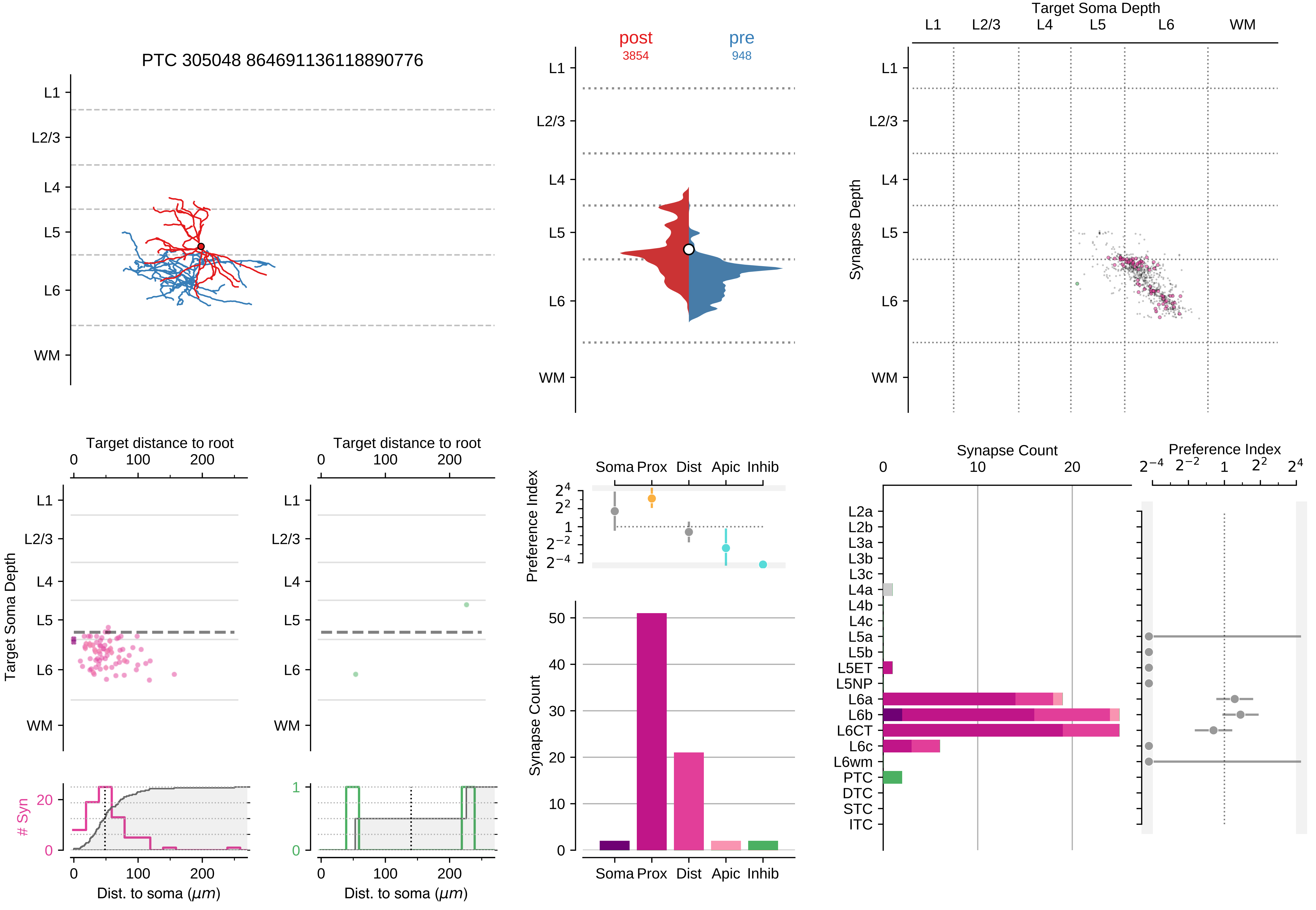

PTC 305048 864691136118890776
L1
L2/3
L4
L5
L6
WM
post
3854
pre
948
Target Soma Depth
Synapse Depth
Target distance to root
0
100
200
Target Soma Depth
Syn
Dist. to soma (μm)
Soma Prox Dist Apic Inhib
Preference Index
Synapse Count
Synapse Count
Preference Index
L2a
L2b
L3a
L3b
L3c
L4a
L4b
L4c
L5a
L5b
L5ET
L5NP
L6a
L6b
L6CT
L6c
L6wm
PTC
DTC
STC
ITC

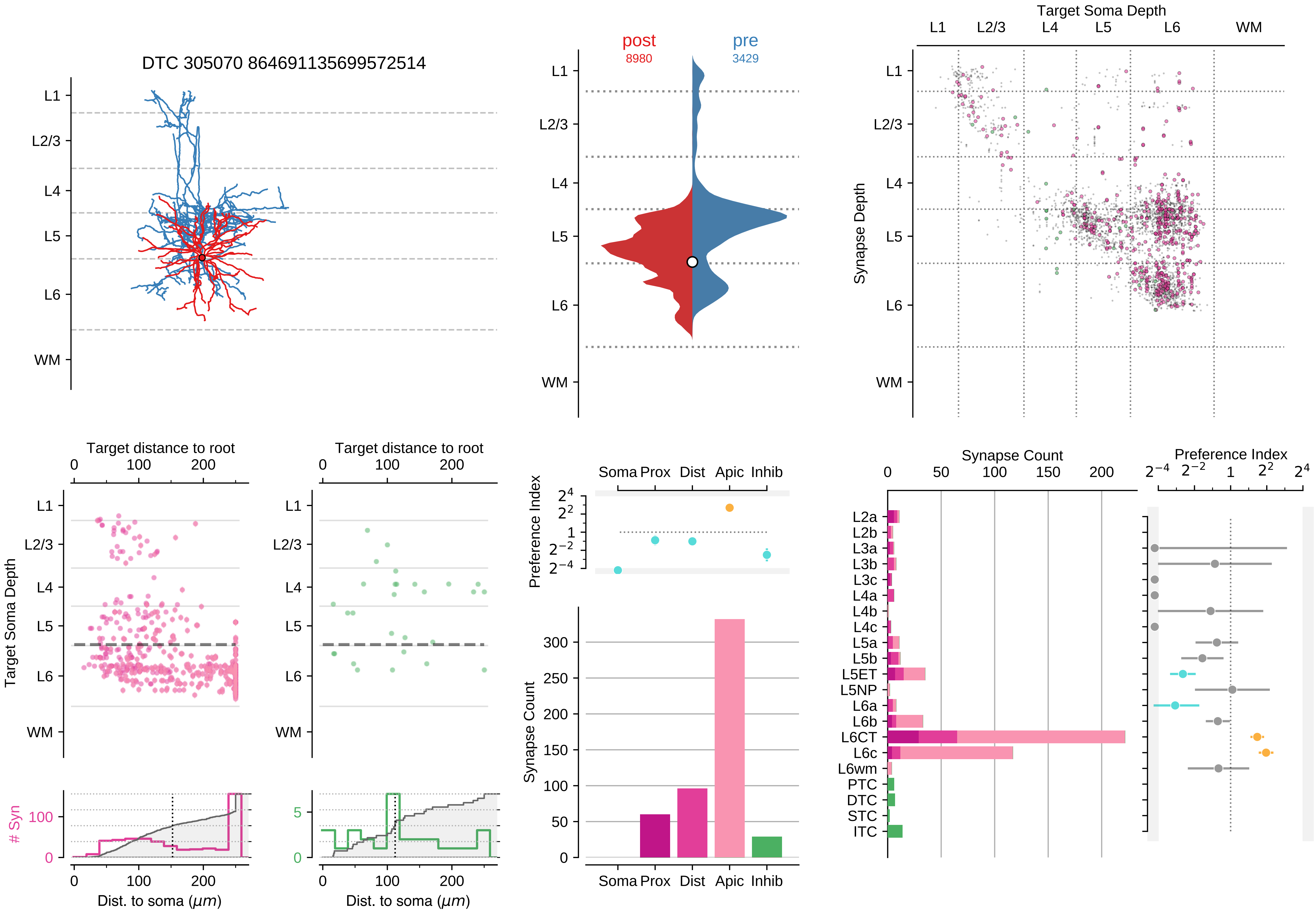

DTC 305070 864691135699572514
post
8980
pre
3429
Target Soma Depth
L1
L2/3
L4
L5
L6
WM
Synapse Depth
Target distance to root
Target Soma Depth
Syn
Dist. to soma (μm)
Preference Index
Soma Prox Dist Apic Inhib
Synapse Count
L2a
L2b
L3a
L3b
L3c
L4a
L4b
L4c
L5a
L5b
L5ET
L5NP
L6a
L6b
L6CT
L6c
L6wm
PTC
DTC
STC
ITC

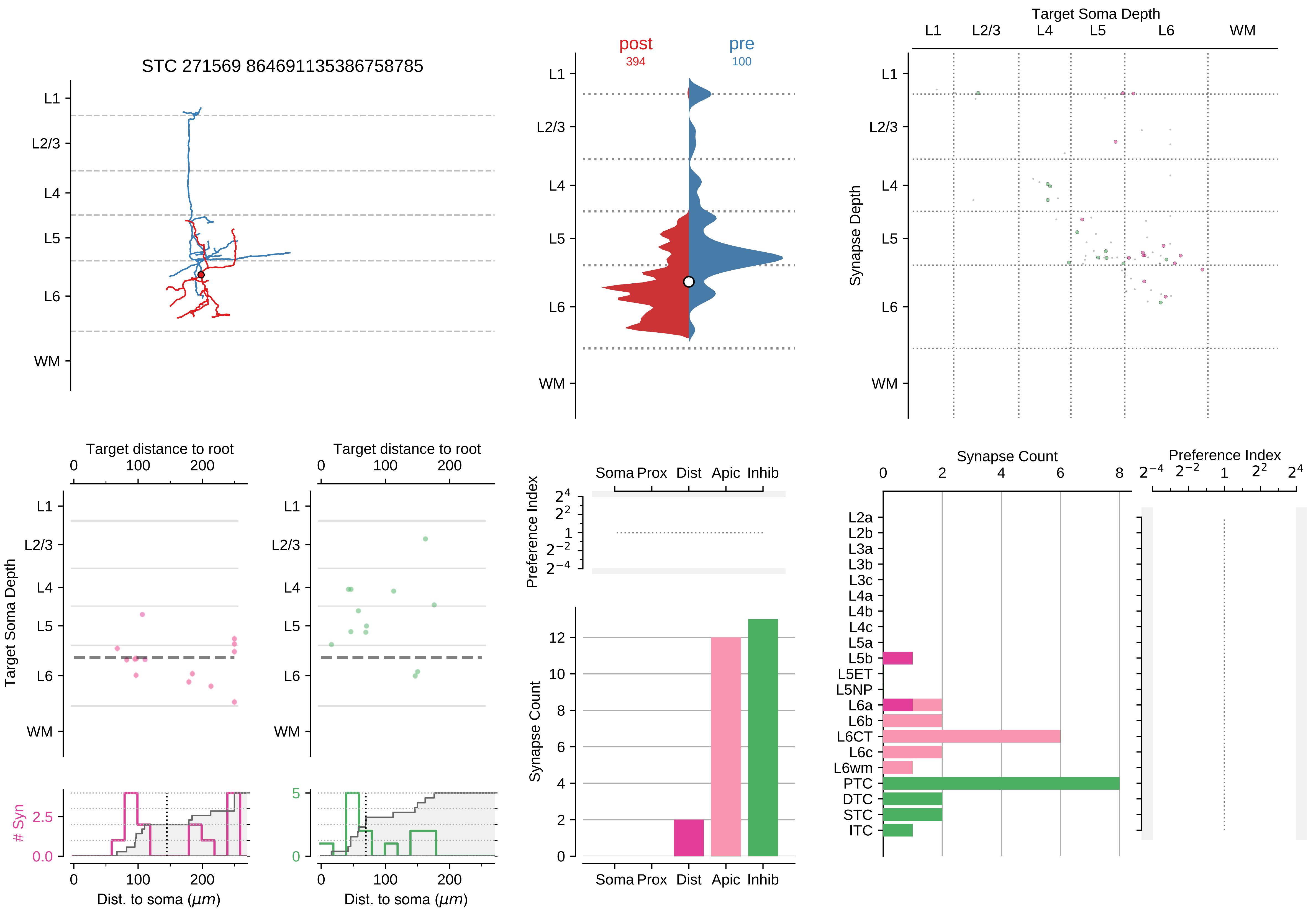

STC 271569 864691135386758785
post
394
pre
100
Target Soma Depth
Synapse Depth
L1
L2/3
L4
L5
L6
WM
Target distance to root
Target Soma Depth
Syn
Dist. to soma (μm)
Preference Index
Synapse Count
Soma
Prox
Dist
Apic
Inhib
L2a
L2b
L3a
L3b
L3c
L4a
L4b
L4c
L5a
L5b
L5ET
L5NP
L6a
L6b
L6CT
L6c
L6wm
PTC
DTC
STC
ITC

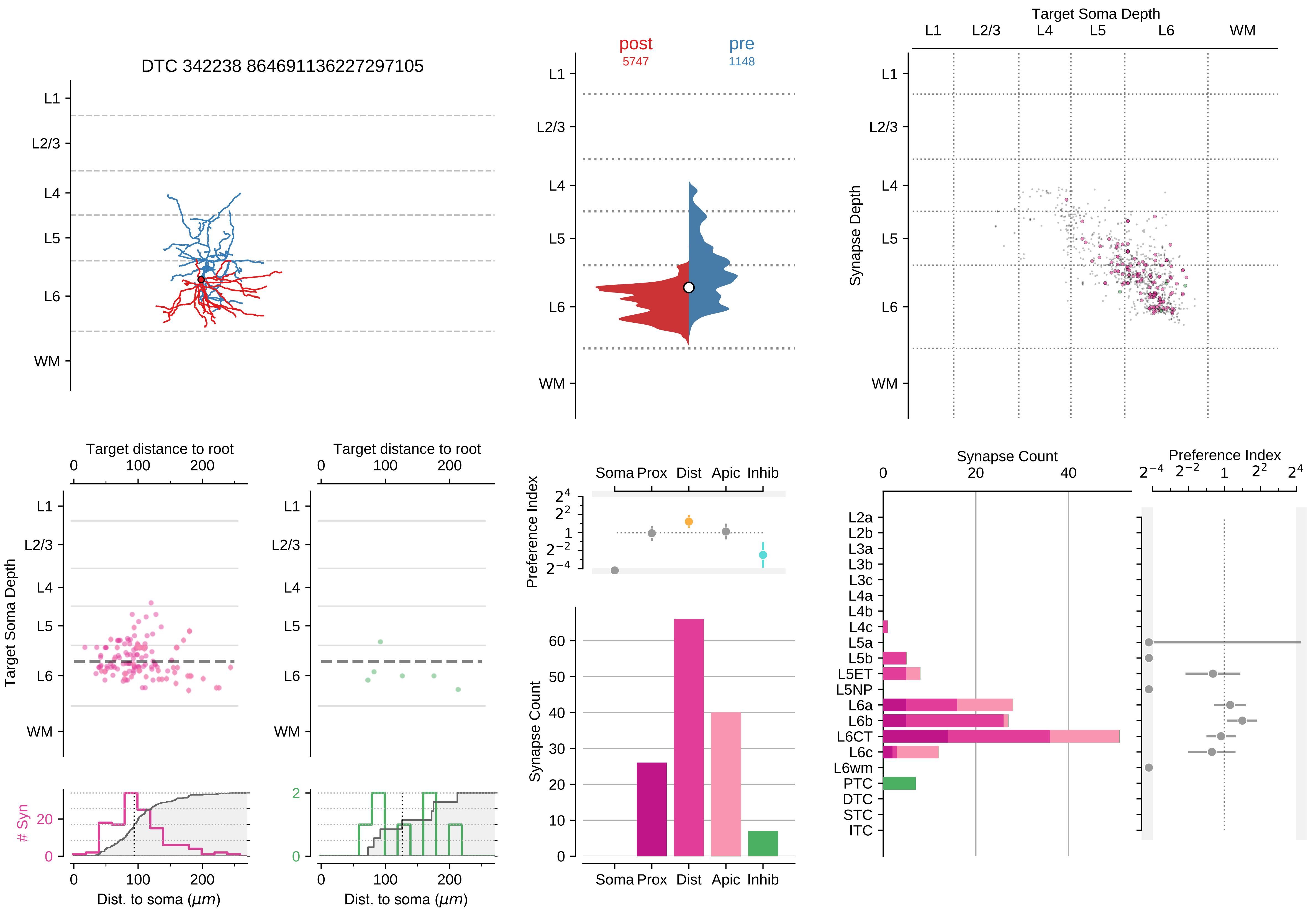

DTC 342238 864691136227297105
L1
L2/3
L4
L5
L6
WM
post
5747
pre
1148
Target Soma Depth
Synapse Depth
Target distance to root
Target Soma Depth
Syn
Dist. to soma (μm)
Soma Prox Dist Apic Inhib
Preference Index
Synapse Count
L2a
L2b
L3a
L3b
L3c
L4a
L4b
L4c
L5a
L5b
L5ET
L5NP
L6a
L6b
L6CT
L6c
L6wm
PTC
DTC
STC
ITC

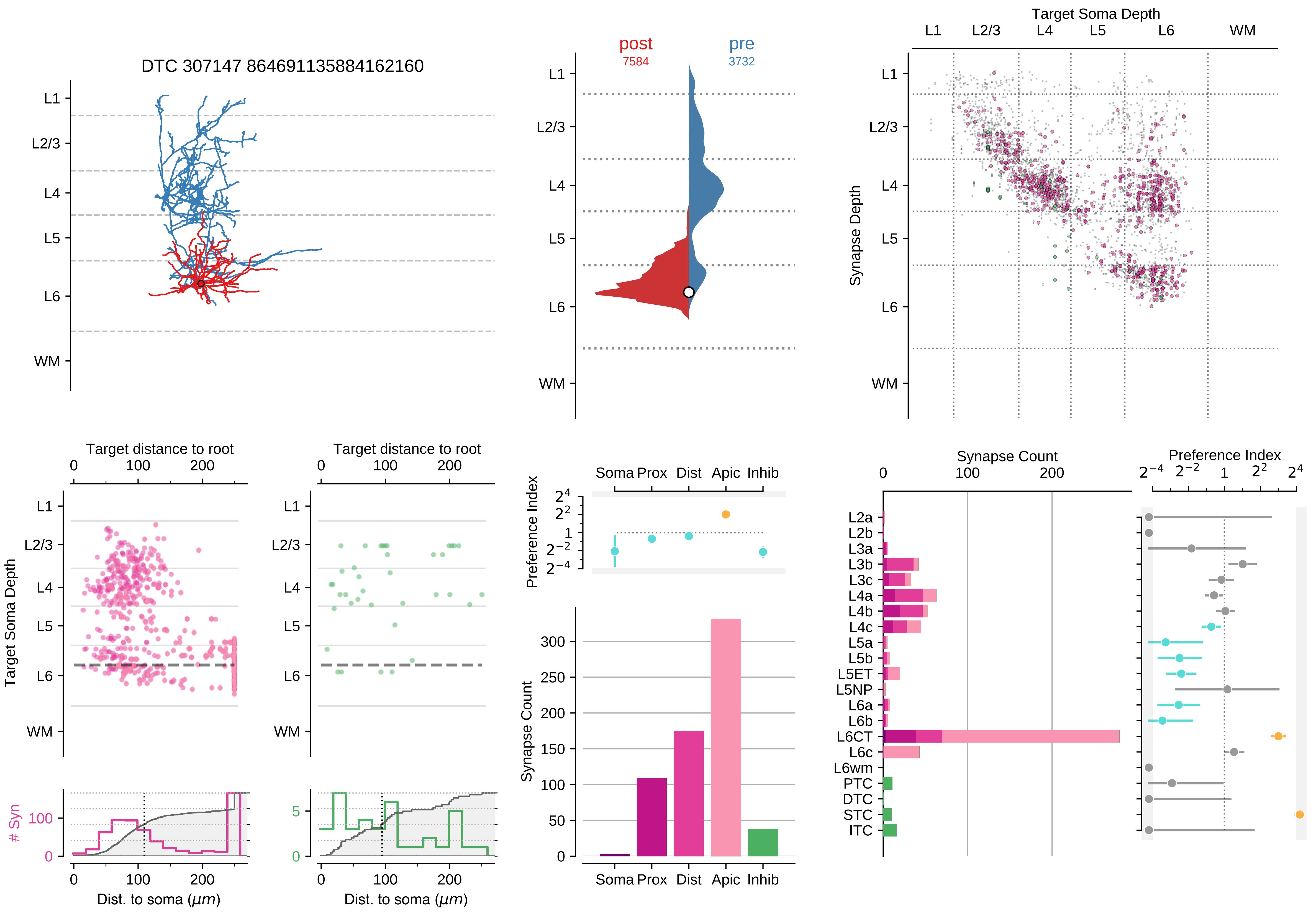

DTC 307147 864691135884162160
L1
L2/3
L4
L5
L6
WM
post
7584
pre
3732
Target Soma Depth
Synapse Depth
Target distance to root
Target Soma Depth
Syn
Dist. to soma (μm)
Preference Index
Soma
Prox
Dist
Apic
Inhib
Synapse Count
L2a
L2b
L3a
L3b
L3c
L4a
L4b
L4c
L5a
L5b
L5ET
L5NP
L6a
L6b
L6CT
L6c
L6wm
PTC
DTC
STC
ITC

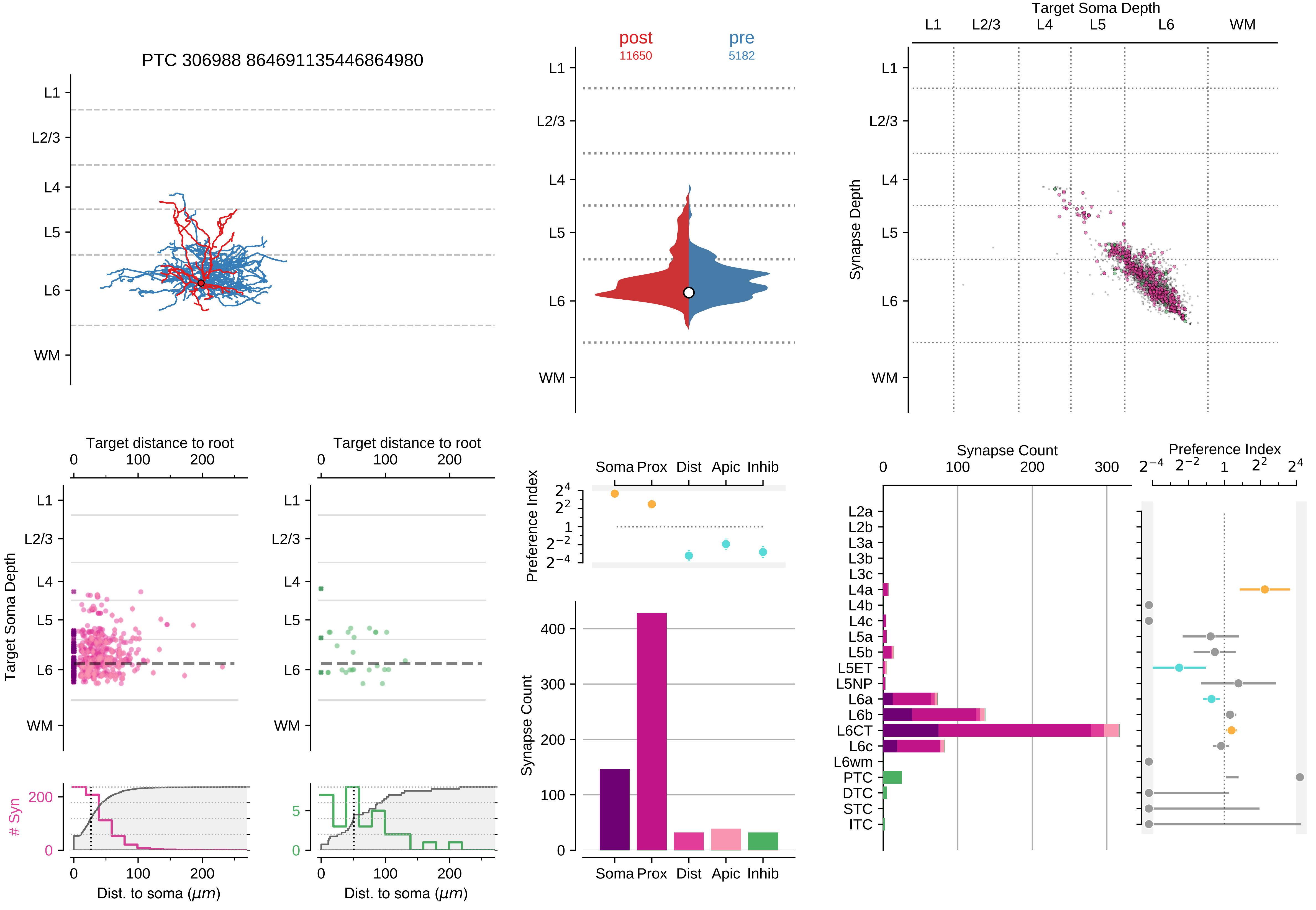
PTC 306988 864691135446864980
L1
L2/3
L4
L5
L6
WM
post
11650
pre
5182
Target Soma Depth
Synapse Depth
Target distance to root
Target Soma Depth
Syn
Dist. to soma (μm)
Preference Index
Soma
Prox
Dist
Apic
Inhib
Synapse Count
L2a
L2b
L3a
L3b
L3c
L4a
L4b
L4c
L5a
L5b
L5ET
L5NP
L6a
L6b
L6CT
L6c
L6wm
PTC
DTC
STC
ITC

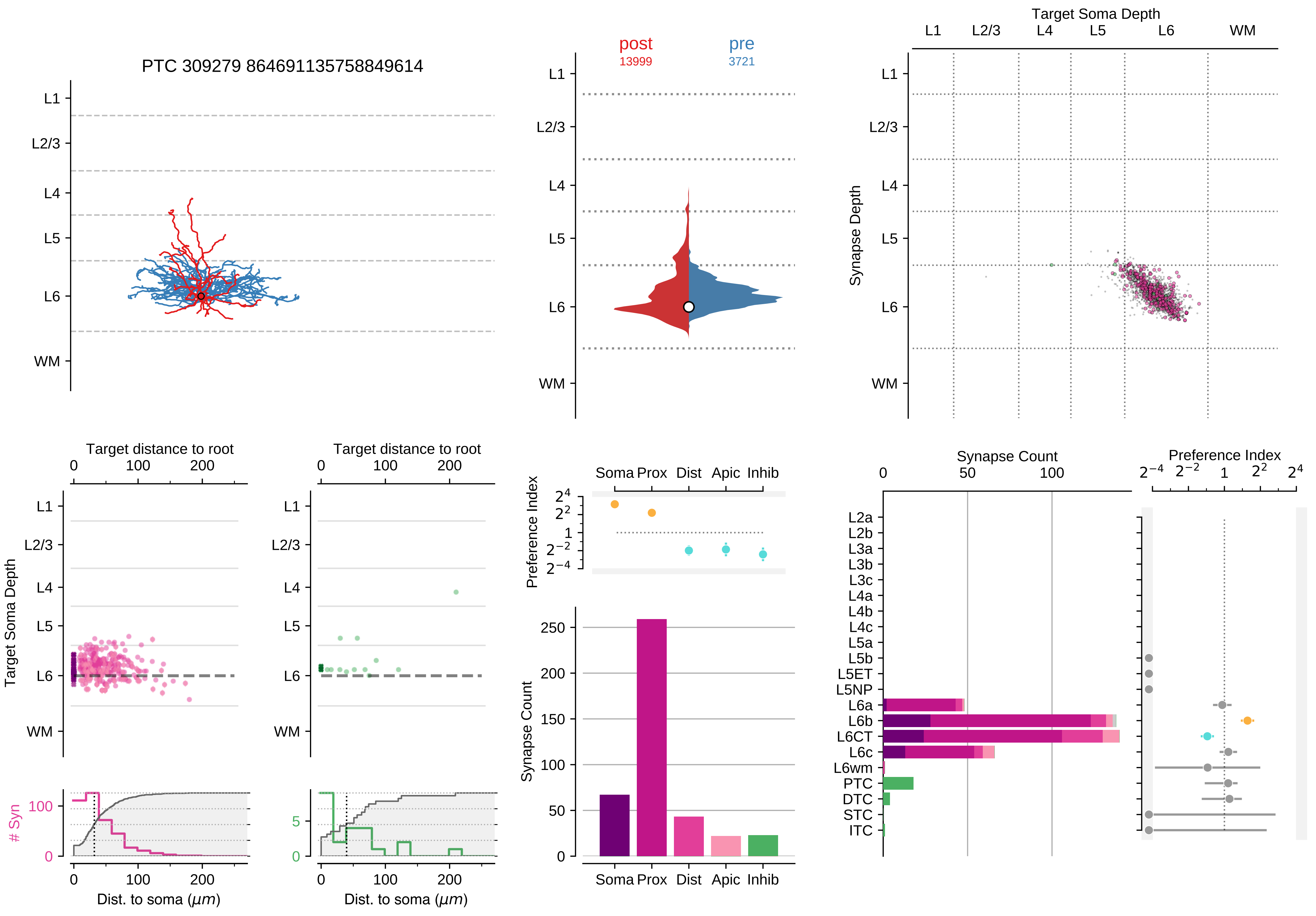

PTC 309279 864691135758849614
L1
L2/3
L4
L5
L6
WM
post
13999
pre
3721
Target Soma Depth
Synapse Depth
Target distance to root
Target Soma Depth
Syn
Dist. to soma (μm)
Preference Index
Soma Prox Dist Apic Inhib
Synapse Count
Preference Index
L2a
L2b
L3a
L3b
L3c
L4a
L4b
L4c
L5a
L5b
L5ET
L5NP
L6a
L6b
L6CT
L6c
L6wm
PTC
DTC
STC
ITC

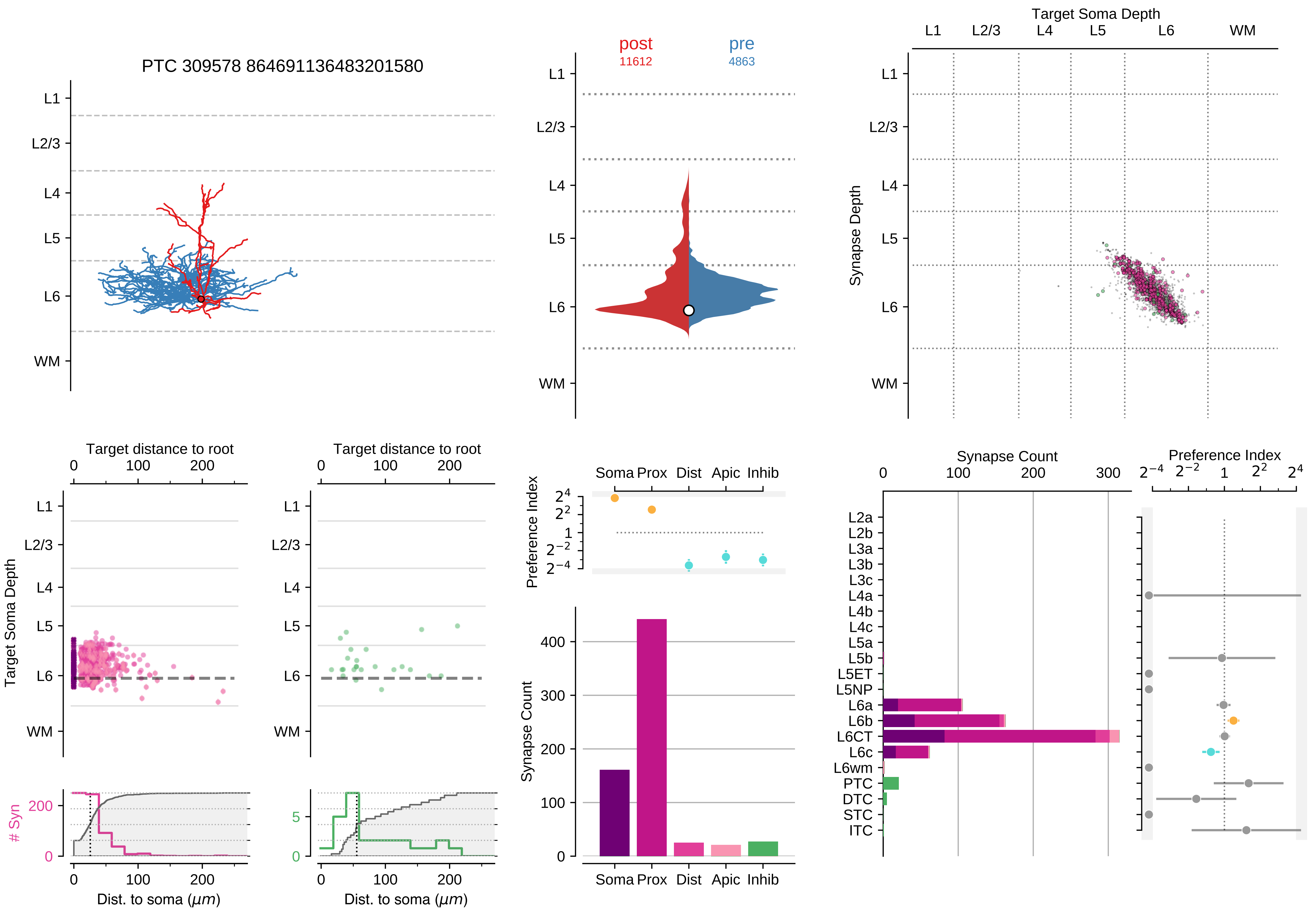

PTC 309578 864691136483201580
post
11612
pre
4863
Target Soma Depth
L1
L2/3
L4
L5
L6
WM
Synapse Depth
Target distance to root
Target Soma Depth
Dist. to soma (μm)
Syn
Preference Index
Soma Prox Dist Apic Inhib
Synapse Count
L2a
L2b
L3a
L3b
L3c
L4a
L4b
L4c
L5a
L5b
L5ET
L5NP
L6a
L6b
L6CT
L6c
L6wm
PTC
DTC
STC
ITC

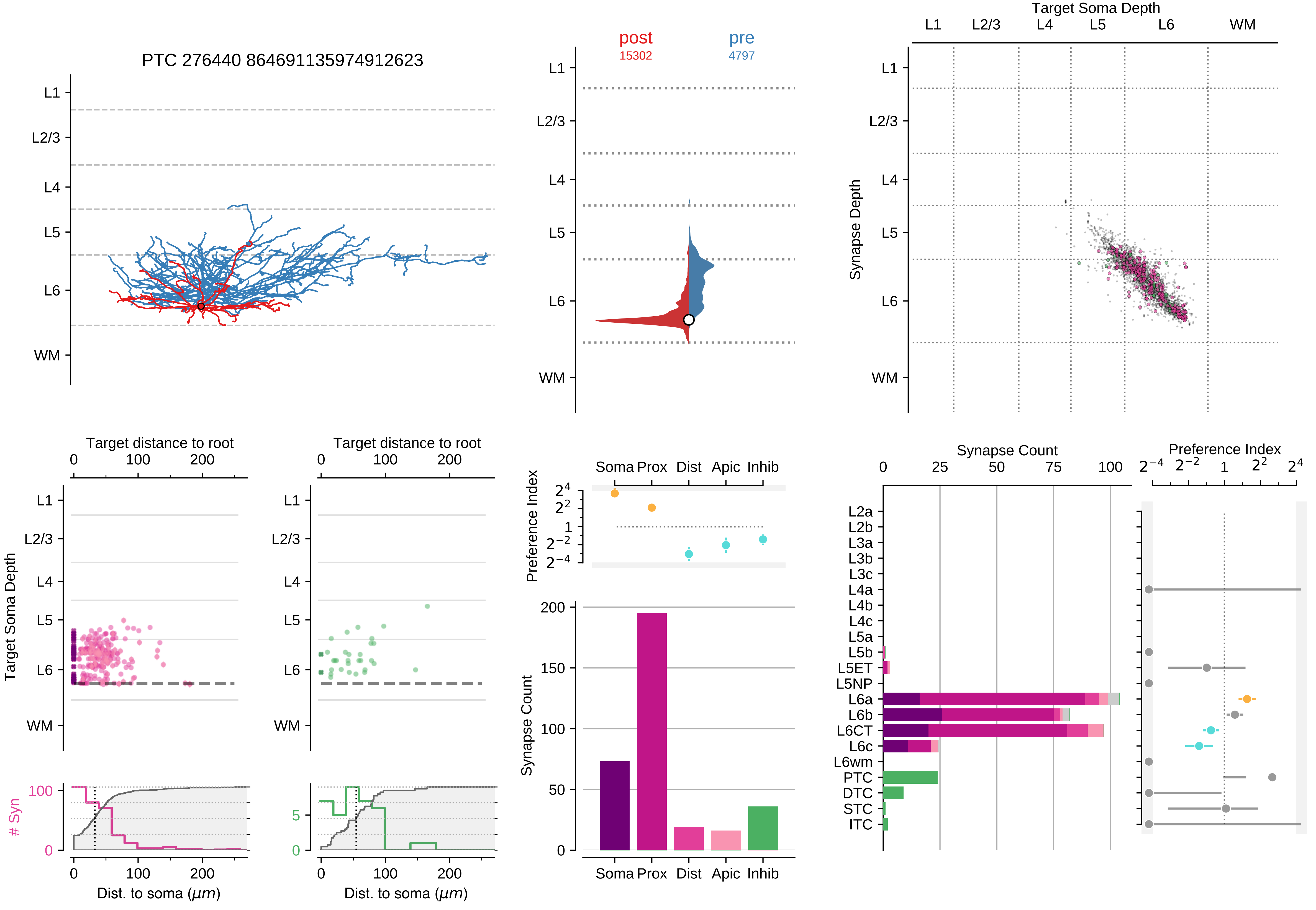

PTC 276440 864691135974912623
L1
L2/3
L4
L5
L6
WM
post
15302
pre
4797
Target Soma Depth
Synapse Depth
Target distance to root
Target Soma Depth
Dist. to soma (μm)
Syn
Preference Index
Soma
Prox
Dist
Apic
Inhib
Synapse Count
L2a
L2b
L3a
L3b
L3c
L4a
L4b
L4c
L5a
L5b
L5ET
L5NP
L6a
L6b
L6CT
L6c
L6wm
PTC
DTC
STC
ITC

Group 15

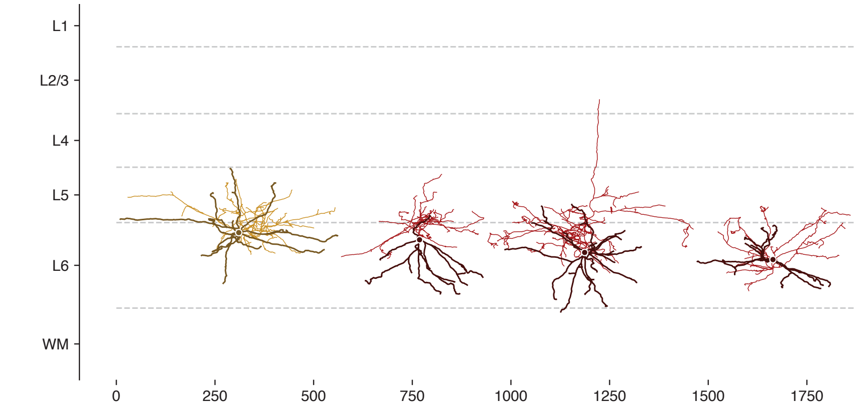
L1
L2/3
L4
L5
L6
WM
0
250
500
750
1000
1250
1500
1750

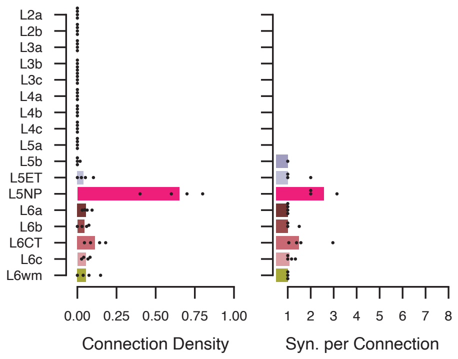
L2a
L2b
L3a
L3b
L3c
L4a
L4b
L4c
L5a
L5b
L5ET
L5NP
L6a
L6b
L6CT
L6c
L6wm
0.00
0.25
0.50
0.75
1.00
Connection Density
1
2
3
4
5
6
7
8
Syn. per Connection

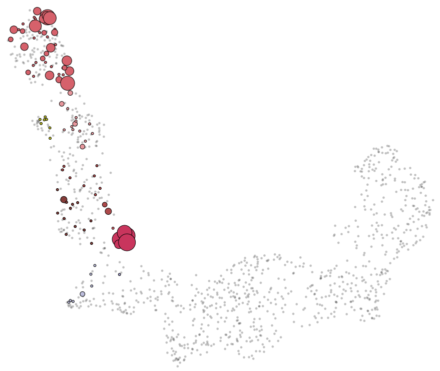

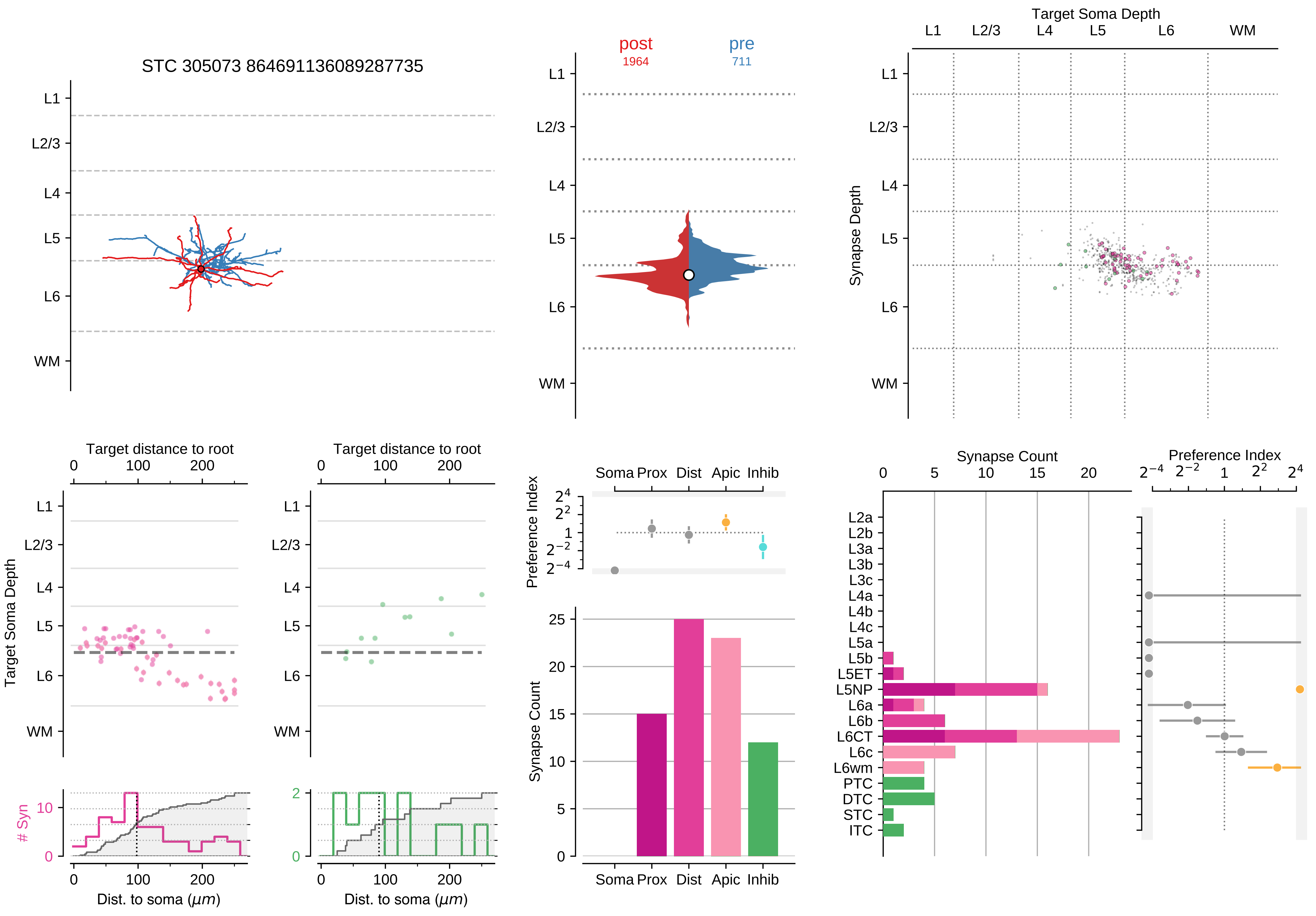

STC 305073 864691136089287735
L1
L2/3
L4
L5
L6
WM
post
1964
pre
711
Target Soma Depth
Synapse Depth
Target distance to root
Target Soma Depth
Syn
Dist. to soma (μm)
Preference Index
Soma Prox Dist Apic Inhib
Synapse Count
L2a
L2b
L3a
L3b
L3c
L4a
L4b
L4c
L5a
L5b
L5ET
L5NP
L6a
L6b
L6CT
L6c
L6wm
PTC
DTC
STC
ITC

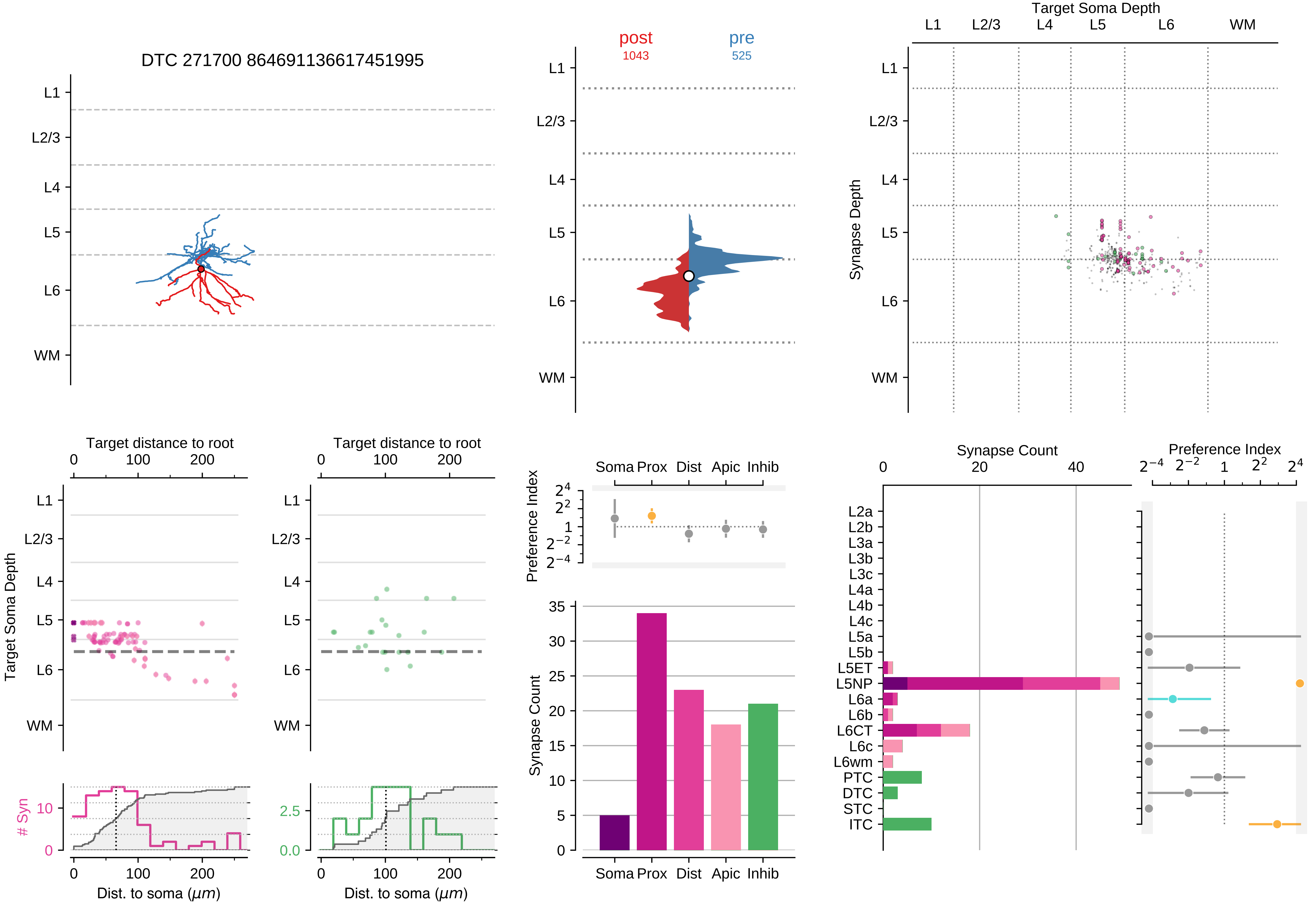
DTC 271700 864691136617451995
L1
L2/3
L4
L5
L6
WM
post
1043
pre
525
Target Soma Depth
Synapse Depth
Target distance to root
Target Soma Depth
Dist. to soma (μm)
Syn
Preference Index
Soma Prox Dist Apic Inhib
Synapse Count
L2a
L2b
L3a
L3b
L3c
L4a
L4b
L4c
L5a
L5b
L5ET
L5NP
L6a
L6b
L6CT
L6c
L6wm
PTC
DTC
STC
ITC

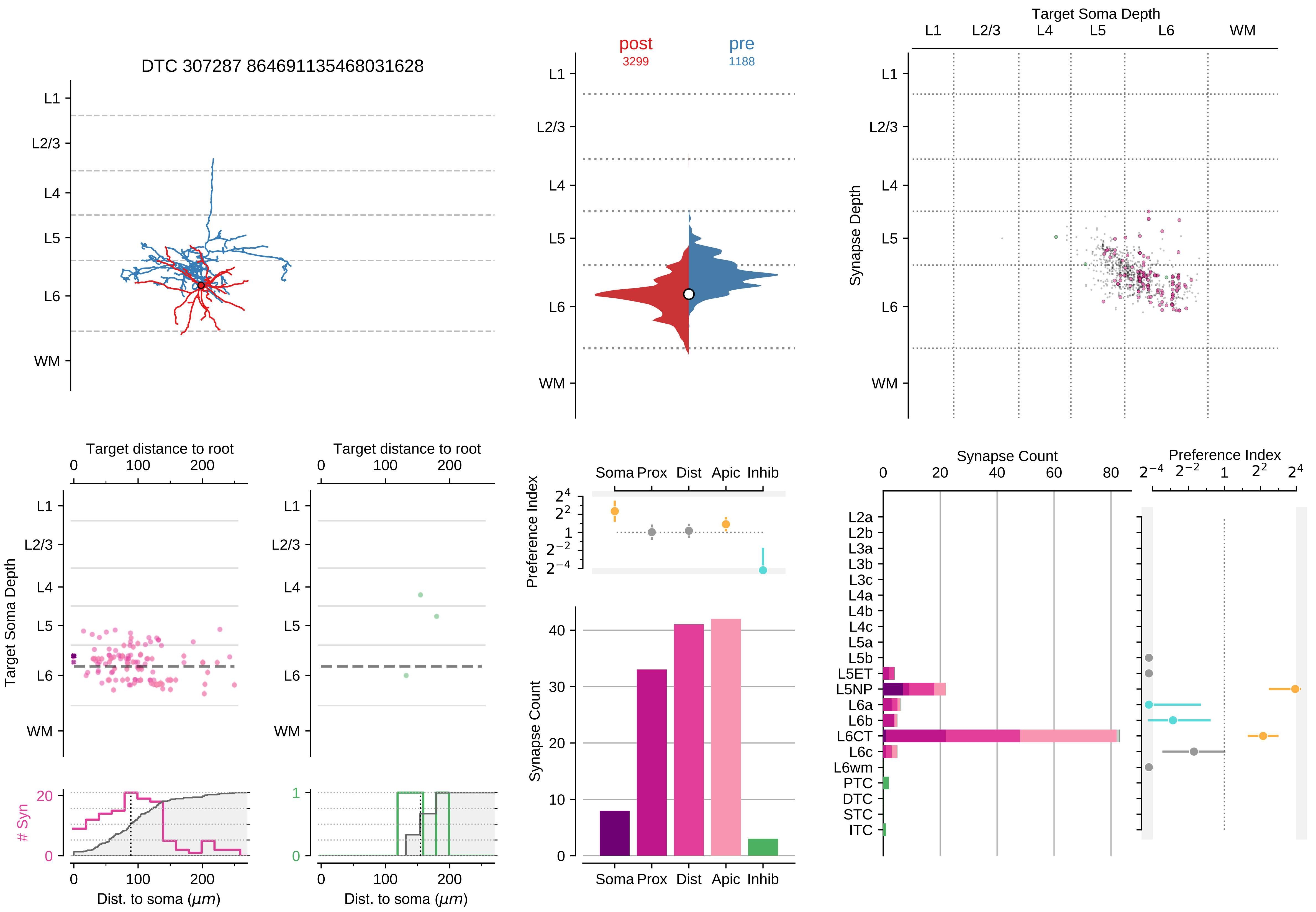

DTC 307287 864691135468031628
L1
L2/3
L4
L5
L6
WM
post
3299
pre
1188
Target Soma Depth
Synapse Depth
Target distance to root
0
100
200
Target Soma Depth
Syn
20
Dist. to soma (μm)
Preference Index
Soma
Prox
Dist
Apic
Inhib
Synapse Count
40
30
10
60
80
L2a
L2b
L3a
L3b
L3c
L4a
L4b
L4c
L5a
L5b
L5ET
L5NP
L6a
L6b
L6CT
L6c
L6wm
PTC
DTC
STC
ITC

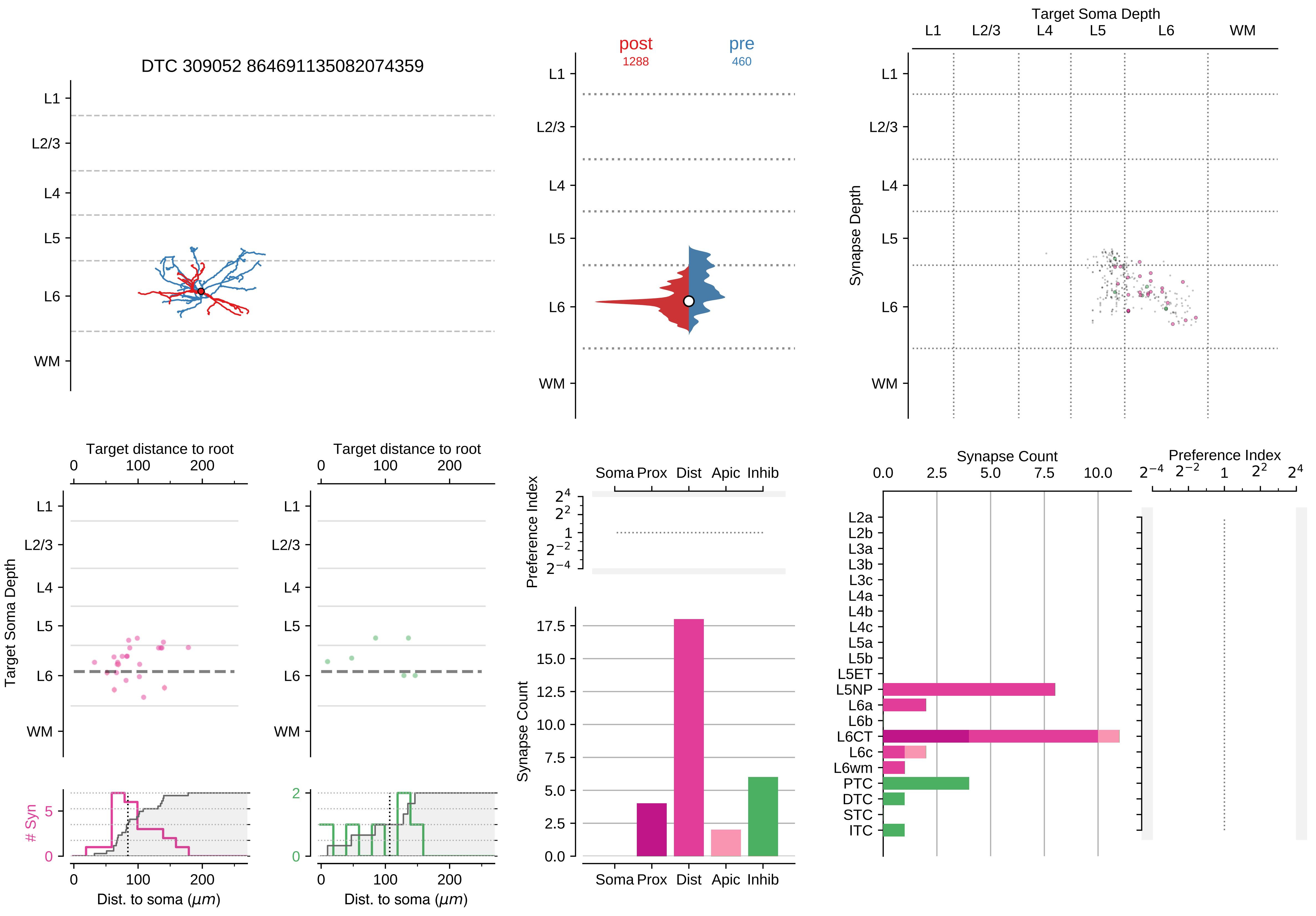

DTC 309052 864691135082074359
L1
L2/3
L4
L5
L6
WM
post
1288
pre
460
Target Soma Depth
Synapse Depth
Target distance to root
Target Soma Depth
Syn
Dist. to soma (μm)
Preference Index
Soma Prox
Dist
Apic
Inhib
Synapse Count
L2a
L2b
L3a
L3b
L3c
L4a
L4b
L4c
L5a
L5b
L5ET
L5NP
L6a
L6b
L6CT
L6c
L6wm
PTC
DTC
STC
ITC

Group 16

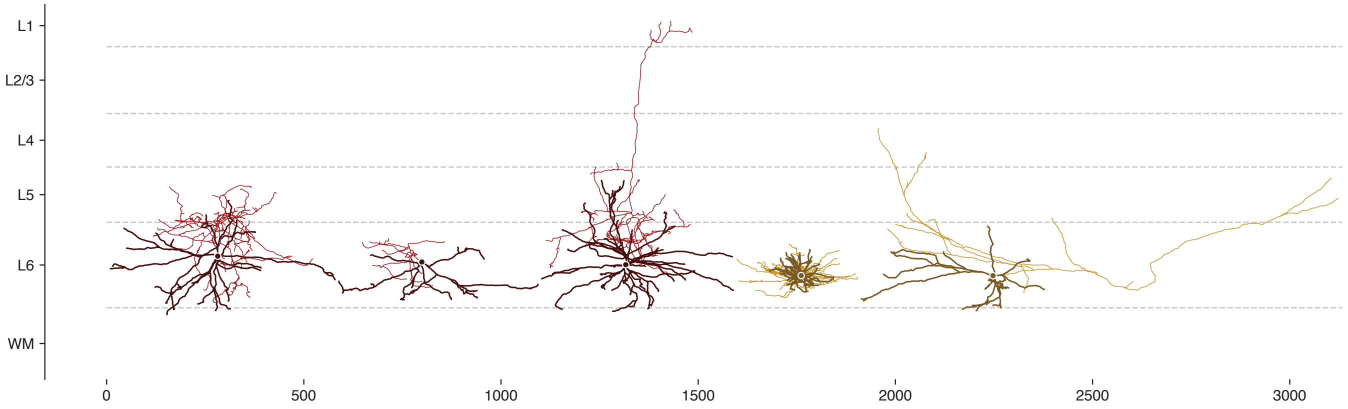

L1
L2/3
L4
L5
L6
WM
0
500
1000
1500
2000
2500
3000

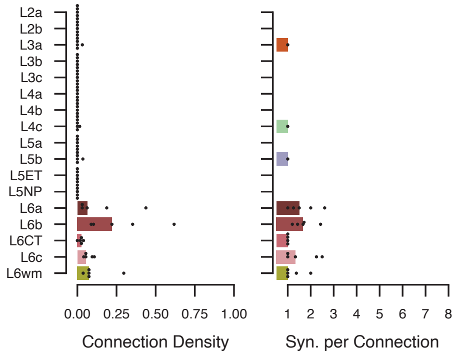

L2a
L2b
L3a
L3b
L3c
L4a
L4b
L4c
L5a
L5b
L5ET
L5NP
L6a
L6b
L6CT
L6c
L6wm
0.00 0.25 0.50 0.75 1.00
Connection Density
1 2 3 4 5 6 7 8
Syn. per Connection

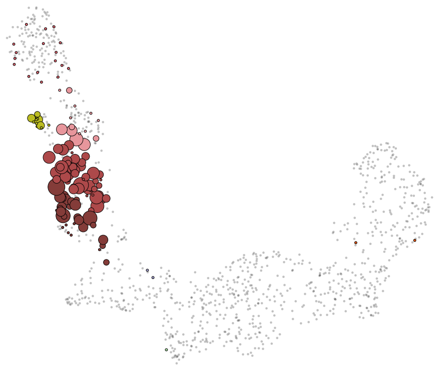

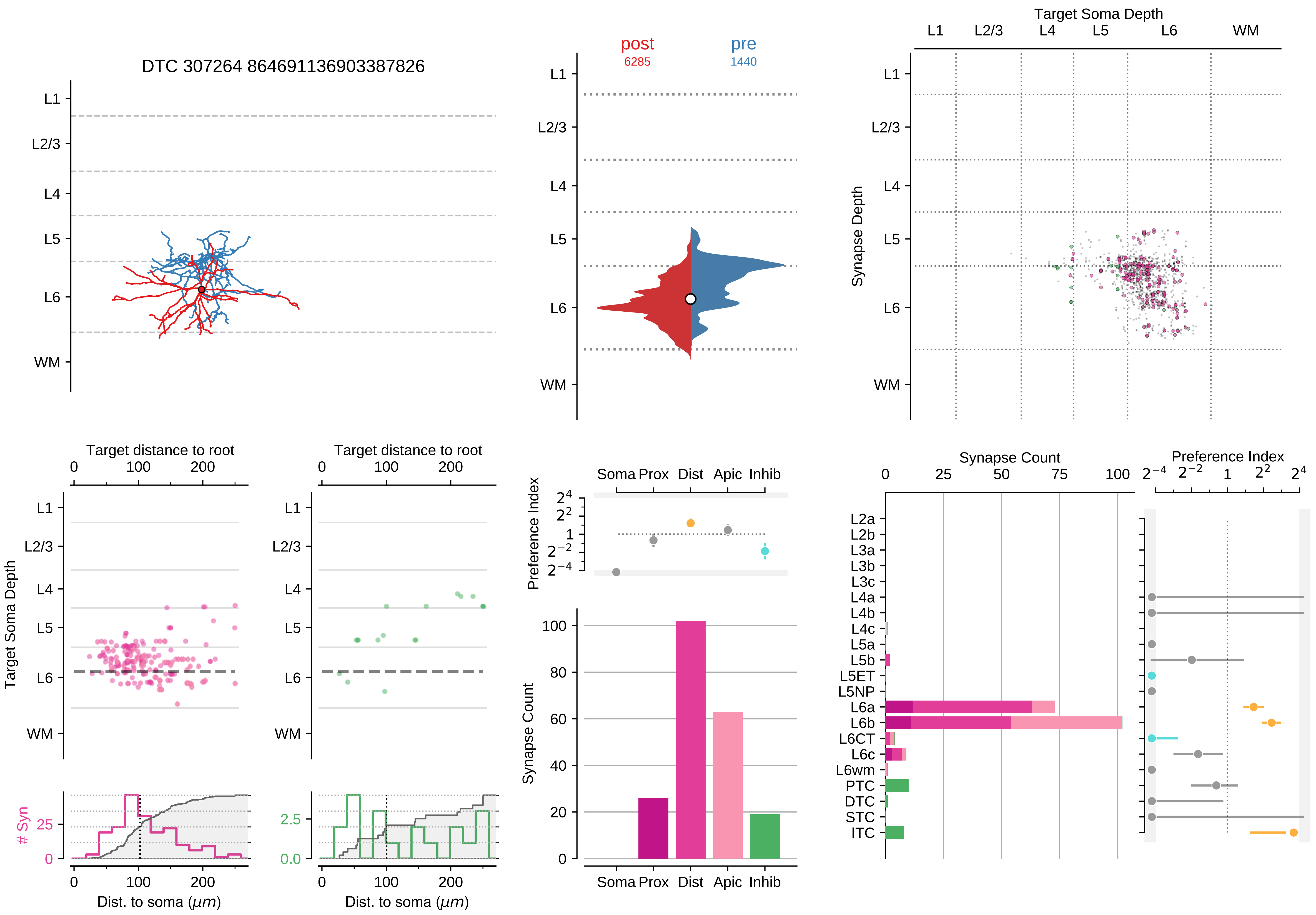

DTC 307264 864691136903387826
L1
L2/3
L4
L5
L6
WM
post
6285
pre
1440
Target Soma Depth
Synapse Depth
Target distance to root
Target Soma Depth
Syn
Dist. to soma (μm)
Preference Index
Soma Prox Dist Apic Inhib
Synapse Count
L2a
L2b
L3a
L3b
L3c
L4a
L4b
L4c
L5a
L5b
L5ET
L5NP
L6a
L6b
L6CT
L6c
L6wm
PTC
DTC
STC
ITC

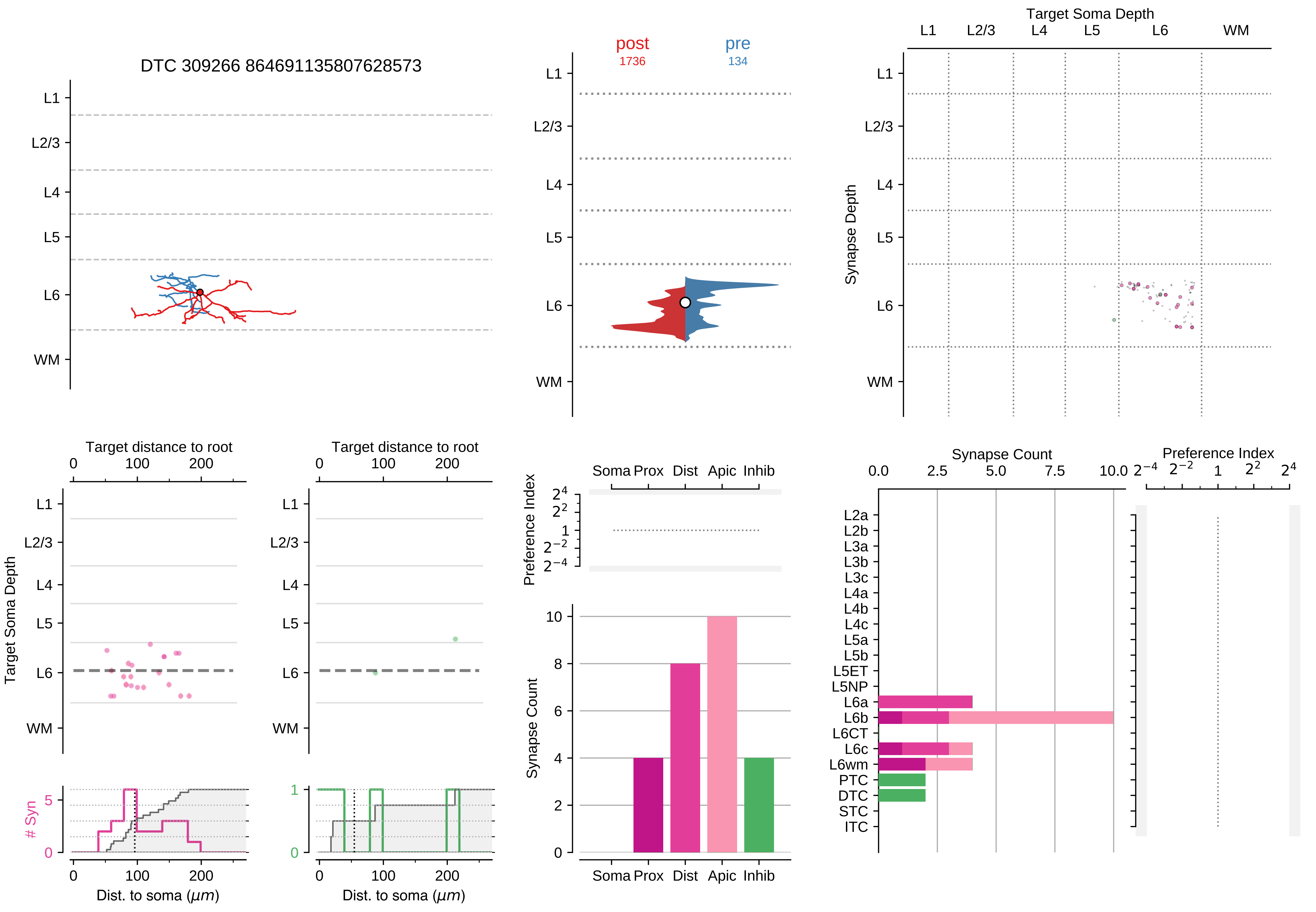

DTC 309266 864691135807628573
L1
L2/3
L4
L5
L6
WM
post
1736
pre
134
Target Soma Depth
Synapse Depth
Target distance to root
Target Soma Depth
Syn
Dist. to soma (μm)
Preference Index
Synapse Count
Soma
Prox
Dist
Apic
Inhib
Synapse Count
Preference Index
L2a
L2b
L3a
L3b
L3c
L4a
L4b
L4c
L5a
L5b
L5ET
L5NP
L6a
L6b
L6CT
L6c
L6wm
PTC
DTC
STC
ITC

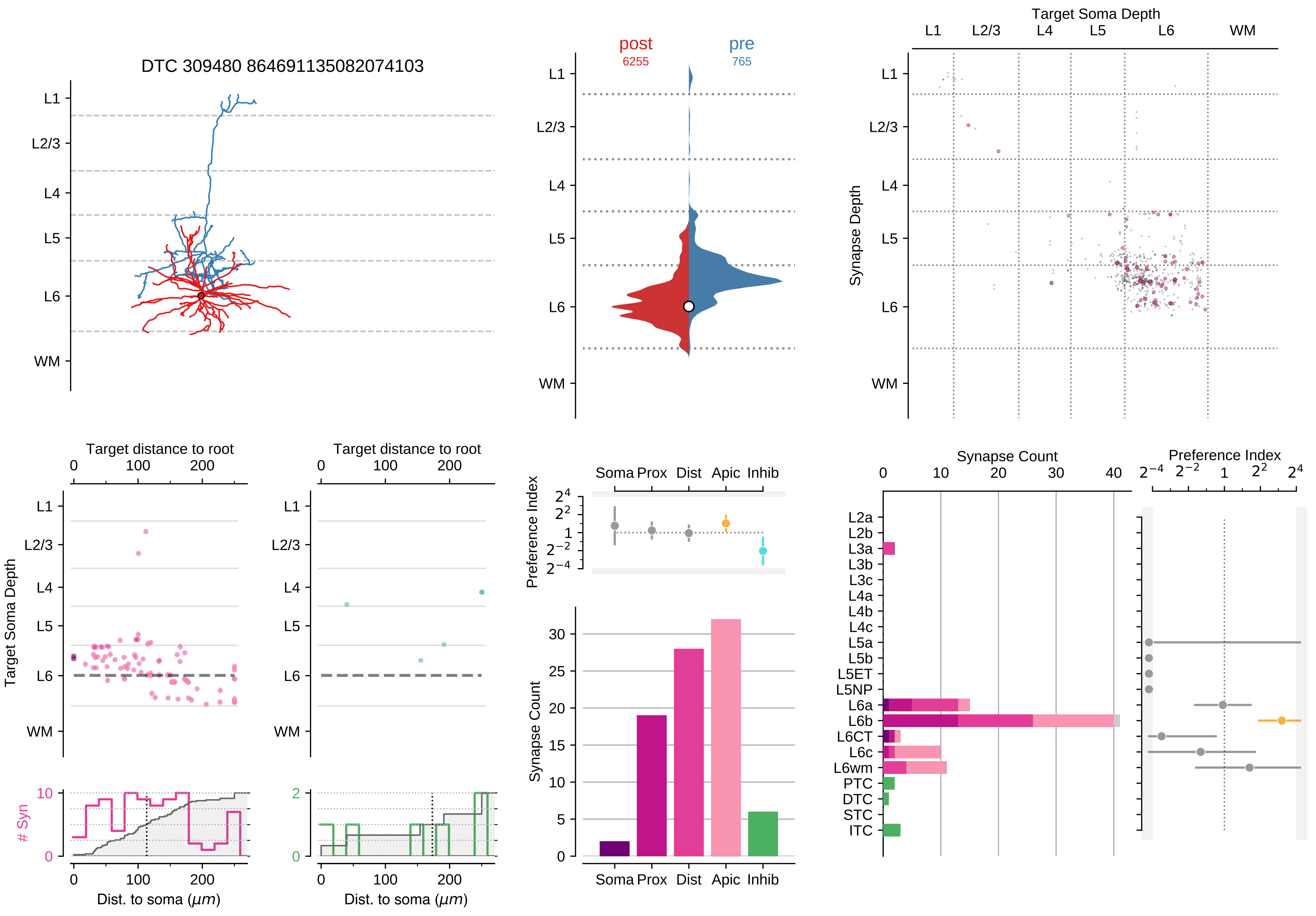

DTC 309480 864691135082074103
L1
L2/3
L4
L5
L6
WM
post
6255
pre
765
Target Soma Depth
Synapse Depth
Target distance to root
Target Soma Depth
Syn
Dist. to soma (μm)
Soma Prox Dist Apic Inhib
Preference Index
Synapse Count
L2a
L2b
L3a
L3b
L3c
L4a
L4b
L4c
L5a
L5b
L5ET
L5NP
L6a
L6b
L6CT
L6c
L6wm
PTC
DTC
STC
ITC

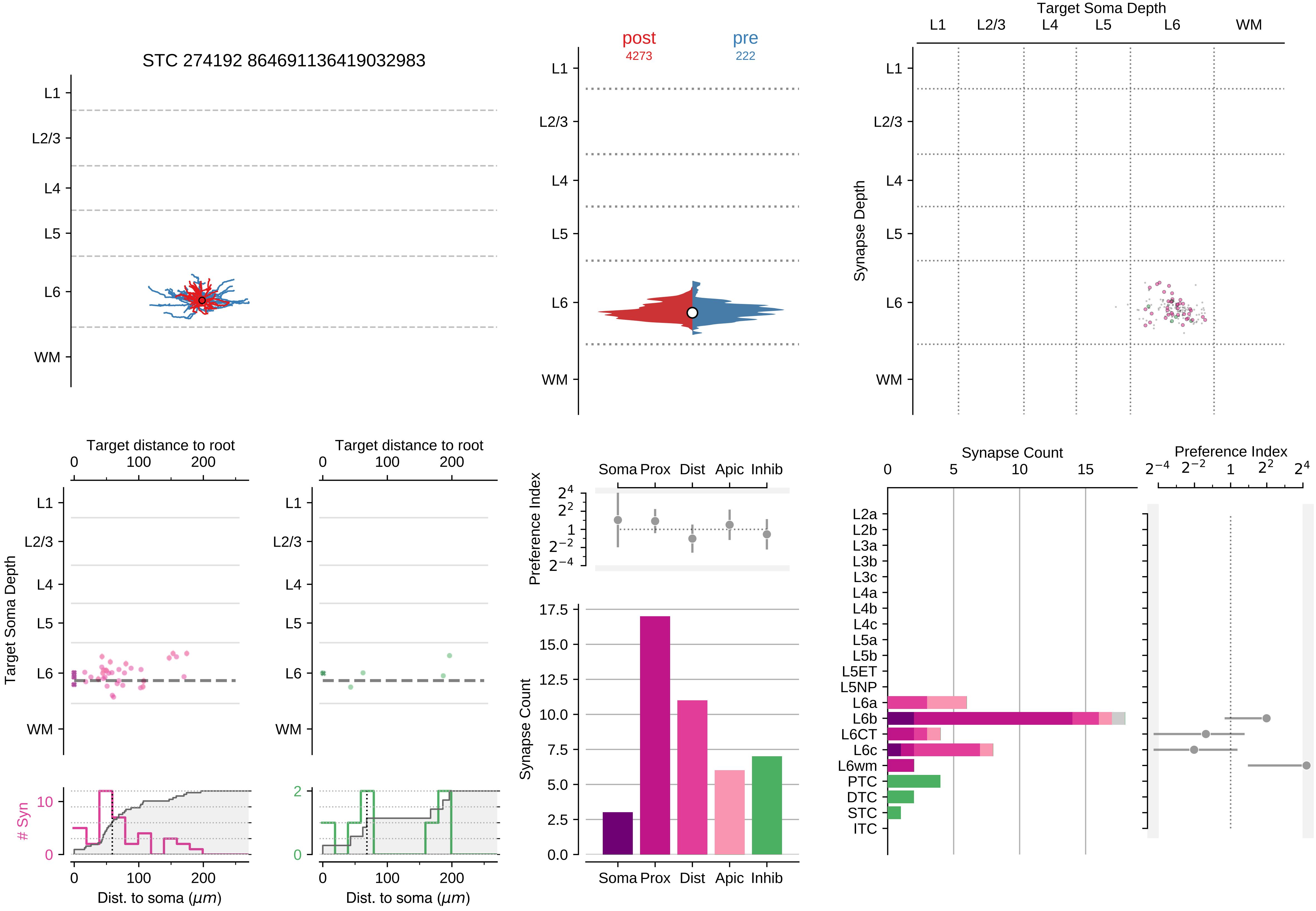

STC 274192 864691136419032983
L1
L2/3
L4
L5
L6
WM
post
4273
pre
222
Target Soma Depth
Synapse Depth
Target distance to root
Target Soma Depth
Syn
Dist. to soma (μm)
Preference Index
Soma Prox Dist Apic Inhib
Synapse Count
L2a
L2b
L3a
L3b
L3c
L4a
L4b
L4c
L5a
L5b
L5ET
L5NP
L6a
L6b
L6CT
L6c
L6wm
PTC
DTC
STC
ITC

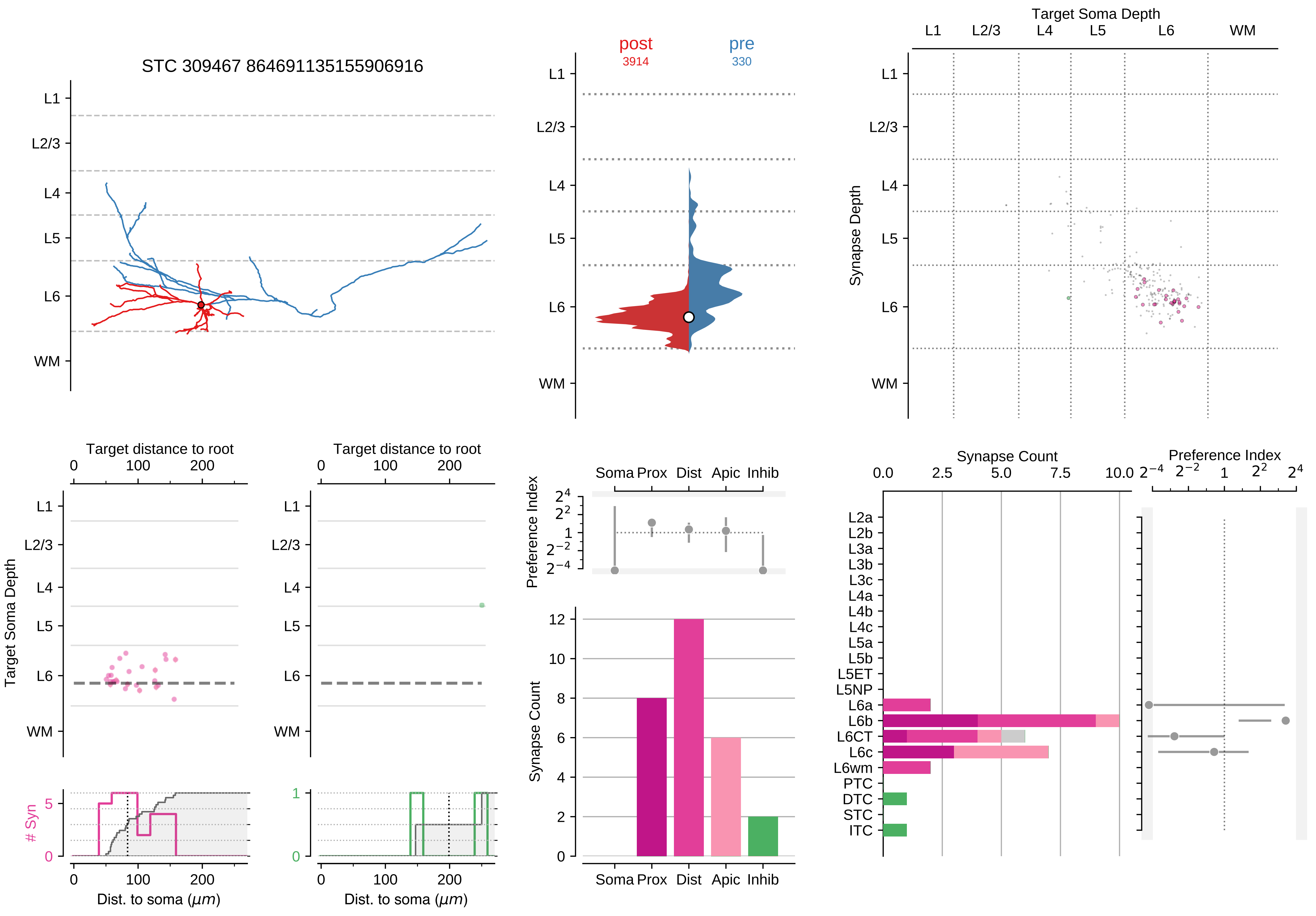

STC 309467 864691135155906916
L1
L2/3
L4
L5
L6
WM
post
3914
pre
330
Target Soma Depth
Synapse Depth
Target distance to root
Dist. to soma (μm)
Syn
Preference Index
Soma Prox Dist Apic Inhib
Synapse Count
L2a
L2b
L3a
L3b
L3c
L4a
L4b
L4c
L5a
L5b
L5ET
L5NP
L6a
L6b
L6CT
L6c
L6wm
PTC
DTC
STC
ITC

Group 17

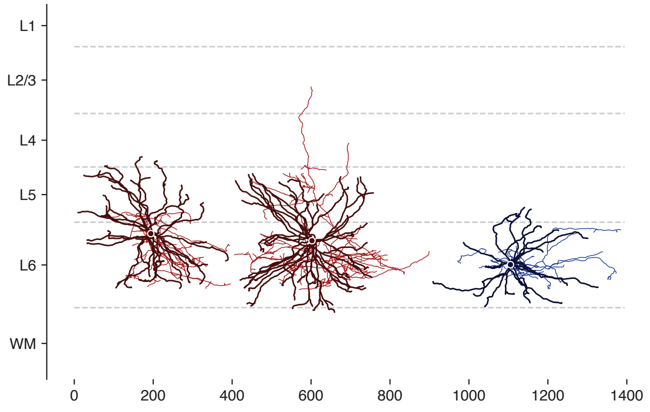

L1
L2/3
L4
L5
L6
WM
0
200
400
600
800
1000
1200
1400

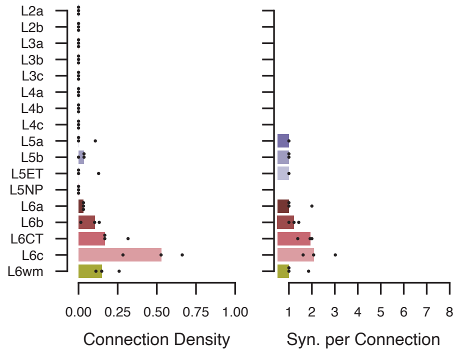

L2a
L2b
L3a
L3b
L3c
L4a
L4b
L4c
L5a
L5b
L5ET
L5NP
L6a
L6b
L6CT
L6c
L6wm
0.00
0.25
0.50
0.75
1.00
Connection Density
1
2
3
4
5
6
7
8
Syn. per Connection

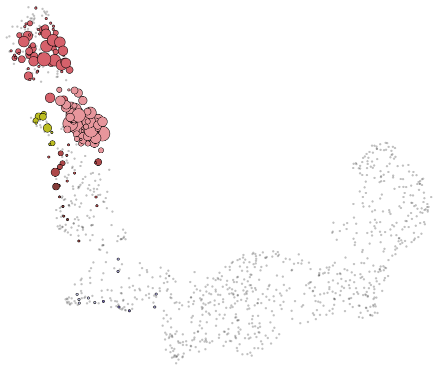

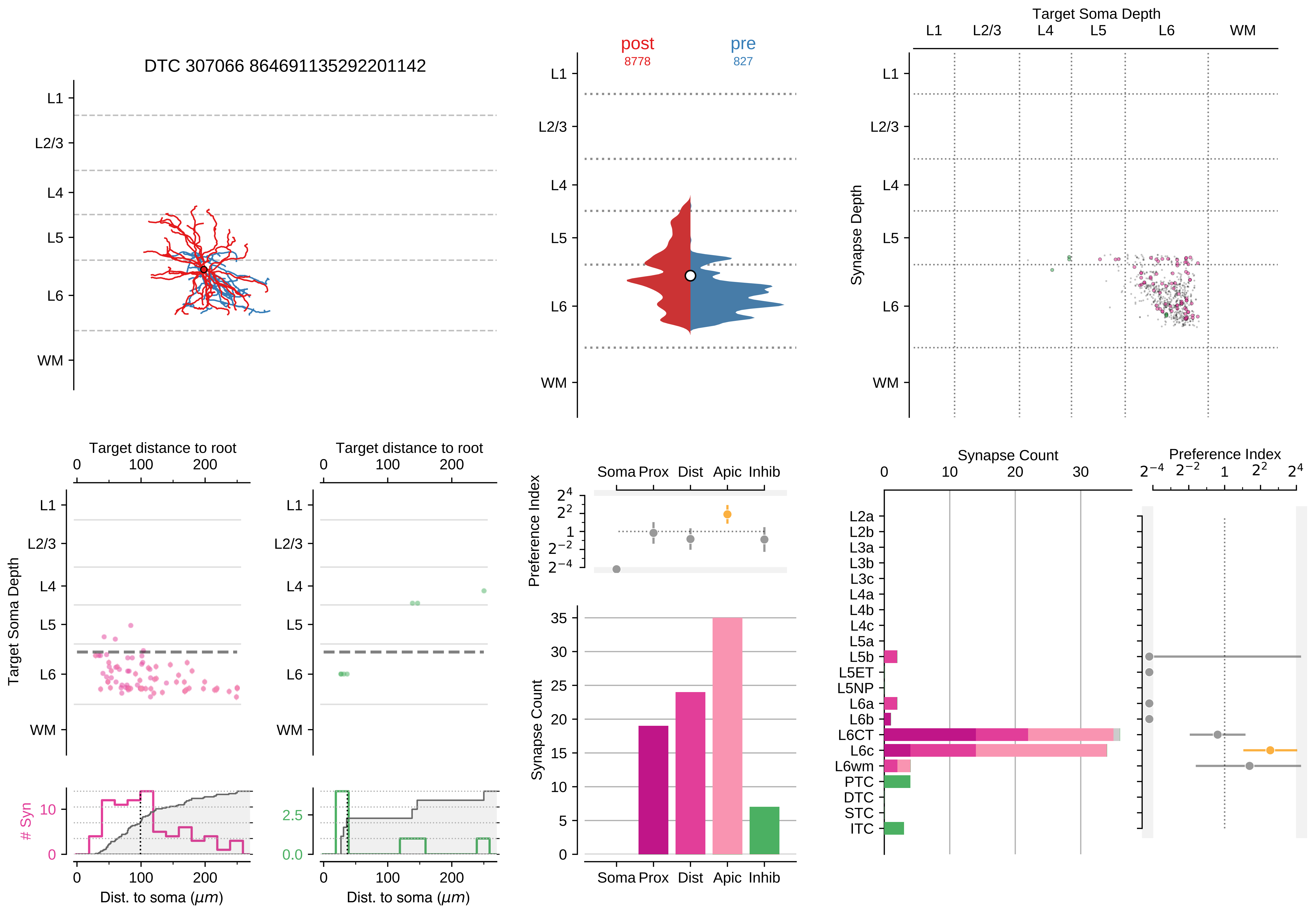

DTC 307066 864691135292201142
L1
L2/3
L4
L5
L6
WM
post
8778
pre
827
Target Soma Depth
Synapse Depth
Target distance to root
0
100
200
Target Soma Depth
Syn
Dist. to soma (μm)
Preference Index
Soma
Prox
Dist
Apic
Inhib
Synapse Count
L2a
L2b
L3a
L3b
L3c
L4a
L4b
L4c
L5a
L5b
L5ET
L5NP
L6a
L6b
L6CT
L6c
L6wm
PTC
DTC
STC
ITC

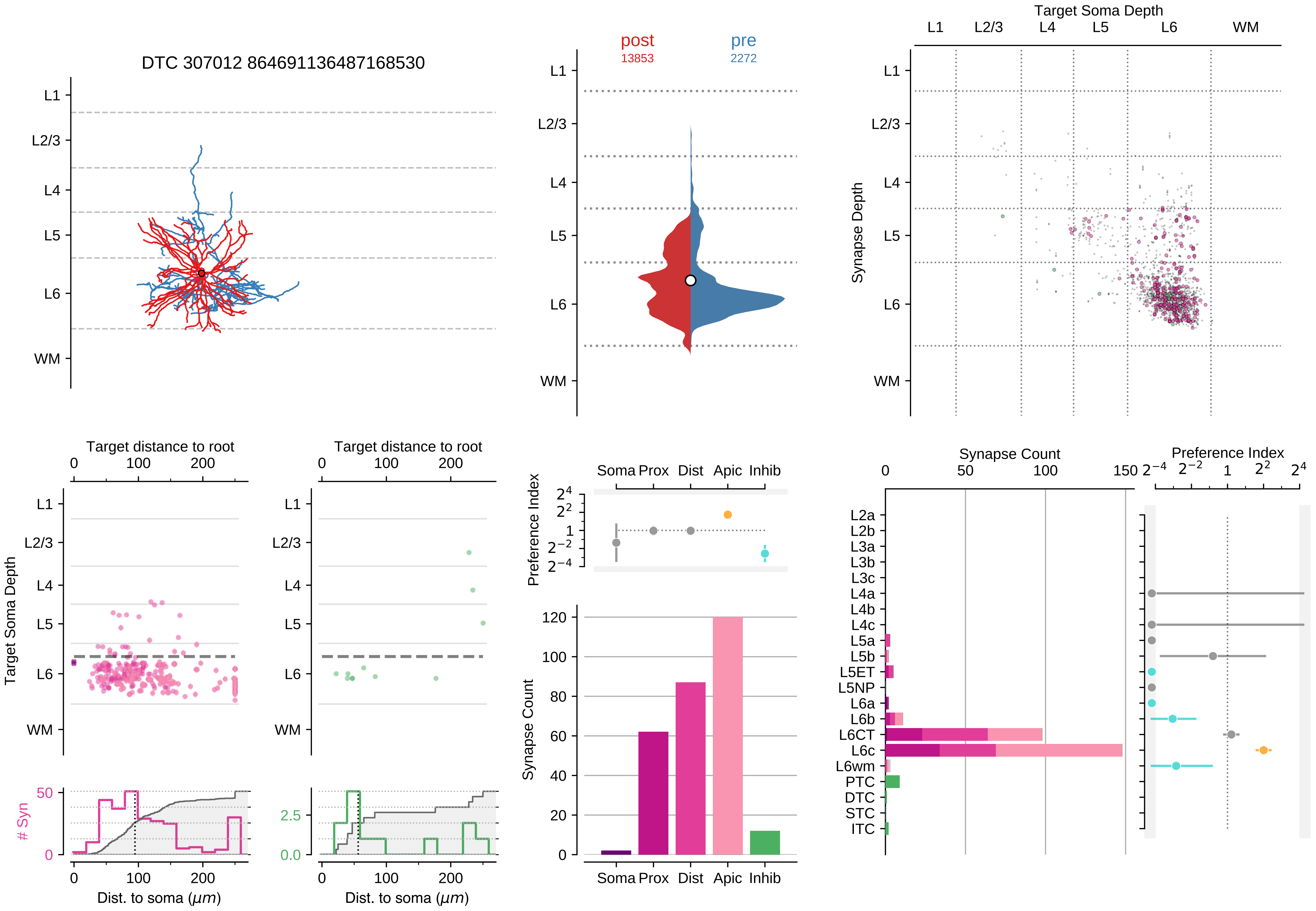

DTC 307012 864691136487168530
L1
L2/3
L4
L5
L6
WM
post
13853
pre
2272
Target Soma Depth
Synapse Depth
Target distance to root
Target Soma Depth
Syn
Dist. to soma (μm)
Preference Index
Soma
Prox
Dist
Apic
Inhib
Synapse Count
L2a
L2b
L3a
L3b
L3c
L4a
L4b
L4c
L5a
L5b
L5ET
L5NP
L6a
L6b
L6CT
L6c
L6wm
PTC
DTC
STC
ITC

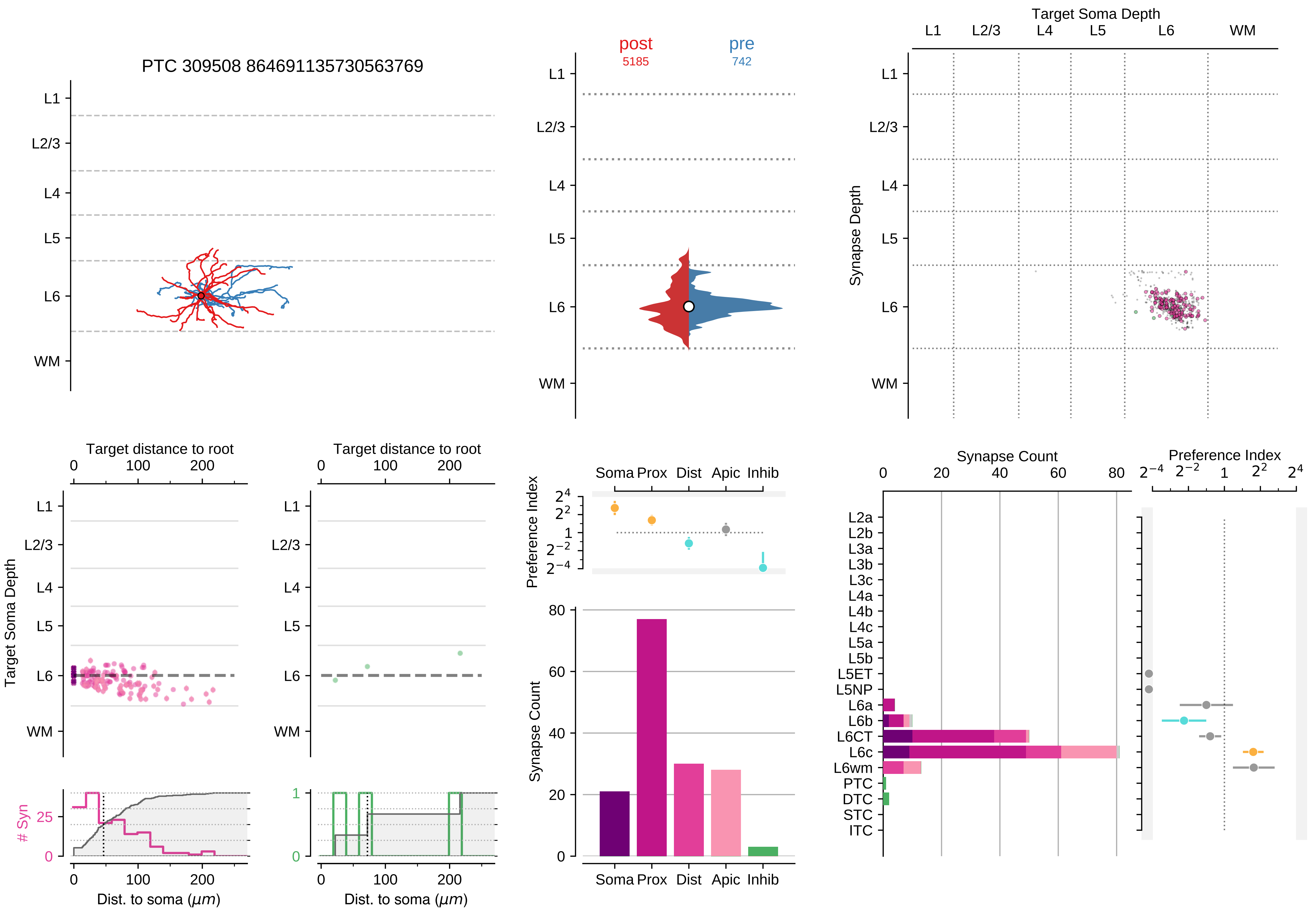

PTC 309508 864691135730563769
L1
L2/3
L4
L5
L6
WM
post
5185
pre
742
Target Soma Depth
Synapse Depth
Target distance to root
Target Soma Depth
Syn
Dist. to soma (μm)
Preference Index
Soma Prox Dist Apic Inhib
Synapse Count
L2a
L2b
L3a
L3b
L3c
L4a
L4b
L4c
L5a
L5b
L5ET
L5NP
L6a
L6b
L6CT
L6c
L6wm
PTC
DTC
STC
ITC

Group 18

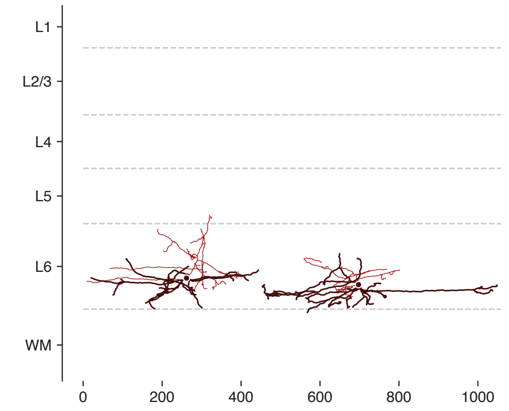
L1
L2/3
L4
L5
L6
WM
0
200
400
600
800
1000

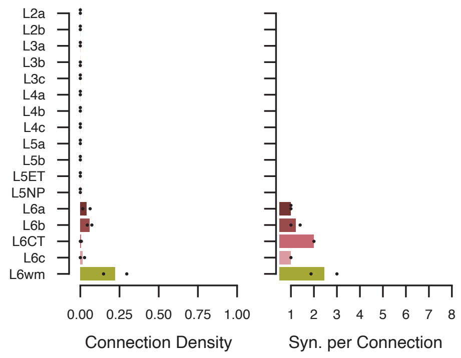
L2a
L2b
L3a
L3b
L3c
L4a
L4b
L4c
L5a
L5b
L5ET
L5NP
L6a
L6b
L6CT
L6c
L6wm
0.00
0.25
0.50
0.75
1.00
Connection Density
1
2
3
4
5
6
7
8
Syn. per Connection

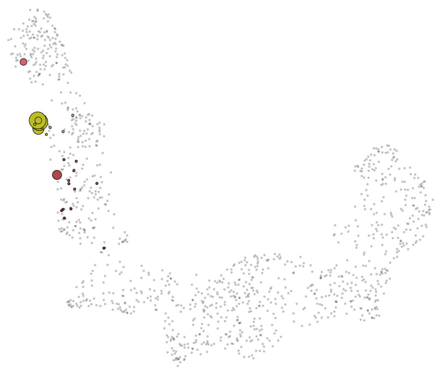

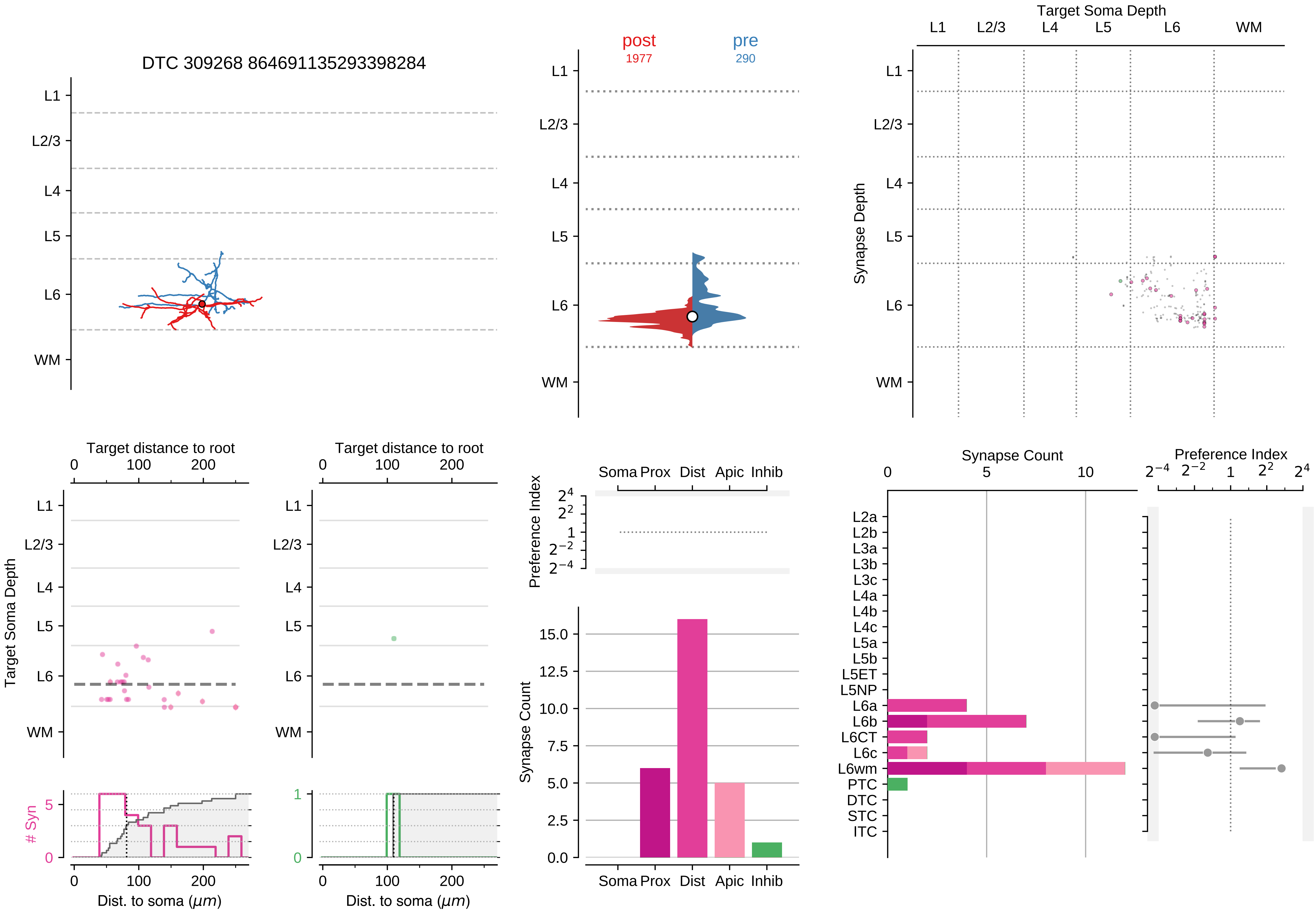

DTC 309268 864691135293398284
L1
L2/3
L4
L5
L6
WM
post
1977
pre
290
Target Soma Depth
Synapse Depth
Target distance to root
Target Soma Depth
Syn
Dist. to soma (μm)
Preference Index
Soma Prox Dist Apic Inhib
Synapse Count
L2a
L2b
L3a
L3b
L3c
L4a
L4b
L4c
L5a
L5b
L5ET
L5NP
L6a
L6b
L6CT
L6c
L6wm
PTC
DTC
STC
ITC

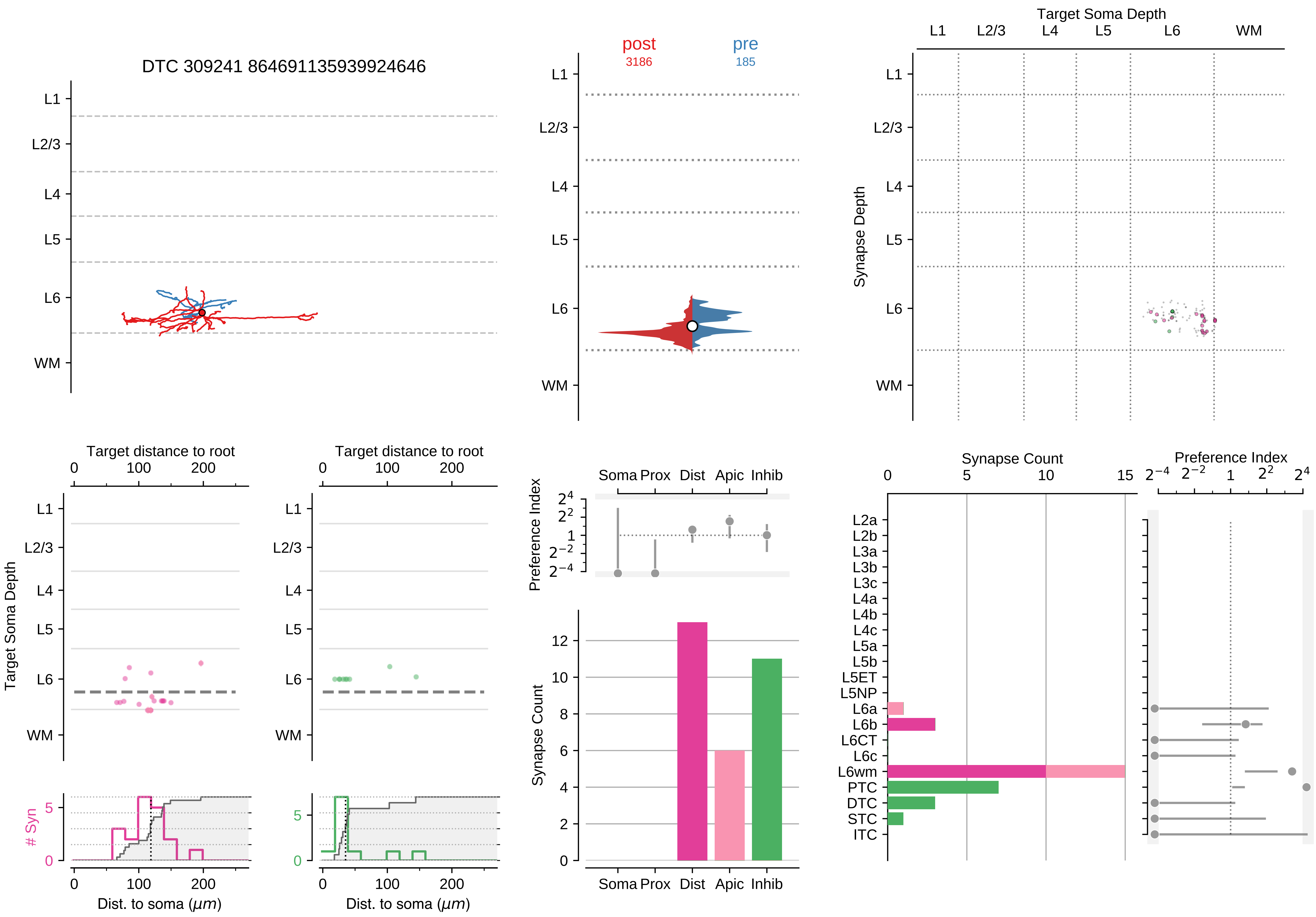

DTC 309241 864691135939924646
L1
L2/3
L4
L5
L6
WM
post
3186
pre
185
Target Soma Depth
Synapse Depth
Target distance to root
Target Soma Depth
Syn
Dist. to soma (μm)
Preference Index
Soma Prox Dist Apic Inhib
Synapse Count
L2a
L2b
L3a
L3b
L3c
L4a
L4b
L4c
L5a
L5b
L5ET
L5NP
L6a
L6b
L6CT
L6c
L6wm
PTC
DTC
STC
ITC

Group 19 (InhTC^{dist})

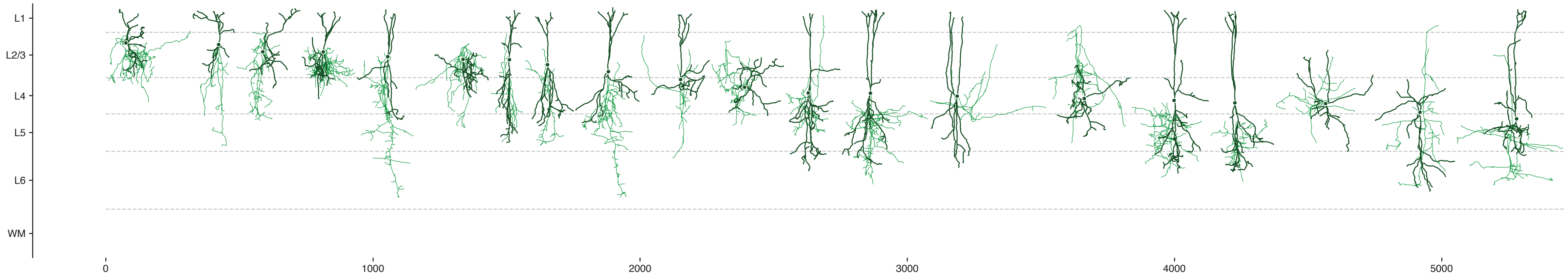

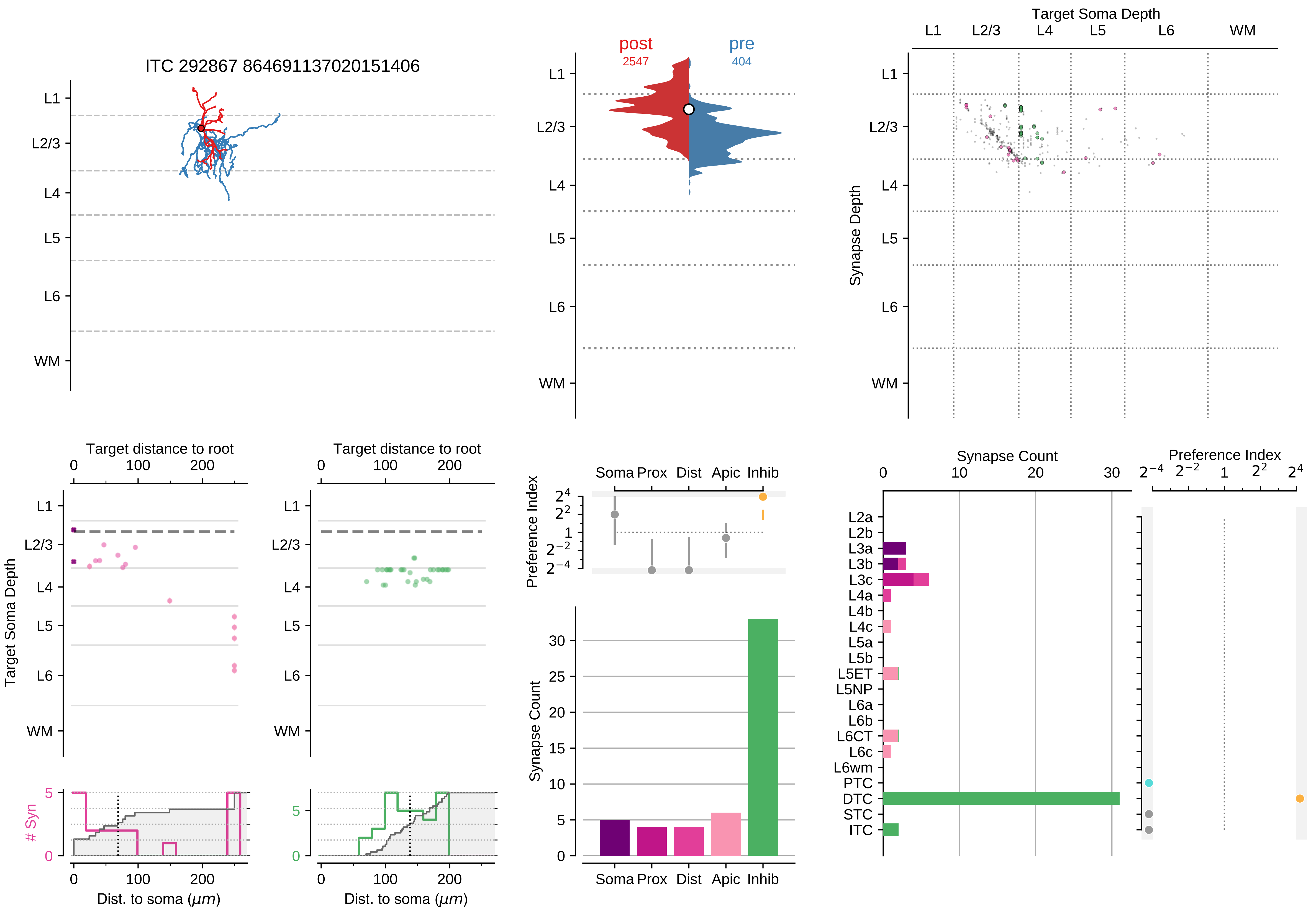

ITC 292867 864691137020151406
post
2547
pre
404
Target Soma Depth
Synapse Depth
L1
L2/3
L4
L5
L6
WM
Target distance to root
Target Soma Depth
Syn
Dist. to soma (μm)
Preference Index
Soma Prox Dist Apic Inhib
Synapse Count
Preference Index
L2a
L2b
L3a
L3b
L3c
L4a
L4b
L4c
L5a
L5b
L5ET
L5NP
L6a
L6b
L6CT
L6c
L6wm
PTC
DTC
STC
ITC

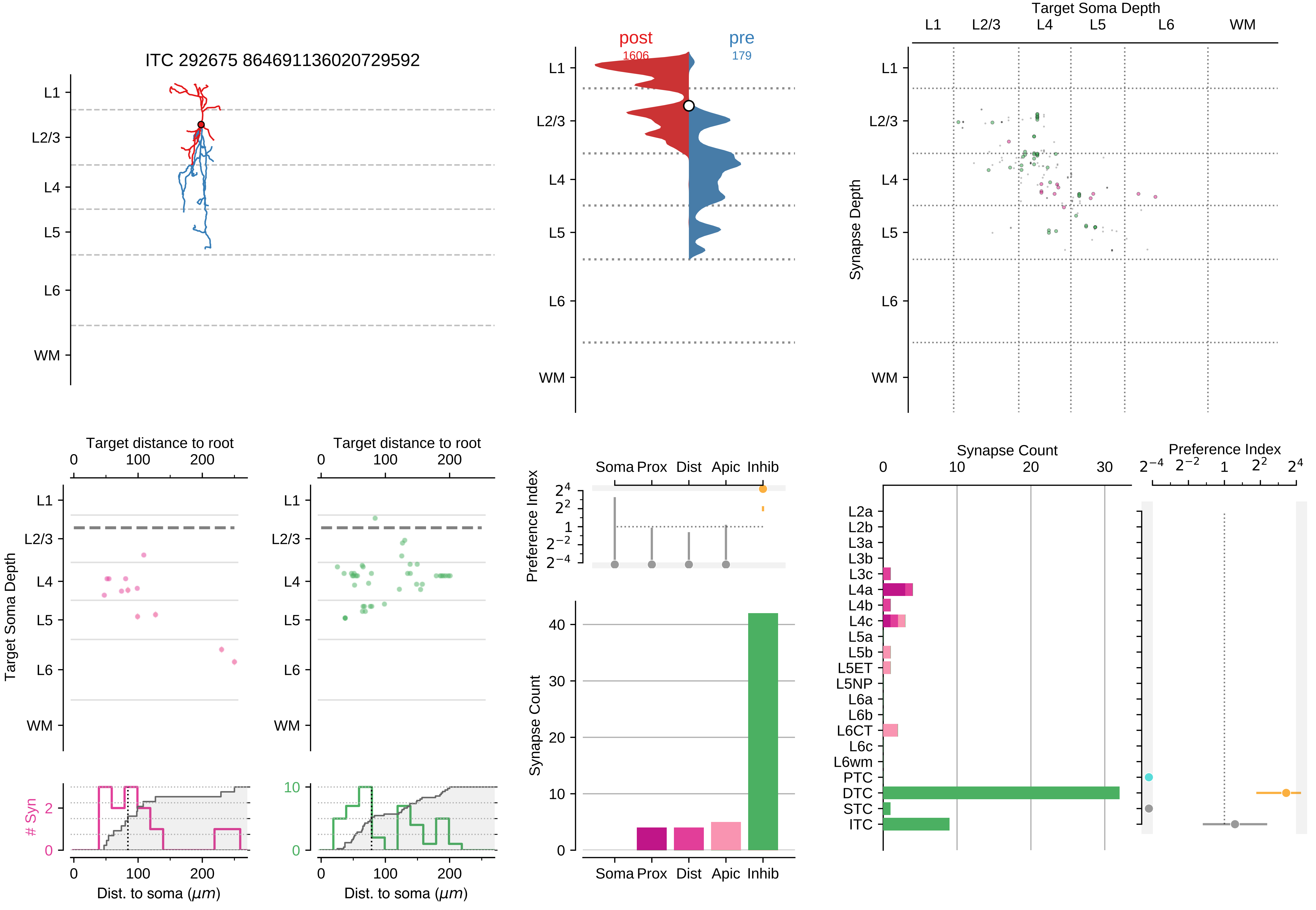

ITC 292675 864691136020729592
post
1606
pre
179
Target Soma Depth
Synapse Depth
L1
L2/3
L4
L5
L6
WM
Target distance to root
Target Soma Depth
Syn
Dist. to soma (μm)
Preference Index
Soma Prox Dist Apic Inhib
Synapse Count
L2a
L2b
L3a
L3b
L3c
L4a
L4b
L4c
L5a
L5b
L5ET
L5NP
L6a
L6b
L6CT
L6c
L6wm
PTC
DTC
STC
ITC

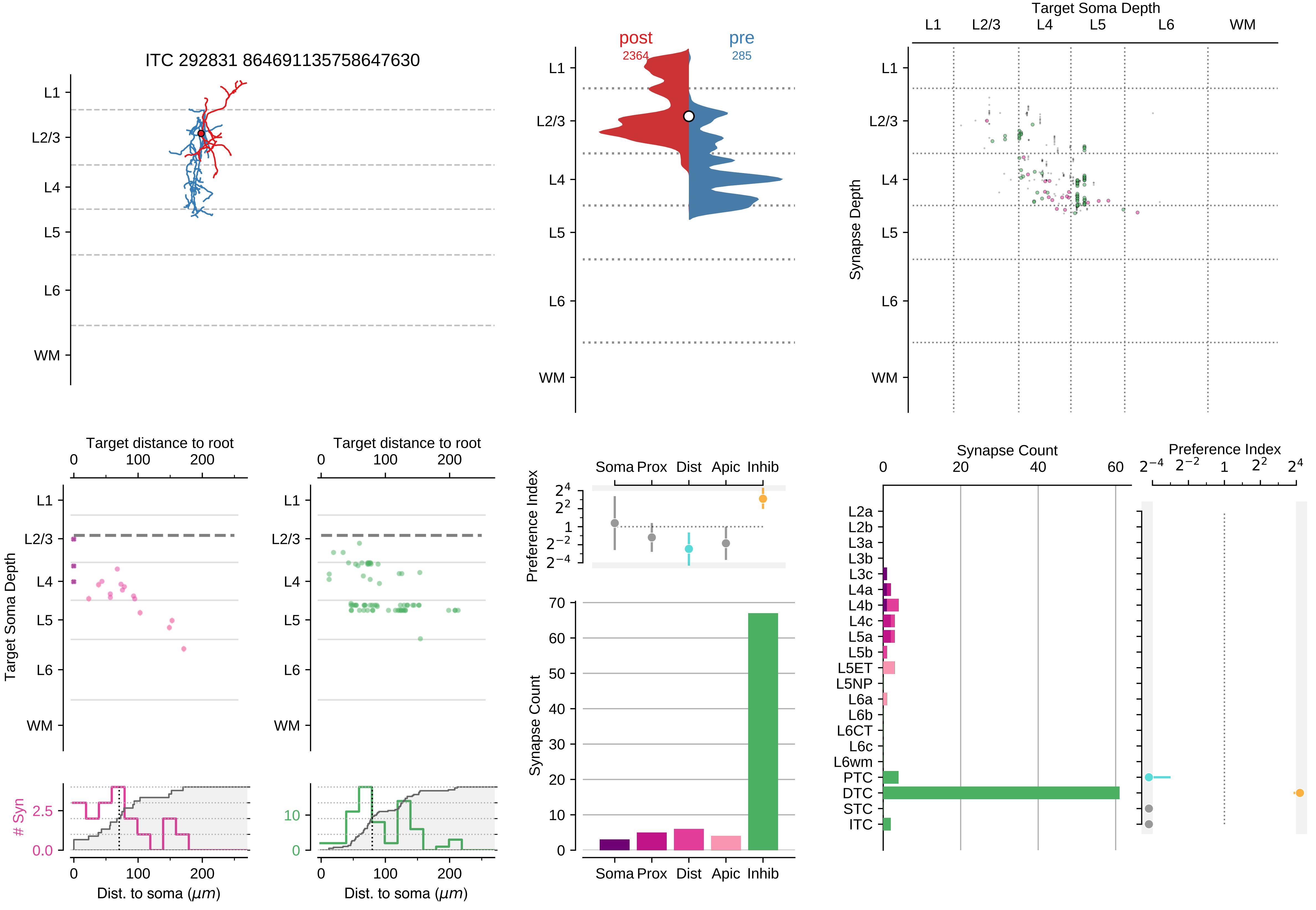

ITC 292831 864691135758647630
L1
L2/3
L4
L5
L6
WM
post
2364
pre
285
Target Soma Depth
Synapse Depth
Target distance to root
Target Soma Depth
Syn
Dist. to soma (μm)
Preference Index
Soma
Prox
Dist
Apic
Inhib
Synapse Count
L2a
L2b
L3a
L3b
L3c
L4a
L4b
L4c
L5a
L5b
L5ET
L5NP
L6a
L6b
L6CT
L6c
L6wm
PTC
DTC
STC
ITC

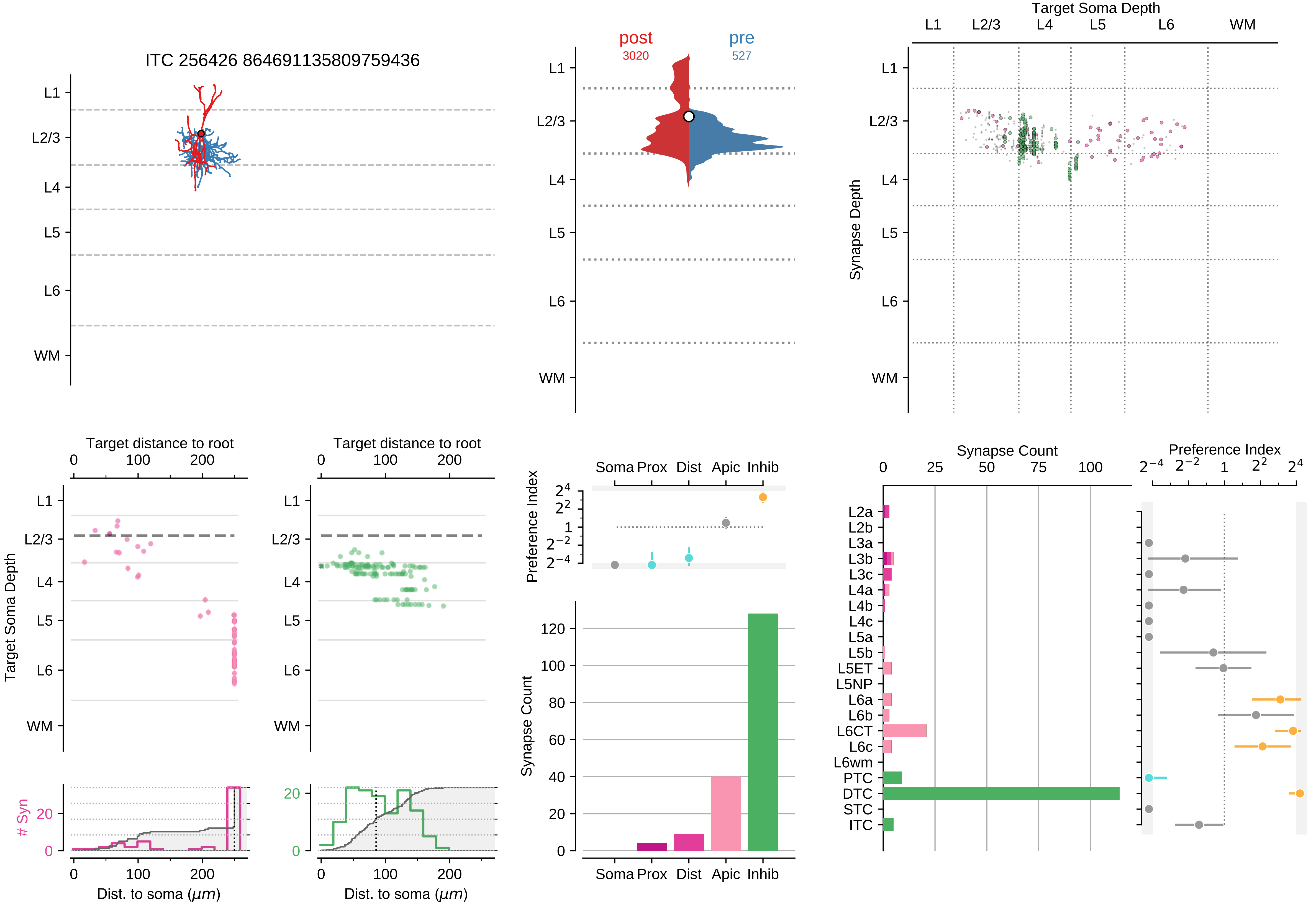
ITC 256426 864691135809759436
L1
L2/3
L4
L5
L6
WM
post
3020
pre
527
Target Soma Depth
Synapse Depth
Target distance to root
Target Soma Depth
Syn
Dist. to soma (μm)
Preference Index
Soma
Prox
Dist
Apic
Inhib
Synapse Count
L2a
L2b
L3a
L3b
L3c
L4a
L4b
L4c
L5a
L5b
L5ET
L5NP
L6a
L6b
L6CT
L6c
L6wm
PTC
DTC
STC
ITC

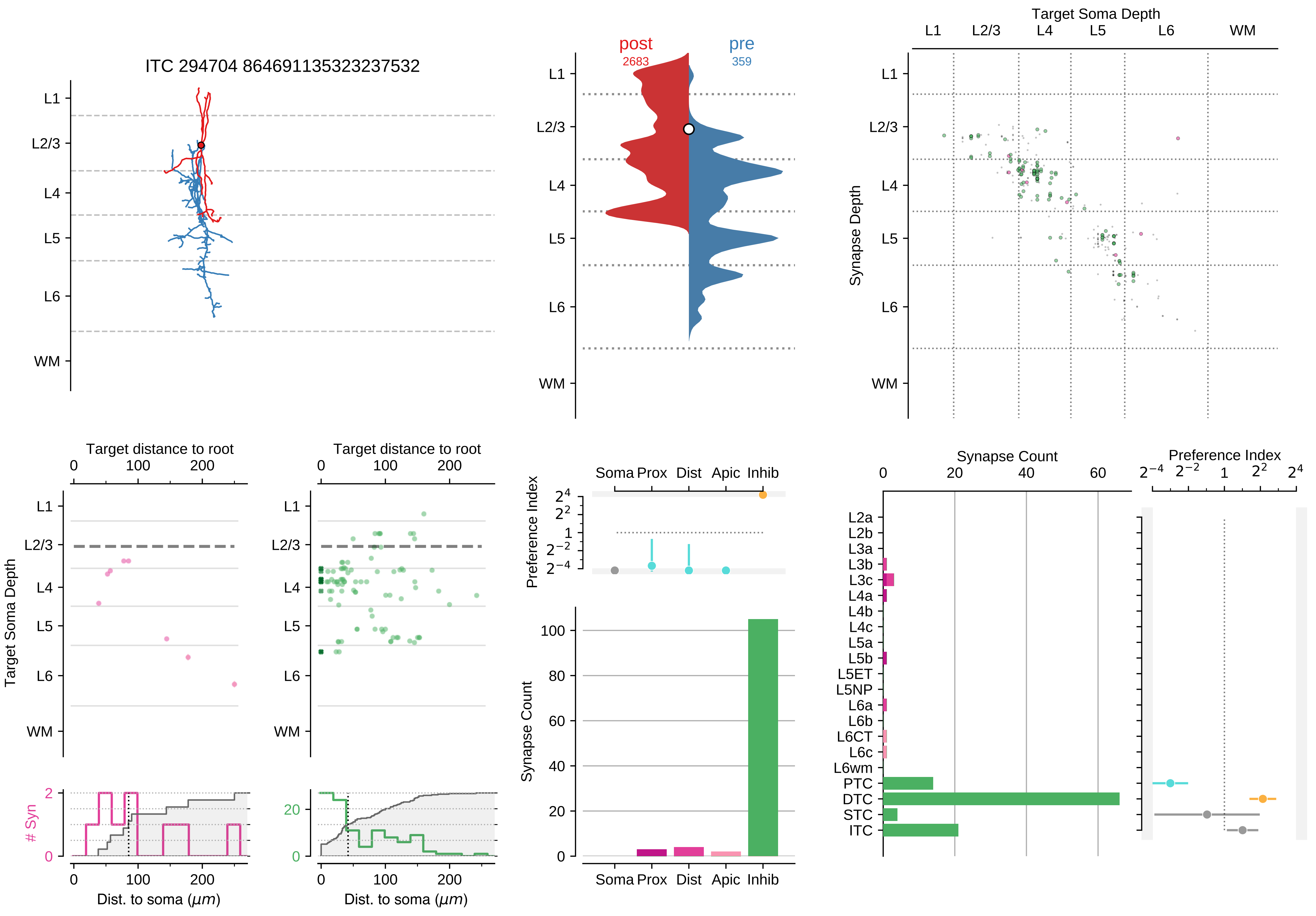
ITC 294704 864691135323237532
L1
L2/3
L4
L5
L6
WM
post
2683
pre
359
Target Soma Depth
Synapse Depth
Target distance to root
Target Soma Depth
Syn
Dist. to soma (μm)
Preference Index
Soma
Prox
Dist
Apic
Inhib
Synapse Count
Synapse Count
Preference Index
L2a
L2b
L3a
L3b
L3c
L4a
L4b
L4c
L5a
L5b
L5ET
L5NP
L6a
L6b
L6CT
L6c
L6wm
PTC
DTC
STC
ITC

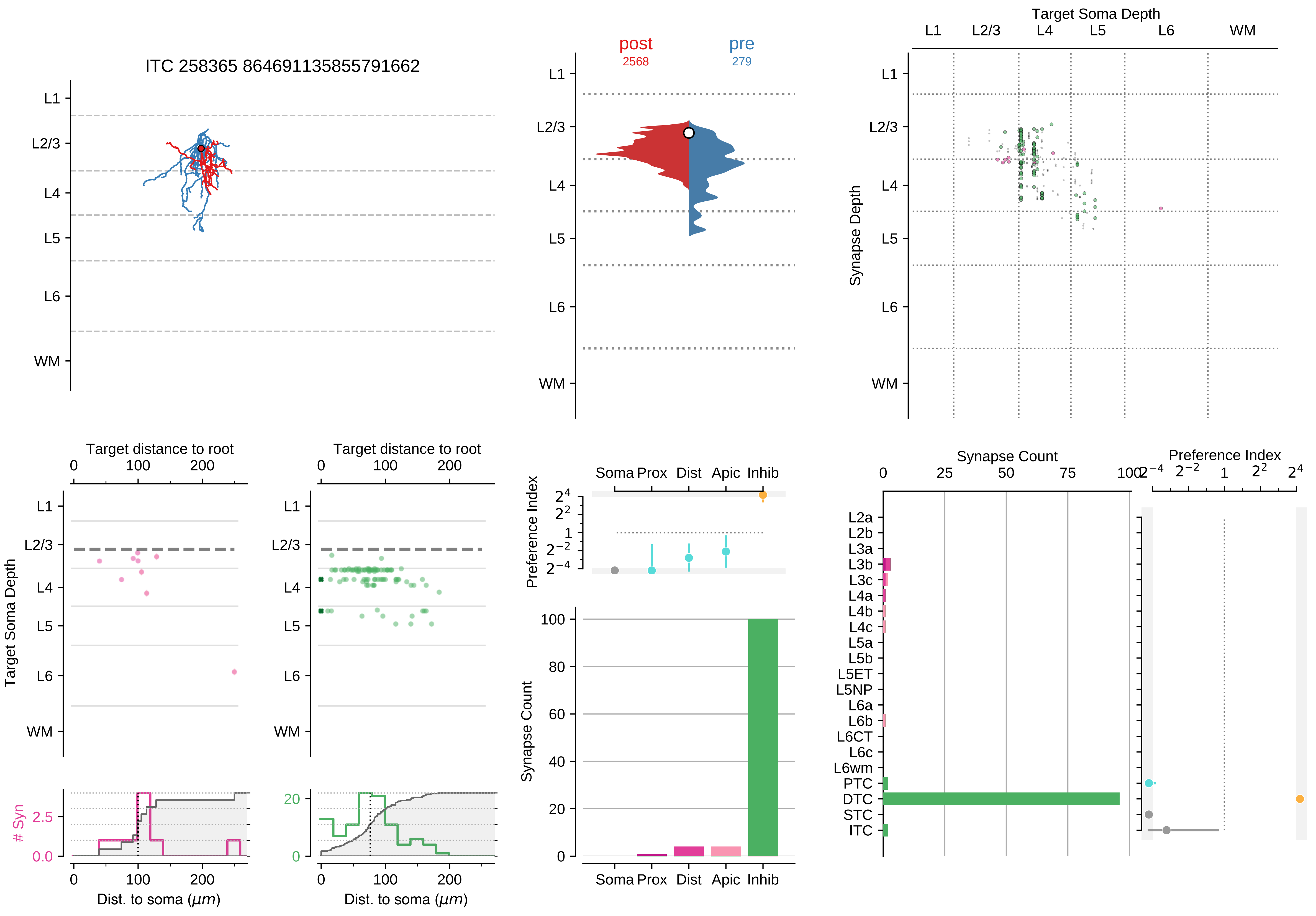
ITC 258365 864691135855791662
L1
L2/3
L4
L5
L6
WM
post
2568
pre
279
Target Soma Depth
Synapse Depth
Target distance to root
Target Soma Depth
Syn
Dist. to soma (μm)
Preference Index
Soma Prox Dist Apic Inhib
Synapse Count
L2a
L2b
L3a
L3b
L3c
L4a
L4b
L4c
L5a
L5b
L5ET
L5NP
L6a
L6b
L6CT
L6c
L6wm
PTC
DTC
STC
ITC

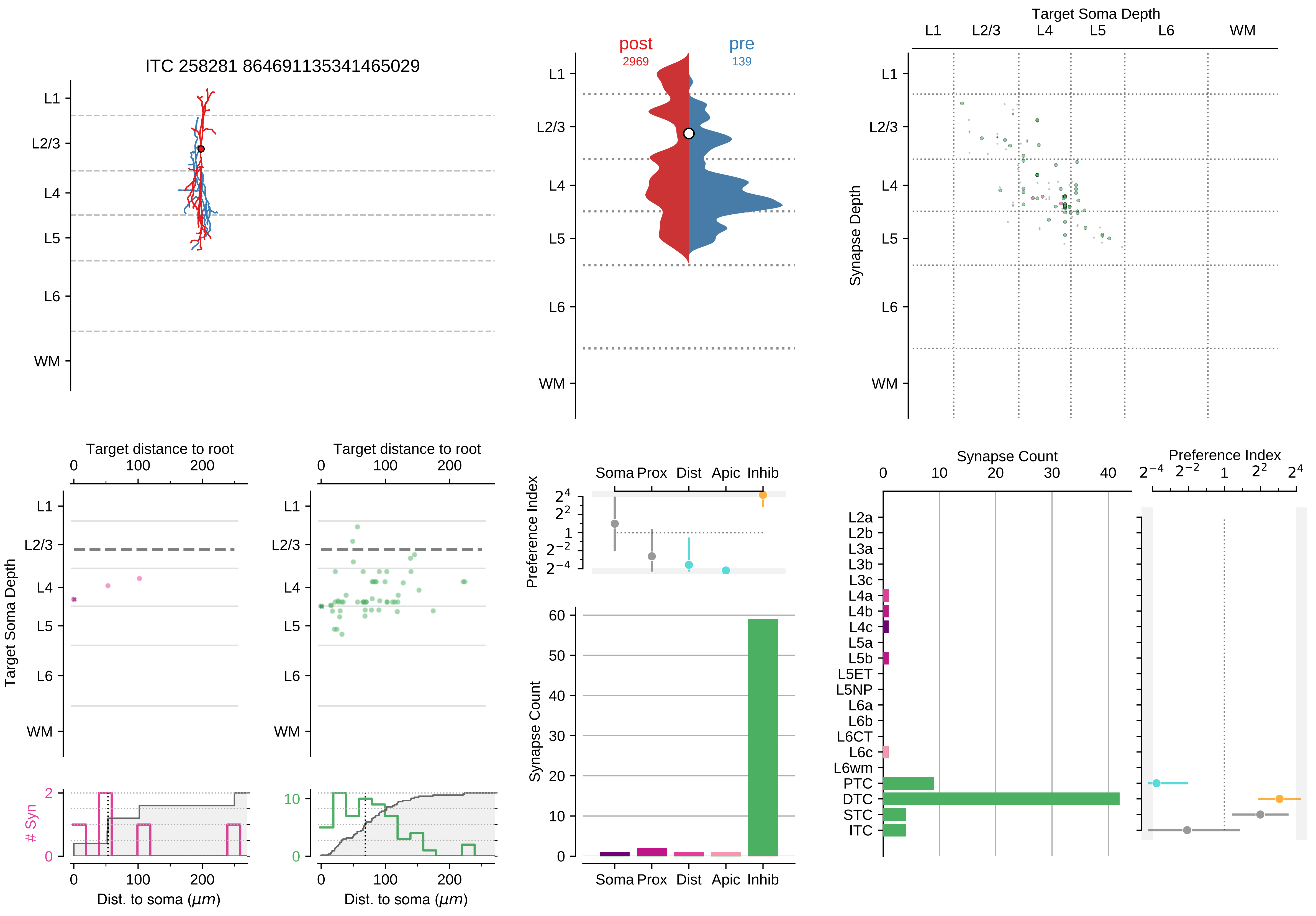

ITC 258281 864691135341465029
L1
L2/3
L4
L5
L6
WM
post
2969
pre
139
Target Soma Depth
Synapse Depth
Target distance to root
Target Soma Depth
Syn
Dist. to soma (μm)
Preference Index
Soma Prox Dist Apic Inhib
Synapse Count
Synapse Count
Preference Index
L2a
L2b
L3a
L3b
L3c
L4a
L4b
L4c
L5a
L5b
L5ET
L5NP
L6a
L6b
L6CT
L6c
L6wm
PTC
DTC
STC
ITC

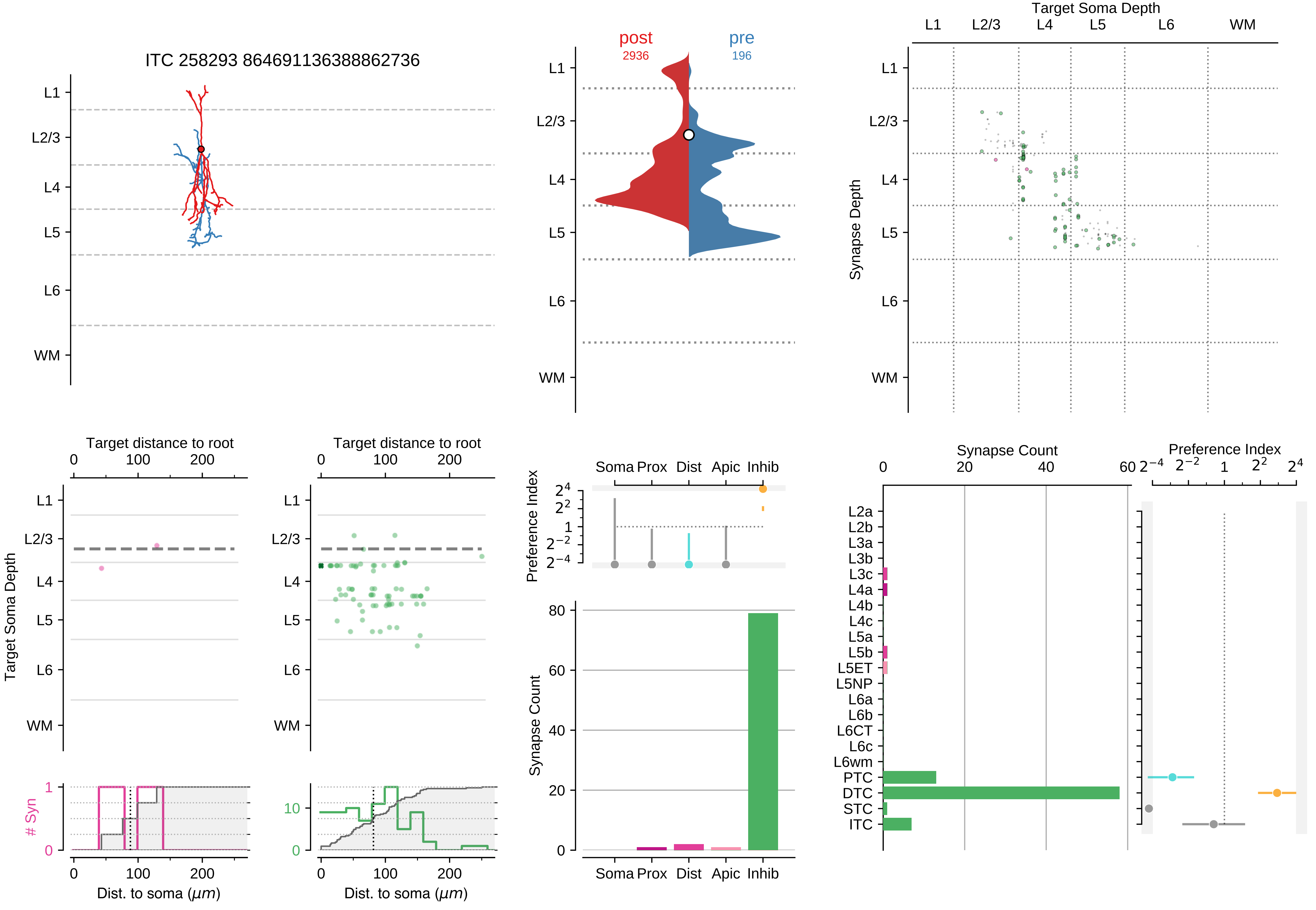

ITC 258293 864691136388862736
L1
L2/3
L4
L5
L6
WM
post
2936
pre
196
Target Soma Depth
Synapse Depth
Target distance to root
0
100
200
Target Soma Depth
Syn
Dist. to soma (μm)
Soma
Prox
Dist
Apic
Inhib
Preference Index
Synapse Count
Synapse Count
Preference Index
L2a
L2b
L3a
L3b
L3c
L4a
L4b
L4c
L5a
L5b
L5ET
L5NP
L6a
L6b
L6CT
L6c
L6wm
PTC
DTC
STC
ITC

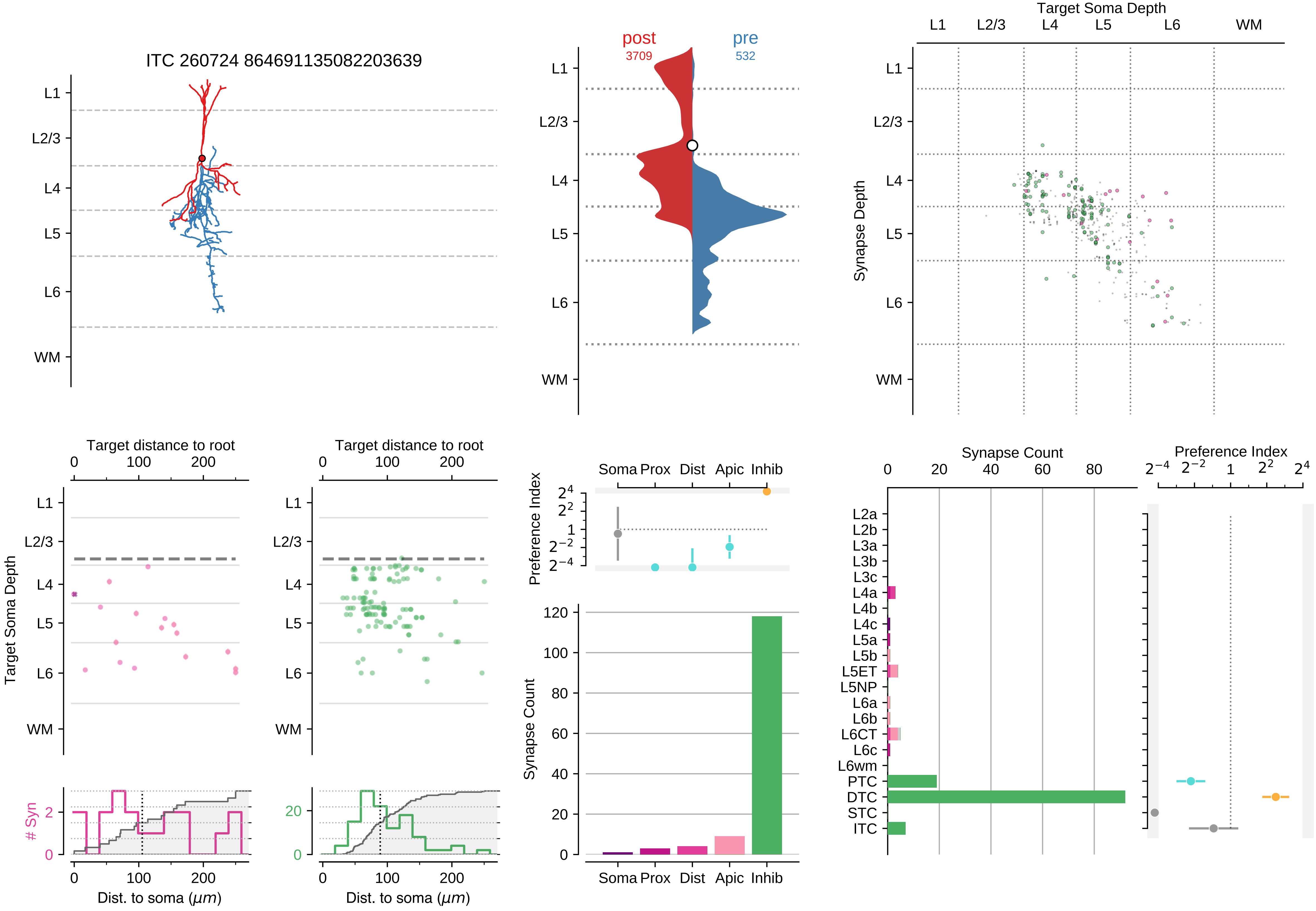

ITC 260724 864691135082203639
L1
L2/3
L4
L5
L6
WM
post
3709
pre
532
Target Soma Depth
Synapse Depth
Target distance to root
Target Soma Depth
Syn
Dist. to soma (μm)
Preference Index
Soma Prox Dist Apic Inhib
Synapse Count
Synapse Count
Preference Index
L2a
L2b
L3a
L3b
L3c
L4a
L4b
L4c
L5a
L5b
L5ET
L5NP
L6a
L6b
L6CT
L6c
L6wm
PTC
DTC
STC
ITC

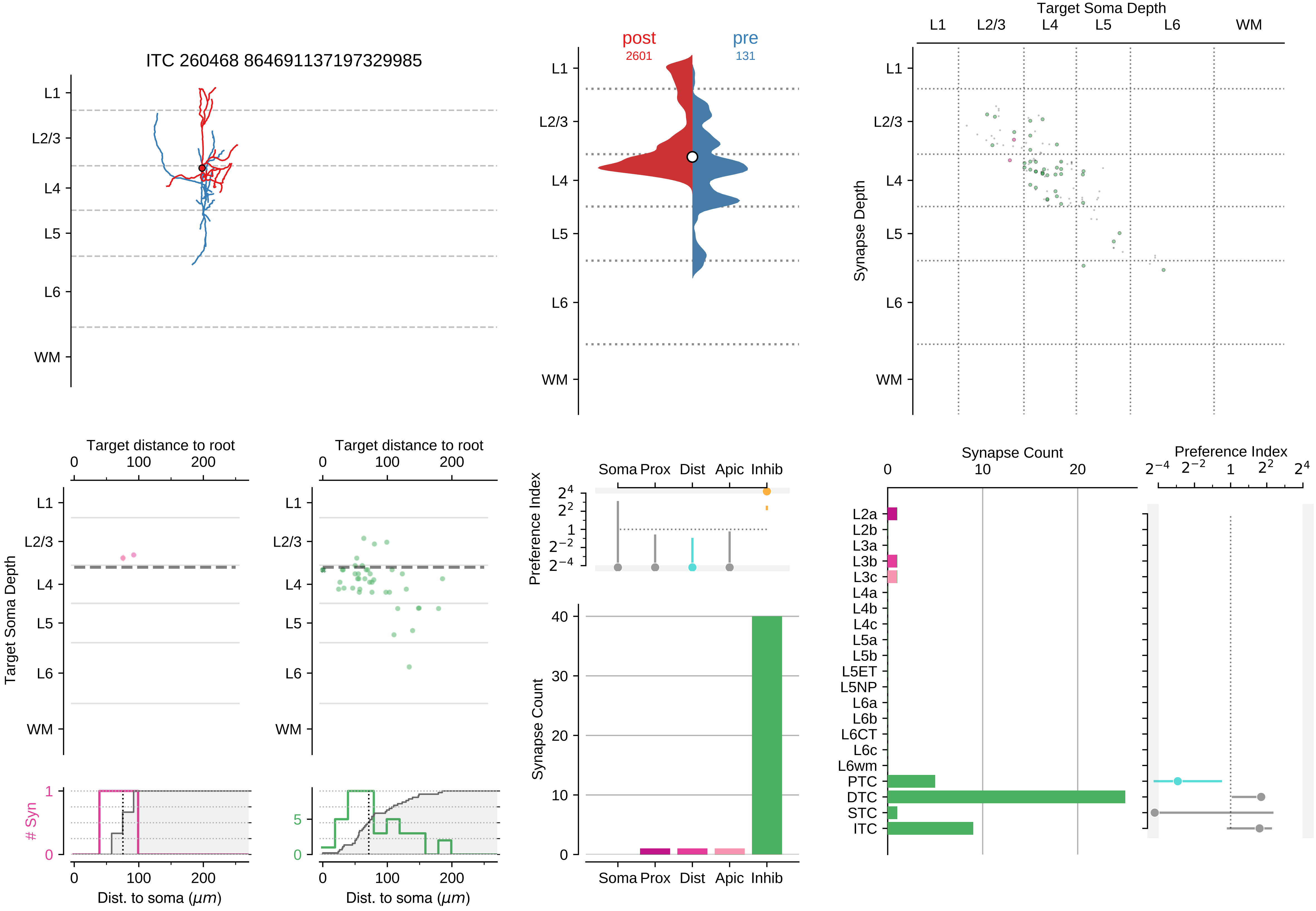

ITC 260468 864691137197329985
post
2601
pre
131
Target Soma Depth
Synapse Depth
L1
L2/3
L4
L5
L6
WM
Target distance to root
Target Soma Depth
Dist. to soma (μm)
Syn
Soma Prox Dist Apic Inhib
Preference Index
Synapse Count
L2a
L2b
L3a
L3b
L3c
L4a
L4b
L4c
L5a
L5b
L5ET
L5NP
L6a
L6b
L6CT
L6c
L6wm
PTC
DTC
STC
ITC

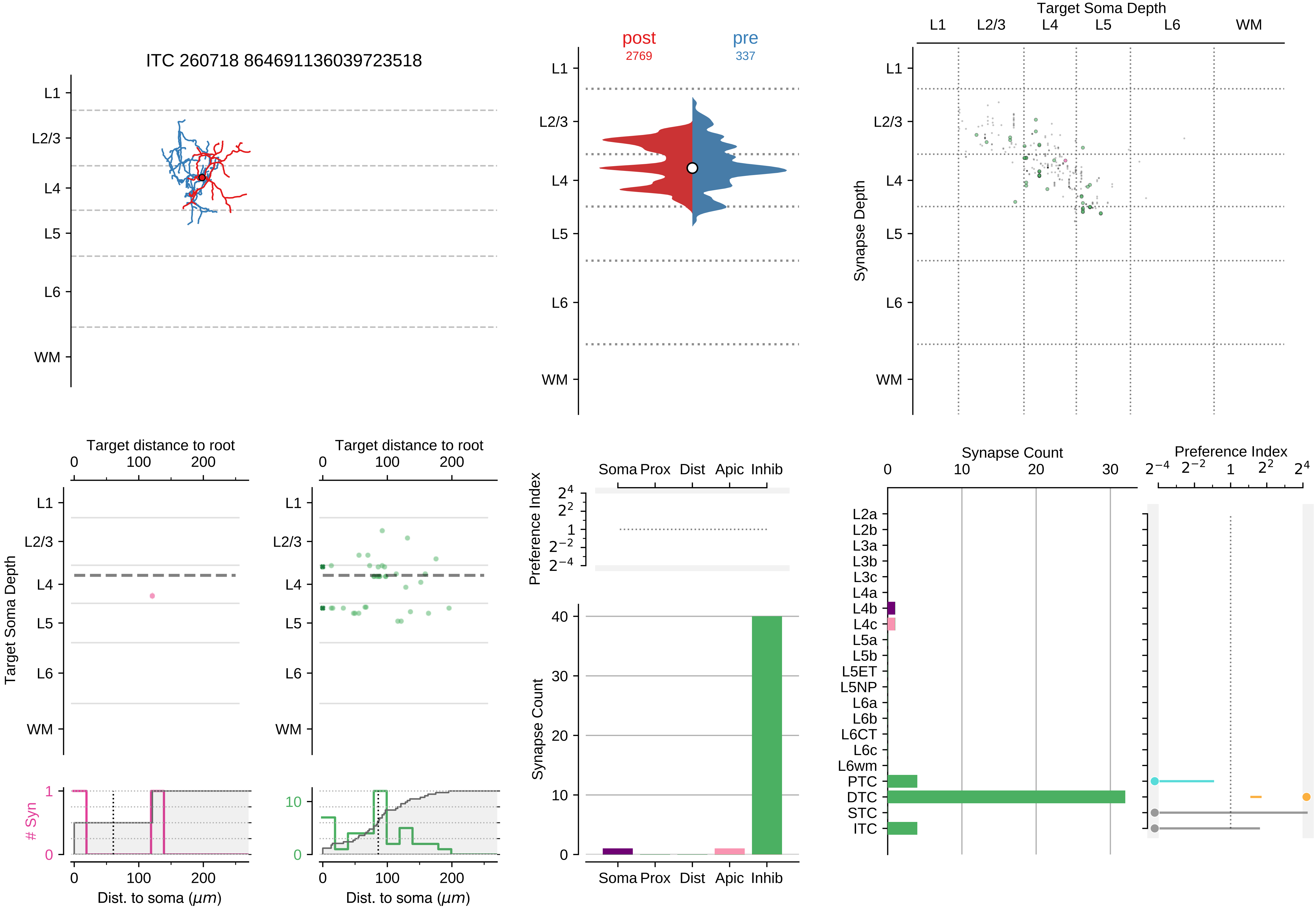

ITC 260718 864691136039723518
post
2769
pre
337
Target Soma Depth
Synapse Depth
L1
L2/3
L4
L5
L6
WM
Target distance to root
Target Soma Depth
Syn
Dist. to soma (μm)
Preference Index
Synapse Count
Soma Prox Dist Apic Inhib
L2a
L2b
L3a
L3b
L3c
L4a
L4b
L4c
L5a
L5b
L5ET
L5NP
L6a
L6b
L6CT
L6c
L6wm
PTC
DTC
STC
ITC

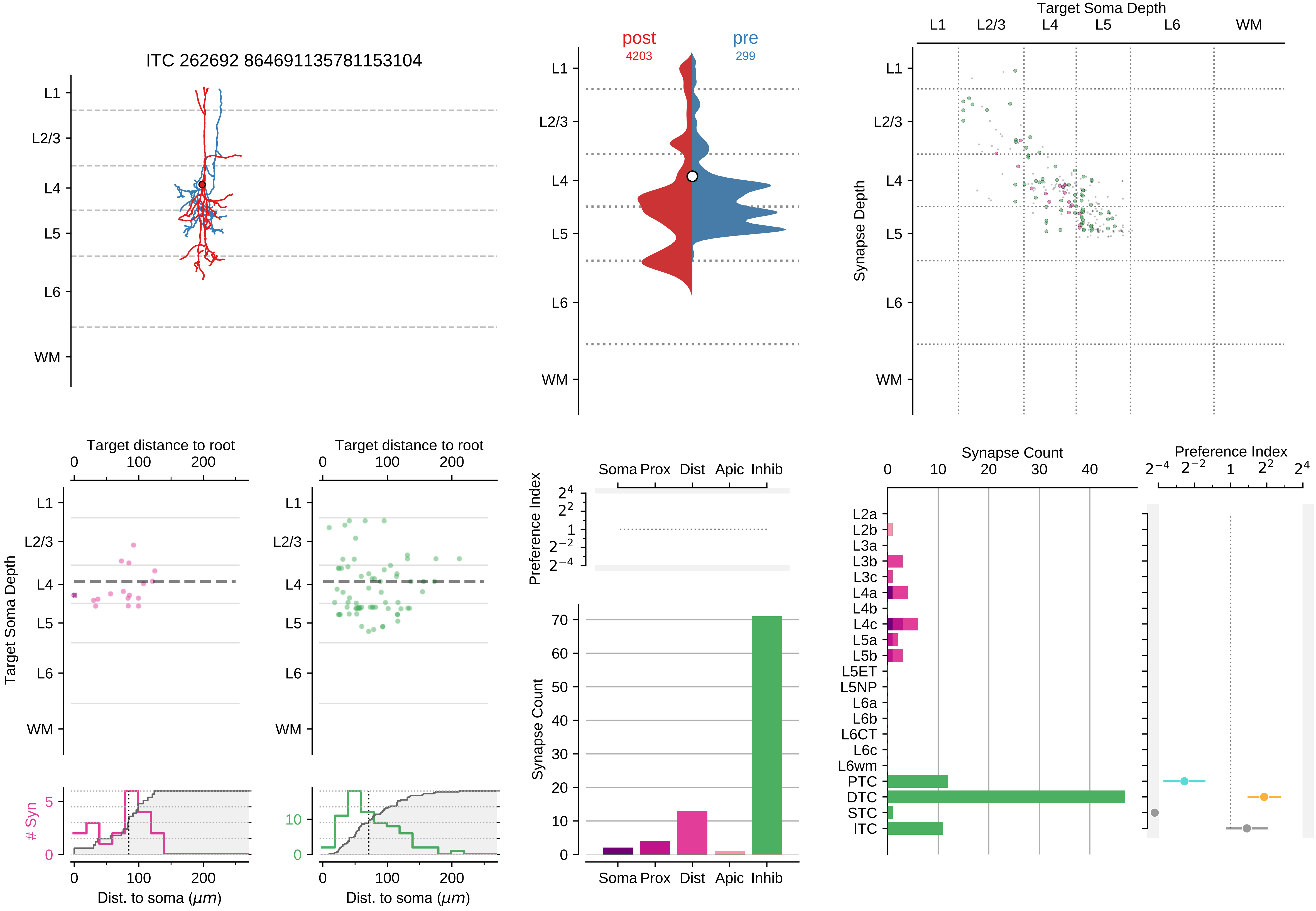

ITC 262692 864691135781153104
post
4203
pre
299
Target Soma Depth
Synapse Depth
L1
L2/3
L4
L5
L6
WM
Target distance to root
Target Soma Depth
Syn
Dist. to soma (μm)
Preference Index
Synapse Count
Soma Prox Dist Apic Inhib
L2a
L2b
L3a
L3b
L3c
L4a
L4b
L4c
L5a
L5b
L5ET
L5NP
L6a
L6b
L6CT
L6c
L6wm
PTC
DTC
STC
ITC

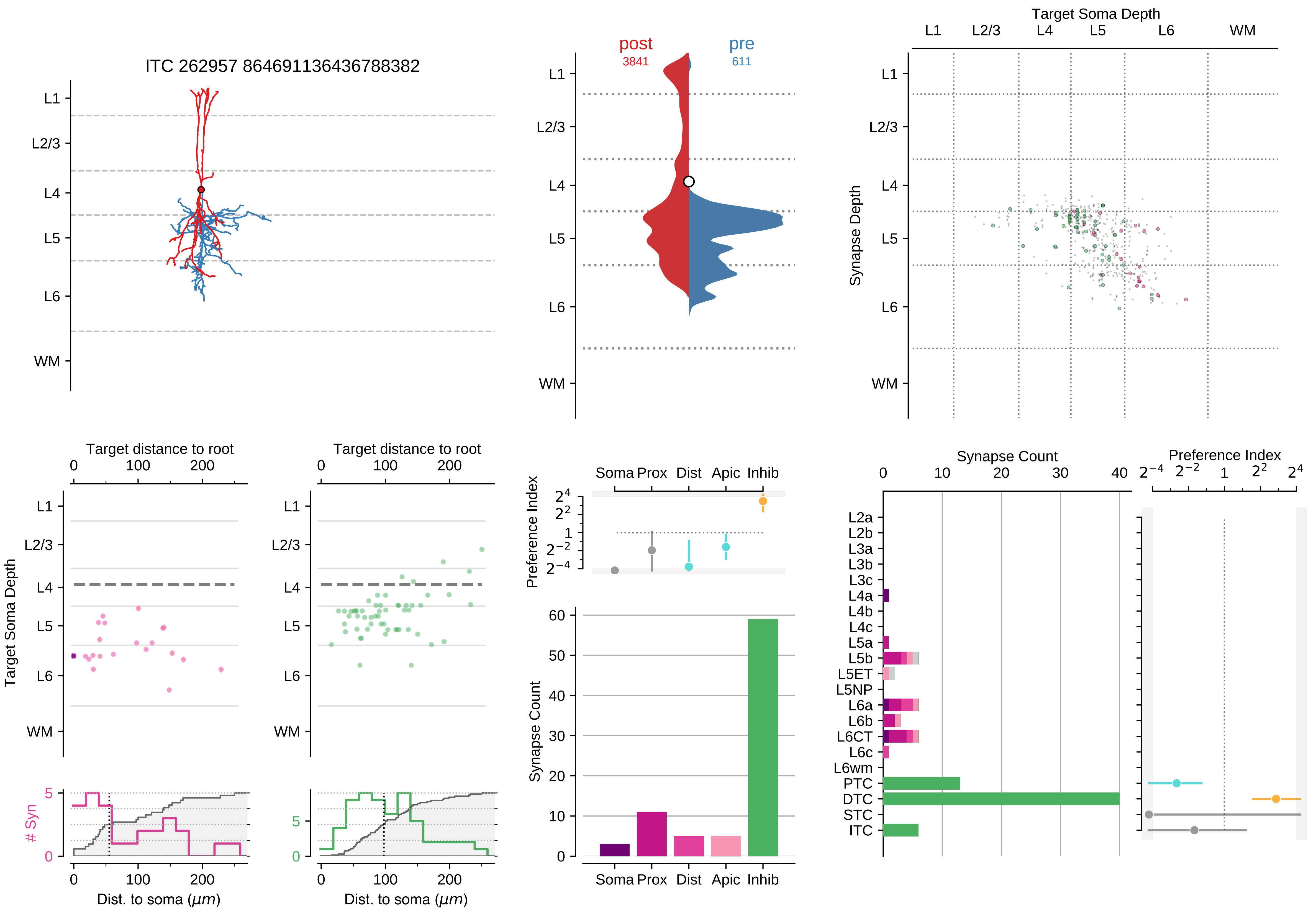

ITC 262957 864691136436788382
L1
L2/3
L4
L5
L6
WM
post
3841
pre
611
Target Soma Depth
Synapse Depth
Target distance to root
Target Soma Depth
Syn
Dist. to soma (μm)
Soma Prox Dist Apic Inhib
Preference Index
Synapse Count
L2a
L2b
L3a
L3b
L3c
L4a
L4b
L4c
L5a
L5b
L5ET
L5NP
L6a
L6b
L6CT
L6c
L6wm
PTC
DTC
STC
ITC

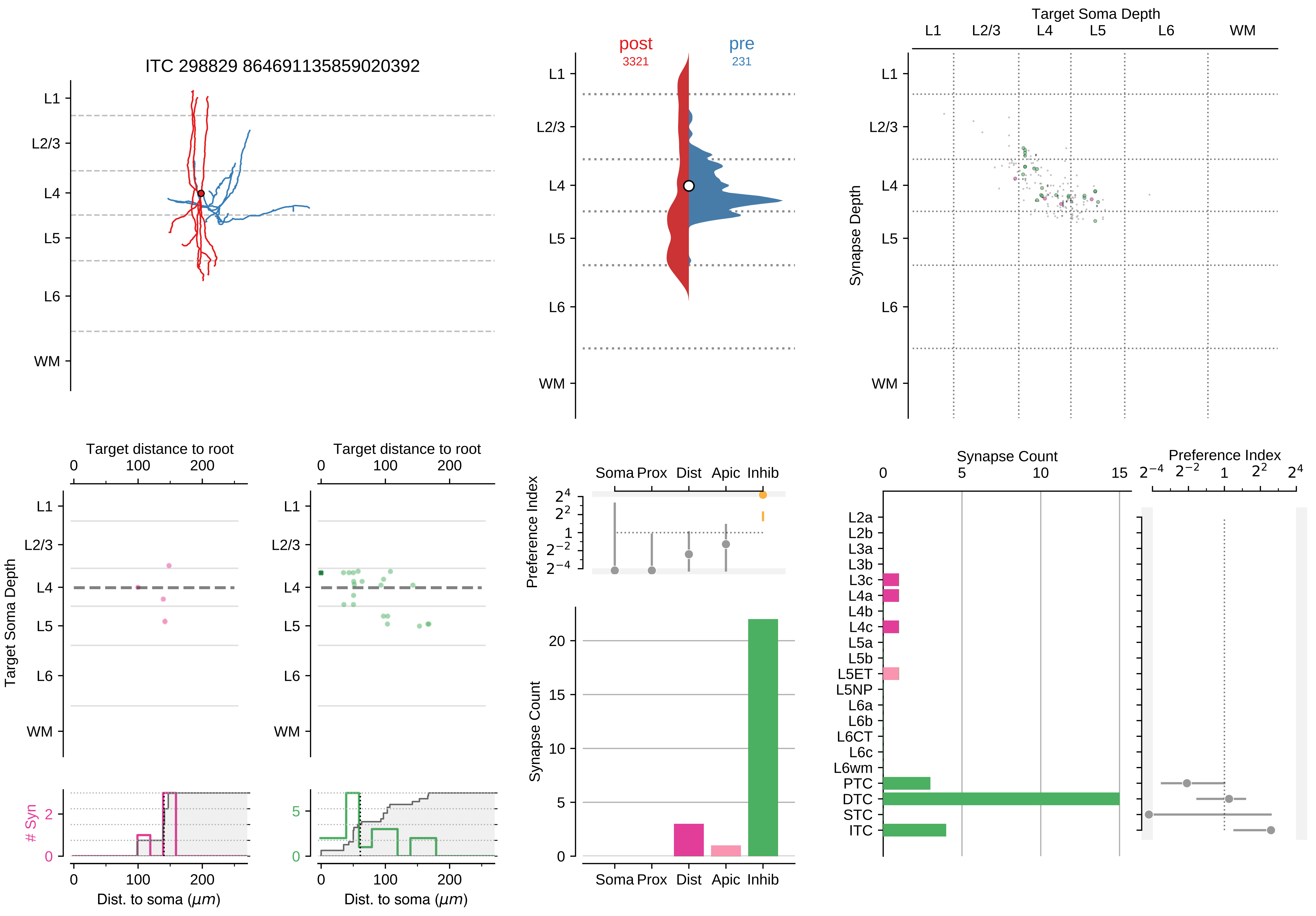

ITC 298829 864691135859020392
L1
L2/3
L4
L5
L6
WM
post
3321
pre
231
Target Soma Depth
Synapse Depth
Target distance to root
Target Soma Depth
Syn
Dist. to soma (μm)
Preference Index
Soma Prox Dist Apic Inhib
Synapse Count
Synapse Count
Preference Index
L2a
L2b
L3a
L3b
L3c
L4a
L4b
L4c
L5a
L5b
L5ET
L5NP
L6a
L6b
L6CT
L6c
L6wm
PTC
DTC
STC
ITC

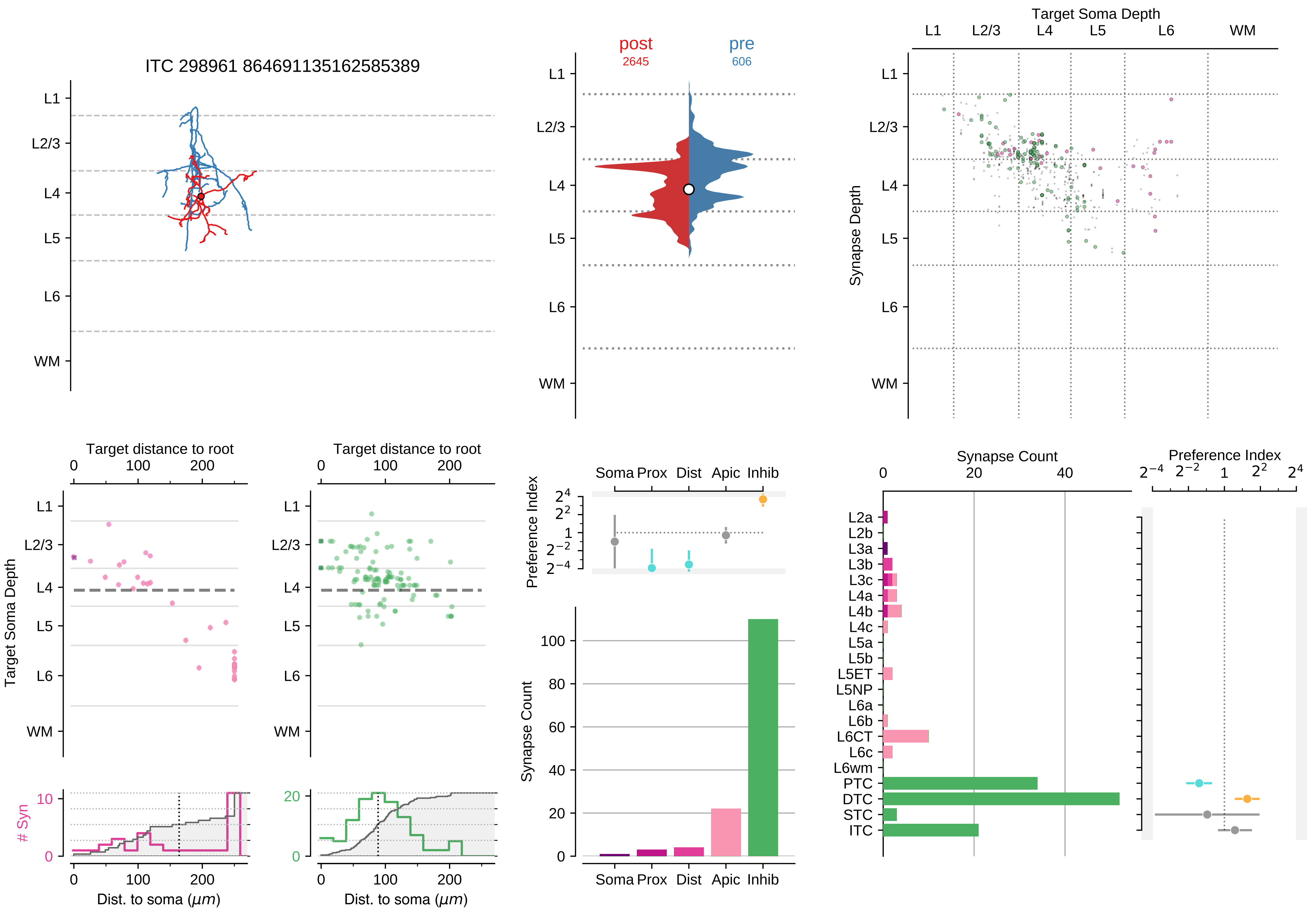
ITC 298961 864691135162585389
post
2645
pre
606
Target Soma Depth
Synapse Depth
L1
L2/3
L4
L5
L6
WM
Target distance to root
Target Soma Depth
Syn
Dist. to soma (μm)
Preference Index
Soma
Prox
Dist
Apic
Inhib
Synapse Count
L2a
L2b
L3a
L3b
L3c
L4a
L4b
L4c
L5a
L5b
L5ET
L5NP
L6a
L6b
L6CT
L6c
L6wm
PTC
DTC
STC
ITC

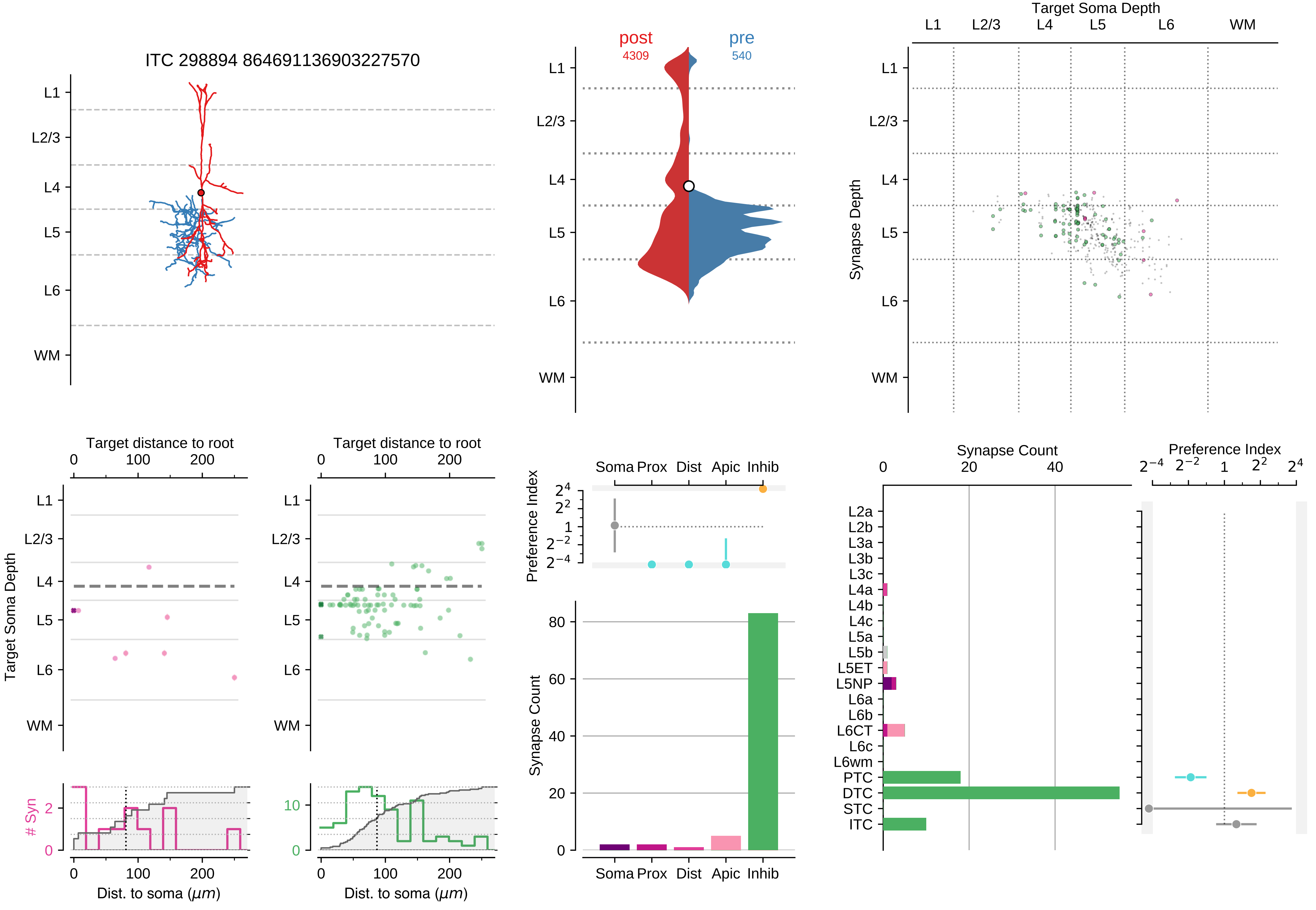

ITC 298894 864691136903227570
L1
L2/3
L4
L5
L6
WM
post
4309
pre
540
Target Soma Depth
Synapse Depth
Target distance to root
Target Soma Depth
Syn
Dist. to soma (μm)
Soma Prox Dist Apic Inhib
Preference Index
Synapse Count
L2a
L2b
L3a
L3b
L3c
L4a
L4b
L4c
L5a
L5b
L5ET
L5NP
L6a
L6b
L6CT
L6c
L6wm
PTC
DTC
STC
ITC

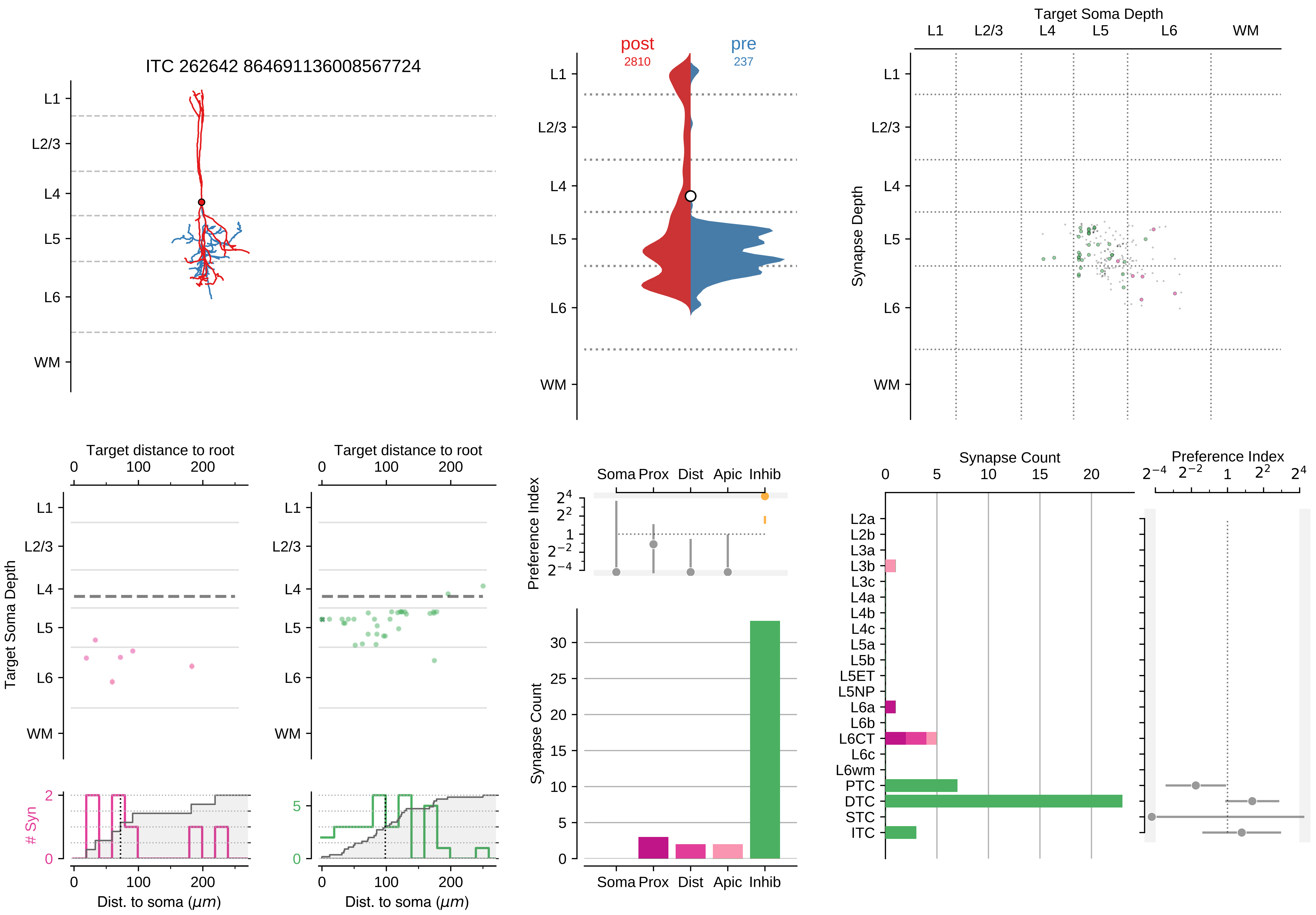
ITC 262642 864691136008567724
L1
L2/3
L4
L5
L6
WM
post
2810
pre
237
Target Soma Depth
Synapse Depth
Target distance to root
Target Soma Depth
Syn
Dist. to soma (μm)
Soma Prox Dist Apic Inhib
Preference Index
Synapse Count
L2a
L2b
L3a
L3b
L3c
L4a
L4b
L4c
L5a
L5b
L5ET
L5NP
L6a
L6b
L6CT
L6c
L6wm
PTC
DTC
STC
ITC

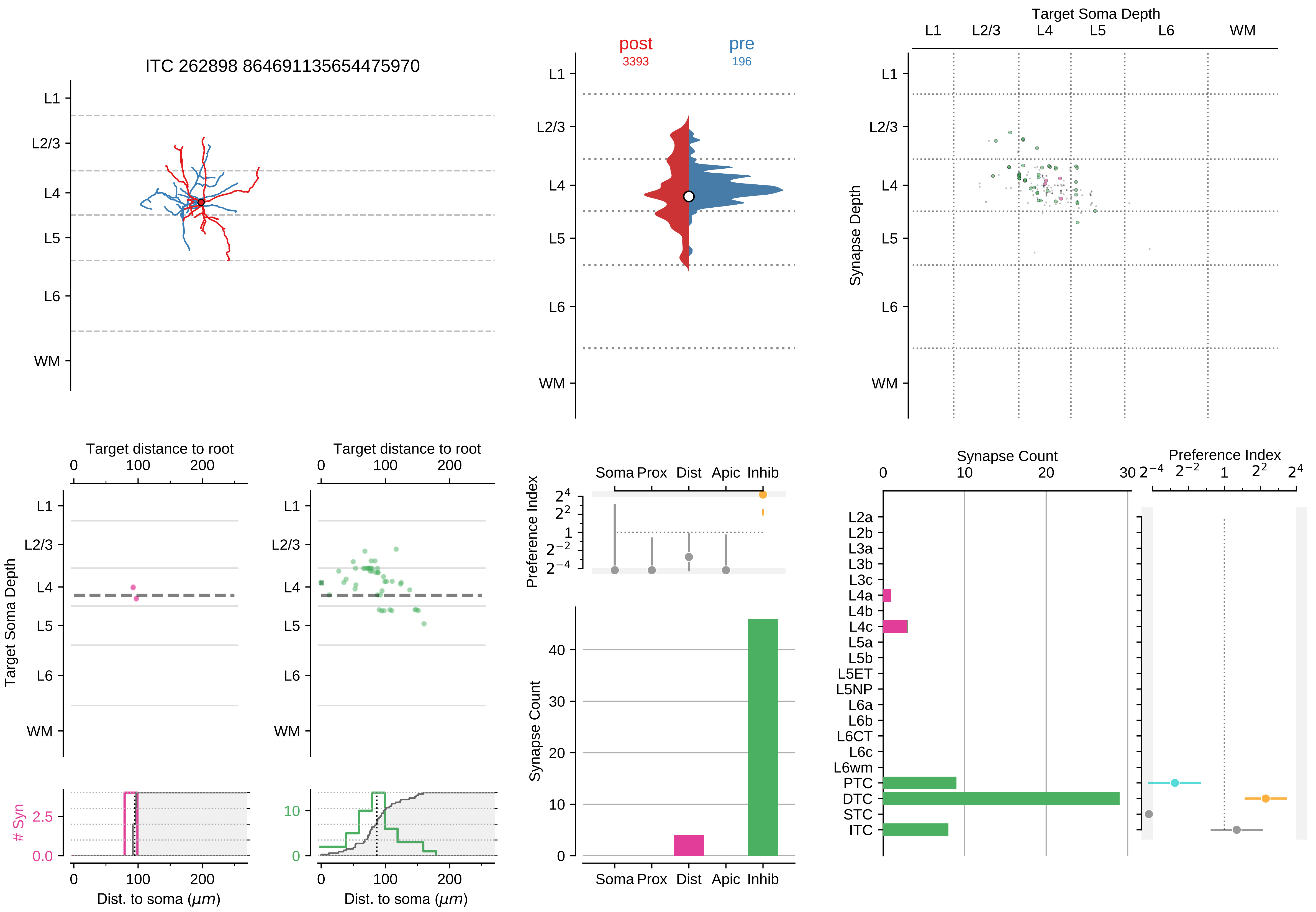

ITC 262898 864691135654475970
post
3393
pre
196
Target Soma Depth
Synapse Depth
L1
L2/3
L4
L5
L6
WM
Target distance to root
Target Soma Depth
Syn
Dist. to soma (μm)
Preference Index
Soma Prox Dist Apic Inhib
Synapse Count
Synapse Count
Preference Index
L2a
L2b
L3a
L3b
L3c
L4a
L4b
L4c
L5a
L5b
L5ET
L5NP
L6a
L6b
L6CT
L6c
L6wm
PTC
DTC
STC
ITC

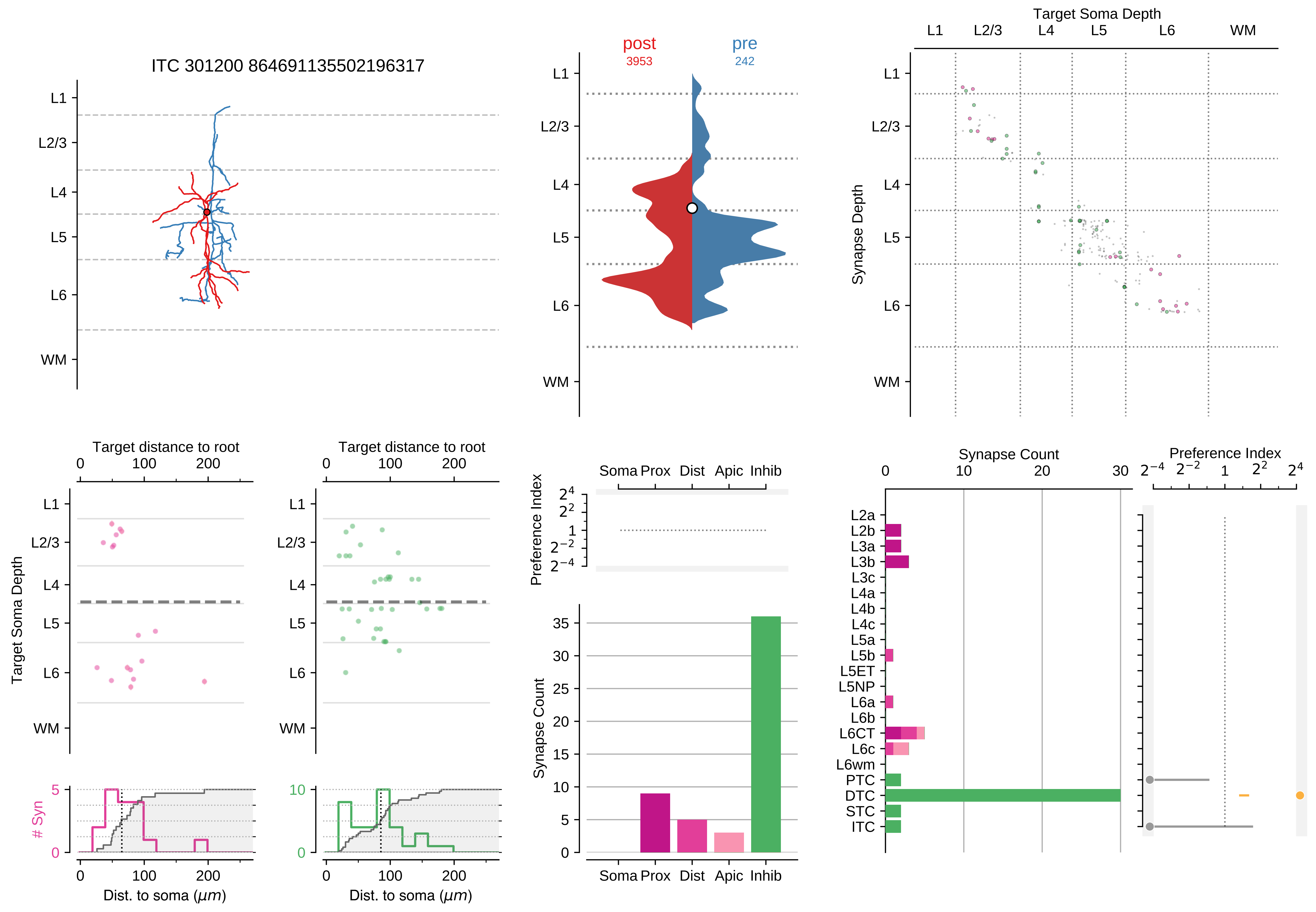

ITC 301200 864691135502196317
L1
L2/3
L4
L5
L6
WM
post
3953
pre
242
Target Soma Depth
Synapse Depth
Target distance to root
Target Soma Depth
Syn
Dist. to soma (μm)
Soma Prox Dist Apic Inhib
Preference Index
Synapse Count
L2a
L2b
L3a
L3b
L3c
L4a
L4b
L4c
L5a
L5b
L5ET
L5NP
L6a
L6b
L6CT
L6c
L6wm
PTC
DTC
STC
ITC

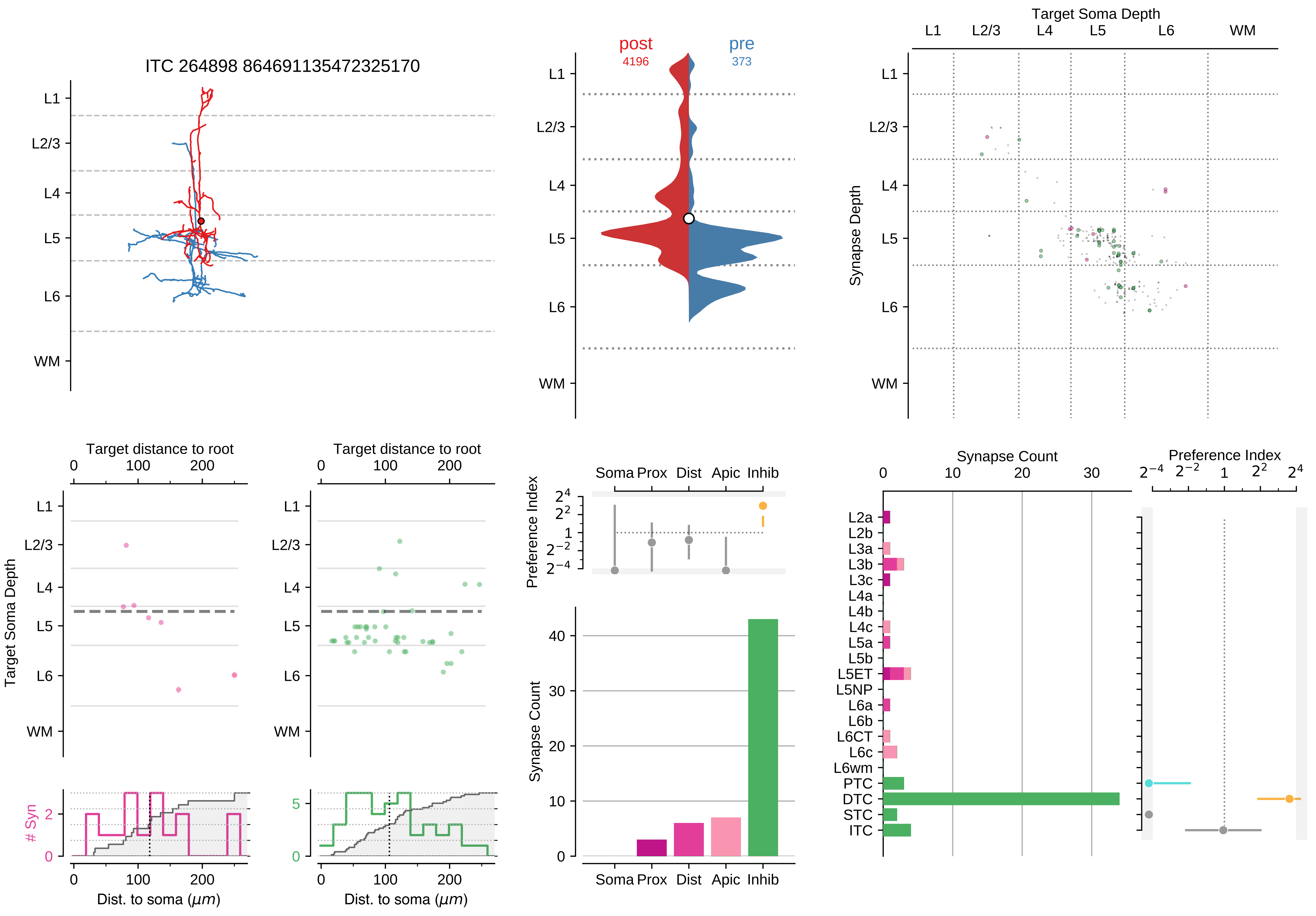

ITC 264898 864691135472325170
L1
L2/3
L4
L5
L6
WM
post
4196
pre
373
Target Soma Depth
Synapse Depth
Target distance to root
Target Soma Depth
Syn
Dist. to soma (μm)
Preference Index
Soma Prox Dist Apic Inhib
Synapse Count
L2a
L2b
L3a
L3b
L3c
L4a
L4b
L4c
L5a
L5b
L5ET
L5NP
L6a
L6b
L6CT
L6c
L6wm
PTC
DTC
STC
ITC

Group 20 ($InhTC^{prox}$)

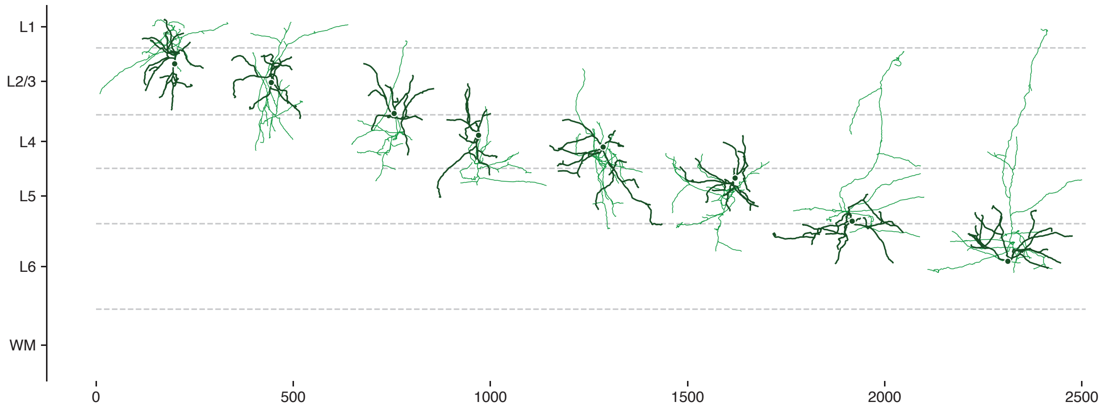

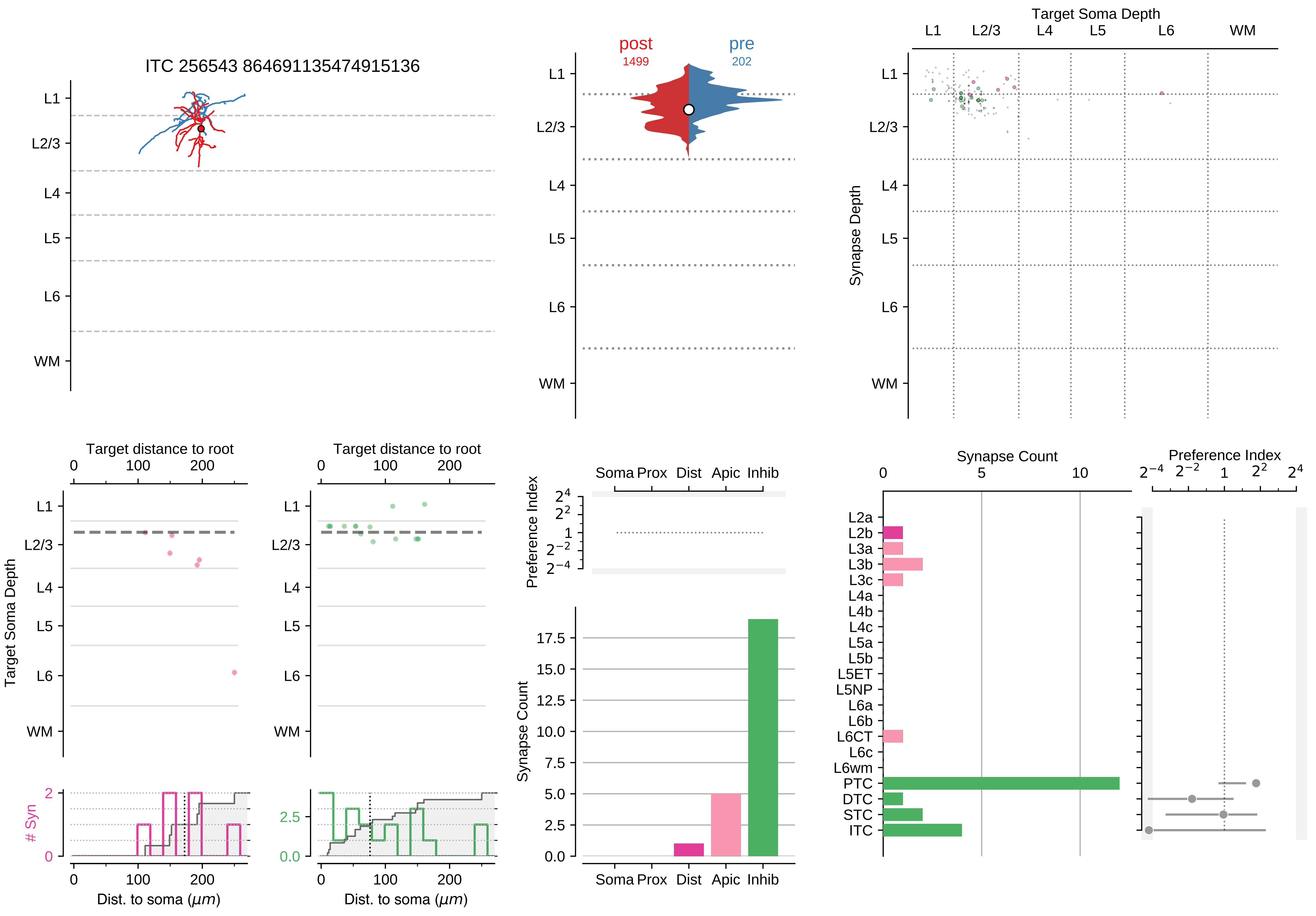
ITC 256543 864691135474915136
post
1499
pre
202
Target Soma Depth
Synapse Depth
L1
L2/3
L4
L5
L6
WM
Target distance to root
Target Soma Depth
Syn
Dist. to soma (µm)
Preference Index
Soma Prox Dist Apic Inhib
Synapse Count
Preference Index
L2a
L2b
L3a
L3b
L3c
L4a
L4b
L4c
L5a
L5b
L5ET
L5NP
L6a
L6b
L6CT
L6c
L6wm
PTC
DTC
STC
ITC

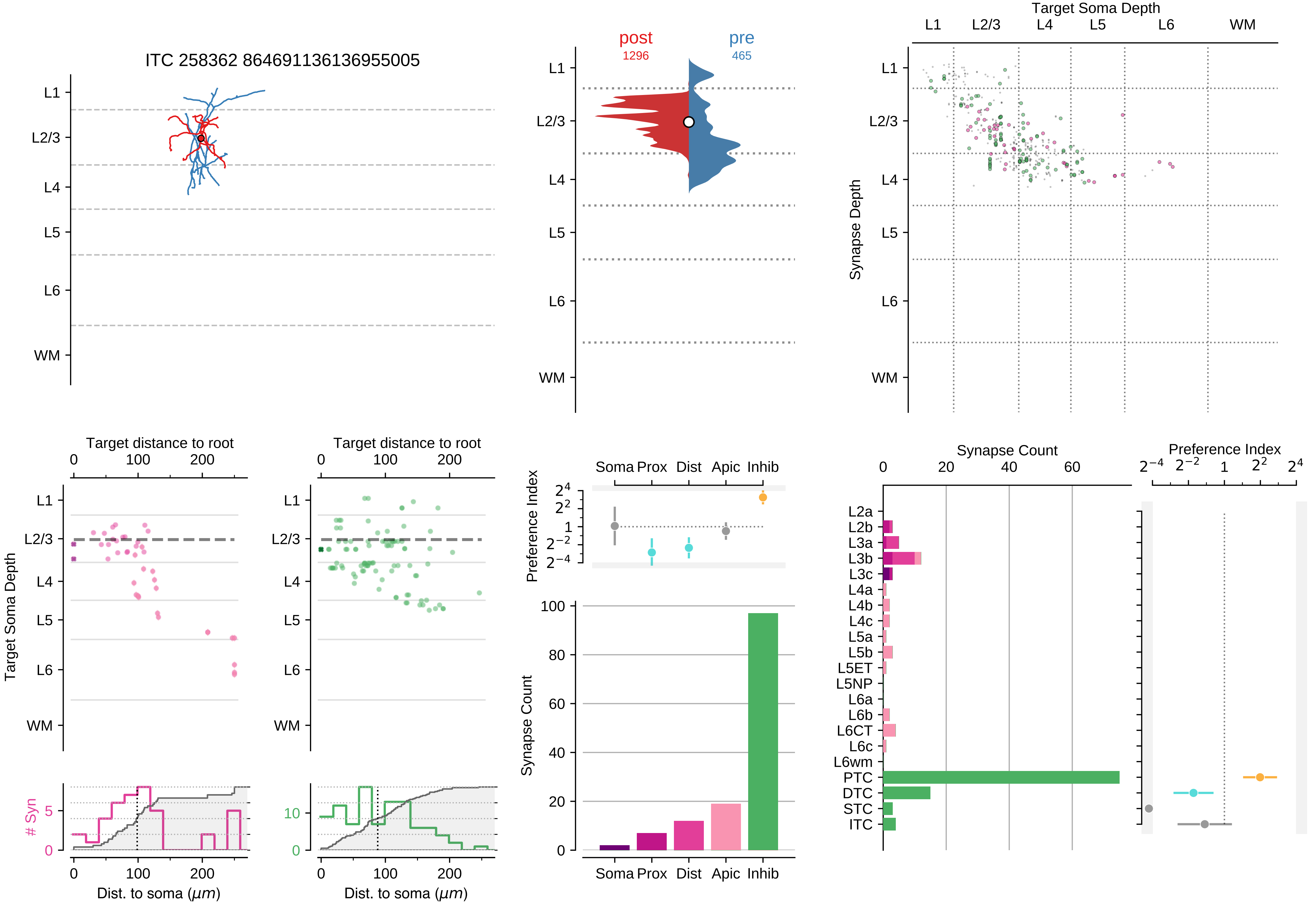

ITC 258362 864691136136955005
L1
L2/3
L4
L5
L6
WM
post
1296
pre
465
Target Soma Depth
Synapse Depth
Target distance to root
Target Soma Depth
Dist. to soma (μm)
Syn
Preference Index
Soma
Prox
Dist
Apic
Inhib
Synapse Count
L2a
L2b
L3a
L3b
L3c
L4a
L4b
L4c
L5a
L5b
L5ET
L5NP
L6a
L6b
L6CT
L6c
L6wm
PTC
DTC
STC
ITC

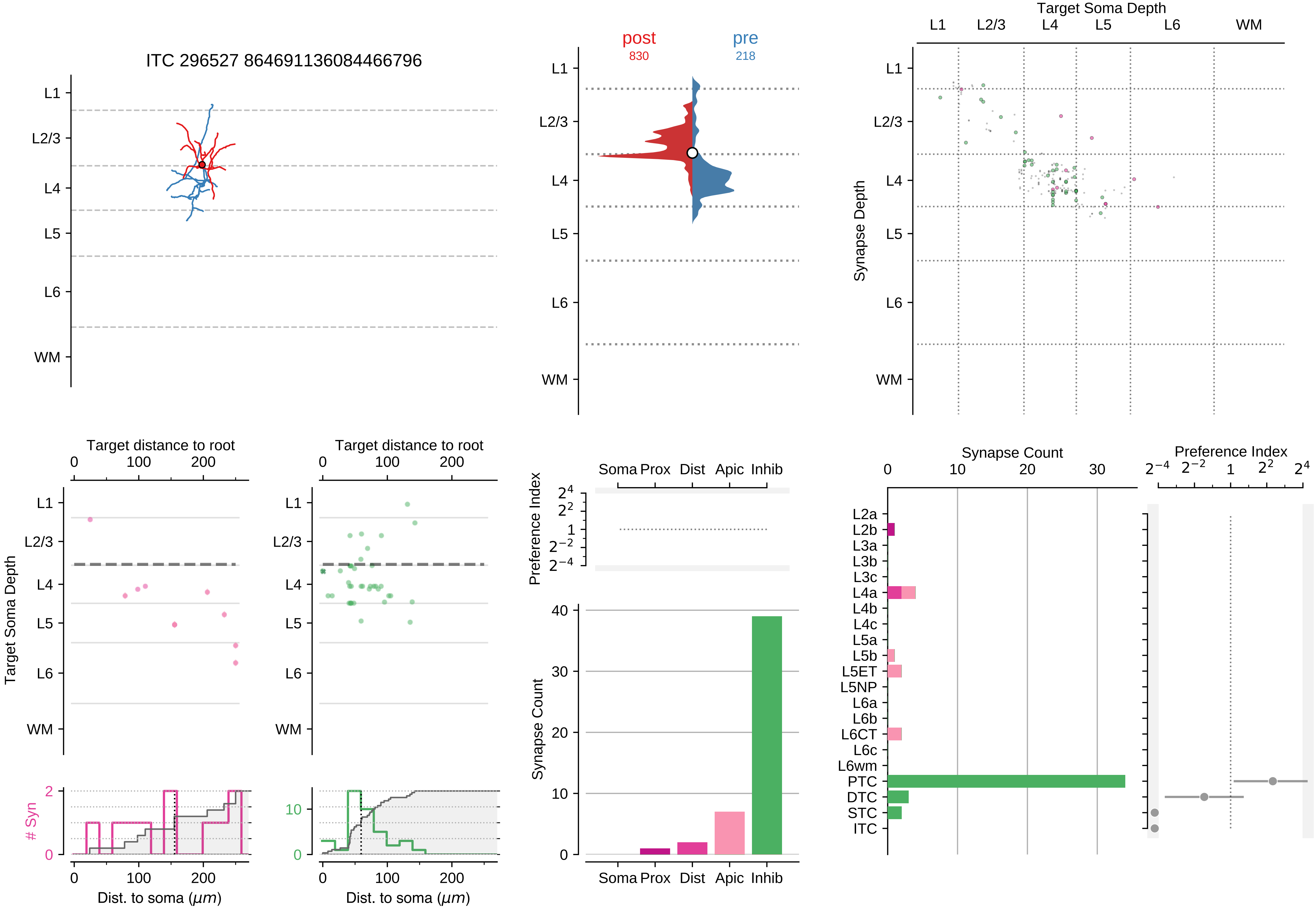

ITC 296527 864691136084466796
L1
L2/3
L4
L5
L6
WM
post
830
pre
218
Target Soma Depth
Synapse Depth
Target distance to root
Target Soma Depth
Syn
Dist. to soma (μm)
Preference Index
Synapse Count
Soma
Prox
Dist
Apic
Inhib
L2a
L2b
L3a
L3b
L3c
L4a
L4b
L4c
L5a
L5b
L5ET
L5NP
L6a
L6b
L6CT
L6c
L6wm
PTC
DTC
STC
ITC

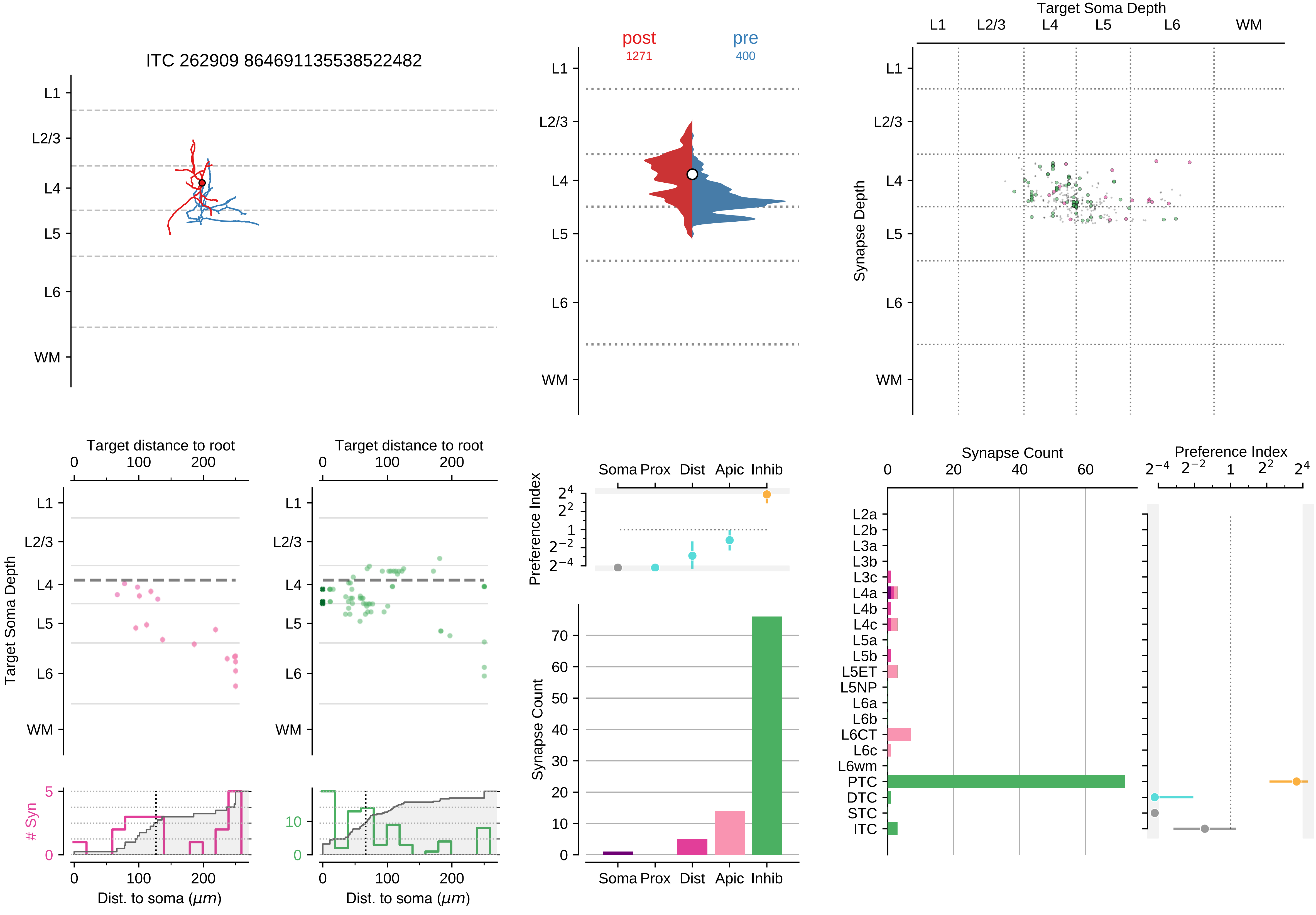

ITC 262909 864691135538522482
L1
L2/3
L4
L5
L6
WM
post
1271
pre
400
Target Soma Depth
Synapse Depth
Target distance to root
Target Soma Depth
Syn
Dist. to soma (μm)
Preference Index
Soma Prox Dist Apic Inhib
Synapse Count
Synapse Count
Preference Index
L2a
L2b
L3a
L3b
L3c
L4a
L4b
L4c
L5a
L5b
L5ET
L5NP
L6a
L6b
L6CT
L6c
L6wm
PTC
DTC
STC
ITC

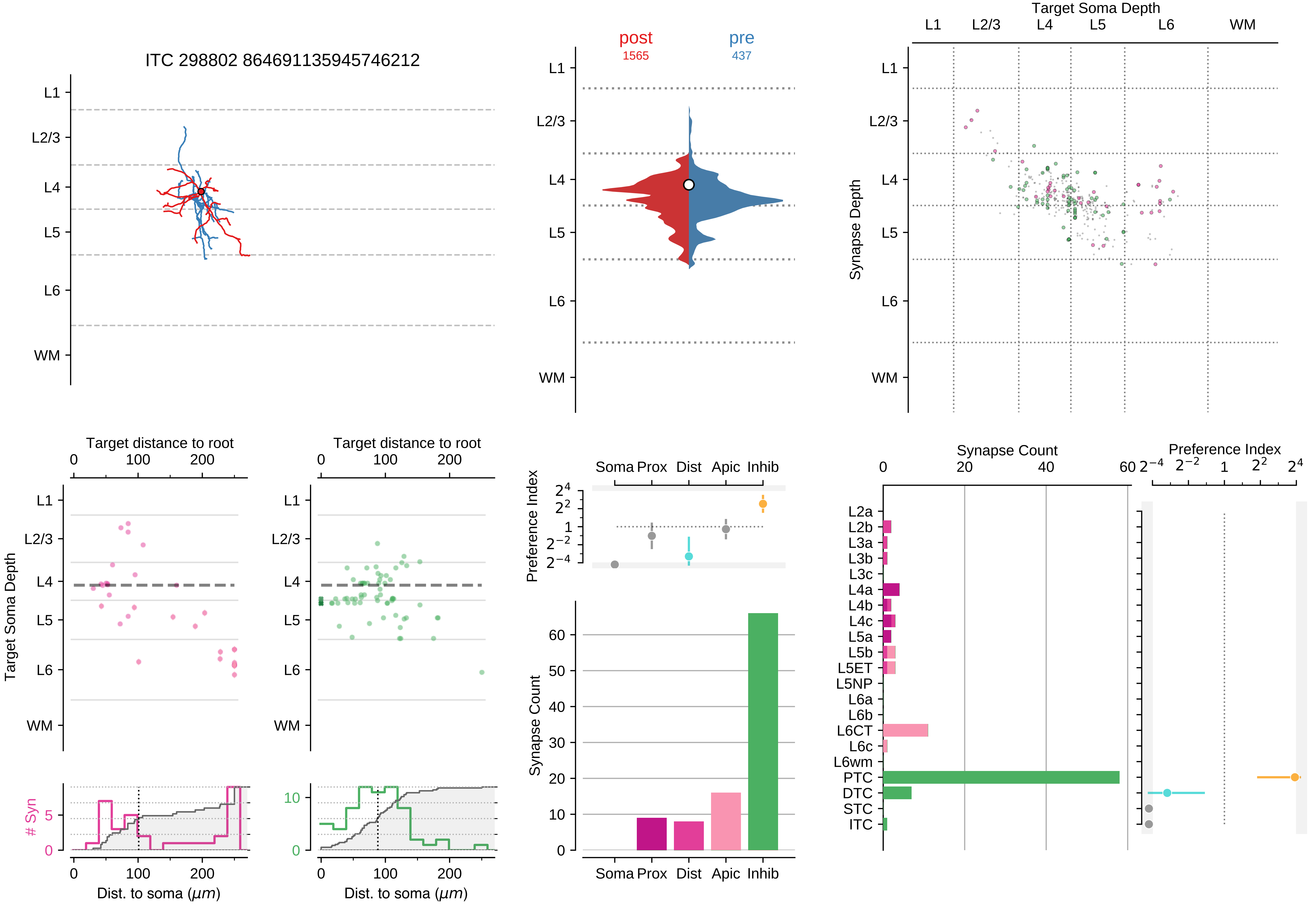
ITC 298802 864691135945746212
L1
L2/3
L4
L5
L6
WM
post
1565
pre
437
Target Soma Depth
Synapse Depth
Target distance to root
Target Soma Depth
Syn
Dist. to soma (μm)
Soma Prox Dist Apic Inhib
Preference Index
Synapse Count
L2a
L2b
L3a
L3b
L3c
L4a
L4b
L4c
L5a
L5b
L5ET
L5NP
L6a
L6b
L6CT
L6c
L6wm
PTC
DTC
STC
ITC

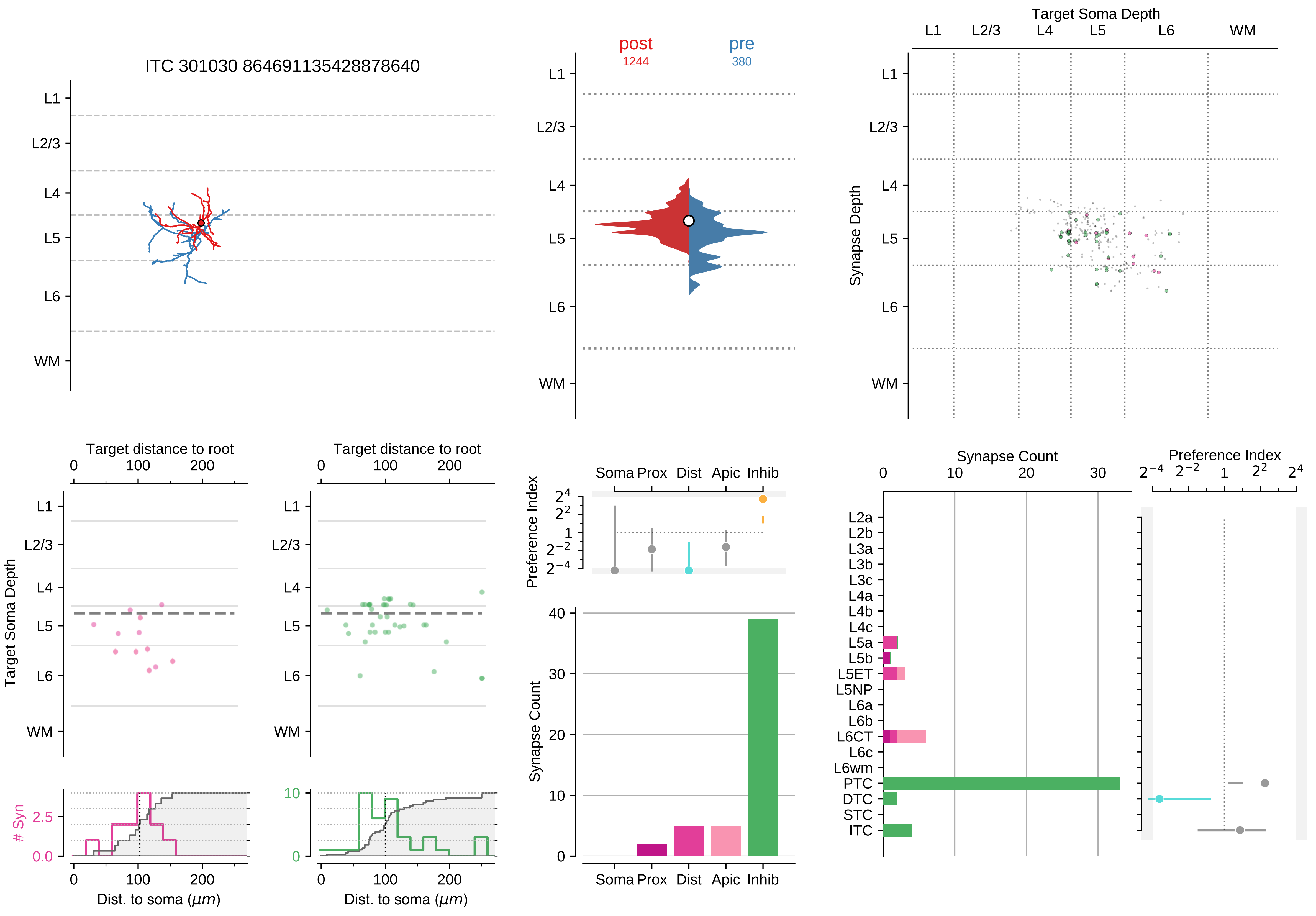

ITC 301030 864691135428878640
L1
L2/3
L4
L5
L6
WM
post
1244
pre
380
Target Soma Depth
Synapse Depth
Target distance to root
Target Soma Depth
Syn
Dist. to soma (μm)
Preference Index
Soma Prox Dist Apic Inhib
Synapse Count
L2a
L2b
L3a
L3b
L3c
L4a
L4b
L4c
L5a
L5b
L5ET
L5NP
L6a
L6b
L6CT
L6c
L6wm
PTC
DTC
STC
ITC

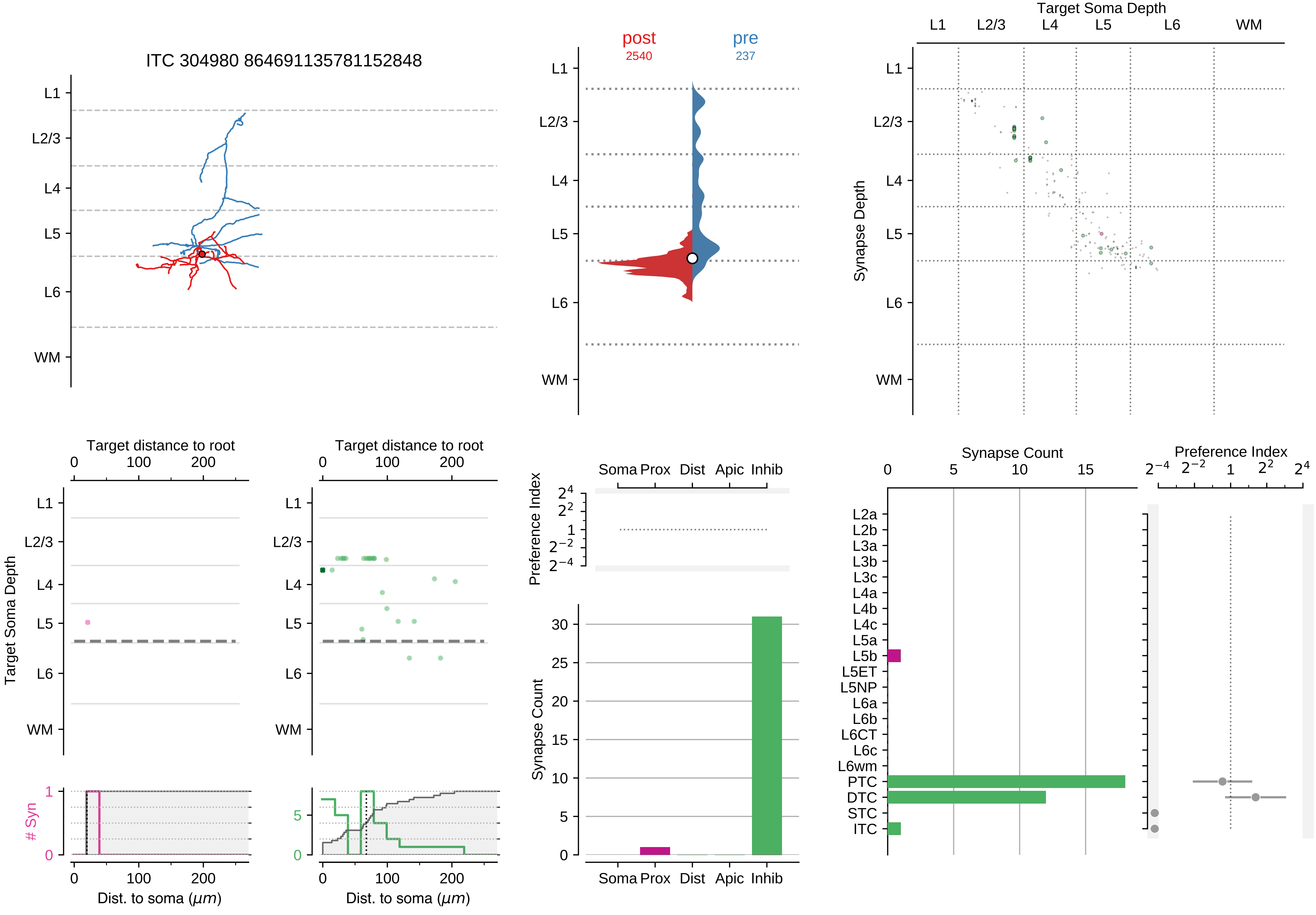

ITC 304980 864691135781152848
L1
L2/3
L4
L5
L6
WM
post
2540
pre
237
Target Soma Depth
Synapse Depth
Target distance to root
0
100
200
Target Soma Depth
Syn
Dist. to soma (μm)
Soma
Prox
Dist
Apic
Inhib
Preference Index
Synapse Count
L2a
L2b
L3a
L3b
L3c
L4a
L4b
L4c
L5a
L5b
L5ET
L5NP
L6a
L6b
L6CT
L6c
L6wm
PTC
DTC
STC
ITC

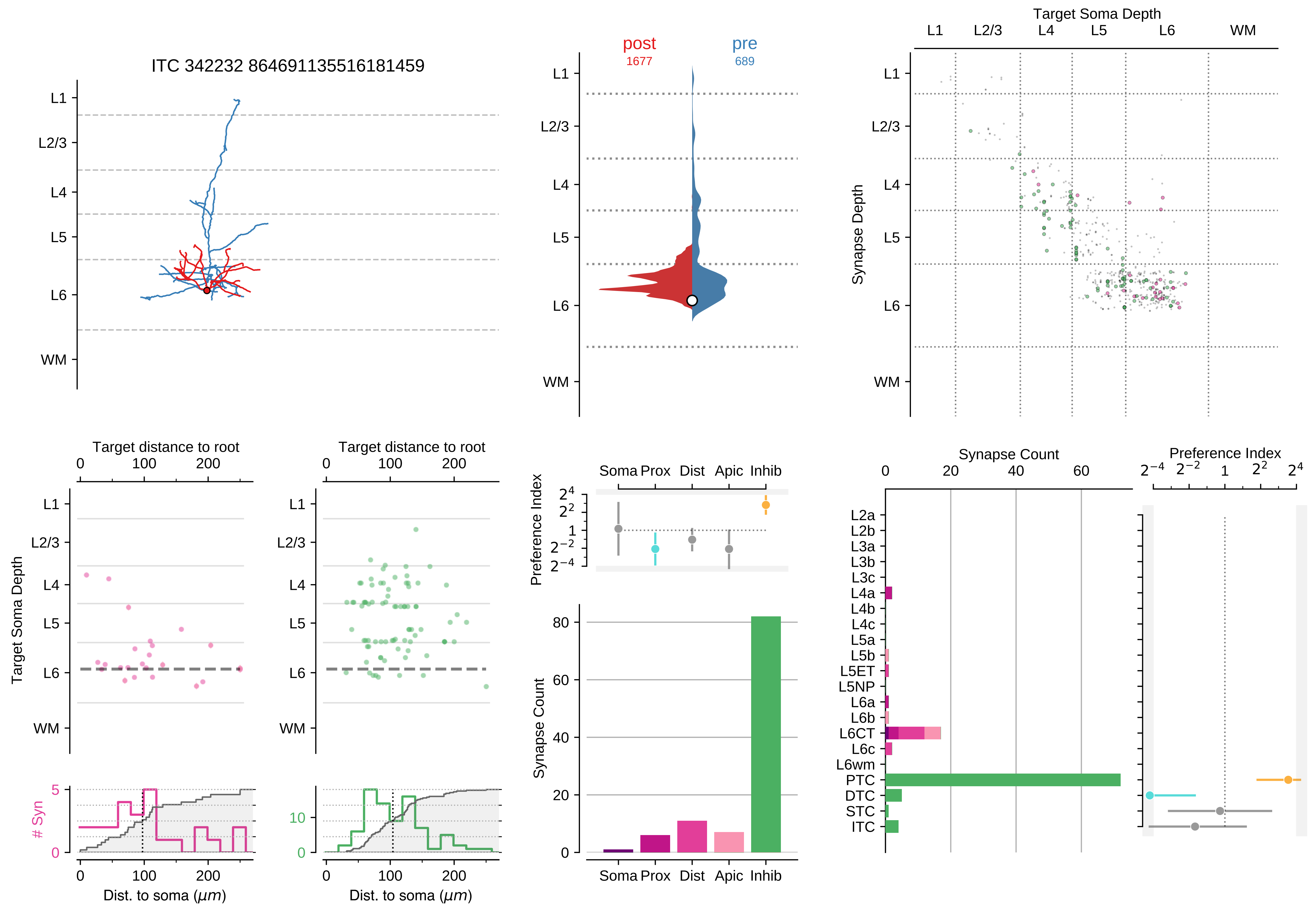

ITC 342232 864691135516181459
post
1677
pre
689
Target Soma Depth
Synapse Depth
L1
L2/3
L4
L5
L6
WM
Target distance to root
Target Soma Depth
Syn
Dist. to soma (μm)
Preference Index
Soma Prox Dist Apic Inhib
Synapse Count
L2a
L2b
L3a
L3b
L3c
L4a
L4b
L4c
L5a
L5b
L5ET
L5NP
L6a
L6b
L6CT
L6c
L6wm
PTC
DTC
STC
ITC

Unclassified (too few synapses in column)

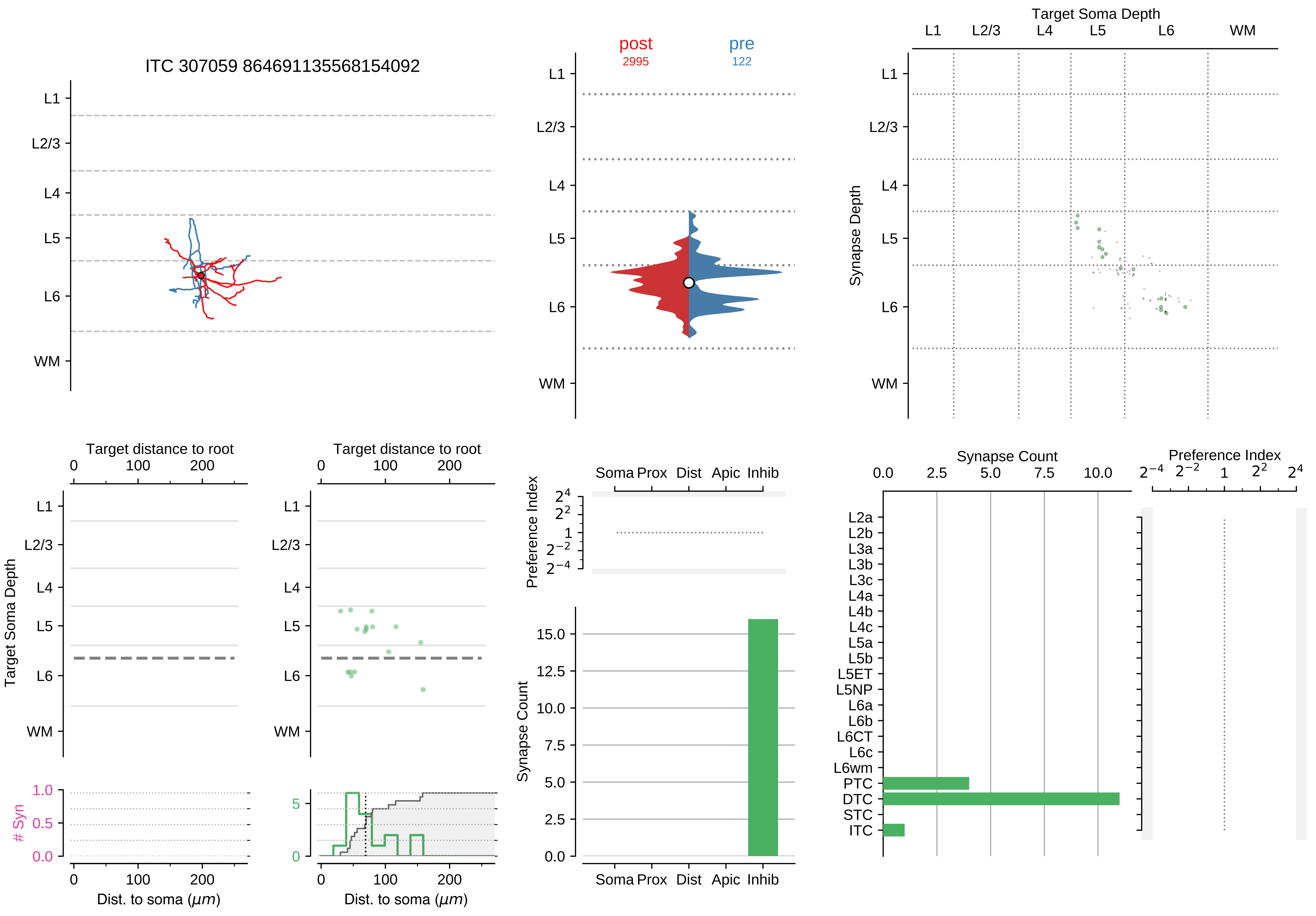
ITC 307059 864691135568154092
L1
L2/3
L4
L5
L6
WM
post
2995
pre
122
Target Soma Depth
Synapse Depth
Target distance to root
Target Soma Depth
Syn
Dist. to soma (μm)
Preference Index
Soma Prox Dist Apic Inhib
Synapse Count
Synapse Count
Preference Index
L2a
L2b
L3a
L3b
L3c
L4a
L4b
L4c
L5a
L5b
L5ET
L5NP
L6a
L6b
L6CT
L6c
L6wm
PTC
DTC
STC
ITC
